# Convergent Evolution of Cysteine-Rich Keratins in Hard Skin Appendages of Terrestrial Vertebrates

**DOI:** 10.1093/molbev/msz279

**Published:** 2019-12-10

**Authors:** Florian Ehrlich, Julia Lachner, Marcela Hermann, Erwin Tschachler, Leopold Eckhart

**Affiliations:** 1 Research Division of Biology and Pathobiology of the Skin, Department of Dermatology, Medical University of Vienna, Vienna, Austria; 2 Department of Medical Biochemistry, Medical University of Vienna, Vienna, Austria

**Keywords:** keratin, convergent evolution, gene family, co-option, feathers

## Abstract

Terrestrial vertebrates have evolved hard skin appendages, such as scales, claws, feathers, and hair that play crucial roles in defense, predation, locomotion, and thermal insulation. The mechanical properties of these skin appendages are largely determined by cornified epithelial components. So-called “hair keratins,” cysteine-rich intermediate filament proteins that undergo covalent cross-linking via disulfide bonds, are the crucial structural proteins of hair and claws in mammals and hair keratin orthologs are also present in lizard claws, indicating an evolutionary origin in a hairless common ancestor of amniotes. Here, we show that reptiles and birds have also other cysteine-rich keratins which lack cysteine-rich orthologs in mammals. In addition to hard acidic (type I) sauropsid-specific (HAS) keratins, we identified hard basic (type II) sauropsid-specific (HBS) keratins which are conserved in lepidosaurs, turtles, crocodilians, and birds. Immunohistochemical analysis with a newly made antibody revealed expression of chicken HBS1 keratin in the cornifying epithelial cells of feathers. Molecular phylogenetics suggested that the high cysteine contents of HAS and HBS keratins evolved independently from the cysteine-rich sequences of hair keratin orthologs, thus representing products of convergent evolution. In conclusion, we propose an evolutionary model in which HAS and HBS keratins evolved as structural proteins in epithelial cornification of reptiles and at least one HBS keratin was co-opted as a component of feathers after the evolutionary divergence of birds from reptiles. Thus, cytoskeletal proteins of hair and feathers are products of convergent evolution and evolutionary co-option to similar biomechanical functions in clade-specific hard skin appendages.

## Introduction

Keratin intermediate filaments form the cytoskeleton of vertebrate epithelia (Peter and Stick 2015; Jacob et al. 2018). The basic unit of these filaments is a heterodimer of a type I (“acidic”) and a type II (“basic”) keratin. All vertebrates have multiple keratins of both types, with, for example, 16 type I and 7 type II keratin genes being present in the zebrafish (Krushna Padhi et al. 2006) and 28 type I and 26 type II keratins in humans (Schweizer et al. 2006). Differential expression of individual keratins leads to different compositions of the cytoskeleton in the various epithelia. Each epithelial cell contains only a limited number of keratins and typically a pair of one type I and one type II keratin forms the predominant keratin heterodimer. Stem cell identity, proliferation, and differentiation of epithelial cells are associated with characteristic keratin expression, facilitating the adaptation of the cytoskeleton to various levels of intracellular dynamics and exposure to external stress.

Terrestrial life of amniotes (reptiles, birds, and mammals) was associated with the evolution of hard skin appendages such as scales, claws, feathers, and hair (Chuong and Homberger 2003; Wu et al. 2004; Alibardi 2016; Eckhart and Ehrlich 2018; Pan et al. 2019). These appendages of the integument are characterized by rigid and mechanically resistant epithelial components in which keratinocytes acquire a heavily cross-linked cytoskeleton and remain tightly connected to each other after cell death, that is, cornification (Eckhart et al. 2013). By contrast, the epidermis between skin appendages contains only a thin layer of more flexible cornified cells, also known as stratum corneum.

Sequence analysis has shown that keratins expressed in hair and claws/nails of mammals and in claws of reptiles contain high amounts of cysteine residues which form disulfide bonds to other proteins and thereby establish the rigid cytoskeleton that is crucial for the mechanical resistance of the skin appendages (Wang et al. 2000; Perrin et al. 2004; Langbein and Schweizer 2005; Eckhart et al. 2008; Strnad et al. 2011; Fraser and Parry 2018). Comparative genomics and gene expression studies indicate that these keratins, originally termed “hair keratins,” have originated in a common ancestor of amniotes in which their primordial function was likely the stabilization of the corneous material of claws (Eckhart et al. 2008). Type I and type II “hair keratins” are conserved in mammals and lizards (Eckhart et al. 2008; Vandebergh and Bossuyt 2012), but their numbers and composition are only partially known in many clades of amniotes. However, some hair keratin orthologs have been lost in snakes, turtles, and birds (Dalla Valle et al. 2011). The loss of hair (claw) keratins in snakes can be attributed to the loss of claws, and the evolution of corneous beta-proteins (CBPs) as quantitatively dominant components of reptilian and avian skin appendages, may have made type I hair keratins dispensable in turtles and birds (Dalla Valle et al. 2011). Of note, CBPs are also called beta-keratins but lack an intermediate filament domain and therefore cannot fully substitute true keratins (Holthaus et al. 2018). Furthermore, a group of hard skin appendage-associated cysteine-rich type I (acidic) keratins were identified specifically in reptiles and birds (sauropsids), leading us to propose the term “hard acidic sauropsid-specific” (HAS) keratins for these bona fide keratins that are not orthologous to type I hair keratins (Eckhart et al. 2008). It is still unknown whether sauropsids also have cysteine-rich type II (basic) keratins different from orthologs of mammalian hair keratins.

Here, we screened genome sequences of sauropsids for genes encoding cysteine-rich keratins. We identified homologs of HAS keratins and a previously uncharacterized group of cysteine-rich type II keratins that are conserved in all clades of sauropsids. Comparative genomics and results of gene expression analyses suggest a model in which the evolution of high cysteine contents in different subgroups of keratins was instrumental in the evolution of hard skin appendages, including feathers.

## Results

### Orthologs of Hair Keratins Are Conserved in Some but Not All Sauropsids

**FIg. 1.**
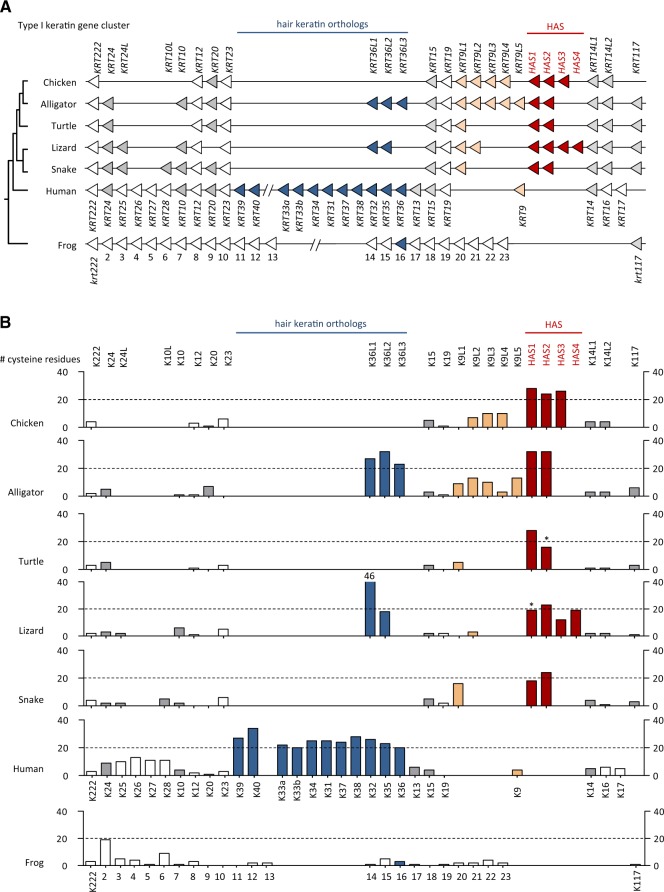
Comparative analysis of the type I keratin gene cluster in terrestrial vertebrates. (*A*) Map of the type I keratin gene clusters in representative species of the main clades of terrestrial vertebrates. Genes are symbolized by arrowheads that point in the direction of transcription. Putative orthology is indicated by equal colors. Note that *KRT222* encodes a keratin-like protein with an incomplete intermediate filament domain. (*B*) Cysteine counts of proteins encoded by the genes shown in panel (*A*). Asterisks mark keratins for which only incomplete amino acid sequences were available and the real number of cysteine residues is probably higher than indicated by the respective bars. Lizard K36L1 has 46 cysteine residues which is higher than the maximum value of this graph. Species: Chicken (*Gallus gallus*), alligator (*Alligator sinensis*), turtle (*Chrysemys picta*), lizard (*Anolis carolinensis*), snake (*Protobothrops mucrosquamatus*), frog (*Xenopus tropicalis*).

**FIg. 2.**
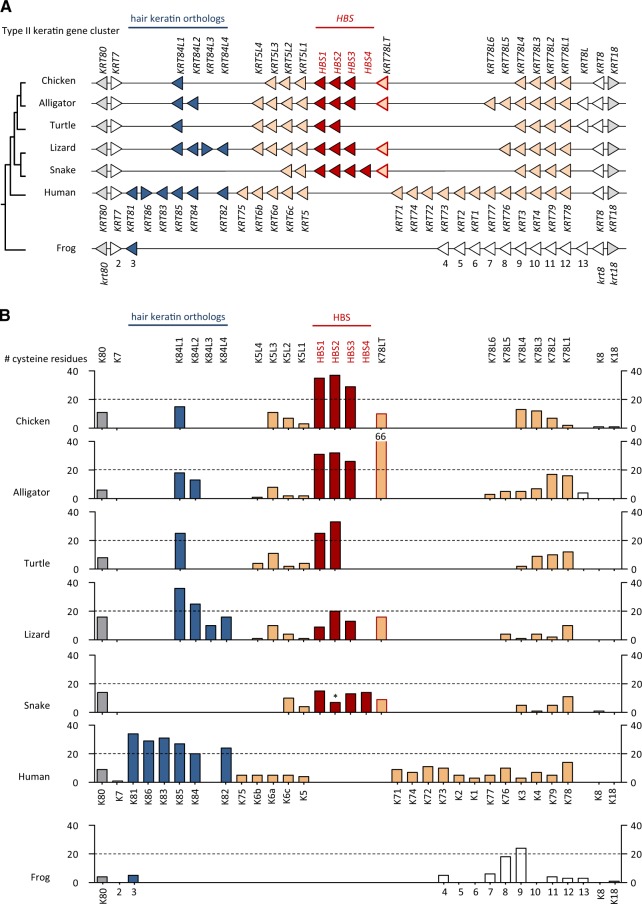
Comparative analysis of the type II keratin gene cluster in terrestrial vertebrates. (*A*) Map of the type II keratin gene clusters in representative species of the main clades of terrestrial vertebrates. Genes are symbolized by arrowheads that point in the direction of transcription. Putative orthology is indicated by equal colors. Note that *KRT18* encodes a type I keratin. (*B*) Cysteine counts of proteins encoded by the genes shown in panel (*A*). An asterisk over snake HBS2 indicates that only an incomplete amino acid sequence was available and the number of cysteine residues is probably higher than indicated. Alligator K78LT has 66 cysteine residues which is higher than the maximum value of this graph. Information about the species is provided in the legend of figure 1.

To determine the distribution of cysteine-rich keratins among vertebrates, we analyzed gene loci syntenic to mammalian type I and type II keratin gene clusters in genomes of birds (*Gallus gallus*, *Taeniopygia guttata*, *Struthio camelus australis*, *Apteryx australis*), alligators (*Alligator sinensis*, *Alligator mississippiensis*), turtles (*Chrysemys picta bellii*), lizard (*Anolis carolinensis*), and snakes (*Protobothrops mucrosquamatus*, *Python bivittatus*), amphibians (*Xenopus laevis*, *Xenopus* *tropicalis*, *Nanorana parkeri*), and fishes (*Danio rerio*, *Takifugu rubripes*, *Oncorhynchus mykiss*). Additional keratins were predicted from transcriptome data of the axolotl (*Ambystoma mexicanum*) and a caecilian (*Microcaecilia unicolor*). In mammals such as humans, type I keratin genes *KRT9* through *KRT40* are localized in one gene cluster (fig. 1) and all type II keratin genes (*KRT1*–*KRT8* and *KRT71*–*KRT86*) and *KRT18* (type I) are localized in another gene cluster (fig. 2). We also subjected assembled and unassembled genome sequence scaffolds of vertebrates to screening by basic logical alignment tool (BLAST) for hypothetical further keratin genes outside of these main keratin gene clusters. Our analysis confirmed the arrangement of keratins in two gene clusters in vertebrates (Vandebergh et al. 2013) and suggested only few changes in gene organization, as compared with the current GenBank gene annotations, within keratin type I and type II gene clusters (supplementary tables S1–S14, Supplementary Material online). The keratin open reading frames were translated into amino acid sequences (supplementary figs. S1 and S2, Supplementary Material online) and the number of cysteine residues was determined for all keratin proteins. The organization of keratin genes in the two clusters and the numbers of cysteine residue per encoded protein were compared between sauropsids, mammals, and amphibians (figs. 1 and 2).

The number of keratin genes in sauropsids varies from 16 to 22 for type I and from 14 to 20 type II keratin genes (figs. 1 and 2). In each reptile and bird species investigated, the total number of keratins genes is smaller than the number of keratin genes in the human genome. However, we also identified a keratin gene, which we termed *Krt117*, that is conserved in fishes, amphibians, and reptiles but not in mammals and birds, suggesting that gene loss contributed to the shaping of the mammalian and avian keratin repertoires (supplementary fig. S3, Supplementary Material online). Of note, the exon–intron organization of *Krt117* differs from that of all other type I keratins identified so far (supplementary fig. S3, Supplementary Material online) and the Xenopus ortholog of *Krt117*, previously termed *krt70*, is expressed during embryonic development but not in adult epidermis (Suzuki et al. 2017).

In mammals, only type I and II hair keratins contain 20 or more cysteine residues per protein (human keratins: figs. 1 and 2, platypus keratins: supplementary table S7, Supplementary Material online). Eleven type I and six type II hair keratin genes are present in the human genome. By contrast, only two and three type I hair keratin orthologs are present in the green anole lizard and the alligator, respectively, and snakes and turtles entirely lack orthologs of type I hair keratins (fig. 1). Similarly, the number of type II hair keratin orthologs is much smaller in sauropsids than in humans, with n = 2 in alligator, n = 1 in the turtle, n = 4 in the lizard, and n = 0 in snakes (fig. 2). Among birds, the number of hair keratins ranges from n = 1 (type I) and n = 1 (type II) in the kiwi (*Apteryx australis mantelli*) (supplementary table S8, Supplementary Material online) to n = 0 (type I) and n = 1 (type II) in the chicken, and n = 0 (type I) and n = 0 (type II) in the ostrich (supplementary table S9, Supplementary Material online) and the zebra finch (supplementary table S10, Supplementary Material online), suggesting that these keratins were differentially conserved during the evolutionary diversification of birds. Only two lizard hair keratin orthologs and one turtle hair keratin ortholog have 20 or more cysteine residues (fig. 2). The comparably low number of hair keratin orthologs in sauropsids suggests that other cysteine-rich intermediate filament proteins may exist to support the structure of hard skin appendages of reptiles and birds.

### Sauropsid Type I and Type II Keratins Unrelated to Hair Keratins Have Acquired High Cysteine Contents by Convergent Evolution

Besides orthologs of hair keratin genes, we identified one group of genes encoding other cysteine-rich keratins in each type I and II keratin gene cluster of sauropsids. Within the type I (acidic) keratin gene cluster, two to four genes encoding keratins with more than 20 cysteine residues are localized on the 3′-side of the *KRT14* gene in representatives of all major clades of sauropsids. These cysteine-rich type I keratins of the green anole lizard were previously termed HAS keratins (Eckhart et al. 2008). Three HAS keratin gene orthologs are present in birds (fig. 1 and supplementary tables S8–S10, Supplementary Material online) and two in alligator, turtle, and snake (fig. 1). In the type II (basic) keratin gene clusters of sauropsids, two to four genes encoding cysteine-rich keratins, here termed “hard basic sauropsid-specific” (HBS) keratins, were identified between *KRT5*-like and *KRT78*-like genes (fig. 2*A*). All HBS keratins of archelosaurs (turtles, crocodilians, and birds) have more than 20 cysteine residues whereas HBS orthologs of lepidosaurs (lizard and snakes) have a lower cysteine content (fig. 2*B*).

**FIg. 3.**
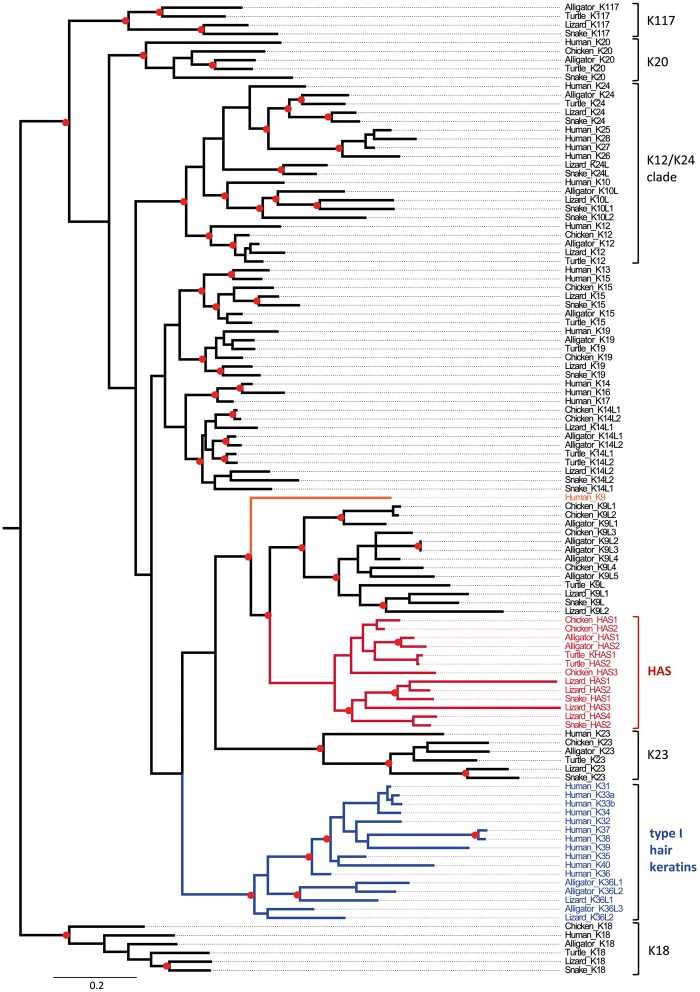
Phylogenetic analysis of type I keratins. A combined evolutionary model of maximum likelihood (ML) and Bayesian analysis of type I keratins is shown. Red circles indicate groups with bootstrap ≥75% obtained by ML analysis and posterior probability ≥0.95 obtained by Bayesian analysis. The scale bar represents substitutions/site. HAS, hard acidic sauropsid-specific keratin.

**FIg. 4.**
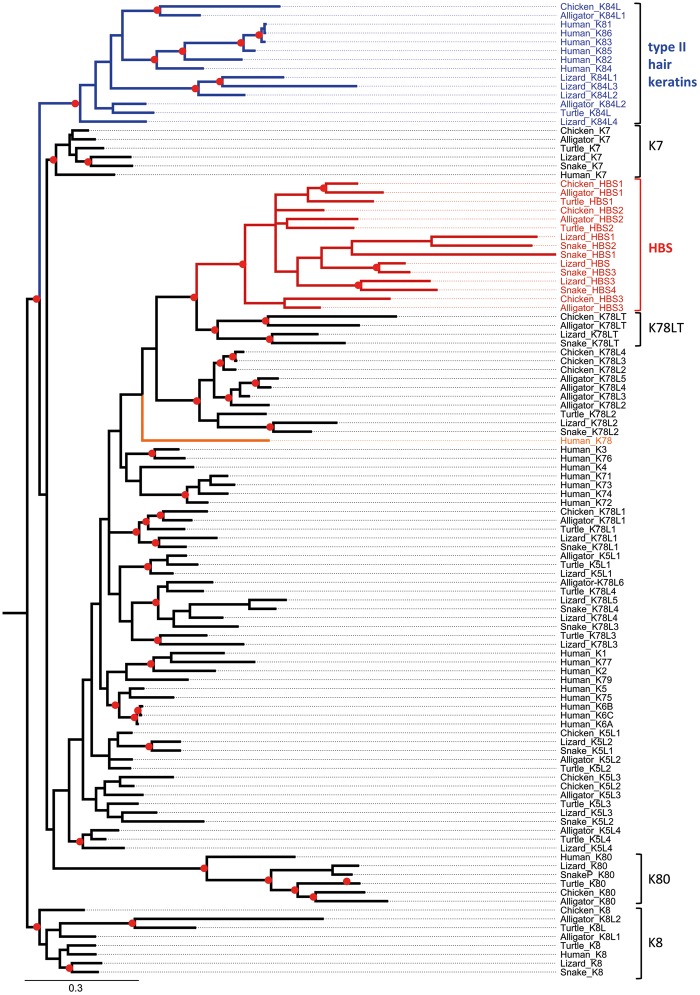
Phylogenetic analysis of type II keratins. A combined evolutionary model of maximum likelihood (ML) and Bayesian analysis of type I keratins is shown. Red circles indicate groups with bootstrap ≥75% obtained by ML analysis and posterior probability ≥0.95 obtained by Bayesian analysis. The scale bar represents substitutions/site. HBS, hard basic sauropsid-specific keratin; K78LT, K78 long tail domain.

Phylogenetic analysis showed that HAS (fig. 3) and HBS (fig. 4) keratins form monophyletic clades and neither HAS nor HBS keratins are closely related to hair keratins (figs. 3 and 4; supplementary fig. S5, Supplementary Material online).The mammalian gene most closely related to HAS keratins is the cysteine-poor keratin K9 and a group of cysteine-poor mammalian keratins, including K78, are most closely related to HBS keratins. Mammalian K9 and K78 are expressed in stratified epithelia but not in hair or nails (Fu et al. 2014; Langbein et al. 2016).

The HAS keratin genes (alternatively termed K9-like cysteine-rich keratins) (fig. 1), are flanked by other *KRT9*-like genes (named *KRT9L1*-*6*) which encode keratins with smaller numbers of cysteine numbers (fig. 1). Likewise, HBS keratin genes (K78-like cysteine-rich keratins) are flanked by genes that encode cysteine-poor keratins in most sauropsids (fig. 2). In the American alligator, the HBS gene cluster is flanked by a gene (*KRT78LT*) which encodes a K78-like keratin with an unusually long tail domain (>1,700 amino acid residues) and more than 50 cysteine residues (fig. 2*B* and supplementary table S2 and figs. S1 and S4, Supplementary Material online). The *KRT78LT* gene is conserved in archosaurs, however, the currently available genome sequences of the chicken and the Chinese alligator contain premature in-frame stop codons in *Krt78LT* orthologs (supplementary fig. S4, Supplementary Material online). Thus, HBS keratins are the only cysteine-rich type II keratins faithfully conserved across sauropsids.

Comparative analysis of fishes and amphibians suggested their keratins have generally low cysteine contents (supplementary figs. S5–S8 and tables S11–S14, Supplementary Material online). We identified, however, one type I keratin with more than 20 cysteine residues in the zebrafish (*Danio* *rerio*) (supplementary table S14, Supplementary Material online) and at least one type I and two type II keratins with more than 18 cysteine residues in the clawed frogs *Xenopus* *tropicalis* (supplementary fig. S1, Supplementary Material online) and *Xenopus* *laevis* (not shown). Molecular phylogenetics showed that these keratins of medium to high cysteine content are neither closely related to each other nor to the cysteine-rich keratins of amniotes (supplementary figs. S5 and S8, Supplementary Material online), indicating that their cysteine contents have increased after phylogenetic divergence of fishes and frogs from the evolutionary lineage leading to fully terrestrial vertebrates.

Importantly, there are no cysteine-rich orthologs of either HAS, HBS, or hair keratins in *Xenopus* *tropicalis* (figs. 1 and 2; supplementary fig. S5, Supplementary Material online), other amphibians (Tibetan frog, axolotl, caecilian) (supplementary figs. S6 and S7 and tables S11–S13, Supplementary Material online), or fishes (*Danio rerio*, *Takifugu rubripes*, *Oncorhynchus mykiss*) (supplementary fig. S8, Supplementary Material online and data not shown), suggesting that HAS, HBS, type I, and type II hair keratins have evolved from cysteine-poor ancestral keratins. Thus, the increase of the cysteine content in four phylogenetically distinct groups of keratins (HAS, type I hair keratins, HBS, type II hair keratins) of amniotes represents a case of convergent evolution.

### Keratin HBS1 is a Protein Component of Chicken Feathers

To determine the expression pattern of cysteine-rich non-hair keratins of a sauropsid species, we reanalyzed a proteomic data set from cornified skin appendages of the chicken (Rice et al. 2013). HAS1 and HAS2 keratins, of which only fragments were annotated when the proteomes were analyzed (Rice et al. 2013), were highly abundant in beak, claws, feathers, and scales (supplementary fig. S9, Supplementary Material online). By contrast, HBS keratins were not found in this data set which is most likely due to the incompleteness of the reference proteome at the time of this proteome analysis.

**FIg. 5.**
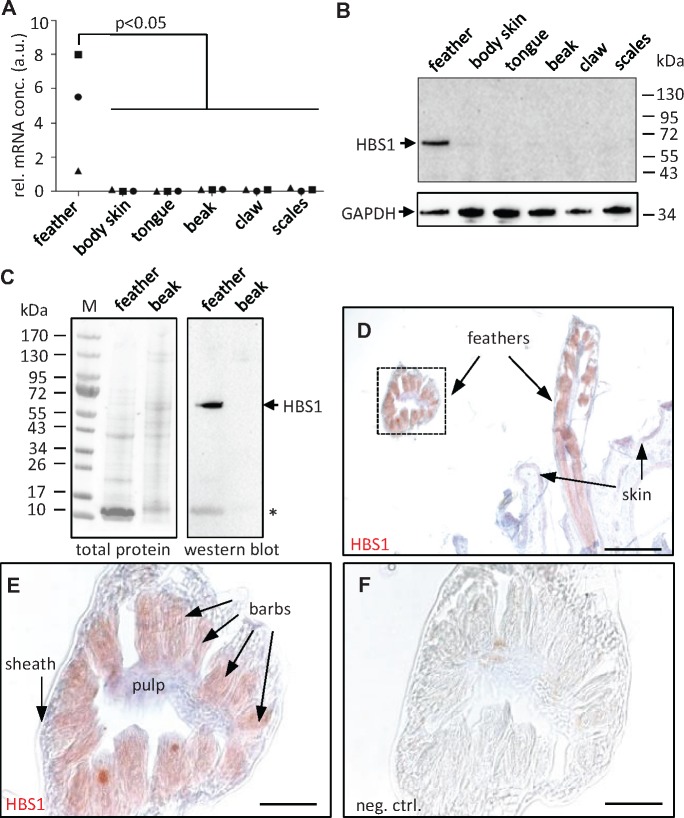
HBS1 keratin is expressed in feathers. (*A*) Quantitative RT-PCR analysis of HBS1 in different chicken tissues on embryonic day 18. The expression was normalized to that of keratin 5. The relative mRNA concentration (rel. mRNA conc.) is shown in arbitrary units (a.u.). The difference between expression in feathers and other tissues from n = 3 chickens was significant. (*B*) Western blot analysis of HBS1 in chicken tissues on embryonic day 18. The membrane was reprobed with anti-GAPDH as a loading control. kDa, kilo-Dalton. (*C*) Western blot analysis of HBS1 in chicken feathers and beak collected on the first day after hatching. Ponceau staining of total protein is shown to confirm equal loading of lanes. An asterisk indicates the position of corneous beta-proteins which represent a major amount of soluble proteins of feathers. M, molecular mass marker. (*D* and *E*) Immunohistochemical staining (red) of HBS1 in wing feathers. The sections were counterstained with hematoxylin (blue). The image in panels (*E*) was recorded at higher magnification than the image in panel (*D*) where the same detail is indicated by a box. (*F*) Negative control (neg. ctrl.) staining in which the primary antibody was replaced by preimmune serum. Size bars: 200 µm (*D*), 50 µm (*E* and *F*).

We chose HBS1 keratin of the chicken for further characterization at the mRNA and protein levels. Quantitative reverse transcription polymerase chain reaction (qRT-PCR) analysis of HBS1 mRNA in the skin of different body sites of chicken at embryonic day 18 showed high expression levels of HBS1 in feathers (fig. 5*A*). HBS2 and HBS3 were also expressed in feathers and in the case of HBS3 at even higher levels in scales (supplementary fig. S10, Supplementary Material online). Next, we raised an antiserum against a unique peptide of HBS1 and used it for the immunochemical detection of the HBS1 protein. Western blot analysis of a feather lysate at day 18 of embryonic development showed a band at 66 kDa, corresponding to the predicted molecular weight of HBS1 (fig. 5*B*). In protein lysates from hatched chickens the expression of HBS1 was confined to feathers (fig. 5*C*). By immunohistochemistry, HBS1 was detected in the barbs and barbules of growing feathers (fig. 5*D* and *E*), whereas a control experiment with preimmune serum did not yield immunostaining (fig. 5*F*). These data demonstrated that chicken HBS1 is predominantly expressed in feathers where it localizes to the epithelial cells that cornify to build the fine, yet mechanically stable feather branches.

### Comparative Analysis Suggests That Cysteine-Rich Keratins Were Co-opted for Functions in the Cytoskeleton of Feathers

**FIg. 6.**
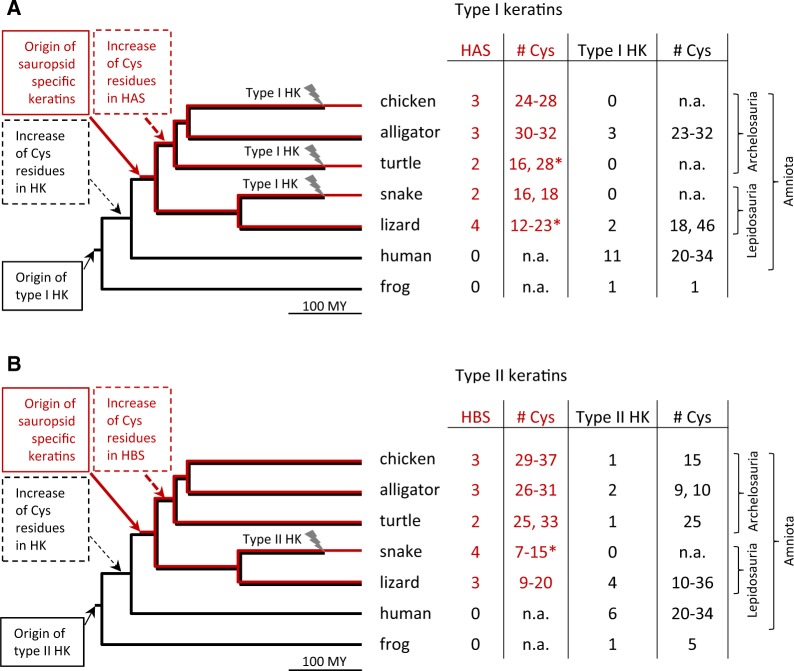
Evolutionary model of the origin of cysteine-rich keratins in amniotes. The numbers and types of cysteine (Cys)-rich type I (*A*) and type II (*B*) keratins per species were mapped onto a simplified phylogenetic tree of tetrapods. The origin and loss (flash symbol) events were inferred from the presence or absence of particular keratin types in modern species and their known phylogenetic relationships. Numbers of cysteine (# Cys) per keratin are indicated. Asterisks indicate numbers of Cys residues that were determined in partial protein sequences; the actual numbers may be higher. HK, hair keratin; HAS, hard acidic sauropsid-specific keratin; HBS, hard basic sauropsid-specific keratin; My, million years.

Finally, we compared the number of hair keratins, HAS and HBS keratins in species from different branches of the phylogenetic tree of tetrapods to infer the evolutionary history of these keratins (fig. 6). The presence of cysteine-rich hair keratin orthologs in mammals and sauropsids suggests that ancestral type I and II hair keratins experienced a rise of the cysteine content already in early amniotes. The absence of hair keratin orthologs in some sauropsids indicates parallel loss of these genes in more than one clade (fig. 6). Cysteine-rich HAS and HBS keratins are only present in sauropsids, suggesting a rise of their cysteine content after the divergence of sauropsids from the mammalian lineage. The alternative hypothesis of cysteine-rich ancestral HAS and HBS keratins being present in the last common ancestor of amniotes followed by the subsequent loss of both HAS and HBS keratins in mammals appears less likely but cannot be entirely ruled out. The numbers of cysteine residues in HAS and HBS keratins of archelosaurs (turtles, crocodilians, and birds) are higher than in the orthologous keratins of lepidosaurs (lizards and snakes), suggesting that the cysteine contents of HAS and HBS keratins increased during the early evolution of archelosaurs. Thus, the expression of the highly cysteine-rich HBS1 keratin in bird-specific skin appendages, that is, feathers (fig. 5), was not driven by lineage-specific gene innovation or sequence changes but rather by co-option of one of the existing HBS keratins during the evolution of feathers.

## Discussion

The results of this study reveal that, in addition to the previously characterized evolution of type I and type II “hair keratins” in early amniotes (Eckhart et al. 2008; Vandebergh and Bossuyt 2012), two separate groups of cysteine-rich keratins, that is, HAS and HBS keratins, evolved in a common ancestor of modern reptiles and birds. The conservation of HAS and HBS keratins in all clades of sauropsids suggests important roles in their integument, likely representing the molecular basis for a significant difference between the integuments of sauropsids and mammals. Our demonstration of expression of one of the newly described keratins, HBS1, in cornifying feathers supports the hypothesis that these keratins are important components of hard skin appendages. The molecular evolution of skin appendages can now be explored in a more comprehensive manner.

Our analysis of genes that encode cysteine-rich keratins was made possible by the determination and publication of genome sequences of many sauropsids (International Chicken Genome Sequencing Consortium 2004; Shaffer et al. 2013; Wan et al. 2013; Aird et al. 2017). Although these genome sequences are very useful resources, it is important to note that there are still gaps and uncertainties in the assembly of regions containing keratin genes which may be partly caused by the presence of stretches of high sequence similarity in keratin genes. Conserved synteny and molecular phylogenetics suggest that hair keratin genes have orthologs in some but not all sauropsids whereas cysteine-rich keratins (HAS and HBS) not orthologous to hair keratins are present in all reptiles and birds investigated. Interestingly, the total number of cysteine-rich keratins (HAS, HBS, and hair keratin orthologs) is generally lower in sauropsid species than in mammalian species, suggesting that the complexity of sauropsidian skin appendages, such as feathers, does not depend on a high diversity of keratins whereas it may be facilitated by diversification of other epidermal differentiation proteins (Strasser et al. 2014; Holthaus et al. 2018). The precise arrangement of HAS and HBS keratin genes was uncertain for several sauropsid species and 3′-terminal exons of some HAS and HBS keratin genes could not be identified due to gaps in the reference genome sequences. In particular, gaps in the current assembly of genome sequences precluded the precise determination of cysteine numbers for lizard HAS1 (supplementary table S4, Supplementary Material online) and HBS2 of the turtle (*Chrysemys* *picta*). Although further characterization of individual HAS and HBS keratin genes is warranted, our data strongly support the general conclusion that the cysteine content has increased in these two clades of sauropsid keratins that are not orthologous to hair keratins.

Our analysis of the sauropsid keratins extends the findings of previous studies (Vandebergh and Bossuyt 2012; Greenwold et al. 2014) by exploring, in detail, the evolution of cysteine-rich keratins. We propose that two groups of cysteine-rich keratins, that is, HAS and HBS keratins, evolved from cysteine-poor keratins and acquired a key molecular trait, that is, the high number of cysteine residues, that is equivalent to the high cysteine content of type I and type II hair keratins which evolved from other cysteine-poor keratins. The high cysteine contents of HAS, HBS, type I, and type II hair keratins have therefore appeared by convergent evolution.

At the level of individual cysteine residues within the intermediate filament domain, which is the amino acid sequence region that can be faithfully aligned, convergent evolution has likely occurred at several amino acid positions of type I keratins (HAS and type I hair keratins) and type II keratins (HBS and type II hair keratins) but the majority of cysteine residues are present at positions specific for only one phylogenetic clade of keratins (supplementary fig. S11, Supplementary Material online). This pattern suggests that keratin cross-linking to other keratins or keratin-binding proteins in hard skin appendages of mammals and sauropsids does not strictly depend on the positions but on the numbers of available cysteine residues.

The immunolocalization of the chicken HBS1 keratin in feathers supports the hypothesis that this keratin and probably also other sauropsid-specific cysteine-rich keratins contribute to the cytoskeleton of hard skin appendages in sauropsids. Keratin filaments are known to be present in feathers (Rice et al. 2013; Wu et al. 2015; Alibardi 2016; McNamara et al. 2018) but specific individual keratins had not been localized by immunohistochemistry in feathers. Since keratin intermediate filaments are formed by pairs of a type I and a type II keratin (Schweizer et al. 2006), it will be important to determine if there is a specific heterodimerization partner of HBS1. In mammals there is often coexpression of specific pairs of one type I and one type II keratin that form heterodimers, for example, K5 and K14 in the basal layer of the epidermis. However, more than one type I and more than one type II keratin are expressed in the cortex and in medulla of hair fibers, suggesting promiscuity in the binding of keratins (Langbein, Eckhart, et al. 2010; Langbein, Yoshida, et al. 2010). Whether this promiscuity extends to HAS and HBS keratins, possibly interacting with hair keratin orthologs of sauropsids, remains to be investigated.

The increase of the cysteine content in different clades of keratins is an example of convergent evolution driven by the selection for stable protein–protein interactions, that is, cross-linking via disulfide bonds. This ability to become cross-linked is a crucial feature of structural proteins of hard skin appendages of terrestrial vertebrates which require mechanical resilience in a dry environment. The evolution of high cysteine contents in keratins was paralleled by the evolutionary rise of cysteine contents in other cornification proteins, such as high-sulfur keratin-associated proteins (KRTAPs) in mammals and epidermal differentiation cysteine-rich protein (EDCRP), epidermal differentiation protein containing DPCC motifs (EDDM), and CBPs in sauropsids (Strasser et al. 2014; Strasser et al. 2015; Lachner et al. 2019). The emergence of the sequence similarity of high-sulfur KRTAPs and EDCRP represents another case of convergent evolution at the molecular level (Strasser et al. 2015). Importantly, a class of small proteins, originally called “beta-keratins” and later renamed “corneous beta-proteins” (CBPs), have evolved specifically as components of sauropsidian skin appendages and some of them have also acquired high cysteine contents (Holthaus et al. 2018). Hair keratins are cross-linked with KRTAPs in hair, and it appears likely that the sauropsid-specific keratins undergo covalent cross-linking with the other sauropsid-specific cornification proteins such as EDCRP and CBPs to form protein networks that confer mechanical resistance to feathers and other hard skin appendages of sauropsids.

The convergent evolution of cysteine-rich keratins in sauropsids differs from most cases of molecular convergence in the literature because the convergently evolving genes (HAS, HBS, and hair keratin orthologs) resided in the same genome. Both hair keratin orthologs, which functioned as claw keratins (Eckhart et al. 2008), and the molecular ancestors of HAS and HBS keratins were present in early sauropsids. We hypothesize that HAS and HBS keratins were originally expressed in epidermal structures, such as scales, that lacked expression of hair/claw keratins, and only later they were co-opted into other skin appendages. It is currently unknown whether HAS and HBS keratins are coexpressed and possibly interact via disulfide bonds with hair keratin orthologs in sauropsidian skin appendages. If so, the sauropsidian cysteine-rich keratins could be considered products of “cooperative evolution.” However, in some clades of sauropsids the evolutionarily older hair keratins were apparently replaced by HAS and HBS keratins, indicating “competitive evolution” among cysteine-rich keratins. In turtles and birds, except the kiwi (fig. 6 and supplementary table S8, Supplementary Material online), type I hair keratin orthologs were lost and the ostrich and songbirds additionally lost type II hair keratin orthologs (supplementary tables S9 and S10, Supplementary Material online), suggesting functional substitution by other keratins. This notion is supported by the detection of HAS1 and HAS2 in cornified claws (supplementary fig. S9, Supplementary Material online) of the chicken which lacks the primordial claw keratins of the type I hair keratin family. Further studies of the expression patterns of HAS and HBS keratins are necessary to test and refine the models of convergent evolution, followed by cooperative or competitive evolution of cysteine-rich keratin genes in sauropsids.

In conclusion, the results of the present study suggest that convergent evolution of cysteine-rich keratins accompanied and possibly facilitated the parallel evolution of hard skin appendages in mammals and sauropsids. The expression of a HBS keratin in cornifying feathers and the presence of similarly cysteine-rich orthologs in featherless reptiles indicate that co-option of these cross-linkable keratins contributed to the evolution of skin appendages in sauropsids.

## Materials and Methods

### Comparative Genomics, Sequence Alignments, and Phylogenetic Analysis

The genome sequences of the chicken (red junglefowl, *Gallus gallus*) (International Chicken Genome Sequencing Consortium 2004), Chinese alligator (*Alligator sinensis*) (Wan et al. 2013), painted turtle (*Chrysemys* *picta bellii*) (Shaffer et al. 2013), anole lizard (*Anolis carolinensis*) (Alföldi et al. 2011), venomous pit viper (*Protobothrops mucrosquamatus*) (Aird et al. 2017), and human (*Homo sapiens*) were investigated for the presence or absence and sequence integrity of type I and type II keratin genes. In addition, genes encoding cysteine-rich keratins were identified in the genomes of the Tibetan frog (*Nanorana parkeri*) (Wang et al. 2018), clawed frogs (*Xenopus* *laevis* and *Xenopus* *tropicalis*), zebrafish (*Danio rerio*), kiwi (*Apteryx australis mantelli*), ostrich (*Struthio camelus australis*), zebra finch (*Taeniopygia guttata*), American alligator (*Alligator mississipiensis*), green sea turtle (*Chelonia mydas*), Burmese python (*Python bivittatus*), and platypus (*Ornithorhynchus anatinus*). Sequences were obtained from the GenBank database of the National Center for Biotechnology Information (NCBI), United States (http://www.ncbi.nlm.nih.gov/; last accessed October 31, 2019). Keratin cDNAs were identified in the axolotl (*Ambystoma mexicanum*) and a caecilian (*Microcaecilia unicolor*). Basic Local Alignment Search Tool (BLAST) (Altschul et al. 1990) was used to search for sequence similarities. The Multalin algorithm was used for multiple sequence alignments (Corpet 1988). The conservation of blocks of order of genetic elements (synteny) was tested by manual alignment of gene maps including conserved genes on each side of the genes of interest. Gene orthologies between species were inferred from shared synteny and phylogenetic analysis.

For molecular phylogenetics, only the amino acid sequences of the intermediate filament domains were aligned (supplementary figs. S12–S14, Supplementary Material online). The molecular phylogenies of keratins were determined by maximum likelihood analysis (model: JTT) with 100, 200, and 500 bootstrap replicates to assess clade support for type I keratins, type II keratins and all keratins together, respectively, on the Seaview platform (Gouy et al. 2010). A mixed amino acid model with gamma correction for rate heterogeneity and invariable sites was used for estimating Bayesian posterior probabilities in MrBayes 3.2.6 (Ronquist et al. 2012). Two parallel Markov chain Monte Carlo (MCMC) runs of four chains each were performed, with a length of 5,000,000 (fig. 3), 10,000,000 (fig. 4 and supplementary figs. S6–S8, Supplementary Material online), or 50,000,000 (supplementary fig. S5, Supplementary Material online) generations and a sampling frequency of 1 per 1,000 generations. To define the burnin and check the convergence of the MCMC runs, Tracer v1.7.1 was used (Rambaut et al. 2018). Trees were visualized with FigTree v1.4.2. Phylogenetic relationships and divergence times of species were obtained from the Timetree website (www.timetree.org; last accessed October 31, 2019) (Hedges et al. 2015).

### Preparation of Chicken Tissue Samples

All animal procedures were approved by the Animal Care and Use Committee of the Medical University of Vienna (66.016/0014-II/3b/2011), and all these procedures were conducted according to the guidelines established by this committee. Fertilized chicken eggs were incubated according to published protocols (Eresheim et al. 2014). Hatched chickens were obtained by Schropper GmbH, Gloggnitz, Austria. Chickens were decapitated at embryonal development day 18 or on the first day after hatching. Tissue samples for protein analysis were frozen immediately at −20 °C until further processing. Proteins were extracted with Laemmli extraction buffer containing 2% SDS by homogenization with the Precellys system (VWR International, Radnor, PA). Protein concentrations were determined with the Micro BCA protein assay kit (Thermo Fisher Scientific). For analyses of RNA, samples from different chicken tissues were cut into small pieces and kept in TriFast (VWR International) at 4 °C overnight. RNA was purified using the Precellys system (VWR International, Radnor, PA) and TriFast according to the manufacturers’ instructions.

### Reverse Transcription-Polymerase Chain Reactions

RNA from tissues was quantified with the Nanodrop system and 400–500 ng aliquots were reverse-transcribed to cDNA using the Iscript Kit (Biorad, Hercules, CA). Quantitative PCRs were performed using the LightCycler 480 DNA SYBR Green I Master Kit (Roche Applied Science) and the LightCycler^®^ technology (LC480). The primer pairs HBS1-s (5′-TGGCCCTGGACATTGAGATT-3′) and HBS1-a (5′-TGGCTCCAGTCTTCACAGAG-3′), and K5-s (5′-TGGAGAGATGGCCCTGAAAG-3′) and K5-a (5′-CCACCTGTACTGAGACCTCC-3′) were used for the amplification of chicken HBS1 and K5, respectively. Quantities of target relative to reference transcripts were calculated according to a published mathematical model (Pfaffl 2001). The statistical significance of differences in expression levels was tested with ANOVA.

Other HBS keratin mRNAs were amplified by semiquantitative PCRs using the primers HBS2-s (5′-ACGCTGTCAGGAGAAGGAAC-3′), HBS2-a (5′-TTCTCACACAGCAAGCAGTC-3′), HBS3-s (5′-GAAGTCCCACCAGCAAGAGA-3′), and HBS3-a (5′-GTGGCGATCTCAATGTCCAG-3′), an annealing temperature of 60 °C and 32 amplification cycles. The ubiquitously expressed apoptotic protease, caspase-3, was amplified as a control gene using the primers CASP3-s (5′-TGGCGATGAAGGACTCTTCT-3′) and CASP3-a (5′-CTGGTCCACTGTCTGCTTCA-3′).

### Generation of Antisera

Antisera against HBS1 keratin were raised by immunizing mice six times with 100 µg of the synthetic peptide SAASCVGREVLSGADGLQC, corresponding to amino acid residues 553–571 of HBS1 keratin, coupled to keyhole limpet hemocyanin (KLH) according to a published protocol (Eckhart et al. 2008).

### Western Blot Analysis

About 50 µg protein per lane were electrophoresed through an ExcelGel SDS gradient 8–18% polyacrylamide gel (GE Healthcare Life Sciences, Chicago) and blotted onto a nitrocellulose membrane. The membrane was incubated with anti-HBS1 antibody (1:500) or, for comparison, with mouse anti-GAPDH (HyTest, 1:5,000) at 4 °C overnight. Then the membrane was incubated with sheep anti-mouse immunoglobulin (GE Healthcare Life Sciences, 1:10,000) coupled to horseradish peroxidase for 1 h at room temperature, and bands were visualized using the enhanced chemiluminescence system (SuperSignal West Dura Extended Duration Substrate, Thermo Fisher Scientific).

### Histology and Immunohistochemistry

Immunohistochemistry was performed according to published protocols (Mlitz et al. 2014; Ehrlich et al. 2019) with modifications. In brief, antigens were demasked with citrate buffer (pH 6), and the samples were incubated with an antiserum against HBS1 keratin (dilution 1:500). Biotinylated sheep anti-mouse immunoglobulin (RPN1001V, lot 9793564, GE Healthcare, Chalfont, UK) at a dilution of 1:100 was used as secondary antibody, and sheep serum (10%) was added to prevent unspecific binding. In control experiments, the primary antibody was replaced by serum of the same mouse prior to immunization (preimmune serum) at the same dilution. The samples were incubated with streptavidin–biotin–horseradish peroxidase (HRP) complex and 3-amino-9-ethylcarbazole (DakoCytomation, Glostrup, Denmark) to develop red color. Nuclei were counterstained with hematoxylin. 

## Supplementary Material

msz279_Supplementary_Data
