# Supplementary material for: Convergent Evolution of Cysteine-Rich Keratins in Hard Skin Appendages of Terrestrial Vertebrates
Source: Mol Biol Evol. 2019 Dec 10;37(4):982–93. doi: 10.1093/molbev/msz279 (PMC7086170; doi:10.1093/molbev/msz279)
Supplement: msz279_Supplementary_Data [file msz279_supplementary_data.pdf]

Supplementary Data: Supplementary Tables and Figures

Convergent evolution of cysteine-rich keratins in hard skin appendages of terrestrial vertebrates

Florian Ehrlich, Julia Lachner, Marcela Hermann, Erwin Tschachler, Leopold Eckhart

Content

Supplementary Tables S1-S14

Supplementary Figures S1-S14

Supplementary Table S1. Keratin genes of the chicken (*Gallus gallus*)

Keratin type	Name[1]	Genomic DNA sequence (acc. nr.)	Gene start[2] (nucl. nr.)	Gene end[2] (nucl. nr.)	Gene name (GenBank)	Gene (GenBank ID)	Protein[3] (acc. nr.)	Differences between new gene prediction and GenBank (GCF_000002315.5)[4]
**type I**	K222	NC_006114.5	7326273	7321193	KRT222	420046	NP_001182406.1	
**type I**	K12	NC_006114.5	7335707	7329801	n.a.	n.a.	n.a.	
**type I**	K20	NC_006114.5	7340108	7336399	KRT40	420045	NP_001264910.1	
**type I**	K23	NC_006114.5	7359178	7349963	KRT23	420044	XP_418167.4	
**type I**	K15	NC_006114.5	7366321	7362226	KRT15	408040	NP_001001312.2	
**type I**	K19	NC_006114.5	7373828	7369522	KRT19	395861	NP_990340.2	
**type I**	K9L1	NC_006114.5	7384221	7380438	LOC100857659	100857659	XP_003642867.2	GenBank gene prediction comprises 2 coding sequences
**type I**	K9L2	NC_006114.5	7391904	7388878	LOC100857659	100857659	XP_004948661.2	GenBank gene prediction comprises 2 coding sequences
**type I**	K9L3	NC_006114.5	7398958	7394346	KRT10	771977	XP_001235182.3	
**type I**	K9L4	NC_006114.5	7409440	7405257	KRT9L	420041	XP_418163.4	
**type I**	HAS1 (K9LC1)	NC_006114.5	7417651	7413171	LOC772080	772080	XP_001233972.1	
**type I**	HAS2 (K9LC2)	NC_006114.5	7426921	7422075	KRTC42L	420039	XP_418162.6	
**type I**	HAS3 (K9LC3)	NC_006114.5	7434663	7432253	LOC771995	771995	XP_003642868.1	
**type I**	K14L1	NC_006114.5	7442528	7438886	KRT14	408039	NP_001001311.2	
**type I**	K14L2	NC_006114.5	7449377	7445505	KRT17	100858439	NP_001264913.1	
**type I**	K18	NC_008465.4	5401187	5408382	KRT18	101749333	XP_025001434.1	
**type II**	K80	NC_008465.4	5202555	5188564	KRT80	431302	XP_428853.4	
**type II**	K7	NC_008465.4	5216450	5224940	KRT7	395772	NP_990263.1	
**type II**	K84L	NC_008465.4	5233503	5230255	LOC426897	426897	XP_015155864.2	
**type II**	K5L3	NC_008465.4	5245539	5241947	KRT75	408042	NP_001001314.2	
**type II**	K5L2	NC_008465.4	5254375	5249671	KRT6A	408041	NP_001001313.2	
**type II**	K5L1	NC_008465.4	5267020	5260881	KRT5	407779	NP_001001195.1	
**type II**	HBS1 (K78LC1)	NC_008465.4	5287405	5280968	KRT75L4	100858302	XP_015155859.1	
**type II**	HBS2 (K78LC2)	NC_008465.4	5305881	5300187	LOC768978	768978	XP_015155862.1	
**type II**	HBS3 (K78LC3)	NC_008465.4	5316137	5311299	LOC107055419	107055419	XP_025001374.1	
**type II**	K78LT	NC_008465.4	5335060	5322171	LOC431300	431300	partially identical: XP_025001353.1	Prediction is uncertain, multiple frame-shift mutations, hypothetical amino acid sequence is shown in Suppl. Fig. S1
**type II**	K78L4	NC_008465.4	5351881	5348560	KRT75L2	431299	XP_428850.2	
**type II**	K78L3	NC_008465.4	5360226	5356373	KRT71	768881	XP_001232027.4	
**type II**	K78L2	NC_008465.4	5369524	5365170	KRT75L1	768880	XP_015155860.1	
**type II**	K78L1	NC_008465.4	5379290	5374907	LOC112529929	112529929	XP_001231586.2	
**type II**	K8	NC_008465.4	5396641	5388108	KRT8	426896	XP_015128103.2	

Notes: [1] Keratins were named according to orthology to human proteins. K9-like cysteine-rich keratins (K9LC) are termed HAS and K78-like cysteine-rich keratins (K78LC) are termed HBS keratins. HAS, hard acidic sauropsid-specific; HBS, hard basic sauropsid-specific; L, like; LT, long tail.

[2] Gene start and end indicate the start and stop codon of the coding sequence. Introns are not indicated.

[3] Accession numbers (acc. nr.) of protein amino acid sequences predicted in GenBank.

[4] Genome sequence assembly: GRCg6a (GCF_000002315.5).

acc., accession; n.a., not available in the genome sequence assembly; nucl., nucleotide; nr., number.

Supplementary Table S2. Keratin genes of the Chinese alligator (*Alligator sinensnis)*

Keratin type	Name[1]	Genomic DNA sequence (acc. nr.)	Gene start[2] (nucl. nr.)	Gene end[2] (nucl. nr.)	Gene name (GenBank)	Gene (GenBank ID)	Protein[3] (acc. nr.)	Differences between new gene prediction and GenBank (GCF_000455745.1)[4]
**type I**	K222	NW_005842040.1	395100	404167	KRT222	102385505	XP_006022588.2	
**type I**	K24	NW_005842040.1	366765	376063	LOC102378366	102378366	XP_025058718.1	
**type I**	K10L	NW_005842040.1	335322	345003	LOC102378129	102378129	XP_025058719.1	Corrected exon 1: start at nucl. 335322
**type I**	K12	NW_005842040.1	303666	309983	LOC102377886	102377886	XP_006022639.1	
**type I**	K20	NW_005842040.1	275299	289066	KRT20	102385271	XP_006022587.1	
**type I**	K23	NW_005842040.1	178687	214213	LOC102377411	102377411	XP_006022637.2	
**type I**	K36L1	NW_005842040.1	127867	137534	LOC102377177	102377177	XP_006022636.1	Exon 1: start at nucl. 6043 relative to GenBank prediction; end of exon 6
**type I**	K36L2	NW_005842040.1	116254	122570	LOC102376945	102376945	XP_006022635.2	Corrected exon 1: starts at nucl 116254, exon 7 with premature stop codon
**type I**	K36L3	NW_005842040.1	100211	104304	LOC102376692	102376692	XP_025058710.1	
**type I**	K15L	NW_005842040.1	76246	84026	LOC102385021	102385021	XP_006022586.1	
**type I**	K19	NW_005842040.1	62672	69119	LOC102384765	102384765	XP_006022585.1	
**type I**		NW_005842040.1						
**type I**	K9L1	NW_005842040.1	30264	35981	LOC102384514	102384514	XP_006022584.1	
**type I**	K9L	NW_005842040.1	3318	14074	LOC102376455	102376455	XP_025058716.1	Incomplete start, inactivating mutation: frame shift at nucl. 11877
**type I**	K9L2	NW_005843869.1	11069	17220	LOC102379508	102379508	XP_006039283.2	
**type I**	K9L3	NW_005842585.1	1032077	1036258	LOC102369678	102369678	XP_006032846.1	
**type I**	K9L4	NW_005842585.1	1012386	1018971	LOC102373165	102373165	XP_025070308.1	
**type I**	K9L5	NW_005842585.1	985187	992565	LOC102369323	102369323	XP_025070304.1	
**type I**	HAS1 (K9LC1)	NW_005842585.1	947406	954479	LOC102368806	102368806	XP_006032843.1	
**type I**	HAS2 (K9LC2)	NW_005842585.1	925915	931931	LOC102369067	102369067	XP_006032844.1	
**type I**	K14L1	NW_005842585.1	878298	886416	LOC102368390	102368390	XP_006032841.1	
**type I**	K14L2	NW_005842585.1	864255	870926	LOC102367973	102367973	XP_006032839.1	
**type I**	K117	NW_005842585.1	819205	826538	LOC102372666	102372666	XP_025070287.1	
**type I**	K18	NW_005842452.1	1162577	1158980	KRT18	102375632	XP_006030669.1	
**type II**	K80	NW_005842526.1	782023	782023	KRT80	102371067	XP_006031790.2	
**type II**	K7	NW_005842526.1	829141	829141	LOC102371330	102371330	XP_006031791.1	
**type II**	K84L1	NW_005842526.1	845249	845249	LOC102377597	102377597	XP_006031815.1	
**type II**	K84L2	NW_005842526.1	860922	860922	LOC102377836	102377836	XP_025069161.1	Last exon with sequence gap, corrected to 860920..861200
**type II**	K5L4	NW_005842526.1	870439	870439	LOC102371761	102371761	XP_006031793.1	
**type II**	K5L3	NW_005842526.1	893670	893670	LOC102372247	102372247	XP_006031794.2	This GenBank gene prediction combines 2 coding sequences. Besides the gene indicated here, another gene starts at nucl. 888065, frame shift: nucl. 887772, premature stop: nucl. 887601
**type II**	K5L2	NW_005842526.1	919981	919981	LOC102372665	102372665	XP_006031796.1	
**type II**	K5L1	NW_005842526.1	935435	935435	LOC102378082	102378082	XP_006031817.1	
**type II**	HBS1 (K78LC1)	NW_005842526.1	955663	955663	LOC102373417	102373417	XP_006031800.1	
**type II**	HBS2 (K78LC2)	NW_005842526.1	977316	977316	LOC102378317	102378317	XP_025069184.1	
**type II**	HBS3	NW_005842526.1	992203	992203	LOC102378551	102378551	XP_025069194.1	
**type II**	K78LT	NW_005842526.1	1027861	1027861	LOC102373665	102373665	XP_006031801.1	Predicted exon 7: 1031791..1032015, predicted exon 8: 1031021..1031049, exon 9: 1027860..1030907 then frameshift
**type II**	K78L6	NW_005842526.1	1060335	1060335	LOC102374427	102374427	XP_014379207.1	Predicted exon 7: 1120942..1121156, exon 8: 1120411..1120445
**type II**	K78L5	NW_005842526.1	1089374	1089374	LOC102379545	102379545	XP_025069173.1	
**type II**	K78L4	NW_005842526.1	1105253	1105253	LOC102380041	102380041	XP_014379196.1	
**type II**	K78L3	NW_005842452.1	1279629	>1284625	LOC102380269	102380269	XP_006030688.1	Incomplete, exon 6 ends at nucl. 1284625, exons 7 and 8 are missing because of a gap in the genome sequence
**type II**	K78L2	NW_005842452.1	1260757	1265945	LOC102380036	102380036	XP_014378646.1	
**type II**	K78L1	NW_005842452.1	1250240	1256757	LOC106722579	106722579	XP_014378640.1	
**type II**	K8L	NW_005842452.1	1201934	1209453	LOC102379785	102379785	XP_006030686.1	
**type II**	K8	NW_005842452.1	1183482	1192777	KRT8	102375882	XP_006030670.1	

Notes: [1] Keratins were named according to orthology to human proteins. K9-like cysteine-rich keratins (K9LC) are termed HAS and K78-like cysteine-rich keratins (K78LC) are termed HBS keratins.
HAS, hard acidic sauropsid-specific; HBS, hard basic sauropsid-specific; L, like; LT, long tail.

[2] Gene start and end indicate the start and stop codon of the coding sequence. Introns are not indicated.

[3] Accession numbers (acc. nr.) of protein amino acid sequences predicted in GenBank.

[4] Genome assembly: ASM45574v1 (GCF_000455745.1).

acc., accession; n.a., not available in the genome sequence assembly; nucl., nucleotide; nr., number.

Supplementary Table S3. Keratin genes of the turtle (*Chrysemys picta bellii*)

Keratin type	Name[1]	Genomic DNA sequence (acc. nr.)	Gene start[2] (nucl. nr.)	Gene end[2] (nucl. nr.)	Gene name (GenBank)	Gene (GenBank ID)	Protein[3] (acc. nr.)	Differences between new gene prediction and GenBank (GCF_000241765.3)[4]
**type I**	K222	NW_007359872.1	6017079	6007457	KRT222	101946940	XP_023956555.1	
**type I**	K24	NW_007359872.1	6026071	6020015	KRT24	101946677	XP_023956552.1	
**type I**	K12	NW_007359872.1	6059837	6053610	LOC101946402	101946402	XP_023956546.1	
**type I**	K20	NW_007359872.1	6079302	6066953	KRT20	101946118	XP_023956553.1	
**type I**	K23	NW_007359872.1	6118243	6103446	KRT23	101944292	XP_008160960.2	
**type I**	K15	NW_007359872.1	6154671	6144684	LOC101944027	101944027	XP_023956548.1	
**type I**	K19	NW_007359872.1	6169782	6164037	LOC101945842	101945842	XP_005294164.1	Prediction ends after intermediate filament domain
**type I**	K9L	NW_007359872.1	6203574	6194703	LOC101943762	101943762	XP_023956544.1	
**type I**	HAS1 (K9LC1)	NW_007359872.1	6239064	6229334	LOC101945565	101945565	XP_023956574.1	
**type I**	HAS2 (K9LC2)	NW_007359872.1	>6251969	6247249	LOC101943485	101943485	XP_023956575.1	Incomplete, corrected exon 1 (starts in unsequenced region: nucl: >6251969), exon 6 (end at nucl: 6247238) is last exon of prediction, corrected exon 6: , exons 7 and 8 in unsequenced region
**type I**	K14L1	NW_007359872.1	6284793	6277519	LOC101943211	101943211	XP_023956549.1	
**type I**	K14L2	NW_007359872.1	6296382	6291231	LOC112058843	112058843	XP_023956551.1	
**type I**	K117	NW_007359872.1	6333434	6327757	LOC101942920	101942920	XP_023956554.1	
**type I**	K18	NW_007281365.1	3188404	3195817	KRT18	101944455	XP_005291938.1	
**type II**	K80	NW_007281365.1	2707507	2683757	KRT80	101937727	XP_005292004.1	
**type II**	K7	NW_007281365.1	2731398	2750824	LOC101942365	101942365	XP_005291931.1	
**type II**	K84L	NW_007281365.1	2774533	2760328	LOC101942824	101942824	XP_005291933.1	
**type II**	K5L4	NW_007281365.1	2797015	2782059	LOC101943098	101943098	XP_005291934.1	
**type II**	K5L3	NW_007281365.1	2812964	2806768	LOC101943376	101943376	XP_008167740.1	
**type II**	K5L2	NW_007281365.1	2839118	2828979	LOC101943657	101943657	XP_005291936.1	
**type II**	K5L1	NW_007281365.1	2863680	2855514	LOC101938008	101938008	XP_008167741.1	
**type II**	HBS1 (K78LC1)	NW_007281365.1	2897943	2888267	LOC101938278	101938278	XP_023962088.1	
**type II**	HBS2 (K78LC2)	NW_007281365.1	2934586	2926151	LOC101938554	101938554	XP_023962089.1	
**type II**	K78L4	NW_007281365.1	2974357	2958939	LOC101938825	101938825	XP_023962090.1	
**type II**	K78L3	NW_007281365.1	2996774	2982340	LOC101939086	101939086	XP_005292009.1	
**type II**	K78L2	NW_007281365.1	3019456	3013010	LOC101943933	101943933	XP_023962091.1	
**type II**	K78L1	NW_007281365.1	3040012	3030390	LOC101939351	101939351	XP_008167744.1	
**type II**	K8L	NW_007281365.1	3108702	3091209	LOC101939638	101939638	XP_005292011.1	
**type II**	K8	NW_007281365.1	3135778	3124174	KRT8	101944191	XP_005291937.1	

Notes: [1] Keratins were named according to orthology to human proteins. K9-like cysteine-rich keratins (K9LC) are termed HAS and K78-like cysteine-rich keratins (K78LC) are termed HBS keratins.
HAS, hard acidic sauropsid-specific; HBS, hard basic sauropsid-specific; L, like; LT, long tail.

[2] Gene start and end indicate the start and stop codon of the coding sequence. Introns are not indicated.

[3] Accession numbers (acc. nr.) of protein amino acid sequences predicted in GenBank.

[4] Genome assembly used for predictions: Chrysemys_picta_bellii-3.0.3 (GCF_000241765.3).

acc., accession; n.a., not available in the genome sequence assembly; nucl., nucleotide; nr., number.

Supplementary Table S4. Keratin genes of the lizard (*Anolis carolinensis*)

Keratin type	Name[1]	Genomic DNA sequence (acc. nr.)	Gene start[2] (nucl. nr.)	Gene end[2] (nucl. nr.)	Gene name (GenBank)	Gene (GenBank ID)	Protein[3] (acc. nr.)	Differences between new gene prediction and GenBank (GCF_000090745.1)[4]
**type I**	K222	NC_014781.1	70386110	70363387	KRT222	100563084	XP_003222508.1	Coding sequence is not disrupted in GenBank. Mutation was not confirmed by our amplification and sequencing.
**type I**	K24	NC_014781.1	70404011	70392422	LOC100552076	100552076	XP_008111591.1	
**type I**	K24L	NC_014781.1	70423458	70411566	LOC103279244	103279244	XP_008111590.1	
**type I**	K12L	NC_014781.1	70454294	70429996	LOC100552270	100552270	XP_016850071.1	
**type I**	K12	NC_014781.1	70467003	70460684	LOC100552468	100552468	XP_003222621.1	
**type I**	K20	NC_014781.1	70488252	70472744	LOC107982248	107982248	XP_016850072.1	Corrected exon 1: nucl: 70488252..70488500, frameshift at nucl. 70488278; not predicted to encode a keratin.
**type I**	K23	NC_014781.1	70552329	70519742	krt23	100552665	XP_008111584.1	
**type I**	K36L1	NC_014781.1	70598936	70591466	LOC100337549	100337549	NP_001280052.1	
**type I**	K36L2	NC_014781.1	70614366	70606275	krt36	100337543	NP_001280048.1	
**type I**	K15	NC_014781.1	70630952	70621017	LOC100552862	100552862	XP_008111595.1	
**type I**	K19	NC_014781.1	70661136	70648031	LOC100563808	100563808	XP_008111599.1	
**type I**	K9L1	NC_014781.1	70711123	70702384	LOC100553064	100553064	XP_008111598.2	
**type I**	K9L2	NC_014781.1	70743419	70729212	LOC100553253	100553253	XP_016850073.1	
**type I**	HAS1 (K9LC1)	NC_014781.1	70773480	70763471	LOC100553450	100553450	XP_016850077.1	Exon in LOC107983006, predicted exon 2: nucl. 7077943..70770861 and exon 3: nucl. 70768746..70768590, corrected exon 4: starts at nucl. 70768463
**type I**	n.a.	NC_014781.1	70773480	70772921	LOC107983006	107983006	XP_016850076.1	exon 1 of HAS1; corrected range: 70773480..70772921
**type I**	HAS2 (K9LC2)	NC_014781.1	70784815	70777029	LOC100337544	100337544	NP_001280049.1	
**type I**	HAS3 (K9LC3)	NC_014781.1	70807056	70799064	LOC100553650	100553650	XP_003222627.3	
**type I**	HAS4 (K9LC4)	NC_014781.1	70828115	70819171	LOC100553851	100553851	XP_016850078.1	
**type I**	K14L1	NC_014781.1	70856045	70844971	LOC100564200	100564200	XP_003222513.1	
**type I**	K14L2	NC_014781.1	70875350	70865556	LOC100564396	100564396	XP_003222514.1	
**type I**	K117	NC_014781.1	70935269	70919125	LOC100554046	100554046	XP_008111601.1	
**type I**	K18	NW_003338797.1	1886076	1901530	KRT18	100567731	XP_003224490.1	
**type II**	K80	NC_014777.1	95942323	95982372	LOC100562605	100562605	XP_008102289.1	
**type II**	K7	NC_014777.1	95891088	95868214	LOC100567055	100567055	XP_003217023.1	
**type II**	K84L1	NC_014777.1	95865739	95849903	LOC100562411	100562411	XP_016846291.1	
**type II**	K84L2	NC_014777.1	95839095	95825623	LOC100337546	100337546	NP_001280051.1	
**type II**	K84L3	NC_014777.1	95817465	95802462	LOC100562208	100562208	XP_003217080.2	
**type II**	K84L4	NC_014777.1	95799482	95789077	LOC100337545	100337545	NP_001280050.1	
**type II**	K5L4	NC_014777.1	95777143	95758353	LOC100562008	100562008	XP_008102284.2	
**type II**	K5L3	NC_014777.1	95731292	95720930	LOC100566287	100566287	XP_003217019.1	
**type II**	K5L2	NC_014777.1	95702695	95689880	LOC100566088	100566088	XP_003217018.1	
**type II**	K5L1	NC_014777.1	95685012	95666735	LOC100561812	100561812	XP_003217078.1	
**type II**	HBS1 (K78LC1)	NC_014777.1	95656070	95645086	LOC100561620	100561620	XP_003217077.2	
**type II**	HBS2 (K78LC2)	NC_014777.1	95623784	95610121	LOC100561422	100561422	XP_003217076.3	
**type II**	HBS3 (K78LC3)	NC_014777.1	95587325	95566790	LOC100561224	100561224	XP_003217075.2	
**type II**	K78LT	NC_014777.1	95532173	95523420	LOC100560827	100560827	XP_016846726.1	
**type II**	K78L5	NC_014777.1	95512139	95490960	LOC100560629	100560629	XP_016846727.1	
**type II**	K78L4	NC_014777.1	95457122	95440262	LOC100560433	100560433	XP_003217072.1	
**type II**	K78L3	NC_014777.1	95436358	95421422	LOC100560234	100560234	XP_008102271.2	
**type II**	K78L2	NC_014777.1	95413159	95401878	LOC100560037	100560037	XP_003217070.1	
**type II**	K78L1	NC_014777.1	95385652	95370266	LOC100565886	100565886	XP_003217017.1	
**type II**	K8	NW_003338797.1	1781585	1779090	KRT8	100566434	XP_003224483.1	

Notes: [1] Keratins were named according to orthology to human proteins. K9-like cysteine-rich keratins (K9LC) are termed HAS and K78-like cysteine-rich keratins (K78LC) are termed HBS keratins.
HAS, hard acidic sauropsid-specific; HBS, hard basic sauropsid-specific; L, like; LT, long tail.

[2] Gene start and end indicate the start and stop codon of the coding sequence. Introns are not indicated.

[3] Accession numbers (acc. nr.) of protein amino acid sequences predicted in GenBank.

[4] Genome assembly used for predictions: AnoCar2.0 (GCF_000090745.1).

acc., accession; n.a., not available in the genome sequence assembly; nucl., nucleotide; nr., number.

Supplementary Table S5. Keratin genes of the snake (*Protobothrops mucrosquamatus*)

Keratin type	Name[1]	Genomic DNA sequence (acc. nr.)	Gene start[2] (nucl. nr.)	Gene end[2] (nucl. nr.)	Gene name (GenBank)	Gene (GenBank ID)	Protein[3] (acc. nr.)	Differences between new gene prediction and GenBank (GCF_001527695.1)[4]
**type I**	K222	NW_015386388.1	4579705	4563973	KRT222	107303117	XP_015687800.1	
**type I**	K24	NW_015386388.1	4593683	4585551	KRT24	107303090	XP_015687766.1	
**type I**	K24L	NW_015386388.1	4610502	4601245	LOC107303091	107303091	XP_015687767.1	Named K10-like in GenBank
**type I**	K10L1	NW_015386388.1	4628673	4615542	LOC107303119	107303119	XP_015687801.1	
**type I**	K10L2	NW_015386388.1	4639339	4631362	LOC107303022	107303022	XP_015687687.1	Incomplete N-terminus
**type I**	K20L	NW_015386388.1	4668627	4658157	LOC107303120	107303120	XP_015687802.1	Incomplete N-terminus
**type I**	K23	NW_015386388.1	4728213	4703693	LOC107303092	107303092	XP_015687768.1	
**type I**	K15	NW_015386388.1	4782878	4774150	LOC107303093	107303093	XP_015687770.1	
**type I**	K19	NW_015386388.1	4810269	4799955	LOC107303094	107303094	XP_015687772.1	
**type I**	K9L	NW_015386388.1	4852795	4844127	LOC107303023	107303023	XP_015687689.1	
**type I**	HAS1 (K9LC1)	NW_015386388.1	4946558	4939829	LOC107303121	107303121	XP_015687803.1	
**type I**	HAS2 (K9LC2)	NW_015386388.1	4974935	4966799	LOC107303122	107303122	XP_015687805.1	
**type I**	K14L1	NW_015386388.1	5009323	5000237	LOC107303095	107303095	XP_015687773.1	
**type I**	K14L2	NW_015386388.1	5025051	5018341	LOC107303123	107303123	XP_015687806.1	Incomplete N- and C-termini
**type I**	K117	NW_015386388.1	5088974	5072974	LOC107303024	107303024	XP_015687690.2	
**type I**	K18	NW_015386810.1	591188	599294	KRT18	107288181	XP_015672253.1	
**type II**	K80	NW_015387607.1	203655	243569	n.a.	n.a.	n.a.	Identified by BLAST between *Krt7* and *Faim2*
**type II**	K7	NW_015387607.1	281992	301511	LOC107292791	107292791	XP_015677400.1	
**type II**	K5L2	NW_015387607.1	380309	370750	LOC107292792	107292792	XP_015677401.1	
**type II**	K5L1	NW_015387607.1	415254	402945	LOC107292794	107292794	XP_015677404.1	
**type II**	HBS1 (K78LC1)	NW_015387607.1	438703	430012	LOC107292789	107292789	XP_015677398.1	
**type II**	HBS2 (K78LC2)	NW_015386143.1	23602	12950	LOC107284963	107284963	XP_015668587.1	Sequence ends after exon 6
**type II**	HBS3 (K78LC3)	NW_015386143.1	56136	42413	LOC107285009	107285009	XP_015668640.1	
**type II**	HBS4 (K78LC4)	NW_015386143.1	93963	74922	LOC107284973	107284973	XP_015668597.1	
**type II**	K78LT	NW_015386143.1	131703	122663	LOC107284982	107284982	XP_015668608.1	
**type II**	K78L4	NW_015386143.1	161149	148418	LOC107284991	107284991	XP_015668619.1	
**type II**	K78L3	NW_015386143.1	205835	180432	LOC107285020	107285020	XP_015668651.1	
**type II**	K78L2	NW_015390075.1	40293	45677	LOC107298626	107298626	XP_015683489.1	Sequence ends after exon 5
**type II**	K78L1	NW_015390075.1	17942	29969	LOC107298627	107298627	XP_015683491.1	Incomplete
**type II**	K8	NW_015386810.1	534700	518951	KRT8	107288180	XP_015672252.1	

Notes: [1] Keratins were named according to orthology to human proteins. K9-like cysteine-rich keratins (K9LC) are termed HAS and K78-like cysteine-rich keratins (K78LC) are termed HBS keratins. HAS, hard acidic sauropsid-specific; HBS, hard basic sauropsid-specific; L, like; LT, long tail.

[2] Gene start and end indicate the start and stop codon of the coding sequence. Introns are not indicated.

[3] Accession numbers (acc. nr.) of protein amino acid sequences predicted in GenBank.

[4] Genome assembly used for predictions: P.Mucros_1.0 (GCF_001527695.1).

acc., accession; n.a., not available in the genome sequence assembly; nucl., nucleotide; nr., number.

Supplementary Table S6. Keratin genes of the frog (*Xenopus tropicalis*)

Keratin type	Name[1]	Genomic DNA sequence (acc. nr.)	Gene start[2] (nucl. nr.)	Gene end[2] (nucl. nr.)	Gene name (GenBank)	Gene (GenBank ID)	Protein[3] (acc. nr.)	Differences between new gene prediction and GenBank (GCF_000004195.3)[4]
**type I**	K222	NW_016683386.1	1124475	1105206	krt222	100488319	XP_004918751.1	
**type I**	Frog1-02	NW_016683386.1	1154939	1150145	LOC100487269	100487269	XP_002940698.2	Corrected head domain
**type I**	Frog1-03	NW_016683386.1	1171666	1168635	krt10	100487422	XP_012808327.1	
**type I**	Frog1-04	NW_016683386.1	1201989	<1197543	LOC100158585	100158585	NP_001121485.1	
**type I**	Frog1-05	NW_016683386.1	1240852	1233754	LOC100488172	100488172	XP_002940703.2	Incomplete at both ends
**type I**	Frog1-06	NW_016683386.1	1254262	1245094	krt24	496660	NP_001011224.1	
**type I**	Frog1-07	NW_016683386.1	1272369	1264944	LOC101731128	101731128	XP_004918844.3	Incomplete
**type I**	Frog1-08	NW_016683386.1	1331129	1323043	xak-c	100486967	XP_002940696.1	
**type I**	Frog1-09	NW_016683386.1	1347940	1343996	krt12	100144436	NP_001116496.1	
**type I**	Frog1-10	NW_016683386.1	1369802	1364487	xk81a1	100145042	NP_001120056.1	
**type I**	Frog1-11	NW_016683386.1	1390695	1385231	LOC549413	549413	NP_001016659.1	
**type I**	Frog1-12	NW_016683386.1	1423486	1440842	LOC101730713	101730713	XP_004918757.1	Incomplete
**type I**	Frog1-13	NW_016683386.1	1455161	1449470	krt16	100499212	XP_004918756.1	
**type I**	Frog1-14	NW_016683386.1	>1473684	1462819	krt14	100127859	XP_012808153.1	
**type I**	Frog1-15	NC_030686.1	1610298	1573905	LOC100491563	100491563	XP_002939081.2	One exon is missing
**type I**	Frog1-16	NC_030686.1	1678655	1672884	krt35	100491736	XP_002939082.3	
**type I**	Frog1-17	NC_030686.1	1696854	1688820	krt15	100379888	XP_002939083.2	
**type I**	Frog1-18	NC_030686.1	1711958	1703070	LOC100490379	100490379	XP_004918754.1	
**type I**	Frog1-19	NC_030686.1	1738209	1727533	krt19	394514	XP_012827034.1	
**type I**	Frog1-20	NC_030686.1	1788322	1780124	LOC100490554	100490554	XP_012808156.1	Incomplete
**type I**	Frog1-21	NC_030686.1	1797326	1791399	LOC100490729	100490729	XP_002939076.3	
**type I**	Frog1-22	NC_030686.1	1812174	1807464	krt17	100490897	XP_002939077.1	
**type I**	K117	NC_030686.1	>1861983	1857265	krt70	448098	NP_001006687.1	Start of gene in sequence gap, first exon missing
**type I**	K18	NC_030678.1	144892767	144889352	krt18	394541	NP_988944.1	
**type II**	K80	NC_030678.1	145340325	145358155	krt80	100316911	NP_001163982.1	
**type II**	Frog2-02	NC_030678.1	145282277	145276908	krt5.7	100101751	XP_002935788.2	
**type II**	Frog2-03	NC_030678.1	145241155	145245991	krt5.3	100491329	XP_012813652.1	
**type II**	Frog2-04	NC_030678.1	145192735	145197334	krt5.2	779830	NP_001072377.1	
**type II**	Frog2-05	NC_030678.1	145173996	145182744	krt5.5	100489468	XP_002935776.1	
**type II**	Frog2-06	NC_030678.1	145153628	145161984	krt75	100491159	XP_002935786.2	
**type II**	Frog2-07	NC_030678.1	145145209	145150875	krt5.6	100489309	XP_002935775.1	
**type II**	Frog2-08	NC_030678.1	145131141	145137134	LOC100490994	100490994	XP_002935785.1	
**type II**	Frog2-09	NC_030678.1	145117276	145123151	LOC100490820	100490820	XP_002935784.2	
**type II**	Frog2-10	NC_030678.1	145095169	145106302	krt6a	100490652	XP_017947337.1	
**type II**	Frog2-11	NC_030678.1	145072999	145077895	LOC100489158	100489158	XP_002935774.1	
**type II**	Frog2-12	NC_030678.1	145055578	145062037	krt5.4	448361	NP_001006716.1	
**type II**	Frog2-13	NC_030678.1	145023433	145028461	krt6	100144701	NP_001166034.1	
**type II**	K8	NC_030678.1	144954443	144962883	krt8	431676	NP_001002797.1	

Notes: [1] Keratins were named according to orthology to human proteins or according to type and position of the gene (Figure 1) ("Frog-type-position").

[2] Gene start and end indicate the start and stop codon of the coding sequence. Introns are not indicated.

[3] Accession numbers (acc. nr.) of protein amino acid sequences predicted in GenBank.

[4] Genome assembly used for predictions: Xenopus_tropicalis_v9.1 (GCF_000004195.3).

acc., accession; n.a., not available in the genome sequence assembly; nucl., nucleotide; nr., number.

Supplementary Table S7. Keratin genes of the platypus (*Ornithorhynchus anatinus*)

Keratin type	Name[1]	Genomic DNA sequence (GenBank acc. nr.)	Gene ID (GenBank)	Protein (GenBank acc. nr.)	Number of cysteine residues	Notes
**type I**	K222	NC_041738.1	100074593	XP_028930428.1	2	
**type I**	K224	NC_041738.1	100091675	XP_028930435.1	1	
**type I**	K24L4	NC_041738.1	100091681	XP_001512134.1	6	
**type I**	K24L3	NC_041738.1	100091690	XP_028931081.1	6	
**type I**	K24L2	NC_041738.1	100091696	XP_028931091.1	6	
**type I**	K24L1	NC_041738.1	100091718	XP_028931269.1	2	sequence incomplete
**type I**	K25	NC_041738.1	100091723	XP_001520523.2	12	
**type I**	K26	NC_041738.1	100091728	XP_001512895.1	15	
**type I**	K27	NC_041738.1	100091669	XP_001513002.1	11	
**type I**	K28	NC_041738.1	100091735	XP_001520530.1	12	
**type I**	K10L2	NC_041738.1	100091742	XP_028931907.1	6	
**type I**	K10L1	NC_041738.1	100091750	XP_007662432.2	2	sequence incomplete
**type I**	K20L2	NC_041738.1	100091766	XP_028931908.1	1	
**type I**	K12	NC_041738.1	100081910	XP_028931359.1	2	
**type I**	K20L1	NC_041738.1	114815072	XP_028931098.1	4	
**type I**	K23	NC_041738.1	100091772	XP_007662433.2	5	
**type I**	K39	NC_041738.1	100091780	XP_028931100.1	27	
**type I**	K40	NC_041738.1	100091796	XP_007662434.2	35	
**type I**	K33a	NC_041738.1	114815087	XP_028931270.1	28	
**type I**	K33b	NC_041738.1	100074485	XP_007655203.1	23	
**type I**	K34	NC_041738.1	100074443	XP_001505493.2	30	
**type I**	K31	NC_041738.1	100088880	XP_001518440.1	32	
**type I**	K37	NC_041738.1	100088890	XP_001518445.2	26	
**type I**	K38	NC_041738.1	100083777	XP_028931108.1	19	
**type I**	K32	NC_041738.1	100083755	XP_007661319.2	25	
**type I**	K36	NC_041738.1	100083734	XP_001514298.2	10	sequence incomplete
**type I**	K13	NC_041738.1	100079550	XP_001510496.2	7	
**type I**	K15	NC_041738.1	100086149	n.a.	n.a.	pseudogene
**type I**	K19	NC_041738.1	107547508	XP_028931283.1	0	
**type I**	K9	NC_041738.1	100082561	XP_028931280.1	5	
**type I**	K14	NC_041738.1	100077389	XP_001511728.2	6	
**type I**	K16	NC_041738.1	114815097	XP_028931325.1	9	
**type I**	K17	NC_041738.1	100092093	XP_028932116.1	4	
**type I**	K18	NC_041737.1	114814771	XP_028930001.1	2	
**type II**	K80	NC_041737.1	100086395	XP_028929028.1	12	
**type II**	K7	NC_041737.1	100087958	XP_028929030.1	0	
**type II**	K81L2	NC_041737.1	103164921	XP_007654211.2	n.a.	sequence incomplete
**type II**	K81L	NC_041737.1	100092864	XP_028929027.1	33	
**type II**	K83L2	NC_041737.1	100092929	XP_028929031.1	34	
**type II**	K83L1	NC_041737.1	100092926	XP_028929024.1	31	
**type II**	K85L2	NC_041737.1	103164923	XP_028928757.1	18	
**type II**	K85L1	NC_041737.1	100089219	XP_028929033.1	25	
**type II**	K84	NC_041737.1	100090360	XP_028929015.1	19	
**type II**	K82	NC_041737.1	103164922	XP_028929034.1	21	sequence incomplete
**type II**	K75	NC_041737.1	114814612	XP_028929009.1	4	
**type II**	K6aL	NC_041737.1	100092858	XP_001514525.1	10	
**type II**	K5L	NC_041737.1	103168998	XP_028929007.1	4	
**type II**	K6AL	NC_041737.1	114814567	n.a.	n.a.	pseudogene
**type II**	K71	NC_041737.1	100092898	XP_028929017.1	9	
**type II**	K72	NC_041737.1	100092892	XP_007654210.1	10	
**type II**	K73	NC_041737.1	100092888	XP_028928758.1	12	
**type II**	K74	NC_041737.1	100092881	XP_001521385.3	10	
**type II**	K2L2	NC_041737.1	100092878	XP_028929018.1	4	
**type II**	K2L1	NC_041737.1	114814568	XP_028928759.1	6	
**type II**	K1	NC_041737.1	114814611	XP_028929002.1	3	
**type II**	K77	NC_041737.1	103164924	XP_028929014.1	7	
**type II**	K76	NC_041737.1	100073951	XP_028929006.1	2	
**type II**	K3L2	NC_041737.1	114814569	XP_028928760.1	4	
**type II**	K3L1	NC_041737.1	100074276	XP_001505912.1	7	
**type II**	K4	NC_041737.1	100078666	XP_028929023.1	9	
**type II**	K79	NC_041737.1	100088469	XP_028929019.1	10	
**type II**	K78	NC_041737.1	100084867	XP_028928761.1	8	
**type II**	K8	NC_041737.1	100078521	XP_001507306.1	0	

Notes: [1] Keratins were named according to orthology to human proteins. K, keratin; L, like.
Genome sequence assembly used for predictions: mOrnAna1.p.v1 (GCF_004115215.1).
n.a., not applicable

Supplementary Table S8. Number of cysteine residues in keratins of the kiwi (*Apteryx australis mantelli*)

Keratin type	Name	Protein (GenBank acc. nr.)	Number of cysteine residues
**type I**	K222	XP_025928687.1	3
**type I**	K12	XP_013801838.1	1
**type I**	K20	XP_013801841.1	0
**type I**	K23	XP_013801848.1	5
**type I**	K36L	XP_013801846.1	18
**type I**	K15	XP_013801843.1	6
**type I**	K19	XP_013801844.1	1
**type I**	K9L1	XP_013801847.1	5
**type I**	K9L2	XP_013801845.1	5
**type I**	K9L3	XP_013801842.1	9
**type I**	K9L4	XP_013801836.1	12
**type I**	HAS1 (K9LC1)	XP_013801837.1	28
**type I**	HAS2 (K9LC2)	XP_013801835.1	18
**type I**	HAS3 (K9LC3)	XP_013801834.1	21
**type I**	K14L1	XP_013801867.1	2
**type I**	K14L2	XP_013801833.1	10
**type I**	K18	XP_013805088.1	0
**type II**	K80	XP_013810925.1	9
**type II**	K7	XP_013810923.1	0
**type II**	K84L	XP_013810920.1	18
**type II**	K5L4	XP_013810918.1	6
**type II**	K5L3	XP_013810922.1	10
**type II**	K5L2	XP_013810921.1	3
**type II**	K5L1	XP_013810917.1	3
**type II**	HBS1 (K78LC1)	XP_013810916.1	35
**type II**	HBS2 (K78LC2)	XP_013810915.1	27
**type II**	HBS3 (K78LC3)	XP_013810914.1	16
**type II**	K78LT	XP_013810912.1	9
**type II**	K78L5	XP_013810911.1	11
**type II**	K78L4	XP_013810926.1	8
**type II**	K78L3	XP_013810927.1	8
**type II**	K78L2	XP_013810928.1	8
**type II**	K78L1	XP_013810929.1	7
**type II**	K8	XP_013813947.1	2

Notes: Keratins were named according to orthology to chicken proteins.
K, keratin; L, like; HA/BS, hard acidic/basic sauropsid-specific.
Genome sequence assembly: AptMant0 (GCF_001039765.1).

Supplementary Table S9. Number of cysteine residues in keratins of the ostrich (*Struthio camelus australis*)

Keratin type	Name	Genomic DNA/cDNA sequence (acc. nr.)	Gene ID (GenBank)	Protein (GenBank acc. nr.)	Number of cysteine residues	Notes
**type I**	K222	NW_009272154.1	104152437	XP_009686054.1	3	
**type I**	K12	NW_009272154.1	104152438	XP_009686056.1	1	
**type I**	K20	NW_009272154.1	104152454	XP_009686074.1	1	
**type I**	K23	NW_009272154.1	104152435	XP_009686053.1	3	
**type I**	K15	NW_009272154.1	104152453	XP_009686073.1	6	
**type I**	K19	NW_009272154.1	104152434	XP_009686052.1	1	sequence incomplete
**type I**	K9L1	NW_009272154.1	104152433	XP_009686051.1	7	
**type I**	K9L2	NW_009272154.1	104152452	XP_009686072.1	7	
**type I**	K9L3	NW_009272154.1	104152432	XP_009686050.1	8	
**type I**	K9L4	NW_009272154.1	104152431	XP_009686049.1	11	
**type I**	HAS1 (K9LC1)	NW_009272154.1	104152430	XP_009686048.1	28	
**type I**	HAS2 (K9LC2)	NW_009272154.1	104152429	XP_009686047.1	26	
**type I**	HAS3 (K9LC3)	NW_009272154.1	104152451	XP_009686071.1	17	sequence incomplete
**type I**	K14L1	NW_009272154.1	104152428	XP_009686045.1	2	
**type I**	K14L2	NW_009272154.1	104152450	XP_009686070.1	2	
**type I**	K18	NW_009270297.1	104152322	XP_009685921.1	0	
**type II**	K80	NW_009270297.1	104152362	XP_009685973.1	10	
**type II**	K7	NW_009270297.1	104152373	XP_009686015.1	0	
**type II**	K5L4	NW_009270297.1	104152571	XP_009686271.1	8	
**type II**	K5L3	NW_009270297.1	104152351	XP_009685961.1	3	
**type II**	K5L2	NW_009270297.1	104152346	XP_009685948.1	3	
**type II**	K5L1	NW_009270297.1	104152560	XP_009686260.1	1	
**type II**	HBS1 (K78LC1)	NW_009270297.1	104152550	XP_009686249.1	27	Gene ID: 2 incomplete coding sequences
**type II**	HBS2 (K78LC2)	NW_009270297.1	104152550	XP_009686249.1	19	Gene ID: 2 incomplete coding sequences
**type II**	K78LT	NW_009270297.1	104152547	XP_009686235.1	7	sequence incomplete
**type II**	K78L	NW_009270297.1	104152337	XP_009685942.1	12	
**type II**	K8	n.a.	n.a.	n.a.	n.a.	

Notes: Keratins were named according to orthology to chicken proteins.
K, keratin; L, like; HA/BS, hard acidic/basic sauropsid-specific.
Genome sequence assembly: ASM69896v1 (GCF_000698965.1).
n.a., not available.

Supplementary Table S10. Keratin genes of the zebra finch (*Taeniopygia guttata*)

Keratin type	Name[1]	Genomic DNA sequence (acc. nr.)	Gene ID (GenBank)	Protein[3] (GenBank acc. nr.)	Number of cysteine residues	Notes
**type I**	K222	NC_044239.1	101233916	XP_004174708.1		
**type I**	K12	NC_044239.1	100229407	XP_012426581.1	1	
**type I**	K20	NC_044239.1	100226488	XP_002194074.3	0	
**type I**	K23	NC_044239.1	101233949	XP_004174709.2	9	
**type I**	K15	NC_044239.1	100223619	XP_002194098.1	5	
**type I**	K19	NC_044239.1	100227535	XP_002197015.2	0	
**type I**	K9L1	NC_044239.1	101233334	XP_030112293.1	11	
**type I**	K9L2	NC_044239.1	100220698	XP_002194122.4	10	
**type I**	K9L3	NC_044239.1	100228906	XP_030112121.1	9	
**type I**	K9L4	NC_044239.1	100230285	XP_030112123.1	10	
**type I**	HAS1 (K9LC1)	NC_044239.1	100227476	XP_012426580.2	28	
**type I**	HAS2 (K9LC2)	NC_044239.1	100224590	XP_002194209.3	27	
**type I**	HAS3 (K9LC3)	NC_044239.1	100221664	XP_030112003.1	25	
**type I**	K14L1	NC_044239.1	100231284	XP_030112001.1	3	Gene ID: 2 coding sequences
**type I**	K14L2	NC_044239.1	100231284	XP_012426586.2	3	Gene ID: 2 coding sequences
**type I**	K18	NC_044242.1	115491360	XP_030115101.1	0	
**type II**	K80	NC_044242.1	115491353	XP_030115077.1	5	
**type II**	K7	NC_044242.1	100224644	XP_030115482.1	0	
**type II**	K5L4	NC_044242.1	100224585	XP_002195031.2	10	
**type II**	K5L3	NC_044242.1	100227468	XP_002195007.1	8	
**type II**	K5L2	NC_044242.1	100230278	XP_002194692.1	3	
**type II**	K5L1	NC_044242.1	100230328	XP_030115537.1	1	exon 1: nucl. 333626 - 332934
**type II**	HBS1 (K78LC1)	NC_044242.1	115491512	XP_030115539.1	35	
**type II**	HBS2 (K78LC2)	NC_044242.1	100217876	XP_030115112.1	36	
**type II**	K78LT	NC_044242.1	100226538	XP_030115078.1	75	
**type II**	K78L4	NC_044242.1	100229354	XP_030115113.1	15	
**type II**	K78L3	NC_044242.1	100232294	XP_002191799.3	14	
**type II**	K78L2	NC_044242.1	115491354	XP_030115084.1	11	
**type II**	K78L1	NC_044242.1	101233849	XP_004175950.2	15	
**type II**	K8	NC_044242.1	115491358	XP_030115098.1	0	

Notes: Keratins were named according to orthology to chicken proteins.
K, keratin; L, like; HA/BS, hard acidic/basic sauropsid-specific.
Genome assembly used for predictions: bTaeGut1_v1.p (GCF_003957565.1).

Supplementary Table S11. Keratin genes of the Tibetan frog (*Nanorana parkeri*)

Keratin type	Name	Genomic DNA sequence (acc. nr.)	Gene ID (GenBank)	Protein (GenBank acc. nr.)	Number of cysteine residues	Notes
**type I**	K222	NW_017306323.1	108804307	XP_018431528.1	4	
**type I**	Nanorana1_02	NW_017306323.1	108804320	XP_018431537.1	9	
**type I**	Nanorana1_03	NW_017306323.1	108804334	XP_018431542.1	4	
**type I**	Nanorana1_04	NW_017306323.1	108804356	XP_018431567.1	6	
**type I**	Nanorana1_05	NW_017306323.1	108804339	XP_018431544.1	12	
**type I**	Nanorana1_06	NW_017306323.1	108804340	XP_018431545.1	6	
**type I**	Nanorana1_07	NW_017306323.1	108804351	XP_018431561.1	3	
**type I**	Nanorana1_08	NW_017306323.1	108804364	XP_018431575.1	2	
**type I**	Nanorana1_09	NW_017306510.1	108787107	XP_018412066.1	4	
**type I**	Nanorana1_10	NW_017306510.1	108787113	XP_018412076.1	5	
**type I**	Nanorana1_11	NW_017306510.1	108787108	XP_018412067.1	3	
**type I**	Nanorana1_12	NW_017306510.1	108787122	XP_018412088.1	0	
**type I**	Nanorana1_13	NW_017306510.1	108787106	XP_018412065.1	0	
**type I**	Nanorana1_14	NW_017306510.1	108787114	XP_018412078.1	1	
**type I**	Nanorana1_15	NW_017306510.1	108787118	XP_018412084.1	0	
**type I**	Nanorana1_16	NW_017306510.1	108787115	XP_018412079.1	2	
**type I**	Nanorana1_17	NW_017306510.1	108787105	XP_018412064.1	6	
**type I**	Nanorana1_18	NW_017306510.1	108787121	XP_018412087.1	1	
**type I**	Nanorana1_19	NW_017306510.1	108787104	XP_018412063.1	4	sequence incomplete
**type I**	Nanorana1_20	NW_017306510.1	108787103	XP_018412061.1	4	
**type I**	Nanorana1_21	NW_017306510.1	108787102	XP_018412060.1	4	
**type I**	Nanorana1_22	NW_017306510.1	108787101	XP_018412059.1	4	
**type I**	Nanorana1_23	NW_017306510.1	108787100	XP_018412058.1	6	
**type I**	Nanorana1_24	NW_017306510.1	108787099	XP_018412057.1	7	
**type I**	Nanorana1_25	NW_017306510.1	108787123	XP_018412089.1	3	
**type I**	Nanorana1_26	NW_017306510.1	108787098	XP_018412056.1	3	
**type I**	Nanorana1_27	NW_017306510.1	108787097	XP_018412055.1	5	
**type I**	Nanorana1_28	NW_017306510.1	108787096	XP_018412053.1	3	
**type I**	Nanorana1_29	NW_017306510.1	108787095	XP_018412052.1	3	
**type I**	Nanorana1_30	NW_017306510.1	108787094	XP_018412051.1	3	
**type I**	Nanorana1_31	NW_017306510.1	108787086	XP_018412042.1	5	
**type I**	Nanorana1_32	NW_017306510.1	108787124	XP_018412090.1	4	
**type I**	Nanorana1_33	NW_017306510.1	108787093	XP_018412050.1	6	
**type I**	Nanorana1_34	NW_017306510.1	108787092	XP_018412049.1	8	Gene ID: 3 coding sequences
**type I**	Nanorana1_35	NW_017306510.1	108787092	XP_018412049.1	0	Gene ID: 3 coding sequences
**type I**	Nanorana1_36	NW_017306510.1	108787092	XP_018412049.1	3	Gene ID: 3 coding sequences
**type I**	Nanorana1_37	NW_017306510.1	108787091	XP_018412048.1	2	
**type I**	Nanorana1_38	NW_017306510.1	108787090	XP_018412046.1	3	
**type I**	Nanorana1_39	NW_017306510.1	108787089	XP_018412045.1	8	
**type I**	Nanorana1_40	NW_017306510.1	108787088	XP_018412044.1	2	
**type I**	Nanorana1_41	NW_017306510.1	108787119	XP_018412085.1	0	
**type I**	Nanorana1_42	NW_017306510.1	108787087	XP_018412043.1	2	
**type I**	Nanorana1_43	NW_017306510.1	108787116	XP_018412080.1	0	
**type I**	Nanorana1_44	NW_017306510.1	108787111	XP_018412073.1	4	
**type I**	Nanorana1_45	NW_017306510.1	108787112	XP_018412075.1	3	
**type I**	Nanorana1_46	NW_017306510.1	108787120	XP_018412086.1	1	
**type I**	Nanorana1_47	NW_017306510.1	108787109	XP_018412069.1	2	
**type I**	Nanorana1_48	NW_017306510.1	108787117	XP_018412082.1	2	K117 ortholog
**type I**	Nanorana1_K18	NW_017307321.1	108796078	XP_018422969.1	1	
**type II**	Nanorana2_K80L	NW_017307900.1	108799101	XP_018426427.1	5	
**type II**	Nanorana2_02	NW_017307900.1	108799102	XP_018426428.1	0	
**type II**	Nanorana2_03	NW_017307900.1	108799100	XP_018426426.1	3	
**type II**	Nanorana2_04	NW_017307900.1	108799097	XP_018426424.1	9	
**type II**	Nanorana2_05	NW_017307900.1	108799098	XP_018426425.1	5	
**type II**	Nanorana2_06	NW_017307530.1	108797348	XP_018424432.1	5	
**type II**	Nanorana2_07	NW_017307530.1	108797347	XP_018424431.1	4	
**type II**	Nanorana2_08	NW_017307530.1	108797345	XP_018424429.1	7	
**type II**	Nanorana2_09	NW_017307530.1	108797344	XP_018424428.1	5	
**type II**	Nanorana2_10	NW_017307530.1	108797356	XP_018424444.1	4	
**type II**	Nanorana2_11	NW_017307530.1	108797342	XP_018424426.1	2	
**type II**	Nanorana2_12	NW_017307530.1	108797355	XP_018424443.1	8	
**type II**	Nanorana2_13	NW_017307530.1	108797352	XP_018424442.1	5	
**type II**	Nanorana2_14	NW_017307530.1	108797349	XP_018424436.1	5	isoform x3
**type II**	Nanorana2_15	NW_017307530.1	108797351	XP_018424441.1	1	
**type II**	Nanorana2_16	NW_017307530.1	108797341	XP_018424425.1	1	
**type II**	Nanorana2_17	NW_017307530.1	108797350	XP_018424440.1	4	
**type II**	Nanorana2_18	NW_017307530.1	108797339	XP_018424423.1	13	
**type II**	Nanorana2_19	NW_017307530.1	108797340	XP_018424424.1	8	sequence incomplete
**type II**	Nanorana2_20	NW_017307321.1	108796077	XP_018422968.1	4	
**type II**	Nanorana2_K8	NW_017307321.1	108796080	XP_018422973.1	0	

Notes: Names indicate the keratin type and the position of the gene in the cluster, unless a name is suggested by GenBank.
Genome assembly used for predictions: ASM93562v1 (GCF_000935625.1).

Supplementary Table S12. Number of cysteine residues in keratins of the axolotl (*Ambystoma mexicanum*)

Keratin type	Name[1]	cDNA sequence (acc. nr.)[2]	Number of cysteine residues
**type I**	K17L	isotig63037	1
**type I**	K9L	isotig132511	2
**type I**	K15L1	istog85088	0
**type I**	K15L2	contig317039	2
**type I**	K15L3	contig327242	2
**type I**	K19L	isotig36490	0
**type I**	K24L1	contig365045	1
**type I**	K24L2	isotig29543	0
**type I**	K24L3	JK974491.1	1
**type I**	K24L4	isotig81597	4
**type I**	K18L1	contig330148	0
**type I**	K18L2	contig321256	1
**type II**	K80L	isotig85003	0
**type II**	K7L	isotig199907	3
**type II**	K5L1	isotig198655	1
**type II**	K5L2	isotig204938	1
**type II**	K5L3	isotig64160	0
**type II**	K5L4	isotig122526	4
**type II**	K8	isotig64108	2

Notes: [1] Preliminary names were given according to orthology to human proteins.
K, keratin; L, like.
[2] Sequences from: https://ambystoma.uky.edu/quick-links/blast - tBLASTn on V4.0-isotigs+Contigs

Supplementary Table S13. Number of cysteine residues in keratins of the caecilian (*Microcaecilia unicolor*)

Keratin type	Name[1]	Protein (GenBank acc. nr.)	Number of cysteine residues
**type I**	K23	XP_030077854.1	2
**type I**	K117	XP_030076825.1	0
**type I**	K17L	XP_030076598.1	1
**type I**	K19L	XP_030076445.1	0
**type I**	K15L	XP_030076444.1	2
**type I**	K24L1	XP_030077816.1	1
**type I**	K24L2	XP_030077793.1	1
**type I**	K24L3	XP_030077100.1	2
**type I**	K24L4	XP_030077098.1	2
**type I**	K24L5	XP_030076687.1	1
**type I**	K24L6	XP_030076596.1	1
**type I**	K24L7	XP_030076337.1	2
**type I**	K24L8	XP_030076336.1	4
**type I**	K24L9	XP_030076250.1	1
**type I**	K24L10	XP_030076248.1	1
**type I**	K24L11	XP_030076246.1	1
**type I**	K24L12	XP_030076243.1	1
**type I**	K20L1	XP_030076749.1	1
**type I**	K20L2	XP_030076338.1	1
**type I**	K18L1	XP_030066300.1	4
**type I**	K18L2	XP_030061931.1	3
**type II**	K80	XP_030054078.1	3
**type II**	K7L	XP_030054077.1	2
**type II**	K5L1	XP_030054076.1	1
**type II**	K5L2	XP_030054074.1	4
**type II**	K5L3	XP_030054073.1	3
**type II**	K5L4	XP_030054072.1	2
**type II**	K5L5	XP_030054071.1	2
**type II**	K5L6	XP_030054068.1	6
**type II**	K5L7	XP_030054066.1	5
**type II**	K5L8	XP_030054064.1	5
**type II**	K5L9	XP_030054063.1	3
**type II**	K8	XP_030054059.1	1

Notes: [1] Keratins were named according to orthology to human proteins.
K, keratin; L, like.
Genome sequence assembly: ASM69896v1 (GCF_000698965.1).

Supplementary Table S14. Number of cysteine residues in keratins of zebrafish *(Danio rerio)*

Name[1]	Protein (GenBank acc. nr.)	Number of cysteine residues	Notes
Danio_26CYS	NP_001002392.1	26	alternative name: krt95
Danio_02	NP_001003480.1	0	
Danio_03	NP_001017588.1	2	
Danio_04	NP_001034908.1	0	
Danio_05	NP_001035449.1	4	
Danio_06	NP_001038375.1	2	
Danio_07	NP_001070122.1	4	
Danio_08	NP_001070922.1	0	
Danio_09	NP_001076492.1	1	
Danio_10	NP_001096111.1	0	
Danio_11	NP_001107814.1	0	
Danio_12	NP_001186881.1	0	
Danio_13	NP_571182.1	0	
Danio_14	NP_571183.1	1	
Danio_K4	NP_571584.2	0	
Danio_K5	NP_571231.2	0	
Danio_K8	NP_956374.1	1	
Danio_K17	NP_001076574.1	0	
Danio_K18	NP_848524.1	0	
Danio_K18b	NP_956862.1	1	
Danio_K24	NP_998688.2	0	
Danio_K91	NP_001003445.1	0	
Danio_K92	NP_001017824.1	0	
Danio_K97	NP_001002383.1	0	

Notes: [1] Danio_26CYS contains 26 cysteine residues. Other keratins were numbered arbitrarily or according to GenBank.
Genome sequence assembly: GCF_000002035.6

A

>Chicken_K12
MDCGSPICPALLRAWQNSPALWSTMALSVRSSVGPRQFSSRSSLGGGSLRLSGNSTGGGFGGSGLGFGGG
SAGGFGAASVLGSGSGFSGGFGSSVGSGFGGGFASGSGGGYGSGSGGGFGGGFGSGFGSGSGIIFGGGFG
SGAAPGLGGGFGIGGGGDGGLLSGSKKETMQNLNDRLAAYLDKVRSLEDANTELERKIREWYEKNGPGAA
VPGSGNDYSKYYPIIEDLRNKIINATIDNARIILQVDNARLAADDFRLKYENEVALRQSVEADINGLRRV
LDELTLTRADLEMQIESLNEELAYLKKNHEEELQGLQSSAVGQVSVEMDAAPGIDLTKLLNDMRGQYEVI
AEQNRKEAEAWFNEKSGELKREISTNTEQLQSGKSEITDLKRTLQNLEIELQSQLAMKKSLEDTLAETEG
GYCAQLSQMQLQIGNLESQLLQVRADMERQSAEYQQLLDIKSRLEMEIETYRRLLDGEFVSGGQGVVFES
SSLTGSKSQTQSLDSSQDPTKTRKIKTIVEEVVDGKVVASHVKEVEEKI

>Chicken_K20
MSFSTQSSQLGTAAHLQGTAPGGSALSVRSAAVQRIQAPSVYGGAGGYGTRISSSSVRRQSSSGAFHSNI
SDNDLLLSGNEKSTMQNLNDRLAAYLERVRSLEKANSLLEKQIKEWHEKNTVGVRSDYSSYFQTIEDLQS
KIGAAQLENAKLVLQIDNAKLAADDFRLKYENELLLRQSVESDINGLLRVLDDLTLTKSDLESQIENVNE
EIVFLKKNHEEEVDRLRKQVGGSVNVEVDAAPGTDLASIMENMRRQYEEMAEKNRQEAKERFEKQTEALN
QEVAINVEQLQEQRREVTDRRQICQSLELELQTHLNMKKPLEDTLAETEARYSFQLAQIQETIAKLEAQL
RQLRADMEAQNTEYSVLLDIKTRLELEIATYRRLLEGEDVGLVQEDAVTTELEKESSKIKKIKTIVEEVI
DGKVVSSQVKEIEEKM

>Chicken_K23
MSTSQGPFQFYSGSLRGSAGCGLAQQGTSYRPPSADGASGTHTSFSSTRPLSLAAGGGASWGCFGEHHWE
SIFLGGNEKQTMQNLNDRLAAYLDKVRSLEAANAQLESCILEWHKTKSHGKRHDFNQYEQNVTDMQRQIE
DGKITNASILLQIDNANMASEDFRLKYEAEKTRRQGVQCDLENLRKELDGLTIITTDLEMEIEGLREEHI
LRRKDHEEDMEANRSSQDFKVNVKVNAPPPEDLTKILAEIREDYEAIIEKNRQSLDSWYKELSTAVSLAA
DPNPEQVQSSENEIKDLTRTLQSLDIELQAQMSKKHMLEDTLADTRNYYAVALQNMQQTISKCEEELSQL
RHDIRQQHNQYRVLLGIKTRLEKEISTYRLLLEGNVDRITRTCESKAEEYAELTNGKIKAIVHDSMNGQL
LSSTTAIHQEE

>Chicken_K15
MTTYTVQNSFASGAGGLGAAGSGSSRLSSVRVGGSYRTPSIHGGSGGRGVSISSTKYVSSVGGGYGGGSC
AGFGGGYGGGYGGFGGGAVCGLGGGAGFGGGFDAICAFGGGDGLLSGNEKITMQNLNDRLATYLQKVRAL
EDANAELEVKIRDWYQKEAPSSPARDYSNYYKIIEDLQDKILAATIDNSRVVLEIDNARLAADDFRLKHE
NELFLRQSVESDINGLRRVLDELTLSRADLEMQIESLKEELAYLKKNHEEEMKEYSNQLSGKVSVEMDAA
PGIDLTRILSEMREQYEALAEKYRKDAEAWFLTQTTELNHEVATHTEQIQSSKSEITELRRTMQSLEIEL
QSQISMKAGLEANLRDTEGRYCAQLAQIQNLITSVEEQLSEIRCDMERQNQEYRMLMDIKSRLEQEIATY
RQLLETQDSQMSGVNPKDASAAGRARGGTEDEGRSSSTRDRRHQ

>Chicken_K19
MATYSFRQTTSSVAGGPCGRSLRLGGGSFRAPSIHGGSGGRGVSVSSARFVSSGLGSGLGGGYGGAFSSS
FSAGFGGGYGGGLGSGDGLLSGNEKTTMQNLNDRLASYLDKVRALEEANSDLETKIREWYLKQGPGPARD
YSPYYKAIEDLRDQILAATIDNSKVVLQIDNARLAADDFKTKFETEQALRMSVEADINGLRRVLDELTLA
RTDLELQIENLKEELAYLKKNHEEEMSALGGQVAGQVSVEVDSAPGIDLSKILADMRDQYEHMAEKNRKD
AEAWFHSKTEELNREVAVNTEQLQSSKSEVTDLRRTLQGLEIELQSQLSMKGALESTLADTEGRYGAQLA
QIQDMIGSIEAQLAELRADMERQNSEYKMLMDIKTRLEQEIATYRQLLEGQESQLFGSLSGSPDKRDKPA
DGK

>Chicken_K9L1
MSCSIKRTSSTSYRSGGGGGICGGAGGRSSSTSCRRYASSVIGGGSSAGGACGIGYGGGMSAGSLAGGFG
GGLGGGFGGGFAGSFGAGGDILLSGNEKITMQNLNDRLASYLDKVRRLEEDNAQLELHIREWYRKQAPSV
SKDYSSYYQTIEQLQNQIISATVDNNRMLLDIDNSKMTADDFRMKYENELVIRQSVESDVNGLRNLLDDL
THTRSSLESELESLKEELIALKRNHEEELRQLQSQTGGDVSVEVNAAPGEDLTKILNDLRNEYEQIIEKN
RREVEQWYEVKIEEVNRQVTSSSQDIQTSSHQLTELKREMQNLEIELQAQLSTKNSLENSLAETESRYGC
LLQQIQGQINSVEEELANIRCEMEGQNQEYKMLLGIKTRLEQEIAQYRALLNEGQQDLAMPQGGGRTSHS
YSSTSYSHGQSGGDKSGQTRVC

>Chicken_K9L2
MSCSIKRTSSTSYRSGGGGGICGGAGGRSSSTSCRRYASSVIGGGSSAGGACGIGYGGGMSAGSLAGGFG
GGLGGGFGGGFAGSFGAGGDILLSGNEKITMQNLNDRLASYLDKVRRLEEDNAQLELHIREWYRKQAPSV
SKDYSSYYQTIEQLQNQIISATVDNNRMLLDIDNSKMTADDFRMKYENELVIRQTVESDINGLRNMLDDL
TLVRSSLESELESLKDELIALKRNHEEELRQLQSQTGGDVSVEVNAAPGEDLTKILNDLRNEYEQIIEKN
RREVEQWYEVKIEEVNRQVTSSSQDIQTSSHQLTELKREMQNLEIELQAQLSTKDSLENSLAETESRYGC
LLQQIQGQINSVEEELANIRCEMEGQSQEYKMLLGIKTRLEQEIAQYRALLHEGQQDLAASQGSFQGGGK
SSQSYSSSSFSQSQSGDVTGQSRVC

>Chicken_K9L3
MSCGTKSSSSTTRISSSGGGCGSGSGGGGGGGSSCGIRKSGGYSSVSTRKYTSSGGSGGFSGRTFGGGGS
KSSYGGGFSSGSCGRIGRSSYGRMSGGSYGGGMGGSMGGGYGSCGGGFGGMGGGYGGGGFGGGSGGYSGG
GFSGFGGSGGFGGGSYGGGSFGGVGFGEDSGLLSTNEKLTMQNLNDRLASYMDKVRGLEEENAHLERLIR
EWYQKQGPTGSKDYSQYYRTIEELQNQIVGANVDLNRMLLDIDNTRMTVDDFRLKYETEYTLHQSVASDI
NGLRPLLDQLTLSRSDLETQFESLKEELIYLKKNHEEEMRGLQTQSSGDVNVEVNATPGTNLMEKLNEMR
CEYERLIENNRREVESWYETKMEEVNQQVHSSGQEIQSSNQQISELRREYQSLEIELQSQLSMVDSLQSN
LEDTERRYNMQLQQIQGMIGPLEEELASIRCEMESQNEEYKMLLGIKTRLEQEIAQYRALLEEGQQDLVI
PAGGMGGGMGGGRIGGGGFSGGGRGGSGSICGGMGGGIGGGSGGIGGGSGGIGGGSGGIGGGSGGSCIIG
GGGSGGGGRTSGGVSSSHSYSSSSQSQSCRGGGESQGYGRKSFD

>Chicken_K9L4
MSCSRSSSSCLRGGSGGSQHGSASAHRYTASRAGGRPAGGSCSGGSSRSSCGATGFGRMRRSSYGGGMSS
GMGGSSYAGGWSAGTYGGTMRCGGMGGGFGAGGSGFGAGYGGGFGSGSAGFSAGSFGGGGFGGENMGILS
NNEKLTMQNLNDRLASYLDKVRSLERENARLEQLIREWYQKQGPTGRKDYSHYYTTIEDLQNQILTAAVE
TNKILLDLDNTRMTAEDFRTKYETEYGLRQNVEADINTLRPLLDSLTLSRTDLEMQFESLKEEMINLKKD
HEEDMKSLQTQSSGDVNVEVNAAPGEDLLKKLNDMRQEYEQIIQKNREEVESWYESKMEEVSQQVHSSGQ
ELESSNQQISVLRRDYQSLEIELQSELSRVGALQSNLEDTERRYNMQLQQIQGMIGPLEEELASIRCEME
SQNEEYKMLLGIKTRLEQEIAQYRALLNEGQHGISTSQGGAGGGSSGGHGHGGTSGSSAQGGSGGGSSWC
SRGRSSGGSGGKTSGGGGGGCGKASGGGGGSGGGEGGKGCLSRSSSSHSQSGSCESPGSFRRSEH

>Chicken_HAS1
MSCNIKETITVSSKGRSSGGSCIIGGGGGARISSYAIGSGRGFSGRSYCGGVNYGGGLSVGSLAGGSYGG
GNCYGNGLGFGLGGGVVVGGLGSDCLLSSCDEKVTMQNLNDRLASYLDKVKCLEKENAELECRIREWYAT
QGLSCEPRDYSCYYKEIEDLQNQIVCATIDNNKIILDIDNSRMAADDFRVKYETELALRQSVEADINGLR
QVLDQLTLCRSDLEAQLESLREELCCLKKNHEEEMNCLRKQSTGDVSVEVNACPGPDLRQILEDLRCQYE
TLIARNRKEVEDWYECKIEEVNREVITSGQEVETCNNQVTELRRQLQALEIDLQAQLSQRNNLESSLAET
ECQYNTLLGELQNQITCVEQQLAEIRAEIECQNQEYKTLLDVKCRLEQEIQTYRCLLEGGQQDLIHGGGI
GVGSGVGGGVIRTSHTYTTTTASHCQPQVPPCKTGDIQVTCRRICD

>Chicken_HAS2
MSSVSSGRYTTCGIGGSRGFSGRSYCGGVNYGGGLSSGSLVGGSYGGGLGAAVLGGCSGIGFSGGSARFG
GGIGGGLGISLGGGVVGGGFSGDGILLSGDEKVTMQNLNDRLASYLDKVRCLEQENADLECRIREWYAKQ
GPFCEPRDYSCYYKEIEDLQNQIVCATIDNNKIILNIDNSRMTADDFRVKYETELALRQSVEADINGLRQ
VLDQLTLCRSDLEAQLESLREELCCLKKNHEEEMNCLRKQSTGDVSVEVNACPGPDLRKILEEMRCQYET
LIERNRKEVEDWYECKIEEVNREVITSGQEVETCNNQVTELRRQLQALEIDLQAQLSQRDNLESSLAETE
CRYNNHLGELQTQITCVEQQLADLRAEMECQNQEYKILLDVKCRLEQEIHTYRCLLEGGQQDLIQQGGIG
QLSGLGGGVARTGGIGGGGIIRTSHTYTSSAQMPSCAAAEIQVPCRRICD

>Chicken_HAS3
MSCAQQCGTRGSAGSWAGTTQGSSASPGRQAAFELAAAARGFSGGSLGEGLLGQQLGAGSMSGGSFGATQ
RACSTLGFSTGGICVRGTGSFGRAGEGILFANNEKATMQNLNDRLASYLDKVRLLESENAELECRIREWY
AKVGPSCEPRDYSCFHKEIEDLQNQILCAAMETNKILLNIDNSQMTAEDFRAKYETECSLRQNVDADICN
LRPVLDQLASCKADLQLQCEAVTEEMCCLRANHEEEMSCLRKQATGDVSVEVNACPGPDLRKILEELRCQ
YETLMERNRKETEQWFACKVEEVNLEVITSSQEIESSNKQVTELRRQLQGLEINVQAQLTMKENLESSLA
ETECRYNKYLSELQSQISCVEQRLAEIRAEMECQNQEYKSLLDVKCRLEQEIHTYRCLLEGGQQDISTCS
TSGTGGTHGTTLRGPRHCSARRPHLG

>Chicken_K14L1
MSTTVRQFSSSTSLKGLGGGSSRLSCGRAAGYRAPSVHGGSSSYSVSSRIVSGLGAGYGGGYCSSVGGGL
GGGFGASYGAGYGAGFGGGFGAGFGGGFGGGDGILPAGEKETMQNLNDRLAAYLDKVRALEEANTDLEVK
IREWYKKQGPGPERDYSPYYRTIEELRSKILAATVENANIVLQIDNARLAADDFRTKFETEQALRLSVEA
DINGLRRVLDELTLSRADLEMQIENLKEELAYLKKNHEEEMNALRGQVGGEISVEMDAAPGIDLTKILAE
MREQYESLAEKNRRDAEQWFFSKTEELNREVAVNTEQLQSGKTEITELRRTIQSLEIDLQSQLSTKAALE
GTLADTEARYGTQLAQLQALITSVEEQLAELRCDMERQNHEYRVLLDVKCRLEQEIATYRRLLEGEDAHI
SSQYSSAMSSHSGRDAITTSRQVRTIVEEVQDGKVVSSREQMALTTR

>Chicken_K14L2
MSTTVRQFSSSTSLKGLGGGSSRLSCGRAAGYRAPSVHGGSSSYSVSSRIVSGLGAGYGGGYCSSVGGGL
GGGFGASYGAGYGAGFGGGFGAGFGGGFGGGDGILPAGEKETMQNLNDRLAAYLDKVRALEEANTDLEVK
IREWYKKQGPGPERDYSPYYRTIEELRSKVLGATVDNANLLLQIDNARLTADDFRTKFETEQALRLSVEA
DINGLRRVLDELTLSRADLEMQIENLKEELAYLKKNHEEEMNALRGQVGGEISVEMDAAPGIDLTKILAE
MREQYESLAEKNRRDAEQWFFSKTEELNREVAVNTEQLQSGKTEITELRRTIQSLEIDLQSQLSTKAALE
GTLADTEARYGTQLAQLQALITSVEEQLAELRCDMERQNHEYRVLLDVKCRLEQEIATYRRLLEGEDAHM
SSHYASQPVKEGPVTTRQIRTIFEEVQDGKVISSREQITQAAL

>Chicken_K18
MSLSRSTTFSTSSRTGSIRRLGPHSSAASVYAGAGGSGSRISITRTASTYGGGYGGGYGGGYGAGYGGGL
LLSGSGVVQNEKETMQDLNERLATYLEKVRSLENNNRRLEAQIRESLAKRGPSTRDWGNYWDTIQQLRDK
IYDSAVENARTVLQIDNARLAADDFRVKYEAELAIRTSVESDISGLRKVLDDTNMARLQLEGEIEALREE
LIFMKKNHEEEVNALQGQAASAGLTVEVDAPQAQDLGQALAELRAQYDALAKKNVEELEKQWGQQISETT
LEVTQSTKEVDTARSRLLELRRSVQSLEIDLDALRSQKRGLEGSLAETEARYGAQLEQLQGLILRGEEEL
AQARAELQRQNREHCALQDAKSKLEAEIATYRALLEGGEGFSLQDALGSEGTVTTQRSTQRMVDGKVVSE
SKEVKVRSY

>Alligator_K24
MSVWLSSGSRWISSPACGGGSVKMTGTGGSFDTGSVCGFGGGSHPGFSTGLTRGAGGGFSGGYGGGFGSI
SAGGSYGGTLCSSSFGGLGTGFGGGFGGPDSNLLSGGEKETMQNLNDRLANYLDKVHALEEANADLEYKI
HQWYEKHGPAAAELTRDYSKYYQIIEDLRNQIISATIGNASIVLQIDNSQLAAEDFRLKYENELFLHRSV
EADSNGLRRVLDDLTIAKSNLEAQLESLTEELVYLKKSHEEEMKSLKSTSTGDVNVEINAAPGIDLTKLL
NDMRAQYESLAEQNRREAEEQFNKMSQPLQKQIYDDAGAVSSATNELTEMKRTIQGLEIELQTVMAQKCS
LEGTLARTEENYSAQLSQRQLHISNLEEQLQQIRSEMECQNAEYEQLLGIKIRLEMEIETYKRLLDGEGG
GFGSGGYGHQESRVQIKEPYKTQVIKTIVEELVDGKIVSSEVKSVEEKPVK

>Alligator_K10L
MSFFGCGSRQISSCHTGGASMKIPCWGVYGLGGGSGLGMYETGGNWCGGLGWGGIGGGTAGSFHNFSYGA
VGNNSSDGRLLFGGEKETMQNLNDRLASYLGKVQALEEANAELEHKIKDWYETLGSYTEGGSRDYSKYYT
TIEDLRNQIIAATVENASIILQTDNARLAVDDFKVKYENELYLRQNVEADIRGLRKVLDEQTLAKSNLEI
QIESLADEVAYLKKNHEEEMNDLRSSASPDVNVEINTAPGIDLTTRLNKMRTEYEALAEQNRKDVETWFN

EKSKELNVQINFSAEETNTNKSQITELKRTFQGLEIEMKSQLALKQSLEATLAETEARYCAQLSQIQVVI
SSVETQIQQIRDDMEWQNAEYEQLLDIKIHLENEIETYRHLIDREESDSGYDTAGTGSGLRGTGSATTSG
SQGSVSAADLGSRGSGSARSESRSSSSSKKPTKTRVIKTIIEELDQHGTVTSSKVQSVEEKPII

>Alligator_K12
MALSVRTGGGSRQFSSRSALGGGSIRLFNSSGGGSFGSSGFGFGGGSSGGFGVASLLGSSSGFSGGFGSG
SGGGLGSGFGGGFGGGSGSGFGGGVGNSFGGGFGGGSGAGFGGGLGNAFGGGFGGGSGAGFGSGFGAGGG
SGDGGLLSGTKKETMQNLNDRLAAYLDKVRSLEEANTELEHKIREWYEKNGPGAGSLGAGNDYSKYYPII
EDLRNKIINATIDNARIVLQIDNARLAADDFRLKYENEVALRQSVEADINGLRRVLDELTLTRADLEMQI
ESLNEELAYLKKNHEEELQGFQGNATGQVSVEMDAAPGIDLTKLLNDMRGQYEAIAEQNRKDAEAWFNEK
SGELKKEISTNTEQLQSGRSEITDLKRTLQSLEIELQSQLAMKKSLEDTLAETEAGYCTQLSQMQLQIGN
LENQLFQVRADMERQNAEYQQLLDIKTRLEMEIETYRRLLDGEFGTGGQGLSFESSSLTGSKSQTQSLDS
SQDPTKTRKIKTIVEEVVDGKVVSSQVKEFEEKM

>Alligator_K20
MAFSTRSFHRSTSSTFQAPAPSLSSSAYRKIGIQKYQAPSVYGGAGGYGTRISTSTKYGGGFGTGSGNTF
QFNVIGNDLFAGNEKLTMQNLNDRLASYLEKVHSLEKTNSQLEKQIREWYANNSSSTRQDYSPYYNTIEE
LRNKIGAAQLENARLILQIDNAKLAADDFRMKYESELAIRQGAENDTAGLLKVIDDLTLTKADLESQVEG
MTEELAFLKKNHEEEVDRLRKQLGGTVNVEVDAAPSIDLAKIMENMRQQYESMAQKNRQEAKDRFDRQTE
ELNQVVAVNTEQLNSKKTEITDLKRTFQSLEIELQSQLSMKKALEDTLAETEARYGSLLAQIQAAIGNVE
GQLVQVRADMERQNNEYNILLDVKTRLEMEIATYRRLLEGEDIGSRQSELSKAEYEKEINKIRKIKTVVE
EVVDGKVVSSKVKEIEEKM

>Alligator_K23
MSSSQGPFQFSSGSLRSTARYNLAHIESFYRPSSTGAGVSSMHASLFSTRPPGVGASWGSTGASHGDSIF
LGGNEKQMMQNLNDRLAAYLDKVRSLEAANAQLESCIVEWHKKRSHGKRYDFNQFEQSIADMQGQIQDGK
ISNASVLLQIDNAKLASEEFRLKYEAEKYQREGVQFDVENLRKELDNLTIVTTDLEMEIEGLREEHILRK
KDHEADMHEQRSTKDVNVNVKVKATPPEDLTKILAGIREDYEAIIEKNRRGLDVWYKQQTSAASLAVTNS
PEQLQNNDKEIKNLKRNFQALEIELQAQLSKKHALENTLSETRAHYASRLQNIQEFISKCEEELSELRHD
VRCQSNKYKVLLGIKTRLEREISTYQLLLEGRTDEITRNFESSAKEHTASTDHRLKTIVQDSVDGQVVST
TIHEIHHWQDLWKIVAKFGCLQKFITVVWQFYDGVLAHVVDDKAFHECDTGIDISYQTEGKLFNLRRLQV
KTKLQEVTVCDLLFDDDCSLNARSESDM

>Alligator_K36L1
MLFKGCTPASTWGFIRSSCKVPYISCAKSVSVCSSGIRSQLPASYSINYYQPNICLPNLYCAAPYFSSSS
QTNGNLLPTCLSPASVPSSCILSSARPINCYLDGSYCRYNEAIFSSDGKETMQLLNDRLASYLEKVKSLE
KENTDLERKIREWQECQTPLVCTDFSSCYSTTEELQQQICSARAENARLRLDIDIVKVASDDFSFKYEYE
CGLCQSAEADISSLHRFLDDLTLCKTDLETELESLTEELLCLRKNHEEEASPLRHQLGDCINVELDTAPS
CDLNKVLEEIRCQYETLIEKNQCEVEDWYHTQMEELNKEVISDEEQLQGCQNEIIQLKHTVQALEIDLQT
QHSLNTALESTLTDTEARYSHQLSELQCLISNMEAQLAELRSNIQHQNSEYKVLLDVKSQLDCEISAYRR
LLDEEDCKAYHTSDTQTQIC

>Alligator_K36L2
MTSNCCTPDPCCGSFTDSCKAPSNSYTKSISDCSSDIKSCLPTSFSTSSKVSVSSRPGSSLVNSYQPTP
CLPRKLVRPMGCYSAGSYCGYNESIFNSNEKETMQFLNDRLACYLEKVRFLEQENADLEYKIREWYECQ
TPYVYTDFNCYYKMIEELQQQICCAKADNARLCLDVDNLKVASDDYATKYEYEHGLCQSAEADVKSLRR
ILDDLTLCKADLETELESLTEELLCLRKNHEEEASPLRRQLGDRINVELDAAPSCDLNKVLEEIRYQYE
TLVDKNQCEVEDWYHTQMEELNKEVISSGEQLQGYQNEIIQLKHTVQALEIDLQTQHNLNTALENTLAE
TETHYSQQLSELQCLVSDVEVQLAELRSDIEHQDGEYKVLLDAKCQLDCEISVYHSLLDVEDCRLPNYP
ANPGCAPSISSAGGKPVWSEPIQSPVFPSDCTPSIPAAEPNLIQPCCNPCAPTSPLTPGCALVTQISTQ
IC

>Alligator_K36L3
MTSKYCTHTISSRSGKGSCVTGGAFPWLSSVCSSSPSRFPGLSYGGRNISASSVKLGSSGLGNWLPSSFL
PSGYYGPGFAGWCDEGIFNSNEKETMQFLNDRLACYLEKVRHLEQENADLECKIREWYECQVPYVCTDFQ
AFYKTLEELQQQILCAKTNNAKMVLQIDNARLAADDFRTKFESEQVIRQSVDADINGLRRIMDEFTLCRA
DLEMQLESLKEELLCLKKNHEEEASVLRCQLGARVNVEVDAAPSCDLNKVLNEIRCQYENLVENNRKDVE
AWFSTKMEELNQQVISSGEQLQCCQTEITDLRRTIQALEIDLEAQTSMKSTLECTLHETENRYASQLAHL
QCLITNVEAQLAELRCDIERQNQEYMVLLDVKTRLETEIATYRRLLEGEDCKLPSFPCVPDCAPPSQITT
KIRTITEEIKDGRVISSRKQVQHCPPLVL

>Alligator_K15
MATTFMRSSSSSYGGGGLGCGVGGGGSSRVSSVRMGGSYRAPTIHGGSGGRNIAVSSARYASSVAGGYGG
GLGGSLGGGYSSGFGGDYGGSAFYGGIGGGAGGGYGGGFGGFDFGGGFGGGLGGGDGGVLSGNEKITMQN
LNDRLASYLDKVRALEEANADLEVKIRDWYQKQGPTSPARDYSPYYKTIEDLRDKILAATIDNSRIILEI
DNARLAADDFRMKYENELYLRQSVEADINGLRRVLDELTLARADLEMQIENLKEELAYLKKNHEEEMREY
SNQMTGKVSVEMDAAPGVDLTKVLAEMREQYEALADKNRREAEAWFFTKTEELNREVATSTEQIQTSKSE
MTELRRTLQGLEIELQSQLSMKAGLEANLADTESRYCAQLAQLQAMITSIEEQLAELRCDMERQNQEYKM
LLDIKTRLEQEIATYRRLLDGQDSQLSGSGWAPKEAGFSSSSSIKVRVEDSVDGKVVSHDRKY

>Alligator_K19
MSTFSLRPTTSSMSGVRGSSSIRFGGGSYRSPSIHGGSGGQRVSVSSARFVSSGAGGGLGGGYGGGFGSS
FSGGFGGGFGGSLGGGDSLLSGNEKITMQNLNDRLASYLDKVRALEEANSELEIKIRDWYQKQGPGPSRD
YSPYYKTIEDLRDKILAATIDNSKIVLQIDNARLAADDFKTKFETEQGLRLSVEADINGLRRLLDELTLA
RTDLEMQIENLKEELAYLKKNHEEEMNAHAGQAGGQVSVEVDSAPGIDLTKILAEMRDQYEFMAEKNRKD
AEGWFNSKTEELNQEVAVNTEQLQTSRTEITDLRRTLQGLEIELQSQLSMKAALEGTLADTENRYGAQLS
QIQCLISNIEAQLGDLRAEMERQNSEYKMLMDIKTRLEQEIATYHQLLEGQDSQLFGLSGTTDSKRGGKG
DK

>Alligator_K9L1
MSCSIKKTSSCSYRSGGGGGFGSCGGGSSSISTKKYTSSVAGGSTYGGGRLGGGSYGGGACSIVYGGGVS
GGSIGGGQGSAFCGGYGGGFGGGFGGGFGGGDGALLTGNEKTTMQNLNDRLATYLDKVRRLEEENADLEE
RIREWYRKQGPSISKDYSPYYQTIEQLQNQIISATVDNNKLILDIDNSKMIADDFRMKYENELAIRQGVE
ADINGLRNFLDELVRVRSTLELEVETLKDELLTLKKNHEEEMRLLQSQTGGDVNVEVNAAPGQDLTKILN
DMRQEYEEIIAKNRREVEQWYEVKMEEVNQQVTSSGQEVQTSSHQLTELRRVFQSLEIELQTQLSMKASL
ENNLAETEARYGAQLQQIQAMINCVEEELAQLRCEMESQNQEYKMLLGIKTRLEQEIAQYHGLLQEGQQD
ITSSQGGIQGGGRSSQSYSSHSCSSTSHSQSHGGEMTGQTRPC

>Alligator_K9L2
MSCGIKSSSSSSRIISGGGGGGGGGGGRRCVGYSSVSSRKYTSSGRSGGGGGFSGRSYSGGGSRSSYGGG
FSSGSCGRISRSCYGGRISGGSFGPSGGGICGGYGGGAGGFGGGGAGFGGGYGGGLGPCGFGGSGGFSGG
SFGGGGFSGGSFGGGGFSGGSFGGGGFSGGSFGGGGFSGGSFGGGGFSGGSFGGGGFSGGSFGGGGFSGG
SFGGGGFSGGSFGGGGFSGGSFGGGGFSGGSFGGGGFSGGSFGGGGFSGGSFGGGGFSGGSFGGGGFSGG
SFGGGGFSGGSFGGGGFSGGSFGGGGFSGGSFGGGGFSGGSFGGGGFSGGSFGGGGFSGGSFGGGGFSGG
SFGGGGFSGGSFGGGGFSGGSFGGGGFSGGSFGGGGFSGGSFGGGGFSGGSFGGGGFSGGSFGGDDTGFL
SNNEKMTMQNLNERLASYLDKVRHLEEENANLERLIREWYQNHGPQCGRDYSQYYGPIEDLQNQIISATV
DLNKVLLDIDNTRMTADDFRIKYETEYSLRQNVEGDINGLRPILDQLTLARSDLEAQYESLKEELISLKK
NHEEEMRGLQSQSGGDVNVEVNTTPDSNLMQKLNEMRCEYEQIIENNRKEVERWYETKMEEVNQEVHTSG
QEIQSSNHQVSELRRDYQTLEIELQSQLSMVQSLQSNLEDTERRYNIQLQQIQNTITTVEEELANIRCEI
ESQNYEYKMLLDIKCHLEQEIAQYRALLEEGQQDIGNQQGGRGGGGGFSYGGIRGGSIGGGGSIGGGTIG
GTCIGGGIGGGGIGGGSIGGGIGGGSIGGCGIGGGGGSGGSGSCVISGGGIGGGGGGGVKISGGGGGFSH
SYSSSSHGISSSS

>Alligator_K9L3
MSCGIKSSSSSSRIISGGGGGGGGGGGRRCVGYSSVSSRKYTSSGRSGGGGGFSGRSYSGGGSRSSYGGG
FSSGSCGRISRSCYGGRISGGSFGPSGGGICGGYGGGAGGFGGGGAGFGGGYGGGLGPCGFGGSGGFSGG
SFGGGGFSGGSFGGGGFSGGSFGGDDTGFLSNNEKMTMQNLNERLASYLDKVRHLEEENANLERLIREWY
QNHGPQCGRDYSQYYGPIEDLQNQIISATVDLNKVLLDIDNTRMTADDFRIKYETEYSLRQNVEGDINGL
RPILDQLTLARSDLEAQYESLKEELISLKKNHEEEMRGLQSQSGGDVNVEVNTTPDSNLMQKLNEMRCEY
EQIIENNRKEVERWYETKMEEVNQEVHTSGQEIQSSNHQVSELRRDYQTLEIELQSQLSMVQSLQSNLED
TERRYNIQLQQIQNTITTVEEELANIRCEIESQNYEYKMLLDIKCHLEQEIAQYRALLEEGQQDIGYVND

>Alligator_K9L4
MSGGSFGPSGGGICGGYGGGAGGFGGGGAGFGGGYGGGPGPCGFGGSGGFGGGGFSGSGGFGGGGFSGGG
GGFSGSGFSGGSFGGDDTGLLSNNEKMTMQNLNERLASYLDKVRRLEEENANLEQLIREWYQKQGPQGGR
DYSQYYKTIEDLQNQIISATVDLNKVLLDLDNTRMTVDDFRLKYETEYGLHQNVESDINGLRPMLDQLTL
ARSDLEAQYESLKEELISLKKNHEEEMKGLQSQSSGDVSVEVNAAPGTNLTEKLNEMRGEYERLIENNRR
EVESWFEAKMEEVNQQVHSSSQEMESSNQQVSELRREYQTLEIELQSLLSMLQSLQSNLEDTERRYNMQL
QQIQAMIIPVEEELANIRCEMESQNEEYKMLLGIKTRLEQEIAQYRALLEEGQQDIGNQQGSGGGYSSGG
IRGGSIGGVVPDATASQDVEYEKWTLD

>Alligator_K9L5
MSCSFKHTSKSYSLGGGCGRGGGFSSSSCRRYASSRASSGGGFSSRSFGGGSARCGYGGSYGGGMSSRSY
SGIGGFGCGSGGGFGGGSTGFGGGSTGFGGGSIGFGGGSIGFGGGNFGGGGGFGGDDTGFLSNNEKMTMQ
NLNERLASYLDKVRCLEEDNLQLERLIREWYQTHGPQGGKDYSHYYGPIEDLLNQIVLATVANNKIILDL
DNARMTAEDFRMKYETEYSLRQSVEGDINGLRPVLDNLTLAKSDLEMQFESLREELIQLKKNHQEEMSSL
QSESGGDVSVEVNAAPGEDLMGKLNEMRKEYEYIIQRNREEVERWYESKMEEVRHEVHTSGQEIESGNQQ
VSELRREYQRLEIELQSQLSTLQSLQSNLEDTDYRYNMQLQQIQNMIIPVEEELASIRCEMESQNEEYKM
LLGIKTRLEQEICQYRELLDEGQHDIGYIQGGGGGRGGHGGVSWSSAGGSGGGGGSGWSSCGGGGTSWSS
GGGCGTGGTGGRSCSKISGGGVCGGGNSSYCFASSSQPQPNKSEGQGE

>Alligator_HAS1
MSSGVKQAFTVSSSAKSSGGSCISGGGGGGSRMPLMLGRCVSGGIGSSKGFSSRSYSGGACYGGGISSGS
FAGGSYGGAGSIGGGHLGNIGFSGGSTCIGGGIGGGIGGGIGGGIVGGSIPDCGFDGGIVCGGPFGGGFG
GSAVGGGFGGSAVGGGFGGGAVGGGFGGGSGSGGFGSDGLLFCCDEKITMQNLNDRLASYLDKVRCLEEE
NAELECRIREWYAKHGPTCEPKDYSCYLREIEDLQNQIVCATIENNKIVLNIDNSRMAADDFRVKYETEL
SLLQSVEADINGLRQVLDQLTLCRSDLEAQLEALKEELCCLKKNHEEEMNCLRKQSTGDVSVEVNTCPGP
DLKQVLDEMRCQYEAIIAQNRKEVEDWYACKIEEVNREVISSGQEIECSNSQVCELRRQLQALEIDLQAQ
IRMRDNLQSSLAETECRYNNQLAELQNQIICVEQQLAELRAEIECQNQEYKVLLDVKCRLEQEIHTYCCL
LEGGHREFSSKGVISSGGISSGGISSGISHSSGIGGGGICKSSGGIIGGGGIVKTIHTHSSSSHCLPSPC
PPGDIKVPCRRLHD

>Alligator_HAS2
MSCGIKRTVTTVSSSAKGSGGSYGVGGGGGGARTSSVCSGKSISGGMGSSRGFSARSFGGGRCYGGGLST
GSLAGGSYGGAVGLCNMGNIGFSSGSTCVGGGFGGGGGFGVGGGSGFVGGIGGPIDCGFGGGFGGGFGGG
IVPGGGSGGDGLLFCCDEKITMQNLNDRLASYLDKVRCLEEENAELECRIRDWYAKHGPTCEPKDYSCYI
REIEDLQNQIVCATVDNNKIILDIDNSRMTGDDFRMKYETELALCQSVEADINGLRQVLDQLTLCRSDLE
AQLEALKEELCCLKKNHEEEINCLRNQSTGDVNVEVNSCPGPDLKQILDDMRCQYEAIIAQNRKEVEDWY
ACKIEEVNQEVISSSKEIECSNSQVSELRRELQALEIDLDSQLRMRDNLQSSLAETERRYNNQLAELQNQ
IICVEQQLAELRAEIECQNQEYKVLLDVKCRLEQEIQTYCCLLEGGQRELISQHGGISSGIIHSSGGGGG
ICKTSGGIIGGGGGGIVKTCHTYTSSSTCPPVDIQGPCRRVCD

>Alligator_K14L1
MTTSVRQYSSSTSLKGLGGFGSGSSRGSSVRLSGGAYRAPTIHGGSGGYSVSSSRMISGVGGGMGGGYGG
SYCSSVGGGFGGGSFGGGLGGGFGGGSGGGFGGGLGGGFGGGSGGGFGSGLGFGGPFGGGDGILRAGEKE
TMQNLNDRLAAYLDKVRALEEANSELEIKIRDWYKKQGPGPDRDYSPYYRTIEDLRNKILAATVDNANLI

LQIDNARLAADDFRTKFETEQALRMSVEADINGLRRVLDELTLARADLEMQIENLKEELAYLKKNHEEEM
NALRGQVGGEITVEMDAAPGVDLTKILTEMREQYEHLADKNRREAEQWFFKQTEELNQEVAINTEQLQSS
KSEITELRRTIQGLEIELQSQLSTKAALEGTLADTEARYGSQLAQIQGLITSVEEQLAELRCDMERQNHE
YKILLDVKCRLEQEIATYRRLLEGEDAHVPALYSSGLSSHSGRDVSSTRQVRTIVEEVQDGKVVSSREQV
HQTTR

>Alligator_K14L2
MTTSVRQYSSSTSLKGLGGFGGGSSRGSSVRLSGGAYRAPTIHGGSGGYSVSSSRMVSGFGGGLGGGYGG
SYCSSVGGGFGGGNFGGGSFSGGSFGGGFGGGLGGGFGGGSGGGFGGGLGFGGPFGGGDGILPAGEKETM
QNLNDRLAAYLDKVRALEEANSELEIKIRDWYKKQGPGPDRDYSPYYRTIEDLRNKILSATVDNANLLLQ
IDNARLTADDFRTKFETEQALRMSVEGDINGLRRVLDELTLAKADLEMQLENLSEELAYLKKNHEEEMNA
LRSQVGGEITVEMDAAPGVDLTKVLAEMREQYEHLADKNRREAEQWFFKQTEELNKEVAINTEQLQSGKS
EIMELRRTIQGLEIDLQSQLSMKAALEGTLADTEARYGSQLAQIQGLITSVEEQLAELRCDMERQNHEYK
ILLDVKCRLEQEIATYRRLLEGEDVHMSSQYSTQFIKEGPKTTRQVRTIVEEVQDGKVISSREQVHLSPR

>Alligator_K117
MASSLSRNLFCFGVPQGPRLNWISAAGLQPGLLSIHGGAGSIGSYLSSASDAFVKCVPGGSYEPGIGSGA
SGIGLLGMNEKDTMQNLNDRLAAYLERVCSLEEANGQLEQRIQELQVKKTLARHYDPSSCFSTIAELRSQ
IQDESVRNSQLTLQLDNAKLAANDFRMKYEAELATRLGVESDIKGLRKVLDGLTEARASLQVQIESLKEE
LTYLQQNHGEEVAALQGHLGGSVSVEVDASPGINLVKTLAEIRDQYEDIIEKNQREAEAWHKQQCETFTQ
EVATSSEALQAARTKATELKRIVQSLEIEFQMLQSTKEALEGTLAETESHYGVEMAQLQNLVAGKEAELA
QLRTDTQRQANEYQHLLDLKTRLEVEIATYQHLLEGQEVRSEAKSPKQEILPEAVSKAPTTRRIKTLIEE
LVDGEVVSSCIEEVEHPL

>Alligator_K18
MSISRSVYSSTRRPVSSSQGFTTTRKTTAASVYAGAGGSGSRISMPWGSRGVYGANSIYLGASGGSGGGY
GASGIYLGGSAVIHNEKETMQDLNDRLASYLEKVRSLETANHNLEVQIREFLDKKGPSTQDWTHYWDIIE
DLRNKIYEESVDNARIVLQIDNARLAADDFRVKYEGELAIRQSVEADIAGLRKVIDDTNMSRLQLESEIE
NLKEELIYMKKNHEEEAKSLQAQVSGSGLTVEVDSPKTHDLGKIMTDIRAQYEALAQKNREDLDKYWNQQ
ITESTIEITQSTKDMDTAHGTLTELRRTYQTLEIELESLRNLKGSLEANLMEVENRYGMQLEHLNGLLLR
TEAELMQVRTEVQRQAEEYQALLNIKVKLEAEIATYRRLLEGGEEFSLKDALAEDSSHKVYIQTSKQIVD
GKLVSETTETRALK

>Turtle_K24
MSLRIASGSRKVSSFGGGGGSVRVSRIGGSFGSGSVCGLAGGSGVGLGSGLGGGAGGSFGGGFSGGFGNV
SVGGGYGDGYCRSSFGGLGSGYGGGFSGADGSLLSGGEKGTMQNLNDRLAKYLDKVRALEEANADLEHKI
RQWYEKHDPAAAGLRYDYSKYYQIIEDLKNQIISATIDNAKLVLQIDNAKLAVDDFRLKYENELFLHQSI
EADINGLRRVLDDLTMTRSNLEAQLESLTEELVYLKKNHEGEMKSFQHGSTGEVNVEINATPGVDLTKLL
NEMRAQYEYLAEQNRREAEEQFNKMSQQLQQQISDDAGAADSARHEITEIKRTLQTLDIELQSLLAKKCS
LEGTLAEIEGNYGCQLSQIQLQISNLEEQLKQIRSEMECQNAEYEQLLGIKTRLEMEIETYRRLLNGEGG
GFRSGGYEYQESRVQIKEPSKTRVVKTIVEELVDGKIISSKVQSVEERPTK

>Turtle_K12
MALSVRTGGGSRQFSSRSGLSGGSVRISSSSGAGGFGGGSGGGFGVASLLGSSSGFGGGFGSSSLGGGLG
GSFGGSSVGGFGGSFGGGLGGGFGSGSGAGFGGGLGGGFGSSSGAGFGGGFGAGGGGDGGLLSGTEKETM
QNLNDRLAAYLNKVHSLEEGNTDLEHKIREWYEKNGPGAGGPGAGHDYSKYYPIIEDLRNKIINASIDNA
RIVLQIDNARLAADDFRLKYENEAALRQSVEADINGLRRVLDELTLTRADLEMQIESLNEELAYLKKNHE
EELQGFQGNATGQVSVEMDAAPGIDLTKLLNDMRGQYEVIAEQNRKEAEAWFNEKSMELKREISTNTEQL
QSGKSEITDLRRILQGLEIELQSQLAMKKSLEDTLAETEASYCAQLSQIQLQIGNLESQLFQVRADMERQ
NAEYQQLLDIKTRLEMEIETYRRLLDGEFGNGFAGGQGGSFESSSLTVSKSQTQSLDSSNDPTKTRKIKT
IVEEVVDGKVVSSHVKEIEEKM

>Turtle_K20
MAFQSRNYLTGTSSIFQPVAPSLSGSTYRKTAIRKYQAPSVYGGAGGYGTRISTSTNYGGGFGGGFGSGF
QLNTSGSDILLAGNEKLTMQNLNDRLASYLEKVHFLEKANSQLEKQIREWYANSSSTTRHDHSPYFKTIE
DLQNKIGAAHLENARLLLQIDNAKLASDDFRMKYENELAIKQNVENDLAGLLRVLDDLTLTRSDLALQIE
GLNEELTFLKKNHEEEVGELRKQLGGTVNVEVDAAPSIDLAKIMENMRQQYDSMAEKNRQEAKDRFDKQT
EEVNQEVAINIEQLQTKKTEITDLRRTFQGLEIELQSQLSTKKALEDTLAEMEARYGSQLAQIQAAIGNV
EAQLMQLRADMQSQSTEYSTLLDIKTRLEMEIATYRRLLEGEDSSRHFQTEISTIEEPEREINKIRKIKT
VVEEVIDGKVVSSETKEIEEKI

>Turtle_K23
MNSNQGRFQFSSGSHRGSAGCDLALRGSSYRPSSAHGGAIGTSVSISSTRPLWLASGVGVSWGAFGTSHG
DSIFLGGNEKQTMQNLNDRLAAYLEKVRSLEAANAQLESCILEWHKKRSHEKKHDFNQYEQNITDMQGQI
EDGKITNASIFLHIDNAKIASEDLSLKYEVEQSLRQGMQISVENLRKELDNLTIVTTDLEMEIEGIQEEQ
ILRKKDHEKDMGEHHSSQDFKVNVQVNAVPKEDLAKILAGIRKDYEAIIEKNHQGLEVWYKEQTATVSPA
TNISPEEIQSNESEIKNLRRTFQALEIELQAQFSKKYALKGTLAETQAHYTFQLQNIQQSISNCEEELSQ
LRQDIKCQNNHYKILLGIKTRLEKEISTYRQLLEGTADGITKTFESSTKEHAGMTSQKLKTVTQDSVNGQ
VVSSTTNEIDQQM

>Turtle_K15
MATSFMQSSSSNYGGGLGIGGGGSSRVSSVRMGGSYRAPSIHGGSGGRGVSVSSARYVSSGGGYGGGLGS
SYGAGYGSGLGGGYGGSSGFYSGFGGDAGGCFGGGAGGGFGGGAGGGFGGVYDFGGGLGGGDGGLLSGNE
KVTMQNLNDRLASYLDKVRALEQANTDLEIKIRDWYQKQGPTSPDRDYSPYYKIIDELRDKILAATIDNS
RVILEIDNARLAADDFRLKYENELFLRQSVEADINGLRRVLDELTLSRADLEMQIENLKEELAFLKKNHE
EEMKEYSNQMGGKVSVEMDAAPGVDLTSILSEMREQYEILADKNRRDAEAWFYKQTEELNREVATSTEQI
HTSKSEITELRRTLQGLEIELQSQLSMKAGLEANLAETEGRYCAQLAQLQAMITSIEEQLAELRCDMERQ
NQEYRMLLDIKTRLEQEIATYRRLLEGQDSQLSGWSPKDASLSSISSSSSGKVRISVEDSVDGKVISTHE

RKY

>Turtle_K19
MSTYSSKQITSSGLGNISGSSLSRAVGSFRAPSIHGGSGGRGISVSTARFVSSGVGGGLGGGYGGGLYGG
FGGGFGGGDGLLSGNEKSTMQNLNDRLASYLDKVRALEEANSELEVKIRDWYQKQGPGPAHDYSLYYKTI
EDLRDKILAATIDNSKIVLQIDNARLAADDFKTKYETEQALRMSVEADISGLRRVMDELTLARTDLEMQI
ESLKEELAYLKKNHEEEMKALGGQTGGQVSVEVDSAPGIDLTKILADMRDQYEVMAEKNRKDAETWFNSK
TEELNQEVAINTEQLQTSKTEITDLRRTLQGLEIELQSQLSMKAALEGTLADTENRYGAQLAQIQHLIGN
VEAQLTDLRADMERQNSDYKQLMDIKTRLEQEIATYRQLLEGQDS

>Turtle_K9L
MSRSTKTTITSSSVVGGGGGGRISSGGGGGGRSSSGGGGGHSSSSTRRITSGGSFSGRSYGGGGSRCDYG
GRISCGGYGGGISGGSCGGVIGGGSYGGGISGGSFGGGFGSSCGGFGGFDGGFGGGFGGGGFGGVGGFGG
AGGFGGDDTGILSSNEKITMQNLNDRLASYLNKVRALEEENANLENLIKEWYLRKGPIATLKDYSRYYEE
IEQLNNQIVNSSVEVNKVILNIDNTRMTIEDFKLKYETELGLRQNVEGDINGLRPLLDQLTLTRSDLELE
FESLKEELISLKKNHEEELKGLQPQTSGDVNVEVNATPGYDLTEILNDLRREYEHIIENNRKEVEKWYET
KISEVNHEVHSSGRDIESSTKQVTELRREYQNVEIELQSLLSTIQSLQSSLADTEGRYNIQLNQIQTIIV
SVEEELASIRCEIDNQNQEYRLLLGIKNQLEQEIAQYRHLLDEGNQEISITREGGGYSSGGGRVIGGGGS
GVRTGGSSITYGGSIGGVRGGSITGGGGSGDRTGGSSITYTGSTGRGSERGGSITGGGGSGGRTEIREGN
VHYGGGGTVRGGEICSGDGTGGRTCGVTGGERISGGSSSSSHSYSSSHGHSSSQPSKSGEIYAHSRKSFD

>Turtle_HAS1
MAGPQTVTLSTKGRSSGGSCAVGGGGGGRISSVSSGRYSSCGIGSGRGFSGRSYSGGASCGAGLSAGSFS
GGSYGGVLAGGHPGYGYGGCSSIGFSGGSTRCGYGGGFGGAVGGGFGGAVGGGFGGGGLCGDGVFVTCDE
KLTMQNLNDRLASYLNKVRCLEEENAALECKIKEWYAHHGHTGVPKDYSCYYKEIEDLQNQIVCATLDNN
KIILNIDNSRMAADDFRLKFETELALRQSVEADINGLRHVLDELTLCRSDLEAQLECLKEELCCLKKNHE
EEINCLRNQSTGDVSVEVNSCPGPDLKKILEEMRCQYETMIAQNRKEVEDWYECKIEEVNREVITSSQEV
ESCNNQVAELRRQLQCLEIDLQSHLSQRDNLEASLAETESRYNSHLCQLQQQITCVEQQLADLRTEIESQ
NHEYKVLLDVKCRLEQEIHTYRCLLEGGQRDIVGSEGGFGVAGGIGGGTVIKTSHCYSSSHSVPHVTSCH
PGDLKGHGRKICD

>Turtle_HAS2
XXXKDYSCYYKEIEDLQNQIVCATLDNNKIILNIDNSRMAADDFRLKFETELALRQSVEADINGLRQVLD
ELTLCRSDLEAQLECLKEELCCLKKNHEEEINCLRNQSTGDVSVEVNSCPGPDLKKILEEMRCQYETMIA
QNRKEVEDWYECKIEEVNREVITSSQEVESCNSQVAELRRQLQCLEIDLQSHLSQRDNLEASLAETESRY
NSHLCQLQQQITCVEQQLADLRAEIESQNHEYKVLLDVKCRLEQEIHTYRCLLEGGQRDIXXX

>Turtle_K14L1
MTTSVRQYSSSTSLKGLGGLGGLGGGSTRVSSVHLGGPYRAPSIHGGSGGFSVSSSRYVSGVGSSLGGGY
GGSYNSSFGGGYGGGLGGGYGGGLGGGYGGGLGGGLGGGFGGGYGGGFGGGFGGGDGILPAGEKETMQNL
NDRLATYLDKVRALEEANSDLEIKIKEWYKKQGPSPDRDYSPYYRTIEDLRSKILAATIDNANIVLQIDN
ARLAADDFRTKFETELALRMSVEADINGLRRVLDELTLARADLEMQIENLKEELAYLKKNHEEEMNILRS
QLGGEITVEMDAAPGVDLTKILADMREQYESLAEKNRKEAEQWFFTKTEELNREVAMNTEQLQSGKSEIT
ELRRTVQGLEIELQSQLSMKAALEGTLAETESRYGAQLAQLQGLITSVEEQLAELRCDMERQNHEYKILV
DVKTRLEQEIATYRRLLEGEDTHIASQYSAALSSQSGRDVISSTRQVRTIVEEVQDGKVVSSREQVQLST
R

>Turtle_K14L2
MTTSVRQYSSSTSLKGLGGLGGLGGGSTRVSSVHLGGPYRAPSIHGGSGGFSVSSSKYVSGVGSSLGGGY
GGSYSSSFGGGYGGGLGGGYGGGLGGGLGGGFGGGYGGGFGGGFGGGDGILPAGEKETMQNLNDRLANYL
DKVRALEEANSDLEIKIKEWYKKQGPGPDRDYSPYYRTIEDLRSKILAATVDNASLLLQIDNARLTADDF
RTKFETEQALRMSVEADINGLRRVLDELTLARADLEMQIENLKEELAYLKKNHEEEMNILRSQLGGDITV
EMDAAPGVDLTKILADMREQYESLAEKNRKEAEQWFFTQTEQLNREVAMNTEQLQSGKSEITELRRTVQG
LEIELQSQLSMKAALEGSLAETEARYGSQLAQLQGLITSVEEQLAELRCDMERQNHEYKILVDVKTRLEQ
EIATYRRLLEGEDTHIASQYASAKEVSTTTRQVRTIVEEVQDGKVISSREQVHHSPR

>Turtle_K117
MASFSRSGVTLGPRLSWVRVVGLQGGVPGGLGVCGGAGSTSSRLFSGSGDGIVGPGLGGGAREPGLLSMNE
KDTMQNLNDRLAAYLETVRSLEEANGQLERQIREVYAKRAQAGCQDLSGYFSTIAELRSQIQAASTSNAQ
LILQIDNATLAADDFRVKFESELAIRRGGESDVAGLRKVLDEVTLSRASLQEELGSLQEELAQLQKNHGE
EAAALQGRLGGSVSVEVDSAPGVDLVKTLAEIRDQYEDVIERNHREAAAWHKEQCETVAQEVATSAEAVQ
GARTKLTELRRVVQGLEVELQSLYSMKEALEGTLAETRAGSGAELEQLRECVARKEAELAQLRTDTQRQA
EEHQQLLDLKTRLEMEIATYRRLLEGDDVRSDAKSPVRQTPPEAVNSSPTSRRVKTVIEELLDGRVVSSR
TEEVEHPL

>Turtle_K18
MNYSRSTTYSSGVRGPNIMGRRYAPNSAASVYAGAGGSGSRISVARTTSLGSSFPGSSAGGSYSIALSGS
GVVQNEKETMQDLNERLASYLDKVRRLETDNQKLETQIREFMDKKGPSSRDWSHYFDIIEDLRKQILDSA
VDNARIVLQIDNARLAADDFRVKFESELAIRQSVESDINGLRKVLDDTNMSRLQLEGEIESLKEELIFMK
KNHQDEVDSLQAQITNSGLTVEVDAAQPQDLGKVMAEIRAQYDNLAQKNMADLDQQWNQMITQSTVEITQ
NTKEIEVARSSVSELRRTYQTLEIELESLRNLKASLEANLQDVEQRYAMQMEHLNGQLLRAEAELAQIRN
DIQRQSEDYQALLNIKDKLEAEIATYRRLLEGGEEFNLNDALVDTGSISQTTHKVVTTTKKMVDGKVVSD
TSESQLFRR

>Lizard_K24
MSLRISTASRRISPIAGGNGGIKLLGSGGSGMHGYGGGSLAALGSGCGGSVCGGFGGAGFGSGGGYGSGV
SFSSRGSGFGGGSGFGGGSGFGGGSGFGGEAGFGSASMSSANYGGNYRPSSAMVSSSGFGTGDSIFLAGA
EKETMQNLNNRLANYLEKVRSLEEANAELEVKIRHWYEKNGPGAPGVVRDYTKYHQIIEDLRNKIVNTTI

DNANVVLQIDNARLAADDFRLKFENELFLRQSVESDINGLRRVLDELTMNRSDLEAQLENLTEELAYLKK
NHEEEINMLKSNSTGDVTVEMNAAPGIDLTKLLNDMRAQYEDLAEQNRREAEEQFHKMSQPLQQQIYDDA
GAVSSARNELIELKRSFQSLEIELQSLLAKKASLEGTLTETEANYSSQLHQLQLQVSNLEEQLQQIRSET
ECQNSEYQQLLGIKTRLEMEIETYRRLLDGENFHSYDTKVHMREPSKTRVVKTIVEEVVDGKVVSSQVKS
VEERATK

>Lizard_K24L
MSLSLLTSGSSRQLSSYSVGGGGGGGSVRLSSGGGFGSGSKFVGGYSGGGYAGGSLAGGYGSGLGGGSGG
GFGGGFSSSSFAGGFGGGHGGGFGGGHGGGFGGGHGGGFGGGHGGGFGGGFGGGDSGLLSGDEKHTMQNL
NDRLANYLGKVHALEEANAELELKIKDWYAKCSIPDTSRDYTKYFNIIEDLRGKILRQTIENAGIVLQID
NARLAADDFKLKYENELFLHQNVENDINGLRRVLDDLTMHRSDLELQIEGLNEELAYLKKNHEEELKSFR
GASSGDVSVEMDAAPGVDLTKLLNSMRSQYEELAEKNRQEAEDQFNKQVGALRQEISANVDQLSTGKTEI
TDLRRTLQGLELELQAQLAMKQSLEGTLAETERNYCDQLAQIQSQIAGLEEQLLQLRADMENQNSDYQHL
LGIKTRLEQEIETYRRLLDGEGGFGSGVSYGSTSYGSTSYGSTGSGGSGTESKKTRKIKTVVEEIVDGRV
VSSQVKSVEEKQAT

>Lizard_K10L
MQLRKDVPKKEHTLTKSAYKSLRLSLLFKISLGCTFSFSCTHNFFLHFNIMSYFGRGSRQVSSSHSGGTS
AKLSGCGAYGLGGGSSAGSLCSRTTWSTGSGWGGIGGGTGGSFRIYSSSGSICIDGGLLFSGEKETMQNL
NDRLASYLAKVHALEKANSELELKIKNWYEKNAFQADGGFRDYSKYYASIDELRNKIVSASVNNSNIILQ
IDNARLAADDFRMKYENEVFFRQSIEADINGLHKVFDEVILSKSNLELQIESLIEELAFLKKNHDEEIRG
LRGPTSGNVSVEMNAVPGPDLTILLNKMREEYEALAEQNRQDAETWFVKKSNELNTTISMSAKETSTNKK
EIAELRRTLQGLEIDLKTQLALRQSLETTLAETEDGYCSQLLQMQGLINNTEAQVKQVRDDMESQNADYR
QLLDIKIHLENEIETYRHLIDSEKSEFVYRDTKTLESGTDLKLRGTEYGTRGLGSGRDSISSGTEAIDTN
SESKEKETKRTTVIKTIIEELDEHGRVTSSKVQSVEEKTVI

>Lizard_K12
MSLSVRAATGPLQFSSRSGYGGGSVRLSSTSSAGSFGGGFGGGPLLNFGGGGYGGGLGAGYGGGLGAGYG
GGLGGGYGGGGFSGGSGAGFGGGYGSGGGGESSLLSGTEKETMQNLNDRLAAYLDRVHSLEEANTELERK
IREWYEKNGPGTGALGAGRDYSKYYPVIEDLRNKIINATIENARIVLQIDNARLAADDFRLKFENEVALR
QSVEADINGLRRVLDELTLTRADLEMQIETLNEELAYLKKNHEEELIGFQSNATGQVSVEMDAAPGVDLT
KLLNEMRAQYESIAEQHRKEAEAWFNEKSGELKREISTHSEQLQYGKSELTDLKRTLQSLEIELQSQLAL
KKSLEDTLAETEAGYCTQLAQMQLQIGNVESQLFQVRADMERQNAEYQNLMDIKTRLEMEIETYRRLLDG
EIGSFESASVTSAKSVDSSRDPNKTRKIKTIVEEVVDGKVVSSKVKEVEEKI

>Lizard_K23
MSSSQGLYHFPAGSFRGAAATVLGCSFAPAEGYYRASSLHGGSSSTRIPMSFGRPIWLTSDGGAGWGGFG
GSHGNLFMGRNEKQTMQDLNDRLAAYLDKVHSLEEANTQLEGCIREWHERRSQGMKHDFTDYEQNIVDMH
ERIEAGRVTNAGIVLQIDNAKMATEDFRLKYESEKALRQSVQDDVENIRKELDNMTIIITDLEMEIEALR
EDHILKKKEHEKDMEASGAFKDVNVNVKVNTAPQEDLAKILAGIRADYEAIIQKNHQSLDAWFQKQIAND
SSEEKPNQEEQQSNQKEISELNRTLQSLEIDLQTHLNKKCSLENNLDETRAYYAFQLQSIQNLISQCEEE
LSEFRHDIKCQNNQYKVLLGIKTRLETEISTYRQLLDGKGMAGSSSTLKEIGSNNQNLKKIVQESVNGQV
VSTHTTDIKRQV

>Lizard_K36L1
MAAPSTHTCGSLKGPCRVSTVSRPNSAASATCRPGNIFPRRSSICLPSLGQPTPSLPNSCRSGGFMPSTC
LPPPSLGSTCVPPPCILPSARPLQCFPRGPEEGILNCNEKETMEFLNNRLGNYLERVRCLEQENAELEAK
IREWYEHENAYVCTDFKPFYCTIDELQQQICCTKADNAKLCLDIDNLKMASDDYCTKYDYELSLRQRDEC
DSSGLRKVLDNLTLCRADLEAQLESLTEEMMCLKKNHEEESSALRSQLGDRINVEVDAAPSCNLNKVLDE
MRCQYEADLEKNRREVEDWYCAQMEELNKEVISSGEQLQCCQTEIIETRRCVQTLEIELQAQQSMKSALE
TTLQETESRYCTQLAQLQCLISNVEAQLTEIRSDIERQNCDYKALLDEKARLDCEIAAYRNMLEGEDCKI
PNRPCDPEGSTTVRSTVCRQVCKPEPICVPICPPDCKPPGPMVGPKLTPSCANPSGPVFATCGPPPCAPP
PCGPRGPCGPRSPVCPQPCGPCPPGPRINTKICRM

>Lizard_K36L2
MASKYYAQTVSSGSVKGGGLTRIASVHSGGSCRVPSLAFGSRSVSSSSIKLGCGNYMPSICPPNASFAPF
LSGNNGWHDEGILNCNEKELMQCLNDRLANYLERVRCLEQDNANLECKIREWHECQAPYESTDFHSHYKI
IEELQHQILCAKTENARLVLEIDNARLAADDFRTKYETELALRQCVESDIHGLRKILDEMTLCRADLEAQ
LESHKEELLCLKKNHEEEANALRSKLGARVNVEVDAAPSCDLNKMLGEIRNQYECLAEKNRRDVETWFSS
KMEELNQQVLSSDEELQNCQSEIIELRHTMQALEIDLDAQQNMKNALECTLHETEARYSTQLAQLQCMIS
TIEGQLGDLRNDMERQSHEYKILLDVKTRLEAEISTYRRLLEGEDHKFPRVPSVPECPPPVSAPAPHITK
KIRTITEEIKDGKVISSREQVQHVAL

>Lizard_K15
MSTYYQSSSSSHGGGMGFGGGGGSSRLSSGGFGGSGGGRMSSSSVRYVSSGGGHGGGYGGSLGGGYGSGF
ISGGGVGLASGFGGGAGGGYGGGAGGGYGGGFGGGFGGGYGGGGFGSCFDFAAGGDGVLLAGNEKVTMQN
LNDRLATYLDKVRALEEANTELEVKIRDWHQKQAPKSPSRDYSQYYKIIEDLRDKILGTTIDNSRVILEI
DNARLAADDFRLKYENELHLRQSVEADINGLRRVLDELTMSKSDLEMQIEGLKEDLAYLNKNHEELMKEF
STQLTGKVSVEMDAAPGIDLTKILADMREQYEHLAEQNRKDAETWFFSKTEELNREVATNTEQIQTSKSE
ISELRRTIQGLEIELQSQLSMKAGLESNLAETEGRYCAQLAQIQNLITGVEEQLAELRNDMERQNQEYKM
LMDIKTRLEQEIATYRNLLEGQDSKLSTWSPKDASYGGGSTTVTYSGGSSGGGGGGAGSGSYGGGSGSIT
TSSTSSGSKVRSSNY

>Lizard_K19
MTSYSYRQSSTSVGGGLGGHSSFRAPSIHGGSGGRGISVSSARFVSSGAGGALGGGYGGGMSGGNYGAGF
GSGFGGGFGGGFGGGFGGGFGGGAACGAGNDGLLSGNEKITMQNLNDRLASYLDKVHALEESNADLEVKI
REWYQKQGPTSPARDYSHYYKTIEDLRDKILDATIDNNKIVLQIDNARLAADDFKTKFESEQALRMSVEG
DINGLRRVLDELTLARSDLELQIETLKEELAYLKKNHEEELGALSGHLGGQVSVEVDSAPGIDLTKILAE

MRDQYELLADKNRRDAEAWFTTKTEELSREVAVNTEQLQSSKTEITDLRRTLQGLEIELQSQLSMKAALE
GTLADTENRYSAQLCQIQNLISNIEAQLVEVRSDMERQNSDYKMLMDIKSRLEQEIQTYRQLLDGHDLHF
SGADSGIIDKSGDGKKH

>Lizard_K9L1
MSVKGGGSSGGSAGGSYGSGGGSHTASGGSSRRISSTGISSGGISGGGTSGGGISSGGISSGGLSSGGVS
GGGGSSSGGGVRITSSSSSSRGYSGRSYGGGGSRSGGFSSGSAGRISRGRVYGGSSYGGGSYGGGSYGGG
YGGGGYGGVSYGSGGGYSSGSFSGGSYGGGGYGGGYYPFRRGSFGMGGGDGGLLSNNEKSTMQNLNDRLA
AYMNKVKYLEDENAQLEVLIREWYQKHGDVGTVRDYTHHYQEIDQLFNELVNETDDYNKILLNIDNTRMT
AEDFQIKYQTEAGLRQNVEADINGLRPMLDQLTLARSDLEAQFESAKEELITLKKNHQEALRELQSQRGH
GDINVEVNAAPGPDVKQRLDQLRAEYEAIIDNNRREVEQWFESKMEEVRQQVSSSGLEISSGNQQVTDLR
RQYQTLEIELQSALSMIQSLQSNLEDTERRYNLQLQQIQSMIQPVEGELASLRGEIESQSQEYQSLLGIK
TRLEQEIAQYRQLLDEGQQEIGSGGGGGYGGGYGGGYGGGRRGSIGRIGGGGMSSGGMSSGGMSGGGMSS
GTISGGGMSGGMSGGGMSSSGISGGGISGGSMSGGGLSSGSMSGGGMSGGSISSGGKMSGSMSSSGMSSG
GVIGGGSIGSGGGGSQIGGGGGSSRHGRKSFD

>Lizard_K9L2
MSSGYNFRQSQSSSGGGSGCSCVGGGSSGGGGSSGSGHSGSGSGGGGFSSGSSIRHTSTGRNSQGFSGES
HDAGRSRRRSGAGIGHSASGGFGGGSVHSGGLGGGSVHSGGFGGGSVHGGALGSGSVHSGGFGGGSVHSG
GLGGDSVHSGGFGGGSFSGGVGRGSGYGGGSMRRGSFSGGFGSSSGFGGGYGGGSFGGGFGPAGGFGGGS
LGGGFGGAGFGGDDTGFLSNNEKSTMQNLNDRFASYLNKVKSLEAENNQLETLIREWYQKHGQTSEPKDY
SSYYTEIDQLVNELISASLESNKILLDVDNARMAAEDFKLKYETEYGLRQNVDADLQGLRPLLDKLTMDK
SDLEMQYESLHEELVHLKKNHDEVMKSQHHQSGGDVNVEVNSSPGQDLTKTLNDLRQEYEEIIMKNRKEV
QQWFEAKMEEARQQQSPESQEGGSGRHQITELTREYQTLEIELQTQLSTIQALQNNLNNTEGGYNMQLQQ
LASQIEPVEAELAGIKGEIQNQTQEYQTLLGIKTYLEQEINQYRQLLEEGKQLAANVQGGGSGGTGSYGG
SSGGGRHSVGGISGGSSGGGGSGGGHSYGGISRGGSSGGGGESGSGHSYGGISEGGSSGGGRSTSGGRHS
TSGISHGGSSIGISQGGSSRGRESGGVGGHSSGRISPGGSSGGGGSGGRGGQSHSGISHGGSSGGGGSGG
RGGQSHSGISHGGSAGGGSAGRPSSGGVSQGGSSGRRGSESGRQSPSGGHSGGGRGSVGGGGHSSGGISH
GRGSGGGSHHLSSSSSQSRPSLTHSHSSSSSQSQSGSHGWHVEQADECQAYAQKGMEE

>Lizard_HAS1
MFTLCLSSKHTYTMMYINGSHSNGATSYNVLGASSGASRRCVFPDVGGSNCGGSPSYCGGTAYEGNQDME
RGIYGGLDGGPLGNGYICTPSSGFGFSNICFKHLGGGFGIWIQAPLCSCDKKMTMQNLNDRLASYLDNVR
YLEEENAQLECKIRDWYDQQGSPNDPKDYNYYYEQTEDLKKQLICATVDCRKLILDIHNNKLTADDFRMR
YEAERVFRQDKETDIDSLRQVLDQLTLGRSDLEAQLESFQEELCCLKKNHEEKVKLLTKQAQGNLNVEVN
SNPSSELQKALDNLRRRYEAIIEKNVKEIETWYESMTEAINMSKELKDDNNQVVDLKGQLQTLESNLQTQ
LSLRDTLQDSLAETQCLYNNSLAEIQNQICYMEQQLVELRVKTECLDQEYIRLSDAKCLLEHEIETYCCL
LGEEQHD

>Lizard_HAS2
MSFSSKHTTTTMYTKGRSSSGSCVTGGSGGRANSGRCSSPARCGMSARSYCGGMSYGGSGIDSSFAGGAL
GCGYGGGYGSSRFGHLGGGFGICNEAPLLSCDEKMTMQNLNDRLASYLDKVRCLEEENGQLECKIKEWYD
RQGPVNESKDYAAYYQQIEDLKNQIVCATVENSKLVLDIDNNRMTADDFRMKYETELALCQNVEADINGL
RQVLDQLTLCRSDLEAQLESLREELCCLKKNHEEESKHLKKQSSGDVSVEVNTCPGPDLKKVLDELRCQY
ENMIENNRREVEKWYESKMEEVNLEVCNSSKEAEDCNNQIVDLKRQLQTLEIDLQAQLSLRDTLQDSLAE
TECRYNGHLAEIQNQISCVEQQLGELRAEMECQNREYRELLDVKCRLEQEIQTYRCLLEGGQHDIATGHS
SGSSGGGISRQSSGSTTRSSPSRPSHPDSKGHSR

>Lizard_HAS3
MASYARHIPVNVRRNRVNLGSDGKSRISSAFSTHCTSSGLDRSSFPCQSHIVDRSPFDFANGGQGTFSSR
SCSSVLMSGGGYGRRMSTGSCLNGSHGGYPLLGYGDGQVFTGGLGGVPSTCGIIFSSTDEKVLMQNLNDR
LGNYIDKVRCLEKENAALESQISDFCLKHGLTGEMKDHSHFYQQIEELKKQVFVANLENNKILLKIDNNH
MDLEDLRKKYETEYVIRENVEADVNGLRPVLKELTGYKADLKAELNSLKEEICDLKKTHEKDLNCRKKRS
HGSNKEVSIGPGTDLKQMLKEMRQKYEAKIESNRKEVVQRYEDKLKEMNLNVNNSSQDIEDGNHKVLDLK
CKLPALEIDLQAQYNMRDTLKVCLAEIECHFQTQLGEMQEQISNMEHQLAELHLEMETQDQEYKGLVDVK
KRLEQEIQTYSSLLKEGQNDPSSTIDMQPERSPLA

>Lizard_HAS4
MSSGTRHTVATPARSSSGSSTLGGGRAGVGSVSSGRYAASGISNGRSNFSVRSNTGAVSPCTHGPGRGHF
SSGSCSGMFVAGGHVAASGYGDGSHVTNPAARNTVVGGAGAGHPTVVGGTGHAIPGDMAAGAHVGMPTGV
VGGMVMGAHGGMPAGVVGSMGSVAGSPHPSMVAGSPHPSMVGGNPHPGMVAGNPHPGMVAGHCGMVGGLP
AGMVAGMGGMMGGGHGGMMSGVTSVMTGPILVSAVGGEPIACASVFSSFDGKMTMQNLNDRLASYLDKVR
CLEEENADLESRIREFYAKQGPLSEPKDYSHYHQQIEDLKNQLICTSVENNKLLLCIDNSKLTADDFRTK
FETECCLRQNVEADINGLHQILDQLTACRADLDVQCDNLQDELNCLKKNHAEEVACLKKQSTGDVNVEVN
TCPGPDLKKILEEMRCKYETMIEGNRKEVEAWYESKIEEVNRDVCNSSQEIEESNNKVTELRRQLQALQI
DYEAQCSLRDTLEASLGETELRYNGHLGDLQERISCLEQQLAELRSEMECQNHEYTELLDVKSRLEQEIG
TYRGLLEGGQNDSNSHNGGGGNTSRVSSASNGRSSESRSTHTYGTHSAVHTHHGGHLHH

>Lizard_K14L1
MSTSFRQYTSSSSVKGGSFGGGSSRLSHLGRGYVAPGIHSGLGGISVSSSRYVSGVGSALGGGYGGSSYC
SSYGGGFGGGFGGGFGGGSFVAGGGDVLLAGGEKETMQNLNDRLASYLDKVRALEEANTDLEVKIKEWYK
KQGPSPERDYSPYFRTIEELRSKILAATVDNASIVLQIDNARLAADDFRNKFETEHALRVNVEADINGLR
RVLDELTLARADLEMQIENFKEELVYLRKNHEEEMSIFRSQSGGEISVEMDAAPGVDLTKILSEMREQYE
SLAEKNRKDAEQWFFSKTEELTREVAINTEQLQSGKTEITELRRTYQNLEIELNAQLSMKAALESSLADT
EARYGAQLSQIQVLITNVEEQLADLRCEMERQNHEYKMLLDVKTRLEQEIATYRRLLEGEEAHISSQYNA
AISGREVSKTSRQVRMIVEEVENGKVVSSREQLQQSAY

>Lizard_K14L2

MTTTTYRQYTSSSSAKGSSFGGGSSRPSSVHVGGGYQPSSIHGGLGGISVSSSRYVSGVGSSLGGGYGGS
SYCSSYGGGFGGGFGGGFGGGSFVAGGSDVLLAGGEKETMQNLNDRLATYLDKVRALEHANTELEVKIRE
WYQKRGPGPQRDYSSYFRTIEDLRSKILAASVQNATILLQLDNAKLTADDFRTKYETEHALRTSVEADIH
GLRKVMDELTLTRSDLEMQLENLKEELAYLHKNHEEELKVLRSQMGGEITVEMDAAPGVDLTKILAEMRE
QYEHLAEKNRKDAEQWFFSKTEELNQEVAINTEQLQSGKSEVIELRRTLQGLEIELQTQLSMKSSLEGTL
AETESRYGLQLNQLQALITSVEEQLAELRCDMERQNSEYKILLDVKTRLEQEISTYRRLLEGEEAHISSQ
YSSGPTTTRQVRTIVEEVQDGKVISSREQVHVSGR

>Lizard_K117
MSTTFSSRSLSFSGSVRGPRSGSTLSIARPQTASNIYGVMNSLPPSAKDTMARRYGPETDILDINQKGTM
QNLNDRLAAYLGRVHSLEESNAQLEQNIREYYAKRASAGGPDLSGYFNTLSELKAKIEQETLNNAGLLLR
IDNAKLAADDFKVKLESEVAIRLSLEGDVNGLRKVLDDVNASRASLQVQVDNLQEELVYLKRNHEEEVAS
LQGRLGGTVNVEVDSMPGVDLQKVLAEIRDQYEDVMEKNRQEAEALHKAQCDAINQQVADSSEALQAAQL
KITELRRLAQALEIELQSLRSTKIALEGTLVETESRYGMELSRLRELISARESELFQLRSEAQRQAEDYK
RLMDIKNKLEQEIATYRRLLEGLDTEPPSPPEHTTSRRVKTLIEELVDGQVVSSRVEEVEQPL

>Lizard_K18
MSFSRSSTVYTSSSSSRPGGVSSASVQLSQGPSSASVYGGAGGSGSRISVSRVRRLGSSQSFGSGGGFGG
GSGYGGYSGSFSLSGSGVVHNEKETMQDLNDRLASYLEKVRSLEADNRKLEIQIREHLDKKGPSARDWSH
YFELIEDLRNQIFDQTIDNSRIVLQIDNARLAADDFRVKFEAELAIRQSVENDINGLRRVIDDTNMMRLQ
LESEIEALKEELVFMKKNHQDEVNNLQAQIANSGLTVEVDAPKTQDLGKIMAEIRAQYDVLAQKNLEDLD
KYWSQQITESTVVITEHSKDIEKARANITDLRRSVQTHEIELESARNLRASLEANLHEVEMRYGLQMEHL
NGIILRTEADLAQLRNDVQRQAEEYQILLNIKDKLEAEITTYRHLLEGGEELSLKDALLESTMQTTQKIH
STKIVDGKVVSETSETRVTKK

>Snake_K24
MSLRMSTASRKISSYEGGAKMSGIGGGSLYGFGGGSMAGLGSSYGGAMCSGFGGSGFGGGIGFGSASMAG
GGYGANYRSSSSVSMSGAYGAGDSVFLAGAEKETMQNLNSRLANYLEKVRSLEEANAELERKIRHWYEKN
APGAPGVTRDYSKYHRIIDELRNQILSMTVENANVVLQIDNARLAADDFRMKFENELFLRQSVEGDINGL
RRVLDDLTMNRSDLEAQLESFTEELAYLKKNHEEEMTLLRSSSTGDVTVEMNAAPGIDLTKLLNDMRAQY
EDLAEQNRREAEEQFIRMSQPLQQQIHDDAGAASSARNELIELKRSLQSLEIELQSLLAKKASLEGTLSE
TEASYSTQLSQLQMQVSSLEEQLQQIRAETECQNSEYQQLLGIKTRLEMEIETYRRLLDGESIRAAYETK
VQIREPTKTRVVKTIVEELVDGKVISSQVKSVEEKTSK

>Snake_K24L
MALSVLTSGSSRQISSYSIGGGGGGSVRLSSGVGFSGGSKFAGGYSGGGFGGGSLAGGYSGGGLGGGSLA
GGYGAGMGGGFASSSFAGGFGGGFGMGGGDGGLLTGDEKQTMQNLNDRLANYLGKVHALEEANSELELRI
KEWYGKFSATDTDRDYSKYFKIIEDLRNQICRYTIENAGIVLQVDNARLAADDFKLKYENELFLHQSVEN
DINGLRRVLDDLALNKADLELQVESLTEELVYLKKNHEEELMSFRGVASGDVTVEMDAAPGVDLTKLLND
MRSQYEELADKNRREAEEQFNKQVGALRQQISANVDQLSSGKSEITELRRTMQGLELELQAQMAMKQSLE
GTLAETERNYCDQLGQIQVQISSFEEQLLQIRSDMENQNAEYQQLLGIKTRLEKEIETYHRLLDGEGGGY
STTVYSSGPPVKESTKTRMVKTIVEEMVDGRVVSSQVKSVEEKPSK

>Snake_K10L1
MSYFACGSRQTTSSRSSGAPLKLSSCSIYGSGGGSSTRSFSSGVVWGSGSGWGKTGGSGTGSCFHIYNSS
SAGGDVGLLAGGEKETMQNLNDRLASYLAKVHALEEANGDLEQKIRNWYQKSATHINEGLYNDSKYNSSI
EDLRNKIITASIRNAGTILQIDNARLAADDFRMKYENELFLHQNVEADINGLKKLLDELSMSKSDLEIQI
KSLTEELTFLKKNHEEEMKNWQGTVAGDINVEINAAPGVDLTAVLNKMRTEYEALAEQNRKDAETWFIEK
SKELNTTINVSAKETTTNKKQIADLRHILQSLEIDMKTQLSLRQSLERTLAETEGQYCAQLLKIQGKISS
VENQVQQIREDMECQNADYRHLLDIKIRLENEIETYRRLIDGEASESGQRSSKDLGSQNLGSEKGSGLRA
SGSDVRGTKTGTETSSRKSETRNIESTTEDKEIKKTRVIKTIIEELDEYGRVTSSKVQSVEETQE

>Snake_K10L2
XXXMQNLNDRLATYLGNVQALEEANSELEGKIAEWYEMFGAGNQAGQQQDYSEYYAIIDDLRNQIIATSI
DNGTLILQIDNARLAGADFKLKYDNEVAIHQSVEMDANGLRKALEEIEITSSDLKVQINSFTEELEQMKK
NHEEDMVSMNDSTAAGDINVEMNVTPGVDLTTLLNQMRGEYEALAEQNREDMEAWFHEQSQELNRQINTS
VEETSTNKSEITEMKRALQGLEIELQSQLSLKQSLEATLAETEGQYCAQLSQIQEVIGSVEGQVQQIRDD
MECQNREYEQLLDVKIRLENEIDTYRSLLDGSESADKTSGGRDMRQLSEVKSKGSKSRDQSFFSSGSKAL
GSSDSRAKESPSGPSGSQSRSGESADQRFKRQEHATAQSQSKGSGDSRQTDKLGSGNNTRDPQKTRVIRT
IVEDRIGDQVVSSRVQSVEEKHIK

>Snake_K20
XXXMQNLNDRLAAYLEKVRSLETSNSVFEKQIREWYEKSSPITKQDYSAYFKVIRELQDKITEAREDNTR
IILQIDNSKLAADDFRVKYETELGLRLSVEYDITGLQGLIDDLTLVRTDLEQQIESLKEELAYLRKQHDE
ETARLRGQLSGNVSVEVDAAPGIDLAQMMENMRQQYEILAEKNRQEAKDRFDKLIEQLNEEVTTTTYQLE
THRSEITDRRNTLQSLEIELRSQLSWKTERENALAEIEAHYRTILTQIQMAIGNIEAQLMQLRSDMEQQS
MEYSILLDIKVKLEAEISTYRRLLEGEDWRMIASDLETSKEAEKEKNKTLRIKTVVEEMVDGKVVSSHVD
EREEKM

>Snake_K23
MNSSHGFYHFSSGCLGGASGCILQPTGDCYRASSFHGASSSLPMPMSFGSPLWLASDAGTSWGAFSGSQG
NLFMGGNEKRTMQELNDRLASYLEKVRSLEEANTQLEGCIREWHQKRSHGTQRDFKDYEQNISDMHDRIE
TGRVTNAGLVLQIDNAKMATEDFSLKYEAEKALRQNVQNDVENIRKELDNMTIIITDLEMEIEALREDHI
LKKKEHEANMQAHGSSKDFKVNVKVDKTPQEDLSKILAGIRADYEAIIEKNRQSLDSWFQQQIGNESSAG
QHNQEELQNSQKEISELNRTLQTLEIDFQTELNKKFALENNFGETKGRYAFQLQSIQNLIKKCEEELSKF
RHDIKCQNNQYKVLFGIKTRLEREISTYRQLLDGDAEGKTSTSLREHRSSNQNLKKIVQETVDGEVVSTH
TTEIKRQA

>Snake_K15
MSTHYYQSSSSSYGGGGGGGSGRVASVRLGPSSIHGGAGGRVSASSARFVSSGSGYGGGYGYGSGLGVGC
GIGYVAGGGVGYGSCFGGGVGGGYGGGVGGGYGGGFGGGYGGGLDFVVGGGGGDGGLLTGNEKITMQNLN
DRLANYLDKVRALEEANTELEIKIRDWHQKQAPTSQAKDYSNYYKIISDLRNKIGDCTCDNSKVVLEIDN
ARLAADDFRLKYENELHLRQGVESDINGLRRVMDELTLFKSDMEMQIEEKKEDLAYLKKNHEELMKEYST
QLTGQVNVEMDAAPGVDLTKILADMREQYEQMAERNRRDAEAWFFTKTEELNREVATHTEQLQTSKTEIS
ELRRTIQSLEIELQSHLSMKAGLESNLADTEGRYCAQLAQIQNLITGVEEQLAELRNDMERQNQEYRMLM
DIKTRLEQEIATYRNLLEGQDSKLPGWSPKDASYGGGSTTITYSSGGSSGGGGAISGGGYSSGGGSSVTT
SKVRSGNF

>Snake_K19
MNTYSFRQSSSTIGGGGFGGSSSFRTPSIHGGSGGYGISVSSARLASPGFGGGLASGYGGGSFSYGSGCF
GGAGGAGGAGGAFGGNLNVSEGLLSGNEKVTMQNLNDRLANYLDKVRALEEANTELEVKIREWYQKQGPT
ASRDFSLYFKTIEDLREKIKDATIGNNSIILQIDNARLAADDFRTKFETEQALRISVETDINGLRRVLDE
LTLSRADLEMQIENLKEELAYLKKNHEEEMSTLSGQLGGQVSVEVDSAPGIDLTKILADMRDQYELLAEK
NRRDAEAWFTSKTEELNKEVAVNTEQLQTSKTEITDLRRTLQSLEIELQSQLSMKSALEGTLADTEARYG
NRLCQIQGLISNIEAQLVEVRSDMERQNNDYKMLMDIKTRLEQEIETYRQLLDGQDFNFSDKAVDGKKP

>Snake_K9L
MSCGTKGGRVSGGGSIKITSSGSSRGLSGKSYGGSRSGYGGGGFSSGSCGRISRGRVSYGGSCYGGGSYG
GGSYGGYGGSGYGGVCYGGGSYGGGSYGSGSYGGGTCYPFSSGSFGGGDGGLLSSNEKATMQNLNDRLAN
YLDQVKRLEDENCQLEIWIKEWYQKHGDIGPDKDYSCYYQEIDQLYNELIAETDDYNKIILNIDNTRMTA
EDFKLKYNTEAGLCKNVEADINGLRPLLDQLTLAKSDLEMQFESLREELICLKKNHEETLRGLRGQRGHG
DVSVEVNAAPGQDLKQRLDQLRCEYENIIEQNRKEVEVWYETKMEEVRQQVSTSDQEITSSNHQLSELRR
EYQTLEIELQSQISMIQSLQTNLEDTERRYNMQLQQIQAMIEPVEGELASIRCEIESQNQEYQLLLGIKT
RLEQEICQYRRLLEEGQQEIGSGGFGGGYGGGRRGGGSISGGGVCVGGGVSGGSLGGGGVCVGGGVSGGS
ISGGGLSGGGHIGGGSSSRPQSYPTSSHSQACKTGHGRKSCD

>Snake_HAS1
MSCRSKQTMMYTKGRSSSSSCGGIRVVSGISGRGPSSVVSSVGGGVSGRTFSSGVSYGGASCTDGGFGGS
YVYGSGGSFRFGQIGGGFGISNETTLISCDEKMTMQNLNDRLACYLEKVRNLEEENADLEARIRQWYDRQ
GPPSELKDYSSYYQQIEELKNQIVCATVDNSKLILDIDNNRMTADDFRLKYETELALCQNVEADINGLRQ
VLDQLTLCRSDLEAQLESLREELCCIKKNHEEEIKSLKSQTCGDVSVEVNSSPGPDLKKVLEEMRCQYET
MIENNRKEVEQWYERKMEEVNQEVCISSKEVEDCNNQVVDLKRQLQTLEIDLQAQLCLRESLQDSLGETE
CRYNTHLAEIQNQISLVEQQLAELRAEMECQNREYRELLDVKCRLEQEIQTYRSLLEEGQQDISSPSSSP
SGGGLSKHSGGVRVLSHSYSSSSPGQASSSDTKGYSR

>Snake_HAS2
MSCGPRHTVLTAGRNSSGSCNLGVGVSSVGSGRYTSCGLGNERASFSGRSHLGGRSHFSTGSCSGAFVGG
GHAGTNGNFVATSHMGTSAARNGMVGGAGGGHVGMVGGIPGGVVGGAHSGLVGAVPTAVVNGMGGVVGAP
HATMVGAPCGVVGAVPAGMVGGMGHPGVVGGMGGVVPNPGAVAGLIGEPVCGGIFSNLDGKMTMQNLNDR
LASYLDKVRCLEEENAELECRIREFYAKQGPLSEPKDYSHYHQQIEDLKNQLICASVENNKLLLCIDNSK
LTADDFRSKYETECCLRQNVEADINGLHQILDQLTACRSDLDIQCENLQDELCCLRKNHEEEVACLKNQA
SGDVSVEVNACPGPDLKKILEEMRCKYESMIEGNRKEVETWYESKIEEVNRDVCTSSQEIEESNNKVTEL
RRQLQALEIEYDAQCSLRDTLEGSLGETELRYNSHLAELQERISCLEQQLAELRSEMECQNHDYTELLDV
KSRLEKEIATYRGLLEGGQHDIVGGGGVSRNSSTAGARSSEARTSHTYVTHSCHGAHLHH

>Snake_K14L1
MTTSIKQYTSSSSSKKGASCSFGGGSSRLSSVPLGGGCRTSSIYGGAGGISVFSSRYVSGVGNALGGGYG
GSSYCSSYGGGFGSGFGGGFGGGFGGGSGGGSVVSSDVLLTGGEKQTMQNLNDRLANYLDKVRALEEANT
SLEIKINEWYQKQGPAPDRDYSSYFRKIEDLKNQILAASIDNASVLLQIDNARLAADDFRTKFETEQALR
LNVESDINGLRRVLDELTLSRSDLEMQIENLKEELAYLKKNHEEEMNAFRKQVSGEITVEMDATPGVDLT
KILTDMREQYETLAEKNRKDAEQWFFTKTEDLNREVATNTVQLQTSKTEIVELRRTIQSLEIDLQAQLSM
KSALEGTLVETETRYGTQLGQIQGMITNVEEELAALRCDMERQNHEYKILLDVKTRLEQEIATYRRLLEG
EEAHISSQYNATKSGKEVSKTTRQVRMVVEELEDGKVVSSREQIQKNSY

>Snake_K14L2
MSTIIRHYTSTSSSSMKGPVGFSGENLVHFGRGCHAPSSHGGAGGISVTNARCVSGIGSTLGGTYGGSSY
YSTFAGGHGGGLSGSLGGGLTGGFVGGSGSDVLLAGNEKETMQNLNDRLAAYLDKVRALEESNASLEVRI
KEWYQKQAPGPDRDYSYYFRIIEDLRSKIVSATVENADVLLQIDNAKLTADDFKNKFEIEQALRMSIESD
INGLRNVLDDLTSTRIHLEKNLELLTEELLHLKKNHKEEMGLLQSQMGGEITVEMDAAPGVDLTKILADM
REQYENLAEKNRRDAEQWFFSKTEELNREVAINTEQLQSGQSEVIELRRTLQGLEIDLQAQLSTKAALEG
TLADIESRYSLQLAHIQGLIINVEEELATLRCDMERQNHEYKILLDVKTRLEQEIAMYRHLLEGEEA

>Snake_K117
MPTTFSSHSMSSGSIQGPRGRRLLALGRPQAVPSIHRVIRDKTRLSYGDGQGFMDTNILDMNEKGTMKNL
NDRLAAYLDKVLSLEKSNMQLEQNIQEFYTKQTSTGTHDMSAYFNAMIQMKSEIQKETLKNAGLLLQIDN
ARLAADDFRVKLESEVAMRLSLEGDLGGLRKALEELNASRASLQVQADNLQEELACLKRNHKEEVASLQG
HLGGTVNVEVDSMPGTDLQKVLGEVRDQYEGVMEKNRQEAEALHQSQCDALNQEVSVSTEALQSAQMKMV
ELKRLAQALEIELQSLWSMKEALEGTLAEVESHYGLELSQLQNLVAAREAELLQLKSDAQNQAEDYKRLM
DIKNRLEQEIATYRCLLEGSESEPLTTPEPSVSRRVKTVIEELVDGKVVSSRMEEVEHQL

>Snake_K18
MSFTKSTVLSAPPISAPPILSPLKFGIGGSAASVYAGAGGSGSRISVSRVSSLSSNYGGGYGGGSYGGGS
YGGSSGGGYGGMSSGLYLSGSGVGQNEKETMQDLNDRLANYLDKVRSLEADNRKLEIQIREYMEKKGPSA
RDWGRHFQIIDGLKNQIYDQSVDNARVVLQIDNARLAADDFRVKYEAELAIRQSVESDIFGLRKVIDDTN
MSRLQLESEIEALKEELIFMKKNHQDEVTTLQAQIANSGLTVEVDAPKSQDLAKIMAEIRAQYDALAQKN

LEDLDKYWSQQISESTTVINQNSKEIETARVTMTDLRRTVQTLEIELESMRNVRASLEGNLLEVESRYAM
QIEHLNGILLRTEAELSQLRNDVQRQADDYQALLNIKDKLEAEIATYRHLLEGGEDLNLKDALLESTMQV
SQKIHSTKIVDGKMVSETSETKTLKR

>Xenopus1_K18
MSYSRSVYSSSSVVGGSPYRSLSSAPRFIPSSSAASVHAGAGGSGARISVSRVSSVGSGFGGGYSGVSNV
NLIGGGQNEKETMQDLNDRLASYLERVRSLEAANKKLEVQIRQHTEKKGPSKDWSPYYKTIEDLRKQVFD
STLENSQLVLQIDNARLAADDFRVKYEAEMAIRMSVEGDITGLRKLIDDTNVSRMNLENEIESLKEELIF
LKKNHQDDVTELQAQVARSAVTVEVDAPKSQDLGKIMTELRAQYDGLAQKNRDDVEKWYQSKVDEHTVQV
NLDTEALHSAKSSVTDLRRTVQSLEIELESLRNQKASLEGTLHDTEARYAMELEMLGGTAMAMEAELVQV
RSDCQRQQQEYQALLNTKMKLEAEIHTYRRLLEGDGSFDLQDAVPTVTTQTVKKVITTTQRIVDGKVVSE
SNDTEVLKS

>Xenopus1_02
MSCKKSCGSSRISVGCGGYGTSVCSFLGNSGGVCGTPAYCGSVSGGAAGGLGGEFGNYAAGWGTGAAYGF
GVGPEGLLAGGEKETMQNLNDRLAAYLNKVRALEEANSDLECKIREWYEKHRNACMPDRDYSKYYHIIED
LKKQIQCASVENARVILQIDNARLAADDFKLKYENELCLRHGVEADINGLRKVLDELNLAKCDLESQIES
LTDEIACLKKNHEEEMKGYQGATGHLSVEMKAAPGNDLTKHLNDMRAEYEELAEKNRKEAEARFNEASRA
LKQEISCGAEQIQCSKNEITDLKRNLQALEIDLQAALAMKKTLECSLAETEGNYCVQLSKIQAKISALEQ
QLCEVRTDMERQSIEYEQLLDIKTRLEMEIETYRRLLDGESCIKSYPVPTPKEPCKTRVIKTIVEDIIDG
KIVSHQVTERKE

>Xenopus1_03
MSYRSFKQSTKSVQHSSGGYGGSQVGGGYSCGVGAGAGYGGDFGYVGGAGGYAGGFGADGFGVGGGYGGA
FGGQVGVFSINEKQTMQNLNDRLASYLDKVHELEGKNAELERKIKDWYEKHGPAPPEAPRDYSKYFKEIE
ALKAQIIAASKQNAVILLQCDNARLAADDFKQKFENEQAIRYSVEADINGLRRVMDDLTLSRAELEGQLE
SLTEELAYLKKNHDDEVRSIKVTEAGKVSVEMNAAPSNDLTKRLNDMRAQYEDLARNNRQEAENNFNRIS
ADLKNQMHHVQITMDESRTEVTNLKKTLQSLQIELQSQLAQKNSLEILLAETEARFCMKLSHLQETIACV
EQRLEQLRAESECQRTEYQQLLEMKTKLEGEIATYRSLLDKLG

>Xenopus1_04
MSQYSFKQSTKSVQYSSGSNAGGGYQCGVGGGFGGDGGFGGGAGYGAGFGAGGYEGGAGGFGGGGYGGGA
GFGGGAGFGGGAGFGGGAGFGGGAGFGGGAGFGGGAGFGGGPSGGLHMVSEKETMQNLNDRLASYLDKVN
ALEAANAELERKIKEWYDKQKPSTTTGEGAKDYSKYYKDIEDLKAKIIAASKENAALLLQCDNARLAADD
FKQKYESELHLRQTVEADINGLRRVMDELNLAKTDLDSQFQGLTEELASLKKNHEDEVKGVQVTEAGKVK
VEMNAAPSNDLTKRLNDMRAQYEDLAKKNRQELENKFNAATGPVKEKIKEDVKEDTTVKTQISDLKRSLQ
ALEIELQSLLAMKKSLELQLAETEGRFCMKLSVLQENIASIEQHLEQLRAESECQKDEYDQLLEMKTKLE
NEIKTYQSLLDKLGAIQVTQGQGQGQGQGQGQAQDQSRGQAPQKH

>Xenopus1_05
XXXMQNLNDRLANYLDKVNALEAANAELERKIKEWYDKQPTTSDGGKDYSKYYSLIDELNLKITNATKDNAGI
TLQADNARLAADDFNNKYENERALYQMMDNDINGLRRVVDDFTLSKFDLESQFEGLFEEMVFIKKKHDDE
IKGTQNTKLSQVSVEMKAQPGTDLTKILNDMRAQHEALAEKNRREAEDRYNTMTAELRQQISAGAGQMQA
SKTEISERKRTLQALETDLHSKLSMKQSLELMLAETEGIYCMKLSRIQVTISSIEEHLAQLKADSESQHS
QYQQLLGIKTRLEQEIETYRRLLDGEGXXX

>Xenopus1_06
MSYSSVKGGGSGKGGYSIESGFNSIRCQLCAARGCNWCCTNCPSVSNVFAGQGGGLGGGFGRGFGGGSGG
GFGARYGGGFGGGSGSGFGAGYGGGFGGGSGGGFGGGAKVAYEVTGTGGQYGSSVFGNEKETMQNLNDRL
ANYLDKVNALEAANAELERKIKEWYDKQPTTSDGGKDYNKYYSLINELNQKILNAALDNAELALHCENAK
LAAEDFRLKYESEWTLSQAVVSDINGLRKLMDDLTLSKSSLESQIESLSEELAYLNKTHDEEIKGTQNTK
LSQVSVEMKAQPGTDLTKILNDMRAQHEALAQKNRREAEDRYNKMSAELRQQISAGVGQVQTSKTETSEL
KKTLQALEIELQAQIAKKQSLELMLAETEGSYCMKLSRIQVTISSIEEHLAQLKGDSECQRSKYEQLLGI
KIRLEQEIEMYRKLLEGGGSNQTSDVGQRRGGGSGTSDTTVRTITVKKIVENVVNGKVVSKEEVIERL

>Xenopus1_07
MALAHGAYDVTGTGGQYDGGFSGNKKETMQNLNDRLANYVDKVRVLEAANAELEWKINEWYDKQRSTSDG
GKDYSKYATLIDDLKLKILSTTKDNRGIPLQSDNARLAIDNFRLKYENEQILHHSMEAAINTLHRVIDDL
TLSKSKLQSQLESLTEELAYLKKNHDEETKGAPKATRGQVSVELKATPGTDLTKILNDMRAQHEALAQKN
RREAEDRYEKLSADLKQRISAGAGRVQTGKSEISELQRTLRALEIELQSQLAMKQSLEVTVGKTEGSYCR
KLSRIQVTISSIEEHLAQLKADSESQHSQYQQLLGIKTRLEQEIETYRRLLDGEGXXX

>Xenopus1_08
MTSIRQSRSMGGGGSNMSVRKSVQTFQRVGSAGGGGGFAGGYGGGMAGGAGGMVMNGVGYGDFEGGYGVE
CNEGFGAGGFGGGFGGGAGGVYGGGAGGGFGGGAGGGFGGGAGGGFGGGAGGGFGGGAGGGFGGGFGGGF
GGRGPGGFEGLLATNEKHTMQNLNDRLANYLDKVKALETDNNDLEKKIREWYEKLRPESGGVGTVDYSKY
LPIIEDLRKKIMESTLENAKILLQTDNARLAADDFRLKYENELALRQSVEADINGLRRVLDELTLCKADL
ELQIESLTEELAYLKKNHKEELDALRGGPAGQLTVEMNAAPAVDLTKLLNDMREQYETLAEKNRRETEAR
FNEQSKDIKKEILAGVQQVQSNTTEISDLKRSLQGLEIELQSQLAMKQSLEKTLAETEGRFCSQLGQLQN
LITSVEEQLFQLRNDMELQSNEYKQLLDIKTRLEQEIETYRKLLEGEGGKLLAGMSDGKTPSDSSKDSSR
TRRVKMVIEEEVNGKVISSSSVREIEEKH

>Xenopus1_09
MSLRSSSSYSVQSKSVSGGGGFGSGYGASFGGGSFSGGSYGGSSFGGGFGAGFGGGAASGFSASSAGGFG
AASASSSFSSFGGSDKQTMQNLNDRLAAYLEKVRALEAANADLELKIREWYEKQRTSGIGAGAKDFSKYF
DIITDLRSKILFATIDNSRVVLQIDNAKLAADDFRLKFENELALRQSVEADINGLRRVLDELTLARGDLE
MQIESLTEELAYLKKNHEEEMSIAKSSAAGQVTVEMDAAPGTDLTNILNNMRADYETLAEKNRRDAELWF
NQKSGELKKEILTGVELVQSSKSEISDLRRSLQSLEIELQSQLAMKKSLEDSLAETDGRYGAQLQNIQLS

IRSLEEQLLQIRSDMERQNMEYKQLLDIKTRLEMEIETYRRLLEGELSSSSSSVQVITSQSSSSSKKDPS
KTRKVKTIVEEVVDGKVVSSRVEEVEENVK

>Xenopus1_10
MTSYRSSSASYYSSSSGKGGFGGSSYGGSSFGGGFGSGAGSGFSSSGGNFAMAAADSSNLSNFGGNEKHA
MQNLNDRLASYLDKVKALEASNVTLEGKIHDWYDSKVDAGIGAGSKDYSKYFEIINELRSKIGAATVGNA
TVTLQIDNARLAADDFRLKFENELALRQSVEADIAGLRRVLDELTLARGDLEMQIESLTEELAYLKKNHE
EEMSHAKSQSAGKVSVEMDAAPGVDLTNILNNMRADYEILAEKNRRDAELWFNQKSGELKKEISVGVEQV
QASKSEITELKRSLQSLEIELQSQLAMKQSVEGNLSEVQGFYSAQLLQIQNTISSLEEQLLQIRSDMEHQ
NMEYKQLLDIKTRLEMEIETYRRLLEGELGQMTTVAKASSVESKTESSTTRTTMVKTIVEEVVDGKVVSS
RIE

>Xenopus1_11
MTSYRSSSASYYSSSSGKGGFGGSSYGGSSLAGSGFGGGFGSGAGSGFSSSGGNFAMAAAASSSLSNFGG
NEKHAMQNLNDRLASYLEKVRALEASNADLEGKIRTWHEKQTGTGLGAGSKDYSKYFEIISELRGKIHGA
TVDNATVTLQIDNARLAADDFRLKFENELALRQSVEADIAGLRRVLDELTLARGDLEMQIESLTEELAYL
KKNHEEEMMHAKSQSAGKVSVEMDAAPGVDLTNILNNMRADYEILAEKNRRDAELWFTQKSGELKKEISV
GVEQVQASKSEITELKRSLQSLEIELQSQLAMKQSVEGNLNELQGFYSAQLLQIQNTISSLEEQLLQIRS
DMEHQNMEYKQLLDIKTRLEMEIETYRRLLDGELGQMTTVSKASSLESSSESSSTTTTRTRTVKTIVEEV
VDGKVVSSTVS

>Xenopus1_12
MANMYRTIAVDRAISSPGGNYSIIPQTRYAGSVYGGAGGYGTKISKSSSYSSGYGGGSSSSLLVNTNSDL
LLNGNEKQTMQNLNDRLATYLEKVRSLEKSNSKIELQIKEWHSKNAGVGEKDYQGYYKTIEALQNEILNS
TMVNAEILLKIDNAKLAAEDFRLKFESERSLKIGVEKDIIGLRKLIDDLNLQRIDLEHQYESLQENLAYL
RKNHEEEASVLRNQITGHINVEVDAAPSVDLAKVLADMRAQCEAVVSKIKQDAKQQFETQIEEVNIQIGG
NVTELEKHRSSVKEVKRHVQSLEIELQSEISKKDALNATLNNINAQYAAQLLHMQTAIKCIEEQLIQIRS
DMSRQSQEYEILLNIKLRLELEIETYRRLLEGEDV

>Xenopus1_13
MSSSSSSFQTVVSSSKVKATRTSSYGGFSSASLSGAGFGGSTATQSSSLSFNNNFGGSDLLLAGWEKHTM
QNLNDRLAAYLDKVRALETANHHLEVQIKEWYEANMHCYKERNDYSKYMVVIEELRRQITNGTPANARIL
LQIDNAKLAADDFRMKYENELALRQSVESDVNGLRRVLDELTLARSDLELQLEGLNEELAFLKKNHQEEI
SSIKVGSGQVTVEMDAAPAVDLTLLLNNMRADYEVVAEKNRKEVEEWFLKKSAALKQDILIEAQEVQSSH
SEISELKRTQQTLEIELQALLSMKSSLEGTLSETEGRYCLSLSQIQMRISSIEEQLHQIRMDMSRQNEDY
LRLLDVKTRLEREIETYRILLEGELNVKVEKVVEQVVEVKKDPTKTRKVKTIVEEVVDGKVVSSSVQEVE
EKLN

>Xenopus1_14
MYKHVVVKGGHVQVGGSGGGNYRKISVGGYASSVYAGAGGRNTKVSTDGSPGELTSRHLYIKTVSIGGNE
KETMRNLNNRLATYLEKVRSLEKANAELEGQIKEWYQQNSGFGKRDDAHYFQVIEELTKQIVNAKMENGK
LLLQMDNATLALDDFKMKLEYELNLGAAVEKDLSELRKVVDDLTMTRTDLESQIESLKEEMVYLKKNHTQ
DMVGLHKQEGTIDVEVDAAPSVDLGKTMEDMRSQYEQLVEKQRLEAKYGFDRKVEEWNEEVQTNTAELEK
CRKDLSELKHKVQDLEIESQAELSKRKAAGNALDNVNSRYTMELADKQEKIARFEEHLQQTRRDVGFQIQ
EFTILFDLKNRLEAEISTYRRLLDAEGQ

>Xenopus1_15
MSFNSNFHFSSSSFSETFSKVRISSSSSSSHSGSNGSRTCISKPNPFPSSPGTFDCTMDFRETLSAPNGK
ETMQNLNDRLASYLEKVRSLEEANTGLESKIQLWHRNRAQNHKRDYSVYEKTIGELQSQLVDGHMNGAKI
KLQMENAKLAFDAFKRKYETEKTIRTALEGDLESLRRAMDNLTIVRTDLEMEVEGMRKELIYMRMGHEED
IKLAQFQKKGASVDVKIDAPPSVDLAKTIAEIRKEYETLIEKNRREASEWYSHQXXXXXXXXXXXXXXXX
XXXXXXXXXXXXXXXXXXXXXXXXXXKHGLEGTLAETEANAAGQLQKIQATICRMEAELSNVRGELERQN
IEYRALLDVKTRLENEIATYRELLEGDKTGGRSRNSEGGGKITTIVQDMVNGRVISSKVSEIPKKL

>Xenopus1_16-HK
MGGGLGGRSEGLLNVGEKETMQQLNDRLANYLGRVRSLEEENGQFERRIREWYDKQVPYNFPDFNNFFRT
IEELQNKILHACTGNANLILQIDNARLTSDDFRNKFESEAAFRMGVEGDMNGLRRVLEELNLGNNDQEIQ
RQNLSEELTYLKKNHAEEVGSLRTQLGARVNVEVNAAPAVDLNQVLGEIRDQYETIVERNRREAENWFLN
KSEELNQQMNSSADQLQTVHTEVIQLRNTVQGLEIDLQSQNSMRSALEGTLADTESRYGGQLSQLQFLIN
NVETQLGSLRSDLERQNFEYKALMDVKTHLEMEIATYRRLLDGEDRQRGSRQSGEVRDMGSNRSRTVTMV
EDIKEGFRKA

>Xenopus1_17
MSYSLSQSSSSRMSSGISSRLSDGIYRAASMQGGYGGSGVSMSSAKFTSAAGSGMGFGSGFASGASAGSG
AFYGASFSSGGGDGLLSGNEKYTMQNLNDRLASYLAKVKTLEVANTDLEAKIRDWYSKQGSVAVSEQSFG
TLYTTIGELRQKILAATINNSKLVLEIDNARLAADDFKLKYENEFSLRQTVENDISGLRHILDDLTMTRS
DLELQIESLKEELIYLKANHEEEVSEKKQHAAGTVSVELDAVPGVGLLNTLNDLREQYESIADRNRREAE
AWFLSQVEGLQKEVVSSTQQIQSTKSESTELRRGLQSLEIELQSQLSAKAGLEASLAETEGRYAAQLFQI
QNIISSMEAQLSDLRSDLERQNQEYRTLLDIKSRLEQEIATYHQLLEGQGGLIAAGANSSSGSSSGSTTT
RVHAIIKEESGGKVVSSTLLKSF

>Xenopus1_18
MSFSSSRVGRLSTGVYGAPSGLQGGYGGSGISKSTVRLTSSALGSNDLSGFGGSGASGFSTSLAFTNGDG
LLTGNEKSTMQNLNDRLSNYLEKVRCLEETNADLEKKIHGWHENQGSVTVHEQNYLPYFATIDELRQKIH
AAFIDKNRVLLGLDNARLAAEDFKFKYENEHSMRLNVETDINGLRKLLDEMTLSRADLELQIENLKEELT
YMKKNHQEEMGAKSQQVIGNVNVEMDAPPMNDLTKILSEMRENYEYLMSKNRRDLETWYLSQSESLQNKV
EYITEQTSKSEITDVRRNVQGLEAELQSQYSKKAAIEDALADTEERYAMQLSHIQSIIGGIEAQLYDLRY
DLEHQNQEHKSLLAIKSHLEAEIATYHKLLEGQEFSIGDSKKGSGGTATITTTITTVVKEEEVSSGQKQN

```
>Xenopus1_19
MASYSVRQTSSSSSYRGLGAPMGAGRMFAKAPSVHGGYGGQGISVSSAKFVSSGIGGGLGGGSYGGGFSS
GYGAGSGYGAGSGYGAGLSVNVSGDVLLSGNEKETMQNLNDRLAMYLDKVRALEEANSGLELKIREWYEK
QGPSPSRDYSHYFAIIEDLRGKILDATVDNSKIVLQIDNARLAADDFRTKYENELALRQSVECDINGLRK
VLDELTLCRTDLEHQIESLKEELAYLKKNHEEEMNAMRGQVGGQVSVEVDAAPTVDMNKKLAEMREQYEL
LAEKNRRDAEAWFFSQSEELNKVVATHTEQIQTSKSEITDLRRTIQGLEIELQSQLSMKAALENTLSETE
ARYGMQLAQIQNVIGSIEAQLADLRSDMERQNHEYKLLMDIKTRLEKEISTYRQLLEGHDAQIGGGKHKD
AKTGGK

>Xenopus1_20
MTMQNLNDRLANYLDKVHALEEANAELEKKIKDWYNNHRPASSKAPDYGEYFKTIDDLQNKIIAAYANNH
KAILAVDNTRLTIDDYRLKYENERALRQSAEADISGLRRVMDDLTLCKSDLEAQVESLTEELAYLKKNHD
EEVKAMQGQATGAVNVEMNVAPSVDLSKSMADMRAEYEKLAERYRREVEEQFQAMTKDLQKQVVASVREV
QTSKSEITDLRNVLQSLEIELQSQLSMKAALEASLAETEGQYCVKLSHIQELILRVQNNISDIWTQMETQ
SSEYKLLLDIKSQLEQEIATYRELLDAQDIK

>Xenopus1_21
MSLRLGSATGKGCSVFESSGVSASGNSRGRAGGVYGGGAHVVNYAGSLRSGYGGHGGHGGSRGVGFSGAS
AGGVCFDSGVGFGHGGESGFGGGAGYGGGYGFGAGYMAAGGGHRSGFGEEPGLLSLNEKITMQNLNDRLA
TYLNKVKSLEEANTDLEKKIRNWYDTHGPSPVQEKDYGPFYRTIEDLQKKILGATIDNGRIVLQIDNAKL
AADDFRMKYENELILRQTVESDINGLKRLLDDLTLNRSDLESQLENLKEELAGLKKNHEEEMKALKVQLV
GDVTVDMKAAPGIDLQKVLGDLRHDYEQIMQKNQKDIEFWYQEKTAELRKNVSGSTQEIQTTNTQVTELR
RTLQNLEIDLQAQLSTKAALEASLAETEGRYCLQLSQLQALIQNVEAELANIRCEMENQSHEYKILLDIK
TRLEQEINTYRNLLDGQGTQFPKGSYDSSFSSGSQSDKQGGSSYYVRTRVEDGDGKVVSSRDQYHHSGYK
Q

>Xenopus1_22
MSSYSIRQTTTSSGSMKGLGGFGGGSSRVSGVYRAPSIHGGSGGQNVSISSTRVMSGMGGGYGSCGVDAG
LGGGYMSNYGGGYGSGYVFGGGVAGGEGLISGSEKYTMQNLNDRLAAYLEKVRSLEKANGELEIKIRDWY
QKQAPGPAADYSHYYKTIEDLRNKILSATMDNAGLLLQIDNAKLAADDFRTKYETELALRMNVEADINGL
RRVLDDLTINRSDLEIQIESLKEELAYLKKNHEEEMIALRGQATGQVNVEMDAAPSSDLTKTLAEMRIQY
ENLAEKNRRDVEEWFFKKTEELNKEVANSSVMIQSSKTEITDLKRTLQSLEIELQTQLSMKAALEGSLAE
TEGRYCVQLSQIQGLISSVEAQLGELRSDMERQSHEYKILMDVKTRLEQEIATYRRLLEGEDAHLSQKDG
PKTTRQVRTIVQETVNGNVVSSREDVRKLPY

>Xenopus1_K117
MSFSTRSVSQSSRYGVLASPMVNRALSVAGGAQTVRMSSAKLTSSAFAGPSALDVSSFGNNGKETMQNLN
DRLASYLERVRSLEQANHELELKIREYCEKKAAIGSPDVSGYYNIINLLRSQINDASIDNARLVLQIDNA
KLAADDFKIKYESELAIRTGVESDIIGLRRVLDELTLNKTDLELEIESLKEELIYLKKNHEEELAVARAS
ARGQVDVQVDSAPPVDLSQIMSDVRAQYEGMMEKNRQEVEAWYRGQSENLNKEVATNTAALQTSKSQITD
LKRTIQSLEIELQSLLSMKGALEGTLAETEARYGAELNHLQAMITQIELDLQNLRSDAERQSLEYKLLLD
AKTRLEMEISTYRRLLEGEDTRFSQTETQKAVTIVSKEQSKSSIKKVKTVIEEVVDGKVVSSRVEEVTEK
S
```

B

```
>Chicken_K80
MTNLCKAFSSGSLCSWEASGRMSQGRAGSTSPDSLGHCSPSSVTSPSCGAPYGDGGYQSSSYLSIDGRLL
APVHLDIDTDFHAVREQEKEDIKLLNNQFVTLIEKVQCLEQQNKILTTRWNFLKDQDNSHSESDVKAIYD
QYMSKMNQEMQALNYEQENLESELTEVLSTMDNFRSKYEDEIRLCSGMEYTFMELKKDLDASTLHRTELE
VKLSGLQELMDLKKTIYEQELEELLTEIKDISVVLGIDNTCKLDLSRIVEEVRAQYEALALRSWEEAEAL
TRRKLNEGNTQSATYGGHLLDSRREIADLNIQIQKLRSCIVSQKSQCLYLEENIKEAGEHGEMALKDAKA
KLVKLEEALQKSKEDMAHLVKEYQELMNIKLALDLEILTYRKLVEGEESSMESPIPTVISTIHSRPKHLS
ASTLRNSKLFARGTSSANGEMKLNGGNTKPLPSRSHSNNLAAPEADPYTCSARPKLSSLLTEECS

>Chicken_K7
MSRSVSFSSRSVAVPGVSQVRVSSVSSVRGGGSLNKAGGFGSSSLYNLGSANKRISLGGSSSYSVRSGYG
YGGMGFGGAIAPSAGIQEVTVNQNLLTPLNLEIDPNIQRVRKEEKEQIKTLNNRFASFIDKVRFLEQQNK
MLETKWSLLQDQKTTRSNIVPMFEAYITNLRRQLDGLLNDKGRLEGELRNMQDLVEDFKAKYEDEINKRT
TAENEFVVLKKDVDAAYMNKVELEAKVDALTDEINFLRSLYEAELRELQAQISDTSVVLSMDNSRNLDLD
SIIAEVKAQYEDIANRSRAEAESWYQSKFEALQVTAGKHGDDLRNTKNEITEINRVIQRLQGEIENAKAQ
RAKMEAAIAEAEERGELAVKDARAKLEELEAALQKAKQDMARQLREYQELMNVKLALDIEIATYRKLLEG
EESRLAGDGVGSVNISMVSSSGGGSSGFLGGGVRGGLALGAGMGSGALGFSSGGSTKSYTVTTTSSTRSF
RK

>Chicken_K84L
MSCSYGTNVGRVGNFSSSSVALSRNGQSFGRVLCRQRGLGSFSTRSLSGVASSRPRIAVGRCPPMRCGYG
LGAAGAGLGCRGAGFGYRVGGSSRPCTIVPITVNEKLLQPLQLELDPNVQTMKYQEKEQIKTLNNKFASF
IEKVRLLEQQNEVLETKWSFLKGQKHGRNTIVPMLEACIGNLKKQLEALGCNRARLETQLKAVQEALETN
KKIYEDERSRRSSTEKELIALKKDVECFFLNKAELGAKVGRMEGEFGFLRVLYEEESQQLRAHISDTAVV
VQMDNSWDPNLDGIVANVKAHYEDTACRSRAETEAWCKGKFKELQVTAGRNADSLQEKKREAAELARMVK
KLSGEVRSAKEQCCKLETAVADAEQRKEMSIKDAKSKLTELEAALQKAKQDLARQLWEFQELMNIKLALD
VEITTYKKLLEGEESRLCAEGGVPINISLPFTAVCHSQGGLAYSPEPSFAGLANGSSRIRSGSTHVKVVS
MGKSTM
```

>Chicken_K5L3
MSRQCTVRSQGRTGFSAASAFIPSACSTGFISRSVAQGGSCSTAAGYGGFGGGFGSRSLYSVGGCKRISM
AGRGSGFYGPAGFGAGTGISCGFGGAFGFGGGMGGPGFPAVPTGGIQEVSVNQSLLKPLNLAIDPSIQSI
RKEEKEQIQTLNNKFASFIDKVRFLEQENKVLETKWALLQEQGHKTVRNNIEPLFEAYINNLRRQLSSLL
ADKENIGGELSKVQSLAEDFKSKYEEEINKRTAAENEFVILKKEVDSTYMTKTELQARLDALLEEIDFLR
ALYEAELSQLQSQIADTSVILTMDNNRSLDMDSIIAEVKAQYEDIAKRSREEAESWYQSRYEELQATAGR
HGDDLRSTKQEISELNRHVQRLRAEIDSVKKQCASLQTAIADAEQRGELALKDARDKLAELETALQQAKA
DLARQLREYQELMNVKLALDIEIATYRKLLEGEECRLSGEGAGAVNISVTRTSVGSGYGSGSCQGFRGSS
GAGAGLCAGGMGFSSGSGQGMAGSCVVGGSSSSMQYISSSSTKRCY

>Chicken_K5L2
MSRQSTVRIQRGRSGFSAASAIVPGTCRTSFSSRSVTRVGGCNAGSGFARVGGGFGSRSLYSFGGCKRIS
AAGRGGGFYGTAGFGGGAGSMLGYGYGAFGGPGFPAGGIQEVSINQSLLKPLNLEIDPSIQRIRKEEKEQ
IKTLNNKFASFIDKVRFLEQQNKVLETKWSLLQEQGMKTVRNNLEPLFETYINNLRMQLNSLLSDKGRLE
GELVNTQYLVEDFKKKYEDEINRRTIAENEFVTLKKDVDASYMNKVELQAKADALTEEINFLRALYEAEL
SQMQTQISDTSVVLTMDNNRNLDLDSIIAEVKAQYEDIANRSRAEAESWYQTKYEELQATAGRHGDDLRS
TKQEISELNRHVQRLRAEIDSVKKQCANLKAAIADAEERGEIALKDAKAKLAELEDALQQAKADLARQLR
EYQELMNVKLALDIEIATYRKLLEGEECRLAGDGVPVNISVTRTTVGSGYGGGSNLSMGGGMCSMGNNYS
CSGPGGSSALGGGSSSSVKFFSSSSSRRSYRS

>Chicken_K5L1
MSRISYRSSTGGGMRGFSSGSAIVGGSGGGTRSSFSSVSVSRVGGGRAGGGGGFGAGGGFGSRSLYNLGG
SKRISLGGLRSGAGVGYGFGGGAGFGLGYGGGAGAALGLGGAGGGGGYGLGGFGMGGPGFGGRGGPGFPV
CPPGGIHEVTVNQSLLAPLKLDIDPEIQKVRTQEREQIKTLNNKFASFIDKVRFLEQQNKVLETKWSLLQ
EQGHTVTRKSLEPLFETYINNLRRQLDSLMGERGRLDSELRSMQDMVEDFKNKYEDEINRRTGAENEFVV
LKKDVDGAYMNKVELQAKADGLADEINFLRALYEAELSQMQQQVSDTSVVLSMDNNRSLDLNSIIAEVKA
QYEDIANRSRAEAEAWYQNKYEELQVSAGRHGDDLRNTKMEISEINRMVQRLRNEIESVKKQCANLQAAI
AEAEERGEMALKDAKSKLAELEDALQKAKADLARQLREYQELMNVKLALDIEIATYRKLLEGEESRLAGE
GVGAVSVSVVSSSSGMGYGGGMGGGLSTGGGLCMGGGLSMGSGLGMGGGGGSYTMSSSVGGGGFGGGSGS
YSYGGGSNFSSSSSRGVSSSTGGSVRIVSKTTTSKKTIR

>Chicken_HBS1
MSRQCYTSSSLLGRRGFSSASAVCGLGRAGSSSASVCQPAARRCGLGGFSSRSVCDLGRGQRISFGGSCR
SAAYGAGSVGRCGTAYGGGRFGVGTVVGFGNCGGYGGLGGYRGLGDGVAMGGYSGGISIGLGGGRSEGIR
GVSIHPELLKPLCVGVDPEECQVRTHEKEQIKNLNNQFACFIDKVRLLEQQNKVLTTKWELLQQYVLPAS
RRNLEPVFENFICNLRKQLECVLGERERLENEERCLRDLVQEYKCKYEDEINKRTAAENEFVVLKKDVDC
LYLTKEELEVRVGLLRQQLEFLKCIYAEERAQMDCQLCDTSVIVQMDNSRDLDMEGIIKSVECCYEEIAQ
KSKAEVEAFYQTRLEELHSSRGKFCDDLRNNQSEIAELNRMIQKLQCESDNVKKQIAALQTAICDAEQRG
DCALKDARQKLVDLQTALQQAKDKMACLLRDYQELLNVKLALDIEIATYRTLLEGEESRICTGNPVSVAV
VSGGGTVGECRSLSGIGGKCSVKTGASSGLGGVVSSFGVSGAGFSARSVDCVPRVGGGFGARSAASCVGR
EVLSGADGLQCAAGVGNLVCAGVEQCSPGTVIIPGPGVCGGGNRYSTAVRVVRTSR

>Chicken_HBS2
MSRQAPTVRSVLGRRGFSSASEICGRSYAAACQPARCAAGAYSSRSVCNLGGSRRISYVNGVCGNGCFGD
FGFGGVVGYGNAGGRVSLCGPRGYSIVRGYPDRKGEGIQGICIDERLLKPLCVGVDPLEHEIRCQEKEQI
KTLNTQFACFIDKVRFLEQQNKVLETKWGLLQQYVLPKKGKNLELYFENYICDLRKRLDCLLCEKQKLGN
EECATSQLVEEFKCKYEEEINRRTTVENEFVALKKDADCIFLNKEELEVKVDLLRRQLELLKCVFEEERA
QVDRQLCDTSVIVKMDNNRDLDMESIIKNVECWYQEIAQKSKEEVDAFYQTRFQELQDKRGKYCDDLQSN
KCEISELTRMIQKLQCELENVKKQVSCLQTSICDVEQRGDCALKDAREKHVELQNALQKAKDELACMLRD
YQELLNVKLALDIEIATYKTLLEGEESRICVGNPVSVSVVSSGYNIPDDCGMLAANAAACGYGSLGRRSG
RHSSQNGGFSSRSAGIHPKRVLSSVAKPCVPEVFCQAGGVNCKTGGFSSRSGGYPARTVISTASGGLNAR
MGACQPGGVVSFGNQGCVIRQLAGPPVMVSSSPEVVACNNGVVGNFGVVRDPCVVP

>Chicken_HBS3
MSQQLAAGRALHGRKFSASSAGGGVSCCRSSAFPSTAPFGRGYGTWSSRSLQNMDGCKWISSGICASGEC
GRHGRMLGFHGCGREGIRAVHVNENLLRPLDVKIDPEIQRIRRQEREQMRSLNNQFACLIDKVQHLEQQN
RLLATKWAFLQKHILPSQKNFKHVFNTFICSLQRRLDSLLHERGQMELELNNTEKLIEDLKCKYEQEVNR
CTAAENEFVLLKKDADCVYLTKAELEAKLETLKQQIELLKCMSAQGTAEMEKSLCDTSVVVKMDNSRSLD
MEGILRGIELWYEDVAQKSKAELDALYRTRYQELEETKRKQCSELKSHQQEIEELSFVMQRWQCDLENVK
KRVSSLRTSVSDTEQRGDRALKDAREKHLELQNALQKAKDELACMLRDYQELLNVKLALDIEIATYKTLL
EGEESRIRTGCPVRVLITTPCNTSSGCSTSLGHGPGTASRKCGSSPGASRASQPGNDGVYHRGSTSSAGK
QCVPSGPRCGCFPRGHQRSCRGGKHTCQSSAHVSAAELCCGTAGSSESSAQPSAPCWIGARSSAGTPCSR
STTRV

>Chicken_K78LT
MSHHFFRFGDGNFSSSSAHCGTLGSGRGSSAMSHWEDDGGQRFGGYGYGYGHGYGSRSLHNLSGFRNFST
GGIYGGDGGWYGRHGGYYDGRYLDRDLGRRVYFVGDFQGGAGRMYGGGSSRGMLSGGLREQGAGQGSGHP
AAGGGIEEVHVNTNLLRPIQVQVDPEFQRMRSDEKEQIKTLNSNFASFIDKVQCLERQNQALMTKWELLQ
QQSSRPEESRSITSFFQSYINNLQRQLDMLQSQKEQLDPEAYEMLQLVEDYKKRFEDEINKRASKEEEYV
ELKKELDSTYMRKMEFEVRVEILKQELEFLRCLYEAELSQLQTVVDNTNVILSMDNGREVNMDGIIEEVR
QEYEKIAQKGKDEVNAMYQGRYQDLQNMRVNQREQLRYTYQEIQELTRQIQRLQPEIEIVRRENDSRQEA
IRDAEQRGSSAVKDGQKKLQELENALQQAREDLARILHDYQELLNAKLSLDVEIAMYRSLLEEEETRIQE
GSPATVRVVTRSGSSAEGFGRQYGDLNREKEEMSTDDSNRMTWTEITPGGGREGERRDSGSTYGSREHRG
GEAGRYHTGRSSGSSSGYGYETGGRISRAGSTSGGGHSSGGFGGSSGSGRCSPHGGGLSSGGSSGSGRRG
SISGEGGSSGHGGSSYGSGSGGSICGGGGSGSSSGGSFGSGGSSSGGGHSSGGFGSSSGSGSGRRGSISG
GGGSSGHGGSSYGSGSGGSICRGGSSGAGSGSGGSFGSGGSSSGGGHSSGGFGGSSGSGRCSPHGGGLSS
GGSSGSGRRGSISGEGGSSGHGGSSYGSGSGGSICGGGGSGSSSGGSFGSGGSSSGGGHSSGGFGSSSGS

GSGRRGSISGGGGSSGHGGSSYGSGSGGSICRGGSSGAGSGSGGSFGSGGSSSGGGHSSGGFGGSSGSGR
CSPHGGGLSSGGSSGSGRRGSISGKEEAQVMGDRAMAVEVEAPSVEEEALGPRPEEALAVVGPRLEVATA
LEALARALAADLAGEGPFLEAVEAQVMEVPAMAVEVEAPSVEEEALALGQDLGVALAAVVRHLEVATALE
ALVEALAVAAAVLTVEG

>Chicken_K78L4
MSRQSVCRSFGGGSRRGYSSCSAVGGGFGGSGGRSRISYSSFSTSRGTGGGGRCGGFSSRSLHNMGGSGR
ISMGGSYGGGYGCRIVGLGGGYGGGFGSIGGGVIGGGVGSFGGPVRGGPGFPGGIQPVQVDSSLLRPVHV
DIDPQIQQVKCQEKEQIKTLNNQFASFIDKVRFLEQQNKVLSTKWELLQQQGPSGPRKNLDVIFENYIQG
LRRRLESLLGQRGELESELQNMRQYVEEYKTKYEEEINRRTAAENEFVVLKKDVDCAYMTKVELEAKVGA
LTDEISFLRCIYEEELAQMQTISRDLSVVVSMDNNRHLDLDSIIEEVRRQYEQIAQNSRAEAEAWYQSRY
EELQTTAGRHGDSLRNTKIEIQELTRNVQRLRAEIENVKKQNQQLQAAIAEAEERGEMALKDARRKLEEL
ECALSKDKEELARLLKEYQELLNIKIALDVEIAMYRKLLEGEENRLCGDNPSNVNVSVVGRTTIAGGRAG
GFGAGSGVGGGVCTVGGGSIIGGSCGVGGGVYNGGFSSGSGRMCSSGGGTFIPTGASSVRRCVTTTTVKS
SGVKY

>Chicken_K78L3
MSRQSACRSYGGGSRRGYSSCSAIGGGFGGGGGSRSRISYSSYSSSRGGGGGGQCGGFSSRSLHNMGGSR
GIAVGGCYGGGGRMGGYGGGIGGYGRGICGGGMGGYGGGIGGGGMGGFGGGMGGGGMGGGGMGGFGGGIG
GGIGGGMGGFGGPGFPGGIQPVHVDSSLLRPVHVEIDPQIQQVKNQEKEQIKTLNNQFASFIDKVRFLEQ
QNKVLSTKWELLQQQGPSGPRKNLDAIFENYIQGLRRRLESLLGQRGELESELQNMRQYVEEYKTKYEEE
INRRTAAENEFVVLKKDVDCAYMTKVELEAKVGALTDEINFLRCIYEEELSQMQTISRDLSVVVSMDNNR
HLDMDSIIEEVRRQYEQIAQNSRAEAEAWYQSRYEELQTTAGRHGDSLRNTKIEIQELTRNIQRLRAEIE
NVKKQNQQLQAAIAEAEERGEMALKDARVKLEELEAALSKDKEELARLLKEYQELLNVKIALDVEIAMYR
KLLEGEENRLCNDGMSNVNVSVVGRTTISGGRGGMGGGFGGSGMGGGFGGSGMGGGFGGSGMGGGMGGGV
CGSGGGFGGGSSGGSCGMGGGMYSGGFSSGSGRMCGSGGGNFSSGGGSSSIRRCVTTTSVKSSGVRF

>Chicken_K78L2
MSRQSVCRSFGGGSRRGYSSCSAIGGGFGGGGGRSRSSYSSFSMSRGYGGGGRCGGFGSRSLHNIGSSGR
IAMGGCYSGGGYGGRMGGFGGGYGGLGSYGGGMGGGGMGGFGGGMGGGGGMGGFGGGMGGYGGGMGGGGM
GGYGGPGFGFGGPGRGGPGIQPVQVDPTLLQPVHVEIDPQIQQVKNQEKEQIKTLNNQFASFIDKVRFLE
QQNKVLSTKWELLQQQGPSGPRKNLDVIFENYIQNLRRQLDSILAQRGQLESELQNMQQYVEDYKNKYEE
EINRRTTAENEFVVLKKDVDTAYMTKVELEAKVGALTDQINFLRAIYEEELSQMQTISRDLSVVVSMDNN
RHLDMDSIIEEVRRQYEQIAQSSRAEAEAWYQSQYEQLQSTAGMHGDSLRNTKIEIQELTRNIQRLRAEI
ENVKKQNQQLQAAIAEAEERGEMALKDARVKLEELEAALSKDKEELARLLKEYQELLNIKIALDVEIAMY
RKLLEGEENRLCNDGMSNVNVSVVGRTTISGGRGGMGGGFGGSGMGGGFGGSGMGGGFGGSGMGGGFGGS
GMGGGMGSGGGFGGGSSGGSCGMGGGMYSGGFSSGSGRMCGSGGGNFSSGGGSSSIRRCVTTTSVKSSGV
RF

>Chicken_K78L1
MSRQSSAFKKGFSSSSACLGGKNKVTFSTVARGCRGPAGNAGGFSSHSLYSLGGCKSTALGGFGAGGICR
GFGAGGQGFGYGVGNGIGFGGGAGCGFGGGFGGFGGGFDGGVGGFAQCPPGGIKEVTINQSLLTPLHLEI
DPEIQKVRTQEREELKTLNNKFASFIDKVRFLEQQNKVLETKWKLLQEQSPTTSTRSLDPYFEAYISRLR
KQLDGLSSEKIQLESELKSFQDMVEDFKTKYEEEINKRTAAENDFVLLKKDVDGVYMNKVELQAKLDSLA
DELNFLRNLYETELCQMQKTVSDTSVVLSMDNNRCLDLDSIIAEVKAQYEEIANRSRLEAEAWYQSKYKE
LQATAGKHGDSLRDTKAEISELNRVIQRLRAEIENVKKQCESVQSSIADAEQRGELALKDARDKLTELET
ALKKAKADLAQQLRDYQELMNVKLALDIEIATYRKLLEGEECRMSGECQSNVSISVVGGSSSIGGGYGGG
LCLGGGLGFGSGSGRGSCSTGGAGFCYSLGGGGFSSGTGSSVVVGSGGVLKNSSVSARRP

>Chicken_K8
MSVTITRKSVRSSSAVPVRSFSSRSFTAGPTVRMSAASAYGTLSGSRFGVGGLQRGPVAVSGGGIAAVTV
NQSLLTPLNLDIDTSIQQVRKEEKEQIKTLNNRFASFIDKVRFLEQQNKVLETKWSLLQSQQPQRSDLRG
LFEGYVGTLRRQLEGLGQERLRLEAELGSMQGLVEDFKNKYEEEINHRTEKENEFVLLKKDVDEAYMSKV
ELESRLESLTDEINFLRQLYDEELSQMQTQISDTSVVLTMDNNRSLDLDSIIAEVKAQYEDIANRSGLEA
ESWYQTKYEELKTTAGKHGDDLRTTRSEISELNRMIQRIQAEIEVLKNQRATLETAIAEAEERGEMALKD
ARAKLAELEAALQKAKQDLARQLREYQELMNVKLALDIEIATYRKLLEGEESRLESGMQNLSIHTRTTGY
SGGFGGGFGSSFSTGYTVTPPALESAAVPKSRAIVIKKIETRDGKLVSECADVLTN

>Alligator_K80
MDTPSKAFSTSSLSSGGASGRMSRASSIFAAVDCNGCSSPGSTASRNFSSASLSGNRPSAYPPLSVDKTL
LTPLHLDIDPSFQAMKNREKEEIKVLNNQFVALIKQVQSLELQNKMLETRWDFLKDQDNSSSEVDIKCIY
DEYMGRMEQEMKSIDQEKEELESELTKVLDSMDDFRAKYEGEMQLRNSLEYTFLELKKDLDAGTLHRTEL
EVKLNGLQEHMELKKSVYEQELQELLAEVKDISVVLGIDTRCNLDLHSIVEEVRAQYEALAVWSWKEAEA
HIWSKLNEGVPRSAMYGNHLFNSRREIADLNIQIQKLRSCIVSLKSQCLSLEESIKEAGEQGEFALQDAS
AKLASLEEALQKGKEDVTHLVKEHQQLMNTKLALDVEILTYRKLVEGEESSIESPAPTVITSIHSKPKSA
ATSFSSSAASKADPRGRGNPTEKMNLNRGTVEAVLSRSQSDGSASQNTPAQHDGARPKPRSLSNVEGHLE
KPNQEK

>Alligator_K7
MSRSVTFSSRSAAMPGISQVRVSTVSSSRGVGGGAGLGRVGSFGSSSLYNLGSSSRRESPIIRGSSYSVR
SGYGYGGGLPAGFGSGLPSPGIQEVTINQNLLAPLNLEIDPTMQKVRQEEKEQIKTLNNKFASFIDKVRF
LEQQNKMLETKWSLLQDQKTARSNIAPLFEAYISNLRRQLDGLMNDKGRLEGELKNMQDLVEDFKNKYED
EINRRTTAENEFVVLKKDVDGAYMNKVELEARVDALTDEINFLRSLYEAELQELQTQISDTSVVLSMDNS
RNLDLDSIIAEVKAQYEDIANRSRAEAESWYQSKFEVLQATAGKHGDDLRSTKNEIAETNRMIMRLQSEI
DNVKNQRAKLEAAIAEAEERGEMAIKDARAKQDELEAALQKAKQDMARQLREYQDLMNVKLALDIEIATY
RKLLEGEESRLAGDRPDKVSISMVSSSGGSGYGSAGGIGYGSSFGLGGAMGSGALSFGTGGGSSPVKAYS
MTTTSSSRTSIR

>Alligator_K84L1
MSCRSYRVQNFSSSSAVLPRNMRPYSVSCQGAGGVGYRGLSYFGSRSLSGAAPYKPRIAVGRCPPLRYGY
EYVLGHGYGNAGFSYRVGGVSGPCPPIAPVTVNEQLLQPVKLEIDPTMQTVKFQEKEQIKTLNNKFASFI
DKVRFLEQQNKVLETKWSFLQNQQCRRNTIMPTLDAYICNLRKQLEALGCEGAQLEADLKAAHEVMETNK
KMYEEECSRRTCAENEFVVLKKEVDCVFLNKAELEARVESLKDEIIFLKSLYEEEIHMLHSHISDTSVIV
QMDNSRNLDLDGIITEVKAQYEDIASRSRAEAEAWYESKFEELRVTAGRHADNLRDTKNEIAELTRIIQR
LRGEVGTAKDQRCKLEAAVAEAEQQGELAIKDARCKLTDLETALQQAKADLAHQLREYQELMNVKLALDI
EIATYRKLLEGEEYRLGAEGACPVNISVCRSQGGLICDPDPCIGGGYSATRSSMRISGVSCGRGIYSTAM
GPTVGDMSSPCDPEDLMLFNIYHMRPIVNSVNHLL

>Alligator_K84L2
MSCRSYRISSSGGGMRGFSSSSAVIPRNVKRYRVSTISCHGGGVGYRGLGYFGSRSLGGIASCKPRMAVG
SCPPLRYGYGYGLGYGYGAASFGYGAGGFSGPCPPIAPVTINEQLLHPLKLEFDPNLQSVKYQEKEQIKT
LNNKFASFIDKVRFLEQQNKVLETKWRFLQEQKRYKSNIEPMFEAYIYNLRRQLEVLAGDRAKLETELSN
MQETLEDFKEKYEEETSRRACAENEFVMLKKDVDCAYMNKAELEAKVESLIEEITFLKNLHEEEIAQLQA
SISDTSVIVQMDNSRALDMDDIIAEVKAQYEDIANRSRADAESWYYSKYEELRETAGKHSDKLRNTKNEI
MELSRVIQRLNGELENAKAQRGKLEAAIAEAEERGEMALKDAKCKLTDLEAALQKAKQDMACQLREYQEL
MNVKLALDIEIATYRKLLEGEEYRLGGEGVCPVNISVCRSQGGIVHDTNPCFGGGYTSSSRALFRSGGLV
SSGGMCSTSAGKSIVTGAGXXXPADGYSAGSGWSSNVKFVSSTTSYRAKY

>Alligator_K5L4
MAQVIKNFSTSSAGIPVGRTSFSSISVSRGGSGGLRQIGGGFGSRSLHSLGGSKRISISGGYHSARSAYG
YGLGGVGYRIGGAGYGFGGGFVPGSGGIQEVTVNQNLLTPLNLEIDPNIQRVRKEEKEQIKTLNNKFASF
IDKVRFLEQQNKVLETKWSLLQEHKTARSNIEPMFEAYISNLRRQVEVLGGERIRLDSELKGMQDVVEDF
RIRYEDEIHKRTAAENEFVVLKKDVDAAYMNKVELGAKVDGLTDEINFLRALYEAELAQMQSQISETSVV
LSMDNNRNLDLDSIIEEVKAQYEDIANRSRAEAESWYQSKYEELQLTAGRHGDDLRNTKVEITEMSRMIH
RLHSEIDSVKKQCASLQTAIADAEQRGELALKDARVKLAELEDALQKAKQDMARQLREYQELMNVKLALD
IEIATYRKLLEGEESRLAGEGVGAVNISVVSSSVGGSYGSGTALGLGTGASLGSGLSVGESGFGASSGRS
LVGGYSSGAGSSSSMKIVSKSMTKRSFRS

>Alligator_K5L3
MSRQSFVQIQSGRKGFSSNSAVIPSACRTSFSSRSVSQSGGCGASGGFRRVGGGGGGAGFGSRSLYNLGG
CKRISIAGSGGGFYGAPGPYGFGAGASSGFCYGGGVGGGFGGGVGGGLGLGGPGFGGGMGGPGFPVFPPG
GIHEVTVNKNLLQPLHLDIDPSIQRIRTEEKEQIQTLNNKFASFIDKVRFLEQQNKVLETKWMLLQEQGQ
KTVRNNIEPLFETYINNLRIQLNGLVGDRGRLEGELDNTQNLLEDFKNRYEEETNRRHDAENDFVTLKKD
VDGAFMNKVELQAKVDSLIDEINFLRAIYEAELSQMQTQITDTSVVLSMDNNRSLDMDSIIAEVKAQYED
IANRSRVEAESWYQVKYEELQATAGRHGDDLRNTKHEISEINRHIQRLRADIESVKRQCASLQAAIAEAE
ERGEMALKDARDKLAQLEDALQQAKADLARQLREYQELMNVKLALDIEIATYRKLLEGEESRLVGAEPVN
ISVVSSAVGTSYGGGSGLGLGGGSGLGIGGGSGFGLGGGSGMCVVGGSGLSMGGGLGIGGSGFSSSSGRG
SCGLNVGGGSGSSVKVIKTTSSRRSYKC

>Alligator_K5L2
MTTRTSVRSSMGGGIKGFSSGSVGLGSGFGGGGGRASFSSVSLSRVGGGRAGGFGGGAGGGFGSRSMYNV
GGSRKVSYGGGLRTGFGGGYGFGGGAGMGGGFGGGSGLALGGGGGGGAGFGMGGPGFGMGGPGFGYGGPG
FGGRGGPGFPICPPGGIHEVTVNQNLLTPLKLEIDPEIQRVRSQEREQIKTLNNKFASFIDKVRFLEQQN
KVLETKWTLLQEQGQTVTRKTLEPLFEAYINNLRRQLDSLLGDGPRLDSELRNMQDLVEDFKNKYEDEIN
RRTAAENEFVMLKKDVDNAYMNKVELQAKADSLADEINFLRALYDAELSQVHQQVSDTSVILSMDNNRSL
NLDSIISDVKAQYEDIANRSRAEAESWYQSKYEELQVSAGRHGEDLRSTKMEITEINRMIQRLRNEIDNV
KKQCANLQAAIAEAEERGEMALKDARAKLADLEEALQKAKADLARQLREYQELMNVKLALDIEIATYRKL
LEGEESRLAGEGVGAVSVSVVSSSTGMGYGGGSLGGGLGGGLGLGGGGGGGSGYGMGGGGSGYGMGGGGS
SYSMGGGFGMGGMGGRSATSMGGGGGYGSGGGMGLSMGGSFSGGSGRGLSSGGGSSSSMKIVSKTTTSKK
TIR

>Alligator_K5L1
MSRQSYSMRTGGGGKGFSTGSAIITSGNRPSFSSASVGRAGGGGGGFGRLGGGGAGGSGGFGTRSLYSLG
GTKRISLGVGSNLRSGFGAGAGGGLGFSSGGGGGVGFGGGAGGLFLSGGGGGGGYGGGYGFGPGGGLGFG
GGPGGGGGLVFGGGHSGGRGPAGFGSGTPCTGTIQEVTINHSLLTPLNLEIDPNIQQVRKEEKEQIKTLN
NKFASFIDKVRFLEQQNKVLETKWALLNDQGQKSSLSKNNMDPLFEAYISNLKKQLNSLLNERGRLDGEL
KNMQDLVEDFKNKYEDEINRRTTAENEFVVLKKDVDAAYMNKVELEAKVDALMDELNFLRALYDAELAQL
SAQMSDTSVILSMDNNRDLDLSSIITEVKAQYEDIANRSRAEAEAWYQTKFEELQATAGKHGDDLRNTKG
EISELTRLIQRIRAEIENARNQCANLQTAIADSEERGELALKDAKLKLAELEDALQKAKADMTRQLREYQ
ELMNVKLALDIEIATYRKLLEGEESRLSGDSVVPVSYSVVSSSPTVSSGGGLGGGYGGLGLGGGGGSGFG
LGGGGGFGYGSGGGLGLGGGSGGGGLGFGGGSGIGLGYGSGLSLGFGGGSSFGLGYGGGGGSGLSAGGGN
FSSGSAKGSGAGTAPGVKIVSKTSTSKKSIKSQSLKNLPQPE

>Alligator_HBS1
MSRQCFSSRGFSSSSAVCGFGRGNGSSASVGWSPGRGYGGFSSRSVCDLGRTARISFGGGYPSRGYPSGV
YGGFGIGRCGAYGGGAAYGGHMGLGNAVCFAGVGSHGGFGGHRGAFAGFGGPCRSEGIRGVNIHPELLKP
LAVGLDPAECQVRTHEKEQIKNLNNKFACFIDKVRLLEQQNKVLTTKWDLLQQCIVPGTRRNLEPLYESF
ICNLKKQLEHLLCDRDKLMCEEKAANHLVDEFKCKYEEQINRRTAAENEFVVLKKDVDCLHLTKEELEIR
VALLRQQLEFLTCIFAEERAQMDCQLCDTSVIVEMDNNRGLDMDSIINSVKCCYEEIAHKSKAEVEAFYQ
TRLEELHTNRGKYCDDLRISQCEIADLKRGIQKLQGELDGVKKQIGCLETAICDAEHHGDCTLKDAREKH
INLQTALQQAKDKLACLLRDYQELLNVKLALDIEIATYRSLLEGEESRICTGTPACISVVSGGCTIGDNL
AVGVGRGGAARGEVGSGGGVGSMAGGGGFSSRSGECITRMGGRLGSRIAVAGFGKGGPVPCEGVFMGAGG
MGSMGNVVHDEVEHFGPGGLGGMGGGACNAGMRASSTMHVMSSGTMPR

>Alligator_HBS2
MSRPGRLTRSINRQRGFSSASDVCGVERRGAYKASPCPPVGRCEVGDYSSNSACTLGGMRRLPHGTGYLS

EGYGAGGHRWAGYRGVDSGRFGSVSSRVDVCGHRGYGTVQGYGTLGGYTACKRDGIQRVCINKSLLTPLC
MGVDPQEHQVRSQEKEQMKCLNNQFACFIDKVQCLEQQNKALETKWNLLQQHTMPAARKTLEAYFENYIC
KLKKQLECLLGEREQLSNKECVAKKLVDEFRCKYEEVINKRMTAEHGFVLAKKDVDYAFLNTEEQEVKVD
LLKCQLELLNHVFTEERAQMDCQLCDTSVMVKMDNRRDLDMETIIKNVECCYKEIAQKSKEEVDTLYQTR
FQELQEQRGRFCDDLESNKREIAQLTQLIQELQCELDNVKKQVGCLQTDIRDAEQHGDFTLQDGRAKHVE
LQNALQKAKDELASMLRDYQELLNVKLALDIEIAMYKTLLEGEESRICSGTPVTVAVVTSSCDIAEHFES
AAGIGGPRRGLSSRSTGVQPRRRASSVVRPRVQEGVGCFPSAGLSSRSDGFSSQSWGYQSRTVASTAGGP
GFSGGVACCPKGGPVPKKGEVNSHIAVCQPGSTANFGPGGCVMRHLGSSPIVGEACPGVGGCDTGVVKP

>Alligator_HBS3
MSRQLFAGKSFYRPKGFTSASAVCGVGRHRHSGPSVCRPFGRCGAGGYSSRSLQNLGGSKNIACGGGYQS
GVYGGFGVDYCMGYGGWRCGDACYSNEGFGGVSFGGKLGLCGPRGHGALGNYGGSCRSEGIYRVRVNEDL
LQPLCVGVDPEIQQIRAQEREQIKKLNNQFACFIDKVRCLEQQNKVLGTKWNLLQEQVLPCRKNLQHLFE
NYLCSLQRQLDCLLSERGQLEPELQNGQKLVEELKCKYEQEINRRTASENEFILLKKDVDCSYMSKVELE
AKVDTLRQEIEFLRCVYAQELVQMEGNLCDTSVVVKMDNNRDLDMEGIIREIECWYEEIAQKSKAEVDAL
YRTRFQELQETKGRYCNDLKCNHHEITELSRVVQRLQHNLDNAKKEVACLQTSICETEQRGDCALKDAQG
KYGELQNALQKAKDELASMLRDYQELLNIKLALDIEIAMYKTLLEGEENRICTGSPVRWSPLLAMSSVIV
GMEMALEQDVATPLYIKGAALDMEDKAQ

>Alligator_K78LT
MSQLSPSCKSGDGGKDFSSCSAHCGSVGVGSSSSSICQSGESRGGRGYFGYGYGSKSLHNLGGTRRISVC
DGYQGGGRGYGVCWERYGGWGYGRGGFGGGNFGGGGARFGGICRGGLVGGGLGGGDIGRSWGIQGVHVNG
NLLRPVHMEIDPDFQRVRAHEKEQIKSLNDKFACFIDKVRCLEQENQVLLTKWELLQQQGGQTGVHRDIE
PFFQSYICHLRRQRDTLQSQKEELNLEDCKMLQIVNEYKASYEKEIDRRLCTEEEFVNLKKNLDCIYMGK
MEVGVKVNILREEIEFLKCLYAMELSDVQRIAQDTNVVVSMDNCRDLDVEGIIAEIRREYEGLAQKSQAE
VNAVYQSKYQDLHNTWGRHCDRLRTRQHEIQELTRLIQKLRLDIGDVKKKNACLQEEIADVERRGCCAVS
DAQEKLRELRCSLLKAKDNLAHLLRDYQELLNVKMALDIEIATYRALLEGEECRITEGLPGPIPVINHGT
WVSGSAGGRYGGFPWCWGGYGGFRGGHCGWGGGRNWGIVGGHCSIRRGFSSRSGGHYSRNIRCSPPNYGG
SKGGGFSSRSEGGNVKVCISRGGEPSFGGSCSGGVGGIFGMGGSCESGGGGHGSVGGSQAGGGGFISGGE
CSFGDGRYGYGKSDCCVVGGYGSGVGGHSGVAVSSKWDGGSSGCEGYSSGGTRFGSRRGGRSSGRNCCVF
GGYGSRRTGTSGIQISSGWTGGRAEGGECTARDGVSGSGGSGWSRGRDCCVIGGDGPGAGVSDGYSSGAG
GYSSGVGYGCGGREYGSGGAIISGEGSSSGASVSCSTGPYGPAGWDHAGGGGCVSGGGGYDSGVTVIPIP
VGGSSSGGVGSCGCGGYGSGAGGSLSGERYSFGGRGYGCGVSSISGGEGSCGGDASIGGGHSGSVGYISG
ARGHSSVNIGSDECSTGCGSGGGAYSAGAGASSGMERCSSGVGGSSGGVVDICGSGVHSSRVVGSSSGAG
GSSSGGGCSGGSGGHTSGSTNTPGAIGHCSSGSSGSGGGSRFGSGGYGSGGGGGSGERGYSSSRIGGCAS
STYSPGAGGSSGGGGLRSGGIGRTGGSSGGIGPGGYGSGAGGSFSGGGYSCGSGGYGSIRGSYSGSGGYG
SGTGGYGSGAGGSPGGRGYSCGSGGYSSGAGGSTSGGVCSSGGGGYVSGSGEYDSGAGGLSSGDVCSTSG
GGYGSGGGGSGGYDSGAGRLSCGDVVDTSGGYGSGGGGSGGYDSGAGRLSSGDVCSISVGGYDSGVGGSG
GYGSGSGAYDSGAGRLSSGDVCRISGGGYGSGGGDSGEYGSGSGADDSGAGRLISGDVCRISGRGYNSGA
ERSEEYSSGNGAYDSEAGRLSCGDVCSISGRSPGADGFCSGDACSISGGGYGSGGGGSGGYGSGSGEHDS
ETGRLSSGDVCSIGAGGYGSGGGGSGGYGSRSGGYSSGAVGSSSVRVCSISGGGYSSGAGGSSSRGERSI
SGGGDDSGICGGGGYSGGAQGWG

>Alligator-K78L6
MFQQFSSRSGGGGRSFSVRSAAATVVPGGNRGSFSSASLSRIGRGNVGLGGGGFGGGFSSRSLHGLGGSR
GIPISFGGGSIHGGGLGFGGGGFGGVGFGGGGFGLGGGLGFGGGGVVPGRYGRWAGSVGGGLGVGGPGMG
GFGGGLACPLPPPGGIQKVTVNPNLLAPLKLEIDSEVQRVRTHEREQIKTLNNKFASFIDKVRFLEQQNK
VLETKWDLLQGTTTTRVSLEPMFEAYINNLQCQLENLLGNRGQLEGELRNLQDLVEDFKKKYEEEINRRT
AIENEFVMSKKDADHSFMNKVELQVKVDSLIDEISFLRAFFKAELDQMQGQITDTNVILQMDNNRDLDLN
SIISEVKAQYEDIANRSRAEAEAWYQSKFQELQTTAMTHGDDLKNTRNEISELTRIIQRLKAEIDNVKKQ
IAGLQTAIADAEQRGEMALKDARAKLSDLQNALQTAKDELAHLLRDYQELMNVKLALDIEIATYRTLLEG
EEMSGEYVDPVSISVVSNTTSLAGGGGGVGGGFSLGGGRGSGLGLGLGGGGGGGGIGGGVGGIGLGLGV
GGGGGSSFGHGIGSGSGISVGGGGGNGVSVGGGSFGLGGGSSSSIKYVSSSSSSRKMVR

>Alligator_K78L5
MALQLRTKSIGGSRSGYSSSSVMAGGGGGGNRVRSSYSSHGGGGQCGGFSSRSYGGGGRISSGSYGGGFG
CRGGGFSGGMIGGGGGGYGGGMGCIGGGLGGMGGGMGGGIGGGMGGGMGGGMGGGMGGGMGGGMGGGCFG
GGGFSGGPGMGPMIGGGPGMGPVIGGGPMGGFGGGIGGGGFGGPGRGDPGFPRGIQPVQVDPSLLKPVQV
EIDPQIQQVKQNEKEQIKTLNNQFATFIDKVRFLEQQNKVLSTKWELLQQQGHSGPRKDLSSLFESYIQN
LKRQLDSILHQREQLESDLQNMQPHVEEYKKKYEEEINRRATAENEFVVLKKDVDNAYMNKVQLEAKVQA
LMDEIDFLRHVFEEELSQMHTISRDLSVVVSMDNTRHLDMDSIIDEVRRQYEDIARSSRAEAEEWYQSRY
EELQNTAGRHGDNLRNTKQEIQELTRCIQRLRSEIENVKKQVRALQAAIAEAEDRGEMALKDARSKLQEL
EAALQKDKEELARLLKEYQELLNIKIALDIEIAMYRKLLEGEEGRLSQDMSNVNVSYKV

>Alligator_K78L4
MGGGIGGGMGGGMGGGGFGGSGFGGGGFSGGGMGPMIGGGPGMGPMIGGGPMDGFGGPGGGGPGFPRGIQ
PVQVNPSLLKPVQVEIDPQIQQVKQNEKEQIKTLNNQFATFIDKVRFLEQQNKVLSTKWELLQQQGHSGP
SKDLSSLFEIYIQNLKRQLDSILDERGQLVSELQKMQGHLEDYKTKYEEEINRRTTAENEFVVLKKDVDN
AYMSKVELEAKVQALTDEINFLRYVYEEEMSQMHTISRDLSVVVSMDNTRHLDMDSIIDEVRRQYEDIAR
SSRAEAEEWYQSRYEELQNTAGRHGDNLRNTKQEIQELTRCIQRLRSEIENVKKQHQQLQAAIAEAENRG
EMALKDARSKLQELEAALQKDKEELARLLKEYQELLNIKIALDIEIAMYRKLLEGEECRLSQDLSNVNVS
VVGRTTITGGHGGSGGGFGGGSGFGGGSGFGGGSGFGGGSGFGGGSGYGGGCGIGGGIGGGGIGGGGMGG
GGYGGSCVSGGGMISGGYSSGSGRGFSSGGGSIGGGSSSVRRSNTTTTIKSSGGRC

>Alligator_K78L3
MALQSCTKSIGGSKRGYSSCSAIGGGGGGGSRVRSSYSSYSSRGYGGGGRCGGFSSRSYGGGGGGRISSG
FYGGGYGCRGVGFGGGAMGYGGAGGYGGGGSCIVGGLGGYGGGMGGGFGGDGFGGGGMGPVMGSGPVGGF
GGGSRGFGGFGGPGSGGPGFPRGIQPVQVDPSLLQPVHVEIDPQIQQVKQHEKEQIKTLNNKFATFIDKV

RFLEQQNKVLSTKWELLQQQGHSGPRKDLSSLFENYIQNLRRQLDSIHGQRGQLESELQNMQNYVEDYKN
KYEDEINRRTAAENEFVVLKKDVDCAYMTKVDLEAKVQALIDEINFLRYVYEEELSQMQTISRDLSVVVS
MDNSRHLDMDSMIDEVRRQYEDIARSSRAEAEAWYQSRYEELQTTAGRHGDNLRNTKQEIQELTRCIQRL
RSEIENVKKQXXX

>Alligator_K78L2
MALQTYTKSIGGGRKCYSSRSAVGGGGGSRVRSSYSSYSSRGYGGGGRCGGFSSRSYGGGGGGRISSGFY
GGGYGCRGVGFGGGAMGYGGAGGYGGGGSCIVGGLGGYGGGMGGGFGGGGFGGGGFGGGGFGGGGMGPVC
GFGGGGPGVGFPLGRGGPGYPGGIQPVQVDPNLMRPVHVEIDPQIQQVKQHEKEQIKTLNNKFATFIDKV
RFLEQQNKVLSTKWELLQQQGPSGPRRDLTALYENYIENLRRQLDNVLSQRGPLESQLHDMQGYVEDYKN
KYEEEINRRTSAENEFVVLKKDVDCAYMTKVDLEAKVQALIDEINFLRYVFEEELSQLQTISRDLSVVVS
MDNSRHLNLDSIIDEIRAQYEQIAQSSRAEAEAWYQSRYEELQSTAGQHGESLRNTRQEIQELTRCIQKL
RSEIENVRKQCEQLQAAIAEAEERGEMALKDARCKLEELECALHKDKEELARLLKEYQELLNVKIALDIE
IAMYRKLLEGEECRMSEGSNVNVSVVGRTTISGGRGGFGGGFGGGMGGGIGGGIGGGIGGGIGGGGGIGG
PCGMGGPIGIGGPIGIGGPCGPGGAGGMGGPCGIGGGGISGGSMVGGGYGGSCIGGGGMVSGGYSSGSGR
GFSCGGGSIGGGSASSIKKCITTTTVKSSGGRY

>Alligator_K78L1
MSRQSCGLNFAGGSRGFSSSSICLGGGNKVSFSAASRGGCGGSVRNPIGFGSRSLYNLGGCKRISIGGFG
GNSGACGAFGAGGRGGGGFGYGGLGGGFGGGSGGGFGGGFSDGSGGMFNGWGGPSFPVCPPGGIQEVTVN
QNLLIPLKLEIDPEIQKVRSQEREQMKTLNDKFASFIDKVRFLEQQNKVLETKWKLLQEQGQGTGPHSRS
LDQLYEVYINSLRKQLDGLTSEKSQLQLELKNCEDLVEDFKTKYDEEINKRTAAENDFVLLKKDVDAAYM
NKVELQAKLDSLADELNFLRCVYDAELSQMQQTVSDTSVVLSMDNNRNLDLDSIIAEVKAQYEEIAQKSR
AEAEAWYQCKYQELQVTAGKHGDSLKDTKAEISEMNRMIQRLRAEIENVKKQCENLQTSVADAEQRGELA
LKDARVKLSELEAALQKAKTELTRQLRDYQELMNVKLALDIEIATYRKLLEGEECRMSGDCQSAVSISVM
GSNSTSGGSGGGGFGMGFGSGSGRGICNLGGGGAAFGGGLCSKHSTDGGSFLKTLSELTLDFKCCSSGRS
QKVLIFRRTI

>Alligator_K8L
MSFFPTSKGQASYPSFSSFTYTSPSTRRITNPISVMMRKKDHGNLGSGYKRFSWDDQAIVKINQNLLDPI
ESGLDPTYHQEKLKEKEELKTLNNQFVHLIERVCSLEQHKKVLETKCSILKSQKATPARMENLLKTYISQ
LKKHLADQGQMKGKLHLELHEIQDIMDENKQKYEEEFNLRNEKENDFLLMKKEMDEAYISQNELEGKLDR
LRDELEFLKNFYDQEMHELQTEVHMYKESVVISMPSQPSLEVDSIVSEAQSQYEKMAERSCKEAEKAYRD
RHNKLKDSMGRHGEELQTMKTEINDLTRKSQHIQLEIQNLKEQCNKLKEDIANADDRYKFPIEEAKNKLH
MLENAIQKARQDMAHHLREYQELLNTKLGLDIEIATYRKLLEGEEGRLESGMQNLSIHHKFHHHHGQGVA
LREISTLGSSSHPSGASPLESLSEERKPEVEN

>Alligator_K8
MSVSGSRQSFKSSSYRGAPRTFSSRSFTSNFTRASPPVASNARVDARGLRGPAMGLGAGGGITAVSINQS
LLAPLNLEIDPNMQQVRTQEKEQIKSLNNKFASFIDKVRFLEQQNQVLETKWSLLQGQRTTRSNMTGMFE
AYITNLRRQLEGLDQDKLRLESELGNVQGLVEEFRSKYEDEINHRTEKENEFVLLKKDVDEAYMNKVELE
SRLEGLTDEISFLRQLYEEELHQLQSQVSNTSVVLSMDNNRSLDMDSIIADVRNQYEDIANQSRAEAEGM
YKIKYEELRNTASKHGDDLRNTKAEISELTRTIQRMQAEIDAVKGQRANLEAAIAEAEERGEMAVRDARG
KLAELEAALQKAKQDMARQLREYQELMNVKLALDIEIATYRKLLEGEESRLESGMQNLSIQTKTTNYSAG
LGRAFGGYNSGYGSSLSSSIYSVNSPLESAAVTKSKAIVIKKIETRDGKLVSESSDILSN

>Turtle_K80
MTSRPNEFSSSSLSSWQASWNQNRGSSVVTSSEYDGCSSPGGPASRSFSTSSLGGYTSSSCPSVFINKKL
LTPLRLDLDPTFQERKKQEKDEMKILNDQFASLIGKVQCLEQQNKVLTIRWSFLKDQDNSRSELDIKLLY
DQYMSKLKQEMRAVGHEREQLESELTEVLKSMDNIRSRYEDEIHKRSGLEFTFVELKRDLDAGSMHRTEL
EVKLQGLQELMELKKAVYEQELQELLAEIKDISIVMGIDNRCNLDLSRIVEEVRAQYEALAFRSWEEAEA
LTWSKLNEGATHSATYRDQLFSGRREIAELTIHIQKLRTCIVSLKSQCLRLEEDIKEAGGNGQLALRNAN
TKLAELEQALHKGKADLVGLVKEYHELMNIKLALDVEILTYRKLVEGEESSMESPTCPVISSIHSSPRHF
SSISSFSGSSQLASRARGNSTDGMTRNGVVAEAVLARSQSGGSANRDADERPSGARPKHRLLSDEEHCRE
ESIPAE

>Turtle_K7
MSRSVSFSSRSAAPPGSFRQVRVSNVSSTRGLGGGAGLGRAGGFGSSSLYSLGSANKRISLGGGGSSYGV
QSGYGLGAGYGAGAGGGFAGAGIQEVTINPNLLVPLNVQIDPTLQTVRKEEKEQIKTLNNKFASFIDKVR
FLEQQNKVLETKWSLLQDQKTTRSNIAPMFEAYINNLRKQLDGWLNDKGRLEGELKNMQDLVEDFRNKYE
DEINKRTAAENEFVILKKDVDASYMNKVELEAKVDALTDEINFLRSLYEAELRELQAQVSDTSVVLTMDN
NRNLDMDSIIAEVKAQYEDMAKKSRMEAESWYQTKFETLQATAGKHGDELRNTKTEITEITRMIHRLQAE
IDNVKSQRAKLEAAIAEAEERGERAIKDAMAKQEELEAALNKAKQDMARQLREYQELMNVKLALDIEIAT
YRKLLEGEESRLAGDHAGNVSISVLNSSAGGGYSGAGGYGFGGGIGLGSGMGSGGLSFSSGGSPGGMKAY
SLRTTSSSRRSDRN

>Turtle_K84L
MSCRSYRISSGGGGVRNFSSCSAVIPRSIRQYSFSTVSSRGGSGVGYRGLGYFGSRSLSGVASSKPRMAV
GSCRSARYGYGNAGLPYGYGGAGFGYRVGGICGPCPPTITPVQVNPHLLQPLNLEIDPNVQSVKYQEKEQ
IKCLNNKFASFIDKVRFLEQQNKVLETKWSFLREQKHYQNNMEPMFEAYISNLKRQLENLGCDRAKLETE
LHNMQDILEDYKRKYEEECNHRTCAENEFVVLKKDVDCAYMNKAELEVKVESLIDEITFLRRIYEEEIAC
LQASILDTSVIVQMDNNRGLNMDDIIADVKAQYEDIANRSRAEAESWYQCKYEELKVTASKHCDNLHSTR
NEITELNRVIQRLHGELENVKAQRCKLEGAIAEAEERGEMALKDAKCKLAELEAALTKAKQDLACQLREY
QELMNVKLALDIEIATYRKLLEGEESRLCGEGVCPVNISVCRSQGGIVCDGDPCFGGSYSSSSRTLLKTG
GVLSSGGICDARIGRSIVANLGDVCAPCVPTGGYSTSSGRSSNVKCVSTTSSYRTKY

>Turtle_K5L4

MAQKTISIRSGGATRNFSASSAIIPGSRTSFSSISVSRGGGGGLGRVCGGGFGSRSFHGLGGSKRISVSG
GYRAVRSGYGYGLGYGGAGYRIGGAGYGFGGGFAPGAGGIHEVTVNQNLLAPLNLEIDHTIQKVRKEEKE
QIKTLNNKFASFIDKVRFLEQQNKVLETKWCLLQEQKTAKSNIEPMFEAYINNLRRQLEALGGERLRLDG
ELKNMQDTVEDFRIRYEDEINKRTAAENEFVVLKKDVDIAYMNKVELEAKVDALTDEVNFLRALYEAELA
HLQSQISDTSVVLSMDNSRNLDLDGIIAEVKAQYEDIANRSRAEAESWYQTKYEELQLTAGRHGDDLRNT
KIEISEMNRMIQRLRSEIDNVKKQCASLQTAIADAEQRGELALKDAQAKLAELEEALQKAKADMARQLRE
YQELMNVKLALDIEIATYRKLLEGEESRLAGEGVGAVNISVVTSAGGSSYGTGGGMGLGVGLGNASGLGY
GIGGSSFSASSGRGLVGGFSTGGGSSSSMKIVSKTTSTKKSIRS

>Turtle_K5L3
MSRQSTIRFQSRKQGFSASSAIIPNSCRTSYSSRSVSRAGGCGAGAGAGRVVGGGSCFGSRSLYNLGGSK
RISMAGSSGGFLCGAPGPVGFAGGAGCGFGFGGGAGAGYGLAGPGFGGGRVGPGFPVCPPGGIQEVTINQ
SLLTPLNLEIDPTIQRVRKEEKEQIKTLNNQFASFIDKVRFLEQQNRVLETKWSLLQEQGQKTVKNNIEP
FFENYIGNLRRQLNNLMNERGRLEGEVNNMQNVVEDFKNRYEDEINKRTTAENDFVTLKKDVDSAYMNKV
ELVAKVDSLTDEINFLRALYEEELAQMQTQVSNTSVVLSMDNNRDLDLDGIIAEVKAQYEEIANRSRAEA
ESWYQTKYEELQVTAGRHGDDLRNTKHEISELTRIVQRLRNEIDNVKRQCANLQAAIAEAEERGELALKD
AQGKLAELEDALQKAKGDLARQLREYQELMNVKLALDIEIATYRKLLEGEESRLCGEGVGAVNISVVSST
VGGGYGSRSGLGMGSGSGVCIGGGSGLGMGGGSGVCVGGGSGLGMGGGLCVGGSNFSSGSGRSIGGLSLG
GGSGSSVKFVSTTSSTKRSYKS

>Turtle_K5L2
MSRISMRKSTSGGGSKTFSSGSAGLGGGFGGGGGGGRSSFSSVSVSRVGGGRAGGFGGGGFGSRSLYSLG
GNKRVSYSTGGGGLRSGFGGGGGYGFGGGSAIGFGYGGGAGGGFALGGGGGYGLGGAGGGYGLGGPGFGG
RGGPGFPVCPPGGIHEVTVNQSLLAPLHLDIDPEVQKVKVQEREQIKTLNNKFASFIDKVRFLEQQNKVL
ETKWTLLQEQGQTTVKRSLEPLFDAYINNLRRQLDNLLGERGRLDSELRNMQDMVEDFKNKYEDEINRRT
AAENDFVVLKKDVDGSYMNKVELQAKADALADEINFLRALYEAELSQMQQQVSDTSVVLSMDNNRSLDLD
SIIREVKAQYEEIANRSRAEAESWYQTKFEELQISVGRHGDDLRNTKVEISEINRMIHRLRNEIDNVKKQ
CASLQAAIAEAEERGEMALKDARAKLAELEDALQKAKADLARQLREYQELMNVKLALDIEIATYRKLLEG
EESRLAGEGVGPVSISVVSSSTGYSGGGSLGGGYGGGLSLGGGGGGGGYSLGMGGGSGFGLGGGGGGGGG
GYGYGSGMSIGGGSGFSSGSGRGLSGSLSSGGRSGSSVKIVSKTTTSKKSIR

>Turtle_K5L1
MSWQSGSIRSGGGSKGFSTVSAVVPSSHRASFSSVSVSRGSGGFGRLSGGGSFGSRSLYNLGGTKKISIG
GGSNLRSGFVGGAGGGFYLGGGGHGGGSSFGGGAGGGGLSIGGGAAGSGFGFGGSGLGFGGGGGFGGGRS
PAGFGGGTLPGMGTIQEVTVNQSLLAPLNLEIDPNIQQVRKEEKEQIKSLNNKFASFIDKRFLEQQNKVL
ETKWTLLQDQGQKSNSNKNNLEPLFESYINNLRKQLNHLLNERGRMEGELKNTQDLVEDFKNKYEDEINR
RTAAENEFVVLKKDVDAAYMNKIDLEAKVDALMDEINFLRTLYDAELAQLNVQVSDTSVILSMDNNRDLD
LSSIVNEVKAQYEDIANRSRAEAESWYQNKFEELRATAGKHGDDLRNTKGEISELNRLTQRLRAEIENAR
NQCASLQTAIADSEDRGELALKDARMKVADLEDALQKAKQDMTRQLREYQELMNVKLALDIEIATYRKLL
EGEESRLSGEGVIPVSYSVVSSSAGVGGGAGLGGGFGGFSAAGGGGGFGYGGGSGLGLGGGGFGFGSGGG
GFGGGSSFGYGGGSGLSAGGGNFSTGSGKGTGVGTSVGVKIVSKTSSSKKTIKS

>Turtle_HBS1
MNRLCFSSGSLGGRRCFSSASAVCGLSGGRTSSGSVSWPAGRSESGGFGSTSLFELGRSQRISYGGGYGL
GHCGAYGGGGYGCGAGYGSGAGFGSHFGVGSGAGYGSCVGYGSGGYRGSSGFGGYSPYHFGSGVRYGVGH
AGRSDGIQAVCIHPELLKPLSVGVDPEEHKIRSHEKEQIKCLNDQFACFIDKVRCLEQQNKVLATKWDLL
QQHAVPAKRDLLPVFENYICQLKKQLECLLHDRARLEDEEKAVQDLVHEYKSKYEGEINKRTHAENEFVV
LKKDVDCVFMTKEELEAKLLQLRQHFEFLKCIFIEERAQLDGQLCDTAVVLEMDNSRDLDLSGIIHSVKC
CYEEIAQKSKGEAETFYQTRLEELSTNRGRFCDDLKNIQGEIAELKRLIHKLQGETDKVKKEIACLQTAI
ADAEQRGDCALKDAREKHIDLQNALQKAKDKLACMLRDYHELLNIKLALDIEIATYRTMLEGEETRICTG
NPTNVAVVSGGHTIGDCRAGFGSVCGPLKGGFNHGIGIFASSDGFSSRSAEGISRMGGGYGTRSAVGSSG
RGYSSGGLGSGGIGYSLGGFSSGSGGYSYRRVLSSDFASSGGVGGHVGGVGSSGSAGAGGDVGYHSSGGV
SSVGGVCSSGDVASSGDAVGSSGGASGIHSSVEHFSSGGVGSSGGGVYSSGGVGSSGGGVYSSGGVGSSG
GGVYSSGGGSGGAYSSGTRMVSVSSSSRRIIR

>Turtle_HBS2
MNRQTFFASSTVGRKGFSSTSDVLSGHRTCVASVGPPVERCKVGGYSSRSVCHLGGSTRIADVVGCPSGA
GCYGGYGDGGTAWGSVGYGSSGGFSGRVGLCGPRGYGTFGGYADCRSDGIRGVSVNESLLKPLCVGVDPQ
EHQAREHEREQMKTLNNQFACFIDKVRHLEQQNKVLETKWNLLQQCVPPAPQKNLEQCFENYICSLRKQL
EDLLSDKEQLTCEESASRKLVDEFKCKYEEEISRRTAAETEFVVLKKEADGTLLHKEELEVNTTLSKQKL
EFLRCVFDEERAQMDGQFCDTAVIVKMDNSRDLDTDCIIKNVEGWYKEIAQRSKEEVDVLYQTRFEELQD
QRGRFCEDLERNKHEIAELTRLIQKLQGETDHVKKQIACLQTAICDAEQRGDCALKDARGKHMELQNALQ
RAKDELACMLRDYQALLNVKLALDIEITTYKKLLEGEESRICLGTPVSVSVVTNSSNIAGDSGTVVGVGS
GCGYGSLSRGYSSGHGRCGSPGGGFSSKSLGVCPRSVVSVAGSQKFPEGADFYPPGGFSSRSGSCISRGV
VSSVCSHHLPGGVHWCPPGGVVSGGGGYGSKVVTSSVGTGQVSGGVACSPDGGGYITSSGYPGNGVCTVE
TGGFIIQYLGSSPAGGPSTGAVDNSGAGPCHTGVPGSCEVVRETGIAL

>Turtle_K78L4
MNRKFSSKSGSGARGFSGRSATTTITSSGNRAGFSSASLSRVGRRSGGYSGGFGSRSLHSLGGNKSTSIR
IGGGGFRSLGLGFGGGGGFGGGGFGGAGCGGGFGGSSGAGPVLSPGGIQQVTVNPNLLAPLNIEIDPEVQ
RVKVQEREQIKTLNNKFASFIDKVRFLEQQNKVLETKWDLLQQQGTTITKIPIDPLFEAYINNLRRRLDS
LVTDRGRLDGELRNMQDLVEDFRKKFEEEIHRRTSAENEFVVLKKEVDAAYMKKMELQAKVDSLIDEINL
LRAVYEAELAQMQGQIKDTNVILQMDNNRDLDLNSIIAEVKAQYEDIANRSRAEAEAWYQSRYQELQVTA
TTHGEDLKVTRNEISEVSRVIQRLKAEIDSVKKQIAGLQTAIADAEQRGELALKDARGKLADLQNALQTT
KDELARLLRDYQELMNVKLSLDIEIATYRKLLEGEECRMSGEYADPVSISVVSSTTSVAGSGGGVGSGYV
LGGGGGGRGSGLSLGGGGGGLGLGIGSGSGFGIGGKSGISVGGSGIGLGGSSFGYGGGVTSGGGSSSSIK
YVSTSSSSSKKLIR

>Turtle_K78L3
MSRQLNARAGGCSRGFGSVSAIISNGGRSSCTSLSRGRSRHCVFGSRSLYNLGGIKSVSNSLIGGGWRLG
GGFGGGFGTSGGAGGFGGGGLFSGGVGGGRSSPGFSICPPGGIQEVTINQNLLVPLNLEIDPEIQKVRVQ
EREQIKTLNNKFASFIDKVRFLEQQNKVLETKWNLLQQQPTSNAGNNLEPAFEAYVGSLRKHLDSLVGER
GRQDSELKNMQDLVEDLKHKYEEEINRRMAAENEFVLLKKDLDAAYMKKLEMEAKVAAMTDEINFLRALY
ETELAQMQRQISDTSVILSMDNNRALDLDGIIAEVKAHYEDMANRSRAEANSMYQGKFQELQVTAGKHDN
DLKNSKLEISELTRLIHRLQAELENVKKQCAKLQSAVAETEGRGELTLKDAREKLVELEMALQKAKEETA
RLLRDYQELLNIKLALDIEIATYKTLLEGEECRMSGECASAVNISVVSSSSGGGSGISWGGGGGGGLSVG
GGGSSSGSGRGIGGYSYGGRGGSSCVKIISTTSSSKRTTIC

>Turtle_K78L2
MYLQPCTPSAGGSRRSYSSCSAIGAGGGGGGRVRSSYSSYSSRGFGGGGRCAGFGSRSLHGVGGGARISY
GGGFGCRGGGFGGGYGGGAGGYGGGAGGFGGGPCGFGGGAGGFGGGAGGFGGGAGGFGGGMGFGGPGFGP
GFGPGRPGQPGGIQPVHVDPSLLQPVNVEIDPHIQQVKTQEKEQIKVLNNQFAVFIDKVRFLEQQNKVLV
TKWELLQQQGGTIIKKDLAPLFENFIQSLRRRLDGLQNQKGQLQSELENMQQYVEEYKHKYEDEINKRTA
AENEFVVLKKDVDNAYMVKVELETRVQGLTDEINFLRFIFEEELSQLQTISRDLSVVVSMDNNRHLDLDG
IIQEVRNQYEIIAQRSRAEAEAWYQSRYEELQSTAGRHGDNLRNTKTEIQELTRSIQRMRAEIESVKKQI
ANLQSAIAEAEEKGELALKDARAKLDDLEHALQKDKADLANLLKEYQDLLNVKLALDIEIAMYRKLLEGE
ESRIAGGGHVNVSVIGGGGSGGVIGGGFGGGSGGGICGGGFGGGNIGFGGGSGGGICGGGFGGGGGFSGG
SGVVVCGGGYGGRTVGGGGFSSGSGRGYGGGGSGICSSGGSSVTRRCTTSFSTKSGSGGY

>Turtle_K78L1
MSRQFSGQSLGSSIRGFSSSSACFGGGNKVSFSTVSRGGCAGSACSPRGFGSRSLHNLGGNKRISIGGFG
GTCGGFGSGGQAGGGFGGGAGSGGQFSGGLGGCFGGGAGGGFGGGWDGPGFPVCPPGGIQEVTINQNLLT
PLNLKIDPEIQKVRKEEREQMKTLNNKFVSFIDKVRFLEQQNKVLETKWKLLQEQGQGPIKKSLDQLFEA
YSNGLRKQLDGLLNERRQLDTERKNFQDLVEDFKHKYEEEINKRTVVENDFVVLKRDLDVAYMSKVELQV
KLDSLTDEINFLRCLYEAELSQMQQTASDTSVVLSMDNNRNLDLDSIIREVKAQYEEIAQRSRAEAESCY
QCKFEELQVTAGKHGDSLRDSKSEISELNRMIQRLQAEIENVKKQCERLQTSIADAEQQGELVLKDAKGK
LAELEGALQKAKADLARQLREYQELMNVKLALDIEIATYRKLLEGEECRMSGECQSAVSISMAGGSSTSG
GGLGGGIYLGGGGGSGGRIGFSSGSGRGGYALGGGSVSGGGYSAGKGGYGSGGGSSTIAKTTVTSTSVKS
TSRRV

>Turtle_K8L
MSVISVNKKYRSSPHNFSSQSYAFTGHSRTSPVTYAISSPGGSKFRGARSPLGLGMSGGVGIEGTLAAVN
FNRSLMSPIGIGLDPEIQRVKAEEKEQIKTLNNKFAHFINKVRTLEQENQVLKTKWNILQGQKTGPSNME
KMLKAYINSLQKQLEDLGQQRVKLESDQGNMQDLVEEFKRKYEDEINNRSERENQFVLTKKDVDEAYIKK
QELEVKLQSLTDEINFLMELYAKELRELQADVEMSNNPVVISVDSSPKLDVDSIIAEARVQYEKMAENSR
EEAENKYRITYEQIQEKAGQHGEELRAAKAEINELNWMIQRIRAEIAALKEQNAKLEANIVQAEEQGKLA
VADATGKLQKMEHALQKAKEDMVVQLREYQALMNLKLALDIEIATYRKLLEGEESRLDSSMQNLSILTKT
TGYTSGMSTRFPGKFGSS

>Turtle_K8
MSLRITQKNYRSTSSVPTRAFSSRSFTSGPATSTRMSSSYSSYGGSRYGGGSIRGPSMGYGLGAGGGITA
VSVNPNLLAPLNLEIDPTLQHVRTQEKEQIKTLNNKFASFIDKVRFLEQQNKMLETKWDLLQGQKTTRSN
MNNLFEAYINNLRRQLDGLGQEKLKLEAELGNMQGLVEDFKNKYEDEINHRTEKENEFVLLKKDVDEAYM
NKVELESRLESLTDEINFLRQLYDEELRELQSQVSDTSVVLTMDNNRKLDLDGIIAEVRAQYEEMANRSR
AEAENMYKTKYEEIQAVAGKHGDDLRSTKTEINELNRMIHRLQSEIDALKGQRASLEAAITEAEERGEMA
IKDASAKLAELEGALQRAKQDMARQLREYQELMNVKLALDIEIATYRKLLEGEESRLESGMQSMSIQTKT
TGYSGGLGSAYGGYGGGYGSSYAGGSYSLKSPTLESSPLSRSKAIVIKKIETRDGRMVSETSDVLSN

>Lizard_K80
MTSRSRDFSRLSSRSLTSPGASYNVRRGRSCSPSSRYGACCNPTRGEPARRNFSSSSLTGYGHHAYPKVT
INQRLLTPLHLEIDPAFQAIRNKEKEEIKTLNNQFASLIGKVQNLEQHNQVLLTRWNFLREQDNSLSDLD
IKLLYDQYMNRLRLEIRSIDNEKEQLDAELDEVLDAMDNVRNSYEEEINKRTGMEFTFTALKKDLDNGFL
HKTELEAKLSGLHAWVELMKNIQEQELEEVMSQVKDVSVVLGIDNNRYNPDPHRIVEDVRAQYEALAIRS
WEELEALTRSKLNEREVLSVKYGDHLLHDRRAIAELNIQIQKMRSCILSLKSQCLRLEDNIKDVGLQGET
ALNDAKAKLAKLEEALHNAKQDLAQLVKQYQELMNIKLALDIEILTYRKLMEGEEISMESPPPAVISRIY
STPKLSSPNFCSTTSSYLATRAREDSPDKMSHSRTSLQSGLSRSQSGGTGVVSGDDLSKSHSSRSAGTAD
GSLSRSHSSMSSSVSEAFRSTSHRGTLESGSSRGTSNKSGNFADIEDTTESSVSRSHSHGSGSHRTHSNK
SGDNPEDRPSMSPSSGNRGISESCLSRSQSNRSGGLTDSVFARTDSGTSDGAQESNYSRSQSHRSVGLSP
SGFSRIQSEESNIVSGTTHVGDLSSGNSNVPKGNASRSQSSRSRDKSDSSLSRIHSGGSEAISEGSFLGS
NSGSSTSYVEGGLSRSQSEEKDFHVSHGSDSYVDPAASKTRNAEEAGVLVDDISRNQSGTSTDISGGSLS
RSQSDENEIHGSEDYAEPTVSRSRSAEDGGVPVGGISRHQCGISRDMSTERHLSRSQSDKNKFRANRSSG
SYTDSVVSQARSAEDGGVPVGGISRSDRSVEGSLSRCQSGENKFCGTEPVVSRASSTEGVSRIHSSGSRG
ASQGSSFRTPSIRSADLTDSVFMRTSSNAEQEIQEEPRSRIHSAARESSESSFSRSQSNRSRNFAESVLA
RTHSGRSEGGLSKSPNDDSQGFVEATAPRSHSHGSGGFAKLSVSTTSNKESRRVSESALSRSPSAGSKDI
SEDSLSRSQSSRSGGFLRGNSNKSKESDSNVSGRYVDEKSHREADVLKSCISRSRGTSAGRVSRMQSDES
EGPVETSTPISLHKRNEGTMEHSLSRSHSEDSESFKATGVSRSASEISKVASEVSPSRSSSKDNESSPEG
LLSRNYSYRCAVESHPCISRTESDISEAGGTISDAGLSTSYSSSSGEASINNPYVSYSEEQEGNCQYEDV
SHLHTATDEDVLESVETGDLAKGSQSGETATPKPVCMVYEFSSDEDHHPPSGELCYYVETSSME

>Lizard_K7
MSQLSYSSRSAAGPAGFSQVRVSSVSSSRGIGGGSSFRKPGGFGSSSLYNLGSSKRISLGGGVGGGGSSF
SVRSSYGYGGGLGSAYGGGYGGPLPSPGIQEVTINTNLLSPLNLEIDPTIQKVRKEEKEQIKTLNNKFAS
FIDKVRFLEQQNKMLETKYSLLQEQKTTKSNLAPIFETYISNLRRQLDSLMNDKGRLEGELRNMQDLVED
FKSKYEDEINKRTTAENDFVVLKKDVDASYMNKVELESKVDGLTDEVNFLREFYEAELRELHNQISDTSV
VLQMDNNRNLDLDSIIAEVKAQYEEIANKSRAEAEAFYQTKYEALQVTAGKHGDDLRNTKTEISDLNRTI
QRMQAEIENIKNQRGKLEAAITEAEERGELAVKDARAKLQELENALNKAKQEMARQLREYQELMNVKMSL

DIEIATYRKLLEGEESRLAGDGAGSVSMTVLNTTGSSAYGSGSGYGSGSGYGSGSGYGYGIGIGSGMGSR
SVGFSSGGGGGQGSIKTTYSVSSSTSQRSVRN

>Lizard_K84L1
MSFHSIPACGPRNFSSCSAVLQHPGNTSRISDSCSSRISGISCGGAGCFGTRSLGGIGSCGPRIAIGNCP
PPRCRYSCCGAGYGCGYGHSGICMPCSPNITRVTCNQDLLEPLNLGIDTTAQATKCQEKNELQCLNSKFA
SFIDKVQFLEQQNLMLKTKWDFLQERKCCKSNMEPMFKEYIANLKKEQECMECERAQLQAEMKNWREALE
VNKKKFEEECHRRTCTENEYVSIKKEVDCVFMDKSEKEAKVQALMKDLFFYKPTFYQEIHELQSCISDTC
VTVQMDNSRGLNMDCVMEVYRRQFEEISSRSRAEAEAWCRCQYQELKTTAAKHCDNLRHVKEELSEITRV
VHRLESEVSNVKAQCTKLEEEVAAAEERGGIAVKDARCKLVELEDALHKAKQDMACQLREYQDLMNMKMG
LDIEITTYKKLLDGEELRMGDGDCAVNISVRRSQGAVVGGSDPRPPCGPHVLCGPNKRTGSCDDY

>Lizard_K84L2
MSSPPSPAKRPSNFTSCSALIPSTESRNYFGFPSTSKGYSSGFKGAGYFSSQSLSGSSSHGPKITGASCH
PAIYGYGHRGIGAGYGYSGAGCSYSLYGPYGFNQAGIGTSGTPAITPVICNMTLLQPLNLEINSAAQAVK
CQEKNQLQCLNSKFACFIDKVRFLEQHNLMLKTKWDFLKEKKCQKSNMDPMFHEYISRLRKELERLQWER
NQLQVEVNNWRDTMEGNKKKYEEEYNRRASAENEYVTLKKEVDYIFMEKSEKEAKVETLMQDIFFYKTTF
EEEICEMQSSISDTCVTVKMDNSRDLDVACIIEEFRCRYEDIASRSRAEAEAWCQCQYQELKTTAAKHSD
NLRNFKDELEELTRTVNRLQAEIANVKAQRCKLEEEVAGAEERGEMAVKDAKCKLFDLEDALHKAKQDMA
CQLREYQELMHLKLALDIEIATYRKLLEGEECRINDGECAVNISVQRSQGAVVCNSDLIGGPKRGGSIMM
NGRDTCNPAGYKQSLSSCGAPCAVQSPPGGDMCNPPPPPQPHGIKSGKGPNVNVGATPAPCASRG

>Lizard_K84L3
MNTGGAGAPGQSRITSVTCNESLLKPLDLGIDTEALAEKSETKNELQSLNSKLASFINKVQLLEQHNLML
KTKWDFVQEMKQHRSDMEPLFDDHTSRLKKELECLEREKKEMQIEHDSSAQTLEKNKSRYEEELNKRATA
ENGFVLLKKDLDFAFADKAELEAKVEKLAKHISFLKHIYAQEISELQNCISETCVMVQLDNRRALDMNRT
IEEFRRQHEYIASRTRAEAEAWLQHQYQELKTTAAKNNDNLNAVKEEIQALTRTAHQLESQITSIKTQRC
RLEDEVVEAKEHGETAVKDARCKLSDLEEALRKAKQDMTCQLREYQSLMNVKMAMNIEIATYRKLIEGEE
CWLNRGDRAVNICVQRSEGAIVSGGNFHHGSGRTLPCETKTSKKDVHDSDKATPGSSVTAKSSPGDAGGS
GDVGGSSSSSSPVPH

>Lizard_K84L4
MSCRSYHVSSRGDGGVRNYSSSSAVFPRNVKRYSAVSTISYKGGSGFGAKGLGFGSKSLCGVASCRPRIA
LGTYQPVKYGYNLGAPSLGYGYGMPSFGNRFGGVSGPRPAGIAPVTVNPHLLQPLHLDIDPSVQMVKQQE
KEQLKTLNNKFASFIDKVRFLEQQNKVLETKWSLLQEQKRVKSNIEPLFDGLIHKLKKQLESLGCDRNKL
ESDLTSSRDILEDYRKRYEDECNRRTSAENEFVTLKKDVDCIYMHKADLEGKVESLVEEVNFLRCLHDME
LQELHSCISDTSVIVQMDNSRELDLDGIIAEVKAQYEDIANKSRYEAESWYQCKYEEMRATAGKHNDHLR
DTKNEIMELNRVIQRLKAEIDTAKGQRSKLEAAVAEAEERGEMALKDAKCKLTELEDALIKAKADMARQL
REYQELMNVKLALDIEIATYRKMLEGEENRLYADSCPVNISVCRTQGGLVCDPDPCIGGGYASSSRNFVR
SGGIVTSGGGGMCSPGLGRNLSAASCIDLGSPGLPTGGYSTGSAKSSNVKFVSTSTSFRTKY

>Lizard_K5L4
MAQKSVRVTKNFSASSAVMPGSRTGFSTVSVSRGGGAGLGHGVGGFGSRSLHGFGGSKRISMGGYRTARS
GYGYGLGHGGVGYRIVGGSGYGVGAGGIHEVTVNQNLLAPLNLEIDPTIQRVRKEEKEQIKTLNNKFASF
IDKVRFLEQQNKVLETKWSLLQDQKITRHNFDAMFEAYITNLRRQLEGILGERPRLESELKNMQDTVEDF
RIRYEDEINKRTAAENEFVVLKKDVDAAFMNKVELEARVDSINDEATFLKSFYDAELAQMHAQVSDTSVI
LQMDNSRNLDLDSIIAEVKAQYEDIANRSRAEAESWYQSKYEELQLSAGRHGDDLHNTKIEISELNRMIQ
RLHSEIDNVKKQCAGLQASIADAEQRGELALKDARNKLAGLEEALTKAKADMARQLREYQELMNVKLALD
IEIATYRKLLEGEESRLAGEGVGAVNISVVHSSGGMSYGGGSSAMGLAGGAGLGYGGGGLSMGGTSISSA
SGRGLAGSIGSGRGSSSSMKIVSKTSTTRKSYQS

>Lizard_K5L3
MSHQATVKIQRKGFSNSSAILPSTSRTSFSSRTVSRGGSCSAGGAGFGRVGGGAGFGSRSLYNFGGSKRI
SGFGGVSGAGYCASIGGGYGLGGGSSYGLGGGIGSGFGVIGTGFGGPGFPVCPPGGIQEVTINQHLLTPL
NLEIDPTIQKVRKEEREQIKTLNNKFASFIDKVRFLEQQNKVLETKLTLLQEQGQKTCKSNIEPLFEMYI
NDLRKKLNNLENDRGRLLGELNNIQNLVEDCKNKYEDEINKRATAENDFVTLKKDVDAAYLNKVELESKA
DSLNCEVNFLRALYEEELAQMHTQISDTSVVLSMDNNRNLDMDSIIAEVKAQYEDIANKSRAESETFYQC
KYEELQTAAGRHGDDLRNTKQEISEINRHIHRLRAEIDNVKKQCANLQAAIAEAEERGELALKDARQKLA
ELEDALQKAKQDMARQLKEYQDLMNVKLALDIEIATYRKLLEGEECRLASDASGAVNISVVSSTVSRGGA
AGCGIGYGGGSSLSLGGGSGFGVGGGGLGIGSGHGIGGGSYSSASGRGIGGGMSLGGGGSSSVKSVKYVS
TSQKSYRS

>Lizard_K5L2
MSRISYRASTGGGSRGFTSGSAIVGGGGGGGGGRSSFSSYSVSRVGGGRAGGGGGGYGLGGGFGSRSLYN
IGGNKRISYSTIGGGYRSGFGGGGGFGAGGGGYGAGGGGYGAGGGAGGFGFGVGLGGGGGGLGFGGPGYG
GPGAGFVVGGPGFSGRGGPGFPVCPPGGIQEVTINQHLLQPLKLEIDPEIQRVRTNEREQIKTLNNKFAS
FIDKVRFLEQQNKVLETKWDLLQQQGTTCQQSTMEPLFDVYITNLRRQLEALTNERGRLDSELRNMQDMV
EDYKNKYEDEINKRTGSENEFVVLKKDVDGAYMNKIELAGKFESLNDEIEFLKAMYDAELSQMQQHVSDT
SVVLSMDNNRCLDLNSIIAEVKAQYEDIAQRSRTEAESWYQTKYEELQVSAGRHGDDLRNTKTEISELNR
MIQRLRSEIDSFKKQCANLQAAIAEAEDRGEMALKDAKHKLSELEEALQRTKQDLARQLKEYQELMNVKL
ALDIEIATYKKLLEGEEIRLAEGVGNVGYTVVSSSTSTGYGGSGLALGGGGGGGHGFGGGFGGGSGGGYG
GGSGYGLGGGGSSYSIGGGSGFGGGSGFGSGVSLGGGSGYSSGGGRVSSGGGGGSSSVKIVSKTTSTKKM
AY

>Lizard_K5L1
MSRQSYSIRAGSGGGSAGKGFSTASAIIPSANRSGFSSLSLARAGGGGLGGGGGLGRITIGSGGGGGGGF
GSRSLYGLGGAKRISVSRSSNLRSGFGAGGGGGFGFGSGSGSAFGFGAIAGGSGFGFGGGGGGGGGGGYG
FGGGLGAARSSLGFGGGVIPGGGTIQEVTINQSLLAPLNLEIDPAIQEVRKEEKEQIKTLNNKFASFIDK

VRFLEQQNKVLETKWTLLQDQGFKTGANKGNLDPLFEAYINNLRKQLNGLINEKGRLDGELRNMQDIVED
FKTKYEEEINRRTSAENEFVMLKKDVDAAYMNKVELEAKVDSLTDELNFLRALYEAELAQLQAQMSDTAV
ILSMDNNRDLDLNGIVSEVKAQYEDIANRSRAEAEAWYQTKFEELQATAGRHGDDLRNTKGEISELNRLI
QRIRAEIENARNQCANMQTAIADAEDRGELALKDARAKLIELEEALQKAKEEMTRLLREYQELMNVKLAL
DIEIATYRKLLEGEESRLFGEGVNPVSISVVSTSAGGGGGGGGGLGGGLGGGLGGLGLGLGGGGFGLGSG
LSFGGGGGSLGSGLSFGYGGGSGGGLGFGGGSFSSGSGKGIGAGSSSGVKIISKTSTSQKSIKS

>Lizard_HBS1
MTDQEQPYNCSHRMRGFSSASSTGVTYKPPVCQPYVATGRSGEGNSSRSFHNFGQAHRISNGRYRGGGIR
EIYINKQLLEPLYLGVDPHDHQVKADEKEQIKDLNNQFACFIDKVRYLEERNQALATKWELLKSQNPAVK
KDLSPLCENYITSMRRKLDNLLCEKNQLQQEHKTMLDLIEANRCKYEEEVKRRTNAENEFIVLKQDVDGI
FKKQKELEQKKEVLEENIGFLTNFFSKERAILESQLNNTSVVVNMDNSRCLDMDGLIQEIESWYQGIVQK
SKGEANLFYWNQMEDLQSKRSQFYEKLRQNNADIAELNRYVQIMRCQVGTEKQKVASLHAALRETKELGD
HALQDAESKYKDLQNRVQTSKDRLVALVRDYQDLMNTKLALDIEIATYKTLLEGEEQRISAGVPVNVAMV
NATCALHNPARHLKP

>Lizard_HBS2
MNICGGPQCFTGRRNFSSASVIGGLGGSQGRYIPVCQPGGRPCGAGGYSSRSLNNIGSRSQRIACGQGYG
GQIYGGMVGQGMGGVGGQGMGGFGCQGMGMGGQGMGMGGQCIGNQGVSMGSPGRSLPGQAMGMGGQVMGM
PGQGGIGMGGQCIGNQGMGISGQGRGAHGQGVPIQVIGVGSQGTGGMSYGGGFGGYGGPQSQGGFTGRIG
SVAGGGRGEGIRGVCIDERLLKPLSVGVDPEEQKARTHEKEEMKTLNNQFACFIDKVRSLEQQNKVLGTK
WELLNQCVQPAKKNLEGYYENFIATLKKQLECLLSERGKLEHEQKNMQELVEEYRSKYEEEVNRRTTAEN
EFVVLKKDVDCVFLTKEELESKVDVLAQELEVLRCVFAEELTQLDSQILDTSVILKMDNTRDLHMDLILQ
NVENWYQNIAHNSKQEAEAFYHNKIAEIQNNRSKFNEELKCNQHEIAELNRVTQRLRTDLGNAKKQVSNL
QSAICDAEQRGDIALKDARNKHAELQNAYQQSKDKLASLLRDYQDLLNTKMALDIEIATYKMMLEGEESR
ICTGNPVSVTMVSGGYPVGECAAGMGGAVAYGGAVGRSLEHGVGTYSGVGEGFSSGSAGCIHTMGSRASA
GSVGRASGVGYGIRHSSGGGSGGVGGFGSNVTTKHVVGAGGGRHSSGISIDGSCVSGHHGGVHHVVGGAG
GSGGHSIGGSGIGVHTSNVYAVGTGGGHSGSMHGGSRSEVRNVHVGSSVRKTAF

>Lizard_HBS3
MMALQMSTARNIMRERGFSSRSAYGLGGHRTYAASVRYPVRNGYGVPGFTSQSLANMGVSRRISCGGYGG
YGAGCYGAYNFGHGGYGSLGFCGMAHFSPRTYEAVGSYGPFGGYSNSKGDNLHEVKVNEKLLQPLHVGAD
PQEHDARNQEREEMKNLNNQFACFIEKVRILEQENKALETKWNLLQQYAMVPRRNLEPYYENFISNMKYQ
IDCLLHTREHLANDKNAIQELVEDFKSKYEEEFKRRTTAENEFVLLKKDVDSISLSKTELEGKMNLLRWE
LEFRKSVYMEELMQLSGQISDMNILVQMDNNRDLDMDSIIKNVEAWYQSIAHRSKEEVNALYADRFQELQ
EQRGKYLNDLKINQYEIEELTRAVHTLQSDHSNVKKQVDALQASICDVDQRGDQALKDAQEKHSDLLSAL
QKAKDDLAGMLKDYHDLLNVKLALDIEIATYRTLLEGEETRLHTGFPFSIDVVRPQYMANSAGFAVPYGY
GSMGGGYNSAYGGGYPIGSHRCYNRNCSSRSVGECSKPTNNSSAATKPSGSADTEFSPGTANSSRGASYS
GGSPTSPGDIDSSSTEKAFETGVSGSGAGFSLGSEQEPTRYVGQGRRTASGPNRGYFPGGYGQHGAIPFR
AEGLGPRVRAASIILGYNPGIDMGNTGVSGISIPAVRGLSLGGGPCVGFGAPIAVCHAGTVGGLGGICYP
GPLGAPGGMCYPGPVGIL

>Lizard_K78LT
MSQPCVSSGSLSGKRCVTSASTISRGYRGPNSLSSICYTGAGRNGGYTNISHYNIGRSRRLSGGSSGGNG
GNSGVYNASFRDFSSLGYSAGSFGYGAGVAKPGSFSSYSLGAYSPGEGFSSRSLEGYGTSYSSGYRGNSF
SSRSLGAGHSKNYGTTGFSSRSLGGGVFGSRGLIAGGLFCSSLGSDAISRHGDGRGIHAVRVNAKLLRPL
CIKVDPEISRVKEEEREQIKTLNDQFACFIDKVRHLEQQNKLLETKWSCLQQQEPMEKQNTEHLYQNYIV
TLKKQLDLLLDEREQLKLEQVKFQDVVEEYKSRYEGVINRRLGAENEFVVLKKDVDCAYTSKVELEVKVD
SLRQEQDFHRCVYDTELESLATASDTNVVVSMDNSRDLDMEGIIESVRCQYEEIAQRSKDEVNALYETKY
KELQTTWGHHCNSLSSSRHEIQELTRLIQRYKADVENGKKQVEALQVAVADHEQQGDCTLKDAKEKLAEV
EKALQSTKDELARLLRDYQELLNVKVALDIEIAMYKSLLEGEESRICNSTPVNISVTGFSNISIAGGGHG
GGAGFGRRYISGPGGRRRHVSSNARFTSRSGHPTPKTGFSSRSGGNLPRCTMSSGFGECTASPVVPSGEP
RDFAPLKLGSTASGVSSSRSRRSRNRSGSCSSGRVRMSPNGGVSSIGNVTVSSSYTTHGTTTNTGEEYSS
RY

>Lizard_K78L5
MSRQFSSRSFSTGGRRGYSFPGSGFRVGNGSTSLVPVGGVGASSGGFGSRSLHSLGGSKKISMGIVSGGG
GSGGYFAGGVGGRNLGSGLGFSSFSTGYGGLGSGVGFGGGACLGGGVALGSGVGQGGPAGGFGGGGYGLS
GPGPLPAPGRIHNVVVNKNLLAPLRVEVDPDIQRVRTQEREQIKTLNNKFATFIDKVRFLEQQNKVLETK
WDLLYQQGTPALRDNLEPLYEAYIDTLRRQFESLTNDKNHLDIELKNTQELVEDFKSKYEDEINKRTACE
NDFVVLKKDVDGSYMHKIDLGSKADSLTQHIQFLRALFETELSQVQSQVRDTNVVLQMDNNRDLDLNGII
AEVRAQYEDIANRSRAEAETWYQSKYQELQTTAQKHCDELKTTKDDIAEMSRVVHKLRAEIEIVKKAVNG
LQSSIAESEQRGEIALKDAQKKLLDLQSALTSSKDELARLLRDYQELMNVKLALDVEIATYRTLLEGEES
RMSGECPTPVNISVISSSSTISGGGGGVGYSGGRGYGVGGGSGLSLGGGGGSGHINTGGGKISRFSSGGS
LSGSGNIASGGYSYGGSSSSIKILPTTSTFSRISS

>Lizard_K78L4
MSRQFSSRSGGGLRISHGYTSSAVALPGRSKGSFSSISLSQSGRGNLGVGLGDGFGSRSLYSLGGNKRTS
LYVGGSGGGFLVGGFGTGGGGGGGYGGGGGYGGGGGYGGGGFGSGGFGVGGYGAGGYGPGGYGLALGGGG
LGGGGGRMAPIVPPGGIQPVSVNPSLLAPLNLEFDPEIQKVRVKEREQIKTLNNKFASFIDKVRFLEQQN
KVLETKLNLLQQQGSTATTIHIDPMFEAYINNLRNQLDGLVGDRGRLDGELRNTQDLVEDYRKRYEDEIN
KRTTAENEFVDIKKNVDGGFMHKIELGAKADGLTDEINFLRALHEAELSQIQGRISDTNIVLQMDNNRDI
DLNSIISEVKAQYEDIANRSRAEAEALYQSKFQELQSTAVNHGDDLKNTKNEIAELSRIIQRMKAEIDNV
KKQIAGLQTAIADAEQRGELALKDARVKLTDLQHALQSAKDELARLLRDYQELMNVKLALDVEIATYRTL
LEGEECRMSGELTTPVSMSVISSSSSIGGSSYGIGGLGGGGGGGRVSSGGFGLGTGGGGGGGSGGGGGGL
GLGIGGGGGGGYGVSSGIATGGRSYSSGSARGFGGGLTAGGGGAGGGGSSSSTKYVSTTSSSSKKIVR

>Lizard_K78L3

MSRQLNAKTFGSGKKFSAGSAIVFGAGRSQRSMGGGSGSYGLYSQSLYNLGGTKSISRSVVPGDLRRGGG
FGGGGGMFSLGGFAAGVRTNGSFVNGRNGSGSFSYPPGHIQEVTVNQNLLAPLNLKIDPEIQEVRKEERE
QIKCLNDKFASFIDKVRFLEQQNKILETKLNLLQQQPMPHLKRKADHTFEAYIHGLRKQLESLSTERGRL
GSEWKNMQDLVEDFKRRYEEEINKRTTAENEFVLLKKDVDLAYMNKMELKTRTASLDKEVDFLRSLYEEE
QAQMQSQVTDTSIILSMDNSRDLDMDVIIAEVKAQYEDMANRSRAEVDAMYQSKFQELQLTAGKHDDALK
NSKTEILELNRLIHRLRAETGTVKKQCAFLQSSIAEAEERGETALKDAREKLTKLENATQKSNEELVRLL
RDYQELLNVKISLDIEIATYKTLLEGEECRMSGECGGAVNISVVSNSSMLGGNGLGRVVRLSGGRGGGGS
SSESSRGIVGIVGYSSGGGGSSHGLKIISTTSTSKRTTIR

>Lizard_K78L2
MSIHTGFQSSKGSRRGYSSCSAIGGGGGGGRHTYSCRGFGGGGGGMRGQYGSRSLHNYGGGTRISYGRGY
GGGMYGGYGGGYGGGAGGMGGGFGSGMSGGYGGGGMGGYGGSGMGGYGGGGMGGFGGGGMGGGFGPGGIG
GGFGPGGMGGGFGGGGFGPGGLAGGPGGIGSVQIDPSLLRPVNVEIDPQIQQVKNQEKEQIKLLNNQFAN
FIDKVRFLEQQNKVLQTKWQILQQQTQGRGPMQNLDGEYQLFLNGLRNQIDQIRSQKQQLMSELQNMQQV
VEDSKNKYEQEINQRTTSENEFVVLKKDVDSAYMTKVDLDAKVGGIVEQINFFRRIFDIELSQIPIGGKD
TSVVVNMDDSRDLNLEGMIDEVRAQYEEVARSSRAEAETWYRSQYEALQNAAGRHGENLQNTRQEIQELT
RNIQRLRTEIEHAKKQAAQMQSAIAEAEERGEMALRDAKQKLDELEGSLTKDKEVLARLLKEYQDLLNVK
LALDMEIAMYRKLLEGEESRISGLGSQVHVSVRGSSYGGGIGGGGLGGLGGGLGGGMGGGGLGMGGGLGG
GSFGGSGMGGSIGGGGMGGGMSGGYGSVSGGYGGGSIGYGGGSGGYGGIISSGGSGGSSYGGGGYSRGSG
SGSSTIIRRTTTSSSSVKGGGY

>Lizard_K78L1
MSRQSYGLSIGGGNKGFSSHSVSLGGGNRVSFSTASVARGSSLRVGSGGGGFGSRSLYNVGCNKRISSGG
RGGGFGGWGQGGGGFGVGACYGGGLGGGYGFGGDFSGAGGPNFPVCPPGGIKEVTINQHLLQPLHLQIDP
EIQKVRTEEKEQMKTLNNKFASFIDKVRFLEQQNKVLETKYKLLQEQGPGAIQKHLDPMYEAYINNLRRQ
LECLQNEKRGLDSELKNFQDMVEDYKTKYEEEINKRTQAENDFVILKKDVDAAYMNKVELQAKCDALIDE
INFMKCLYEAELSQMQQTVSDTSVILQMDNNRCLDMDSIIAEVKARYEEIAQMGRIDAESFYQSKYEELQ
ASVGKHGDNLQKTKAEISEHNRMIQRIRAEIDNVKKQCEKLKSSIAEAEERGELALKDARNKLTELEEAL
QKAKEEMTRLLKEYQELMSCKLALDVEIATYKKLLEGEESRMSGECQSTVNISVIGATSHISGGGYGGGY
GGGYGGGMSFGSSSGGHGGFSSIGGGGGGHASGSTIVKKTTTSSTIRSTSTSRKQ

>Lizard_K8
MKRTSVRSFSSRSYTPGPVVGGSRISTSYASSSYGGSRLGSGVSFRGASASLGGGGGGGGITAVSVNRSL
LAPLNLEIDPAIQQVRTREKEEIKSLNNKFAGFIDKVRFLEQQNKMLDTKWSLLQNQKITRSNMDSMFEA
YINNLRRQLDGLGQERVRLDSELGNMQGLVEEFKSKYEDEINHRTEKENDFVLLKKDVDEAYMNKIELES
RLESLTDELNFLRQLHEEELRVLQSQISDTSVVLSMDNNRSLDLDGIIAEVKAQYEEVAARSRAEAESTY
KSKYEELQSVAGKHGDDLRITKNEISELNRLITRIQSEIDGLKGQRANLESAIAEAEERGELAVKDATAK
LSELEAALQRAKQDMARQLREYQELMNVKLALDIEIATYRKLLEGEESRLESGMQNLSIQTKTTGYSGAL
SSGYGGYGSGYGGGFSSYSSGGYSMNSSPLESSVVSKSKAIVIKKIETRDGKLVSESSDVLSN

>SnakeP_K80
MTSHSGNFSAGSLTSPEASYSWRRQCSSSHSTRDEACSSPNLGEASKKNFSSSSLTGHGYHYTYPRVSVN
KHLLAPLHLDIDPALQEIRTKEKEEIKTLNNQFAALIGKVQSLEQHNQVLLTRWNFLKEQDSSLSDLDIK
LLYDRYMNKLNLEIRSIDVEKEQLDSELDEVLASMDVFRSKFEEEINKRSGLEFTFTTLKKDLDNGFLHK
TELETKLSGLHAWVELMTTIQEQELEEVMSQIKDVSVVLGIDNNRYMPDPHRIVEDVRAQYEDLAIRSWE
ELEALTRSKLNEREVLSAKYGDHLLNDRRVIAELNIQIQKMRSCIVSLKSQCLQLEDNIKNVGLQGETAL
NDAKAKLAKLEDALHSARQDLAQLVKEYQELMNIKLALDIEILTYRKLVEGEESSMESPPPAFISKIFSR
PKSFLPDSDITNVSDVASRAKDLTNEMTQSWSSLETGLSRSHNGGNKVASSNGSFSSCNNRSGGTPDCSL
STSYISEGRDIFESSSYSQRDNLGSPSRSSSNRSGNFADAEDTMESNSSRSYSYRIGSNRSHSKRSGDNL
ESSRYPSGEIRDISEGSLSRTQSNESGALTETVFARTYSETSDNIQEGNNSGSPSHTSAEFLSSKFSRTQ
SGESNNSSGPRYHSSRKESVPDGNQSQSQGNRDGELTDNTFARSHSGDSSEGSLFRSYSGTSASYIEGDL
SRSQSDENECRNIHGNEGYAEPIVSETTRAEDGRVPVGGISGTYSEASGDISEVSLSESQSDETEFRGSH
EEFAGPVLCRTRSAEDGGVPVGGISESSSEVSRNSSLGDQNQSSTDTVFPQAHTEGTQEDSLNNYPSNTN
TAFVEGSVSRSLSDERQSVSKGSGFKTPSIKSTTLSDSVFVRTPTSVDEEMCEESVLKIHSRARESSLET
NISGDPSPRSEELLPSDLSRLHKMESRGVSDNNPSLRGCGSKSKSESGFSKNQSNRKGGLTESVFARAHS
DPAKGDYFSSQNDGSEGFVKAAVSRINRGILEGGSSGSQSSRREGFADTDLMKYQSKESKGSHEDTSFPD
YGVRYASETNEAVLQSGLSRIEYEQAGSFSRNRSERGNPVETSTPIQLHRKNEEATEHSFSRSHSEESGS
FKAADLLSRSSSQESRGISEATISSSCGKENEKCTEGMLFRNYSYRCAVTSHPRTESAEAEDIPESELYR
SPSSESGRLSSPGIYRGRSGEREDESEVAFSRSYSNRDEVAMNNQCISHDNRNE

>Snake_K7
MSQLTSFSSRSATAPSNFSQVRISSVSSSRGIRGGGSFRKPGVFGSTSLYNLGSNSRRIAPSFPVSPSFG
YGGGIQEVIINPNLLSSLNLAIDPTIHKIRKEEKEQIMVLNNKFASFIDKVRFLEQQNKMLETKWSILQQ
QKTSTRNNTAPVFDAYINNLRRQLDSLQNDKGRLEGELKNMQDLVEDFKNKYEDEINKRTTAENDFVLIK
KDVDASYMNKVELEARVDGLTDEINFLRALYDAELAELQNQVSDTSVILQMDNNRNLDLDSIIAEVKAQY
EEIANKSRIEAESWYQSKFEVLQATAGKHGDDLRSTRNEIAEINRLILRLQADIDNVKNQRAKLESAIAE
AEERGTLAVKDAKGKLEELEVAMNKAKQEMAHQLREYHELMNVKLGLDIEIATYRKLLEGEESRLSGDGA
GSVNMTLLSSGGGGGGAYSSGSGIGYGVGFALGSGMGSGPLSFTSGAGSGSLKSTYSVTSTSRRSTRN

>Snake_K5L2
MSHRANIKIQRRGFSNSSAILPNLCRTNWSSQSVGRSGNCGIGRASWGRAGGTGFGSQSLYNLGGPRRIA
VGGSYGSGIGGGYGYGGGVCNGYAVGGGIGGGYGLGGAGFGGGRGDSCFPVCPPGGIQKVTVNPNLLVPL
KLEIDPTIEKVRKEEGEQIKILNNKFASFIDKVRFLEQQNKVLETKWTLLQKHDQKTVMSNVEPLFEAYI
NNLRTQVNDLEKEKVKLDGELHTTQNLVEDYKSKYEDEINKRTDAENDFVILKKEVDAAYMNKADLETRL
QSLVDETDFLRLLHETELADLQRQISDTSVVLSMDNNRYLDLDSIIAEVKAQYEDIAKKSRAEAEAWYQS
KFEELQGTAGRHSDDVRNAKHEISELNRHVQRIKAEIESVKKQCANLQAKIAEAEDRGEQALKDARQKLA
ELEDGLQKAKQNMAKLLKDYQELLNVKMALDVEIATYRKLLEGEECRLANDAADAVNISVISSGISRGSA
AGSGLGYGSGFSMGGGSCFAAGGGNCNIGSVDLGDGSCSSMKFVSYPPTGYIN

```
>Snake_K5L1
MSRVSYRSSSGGANRGFTSGSAIVGGGGGGGRSGFSSYSVSRVGGARGGGGSSYGLGGFGSRSLHNMGGS
KRIAYGTVCGGLRSGGYGGGYGIGGGSGFGYGMGAGGAGYGMGGGPGGFLVGGPGFVRGGPGFPVCPPGG
IQEVTINQHLLQPLNLEIDPEIQRVRSHEREQIKTLNNKFASFIDKVRFLEQQNKVLETKWELLQRQGTQ
VQQRNLEPLFENYIANLRKQFDYLVNERGRLDMELRNMQDMVEDFKNKYEDEINKRTGAENEFVVLKKDV
DGAYMNKIELASKSDSLNDEIEFLKALFEAELSQMQQQVSDTSVVLSMDNNRSLDLNSIIAEVKAQYEDI
ANRSRTEAESWYQSKYEELQVSAGRHGDDLRNTKTEISELNRVIQRLRSEIDAYKKQCANIQAAIAEAED
RGELALKDAKQKLNELEECLQKTKQELARQLKEYQDLMNIKLALDIEIATYRKMLEGEEIRLQEGVGNVS
YSVISNTTTTGYGSGSGLGLGGGFGGGSAGGLGGGFGVGIGGGGGGGGYAISSGSNYGGTGFGSGFSSGG
GSSYSIGGGRHSSGGGGGSSVKIISKTSTSRKSAY

>Snake_HBS1
MSRQISAGSQRNAQSHFNHYSASSSSSWFHSGSKSSRNMSNFGGRTTLSLCGDHKSGTCGSGIQGVPRRY
SIQGIHVDPKLLEPFHVSISPEIQKVKCKEMEQMKSLNSKFAGFIDKVQRLEQKNQLLETKWAFLQQQTK
PPTESLECMFEQYIASLKKELEYLQYEKENLQNDRESAKAQTADCKCKYEEKVKQHVAAEKEIIELRKDV
DYIFSNNAELERKVSLLDREIKFLKCVYKKEKEQLDKQDQDVDVVVKMDNSRNLDMDNIIAMVRKQYEEI
MQRSKAEADKFYQNKYEEAQSKSSSYKWDLKNREQQISELIQQVEKVQGEIQSLQKENEELKKTICEAAK
GGEQASKEGQQKYAELVKALQNGKDELASLVRDYQKVLNVKIVLDIEIAAYKCLLEGEEERISRDSSSSI
SISVTNSPCSSQSAAKQGKKCSEKAEGSCQCKPAGTKISISSDSETKCQSEKSSTVCKWSTNTK

>Snake_HBS2
MDQQPFPQANLRRSSITAIGCNDEIQINGMQNHSANCSFKRGNSSKSFHSFGRNHRISCYMGHQLKNLRG
SRMDMRSITDHSFFVDAYNLGYRDIPKPRYRNEGLLEVNINKLLLEPLYLGVDPHDHQVKVQEKDQMKDL
NTQFASFIEKVRSLEQRNRILITKWELLQNQKMPKVKKDLSTLFDNYIANLRKKIDYMFNEKNKLENQQR
MMQDLIEEYQCRYKEENNRKEHLENEFILLKQQVDNEFKEQKERELKRELLMENKEFLKYFFTEEHAILN
CHPPDTSVVLNMDNSRGLDMDVLIKNIDSWYHNIAQRSKEEANLFYQNQIEDLQNKKCQCRESLQKTSDE
ITELNRLVHLMQSKLDNEKKKTAALQIAIGDIEHKGDHALKDARAKQTELQNRIQNSKDQLAVILRDYQD
LMNTKLALDIEIATYNTMLEGEENRXXX

>Snake_HBS3
MNIPVGSRCLGGSRRFSSASMVGGRSSAPPCQPYGRPHGAGGYSSMSLNNFGGSQRISYSQGYGGQIYAG
VGGQRIGMGGQAMGMGAHAYSGGFGGGYGGAECQGGYGGYGRMGSMSSMRSEGIRGVSIDERLLKPLSLG
VDPEEHKARTHEKEEMKILNNQFACFIDKVRSLEQQNKVLSTKWELLNQYVQPTRKNLEAYYGNFIGSLK
KQLECVLTEREKLEHEQKNMQELVEEYRSRYEDEVNRRTTAENDFVVLKKDVDCVFLTKEELESKVDVLA
QELEVLRCIFAEELTQFNHQICDTSVVLKMDNTRALDMDLILKNVEAWYQNIAHTSKQEAEAFYHSKIAE
IQNNRGKFNEELKNNQHEIAELNRVVQRLQADTDNAKKQVSSLQSAICDSEQRGDIALKDARTKHLELQT
AYQQSKDKLACLLRDYQELLNTKLALDIEIATYKALLEGEESRIYTGNPVSVAMVSGGYPVGECPPGMGV
GVAYGAAAGRGMEHNHGNFNGTGEGFTTGSAPCIHTMGPRNAGGSGGMAAGVNHGMVHGAGLGTAGGVSA
SSGGYTANVSTKHVMTSGGGRHNSGLSADGNCVNSHHSGIHHSVGATYVSGGSGMGGHSSGAGGISGAVF
GASRGESSSGLRNVQVTSVSSSVRKFMY

>Snake_HBS4
MTHQVSATRNTFLSGRGFSSVSALGGLGGHRTYVASVRHPVGSGGEVHGFSSQSLSNMGGRGKVAHGGYV
LGYYGCANHGSPAFVGPVGHYGPRVYDSAGNCGIFGGHSNGRGDGIHGVRINEKLLKPLHVGVDPQEHIV
RDHEREEMKSLNNQFASFIDKVRNLELQNKVLETKWNLLQEHVIPAKKSLEPYYENFISNMKKQLECLLN
NQEHLRNENDGIEQLVEDFKCNYEEEFKRRTDAENEFVLLKKDVDSISLSKSELEGKVHLLSGELEFRRG
IYAEELAQLSTNVYDTNILLQMDNSRNLNIDSIIKSVEAWYKNVAQGSKEEVNAFYENRFQELQQQRGKY
SQTLKINQQEIADLTRLVHKLQSEYDIIKKQVNGFQTSVCEIEQHGDCVLKDARDKHIDLQTAVQKAKDD
LARLLRDYQELLNTKLALDIEIATYKTLLEGEETRIHLGNPVSIDVIKPGIENAVGSSFSSGYAPLGGGY
GGGYDMGFGKGYGVGGQERSNRNYSSRSAGFDQNTGRSNIAPGNRSADAEYYQGRGYSYRNESCSGHPSA
GMRQFSTEEAGRGAGHGPGAGFSSRTAYGSVCGGGRGYGSTSVVGNCPDGNYPRGGYEPSGVAAFGDGGW
YSRAGVPMGGAAEGNPASVGIGGAAGGPAGVCYSGPLGTSAGVCYAAPAGTPAGVCYAAPAGTSSGVCYA
GPVGTSGGLCYPTAVRLF

>Snake_K78LT
MSRQCCTSKSFNGRRNYISSGGLHAGSNASSSFRLLEHSRRYSGANLSSRQGYGAAPFGYSLGPVGFGAG
IAKNEEFSSQSAGTNSQARAAGNQSSGHHRHEAFTSQSVGGGNSHGRRYRPEGFSTRSLGCNVRRAVGYE
CLSRFGEGRGIHTVRINPNLLQPLRIQIDPEISRVKEEEREQIKTLNDKFVSFIDKVKHLEQQNKLLETK
WNCLQQQEPVEKANIQPLFENYITSLKQQLQRLLNEREQLQLEQSKFQDMVEEFKSRYEEEINHRMSAEN
NFVLLKKDVDIAYSNKVDLEVKVESLRQELNFIRCVFEAEIESLQAANAETNVIVSMDNSRVLDMEGIIH
SVRSQYEEIAQKSKDEVNALYENKYKELQNTWGNYCNNLSSGRHEIQELTRMVQRCRSEVESTKKQIDAL
QTSIADNEQRGESALKDAKLKLTEVENNLQSSKDELARLLRDYQNVLNVKIALDIEIAMYKSLLEGEENR
ISNAPPVNISVVGSSNISVAGGCSYGRTIGRRQGYTQRNKNGSGRSSSRSGDHGSKHALTPRSGDSLSKS
SISSGFGQSGSPTVVSSTPKNSDLANVGSPGSGGYSSKSRGYSSTNTTHISATTGVSSKGASGGISVSGS
YNIQSSGGYSGDDYSSLY

>Snake_K78L4
MNRQFSSRSFSTGGRRNYSVSNSGFRVGNSSTSVIGGVGSFGGGFGSRSLYNVGGSKRISMGLASGGGGG
AHFGRSLGGGLGFSSYSSGYGGYGGGVALGGGIGLGSHVGGFGGGSGGGYALSGSMPCPPPGRIHNVIVN
KNLLTPLHVEVDPEIQRVRVQEREQIKTLNNKFASFIDKVRFLEQQNKVLETKWDLLCQQGSPALRDNLE
PIYEAFIEALRRQFDSLCNDKNHLDIELKNTQELVEDFKNKYEDEINKRTACENDFVVLKKDVDGSYMHK
IDLGSKADSLTQYIQFLRALFDAELTQLHSQVKETNVILQMDNNRDLDLDSIIAEVKAQYEDIANRSRAE
AEAWYQTKFQELQSTAFKHGDDLKTTKDEIADLSRIVQRIRSEIEIVKKAISSLQSSLGEAEQRGELALK
DARTKLLDLQTALTSAKDELARLLRDYQELMNVKLALDVEIATYRTLLEGEECRMSGEFETPVNISVISS
SSTISGGGGGGYGVGGGSGYGAGGGAGYAIGGGSSGLNMGGGGGGGHSHISVGGGKVSRFSSGGSLSGSG
SLTGGGYTYGGGSSSMKIVSSTSSSSKRLIS
```

>Snake_K78L3
MSRQFSSRSGGGIRTSRGYTSSTAVIPGGTKASFSSVSLPRGVKGSTGLGGGFGSRSLYNLEGSKRISFG
ISHGGFGGGFGGGLGFGGGGGGGFGSGDGYGYGAGGYGLSLGVGSYGGGGRMGPMVSPGGIQKVTVNPNL
LSPLHLEIDPEIQKVRQHEREQIKTLNNKFASFIDKVRFLEQQNKVLETKWNLLQQHGSSVPKLSLEPLF
ENYILNLRRQLDSLIGNRAKLDSDLRNMQDLVEDYRRKYEEEINKRTTAENDFVGIKKDVDVSFMQKVDL
QAKVDRLIDEINFLKALHEAELIQIQGQISDTNVVLQMDNNRDLDLNSIIAEVKAQYEDIANRSRAETEA
WYQNKFQELQIIAVGHGDDLKTIKNEIRELNRLINRLKAETENVIKTISRIQKAIADAEERGQLALKDAQ
VKLNDLQQAIQNSKDELARLLRDYQELMNVKLALDIEIATYRTLLEGEECRMSGELTAPVSMSVMNSSST
MSGGSQVSSGGFGLGAGGGGGGSGAGGSLGLTMGGVGAGGFGISSGITTGGSSYSTGSYGGGIGGGGGRG
SSSMKYVSTTTSSSSRKAVR

>Snake_K78L2
MSFQAICQGSKGSRRGYSSCSAFGGGTGGGIRRSYSSCRGYDGGMRGQYGSRSLHNYGGVTRISYGSGYG
GSICGGYGGGYGGMGGGYGGIGGGYGGIGGGYGGIGGGYGGIGGGFGGRGLGGLGSRGIGSVQVDPNLMR
PVRVEIDPQIQQVKNQEKEQIKVLNNQFASFIDKVRFLEQQNKVLQTKWQILQRQSQGIGPTMNLDADFQ
QFLNSLRSQIENIRSQKGQLTTELQSMQHYVEEYKNKYEEEINRRTTAENDFVVLKKDVDCAYLSKSNLD
SRVGALADQINFLRRIFEVEISQYQTGGQDTSVVVSMDNNTHLNLEGIIDEVRSQYEEIARSSRAEAESW
YHSQYEALQNTAGRHGDNLRNTRQEIQELTRNIQRLRSEIEHVKKQXXX

>Snake_K78L1
MSRQSFGMSVSGGNKGFSSSSACFAGGQRMSFNSVSASRGCSVRSGGGGAGFGSRSLYNLNQGGNKRISY
GGVGSGFGAGFGGGCGQGGGSFGAGGGYGAGYGFGNAFPACPPGGIKEVTINHHLLQPLNLEIDPEIQKV
RVEEREQIKTLNNKFASFIDKVRFLEQQNKVLETKWKLLQEQGQGTGGQKNLDHFFEAYISNLRRQLDGL
LNERRGFESELKNFQDLVEDFKNKYEEEINKRTAAENDFVLLKKDVDAAYMNKVELQAKMDALTDEINFL
RCMFEAELNQMQQSVSDTSVVLQMDNNRSLDLNSIIAEVKAQYEDIAQKSRAEAESWYQCKYEELQVSAG
RHGDNLRDTKTEIAELNRTIQRMRAEIENVKKQCEKLQASIAEAEERGELALKDAREKLSELEEALQKSK
EEMARLLKEYQELMNVKLALDIEIATYRKLLEGEESRMSGECQSAVSVSVIGSSSGGGMGSGFGGGVCFG
GSSGGFGGFSSGSGRGGYSVGGGGVCSVGGGYGGGYSSSCGSAIVKKTTTSSSSSFMSSGRK

>Snake_K8
MMRKNYRNTSSTVRSFSSRSSGPVASSRISTSYSSAGSGFGGSRYGGGASFRGTSAGSLGGITAVSVNKS
LLAPLNLEIDPAIQQVRIKEKEEIKTLNNKFANLIDKVRFLEQQNKMLETKWSLLQNQKTTRSNMDSMFE
AYINNLRRQLENLGQERARLDAELGNMQGLVEEFKNKYEDEINHRTEKENEFVLLKKDVDEAYMNKVELE
SRMECLTDEINFLRQFYDEELRVLQSQISDTSVILSMDNNRSLDLDGIITDVKAQYEEIANRSRAEAERM
YQIKYEELQTVAGKHGDDLRSTKNEINELSRMINRIQSEIDGLKGQRASLEAAITEAEERGELAVKDANN
KLAELESAMQRAKQDMARQLREYQELLNTKLALDIEIATYRKLLEGEEIRLESGMQNLSIQTKTTGYSGG
LSSGFSGYGGGYNSSYSSGGYSMNMLPMESSAISKTKAIVIKKIETRDGKLVSESSDVLSK

>Frog2_K80
MASNAQKSSHTFSVWQQEDMVVRESLFPSPPPSVSCQVISSATNGNHSTLLEETFESKLKITSDIHSSLH
SEEAETKLRAALATFNDKIRYLEKQREILESRWSMIQTEETTQTDLEPQYLSYISRLLGEVKNVTQNNHQ
TQRQLLDMMDSVNDMKDKFEDELFRRTDVEYSFVELKKDADGRSLEQTELETRKQDIKEMIDLMKTVYKQ
ELKELMEDSGDISVLVNMEQSCPLNLDKVVEEVKAQYERIASWSRDEAQALSKNKLAEGVQRVGRYELEL
KSSRSEITHLNSKIQRLRSEIQSIQKQCSKLEQDVSLAQTNSNMAIVDANTKVTEIQDVLLKAKQDMAKQ
LRDYQELLHVKMALDVEIAAYNLLLEGEESRLQASAPVNVQHGNDVCAFTEPSRIRMHF

>Frog2_K7
MAFSSSRQTSFSTRSAVPAASFSQMRISSTRSSSGGAGLGRAGGSFSSSSLSNIGGGVRKSVSYGVSSGR
SGGAGFGGAGFGGAGFGGAGFGGAGFGGGVAYSGPGIQEVTINQSLLAPLNLEIDPTIQHVRQEEREQIK
TLNNKFASFIDKVRFLEQQNKVLETKWELLQNQKSAKASNVGPLFDAYIANLRRQLDGLTGDKGKLEGEL
RNMQDLVEDFKNKYEDEINKRTSAENEFVVLKKDVDAAYMNKVELEAKMEALTDEINFLRALYEAELREL
QEQISDTSVVLSMDNNRALDMDSIIAEVKAQYEDIANKSRANAEAVYQNKFQELQATAGRHGDDLRTTKT
EISELNRAMQRLQAEIESVKAQRAKLEAQIAEAEERGELALKDARTKLAELEAALQKAKQDMARQLREYQ
ELMNVKLALDIEIATYRKLLEGEETRISEGPGPVSVSVVNSTSSSMGGSSSGYGAGYGSGFGAGGSFSSG
GFGSSSTKFGSGGSSGVKSYSVTTSSSSRSFRQ

>Frog2_03
MSQHSICGSKGYKNFSSVSACLPKNVSRYSVTSLSSKRGVGHGHRAQPSFSSRSAYNTGSGIHKISVGSH
HSGGGHGCGFNYGFGHGSGFERGFAGGAMNGGIAPVTVNQSLLAPLNLEIDPNIQRVRREEKEQIKTLNN
KFASFIDKVRFLEQQNKMLETKWAILQEQRTARSHIEPLFEAYIGNLRRQLDCLLGEKSRLEGERKNMED
IVEDFKRKYEEEVNRRTTAENEFVVLKKDVDAAFLHKAELQTRVESQTDEVNFLRTMFEAEIAQLQGQIS
DTSVIVSMDNNRNLDLDGIIAEVKAQYEEIANRSRAEAESWYQSKYEELRLTAGRNGDDLRNTKNEVADL
NRMINRLRGEIETVNAQRGKLEGAITEAEERGEMATKDAKKKLADLEEGLQKAKQDMARQLREYQELMNV
KLALDIEIATYRKLLEGEEGRLSGEGAGSVKISVVSSTVGGSHGGGNSYHHESGMSSGNGHGCSGKFGGG
SGFSSMGGAFISRGKHSSVSSSGSKKM

>Frog2_04
MSVSRQTVYSTGGTKRFSASSAVPSGGTRSSFSTLSVSRAGGGGGRASYGGYGGFGSRSLQNAGGSRRIA
VSYGAPSHFGFKSSGIESHIGGNYGSDMMSGIGMGQGPFGGPGFPVCPPGGIQEVTVNQTLLAPLNLEID
PSIQRIRTEEREQIKTLNNKFASFIDKVRFLEQQNKVLETKWNLLQEQGVKSVKCNIDPLFEAYISALRR
QLDSLLSEKVRLDSDLRQMQDAVEDYKKKYEDEINKRTSVENEFVVLKKDVDAAYMNKVELEAKLNALTD
EINFLRAVYEAEIAQMQTQISDTSVVLSMDNNRTLDLNGIIAEVKAQYEDIANRSRAEAESWYQTKYEEL
QQSAGRHGDDLRSTKTEISELNRMINRLRSEIDSVKKQCAKLQAAITEAEERGELALKDAKKKLADLEEA
LQKCKQEMARQLREYQELMNVKLSLDVEIATYRKLLEGEECRLAGEGVGPVNISVVSSSYGGGSGSGSGY
GMGGGSGFGGGSGFGAGSGYAIGGGQSFGGGSVSGGMGGASFSVSSGKMAAFGPGSGSSSNVKIISKTST
SSRKL

>Frog2_05

MSHYKQFSVRSVATSGKPGFSSFSVSKSGGGFGGSSGGGSFGSRSLYSVGSRKVSSVGSASGGGFGFAAG
GFGGRGFGGGVGGGAFGGAGGGAFGGAGGGAFGGAGGGGFGGAGFGGFGGAGFPGGPSVGIQEVTVNQSL
LAPLNLEIDPTIQKVRLEEREQIKTLNNKFASFIDKVRFLEQQNKVLETKWSLLQEQGGQVKGSRKNNID
PIFDAYINSLKRQLDALQNDKLRLDGELRNMQDLVDDFKNKYEDEINKRTSAENEFVVIKKDVDAAYMGK
VELEAKVDALTDELNFLRTFFEAELGELQAQISDTSVVLSMDNNRALDLESIIAEVKSQYEDIAKKSRAE
AESVYQGKVQELQASAGEQGDVLRNTKSEISELNRKSQRLKAEIENVKKQIAKLQASITEAEERGDLVLK
DAQSKLAELEAALQKVKQDMARQLREYQELMNVKLALDVEIATYRKLLEGEEFRLNSDVNNVSISVISSG
GKSSLGGGGGGSGAGFGFGSGGAAFGGSGGAAFGGSGGAAFGGSGVSFGSGGGGFSSSSSSFGGGVGSVR
YSSSQSSYRS

>Frog2_06
MSWSLHQRVTTSTGSGKGFSGLSLGGGSRMGINSASASSRLSNSSARFGSRSLILSGSRKVSSVASSGRF
GAGYGAISAAGAGGFGGPGSFSGIQQVNINQSLLAPLNLEIDPSISVVRKEEREKIKLLNNKFASFIDKV
RFLEQQNKVLETKWSLLQKQQVSKSRSSEIESIFNTLITSLRSQLDSLVKDKGLLGGELQVMKDHAENFK
NKFELEFKKRTADENDFVSLKKDVDATYLIQVQLETKQKALDDELAFLRNLYKEELDGIKQQTAGTSVVL
SMDNSRMLDLGDIIADVKAQYEDTTRRSKEEAEAAYQSKYQQLQLAAGQQGEDLKSSKKEISEVNRSVQK
LRNEIESVKKQIASLQVSIQDSERRGDIALRDAQEKLVNLERALQKAKQEMAQQLREYQTLLNVKLALDV
EIATYRKLLEGEESRITGQVSDAVTVSFVSGAKSSFDATKRSAGIDTGVKRRGSSGVKIISKTESSSLVS
LYQ

>Frog2_07
MNRGQQSFASRSVAGTKSFSSSSLGGGGLGARGGGGRISFSSASVSKGMGGGRASGGGGGFIQSGGASFS
SQSLHNLHPLGGTRKTAISSGSVRGAGGIGFGGAGGGGFGGAGGGGFGGAGGGGFGGGGFGGGGFGGGGF
GGGGFGDGGFGGGLGGIDGGFGGMNFPVCPPGGIQEVTINQSLLAPLNLEIDPAIQKVRTEEREQIKTLN
NKFASFIDKTRFLEQQNQVLKTKWELLQEHGQKGVTKRSNIVPLFEAYISNLRRSLDSLLNDKHRLESDL
RSTQDLVEDYKNKYEDEINKRTTAENEFVVLKKDVDGAYMNKVELDCKVDALTDEINFLRTLYEAEVQDL
QGQLSDISVVLSMDNNRALDLDGIIAEVKCQYEEIANKSKAEAEAACAMKFQQLQATAGQHGDSLKSSKT
EIADLNRMIQRLKAEIESVKKQCANLQTSITEAEERGELAVKDAQKKLDELQEALRKAKEEMAKQLHDYQ
RLMSIKLALDIEIATYKKLLEGEECRISGEYQNPVSISVISGSTSISSGVGGGFGAGGGGYGAGGGGFGA
GGGGFGAVGGGYGAGGGGFGAGGGGFGAGGGGGGGYGVGGAGGGGFGAGGYGVGGGGQFGGGGGGYSMGG
GGGGGFSSGSRQGVAIISTTSSSSSSKMNY

>Frog2_08
MSQVRQSLSSKPGSCNVRGYTACSIGGGFVGPKYNFSSLSSAGGNPITYSSGKLKSTGSLYCLGGHKRTS
ISSGRVLGSTLGTSKGFGTDSFPVCPPGGIQKVTVNQILLQPLNVEIDPNIQKVRTAERDQIKGLNNKFA
GFIDKVRFLEQQNQILGTKWNFLQEKSQKLCSRRDTIKPLFDAYITNLQRQLEGLRSEKCRLDGELKNMQ
DVVEEFKSKYEEEINKRTCTENEFVALKKDVDILYMKKAELEAKEESLGNEINFLRNIFDAELAELQDCL
SDINVVLSMDNNRDLDLNGLIAECKAQYEEIAARSKTEAEAAYSKKFQQLKEAAGQHGDNLRSSKTEIQD
LNTMIKRLQSEIECVKKQIAGLQAAICDAESRGEATLKDANCKMSELEAVLQKAKEDLAIQLREYQELLR
VKLALDVEIATYRTLLEGEESRMHGEITNDVKISVYSTKKLGDSNAECISTCNLKSSGSILAGVEAPKCA
PMCAPKCAQPPKPCGTAYAKLGY

>Frog2_09
MSQVRKSLSSKPGSCNVRGYTACSIGGGFVGPKYNFSSLSSAGGNPIHHNSGKLKSTGSLYCLGGHKRTS
ISSGRVLGSTLGTSKGFGTDSFPVCPPGGIQKVTVNQILLQPLNVDIDPNIQKVRSAERDQIKCLNNKFA
SFIDKVRFLEQQNQILGTKWNFLQDKSQKLCSRRDTIKPLFDAYITNLQRQLEGLRSEKCRLDGELKNMQ
DVVEEFKCKYEEEINKRTCAENQFVGLKKDVDVLYMKKAELEAKEETLANEISFLRSLFDAELAELQDCL
SDINVVLCMDNNRELDLNGLIAECKAQYEEIAARSKTEAEAAYARKFQQLKETAGLHGDNLKSSKTEIQD
LNTMIKRLQSEIECVRKQIAGLQAAICDAESRGEATLKDARCKMSELEAVLQKAKEDLAIQLKEYQELLR
VKLALDVEIATYRTLLEGEESRMHGEITNDVKISVYTTGKLGSSNAECISTCNLKSSGSILAGVDGPKCA
TKCISKCVPKCAPKCAQSPKPCGTTCTKPSYM

>Frog2_10
MVTVDPNIKRVKAEEREQIKTLNNKFAAYIDKVRFLEQQNKVLETKWKLLQEQGTKGSTKRANLEPLFEK
YIGDLNKYLSTLINEKGSLEHELKNLQGFVEDYKKKYEEEINKRTKAENDFVVVKKGVDTAYMANRELEA
KADGVTSEINFLKTLYAAELSQVHDSVTNTSVVLTMDNNRDLNLEGIIQEVKAQLELSAQRSKMEAEALY
DNKYKELQRTAEGHGDSIKNSKSEIAELNRRIQRLKAEIESTKKQIAGLNQSIAGAEEKGNLALKDAEKK
IQDLEAAEKKLKEDMARQLKEYQELLAAKISLDVEISTYGLMLGGEERRMSGQIVNNVNISVISGGSNVY
SALGAAAGGIRGGMGGGMGSGMGMGGDMRLGHSGGRGGHGAEGMGH

>Frog2_11
MSFSQVSIRKRQTVSSGGGGRGGFSTCSLGAIGGGSRAGGSYGSRSVYNLGGKYRTSISAASARGGAGGF
GGAAGGGFGGAAGGGFGGGFGGGHGGGMFGGAGGMGGMGGMGGPGFDGGDMGFPVCPPGGIQQVTINTHL
LQPLDLAIDPNVQRVKAEEREQIKTLNNKFAAYIDKVRFLEQQNKVLETKWKLLQEQGTKHSTKRANLEP
LFEKYIGDLRRYLDTLINERGRLEHELKNQQDLVEEYKKKYEDEINKRTKAENDFVLLKKDVDAAFMAKT
DLEARVDGLTSEINFLRTLYAAELSQVHEQVTDTSVILTMDNNRDLNLSGIIQEVKCQYEQIAQRSKMEA
EALYDNKYKELQKTVEGHGHSIKNSKAEIAELNRNIQRLRAEIENLKKQIAGLNQSIAGAEEKGNLALKD
AEKKLHDLEAAEKKLKEDMARQLKEYQELLAAKISLDVEISTYGLMLGGEEHRMSGQIANNVSISVISGG
SNVYSALGGAAGGMGGGMGSGMGIGGGMGDGMGSGSCLQGSMGSVHGGKYGHGAGYEGGSMHGGSGSFGH
HGGKTSSSVTSTTYTTKKTY

>Frog2_12
MSFSQVSIKKRQTVSSGGGGGGRGGFSACSLGAIGGGSRAGGSYGSRSVYNLGGKSRTSISAASARSMVG
GLGSAAGGGFGGGAGGGFGGGAGGGFGGGAGGGFGGGFGGGHGGGMFGGAGGMGGMGGMGGMGGMGGMGG
MGGMGGPGFDGGDMGFPVCPPGGIQQVTINTQLLQPLNLTIDPNVQKVKTEEREQIKTLNNKFAAYIDKV
RFLEQQNKVLETKWKLLQEQGTKGSTKRASLEPLFEKYIGDLRRYLDTLTNEKARLLQELKNLQDLVEEY
KKKYEDEINKRTKAENDFVVLKKDVDAAYMIKTELEAKVDAVTSEINFLRTLFAAELSQVHDSVTDTSVV
LTMDNNRDFNLEGIIQDVKAQLELAAQRSKMEAEALYDNKYKELQRTAEGHGDSIKNSKTEIAELNRRIQ

```
RLRAEIENIKKQIAGLNQSIAGAEEKGNLALKDAEKKLQDLEAAEKKLKEDMARQLKEYQELLAAKISLD
VEISTYGLMLGGEEGRMSGEIANNVSISVISGGSSVYSALGGAAGGMGGGMGGGMGGGMGMGGGMGMGVG
MGGGMGGGMGGGMGGGMGMGGGMGSACGYGQGMGGMGFEGGSMHGGSSYGHSGGKTSVAIASTTSTTKKT
Y

>Frog2_13
MSFSQVSVKKHHSGSGGGGRGGFSACSLGAIGGGSRSGGSYGSRSVYNLGGKSRTSISAASTRSMAGGLG
SAAGGGFGSAAGGGFGSAAGGGFGGGAGGGFGGGFGGGHGGGAGGMGGMGGPGFDGGDMGFPVCPPGGIQ
QVTINTQLLQPLNLTIDPNVQKVKTEEREQIKTLNNKFAAYIDKVRFLEQQNKVLETKWKLLQEQGTKGS
TKRANLEPLFEKYIGDLNKYLSTLINEKDRLTQELKNLQVLVEDYKKKYEDEITKRTKAENDFVLLKKDV
DTVYMAKTELEAKVDAVTSEINFLRTLYAAELSQVHDTVTDTSVVLTMDNNRDLNLEGIIKDVKAQLELC
AQRSKMEAEALFDNKYKELQKTAEGHGDSIKNSKSEIAELNRRIQRLKAEIENIKKQIAGLNQSIAGAEE
KGNLALKDAEKKLQDLEAAEKKLKEDMARQLKEYQELLAAKIALDVEIGTYGLMLGGEEHRMSGDIVSNV
SISVISGGASVYSALGAAAGGMGGGGMGGGMGSGHGGGYGHGGGFGSGHGSGHGGGFGAGHGSGHGGGHG
KTSVAIASTTSTSKKSY

>Frog2_K8
MSIKTTRVTYRTSSAAPRSGGFSSYSYSGAPAASRASTASFSLGSSYGGSSIGGSRFGSGYRSGFGGAGV
GSAGITAVSVNQSLLAPLNLEIDPTIQQVRTEEKEQIKTLNNKFASFIDKVRFLEQQNKMLETKWSLLQN
QKATRSNMDAMFEAYIGNLRRQLDGLGQDKMRLESELGNMQGLVEDFKNKYEDEINKRTELENEFVLLKK
DVDEAYMNKVQLEARLEALTDEINFLRQLYEEELRELQSQISDTSVVLSMDNNRSLDLDGIIAEVRAQYE
DIANKSRLEVESMYQVKYQELQASAGRHGDDLKNTKAEISELTRYTTRLQSEIDALKAQRSNLEAQIAEA
EERGELALKDARTKLAELEAALKNAKQDMARQLREYQELMNVKLALDIEIATYRKLLEGEESRLESGFQN
LSIQTKTVSGVSSGYGGGISSGYGGGISSGYGGGISSGYGGGYSSYSSNVSSLPLETSKTSKRSIVVKTV
ETKDGRVLSESSDVFSKP
```

Suppl. Fig. S1. Amino acid sequences of type I and II keratins of sauropsids and frog (*X. tropicalis*). The amino acid sequences of sauropsidian and frog type I (**A**) and type II (**B**) keratins used for comparative analysis of cysteine contents and phylogenetic analyses (Figures 1-4, Suppl. Figure S5) are shown in FASTA format. The sequences are sorted according to species and, within each species, according to the order of the corresponding genes in the type I or type II keratin gene clusters (Figures 1 and 2). Accession numbers and data about the genes are provided in Supplementary Tables S1-S6. Species: chicken (*Gallus gallus*), alligator (*Alligator sinensis*), turtle (*Chrysemys picta bellii*), lizard (*Anolis carolinensis*), snake (*Protobothrops mucrosquamatus*), western clawed frog (*Xenopus tropicalis*).

A

>Platypus_K224
MSHSRQKSSLWLFDLGRGGGGFAGKKCGPLGGGFRTIFGDGERGLLSGNEKITMQNLNDRLATYLDKVRALEEANKELEFKIEERYEKFGREAG
GRGAKRDYSKYFQTIEELKKQILDATTKSASFVLQNDNVRLAADDFKLKYETELALRQGVEADSSGLRRVLDDLTLSKTDLEMQVETLREELAY
LKKNHAEEMKGLQSASGSDVSVEMNAAPGTDLTKLLNDMRAQYEKLAGQNRREAEAWFHDKSESLQQQISMVTDESNTAKSEMTELKRTLQALE
IELQSAVALKNSLEGSSGETETNYRAQLSQTQVQISALEARLLRIRTEMENQSTEHNQLLDVKTRLENEIETYRRLLDEEGGHGGGSRTGNPVK
EPTKTFLYKTIVEELVDGIVVSSQIRNVHQRSA

>Platypus_K24L4
MSCLARAAARSGGSGSVRVSGGGSSFSSGSRCGMGGGSAYGSGGAGSCRMGGGAGGGFGGSFGGGCGSSVVGGYGGGFGGGAGGGFGGGAGGGH
SAGASGGFYTYGGGVGGGAGGGFGGGAGDGGLFSGGEKQTMQNLNDRLASYLDKVHALEEANTDLEVKIKQWYEKFGPGSTVPGSGRDYSKFYP
IIDDLRNKIMAATIDNAGMVLQIDNARLAADDFRLKYENELYLRQSVEADINGLRKVLDDLTMTRSDLEMEIESLTEELAYLKKNHEEELKGMQ
GSSGGDVTVEMNAAPGTDLTKLLNDMRAQYEALAEQNRKEAEDQFNKQSASLHAQISTDVGAASSAKSEITELKRTLQALQIELQSLMTMKSSL
EGTLAETESGYVMQLSQIQHQISSLEEQICQIRGETECQNAEYEQLLNIKTRLEMEIETYRRLLDGEAGGSGLASSSTSSSLGGGSRSSVVVDS
RVGQGRDANKTRVTKTIVEELVDGKVVSSQVSNVSEMKVK

>Platypus_K24L3
MSCLARAAARSGGSGSVRMSGGGSSFSSGSRCGMGGGSAYGSGGAGSCRMGGGAGGGFGGSFGGGCGSSVVGGGFGGGFGSGFGGGAGAGFGGG
HGAGAGGGFYTYGGGVGGGAGGGFGGGAGFGGGAGDGGLFSGGEKQTMQNLNDRLASYLDKVRALEEANTDLEVKIKQWYEKFGPGSTVPGSGR
DYSKFYPIIDDLRTKIIAATIENAKIISPIDNARLAAEDFRLKYENEMYLRHSVEADINGLHKVLDDLNMSTSDLKMQFESLTEELAFLKKNHE
EEMKSMQGTTTGDVTVEMNAAPGTDLTKLLNDMRAQYEALAEQNRKEAEDQFNKQSASLHAQISTNAGATSSAKSEITELKRTLQALQIELQSL
MSMQSSLESTLADTEAGYVMQLSQIQHQITSLEEQICQIRGETECQNAEYEQLLNIKTRLEMEIETYHRLLEGEDPHIQSNQSTSSSPSKMLPH

>Platypus_K24L2
MSCLARAAARSGGSGSARVSGGGSSFSSGSRCGMGGGSAFGSGGAGSCRMGGGAGGGFGGSFGGGCGGSVVGGGYGGGFGSGLGGGAGAGFGGG
QGGGAGGGFYTYGGGVGGGAGGGAGGGAGFGGGAGDGGLFSGGEKQTMQNLNDRLANYLDKVRALEEANTDLELKIKEWYEKFGPGSTAPGSGP
DYSRFYPIIDDLRTKIIAATIENAKIISPIDNARLAAEDFKLKYENEMYLRHSVEADINGLHKVLDDLNMGTADLQMQFEGLTEELDFLKKNHE
EEMTGVQGSSGGDVTVEMNAAPGIDLTKLLNDMRAQYEALAEQNRKEAEDQFNKQSASLHAQISTDVGATSSAKSEITELKRTVQALQTELQSL
MSMQSSLESTLAETESGYVMQLSQIQHQITGLEEQICQIRGETECQNSEYEQLLNIKTRLEMEIETYHRLLDGEAGGASSGAAVSSSAGAASSS
AGAASRSTASRAAVSFDGASGASLARDPNKTRVTRTIVEDLVDGKVVSSQVSNVSEVRVK

>Platypus_K24L1
MRNLNDRLASYLDKVRALEEANTDLEIKIKQWYVNLGPGSRDPASGRDYGKFYLIIEDLKNKILTATVDNASVILPIDNARLAADDFRMKYENE
LYLRQSVEADINGLRKVLDDLTMTRSDLEMQIESLTEELAYLKKNHEEIFTDVGEASSAKGEMTELKRTLQALEIELQSLFATQSSLEGTLADT
ESGYVLQLSQIQHQISSLEEQLCQIRGESECQNAEYEQLLNIKTRLEMEIETYRSLLDGEGGGSRISGPKSTGSKSSIPPEGKSIQGRDSNKTR
ITRTIVEDLVDGKVVSSQVSNISEVRVQ

>Platypus_K25
MSLRLSSGSRRICSRPPAGSVRLSASGAGFGAGNGCGVPGIGSGFSCAFGGGSSGGNLGSASCSAFVGTEGGLLSGNEKVTMQNLNDRLASYLD
NVRALEEANADLEQKIKGWYEKFGPGSCRGLDHDYSRYFPIIEDLRNQIISSTTCNASAVLQIDNARLTADDFRMKFENELALHQTVEADVNGL
RRVLDELTLCRTDLEIQYETLSEELTYLKKNHEEEMKALQCAAGGNVNVEMNAAPGVDLTVLLNNMRAEYEDLAEQNRRDAEAWFNEKSASLQQ
QISEDVGATTSARNELTEMKRTLQTLEIELQSLLATKHSLECSLTETEGNYCVQLSQIQAQIGALEEQLQQVRTETEGQKLEYEQLLDIKVYLE
KEIETYCRLIDGEDGACKSGSFKSTGYASGVLASQLKEPSKTIVVKKVLEEVDQRSKVLTTRLHSLEEKPQSK

>Platypus_K26
MSFRLSSGSRRICSRAGAGPGRLSNTGTGLGAGNGCGVAGVGNGFPCAFGGGPSGGGFCGGGVGGLGGGACSAFIGSEGGLLSGNEKVTMQNLN
DRLASYLDNVRALEEANADLEQKIKGWYEKFGPGSCRGLDHDYSRYFPIIEELKNEIVSVTTCNATIVLQNDNARLTADDFRLKYENELALHQS
VEADINGLRRVLDELTLCTTDLEIQYETLTEELTYLKKNHEEEMKVLQCAAGGNVNVEMNAAPGVDLTVLLNNMRAEYEDLAEQNRRDAEAWFN
EKSASLQQQITDDAGATATARNELTELKRTLQTLEIELQSLSAMKHSYECSLSETEGNYCIQLTRIQAQIEALEDQLQQIRTESEGQKLEYEQL
LDIKIYLEKEIETYCCLIDGEDKACKSSCLKSKGYVPGGSGSQSKDSTQTTVVKTVVEELDQRGKVVSLRVHSVEEKSSKISNITMEQRVPS

>Platypus_K27
MSLRFTSASRRVGTCGGAGSVRPSAGGPGFGAGNGFPCAFGSSGGNPGSASCSAFVGSEGGLLSGNEKVTMQNLNDRLASYLDNVRALEEANAD
LEQKIKGWYEKFGPGSCRGLDHDYSRYFPTIEDLRNQIISATTSNASVVLQNDNARLTADDFRLKFENELALHQSVEADINGLRRVLDELTLCR
TDLEIQHETLSEELTYLKKNHEEEMKVLQCAAGGNVNVEMNAAPGVDLTVLLNNMRAEYEDLAEQNRRDAEAWFNEKSATLQQQISDDAGATTS
ARNELTEMRRTLQTLEIELQSLLATKHSLECSLTETEGNYCVQLSQIQAQIGALEEQLQQVRTETEGQKLEYGQLLDIKVCLEKEIETYCRLID
GEDGACFKSIGYGSAGPAHQAKDSSRTTTVKTVVEEIDPRGKVLSTRVHSVEEKSTKINIKTEPRMPS

>Platypus_K28
MSLRFSAGSRRGCSRPGAGCGNSNFSCSLGGGPAGGSFGSSLNGPGGNHIGGSLGNASCSAFVGSEGGLLSGNEKVTMQNLNDRLASYLDNVRA
LEEANADLEQKIKGWYEKYGPGSCRGLDHDYSRYFPVIEDLRNQIVSSTTCNANVVLQIDNARLAADDFRLKFENELALHQSVEADINGLRRVL
DELTLCRTDLEIQYETLSEELTYLKKNHEEEMKVLQCAAGGNVNVEMNAAPGVDLTVLLNNMRAEYEDLAEQNRRDAEAWFNEKSATLQQQISD
DAGAATSARNELTEMRRTLQTLEIELQSLLATKHSLECSLTETEGNYCVQLSQIQAQIGTLEDQLQQIRTETEGQKLEYEQLLDIKIHLEKEIE
TYCRLIDGDGESCFQSKSYVPGGLGSPSKDLSKTTLVKTVVEEIDQRGKVLSSRVHSIEEKSSKLSAIKTEQRTPF

>Platypus_K10L2
MSYRLGSTKDFSSSRSGGGGRSSVKVSSSRGSSSVGGGFSLGGGSAGGSYCYGGSSGGGFCSGGFGGGSGGGFGGGMLGGGGYGGGLCGGFGGG
SYGGGGFGGGGYGGSFGGGSCGGGGFGGGFGGGDGGLLSGNEKVTMQNLNDRLASYLGKVRELEESNFELERKIKEWYEKYGSSTQREPRDYSR
YYQVIEDLKNQIITLTTDNASVLLQNDNARLAADDFRLKYENEVALRQSVEADINGLRRVLDELTLTKADLEMQLESLSEELAYLKKNHEEEMR
DLQNVTTGDVNVEMNAAPGVDLTELLNGMRAQYEQLAEQNRRDAEAWFNEKSKELTTEINASVEQMSGHKSEITELRRTLQALEIELQSQLALK
QSLEASLAETEGRYCVQLSQIQVQITSLEEQLQQIRAETECQNAEYQQLLDIKTRLENEIQTYRSLLEGEGGSSGGGYGGRGGGSGGNYGGSSG
GGSSPGGGYSGGSGGGQQGNSGFKVSSSGSRSSGGGSTGESTTKGPGTRF

>Platypus_K10L1
MQNLNDRLASYLDKVDELGQANKALETKIKEWYEKFGQGAVNAPARDYSKYYSVIEGLKTQITQVAQDHATVVLQIDNARLTADDFRLKFESEA
ALVQSVEVDIAGLRRVLEELTLTKTDLDLRTESLTEELAYLKKNHEEEIKSDSKTAPGDVSVEIDAAPGIDLTLLLNRMREQYESLAEQNRQEA

EARFTERSEELSVQISTSVEETSTNKSEMAELRRTLQTLELELKSQIPLKQSLESSLAETEGNFCAQLSQIQVLVGSLETQVQQIREETECQNA
EYEQLLDIKIRLESEIETYRQLLDREGR

>Platypus_K20L2
MPISRGKFIRSNSNEAIFINPVFRMPAKQVFYTPSVYGGAGGWGTRISTSTTQIQYGEGLGAGKLLTGNEKGTMQNLNDRLANYLEKVRSLQQA
NSKLEVQIRQWHEKNSTPLGRDYRSYYKAIEELQEQIKNAQINNAQLVLQMDNAKLAAEDFRIKYETELGMRLSVENDIQGLNKVTDDLTLKKA
RLESEIENLTEELVLLRKNHEEEVDTLRKHLGATVKVEVDAAPGPDLGAIINEMRDKYESLAKKNIQKAKERYETQTIELETQITLNNRELETS
TTQVKELRRKCQNLEMELQSLLSQRDSLDLTLAETNTRYEARLAQLQAVLKALKDQLIQVRSESEKQRDQFNTLLDVKNRLEQEIITYRRLLEG
EDIVTLDSETTTLEERDAKKIRKIRMVVEEVVDGKIVSSKTKEMEETLESGQMISSHTPTANLESLKF

>Platypus_K12L
MWTRQGRRPLPQVHALEEANTELEGKIRNWYEARGPGAIALGTGGDFSKYDPTIEELRNKIISATIKNAQMVLQIDNARLAADDFRLKYEGELA
LRQSVEADINGLRRVRDELTLARADLEMQIEGLNEELIYLRKNHEEELQSFQAGGPGQVSVEMDAAPGIDLTKLLNDMRGRYEAMAERNRKEAE
AWFIEKSGDLRKEISTHSEQLQSSRSEVTDLRRTLQNLEIDLQSQLAIKKSLEDTLAETEGGFCSQLSQMQYQIGNVEAQLTQVRADTERQNSE
YRQLLNIKARLEMEIETYCRLLEGGLAGDSSDENSPPASPRSQAQSLDSSKDSTKTRKIKTIVEEVVDGKVVSSQMREVEEQM

>Platypus_K20L1
MPFIHRRHFRDDEDICISPMYRVPGRHLYYALSVYGGAGGWGTRISTGPTPFQYGKEVGMGRLLLGNEKLAMQNLNDRLANYLEKVHSLQQANF
KLEAQIRQWYQKNSTPVDEDFSSYHQMIEELQNQIQNAQKNKVQWILKIDDVKLKEENLRIKFDNERAMCLSVEGDIQKWTKAIDDLTLMKARL
EMQVENLLKERVVLKKNYQEEANNLRKHLGATINVKLDAAPGPDLGAIINEMREKYEKLAEKNIKEAKWRLEKLMIELEKQVVDNDKELEVNMI
KLKELRHKRQNLGIELQSQLCQKKSLEAILGRPKTHCEPELALIQEKLSIFKAQLIQIRSESERQKDEFKILLDIKIRLEKEITTYRSLLERGA
VGPD

>Platypus_K23
MSSCYGSFQAPPRGGWARPGKIWRAPSVHGGAGGICISVSSTRPFCGASWERGYGGGFGGGEKRGLLGGNGKETMQNLNERLAAYLEKVRSLE
AANTKLESRILEWHKKRDPDKKQNYTVYEENINTLQEQIVDGKLNNAKIVLQIDNARMAVDDFSFKYENELSFTNDMEVEVEALRKILDDLTII
TTDLEMEVEGMREELILMKKKHEQEIATHCMPNDFKVNVKVNATPGEDLVKILEDMRKEYEVIITKKRKDLDTWYKGKSASVSQDTGIEVVKTN
KSDINELRRTFQALEINLQAQRSTKSALEAMLDETKARNSCQLQNMQQVISHYETQLAQLRHDLERQNNEYKILLGIKTHLEKEIATYRQLMEG
EGNGGMDDSKSTRKESTKSAAPKIKAIVQDSVNGRVVLSQVSEIQ

>Platypus_K39
MTTILFEDNWTSSHLTSNCNPPSHLRSCGWYGEGYFNGNEKETMQSLNARLASYLDKVRCLEQENANLECKIQEFCQNQAPAVCPDYLAYFRMI
EELQQKILCTKAENSRLVTQIDNTQLASDDFRTKYKVELSLRQLVEADINGLQRILGKLILCKADLEMQVESLKEELLCLRNNHTEEVNSLQCQ
LGNRLNIQVDIAPSVDLNQALEKMRCQYESLLETNRRDIQEWFNTQREELNQQIASSSGQLQCCQSESIELRRTVNALEIELEAQHSLRNSQEN
ILTEVKARYSSQLAQVQALIDNVEFQLAEIRCDLERQNQEYQVLLDVKARLEGEISTYRSLLESEDCKLPSNPCARESTVSTCPPCKPCAIECT
APTQAACEPCNLCAPPASLGKCMPCKTCTPAPRIIVKIFTIIEEIRDGKVISTQKLVQPCVFYNPQFQV

>Platypus_K40
MTSACSSSCSPESCCRATDCPSVSTCSTDIPCLPATCGAARCQTPRFLCRSRLSSSCYLPVVGHCGWRDEWAFNSNEKETMQFLNDRLANYLER
VRCLEVENAELECKIREQCEQQIPFVCPDYQCYFNTVENLQQKILCTKAENSRLAVQIDNCKLAADDFRTKYEAELSLRRPVEADVGGLRRILD
DLTLCKSDLEAQLESLKEDLLCLKKNHEEEVNALQAQLGDRLSVELDTAPTVDLNKLLDEMRCQYETVVANNRREAEEWFAVQTEELNQQQLSS
AEELHCCQSEIIELDRTANALEIELEAQESLKESLECTLSETEDRYSSQLAQMQCLIDNVEIQLAEIRCDLERQNQEYQILLDVKARLECEIAT
YQSLLENEDCKLPCNPCSAPNASCTFCDLSEPFVICTVENCQA

>Platypus_K33a
MPYNCSLPSFSCRSSCATRPCVPPSCQPCCSLPGARNIPANIGTCGWFCEGSFNGNEKETMQFLNDRLANYLEKVRQLERENAELEARIRQWCQ
EQVPFVCSDYQSYFRTIEELQQKILLTKSENTRLVVQIDNAKLASDDFRTKYQTELSLRQLVEADINGLRRILDELTLCKSDLEAQVESLKEEL
LSLKQNHEQEVNSLKCQIGERLNVEVDAAPTVDLNRVLNETRCQYEALVETNRRDVEEWFVKQTEELNQQVVTSSEQLQCCQSEIIELRRTVNA
LEVELQAQHNLRNSLENTLTETEARYSSQLAQVQCMITSVEDQLAEIRCDLERQNQEYQVLLDVKARLECEIATYRNLLESEDCKLPSNPCATT
NACGRAIGPCSVTPCAPCTPCMPRSRCGPSNSFVC

>Platypus_K33b
MPYNCSLPSFSCRSSCATRPCVPPSCQPCCSLPGARNIPANIGTCGWFCEGSFNGNEKETMQFLNDRLANYLEKVRQLERENAELEARIRQWCQ
EQVPFVCSDYQSYFRTIEELQQKILLTKSENTRLVVQIDNAKLASDDFRTKYQTELSLRQLVEADINGLRRILDELTLCKSDLEAQVESLREEL
LCLKKNHEEEVNSLKCQIGERLNVEVDAAPTVDLNMVLNQLRSQYEALVATNRRDVEEWFVKQMEELNQQVVSSSEQVQGSQSEIIELRRTVNA
LEVELQAQHNLRNSLENTLTETEARYSSQLAQVQCMITSVEDQLAEIRCDLERQNQEYQVLLDVKSRLECEIATYRNLLESEDCKLPSNPCATT
NACSTSCGPRPRCC

>Platypus_K34
MPYNCSLPSFSCRSSCTTRPCVPPSCQPCCSLPGARNIPANIGTCGWFCEGSFNGNEKETMQFLNDRLANYLEKVRQLERENAELDARIRQWCQ
EQVPFVCPDYQSYFRTIEELQQKILFTKSENSRLVVQIDNAKLASDDFRTKYETELSLRQLVEADINGLRRILDELTLCKADLEAQVESLKEEL
LCLKRNHEEEVNTLRCQIGDRLNVEVDAAPPVDLNRVLNETRCQYEALVETNRRDVEEWFIKQTEELNQQVVSSSEQLQCCQSEIIELRRTVNA
LEVELQAQHNLRNSLENTLTETEARYSSQLAQVQCMITNVEAQLAEIRCDLERQNQEYQVLLDVKARLECEISTYRNLLESEDCKLPSNPCATT
NACGRPIGPCAPNPCAPCVPCTPCVPRSRCGPCTSFVR

>Platypus_K31
MASPCCRSTVGTTGSLRSGRFSEARAASMCLTAALANVNRPRVGVTPLGRPSLCVPASCRAACPLPGSCNIPANIGNCGNFCEGILNGHEKETM
QFLNDRLANYLEKVRQLEQENAELETKIREWSKCQVPFVCPDYQCQFRTIEELQQKILCTKSENARLVVQIDNAKLAADDFRTKYETELSLRQL
VEADIGGLRRILDDLTLAKSDLEAQVESLKEELLCLKKNHEQEVHNLQCQLGDKLRIELDTAPPVDLNRVLGEMRCQYEAMVETNRNDVEQWFT
AQSEGLSMQVMSCSEELQCCQSEILELRRTVNALEIELQAQHKMRNCLENSLAESEARYSTELAQMQFLISNTEDQLAEIRCDLERQNQEYQVL
LDVKARLESEISTYRGLLESEDCKLPCHPCATPAMCTPCAPPACTPCKPCPSSSRSACGPCAPPRC

>Platypus_K37
MTSSCIRTSGSLSSLKGSGRLPGGINISSIDVGGRPGAGVKAASMCLTAALANVNRPRVGVTPLGRPSLCVPASCRAACPLPGSCNIPANIGNC
GNYGEGILNGHEKETMQFLNDRLANYLEKVRQLERENAELEAKIREWSKCQVPFVCPNYQSYFRTVEELQQKILCNKAESARLIVQVDNAKLAA
DDFRTKHESELALRKLVEADMSGMHRILDGTTLAKCDLEAQLESLKEEQLSLKTNHEQEVHNLQCQLGDKLRIELDTAPPVDLNRVLGEMRCQY
EAMVETNRNDVEQWFTAQSEGLSMQVMSCSEELQCCQSEILELKCAVNALEIERQAQHKMRDCLENSLAESETRYSTELAQMQFLISNTEDQLA

EIRCDLERQNQEYQVLLDVKARLEGEIATYRNLLESEDCKLPCNPCSTPASCGPPPGTPCGPRPGGAPRPPCGPRPACGGMFASAPKESLVGFS
AMLLLQFPFEMLKASLLTGK

>Platypus_K38
MASSTGRPKSCIKNPSSRSGCGPAEGGRRSGGFLVQSNQPSGSRSGACPIGSCPPNRVQPRPNGPSPGSARSFPVASGRPAIPGPRSRDLPAHA
GPGGWVGEGVLPGGEKETMQFLNDRLANYLDQVRQLERANAELEAKVSQWGACQIPTMCSNYQSYFRTIEELQQKILCSKAENARLATDVDNAK
LAADDFRNKYETELSLRQPLEADIGGLRRILDELTLATCDLEAQVESLKEELLCFKKGHEQELYSLQCQMGDKLQVELDTTPPVDLNRVLGEMR
CQYEAMVESNRNDVEQWFTAQSEGLSMQVMSCSEELQCCQSEILELRRTVNALEIELQAQQKMRDCLDNTLAETDTRYSTELGQMQFLISKTED
QLAEIRADLERQNQEYQVLLDVKARLEGEIATYRNLLESEDCKLPSHPCATSSSGPPSGTAPPCPPRAPGPPSTPRPSGTPRNGHRPGAQCSGR
SYSSGRF

>Platypus_K32
MASSFCTSTGSVKGWARAPSVCSTGATYRPETYLSYFCQPLGCMSSPCRTGGSLPKSFLATTCRSGSRLPGSCPLPCSVGGSGWYCDASLQGSE
KETMQMLNDRLANYLEKVRQLERENTELDAKIRDSCQTQMPTMGPDYQSYFRTIEELQQKVLCTKADNARLVVQIDNAKLAADDFRTKYETELT
LRQLVEADINGLRRILDELTLCKADLEAQVESLKEELLCLRKNHEEEVNALRCQLGDRLNIEVDAAPPVDLNRVLDEMRGQYEGLVENNRRDVE
QWFNTQMDELNQQVVTSSEQLQCCQSEIIELRRTVNALEIELQAQHSMRNSLESTLAETEGRYGSQLAQMQGLISNVEAQLADIRCDLERQNQE
YQVLLDVKARLEGEIATYRRLLESEDCKLPCHPCTAPCVPCGPRPVCVPCAPCGPGRY

>Platypus_K36
MQFLNDRLANYLEQVRHLEQENAELEAKIRQWGEAQIPYVCPDYQSYFRTIEELQQKILLTKADNARLVLQIDNAKLAADDFRTKYETELNLRQ
LVEADINGLRRLLDELTLCKADLEAQVESLKEELLCLRKNHEEEVNALRCQLGDRLNIEVDAAPPVDLNRVLEDMRAQYESLVENNRRDVESWF
TTQTEELNQQVVTSSEQLQCCQAEIIELRRTVNALEIELQAQHNMRNSLESTLAETEGRYGSQLAQMQGLISNVEAQLAEIRCDLERQNQEYQV
LLDVKARLEGEIATYRRLLEGEDCKLPPHPCTPVPTLRTPCTPASQVNTQIRTITEEIRDGKVISSRELVQHRPM

>Platypus_K13
MSCRLQTSSSGLGGSCRLGGGRSVSSSSSRFVTSGSGGGMSCGFGGGAGGGFGGGFGGGYGGGFGGGACGAGGGFGGGFGGGFDFGGALGGADG
GLLSGNEKVTMQNLNDRLASYLDKVRALEEANADLEQKIRDWHLKQSPSSPERDYSHYYKTIEDLRDKIMAATIDNNRVVLEVDNARLAADDFR
LKYENELVLRQSVEADINGLRRVLDELTLAKTDLEMQIESLTEELAYLKKNHEEEMKEFSNQLVGQVNVEMDAAPGVDLTRVLSEMREQYEAMA
EKNRRDAEAWFHSKSEELSKEVASSTELIQTSKTEVTELRRTLQGLEIELQSQLSMKAGLEATVAETECRYAMQLQQIQGLIGSIECQLSELRS
EMECQNQEYKMLLDIKTRLEQEIATYRSLLEGQDSKMPGFTSKNGAGVTTGISGSVAGPGRRPSVEPRRP

>Platypus_K19
MAFRARNAYVSTAYGGSGPSGRFGGATFRAPSIHGGSGGRGVSVSSARFVSSGAGGLGAAAFGGGFGGGDGLLAGGEKATMQNLNDRLASYLDK
VRALEEANADLEVKIRDWYLKQGPGPARDYSAYYRTIEELRDKILGATIENSKLVLQMDNARLAADDFKTKYETELALRMNVETDINGLRRVLD
ELTLARADLEMQIEGLKEELVYLKKNHEEEMNALSGQVGGQVSVEVDSAPGVDLGKILTDMRDQYEEMAEKNRKDAEAWFNSKTEELTREVAVH
TEQLQTSKSEVTDLRRTLQGLEIELQSQLSMKAALEGTLAETEGRYGVQLAQIQALISSIEAQLGDVRMDSERQNREYKLLMDIKSRLEQEIST
YRNLLEGQDAHYTNVPSARGL

>Platypus_K9
MSSRQSTSYSSKSRSGGGGGGGGRGLSSSSSSVRFGSGGGGRFCSSGSSYGGGSFGGGSFGGGACGLGGGSYGSSFGGGSMYGGSGSCGGFGGG
YSGGYGGGFGGGAGGGFGGGFGGGFGGGLGGGSGGGFGGGFGGGFGGEDGGLLSGNEKVTMQNLNDRLASYLEKVRALEEANADLEQKIHDWYQ
KQSPSSPTRDYSHYYKTIEDLRSQIITTTVENNKIVLAIDNTRMTVDDFRMKYEMELNLRQSVEADVNGLRHVLEELTLSRSDLEMQLQSLQEE
LEALKKNHEEEMKQMTGQRSGEVSVEMNAAPSIDLTKALNDMRQEYEMLNAKNLKEIEDQYEAQMNKMHQQVTSHSQEIQTTSSQVTELRRTLQ
GLEIELQSQLSTKAALEQSLEETKGRYCVQLAHIQEQIGALEAQLSEIRGETECQSQEYNVLLDIKARLEQEIATYRSLLEGGQQDIGNASGGS
GRGSGSGVLVGGGGSGGIRGGSFGGGSGTVGGGSGGGSSSSFGGGKSSQAQGSVIPPQPPVQHEDRHGSLKRY

>Platypus_K14
MTSCSRQYSSSSSMKGSCGLGGGSSRVSSVLAGGSCRAPSAYGGSGFGGMSVTSSRFSSGGACGVGGGYGSGFGGGSFGGGLGSGFGGGLGGGF
GGGLGGGFGGGYGGGFGGGLGGGFGGGDGLLAGGEKVTMQNLNDRLASYLDKVRALEEANSDLEIKIRDWYQKQRPTEARDYSPYYKTIEDLRN
KILTATVDNASVLLQIDNARLAADDFRTKYETELNLRMSVEADINGLRRVLDELTLARADLEMQIESLKEELAYLRKNHEEEMNALRGQVGGDV
NVEMDAAPGVDLSRVLSEMRDQYEKMAEKNRKDAEDWFFSKTEELNREVASNSELVQSSKTEISELRRTMQNLEIELQSQLSMKASLEGSLEET
KGRYCMQLSQIQGLISNVEEQLAQLRCEMEQQNQEYKILLDVKTRLEQEIATYRRLLEGEDAHLSSQHTSSSQYTSGPQSTRDVTSTSRQVRTI
VKDVHDGKVVSSHEQVLRTKN

>Platypus_K16
MTSCSRQFSSSGSTKGSCGLGGGSSHVSSILTGGSCRVPSTCGGSGFKGMSVTSSRFSSGGACGVGGGYGSGFGGGSFGGGLGSGFGGCYGGSF
GGGFGGGFGGGFVMGDGLLAGGEKVTMQNLNDRLASYLDKVRALEEANTDLEVKIRDWYLKQRPSEDRDYSPYYRTIEELKNKILTATLENAQP
LLQIDNARLAADDFRNKYEHELVLRQNVEADINGLRRVLDDLTLTRAELEMQIEGLKEELSYLKKNHEEEMRALRGQVGGDVSVEMDAAPGVDL
SRILSEMRDQYENLAEKNRRDAEAWFLSKTEELNQQVTSNSQQVQSSKSEVSELRRLFQSLEIELQSQLSMKASLEGNLEETKGRYCMQLSQIQ
CLIGNVEEQLAQLRCEMEQQNQEYKILLDVKTRLEQEIATYRRLLEGEDAHISGQQISNSQYLSGSQSTRDGSSSSRQVRTVVKDVRDGQVVSS
HEQVLRPKH

>Platypus_K17
MATTTTIRQYTSSSSSKGAGGLGGGSTRSSCRLPGGFSSSVSSSRLVSGGGGGGGYGAGYTCSLGGSFGGGLGGGFGGGDGLLAGGEKATMQNL
NDRLASYLDKVRALEEANADLEVKIRDWYQKQAPGPARDYSAYYLTIEDLKNKILTATVENANILLQIDNARLAADDFRTKYETELALRMSVEA
DINGLRRVLDELTLARADLEMQIENLKEELAYLRKNHEEEMNALRGQVGGEINVEMDAAPGVDLSRVLSEMRDQYEKMAEKNRKDAEDWFFSKT
EELNREVATNSELVQSGKSEISELRRNLQALEIELQSQLSMKASLEGSLQETESRYCVQLSQIQGLIGNVEEQLAQLRCEMEQQNQEYKILLDV
KTRLEQEIATYRRLLEGEDAHLASQYKPKEASLTTRQVRTIVEEVQDGKVISSREQVHQTTR

>Platypus_K18
MMNFAPSARSYRSSSSYRSSNVFRSSGSSGPRPSSAASIYAGAGGSGSRVSRVFVSGPGSPARFQPRPGALGSGPGLGLGLAGGPENDKQKMQG
LNERLASYLEQVRGLEADNHRLEAQIRQHLDNRAPHTQDWTPFFKTIEDLRAEIFAKSVENANLVLNIDNARLAADDFRIKYETELAMRQSVES
DIHGLRKVIDDTNMNRMQLETEIETLKEELLYMKKSHQEEVEGLQREIAGSGLTVEVDAPKTQDLGQVMAELRAKYEALAEENRKELDKYWSQQ
IEESTIVMTSRCEEVEKARGEVTDLRRSLQTLDIELDTLRNLKGNLEANLQEVEMRYRLQLDQQNALLMHLEAELAKVRAECQQQAQDYGALLD
VKSKLVNEIDMYRKLLDGNDDFSLRDALDSKSNSNPGIVHSFHKTTRTVVDGKVVSETSDSKVIRH

>Kiwi_K12

MALSVRTSGSGFSGGFGGSSGGGFGSSLSSGFGGGFGSGLGGSYGSGLGSGFGGGFGSGLGGSYGSGLGSGFGGGLGSGFASGSGAGFGGGFGT
GGGGDGGLLSGSKKETMQNLNDRLAAYLDKVRSLEEANTDLERKIREWYEKNGPGGGIPGSGNDYSKYYPIIEDLRSKIINATIDNARIILQVD
NARLAADDFRLKYENEVALRQSVEADINGLRRVLDELTLTRADLEMQIESLNEELAYLKKNHEEELQGFQGSTAGQVSVEMDAAPGIDLTKLLN
DMRGQYEAIAEQNRKEAEAWFNEKSGELKREISTNTEQLQSGKSEITDLKRTLQSLEIELQSQLAMKKSLEDTLAETEGGYCAQLSQIQLQIGN
LESQLFQVRADMERQNAEYQQLLDIKTHLEMEIETYRRLLDGEFVKIKTIVEEVVDGKVXKTRKIKTIVEEVVDGKVVASHVKEVEEKM

>Kiwi_K20
MAFSSQSSQWGSTPRFYPPTPSVTGLTVRKPATQQFQAPSVYGGAGGYGTRISTSTNYRQGFGGKFSNNFQLNLTGNDELLTGNEKTTMQNLND
RLASYLEKVRSLERANSQIEKQIREYYEKNTSDISHDHSPYFKIIEELQNKIGVAQLENARLVLQIDNAKLAADDFRLKFENEHLLLQNVQNDI
NGLFRVRDDLTLTKSDLESQIESMNEELVFLKKNHEEEVDRLRKQVGGSVNVEVDAAPGIDLATIMENMRLQYEGMAEKNREEAKERFEKQIDE
LNQEVAINTEQLQNQKTEITERRQTFQGLELELQSHLNMKKSLEDTLAETEAHYSYQLAQIQEAAANVEAQLRQIRADMAGQNNEYSILLDIKT
RLEMEIATYRRLLEGEDSGHFQVDIATAEAEKELSKIKKIKTIVEEVVDGKVVSSEVKEIEEKI

>Kiwi_K23
MSTSQGPFQFFSGSLRGSSGCGLAQQGSSYRPSSADGGASSTHIPSSSARPLWLATGGGASWGGFGEHHGESIFLGGNEKQTMQNLNDRLAAYL
DKVRSLEAANAQLESCIQEWHKTKSHSKRHDFNQYEQSITDMQRQIEDRKITNASILLQIDNANMASEDFRLKYEAEKSRRQGVQFDVENLRKE
LDNLTIVTTDLEMEIEGLREEHILRRKDHEEDMDAHCSSKDFKVNVKVNAAPPEDLAKILAEIREDYEAIIEKNRKSLDSWYKEQSAAMSLAAI
PNPEQLQSSENEIKDLTRTLQSLEIELQAQMSKKHMLEDTLADTRNYYALALQNMQQIISKCEEELSQVRRDIKHQNNQCKVLLGIKTRLEKEI
STYRLLLEGNVDGTTRKSESNAKEHAGLTNQKIKAIVHDSVNGQVVSSTINEIHQQA

>Kiwi_K36L
MTSKHYIQTVSSGSAKGSCGVGGGFTRISSVHSGGSSRIPGLSYGMIKVSDTSAKLGPSGLETCLSGSFLPTSYYGPGFAGWHDGGIFNSNEKE
TMQFLNDRLACYLEKVHHLEQENADLERKIRDWYDCQVPYVCTDFQAFYKTLEDLQQQILCAKTSNAKLVLQIDNARLAADDFRTKFESEQALR
QTLEADVSGLRRILDELTLCRADLEMQLETLKEELLCLKKNHEEEASPLRSQLGTRVNVEVDAAPSCDLNKVLTEIRCQYEALVEDNRKDVESW
FCTKMEELNQQVISSGEQLQHCQTEITDLQRTIQGLEIELETQLSMKSTLECTLQETENRYASQLVQLQSLITSKEAQLAELRCNMERQNQEYR
ILLDVKTRLECEISTYRRLLDGEDCKIPCFSRLTETAPVSPITTKIRTITEEIKDGRVISSHEQVQHCPPV

>Kiwi_K15
MTTSFRQSSSSTYGGGFGGGGSSSSRLCSIRTGGSCRAPSIHGGSGGRGVSVSSARYVSSVGSGYGGGLCSGLGSGYGGGYGGVSALQSGLGWG
ASCGFGGGAGFGGGAGFGGGLDFGGGGDGLLSGNEKITMQNLNDRLAAYLDKVRALEEANADLEVKIRDWYQKQAPTSPERDYSHYYKIIEDLR
DKILAATIDNSRIILEIDNARLAADDFRLKYENELFLRQSVESDINGLRRVLDELTLARADLEMQIETLKEELAYLKKNHEEEMKEYKNQLSGT
VNVEMDAAPGIDLTRILSEMREQYEALAEKNRKDAEAWFFTQTDELNREVATHTQQIQTSKSEITELRRTFQGLEIELQSQLSMKAGLEANLRD
TESRYCAQLAQIQTLITNVEEQLSELRCDMERQNQEYRMLMDIKSRLEQEIATYRQLLEGQDSQMSGWSSKEAHLSSGRVRISMEDSIDGKVVS
TREKKFP

>Kiwi_K19
MASYSYRQTTSSVSGGLGGSSARLGGGSFRAPSIHGGSGGRGVSVSSVRFVSSGLGSGVGGGYGSSFSSSFSGGFGGGYGGCLGSGDGLLSGNE
KTTMQNLNDRLASYLDKVRALEEANSDLEIKIRDWYQKQGPGPARDYSPYYKTIEDLREKILAATIDNSKIVLQIDNARLAADDFKTKFETEQA
LRMSVEADVNGLRRVLDELTLARTDLELQIENLKEELAYLKKNHEEEMSALGGQVAGQVSVEVDSAPGIDLSKILADMRDQYEHMAEKNRKDAE
AWFHTKTEELNREVAVNTEQLQSSKSEVTDLRRTLQGLDIELQSQLSMKAALENTLADTEGRYGVQLAQIQALIGNIEAQLAELRADMERQNSE
YKILMDIKTRLEQEIATYRQLLEGQESQMFSSLSATADKGDKLADGKKY

>Kiwi_K9L1
MSCSIKRTSSISYRSGGGGGHCGGASGRSSSASSRRYASSVIGGGSYGGGASSISYGGSLCAGGFGGGMGGGFGGGLGGGFGGGLGGGFGGGGD
VFLGGNEKVTMQNLNERLASYLDKVRRLEEENAQLEHHIREWYRKQAPSVSKDYSSYYQTIEQLQNQIISATVDNNKLLLEIDNSKMTADDFRM
KYESELVIRQSVEADINGLRNMLDELTLVRSSLESELEALQDELIALKRNHEEEMRQLQSQTGGDVSVEVNAAPGIDLTKTLNDLRNEYEQIIE
RNRREVEQWYEVKMEEVNRQVTSSNEDIQTSSHQLTELRREMQNLEIELQAQLSTKSSLENSLAETESRYSLLLQQIQGQINSVEEELASIRCE
MEGQNQEYKMLLGIKTRLEQEIAQYRALLQEGQQDLTTSQGAVQGGGGSSHSYSSTSYSHSQYGEKTGQSRVC

>Kiwi_K9L2
MSCSIKRTSSISYRSGGGGGHCGGASGRSSSASSRRYASSVIGGGSYGGGASSISYGGSLCAGGFGGGMGGGFGGGLGGGFGGGLGGGFGGGGD
VFLGGNEKVTMQNLNERLASYLDKVRRLEEENAQLEHHIREWYRKQAPSVSKDYSSYYQTIEQLQNQIISATVDNNKLLLEIDNSKMTADDFRM
KYESELVIRQSVEADINGLRNMLDELTLVRSSLESELEALQDELIALKRNHEEEMRQLQSQTGGDVSVEVNAAPGIDLTKTLNDLRNEYEQIIE
RNRREVEQWYEVKMEEVNRQVTSSNEDIQTSSHQLTELRREMQNLEIELQAQLSTKSSLENSLAETESRYSLLLQQIQGQINSVEEELASIRCE
MEGQNQEYKMLLGIKTRLEQEIAQYRALLQEGQQDLTASQGALQGGGRSSHSYSSASYSHSQSGEMTGQSRVC

>Kiwi_K9L3
MSCGTKSSTSIVKIGGGSSGGGGGGGCGGGGGGGSGSSGGRRSGGYSSVSSRRYTSSGGGGFSGRSYGGGGSRSSYGGGFSSGSCGRISRSSYG
GRMSGGSFGGGMGSGSMGGGFGSGGGGFGGMGGGYGSCLGGGGGFGGGGYSGGGFSGFGGSGGFGGGGLGGGGFGSGGYGGGSFGGMGFGEDTG
LLSTNEKLTMQNLNDRLASYLDKVRGLEEENAHLERLIREWYQKQGPTGTKDYSQYYRTIEELQNQIVGASVDLNKILLDIDNTRMTVDDFRLK
YETEYTLHQSVASDINGLRPLLDQLTLARSDLETQFESLKEELIYLKKNHEEEMRGLQTQSGGDVNVEVNATPGINLMEKLNEMRCEYERLIEN
NRREVESWYETKMEEVNQQVHSSGQEIQSSNQQISELRREYQSLEIELQSQISMVDSLQSNLEDTERRYNMQLQQIQGMIGPLEEELASIRCEM
ESQNEEYKMLLGIKTRLEQEIAQYRALLEEGQHNITIPGGGMGGGMGGGMGGGRIGGGGYSSGGGRGGGGSGGMSGGYGGGMGGGSGGMGGSSG
GGSCGIIGGGAGGAGGLGGGSGGGCSTIGGGGGGGRISGGVSSSHSYSSSSQSQSCSGGTQGYGRKSFD

>Kiwi_K9L4
MSSGFYGGGMSSGGYGGRMSSGGYGGGMGCGSMGGGFGSGGSGFGGGYGGSFGGGGGGFSGGSFGSGGFGGGNTGFLSNDEKLTMQNLNDRLAS
YLDTVRNLEKENTQLEQLIREWYQKQGPTGAKDYSHYYGTIEDLQNQLVTAAVETNRMLLDLDNTRMTAEDFRIKYETEYGLRQSVEADINQLR
PLMDNLTLGRSDLEMQFESLKEELISLKKNHEEEMKSLQTQSSGDVNVEVNAAPGEDLLKKLNDMRQEYESIIQKNREEVERWYESKMEEVSQQ
VHSSGQELESSNQQISALRREYQSLEIELQSELSMVNSLQSNLEDTERRYNMQLQQIQGMIGPLEEELASIRCEMESQNEEYKMLLGIKTRLEQ
EIAQYRALLEEGQHNITTSQGGAGDGSASGGRHGGISYSSGRGSTGGGSSWSSGGGSSGGRSMEGSYGRTSGGGGESGGIRGGACGRISGGGGG
SGGGGGGKSCLSHSSSSHSQSGNSCESQGGPLKLCLEGNKTLERGATMEEVSEAIKTMKSGRMEWDLGIIQFGNSKLAETLQRAGKISATLQVL
QAASAPAHNRPTLRRANQLLPTSHRQAGSRPTISEACKSSEELRPAGAFPCRNDLPPCKTLQEKWMPLSYNTVVVVTESTRHGLVPLQAGIQAC
MSHFPGYCANQSCSDGRAVVDASDLERIWDSLPQMD

>Kiwi_HAS1
MSCNIKETITVSSKGRSSGGSCIIGGGGGGGARISSYGIGSGRGFSGRSYCGGVNYGGGLSVGSLAGGSYGGGNCYGNGLGFGLGGGVVVGGLG
GDCLLSSCDEKVTMQNLNDRLASYLDKVKCLEKENAELECRIREWYATQGLSCEPRDYSCYYKEIEDLQNQIVCATIDNNKIILDIDNSRMAAD

DFRVKYETELALRQGVEADINGLRQVLDQLTLCRSDLEAQLESLREELCCLKKNHEEEMNCLRKQSTGDVSVEVNACPGPDLRQILEDLRCQYE
TLIARNRKEVEDWYECKIEEVNREVITSGQEVETCNNQVTELRRQLQALEIDLQAQLSQRNNLESSLAETECQYNTLLGELQNQITCVEQQLAE
IRAEIECQNQEYKTLLDVKCRLEQEIQTYRCLLEGGQQDLIHGGGIGVGTGVGGGVIRTSHTYTTTSSSHCQPQVPPCKTGDIQVTCRRICD

>Kiwi_HAS2
MSCTIKQTTGSLRGRTSGGSCVIGGGGSGGGVRISSVSSGRYTTCGIGGSRGFSGRSYCGGVNYGGGLSTGSLVGGNYGGGLGAGVLGGCSGIG
FSGGSVRFGGGIGGGIGGGIGIGLGGGVAGGGFSGDGILLSGDEKVTMQNLNDRLASYLDKVRCLEQENADLECRIREWYAKQGPFCEPRDYSC
YYKEIEDLQNQIVCATIDNNKIILNIDNSRMTADDFRVKYETELALRQGVEADINGLRQVLDQLTLCRSDLEAQLESLREELCCLKKNHEEEMC
CLRKQATGDVSVEVNACPGPDLRKILEEMRCQYETLIERNRKEVEDWYECKVSEISSALTFPG

>Kiwi_HAS3
MGKQLNAGSGTGGSFGATQQACPTFGFSGGSICTRGLGGGFTRANAGCGEGILLANNEKATMQNLNDRLASYLDKVRLLEGENTDLECKIREWY
AKVGPSCEPRDYSCFHKEIDDLQNQILCAAMEANKIVLNIDNNRMTADDFRVKYETECSLRQNVDADLCNLRPILDQLASCKTDLQLQFEALTE
EMCCLKTNHEEEMSCLRKQATGNVSVEVNACPGPDLRKILEDLRCQYETLMERNRKETEQWYACKVEEVNLEVITSSQEIESNNKQVTELRRQL
QALQINVQAQLNMKENLESSLAETECHYNKYLAELQSQISCVEQRLAEIRAEMECQNQEYKTLLDVKCRLEQEIQTYHCLLEGGQQDIVYADYR
QTRA

>Kiwi_K14L1
MATTVRQYSSSTSLKGFGGGSSRLSSARLGGGGYRAPSLSGRASSYSVSSRTVSGLGSGLGGGFGGTYCSSIGGGLGSGFGGGYGGSFGSGFGS
GFGGGYGGSFGSGFGGGFGGGYGGSFGGGEGILQAGEKATMQNLNDRLAAYLDKVRALEEANTDLEVKIREWYKKQAPGPDRDYSPYYRTIEEL
RSKVLVTTVDNANLLLQIDNARLTADDFRTKFETEQALRLSVEADINGLRRVLDELTLARADLEMQIENLKEELAYLKKNHEEEMNAMRGQVGG
EISVEMDAAPGIDLTKILTEMREQYETLAEKNRRDAEQWFFSKTEELNREVAINTEQLQSGKTEITELRRTIQSLEIDLQSQFSTKAALESSLA
DTEARYGTQLAQLQVLITSVEEQLAELRCDMERQNHDYRNLLDVKTRLEQEIATYRRLLEGEDAHMTSHMREVPVTTRQIRTIVEEVQDGKVIS
SREQVQQSPR

>Kiwi_K14L2
MATTVRQYSSSTSLKGFGGGSSRLSSARLGGGGYRAPSLSGRASSYSVSSRTVSGLGSGLGGGFGGTYCSSIGGGLGSGFGGGYGGSFGSGFGS
GFGGGYGGSXPSSTIVFLGPQMCVQGFCARMVTQPVCYLHGTRETANVCTRGLPCCTRCLAQAQEPLPSLAMATTVRQYSSSTSLKGFGGGSSR
LSSARLGGGGYRAPSLSGRASSYSVSSRTVSGLGSGLGGGFGGTYCSSIGGGLGSGFGGGYGGSFGSGFGGGFGGGFGGGYGGSFGGGEGILQA
GEKATMQNLNDRLAAYLDKVRALEEANTDLEVKIREWYKKQAPGPDRDYSPYYRTIEELRSKILAATVENANIVLQIDNARLAADDFRTKFETE
QALRLSVEADINGLRRVLDELTLARADLEMQIENLKEELAYLKKNHEEEMNAMRGQVGGEISVEMDAAPGIDLTKILTEMREQYETLAEKNRRD
AEQWFFSKTEELNREVAINTEQLQSGKTEITELRRTIQSLEIDLQSQLSTKAALESSLADTEARYGTQLAQLQVLITSVEEQLAELRCDMERQN
HDYRNLLDVKTRLEQEIATYRRLLEGEDAHISSQYSSAMSSHSGRDVMTTSRQVRTIVEEVQDGKVVSSREQVALTTR

>Kiwi_K18
MSFSRSTVYSSSYRPVSSQSIVSTRRFPPLSSAASVYAGAGGSGSRISISRTSSLGSSGGIYEARIGGYGAGIGGYGARMSSSLLLSGSGVVQN
EKETMQDLNDRLATYLDKVRSLEQENHKLEIQIRDFMQKKGPSSHDWSHYWEVIEDLRSKIFDATVDNARTVLQIDNARLAADDFRVKYEAELA
IRLSVENDIMGLRKVIDDTNMARLQLEGEIESLKEELIFMKKNHDDEVKSLQAQIANSVLTVEVDAPKSQDLGKIMAEIRAQYDALAQKNLEDL
EKQWGQQITESTIEITQSSKEVDTAHSTAVELRRTVQTLEIDLESLRNQRGGLEASLLEVENRYAMQMEQLNGLILRVEAELTQVRGELQRQAE
EYQALLNIKDKLEAEIATYRKLIEGGEEFSLRDALEKETTSTVQRSTQKIVDGKVVSETKELKVRTY

>Ostrich_K222
MELSQLLNEIRANYETLITRNQIKIIISTRTQLEEATIKRMDRDAEALKAARAELSEARRQWHHMQMEIESLHAVEKGLERSLRATEQQYHMQL
QNLEAEIESLEKELQEVRRGIEKQLQEHEILLNTRMKLEEEIATYRSLLEQEENRFRCSIPDQKDDKKPTTSKIAFTLPSSGVKKRETEKVELV
SKQAILDGNIMKESAEAHGTIQTEKVDEVIKEWEGSFFKDNPRLRKKSVSLRFDLHLAATDEGCLHTKQKTLPDIEVRLVMRRSCSIPSIKP

>Ostrich_K12
MAFSGRTSGGSRQFSSRSGLGGGSMRMSSSSSGGGFGGSGFGFGSGSGGGFGAASMLGSGSGFSGGFVGSSGGGFGSSLGSGFGGGFGSGLGGS
YGSGLGSGFGGGLGSGFGSGSGTGFGGSFGTGGGGSGGGGDGGLLSGSKKETMQNLNDRLAAYLDKVRSLEEANNDLERKIREWYEKNGPGAGI
PGSGNDYSKYYPIIEDLRNKIINATIDNARIILQVDNARLAADDFRLKYENEVALRQSVEADINGLRRVLDELTLTRADLEMQIESLNEELTYL
KKNHEEELQGFQGSTAGQVSVEMDAAPGIDLTKLLNDMRGQYEVIAEQNRKEAEAWFNEKSGELKREISTNTEQLQSGKREITDLKRTLQSLEI
ELQSQLAMKKSLEDTLAETEGGYCAQLSQMQLQIGNLESQLFQVRADMERQNAEYQQLLDIKTRLEMEIETYRRLLDGESAGQGVTFESSPLTG
SKSQTQSLDSSQDPTKTRKIKTIVEEVVDGKVVASHVKEVEEKI

>Ostrich_K20
MAFSNRSFQRGSSSHFHPPAPSVSGLTVRRTAIQKLQAPSVYGGAGGYGTRISTSTNYGQGFGGKFSNNFQLHITGNDELLTGNEKTTMQNLND
RLASYLEKVRSLEKANSQIEKQIKEWYEKNTSDIRHDHSPYFKIIEDLRNKIGVAQLENARLVLQIDNAKLAADDFRVKFENEHLLLQSVQNDI
NGLLRVLDDLTLTKSDLESQIESVNEELVFLKKNHDEEVDRLRKQVGGTVNVEVDAAPGINLATIMENMRKQYEEMAEKNREEAKEQFEKQTED
LKQEVAINIEQLQSQKREITERRQTFQGMELELQTHLNMKKSLEDTLAETEARYSHQLAQIQEAVANLEAQLRQIRADMAAQSNEYSVLLDIKT
RLEMEIATYRRLLEGEDSGCGSGTKLSKIKKIKTIVEEVVDGKVVSSQINEIEEAI

>Ostrich_K23
MSISQGPFQFFSGSLRGSSGCGLAQQGSSYRPSSADGGASSTHLSFSSARPLRLATGGGAPWGGFGEHHGESIFLGGNEKQTMQNLNDRLAAYL
EKVRSLEAANAQLESCILEWHKTKSHSKRHDFNQYEQSVTDMQRQIEDGKITNASILLQIDNANMASEDFRLKYEAEKSRRQGVQFDVENLRKE
LDNLTIVTTDLEMEIEGLREEHILRRKDHEEDMDAHRSSQDFKVNVKVNAAPPENLAKILAELRDDYEAIIEKNRKSLDSWYKEQSAAMSLAAI
PNPEQLQSSENEIKDLTRTLQSLEIELQAQMSKKHMLEDTLADTRNYYALALQNMQQIISKCEEELSQVRHDIKHQNNQYKVLLGIKTRLEKEI
STYRLLLEGNVDGTTRKSESNIKGKSPLFGSL

>Ostrich_K15
MTTVYRQSSSSTYGGGFGGGGSSSSRLSSIRTGGSYRAPSIHGGSGGRGVSVSSARYVSSVGSGFGGGLCSGLGSGYGGGYGGGSALQSGFGWG
AASGFGGGAGFGGGLDFGGGDGLLSGNEKITMQNLNDRLAAYLDKVRALEEANADLEVKIRDWYQKQAPTSPERDYSHYYKIIEDLRDKILAAT
IDNSRIILEIDNARLAADDFRLKYENELFLRQSVESDINGLRRVLDELTLARADLEMQIETLKEELAYLKKNHEEEMKEYKNQLSGTVNVEMDA
APGIDLTRILSEMREQYEALAEKNRKDAEAWFFTQTDELNREVATHTQQIQTGKSEITELRRTFQGLEIELQSQLSMKAGLEANLRDTEARYCA
QLAQIQALITNVEEQLSELRCDMERQNQEYRMLMDIKSRLEQEIATYRQLLEGQDSQMSGWSPKDDSIAVSLPDFSDIARNIAYLFDSHAETES
PFLLSEHILCAKTNNAKLVLQIDNARLGADDFRTKIPCFPRVTDSAPVSQITTKIRTITEEIKDGRVISSHEQVQHCPPI

>Ostrich_K19

MASYSFRQTTSSVSGGLGGSSVRLGGGSFRAPSIHGGSGGRGVSVSSARFVSSGLGSGVGGGYGSSFSNSFSGGFGGCLGSGDGLLSGNEKTTM
QNLNDRLASYLDKVRALEEANSDLEIKIRDWYQKQGPGPARDYSPYYKTIEDLREKILAATIDNSKIVLQIDNARLAADDFKTKFETEQALRMS
VEADINGLRRVLDELTLARTDLELQIENLKEELAYLKKNHEEEMSALGGQVAGQVSVEVDSAPGIDLSKILADMRDQYEHMAEKNRKDAEAWFQ
NKTEELNREVAVNTEQLQSSKSEVTDLRRTLQGLDIELQSQLSMKAALENTLADTEGRYGAQLAQIQALIGNMEAQLAELRAEMERQNSEYKIL
MDIKTRLEQEIATYRQLLEGQESQ

>Ostrich_K9L1
MSCSIKRTSSTSYRSGGGAGLCGGGSSRSSSVSARRYASSVIGGGSYGGGACSISYGGPVCAGSLAGGFGGGLGAGFGAGLGAGFGAGGDVILS
GNEKVTMQNLNERLASYLDKVRRLEEENAQLEHYIREWYRKQAPSVSKDYTSYYQTIEQLQNQIISATVDNNKLLLDIDNSKMTADDFRMKYEN
ELVIRQSVEADINGLRNLLDDLTLVRSSLESELESLKDELMALKRNHEEEMRQLQSQTGGDVSVEVNAAPGEDLTKTLNDLRNEYEQIIERNRR
EVEQWYEVKIEEVNQQVTSSSQDIQTSSHQLTELRREMQNLEIELQAQLSTKNSLENSLAETESRYGCLLQQIQGQINSVEEELASIRCEMESQ
NQEYKMLLGIKTRLEQEIAQYRALLQEGQQDIVVSQGAVQGGGGSSHSYSSTSYSQYGDKTGQSRVC

>Ostrich_K9L2
MSCSIKRTSSTSYRSGGGAGLCGGGSSRSSSVSARRYASSVIGGGSYGGGACSISYGGPVCAGSLAGGFGGGLGAGFGAGLGAGFGAGGDVILS
GNEKVTMQNLNERLASYLDKVRRLEEENAQLEHYIREWYRKQAPSVSKDYTSYYQTIEQLQNQIISATVDNNKLLLDIDNSKMTADDFRMKYEN
ELVIRQSVEADINGLRNLLDDLTLVRSSLESELESLKDELMALKRNHEEEMRQLQSQTGGDVSVEVNAAPGEDLTKTLNDLRNEYEQIIERNRR
EVEQWYEVKIEEVNQQVTSSSQDIQTSSHQLTELRREMQNLEIELQAQLSTKNSLENSLAETESRYGCLLQQIQGQINSVEEELASIRCEMESQ
NQEYKMLLGIKTRLEQEIAQYRALLQEGQQDIVASQGTLQGGGRSSHSYSSASYSQSQSGDVIDRCRQ

>Ostrich_K9L3
MSCGTKSSTSTIKIGSGGSGGGGGGGGGYGGGGGGGSGSSGCRSGGYSSVSSRRYTSSGGSGGFSGRSFGGGGSRSTYGGGFSSGSCGRISRST
YGGRMSGGSFGGGLGCGSMGGGFGSGGGGFGGTGGGYGSSLGGGGFGGGGYSGGGFSGFGGGGFGGGSFGGMGLGEDTGLLSTNEKLTMQNLND
RLASYLDKVRGLEEENAHLERLIREWYQKQGPTGTKDYSQYYRTIEELQNQIVGASVDLNKILLDIDNTRMTVDDFRLKYETEYTLHQSVASDI
NGLRPLLDQLTLARSDLETQFESLKEELIYLKKNHEEEMRGLQTQSGGDVNVEVNATPGINLMEKLNEMRCEYERLIENNRREVESWYETKMEE
VNQQVHSSGQEIQSSNQQISELRREYQSLEIELQSQISMVDSLQSNLEDTERRYNMQLQQIQGMIGPLEEELASIRCEMESQNEEYKMLLGIKT
RLEQEIAQYRALLEEGQQDLTIPGGGLGGGLGGGMGGGRLGGGGYSSGGGRGGGSGGLSGGYGGGGISSGSGMGGGMGGGGGGMGGGSGGGSCG
IIGGGGGGGLGGGSGGGSSSIVGGGGGGGRLSGGVSSSHSYSSSSQSQSCGGGTQGHGRKSFD

>Ostrich_K9L4
MSCGSKQTSKSCLRGSGGGSASSCGGGGGSKYSSVSSRRHTSSRSSGGGRISGSSCGGGSSRSSYGGGMSSGMSSSCYGGGMSSGGYGGRMGCG
SMGGGFGSGGSGLGGGYGGNFGGGGGGFSGGGFSGGSFSSGGFGGGNMGFLSNDEKLTMQNLNNRLASYLDTVRNLEKENIHLEQLIREWYQKQ
GPTGTKDYSHYYGTIEDLQNQLVTAAVETNRMLLDLDNTRMTAEDFRKKYETEYGLRQSVEADINELRPLLDNLTLGRSDLEMQFESLKDELIG
LKKNHEEEMKSLQTQSSGDVNVEVNAAPGEDLLKELNDMRKEYESIIQKNREEVETWYESKMEEVSQQVHSSSQELESSNQQISALRREYQSLE
IELQSELSMVDSLQSNLEDTERRYNMQLQQIQGMIGPLEEELASIRCEMESQNEEYKMLLGIKTRLEQEIAQYRALLEEGQHNITTSQGGAGGG
SAGGGRHGGISCSSGRGSTGGGSSWSSGGRSSGGRSMEENYGRTSTGGGESGGGIRGGASGRISGGGDGSGGGGEGKSCLSPSSSSQSQSGYSC
ESEGKKWNKRLCLQGNKILERGATMEEVSEAIKTMKLGSFRKSEN

>Ostrich_HAS1
MSCNIKETITVSSKGRSSGGSCIIGGGGGARISSYGIGSGRGFSGRSYCGGVNYGGGLSVGSLAGGSYGGGSCYGNGLGFGLGGGVVVGGLGGD
CLLSSCDEKVTMQNLNDRLASYLDKVKCLEKENAELECRIREWYATQGLSCEPRDYSCYYKEIEDLQNQIVCATIDNNKIILDIDNSRMAADDF
RVKYETELALRQSVEADINGLRQVLDQLTLCRSDLEAQLESLREELCCLKKNHEEEMNCLRKQSTGDVSVEVNACPGPDLRQILEDLRCQYETL
IARNRKEVEDWYECKIEEVNREVITSGQEVETCNNQVTELRRQLQALEIDLQAQLSQRNNLESSLAETECQYNTLLGELQNQITCVEQQLAEIR
TEIECQNQEYKTLLDVKCRLEQEIQTYRCLLEGGQQDLIHGGGIGVGTGVGGGVIRTSHTYTTTSSSHCQPQVPPCKTGDIQVTCRRICD

>Ostrich_HAS2
MSCSIKQTTGSLRGRTSGGSCVIGGGGGGGVRISSVSSGRYTTCGIGGSRGFSGRSYCGGVNYGGGLSTGSLVGGNYGGGLGAGVLGGCSGIGF
SGGSVRFGGGIGGGTGIGVGGGVAGGGFSGDGILLSGDEKVTMQNLNDRLASYLDKVRCLEQENADLECRIREWYAKQGPFCEPRDYSCYYKEI
EDLQNQIVCATIDNNKIILNIDNSRMTADDFRVKYETELALRQSVEADINGLRQVLDQLTLCRSDLEAQLESLREELCCLKKNHEEEMNCLRKQ
ATGDVSVEVNACPGPDLRKILEEMRCQYETLIERNRKEVEDWYECKIEEVNREVITSGQEVETCNNQVTELRRQLQALEIDLQAQLSQRDNLEA
SLAETECRYNNHLAELQSQITCVEQQLADLRAEMECQNQEYKILLDVKCRLEQEIHTYRCLLEGGQQDLIQQGGIGQSSGLGGGVARTSGVGGG
SIIRTSHTYTSSSQMPSCAAAEIQVPCRRICD

>Ostrich_HAS3
MQNLNDRLASYLDKVRLLEGENADLERKIREWYTKVGPSCEPQDYSCFHKEIDDLQNQILCAAMEANKILLNTDNNRMTADDFRVKYETECGLR
QNADADICNLRPVVDQLASCKTDLQFQFEALTEEMCCLKTNHEEEMSCLRKQVTGDVSVEVNACPGPDLRKILEDLRCQYETLMERNRKETEQW
YACKVEEVNLEVITSSQEIESSNKQVTELRRQLQALEINVQAQLNMKENLESSLAETECRYNKYLAELQSQISCVEQWLAEIREEMECQNQEYK
TLLDVKCRLEQEIQTYHCLLEGGQQDIVYADYRQTRA

>Ostrich_K14L1
MATTVRQYSSSTSLKGFGGLGGGSSRLSSVHVGGGGYRAPSVSGRTSSYSVSSRTVSGLGSALGGGYGGSYCSSVGGGLGSGFGGGYGGSFGSG
FGGGFGGGFGGVFGGGEGILQAGEKETMQNLNDRLAAYLDKVRALEEANTDLEVKIREWYKKQAPGPDRDYSPYYKTIEELRSKILAATVENAN
IVLQIDNARLAADDFRTKFETEQALRLSVEADINGLRRVLDELTLARADLEMQIENLKEELAYLKKNHEEEMNALRGQVGGEISVEMDAAPGID
LTKILAEMREQYETLAEKNRRDAEQWFFSKTEELNREVAINTEQLQSGKTEITELRRTIQSLEIDLQSQLSMKAALEGTLADTEARYGTQLAQL
QGLITSVEEQLAELRCDMERQNHDYRILLDVKTRLEQEIATYRRLLEGEDAHISSQYSSAMSSHSGRDVMTTSRQVRTIVEEVQDGKVVSSREQ
VALTTR

>Ostrich_K14L2
MATTVRQYSSSTSLKGFGGLGGGSSRLSSVHVGGGGYRAPSVSGRTSSYSVSSRTVSGLGSALGGGYGGSYCSSVGGGLGSGFGGGYGGSFGSG
FGGGFGGGFGGGFGGGEGILQAGEKEKTKNVNDRLAAYLDKVRALEEANTDLEVKIREWYKKQAPGPDRDYSPYYKTIEELRSKVLVATVDNAN
LLLQIDNARLTADDFRTKLETEQALRLSVEADINGLRRVLDELTLARADLEMQIENLKEELTYLKKNHEEEMNALRGQVGGEISVEMDAAPGID
LTKILAEMREQYETLAEKNRRDAEQWFFSKTEELNREVAINTEQLQSGKTEITELRRTIQSLEIDLQSQLSMKAALEGTLADTEARYGTQLAQL
QGLITSVEEQLAELRCDMERQNHDYRILLDVKTRLEQEIATYRRLLEGEDAHMSSHYASQSVREAPVTTRQIRTIVEEVQDGKVISSREQIHQS
PR

>Ostrich_K18
MGGSLLLSGSGMVQNEKETMQDLNDRLATYLDKVRSLEQENHKLEIQIRDFMQKKGPSSRDWSHYWEVIEDLRNKIFDATVDNARTVLQIDNAR
LAADDFRVKYEAELAIRLSVENDIAGLRKVIDDTNMARLHLEGEIESLKEELIFMKKNHEDEVKSLQAQISDSALTVEVDAPKSQDLGKIMAEI

RAQYDALAQKNLEDLEKQWGQQITESTIEITQSSKDVDTARSTVVDLRRTVQTLEIDLESLRNQKAGLEANLLEVENRYAMQMEQLNGLILRVE
AELAQVRGELQRQAEEYQALLNIKDKLEAEIATYRQLLEGGEEFSLRDALEKETTSTVQTSTQKIVDGKVVSETKEVKVRTY

>Zebra_finch_K12
MALSVRTSGGPRQFSSRSGFGGGSLRISSSSGGGGFGGSGLGFGGGSGGGFGAASLLGSSSGFGGGFGGGLGGSYGSGLGSGFGGGLGSGFGSS
SGAAFGSGFGGGLGTGFGGGFSPAGAGDGGILSGSKKETMQNLNDRLAAYLDKVRSLEDANTELERKIREWYEKNGPGTGTPGSGNDYSKFYPL
IEDLRNKIINATIDNARIILQVDNARLAADDFRVKYENEVALRQSVEADINGLRRVLDELTMTRADLEMQIESLNEELAYLKKNHEEELQGIQS
SELGQVSVEMDAAPGTDLTKLLNDMRGQYEVIADQNRKEAEAWFNEKSGELKREISTHTEQLQSGKSEITDLKRTLQSLEIELQSQLAMKKSLE
DTLAETEGGYCAQLSQIQMQIGNLESQLFQVRADMERQNAEYQQLLDIKTRLEMEIETYRRLLDGEFVSAGQAATFESSSLTGSKSQTQSLDSS
QDPTKTRKIKTIVEEVVDGKVVASHVKEVEEKI

>Zebra_finch_K20
MAFSTNSTQWSTSSRVQPSAPSVSSVTSRKMSIQKIQAPSVYGGAGGYGTRISASTSPGQGLRGSLQLSITGSDVLLAGNEKSTMQNLNDRLAA
YLERVRSLEKSNSLIERQIREWYEKNTTSIGQDYSSYFKTIDELRNKISAAQMENSRLVLQIDNAKLAADDFRLKFENEHLLRQSVESDITGLL
RVRDDLTLTQADLESQIENITEELVFLRKNHEEDRDRLRREASSSVNVAVDAAPGIDLAAVMENMRKQYEEMAEKNRQEAKEHFEKQTAELNQE
VALNIEQLEVQRKEITERRQVFQNLELELQSLLTMKQTLEGTLAETEARYSYQLDQIQGAISSLEAQLRQLRADMEAQNSEYSILLDVKTRLEM
EIATYRRLLEGEESGHLEEISLAVDAEKESSKFKKIKTIVEEVVDGKVVSSEVREFEEKM

>Zebra_finch_K23
MQAHKCFRIDQEEARFVMTQSCCCSHSSKGNSHRKRHKAAQGRRRGCSVLGSQTWGSIHTGLSMSTSQGPFQLFSGSLRGSSGCGLAQQGTSYR
ASSADGGASSTHPFSSARPFWLAAGGAPWGGFGEHYGESIFLGGNEKQTMQNLNERLAAYLDKVRALEAANAQLESCILEWHRTKSHGKRHDFN
QYEQNVTDMQRQIEDSKITNASILLQIDNANMATEDFRLKYEAEKCRRQGVQLDVENLRKELDGMTIITTDLEMEIEGLREEHILRRKDHEEDM
EANRSSQDFKVNVKVNAAPPADLGKILAEIREDYEAIIEKNRQSLDNWYKEQSAAMSLAATPNPEQIQRNENEIKELRRTLQSLDIELQAQISK
KHMLEDTLADTRNYYAAALQNMQQIISRCEEELSQVRHDMKQQNNQYKVLLGIKTRLEKEISTYRLLLEGNADGTARKSETKEHSGLSNRKLRA
IVHDSVNGEVVSSTINELPPQA

>Zebra_finch_K15
MATSFMSSSSTYGGGFGGAGGSSSRLSSVRVGGTCRAPSIHGGSGGRGVSISSARYVSSSGGGCGFGGGYGGGFGGGAVCGFGGGSGFGGGSGF
GGGFDFSGGFVAGDGLLSGNEKITMQNLNDRLASYLNKVRALEEANSELEVKIRDWYQKQAPTSPARDYSNYYKIMEDLRDKILAATIDNSRVI
LEIDNARLAADDFRLKYENELFLRQSVESDINGLRRVLDELTLSRTDLELQIETLKEELAYLKKNHEEEMKEYSKQLSGTVNVEMDAAPGIDLT
KVLAEMREQYEALAEKNRKDAEAWFFTQTDELNREVATHTQQIQTGKSEITELRRTLQSLEIELQSQLSMKAGLEANLRDTEGRYCAQLAQIQV
LITSVEEQLAELRCDMERQNQEYKMLMDIKSRLEQEIATYRQLLEGQDSQLSGLNPKDAFGSSGRVRSGADDDVRSGSTRDRRNQ

>Zebra_finch_K19
MASYSFRQTSSSMAGGPGASSLRFGGSFRAPSIHGGSGGRGVSVSSARFVSSGLGSGLGGGYGGGSFSSSFSYGGGLGGDGLLSGNEKATMQSL
NDRLASYLEKVRALEEANSDLETKIRNWYQKQGPSPARDYSPYFKTIEDLRDKILDATIDNARIVLQIDNARLAADDFKTKFETEQGLRMSVEA
DINGLRRVLDELTLSRADLELQIEGLKEELAYLKKNHEEELSTLRGQVAGQVSVELDSAPGIDLSKILADMRDQYEHMAEKNRKDAEAWFHSKT
EELNREVAVNTEQLQSSKSEVTDLRRTLQGLEIELQSQLSMKAALEGTLADTEARYGAQLAQIQGLVGSIEAQLAELRADMERQNTEYKILMDI
KTRLEQEIATYRQLLEGQESQMFGSLSGTADKRDKH

>Zebra_finch_K9L1
MSCSIKRTSSTSYRSGGGGGGACGGGSSSMSCRRYASSVIGGGSYGGAACGGYGGGLSMGSLSMGSLGGGFGGGSAGICGPGGDLLLSGNEKVT
MQNLNDRLASYLDKVRRLEEENAQLEHHIREWYRKQAPSVSKDYSSYYQTIEQLQNQIISATVDNNRMILDIDNSKMTADDFRMKYENELGIRQ
TVEADINGLRNILDGLTTTRSALECELESLRDELIALKRNHEEEMRQLQSQTGGDVSVEVNAAPGEDLTKILNEMRDEYEQIIEKNRREVEQWY
EVKIQEVNQQVTSSSQDIQTSSHQLTELRREMQNLEIELQAQLSTKSSLENSLAETESRYGRMLQQIQAQINCIEEELASIRCEMEGQSQEYKM
LLGIKMRLEQEIQQYRALLQEGQQDLGSCQGGLQGGGMTSHSSSSYCHGQSSDKTGQSRVC

>Zebra_finch_K9L2
MSCSIKRTSSTSYRSGGGGGGACGGGSSSMSCRRYASSVIGGGSYGGAACGGYGGGLSMGSLSMGSLGGGFGGGSAGICGPGGDLLLSGNEKVT
MQNLNDRLASYLDKVRRLEEENAQLEHHIREWYRKQAPSASKDYSSYYQTIEQLQNQIISATVDNNKMLLDIDNSKMTAEDFRMKYENELVIRQ
TVEADINGLRNLMDDLTLVRSSLESELESLKDELIALKRNHEEEMRQLQSQTGGDVSVEVNAAPGEDLTKILNEMRDEYEQIIEKNRREVEQWY
EVKIQEVNQQVTSSSQDIQTSSHQLTELRREMQNLEIELQAQLSTKTSLENSLAETESRYSCLLQQIQAQINCIEEELASIRCEMEGQSQEYKM
LLGIKTRLEQEIQQYRALLQEGQQDLVSSQGAFQGGGKSSQSYSASYSHSQCGDMSGQSRVC

>Zebra_finch_K9L3
MSCGTKSSTSTIRISSGGGGGGGGGGRCSGGGGGGGRSGGYSSMSSRRYTSSGGSGGFSGRSFGGGGSRSSYGGGFSSGSCGRISRSSYGGRMS
GGSYGGGMSCGSMGGGLGSGGGGFGGMGGGYGGGGYSGGGFSGFGGSGGFGGGGSYGGGSFGGMGFGDDSGLLSTNEKLTMQNLNDRLASYMDK
VRRLEEENSQLEQLIREWYKSQAPSQSKDYSQYYRTIEELQNQIVGANVDLNRMLLDMDNTRMTVDDFRLKYETEYTLHQSVASDINGLRPLLD
QLTLSRSDLETQFESLKEELIFLKKNHEEEMRGLQGQSGGDVNVEVNATPGINLMEKLNEMRCEYERLIENNRKEVESWYETKMEEVNQQVHTS
GQEIQSSNQQISELRREYQSLEIELQSQISMIDSLQSNLDDTERRYNMQLQQIQAMIGPLEEELASIRCEMESQNEEYKMLLGIKTRLEQEIQQ
YRALLEEGQQDLVIPAGGMGGGMGGGMGGGMGGGRIGSGGYSGGGRGGGGGMSGGYGGGGSGMGGGMGGGSCGGGMGGGSGGGGMGGGSGGMGG
GSGGMGGGSGGGCIGGGGGGRTSGGVSSSHSYSSSSQSQSCRGGSESQGYGRKSFD

>Zebra_finch_K9L4
MSCGSKQPLKSCLRGSSGGGGSSYSSASSRTVTSRTSGGGRFSGSSCGGGSSRSSCAGGSAYGRRSSYGGGMSSSSWGGRLGGACHGGGMGHSS
MGTGFRSGSGFGGGYGAGFGGGAVGFSGGSFGSGGFHGGNVGILSYDEKLTMQSLNDRLASYMETVRNLEKENAQLEQLIREWYQKQGPIGPKD
YSHYYGQIEELQKQIMAAAVETHKVLLDLDNTKMTAEDFRIKYEMESGLRQNVEGDLNSLRPLLDNLTLTKSDLEMQFESLKEEILDLKKNHEE
EMKLLQSQSNGDVNVQINTAPGEDLLKKLNEMRQEYEAIIQKNRAEVEKWYESKMEEVSQQVHSSSQEIESSNQQISVLRREFQSLEIELQSQI
SLIDSLQSNLEDTERRYNMQLQQIQAMIGPLEEELASIRCEMESQNEEYKMLLGIKTRLEQEIQQYRALLQEGQHGISTSQGGAGGGSSGGGGH
GGSSGGGSCWSSGGRSSGGKTGGSYGRSSSSESGGGGSGGRTGGGTCGKTSGGGGGSSGGGGGKSCLSRSSSSQSQPGDSCESPGSLRKVEE

>Zebra_finch_HAS1
MSCNIKETITVSSKGRSSGGSCIIGGGGGARISSFGIGSGRGFSGRSYCGGVNYGGGLSVGSLAGGSYGGGNCYGNGLGFGLGGGVVVGGLGGD
CLLSSCDEKVTMQNLNDRLASYLDKVKCLEKENAELECRIREWYATQGLSCEPRDYSCYYKEIEDLQNQIVCATIDNNKIILDIDNSRMAADDF
RVKYETELALRQSVEADINGLRQVLDQLTLCRSDLEAQLESLREELCCLKKNHEEEMSCLRKQSTGDVSVEVNACPGPDLRQILEDLRCQYETL
IARNRKEVEDWYECKIEEVNREVITSGQEVETCNNQVTELRRQLQTLEIDLQAQLSQRNNLESSLAETECQYNTLLSELQSQITCVEQQLAEIR
AEIECQNQEYKTLLDVKCRLEQEIQTYRCLLEGGQQDLIHGGGIGTGGGVIRTSHTYTTTSAAHCQAQVPPCKTGDIQVTCRRICD

>Zebra_finch_HAS2
MSCSIKQTTGSLRGRTSGGSCVIGGGGGGARISSVSSGRYTSCGIGGSRGFSGRSYCGGVNYGGGLSTGSLVGGNYGGGLGAAVLGGCPGMGFS
GGSARFGGGMGGGMGVGLGGGGFAGDGILLSGDEKVTMQNLNDRLASYLDKVRCLEQENADLECRIREWYAKQGPFCEPRDYSCYYKEIEDLQN
QIVCATIDNNKIILNIDNSRMTADDFRVKYETELALRQSVEADINGLRQVLDQLTLCRSDLEAQLESLREELCCLKKNHEEEMCCLRKQSTGDV
SVEVNACPGPDLRKILEEMRCQYETLIERNRKEVEDWYECKIEEVNREVITSGQEVETCNNQVTELRRQLQALEIDLQAQLSQRDNLESSLAET
ECRYNNHLAELQSQITCVEQQLADLRAEMECQNQEYKILLDVKCRLEQEIHTYRCLLEGGQQDLIQQGGIGQSSGLGGGVARSSGIGGGGIIRT
SHTYTSSAQIPSCAAAEIQVPCRRICD

>Zebra_finch_HAS3
MSCTVRQVVTTCTQGRASTGSNAAGTGRRVSSASSGRHAAYDMGAVVGSFSGGSVSEGLLGKQLSAGSAAGGSFGATQRACSALGFSGGVCTRG
GFPRAGAGCGDGILFANNEKATMQNLNDRLASYLDKVRLLEGDNADLECKIREWYAKVGPICEPRDYSCYHKEIEDLQNQILCAAMETNKILLN
IDNNRMTADDFRVKYETECGLRQNVDADINNLRPVLDQLASCKTDLQLQCEALTEEMCCLKTNHEEEMSCLRKQATGDVSVEVNACPGPDLRKI
LEDLRCQYETLMERNRKETEQWYACKVEEVNLEVVTSSQEIESSNKQVTELRRQLQALEINVQAQLTMKENLESSLAETECRYNKYLAELQNQI
SCVEQRLAEIRAEMECQNQEYKTLLDVKCRLEQEIQTYHCLLEGGQHDIIGSAGRGVGAAPAGRSTGLKGSLCQPCLP

>Zebra_finch_K14L1
MSTTVRQYSSSTSLKGFGSLGGGSSRVSSVRVGPGGYRAPSVHGGSGSYSVSSRVVSGLGGAFGGSYCSSAGGALGAGFGGSFGAGFGAGFGAG
FGAGFGAGFGGGDGILPAGEKETMQNLNDRLAAYLDKVRALEEANTDLEVKIREWYKKQGPGPDRDYSPYYRTIEELRNKILSATVENANIVLQ
IDNARLAADDFRTKFETEQALRMSVEADINGLRRVLDELTLSRADLEMQIENLKEELAYLKKNHEEEMTALRGQVGGEISVEMDAAPGIDLTKI
LADMREQYETLAEKNRRDAEQWFFTKTEELNREVAINTEQLQSGKTEITELRRTIQSLEIDLQSQLSTKAALEGTLADTEARYGTQLAQLQMLI
TGVEEQLAELRCDMERQNHEYRVLLDVKCRLEQEIATYRRLLEGEDAHISSQYSSAMSSHSGRDVMTSSRQVRTIVEEVQDGKVVSSREQVALT
TR

>Zebra_finch_K14L2
MSTTVRQYSSSTSLKGFGSLGGGSSRVSSVRAGPGGYRAPSVHGGSGSYSVSSRVVSGLGGAFGGSYCSSAGGALGAGFGGSYGAGFGAGFGAG
FGAGFGGGDGILPAGEKETMQNLNDRLAAYLDKVRALEEANTDLEVKIREWYKKQGPGPDRDYSPYYRTIEELRNKILVTTVENSNLLLQIDNA
RLTADDFRTKFETEQALRVNVEADINGLRRVLDELTLSRADLEMQIENLKEELAYLKKNHEEEMTALRGQVGGEISVEMDAAPGIDLTKILADM
REQYETLAEKNRRDAEQWFFTKTEELNREVAINTEQLQSGKTEITELRRTIQSLEIDLQSQLSTKAALEGTLADTEARYGTQLAQLQMLITGVE
EQLAELRCDMERQNHEYRVLLDVKCRLEQEIATYRRLLEGEDAHMSHYASQPVKEGPVTTRQIRTIFEEVQDGKVISSREQITQAAH

>Zebra_finch_K18
MSFSRRTVYSSSARSGGIGTLGTLGTLGTLGTGPGRRSAPLGSAASVYAGAGGAGSRISVTRTLSSSAYGAAYGAGYGAGYGAGYGAGLGGSIL
LGGSGAVANEKEAMQDLNDRLATYLEQVRRLEQENRRLETQIREFMARKGPSAHDWSPQWELIEELRDKILESMVENARTVLQIDNARLAADDF
RVKFEAELAIRLSVDSDIAGLRKVLDDTNMTRLQLEGDIEALREELILLRKDHEQEVQDLRAQVSQSALTVEVDAPRSQDLGKVLGELRAQYDT
LAQKNLEDLEKQWGQQITETTLEVTQSTKDLDVARGTVGALRRSLQTLEIDLEALRNQNAGLEAALAEAEARAGAHLAQVQLQVSAAEAELRDV
RAQLQRQNEQHRELLGLKDRLEAEIATYRQLLEGSDGFSLRDALDKETTATTAGMGTTQHLVTETREVKVRTY

>Nanorana1_02
MSCHWKSGSVRSVSSVSSHRISSLVGDLAIASDHFAVGNHGANDGLLHGGEKETMQNLNIRLASYLDKVKALEDANADLETKIKEWYIKHQPKV
EDYSPYFRIIEELKCKILSESNENARIVLQIDNARLAAEDFKIKYENELCLYQSVESDICGLRKVLDELTFAKSALVPQFESLQEELECMKKNH
EAELHAFKGERADVNVEMNAVPGVDLAKLLNDMRAQYEQIAEQNRQKAADWFNEKSAEINQEISHSSEMVECHKTQLTELRRTLQELEIELQAQ
LAMKKSLECSLAETERCYSTQLAQLQVVVGNVEEQLSQIRNDLERQNAEYKLLLDIKTRLEKEIETYRRLLDGEPWHCKEVHIPVKEASKTRKV
TTIVEEVVNGRIVSQKVNATEEKLKN

>Nanorana1_03
MQNLNIRLASYLDKVKALEDANADLEAKIKEWYLKHQAKTVPGDYSKYFSIAEDLKNRILVETTENARIVLQIDNAKLAADDFRIKYENEVLLH
QNVEADICGLRRVLDDLTFTKSNLEPQLESLKEEIECLRKNHEEEMNALRGQKGDVNVEMDAAPGIDLTKLLSDMRSQYEEIAEQNRQKAEQWF
REKSAELNHEISHSSEMVESHKSQLTDLRRTVQELEIELQAQLAMKSSLEGSLAETEHCYCAQLSQIQILIGNVEEQLSQVRSDLERQNAEYQL
LMDIKTRLEMEIETYRRLIDGEPLGTGSDTTSEQNKTRKVTTIVEEVVDGKIVSQKVNATEEKV

>Nanorana1_04
MQNLNDRLASYLNNVQDLEDANADLESKIKAWYEQHCNDNKQVKDYSKYFKIIEDLKKQIQAATLDNARIVLQVDNARLAADDFKMKFENELSL
RLNVEADINGLRKVLNELSLAKCDLEAQIQSLTEEIGCLKKNHEEEMKSFLGVTGQLSVEMNAAPGNDLTKILNNMRADYESLAEKNRREAEAQ
FIEMSRVLKQEISSGTELIQSTRNEIIDLKRSVQSLEIELQAARAMKTTLESSLAETEGSYCLQLGHIQAKISNLEEMLCQVRTEMESQGIEYD
HLLDIKTRLEREIETYRCLLDGQSIPYKNKPQKFRKVRTIIEEVVDGRTVSQQVREREEKV

>Nanorana1_05
MSCQKPCAPSRLSVGGGNYGTSVCSFPGRAGATCGAQSFGAHQSGGAFGDFGVGSLSGGAVGNFSAGYFGGDGLLCGSEKETMQNLNDRLASYL
NKVQDLEDANADLESKIKAWYEQHCNDNKQVKDYSKYFKIIEDLKKQIQAATLDNARIVLQVDNARLAADDFKMKFETELSLRLNVEADINGLR
KVLNELNLIICDLEAQIQSLTEEIVCLKKNHEEEMKSFLGVTGQLSVEMNAAPGNDLTKILNNMRADYESLAEKNRREAEAQFNEMSRVLKQEI
SSGTELIQSTRNEIIDLKRTVQSLEIELQAACAMKTTLESSLAETEGSYCLQLGHIQAKISNLEEMLCQVRTEMESQGIEYDHLLDIKTRLEKE
IEIYRCLLDGQAMXEEFYXDIGTNTVKKNEFILNSNKPQKYRKVRTIIEEVVDGKTVSQQVQEREEKV

>Nanorana1_06
MQNLNDRLASYLNKVQDLEDANADLESKIKAWYEQHCNDNKQVKDYSKYFQIIEDLKKQIQAATLDNARIVLQVDNARLAADDFKMKFENELSL
RLNVEADINGLRKVLDDLSLAKCDLEAQIESLTEEIVCLKKNHEEEMKSFLGVTGQLSVEMNAAPGNDLTKILNNMRADYESLAEKNRREAEAQ
FIEMSRVLKQEISSGAELIQSTRNEIIDLKRSVQSLEIELQAARAMKTTLESSLAETEGSYCLQLGHIQAKISNLEEMLCQVRTEMESQGIEYD
HLLDIKTRLEKEIEIYRCLLDGQAYGTLYTXMLLLTDTSSFSSEPQKFRKVRTIIEEVVDGKTVSQQVKEREEKV

>Nanorana1_07
MPGGISVGGQRGGFSNGSLGAFSYGGGDGILQAGEKETMQNLNSRLATYMDTVHALEDSNAELEIKITNWYDTHKLPNVDYTKYYSIIEALKDK
IFSATLANNHLTVQSDNARLAADDFRVKFDSERCLYQGVESDANGLRKVLDDLTLDKSSIESQIESLREELAYCKKKHVLEMKDLQGVSGDVTV
QMDAAPGVNILKILNDTRAQYEHLAEENRRKAEEEYSQKVSELNSEISHSSEQIESVKSDVMDLRRTMQNMEIELQTQLAMKNSLENSLEETED
RYCDQLAQIQDKITGLEDQLGHLRNDLETQSREYKLLLDIRNKLENEIDMYRTLLEEEG

>Nanorana1_08
MQNLNGRLGSYLDKVRQLEAANAEYEQKIKEFLEKQRSGGTKGEAGKDYTKYFTIIADLQSKIIAATNDNANLVLQSDNARLAADDFKMKYENE
FALRRSVEADINGLRKILDELKFRISELQLEIESLTEEIVLLRNNHQEEIKGSQGGGVGDVNVELNAAPGNDLLKEINNLREQYEAMAEKNRKD

AEEQFKKASEGLKKEISTGVEQISTSKSEISDLKKTLQALEIELQSQWSMKKSLEENLSQTEGQYCMKLSQIQAQIASIEQQLAQLRADMECQT
AEYEELLDIKTKLENEIEIYRRLLEGER

>Nanorana1_09
MSYAQMSSTRSVKVGSVSGGYSGGGGSHGQCGFGGDFSQAGGNYGQVASGFSQAAGGFGQAGGFGYGGGEFCGQGAGGGYGGAGGAGGAGGASF
GGGGGGFGDAGFGGGDSFLSGNEKQTMQNLNGRLGSYLDKVRQLEAANAEYEQKIKEFLEKQRSGGTKGEAGKDYTKYFTIIADLQSKIIAATN
DNANLVLQSDNARLAADDFKMKYENEFALRRSVEADINGLRKVLDDLTMKRSDLELEFEGLTEEIAVLRKNHQEEIKGSQGGGVGDVNVELNAA
PGNDLLKEINNLREQYEAMAEKNRKDAEEQFKKASEGLKKEISTGVEQISTSKSEISDLKKTLQALEIELQSQWSMKKSLEENLSQTEGQYCMK
LSQIQAQIASIEQQLAQLRADMECQTAEYEELLDIKTKLENEIEIYRRLLEGEDAHLRSSASLRLS

>Nanorana1_10
MAFYKAESMQFSGGSGQCGVGGMQGGYGQVGGGYGQAGYGQGCGQGFEQKVGFAYDAAQCQGFGGGAVGGGFGGGAAGGGYGGGAAGGSYGGGA
AGGGYGGGAAGGGYGGADAGYGGGEMFFSGNEKQTMQNLNDRFATYLDRVKQLEAANVDLEKKIKEFLDKQRAGSTTGEGKDYSPYFKTIEDLK
AKIIVAFNNNNTIVLQIDNARLAADDFKLKYENELTLRRAVEADIVGLRKILDDLTLSKSDMESQVESLVQEIALTKKNHDEEVKGGQQTTVGD
VSVEMNAAPGSDLLKKMNDMREQYEALAEKNRKEAEEQFKKASEGLQKQIQVVVTEEKTTKSEVSELKKTLQALEIELQSQLAMKKSLEETLAQ
TEGQYCAKIAQIQMRITAVEEQLTQIRSEMECQTAEYEDLLDIKTKLEAEIATYQKLMDGR

>Nanorana1_11
MAFYTSQSSSGGAYGGGGLVMNGEGFGECFDGGFGGGESSTGFGGGQQVRFSSGGFRAGAGGGAGGGGGGGFGGGYGGSAGGAYGGSAGGGYGG
SAGGGYGGSAGGGYGGGYGGAGGGGFGGGGFGAGSGLLSTNEKQTMQNLNDRLATYLDKVKTLEEDNAEYERKIREWYEKQRPGSSTGAGATDY
SKYFDIIDDLRSKILAATIENSKLILQIDNARLAADDFRLKYENELSMRQSVEADINGLRRVLDELTLSRSDLELQIEALAEELSYLKKNHQEE
MGTLAGSQSGQLSVEMNAAPGIDLTKILNDMREQYEALAEKNRKEAEIQFLQQSSELKKEITAGLAETQTKSTEMTELKRTLQSLEIELQSQLA
MKRGLEQTLAETEGRYCSQIAKLQDIIGGVEDQLSQCRFDTERQSEEYRQLLDIKSRLEKEIEQYRVLLDGEGGSFGISSSSSTTQKTSGSVGS
SKDSSKTRKVMTIYEDIVDGKVVSSTKTEHLQKM

>Nanorana1_12
MQNLNDRLASYLEKVRALEAANVELETKISQWYEKQVGIGAGNAANDYSKYFDTINDLRSKILAATVDNSTILLQIDNTKLASDDFRQKWEYEQ
SLRQNVEADINALRKVLDELTLSRGDLELQIESLSEELAYLKKNHEEEMQVSRSSAAGQVEVQMDATPGVDLTSILDDMRANYEALAQKNRKDV
EAQFQKKSGELKKELSANVEEVQSSKTEVSDLRRTLQGLEIELQSQLAMKKSLEDTLADIDGRYGAQLQQLQQVIGGLEAQLQQIRSDMERQST
EYRELLDIKTRLEMEIETYRRLLEGEL

>Nanorana1_13
MQNLNDRLASYLEKVRALEEANTELEIKIRDWYEKQVGSGVGVGGKDYSKYFEVIEDLRSKILSATIQNSRIVLEIDNARLAADDFRLKYENEV
ALRQSVESDINGLRKVLDELLMSRGDFEIQIESLLEELAYLRQNHEEESQVAKGSAAGQVSVEMDAPPGIDLTKILNDMRGDYEALADKNRKDA
EAWFAQKSGELKKEISVGVEQVQSTKGEISDLRRSLQGLELELQSLLAMKKSLEDTLADIDGRFGAQLQQLQQVISGLEEQFMQIRSDMERQST
EYRELLDIKTRLEMEIETYRRLLEGEIDQFSSQSSSIVTESRSSSSTATKTRKVKTIIEEVVDGKVVSSTVQEIEENMN

>Nanorana1_14
MASRFSSSSAYQVSSSGGGYGGGYGGGSSSYGGGGGGGSYGGGSGGGSYGGGFASGGGSSYGGGFASGGGSYGGGFGGGYGGGSSGGFSFGGGG
AGSGGLGSFGGGEKQTMQNLNDRLASYLDKVRALEAANTELETKIREWYEKQVSGGVGSGAKDYSKYYDIINDLRAKILAATIDNSRIVLQIDN
ARLAADDFRLKFENELALRQSVEADINGLRKVLDELTLSRGDLELQIESLSEELAYLKKNHEEETQIARGSAAGQVSVEMDAVPGIDLTKILND
MREDYEAMADKNRRDAEAWYASKSGELKKEISVGVEQVQSTKSEISDLRRTLQGLEIELQSQLAMKKSLEDTLADTEGRYGGQLQQLQQIISGT
EDQLIQVRSDIERQSMEYRELLDIKTRLEQEIETYRRLLDGELGXVQKISLTHFCILDSKKDPTKTRKVKTIVEEVVDGKVVSSRVEEVEEKMN

>Nanorana1_15
MSNRFSSASSYQTSSSGGGFGGGFGGSSYGAGSYGGGYGGGSSGGGYGGGSSGGGFSFSAGGSANFAGNDKQTMQNLNDRLASYLEKVRDLEAS
NSELEIKIRDWYDKQVGAGAGAGGKDYSKYFDTIDDLRGKIFDATLLNSKLVLQIDNARLAADDFRMKYENELALRQSVESDISGLRRALDDLT
MSRGDLEIQFESLAEELAYLKKNHEEEMQIARSSATGQVNVEMDAAPGIDLTKLLNDMRADYEALADKNRREAEALFAAKSKELHQEISAGVQQ
VETTKSELTDLRRSLQGLEIELQSQLAMKKSLEDTLAETDGRYGGQLQQLQQVIGGLEEQLQQIRSDMERQSMEYRELLDIKTRLEMEIETYRR
LLEGELDVTSTENRPQYLYXXHFLIFISDPTKTRKVKTIVEEVVDGKVVSSSVQEVEEKMN

>Nanorana1_16
MSTSSYQVRSSSHKMASNSRGSGYGGYSSASLSGAGFGGGLMSQSTSANYASSGFGGSDGLLLGGEKHTMQNLNDRLANYLDKVRDLEVANAEL
EKKIREWYEQHSATNNSQSRDYSKYFNIIEDLKNRIIIATTENARVVLQIDNARLATDDFRMKYENELCLRQSVESDINGLRRVLDELTLGRSD
LELQIESLTEELAFLKKNHQEEMSAYKGSTGQISVEMDAAPGVDLTKILNDMRADYELLADKNRKEAEASFLKKSNELKKEISVGVQEVQSTRT
EISDLRRTLQSLEIELQAQISMKNSLEGTLEETEGRYCAQLNQLQNQISSIEEQFQQVRSDMERQKAEYVRLLDIKTRLEMEIETYRRLLEGEL
GSFAIQQTTSQASSVDSKKDPTKVRKVKTIVEEVVDGKVVSSQVKEVEEKMN

>Nanorana1_17
MYSQVAFKKVNSVKTGSVHGTSIGGINYRKVTSGGYAQSVYAGAGGLGTRVSGVLQHTVHSGSPLQHGKRETRIFGNEKETMNNLNDRLASYID
KVHSLEESNVHLEAQIRDWYSKNTGNTERNDDHNFQVIKDLRNQIISATVENATITLKIDNTRLAADDFRIKFENEQALRLLTEKDTIELRKAI
DQLTLARAELEMQIEGLNEELVYLKKNHTEEVNEIRKQSSGSVDVEVDVAPTVNLGKAMEDMREQYAKMIEKQRLEAKFWFDKKLEDWNEEVHV
NTTELEKYRKDLTEVKQTVQNLEIHSQAEFSKKNAAEATLGNVKDQYAMQLTDLQENITHVESMLQQTRRDVNHQISEFTLLLSLKTRLEAEIA
TYRSLLDGGTRECDDLEWLRCMDQMSVDCRAETKCRNKKGRDDTWTSWNQIRQQVRGQWSTVWMKWFRVLVERCTKWQARSKVRFKPRLYTRSR
TLKCRITYQEQSHQS

>Nanorana1_18
MAFTQVSIKRLGSIDRSPNLPGLGYSNVATTRYAGSVYGGAGGYGTKISTGSGFGSGGYSNLQISTGSDVLLAGNEKETMQNLNDRLATYLDKV
RSLEKANSDIEIQIKEWYIKNSGVVERDYSSYFKAIEGLKNQILNATTDNARILLQIDNAKLAADDFRLKYETEQVLRFGVEKDIAGLRRVIDD
LSLTRGDLELQIENLQEELAYLKKNHEEEVTALRQQVGGTVNVEMDAAPSVDLAKVLSDMRLECETVSEKIREDARLQFETRIEAVHREITVST
GELEQFKTTITEMHRNIQGLEIELQAEQSKRDGLAATLDNVKAQFASRLAEIQRAISNREAQLMQIRTDMGRQAHEYEILLNIKIRLEMEIATY
RRLLEGEETR

>Nanorana1_19
MSLWRVDDLVRTKRQDNGHRLIHGNRKASADFCCPSQKDVLIKLNDRLAAYLERVRTLEESSQQLEEKIKKVSEKRATGHDHSSYLKTIDDLES
QIRSAKQANAGLWLNIENTKTAADCFKEKYVAELTLQDTIKDDVKKLKAASTGLQMETRCLEVEEQILTGELKNLMQDHKEEREHLLQEKSKRK
VNVEVDSVLPTELTGALEKMREQYKAIANHHQQHYESLLVEKVWRTMLHLKVL

>Nanorana1_20
MSLYSSKFLSSCTSINGGIGEPHGSSSYSMGTGNSLSRSRSLSSSLGRIDFTKDYLNDSLLSGNEKHTMQNLNDRLASYLEKVRSLEEANAELE
KKIIDWQKNKSQNKKKDYSSYEKAIADLQTQLLNGHVNGAKLTLQMENAKLALDDFKRRYDTEKAFRAVLETDVEGLRRVMDNLTIVRTDLEMD
VEGMRKQLIYMRKSHEEDMRLAQAQKQGSTVNVEVDAPKGADLAKCIAEVRKDYEAIIANNRKEAEEWYTHQSTTVQKEVNTHTEALQSSKNQT
RELKRTLQNLEIEMQAELSKKQGLENTLDETQCHAGAQLQKIQELICQMENELSKVRNDLERQSNEYKILLDIKSRLENEIGTYRRLIDGNNSR
YNYVYTNRKVKTIVQDMVNGRVVNSRISEIPQKI

>Nanorana1_21
MSVKLSQRSSSGSLRGLGSGSCGVGRISNVRTGGSYRASSVHGGHGSRVMGSSYGVGGLGCASGGSISLGLGGAGGYGSGFGYGSGSAGGLGSG
FGFGGGSFASGGESIININEKETMQVLNDRLASYLHKVRSLEDENSQLEKKIREWYEKQVPYTSPDFNPYFRTIEELQHKILQANATNASILLQ
IDNARLAADDFRTKFENEAALRMGVEADISGLRRVHDELQLSKRDLEMQHQNLNDELNFLKKNHEEEVLSLRSQLGAKINVEVDAAPAVDLNRV
LTDLRSQYENMMEQNQKEAEKLFHDKSDELNQQMTSSSQQIQTFNTEVLQLQHTFQNLEIDLQTQNSMKTALENTLADTEARYCAQLSQLQEMI
NDVEVKLAELRCDLERQNFEYKTLMDVKTHLEREIATYRQLMDGEDIQRETIRLRRSLQQTILSVLQIPLLPPTV

>Nanorana1_22
MQHLNDRLASYLDKVRSLEQENAQLERNIQEWYERNQPSALPNFSSFFRIIQELQGQISSTSLDNARITLQIDNARLAADDFRNKYEMERQLRC
SVEADVNGLRILLEELNRDICNLQAQVGKLQEELQQMRRNHKEEVNALQAQLGARVSVEMNAAPSADLNRTLSEIREQYENLMEQNLREVETIF
QQRTEELNRDVASGSEQLQSVQTEVIDLNRTVQTLEIELQSQLSMTSALENTLAETEATFGAKLAQLQCLINSVESQLAQIRSDLERQNQEYRI
LMDQKTHLEMEIATYKRLLDGHGSHHSMKAHHGTSHSNHHQSVC

>Nanorana1_23
MEKVPGINQLDKVKFRPQAFSVVTPLLDPLMETVILVAICLKSQVTMVLVMEDLQITIMEDPTLLANMDYHMVYLMVYLMLLIMEDHIFPSMEV
PILLIMEAPTDFIAEVLIVLIILPMPPSMEVPIKSIMATTIEPPVYMEGQEETMQHLNDRLASYLDKVHSLEQENAQLERNIQEWYERNQPSAL
PNFSSFFRIIQELQGQISSTSLDNASITLQIDNARLAADDFRNKYVMERQLRCSVEADVNGLRILLEELNRDICNLQAQVGNLQEELQQMRRIH
EEEVNALEAQLGARVSVEMNAAPSADMNRALSEIREQYENLMERNMREVETIFRQRTEELNRDVASGSEQLQSVQTEVIDLNRTVQTLEIELQS
QLSMTSALENTLAETEATFGAQLAQLQCLINSVESQLAQVRSDLERQNQEYSILMDQKTHLEMEIATYKRLLDGQQAFAGDVSLILSKSTCDVA
DRLHLRWLYNMETLMEAAHILYREDREWNEIASSQGEYSELPGRRVEQSAAVVKMMRSLSTPAQCQHIAGPLSN

>Nanorana1_24
MQHLNDRLASYLDKVRSLEQENAQLERNIQEWYERNQPSALPNFSSFFRIIQELQGQISSTSLNNASITLQIDNARLAADDFRNKYEMERQLRC
SVEADVNGLRILLEELNRDICNLQAQVGNLQEELQQMRRNHEEEVNALQAQLGARVSVEMNAAPSADLNRTLSEIREQYENLMERNLREVETIF
RQRTEELNRDVASGSEQLQSVQTEVIDLNRTVQALEIELQSQQSFRSALENTLAETEATFGDQLAQLQCLINNVESQLAQIRSDLERQNQEYRI
LMDQKTHLEMEIATYKRLLDGHVFWRNPVDESIKLSPEVFWCLSLSSSYVTSGICFIQANLAWDMNKIHPDPYTIHIQKCNGTIIAAGGVIFVR
FQDHQKVIQFGPYLQELRPRSHSLGSLVQPLQHPKAMQQERDPRGHFPFLPQAMFDEYPPHDTFMRFPYHEWGGRGGGIGGKKSSYFCIYVVLD
YRVLQKLLIESPKRTERSQSQQVKGQVQGGMVRQ

>Nanorana1_25
MQHLNDRLASYLDKVRSLEQENAQLERNIQEWYERNQPSALPNFSSFFRIIQELQGQISSTSLDNARIILQIDNARLAADDFRNKYEMERQLRC
SVEADVNGLRILLEELNRDICNLQAQVGNLQEELQQMRRSHEEEVNALQAQLGARVSVEMNAAPSADMNRALSEIREQYENLMERNLREVETIF
RQRTEELNRDVSSGSEQLQSVQTEVIDLNRTVQSLEIELQSQMSLKSALENTLAETEATFGAQLAQLQCLINSVESQLAQVRSDLERQNQEYRI
LMDQKTHLEMEIATYKRLLDGHDIQ

>Nanorana1_26
MQHLNDRLASYLDKVRSLEQENAQLERNIQEWYERNQPSALPNFNSFFRIIQELQGQISSTSLDNARIILQIDNARLAADDFRNKYEMERQLRC
SVEADVNGLRILLEELNRDICNLQAQVGNLQDELQQMRRSHEEEVNGLQAQLGARVSVEMNAAPSADMNRALSEIREQYENLMERNLREVETIF
RQRTEELNRDVASGSDQLQSVQTEVIDLNRTVQTLEIELQSQLSMKSALENTLAETEATFGAKLAQLQCLINSVESQLAQVRSDLERQNQEYRI
LMDQKTHLEMEIATYKRLLDGHDIHTSSHNFLEGSDGSHHTMKAHHGTSHSNPHHTPLGSASFRHNLAWGMNKIHQDPYTVDTIEIMQGYGLVS
DMFITIGWW

>Nanorana1_27
MQHLNDRLASYLDKVRSLEQENAQLERNIQEWYERNQPSALPNFSSFFRIIQELQGQISSTSLDNSRIILQIDNARLAADDFRNKYEMERQLRC
SVEADVNGLRILLEELNRDICNLQAQVGNLQEELQQMRRNHEEEANALQAQLGARVSVEVDAAPSADMNRALSEIREQYENLMEQNLREVETIF
RQRTEELNRDVASGSEQLQSVQTENIDLKRTVQTLEIELQSQLSMKSAMENTLAETEAAFGAQLAQLQCLINSVESQLAQVRSDLERQNQEYRI
LMDQKTHLEMEIATYKRLLDGHDIHTSSHTFLGGSDGRWPSGSSSSRQNLARDKVHQDPYTVDTCEIMQGYGLVRDMLISIGANFTLEAWIQTG
NSSVRMAVLVTATTVVGFPYSGSGVRCQWSAFTNTTSLGLKG

>Nanorana1_28
MQHLNDRLASYMDKVRSMEQENAELERNIQEWYKRNQPSALPNFNSFFRIIQELQGQISSTSLDNASITLQIDNARLAADDFRNKYEKARQLRC
SVEADVNGLRILLEELNRDICNLQAQVGNLLEELQQMRRNHDEEVNALQAQLGARVSVELDAAPSADMNRALSEIREQYENLMEQNLREVETIF
RQRTEELNRNVASGSDQLQSVQTEVIDLNSTVQTLEIELQSQLSMKSALENTLAETEATFGAQLAQLQCLINSVVSQLSQVRSDLKRQNQEYRI
LMDQKTHLEMEIATYKRLLDGHDIHFLFMRPPVSASSRQNLAWDMNKIHHDTYPVDTIEIIFLFMRPPVSASSRQNLAWDMNKIHHDTYPVDTI
EIM

>Nanorana1_29
MQHLNDRLASYLDKVRSLEQENAQLERNIQEWYERNQPSALPNFNSFFRIIQELQGQISSSFLDNARIILQIDNARLAADDFRNKYVMERQLRC
SVEADVNGLRILLEELNRDICNLQAHIGNLQEELQQMRRNHEQEVNALQAQLGARVSVEMNAAPSADMNRALSEIREQYENLMERNLREVETIF
RQRTEELNRDVASGSAQLQSVHTEVIDLNRTVQTLEIELQSQLSMKSALENTLAETEATFGAQLAQIQCLINSVESQLAQVRSDLERQNQEYRI
LMDQKTHLEMEIATYKRLLDGHNIHTSSHTFLGGSDASHHTMKAHHGTSHRSASPRQNLAWDMNKMHPDPYTVDTIEIMQGFGLVRDMFITIGV
NFTLEAWIQTGDSSVRTAVLVTTTIIVGFPYGGVRSSVSVERLH

>Nanorana1_30
MSYSNKQNQSEKHKGSKILSQITSLSLRGFRKDNNAGTWRPISFQVKPSSSATVTKHHSFTHGANYGFTNSSFGKEVSLNRESKKHILLPTNEK
ETMRILNDRLASYLEKVRSLEEENAHLEKKIRDWYDTNHPQMLPDFSHYFAEMEELQRKISSTSLDNASITLQIDNARLAADDFRNKYEMERQL
RCSVEADVNGLRLLLEELNRDICNLQAQVGNLQEELQQMRRNHEEEVNALQAQLGARVSVEMNAAPSADMNRALSEIREQYENLMERNLREVET
IFRQRIEELNRNVASGSEQLQSVQTEVIDLNRTVQTLEIELQSQMSMKSALENTLAETEATFGAQLAQLQCLINSVESQLAQVRSDLERQNQEY
RILMDQKTHLEMEIATYKRLLDGHDIHTSSHKFLGGSDGT

>Nanorana1_31

MNHSQHKQSLSSSTSFKASVPVRSHSPKLSVHHGISHFSHHGGYHKVHHTAHNTHGGSSKPIMFSKHSSFPHGYGYGNVHGVHNNDSHFNSIGS
HSSWKNDGFLNVNEKETMQLLNDRLATYLEKVRSLEQENAQMEKKICEWHEKNVHSSMPDFQHYYKTIEELQNKISATSLDNARLVLEIDNARL
ATDDFKNKYELEIRLRTFVENDLKGLRMVLEGLNIERGDLQVQLECLQDEVLQMKKHHEEDVSSLRNQLGSRINVHVDAAPSVDLNRVLCEVRE
QYENMMEKNLKEVESIFILRSEELNRQMASGSEQLQSVQTDLIDLKRCLQTLEIELQSQLSMKSALECTLAETEATFGSQLIQLQSLIDNVEDQ
LSQVRSELERQMYEYKILMDQTTRLEMEISTYKKLMEADIHVPEHHPIHQEKIYKEISHGSSKHISTQEYDHGKGGHQTKEPSH

>Nanorana1_32
MQHLNNRLATYLEKVRSLEQENAQLERNIREWYERNQPSTFPDSSGFFRTIQELQSQISSASTENARIVLQIDNARLASDDFKNKYEMEMRLRS
SVESDVNGLRRVLEELNRERCDLESQVQSLQEELLQMKRNHEEEVNSLRAQLGARVNVEVDAAPSVDLNQSLSEIREQYENLMDRNMRDVENMF
LQRTEELNREMASGSEQLQSVQTEVIELRRSVQTLEIELQSHMSMKSALEGTLAETEATFGAQLSQLQCLINNVESELAQTRGDLERQNHEYKI
LMDQKTHLEMEIATYKRLLDGHDCKVPSPC

>Nanorana1_33
MKNNSSAASPMFKYGSFYFPEQGGFNSTHSGPSTLKTFHESNGMTLGRAYKNLYEHSKKSDHKRVNSPTRGGSYNSYHGETFDHCSSSSANGNL
WYKEIAHSPGHSYNMQSGGAHSKNYGRSKSNSLFKINERETMQFLNERLASYLEKVRTLEQGNGQMERRISEWYENNAPSALPDSSQFLKTIEE
VQKKIFAATVENANIALQIDNSKWATDDFSNKYEIEVRLRNNVGADVKGLQGILEVLHKEKLDLEFQVQELQEEVQEMKKNHEEEVNSLRGQLG
ARVSVELDAAPSIDLNSVLSDIREEYENLMERNLRDVEAMFLARTEELGCQVEPGSENQQSVQIEIIDLKRSVQNLEIDLQSQLNMNLALQDNL
TETEANYSSELAQLQRMINNAESELAQVRCDLERQNIEYKILMDQKNLLETEIATYKHLLEGQDSCVNKADSIASDITTSTDPEGSNPTECHPA
VKHSSEASC

>Nanorana1_34
MQMLNDRLSSYLDKVSALEQENAQLERNISEWYANNAPSSLPDSSQYFRIISDLQSQIAAATMENARIVLQIDNARLTADDFREKYEMELSISN
NTEADINALRRVLEGLNRDNCDLEMQVQNLQEELQEMKRNHEEEANCLRAQLGARVNVELNAAPSIDLNRALTEIRDEYENLMERNLSDVEDMF
RQRSEELNRQVTSGSEQLQSVQTEVIELNRTIQTLEIELQSQMSMTTALESTLAETQATYNSQIAQLQAMINNVECQLTQIRSDLERQNYEYKI
LMDQKTHLEMEIATYKRLLDGHNIHVSDYGMSSGKHGWRVTEEDDHTSASTYQMLLIDKTSANSKEMPPQAPIIADSSSPYMDSAGGYDLSGFT
HSIAMNHREKYRYSLARSYKNISRMGSPVLQMSSRPHHGCLTRFSHGGCSKSTGQSQFFYYGELHESHHRRSYMYDKVDSKAFGHGSSKAFLHS
GHHATSYGCKALQDTKNLPVSHGYNVHCHNTYLRNSREYDKCKNHSIFDFN

>Nanorana1_35
QKKTMIHLNERLSSYLEKVHSLEQDKERFEKKIAKWYENNAPGMPADLSKYFKTIQELQNQISTVFTNNVGIIRNIDNARMAVDEYNNKYEVEL
ILKTSIDADVNWLHAVLDGFNDQSVKLDSEADKLQQEMLKIRRDSEQDINFLTSQLGTRVSVEVEVSASADLNNALSGIREEYEELMEKNLRKI
ENLFLQRSEKLNQKVTSGSEQLQSAHTELIDVKHSLQTLEIELQREQYMISTLEETLAEKQAYYVSELAQLQGVINSVESQLDQIRTELESQIE
EYQLLMDKKTHLEQEISTYQYL

>Nanorana1_36
EKEIMQLLNERLSSYLTKVRSLEMENAQLDRKIFEWYASYQPKTPPDGTHYYRLIDELQNKIAEAAIENAWMLLETDNARLAADDYKTKLEMEQ
TLASNVEMDIQKLERVLEGLWGEAAERGMQVRSLQEERDCLRNNSEEVIESLLSQLGARINVELNAAPSIDLNEALSEIREEYESLMERNLGEA
ANMFLGQSEELTREMEFGSEELQSIETELIELKRCMQALEIELQSELSLKSALEGTLAEMEATFGSKLAKLQCLINGLESDLAQIRSDLERQNH
EYRTLMDQKNHLEMEIATYKRLLDGHDIE

>Nanorana1_37
MSHSNIHSSSRSLIDLGHCGGHKGSSHISSHHHGGLPAVHHKSSISHHGGHYKSPSVHGKVTRVSFSTHSSSGHGHGLRSGHGGHGHGSNISVA
GGHHQWKNAGFLSINGKETMQHLNQRLSTYLEKVHSLEQENAQLERKITEWYANNAPSSLPDSSHYFRTIQELQNQVSTANVDSARIILQIDNA
QLAADDLKSKYEMELNLRNNVEADVASLRKALEDINMQRQDLEIQLRCLQEELLQLKKNHEEEANSLRAQLGARVNVELDAAPSVDLNQTLSEV
RDQYENLMERNLKEVESIFLARIEELNSEMSSGDEQLQTVSNELIDLKRTAQTLEIELQSQLSMKSALESTLAETEGTFGSQLDQLQTLIDNVE
SQLGQIRSDLERQNHDYKILMDQKTHLEMEIATYKHLLDGHDIQYVKTSL

>Nanorana1_38
MSYKTFQPFRGFYRAGREYLGYPMYRSHTSSSDVKPKPYISHNVPYYRIHQVHVGSGGSGIRHSSSGQRDVMGNAHGFNRDHKIRQSSNRTGWT
NSKLFNFDEKKTMQSLNGRLESYLENVKQMEEENVIIEKKICEWYEKHSGMVYPDFSSYLHTVTDLQDQILLATEQIASLVKQISNGHTTADDF
RNKEEMNFKMRTLAEKDLCDHTGILETINMEKQNLNANIQNLEEELQQLMKTSEEEIKCLQAQLGTRVNVEVEMTPSIDLNKALSEIRKEYESH
MERNLSDIESKFYDLTSELSMMSSSIDQLQQFSNEAIDLKLQKKTLEAELKKQMSMISVQNSTFMEINEGYSSQLKYHQDVIDINELQMEEIQS
KVKQLNVEYELNANTKIFLEKEIATYKKHLEGQTTKYV

>Nanorana1_39
MNQTVKQVKSGSTQGRSQASSYNQTVRRTGSVVSYHGGRHQAQHGGHIRAPSVHGGSGGKGISISRHFSHGHSFGSVHGSSHGNHFVHQSTHGV
FKNDGLLSFNEKETMQLLNNRLATYMDTVCTLEQENSQLERNIREWYEKNEPNGLPDCNKYYRTIQELQRQISEASIQNAKVFLDMDNAKLAAD
DLLNKYELESNLRNSIESDVKGLHRVLERLNMEMGDLQMCVCNLQEELQELRKTHEEEVNCLRTQLGARVSVEVNAAPCKDLNNTLSKIRQEYE
NLMDRNLNEVEAIFLARSEELNSEMVFGSEQIQSVSSDLIELKRRVQTLEIELQSQLSVKLALEDSLAETEASYQSQLAQLQCLINNIESELGQ
TRFDLEQQNTAYKILMDQKNHLEMEIATYKHLLEGHHIHASVHSNNTEIHHGVKVQLTKGQVHQANVQLTKAQVHQAKC

>Nanorana1_40
MCGSGSTENRSGFSTYSLESYGSGKNDGLFSMNTKETMMVLNDRLATYLEKVASLEMENQRLERKIKVWYEKNVPQQLPDNSKYLKVIEELQKE
ILDARIKNNRVFLHIDNARLAGDDLQTKYNMELSLHNTIEADVVDLRKGLDGLTLERCELEFQLEQLSEDLILLKKNHNEEVDNLKEQLGARIH
VEVDVAPAKDINKVLASIRNEYESLMERNLKDVEKWFVTQSEELNSQMVSGAEHLQTVKYEVIDLNHTIQNLEIDLQTQLNTVG

>Nanorana1_41
MSFSFSQSGGVSSRLSDGIYRAASVHGGGYGGSRISMSSGGSARSGLGGGAGFGGGAGFGGGAGFGGGAGYGGGSSFSASMSAGSGGDGLLSGN
GKYAMQNLNDRLATYLDRVRSLEQANNDLEVKIRDWYAKQGSATVREQSFAGFYTGIDELRDKIFVATINNSKMILEIDNARLAADDFRLKFEN
ELSLRQSVENDINGLKRVLDDLTMTRSDLELQIEGLREELAYLKKNHEEEVGEKKEQAAGTVNVELDAVPGIDLLRTLNELREQYEHIADKNRR
EAEALFLSQAEALQNEVVTNTQQAQSVKSESTDLRRNLQGLENELQSLLSVKAGLESSVANVEDRYAAQLYQIQNIIGGIEAQLADLRSDLERQ
NQEYTTLLDIKSRLEQEIATYHQLMEGEGGK

>Nanorana1_42
MSYSYAHSSSSRAGSGLSSRISSGVLGSSSVHGGYSGSGISQSVHRLASSGFGGGSSYGGSGSSGFGSNIASSSGEGIFSGNEKYKMQNLNDRL
SNYLDKVKCLEEANTELEHKIHDWYEKQGSVTVREQSYSHYHTTIEELREKILAATNDNHQITLAIDNARLAADDFKLKYENELHMRMGVESDI
QGLRRVLDELTLARSDLELQIENLKEELAYLKKNHEEEMSDRGKQMSGTVNVEMDAAPGTDLCKTLGDMREQYEYIADKNRRDAEAWFVAQSES

LQKEVVSNTQQAQSSKTEITDLRRTMQGLEIELQSQHSMRAGLEASISETEGRYAAQLNHIQSVIGTLEEQLSNLRSELEHQNVAYRTLLDIKA
HLEAEITTYRKLLDEHDQSTGASHKESKGN

>Nanorana1_43
MSYSSRQTSVTSSYRGLGSGIGGGGGRVSFKSPSVHGGYGGQGVSVSSARLVSSGYGGGLGSGFGGGFSSGGGFSSGGGYSSGGGYGSGGGYGS
SFSVSTSGDGLLAGNEKETMQNLNDRLSTYLDKVRDLETANSDLEVKIREWYEKQGPSPTRDYGHYFKVIEDLRDKILGATIDNSRIVLQVDNA
RLAADDFRTKFENELALRQSVEADINGLRRVLDELTLARADLELQIETLKEELAYLKKNHEEEMNSLRGQVGGQVSVEVDSAPGTDMTKILSDM
REQYEHLADKNRKDAEAWFLSKTEEINTEVAVHTEQIQTSKTEITDLRRTLQGLEIELQSQLSMKASLEGTLSETEQRYGAQLAQIQARISNLE
AQLTDLRSDMERQSYEYKQLLDIKTRLEQEITIYRQLLEGHDTP

>Nanorana1_44
MAFYSSSSSYRTSGGSSGGAKGPGCRNSVSFSKYAPSGAGGAFGGNVGYCQAGDLGAGYDGEFAGVGYGGGASYGGGAGAGFGGGVCSGFGGGA
GAGFGGGANAGYGGGANAGYGGGANAGYGGGAGAGFGGGYGFNQGGGFGEGLLAVNEKQTMQNLNDRLANYLDKVRSLENANAELEKKIREWYE
KQSPVSKTADYSQYFKTIEDLRAKIFAATKENNKVILDIDNTRLTTDDFRLKYENELVLRQSVEADINGLRRVLDDLTLSKSDLESQIESLNEE
LTYMKKNHEEEMKGLQGQAAGSVNVEMNAAPGLDLSKILADMRADYEKLSEKYRKESEEMFLAKTKELQVQVVTGVQEVQTSKTEITNLKHTLQ
SLEIELQSQLSMKSALEASLADTEGRYCAQIAQIQDLIAKLEEELADLRNQLEMQTSEYKMLLDVKSRLEQEIAKYTQLLDGQDFK

>Nanorana1_45
MSVKQGVKGLGSSGSNSVRASSVRVCSGTQSHGGFYGGGAGGSLAGGYGGYGFSSGSGFGASSGFGHGGGPGFGIGGGHGSGFGYGQDQGLLNF
NEKNTMQNLNDRLASYLDKVQDLEQANAELEKKIRQWYESHGPKPSQDYSHYFKTIEEIQKKIHDATIDNGRITLQIDNARLAADDFKLKYENE
LFLRQTVEADINGLRRVLDDLTLSRSDLEAQLENLKDEKAMLKKNHEEDMKALQGQVQGTVNVDLNPTPGIDLQKILGDMRHDYESIMAKNKKD
MEDWYRVQTEELSQKMKKETHEVKSTQTEITDLRRTFQSLEIDLQAQISMKAALEGTLAETEGRYCMKLAEIQNMIQKVEEELANIRCDMSNQS
QEYQILLDTKARLEKEINTYRSLLDGQGTQISKGSQDYSSGSQSDKTGGSYNVRIRTEDSDGRVISTHDQKYQSGYRK

>Nanorana1_46
MSSRSIQGLSGFGGSSNRMYMASHRVPSIHESSGKNIYFSTSRLSSEVNGLGSNLGGGLGELGYGFGGGFGGGDGLLGGREKETMQNLNDRLAT
YLAKVHALEKANAELEIKIREWYEQQAPSTSYDYSQYYKAIEDLRNKIHSATFDNASLILQIENVKLAADDFRTKIETEHALRLNVEADINGLR
KILDDLTLNRSDLELQVESLKEELVYLKKNHEEEMNALHGEAHGTVSVEMDAAPAVDLTKILAEVRDKYETLSEKNRKEAEDWYFKKTEELNRE
VASHSEQIQSSITESTDLKRTLQSLEIELQMQLSMKSALEGTLAETESQYCVKLSQIQDLISGIQDQLAELRSDIERQNQEYKILMDMKTHLEQ
EITTYRRLLEGEDVQ

>Nanorana1_47
MASYSISQSSTSGSSRGLGSGGLGGGSSRVSMSGYRTPSIHGGSGGKNISISTSRMSSGGSGLGCSLGGGLGGAGGGYGGSYSSNFGYGSGSGF
GGGYGGGFGGGFGGGLGGGDGLLGGGEKETMQNLNDRLASYLDKVRSLEQANSELEIKIREWYEKQAPSPSRDYSQYYSIIEDLRNKILNASVN
NASVVLQIDNAKLAADDFRTKYETEHALRLNVEADINGLRKVLDELTLTRSDLELQIESLKEELAYLKKNHEEEMIALRGQVRGTVSVEMDAAP
AVDLSRILSEMRDQYEVMADKNRKDVEGWYIKKTEELNREVASHSEQIHSSKTEITDLRRSLQGLEIELQTQLSMKSALEGTLADTEGRYCVQL
SQIQDLIGSVEAQLAELRSDMERQSYEYKILMDVKTRLEQEIATYRRLLDGEDVKISYKESPQTSRQVRTIIEETVDGKVISSSERVQKAKY

>Nanorana1_48
MMSFSARSINQSGHFGGGRAGVSRARSVAGGAASVSMSSTSFNMAAPAFGAGFAAQQASNFAASGKETMQNLNDRLGSYLERVRCLEKANSDLE
VKIREFYERKSAISAFDPSGYYDTISKLRSQIQGATIENARHLLQIDNAKLAADDFKMKYEAELAIRQGVECDIGGLRKALDELTINRSDLELE
IEGLKEELIYLKKNHEEELAVSQGRIGGQVNVEMDSTPQVDLSKVLAGVRDQYEQMMEKNRQEVETWHRGQSENLRQEVALGQQSFQVNKSEVT
DLRRTSQTLELELQSLLNMKRALEGTLSETEARYGTEIAQLQSLISQLEIDLQQVRSDAEHQSHEYKTLLNIKTRLEQEIATYRRLLEGEDASM
TSVFNQSYVKIISKEEAQSSSTTQIKVKTVVEQVVDGKVVSSSVKEVTQKAKTTP

>Nanorana1_K18
MSFSNASYASLRSNRPFAYGPQMVGGSSAASVHAGAGGSGSRISVSRGATVGSGFGGGSGFSGGSGAVSSGMGLLGGVQNEKETMQDLNDRLAS
YLERVRSLESVNQKLELQIRQHMEKKGPTKDWAPYFKTLEELRKQVFDRTLDNSQLVLQIDNARLAADDFRVKYESEYAIRLSVETDIGGLRKV
IDDTNISRLNLENEIESLKEELIFLKKSHEDEVNEVHAQIASSSLTVEVDAPKTQDLGKVMSEIRAQYDTMAQKNREDAEKWYQAKVEEHTMQV
SQDTEALRSSKTTVTELRRSLQSLEIELETLRNQKQSLEGSLNDTENRYAMELEILSAQLQALEGELTQVRNDCQRQQHDYQILLNAKIRLESE
IQTYRRLLDGDDFSLQDALSASNVSTTQTVKKVITTTQRIIDGK
VVSESNDTHVLES

>Axolotl_K24L3
QVMQNLNERLASYLDKVRALEEANTELETKIRDWYQKRQGSAAAGSASDYSKYYQIIDDLRAKISAATIDNARVLLQVDNARLAADDFKLKYEN
ELAMRQSVEADINGLRRVLDELTMSRSDLEMQIESLTEELAFLKKNHQEELQGVSSSAAGQVSVEMDAAPGNDLTKILNDMRADYEALADKNRR
DAEAAFMKQTGELKAAISQEVSSVQSSKSEVSDLRRTLQGLEIELQSQMAMKSSLESTLAETEGRYGAQLCQMQQRISSLEEQLMQLRADMQQQ
SMEYQQ

>Axolotl_K24L2
NEKQTMQNLNDRLSSYLEKVRALETANADLEIKIRDWYAKQITSGTISPDHDHNKFFETINDLRSKIKSPSVDNARMVLQIDNARLAADDFRLK
YENELYFRQSIEADISGLKRVLEEQNMSRAELEVQIETMTEELTYLKTNHEEELQALQGQAAGQVNVEIDAAPGVALTKRLKDMRTDYEALTER
NRMEAEQWFIQKSMELKREMTKGVEEVQTTTTEVSEQRRLAQSVETEMQSQLAMKKSIEDSLIETENRYGVQLMQIQLIISTTEEQLGQVRGDM
ERQRSEYQQLLDIKVRLEMEIETYRRLLDEEG

>Axolotl_K15L1
IEELRAQLFQSTTSRTILEVDNARLAAEDFKMKYENELAMRQSVEADINGLRRVLDELTMSRSDLEMQIESQKEELAYITKSHEEEVGEMRTKM
TGQVSVEMDAAPGLDLTKVLTEMREQYEFMADKNRREAEATFIASSEQLQKEVLANVEQVQSGKGEITEIRRGMQGSEIELQSQYSMKVGLEAS
LSDTEARYGAQLLQIQSVISGYEAQLINLRSDMEQQSLEYKTLLDAKNQLEMEINMYRQLLE

>Axolotl_K24L4
NEKQTMQNLNDRLASYLDKVKALEDGNGELEKKIRDWYEKHRPGSASGDPGRDYSKYYPIIEDLKKKIIAATLENAKCMLQIDNARLAADDFKL
KYENELCMRQNVEADINGLRRVFDELTMCKSDLEIQLESLTEELAYLKKNHEEEMKSLSGGATGEVTVEMNAAPTTDLLKALNDMRGQYEAIAE
KNRSEAEARFQEQSKELKKEVSKGVEQVQSTKTEISDLRRSMQALEIELQSQLAMKRSLEETLAETEGRYCSQLNKIQITISGLEEQLNQIRMD
MENQSSEYQMLLDIKTRLEQEIQTYRRLLDGEG

>Axolotl_K15L2

SGAGGLFSSGGGFSSGGGFASGGGYSSGGFGGGFGGGFGGGAGESSGGLLGGNDKHVMQNLNERLASYLEKVQALESSNTELEGKIRAWYDKQG
ASGATATQRDYGHFYKTIDELRNQIMESTIGNSKVLLEVDNARLAAEDFKLKYENELGMRQNVEQDINGLRRVLDELTMSRSDLEMQIENLKEE
LAYLKKNHEEEMGEMTKQMSGQVSVEMDAAPGNDLTKVLTEMREQYEFMADKNRREAEAQFIASSEQLQKDVVANVQQVQSGKSEITEIRRSLQ
GLEIELQSQLSMKAGLEASLSETEGRYGAQLHQIQSVISGLEAQLINLRSEMEHQNQEYKTLLDVKNRLEMEIDMYRQLLDGQDAKSATWTSSA
AKGGSGSSSSATTKTSSTTQVRTIVTEEVNGKVVSSQ

>Axolotl_K17L
EKATMQNLNDRLASYLDKVRSLEQANSKLEMQIREWYEKQGPGPSRDYSPYYKTIEELRSKILAATLDNARTLLQIDNAKLAADDFRNKYETEL
GLRMSVEADINGLRRVLDDLTLARSDMEINIESLKEELAYLKKNHEEEMNALRGQVGGQVNVEMDAAPATDLSKILSDMRSQYELMADKNRKDA
EAWFFSKTEELNREVATHSQEIQSSKSEITDLRRTLQGLEIELQTQLSMKSALEGTLAETEGRYCMQLNQIQGMISNVEAQLGELRSDMERQGH
EYRILLDVKTRLEMEIATYRRLLE

>Axolotl_K19L
SEKETMQNLNDRLGSYLDRVRSLEVANTDLEQKIREWYEKQGPSPTRDYSHYFKTIEELKEKIFNATVDNAKVVLQVDNARLAADDFRSKFENE
QALRLNVEQDINGLRRVLDELTLSRTDLEMQIENLKEELAYLKKNHEEELGAMSGQVGGQVSVEVDAAPSVDLGRILSEMREQYEKMADKSRRE
AEEWFFTKTEELNKEVASHTQEVQTSRTEITDLRRTMQGLEIELQSQLSMKASLEGTLAETEARYGAQLQQIQGMISHIEAQLGDLRSDMERQN
QEYKMLMDIKNRLEMEIATYRQLLE

>Axolotl_K23L
NEKETMKNLNERLASYMDKVQELQEANKELEVKICDWHAQRATRGKRDYSTYEKTIADLQTQIVTGRMDNARLTLQMDNAKLAEDDFTTKHDRE
KAARMALEFDVERIRKLLDDMTIVKTDLEMEVEGLRKDLLCMKKEHEEEMSTLRAQQRSSNVSVELNAKPSHGLSKILKEVREEYEAIIEKYSR
EAGAHYKEQAKSFQQEVSTNTEYLENDNKEALELKRAYQDLQIELQAEISRKSGLERTLEETECNFAVDLQRKQQSVFQLEEELSNLRDQLAQQ
QNEHQTLLDIKNRLENEITTYRHLLE

>Axolotl_K15L3
TNYSAGGGSLGKDGGFGRDHQHRQTFSSVHGSQAGAGSGFGGGCYGGGSSYSGGSSYGDFSSGARRSSLPVNEKVTMQDLNDRLASYLEKVKTL
EETNHALEVKIQEFQRGPRTVLEQSWKTYEDTINPLKEKIKDMIISNGQKTLELDNIRLTADDFRTKYEMEVSLRRTVEDDINGLKELKQQAEL
GSSMLDQEMTSLQEEISDLKKNHEEELAEMRSQMEGTLNVEVDSAPSVDLNRIIANMREDYERVIKKHKQEAEAWYQSQVEAQHVEVVKTTQDV
QTSKSETRELQQKCQSLHAELDALHSVKASLEMAVGATEARYQEKVHQIAMRAAQVEAQLAALRGDLVQQNQDYQNLFNIKVKLEAEIATYRQL
LEG

>Axolotl_K18L2
FTASSPVKNGFSSAGVINGAVKVNDKETMRCLNDRLASYLEKVRYLEKTNQELEIKIQDKMKADAPVFRDHGPVFAQAHALSQEIAIASQENAR
LMLSIDNAKLAAGDFRMKWETEAAILQSVERDIAALRGVKDEHVGLLGSLRAQMESLEDELHFLKQNHKEEMDAMKESVANSKVDVEVDAVRGP
DLASILADVRSQYEEIVKKNKEEADALFQTKVDSLMMQVEQDTKASENAKEELTQRRRLLQSLEIELESLKNQTNALRGNLEEASLTYRNDMDG
LQESISRMEAELAEIQRNMQSEKFEYEALLRIKETLETEIAVYRRLLGGEEEEPSKIPPPKEPEPTTKKIVKVVTQTLVDGKVIEESSEVEEFE
VKKK

>Axolotl_K24L1
SSQSHSGGFRSSSAMSSGFGAGSSFSTSGAGGSGGGFSFGGGSFGGGAGFSLGGGDDKQVMQNLNDRLASYLDKVRALEEANTTLEVKIRDWYA
KQQANSGANASRDYSKYYQTIDDLKSKILAASVENARVVLQIDNARLAADDFKLKYETEMHMRQNVEADINGLRKVLDELTMSRSDLELQIENL
SEELAYLKKNHEEEMHSTAGGSGDVSVEMDAAPSLDLLQTLNSMRADYEAMAEKNRQEAEDMFKRKCAELNREIAVEVQAVSTGRSEMSELKRT
LQGLEIELQAQLAMKKSLEQT

>Axolotl_K9L
SHILAADIENGTLILAIDNTRLTADDFKLKYENELFFCQAVDADNNGLRRVLDELTVTKSDLEMQIESLTEELVALKKNHEDELKEGSQGQEAG
SVNVKMNAAPGVDLLKALNVMREDYEKLSAKYRKEAEDQFQAMSKDLVQQVATDSQATHSVKSDASSLRQSLQSLEIELQSQLSMKASLESTLA
ETEGRYCMQLNQLQIRISQVEEELGDLRADMENQSSEYQMLLDIKARLEQEISTYRSLIE

>Axolotl_K18L1
LQRQSSIGFQRFGPLGSSASMHAGAGGQGARISVSRVSTVGSGFGGGSGFGGGYGGGSSGSFSLSGSGNEKETMQDLNDRLASYLDKVRSLESA
NQGLEVKIREYLEKKGPSTKDWSPYFRTLEDLRRQVHESTVDNAQLVLQIDNARLAADDFRVKYEAELAIRTSVEMDIAGLRKVIDDTNMSRLN
MENEIESLREELIFLKKNHEDEVRNLQAQIASSGLTVEVDAPKTQDLGKIMAEIRAQYDVLAQKNREDAERNFQRQVEEVTIEVTRNTEDLQAS
RTTVTDLRRTLQGLEIELESMRSLKANLEDTLRDTEGRYGMEVDHINNMILRLEEDLTQVRNDIQRQAQDYQSLLNIKMKLEAEIETYRRLLDG
GDEFTLQDALSSSNASSTQMVKKVITTTQRVVDGKVVS

>Caecilian_K23
MSSSRPFWSSSGSVKGGEGRLIHIDSSYSTSSVHGGAGGSRVSVSTARPLASSGLGWGSSGNNFGGGSGAHVLFTNDKETMQNLNARLASYLEK
VQSLEKANAELEEKIKKWHEERTPQNKRDYSKYEKIIADLHAQIANGRLNNARLTLQMDNAKLAAQDFKLKYETEQTLRMAIEADVNGLRKLLD
DLTIVRTDLEMEVEALRKELILMKMVHEQEMDALQARQRSSTVDVQVAAAPSVDLTKILAEMRQEYEAIIEKNREEAASWYKQKSSTMQQEVTT
NTEALESSRKEVNQLKRTLQSLEIEMQSELSKKCSLENTLAETQANYAVKLKNVQTVICQLEEELANVRIDIERQGNEYKILLDIKSRLENEIA
TYRQLLDGDDVRTDTSEFSKETNRKVKTIVQDVIDGRVVSSKVSEIHQK

>Caecilian_K117
MASFSSRSFSQSFSSQDTSVRGPQVGLLSSSQKGGRRAFSVVGGAGGTGARISSSSASFVKAGIGGGYGSAFGIGGGFVVGFAGGVLAKDRGSL
SMNEKETLQNLNERLATYLEKVRSLEKANGQLELQIRQFFEGRTPVAERDLSGYYDEINTLRSKIQEATLDNARLVLQIDNSELAADDFKLKYE
NELRVRLGVEADNSGLRKVLDELTLSKSNLEMQIEGLQEEVIHLKKNHEEEQANLRSQLGGSVSVELDSTPGIDLTQIMADMREQYEEMIKKNR
EEVEAWHTAQSEALNREVATHTEAIQTSKGTITDLRRSIQGLEIEMQSGISMIGSLESTRSETETRYAIQLQQLQDSISRLEAGLFQVRADASR
QAIEYTQLLDVKNRLEAEIATYRHLLDDEDSSKSSVYELQKVSSIISSRGKSSGRPY

>Caecilian_K17L
MSTSYSIRQTSSTSGSSMGLGGFGGGPSRFSSVHLENYGAPNMFGGAGGRGVSASKSRLVSGLGAGFSNGYGGDFISSYGVGGGDVFLPGGEKE
TMQNLNNRLASYLDKVRSLEEANSKLEIQIREWYQKQGPGPERDYSSYYKLIEDLQSKIIAANLDNARVLLQIDNAKLAADDFRTKYENELALR
MSVEADINGLRKVLDELTLTKADLEMRIESMKEELIYLKKNHEEEMNAFRGQVGGRVNVEVDAAPSVDLTKILAEMRNQYETIAEKNRKDAEDW
FFSKSEELNKEVATHAEQIQTSKSEITDLRRTLQGLEIELQTQLSMKAALEGTLAETEGRYCMQLSQIQDLISSVEAQLADLRSDMERQSYEYK
ILMDVKTRLEMEIATYRRLLDGEESIGSGKKDVSQTTRKVRTIIEEHVDGKIVSSREQVHHSTY

>Caecilian_K19L

MASSYSMRQTTYSGSSGLGGSGGRLSFKSPSVHGGYGGKGVSVSTARFVSSGAGGGLGAGGFGSSYGGSYGGGSYSVTGGDVLLSGNEKETMQN
LNDRLATYLEKVRSLEAANTDLEVKIRDWYEKQGPSPSRDYSHYLKIIEELRDKIMGATIDNSKVVLQIDNARLAADDFRNKYENELALRQSVE
ADISGLRRVLDEMTVSRADLELQIESLKEELAYLKKNHEEEINALSGQVGGQISVEVDAAPPIDLNKTLAEMRGQYENMVEKNQRDAEAWFITK
TEGLNKEVATHTEQIMTSKTEITDLRRTVQGLEIELQSQLSMKASLESNLAETEARYGAQLAQIQSLISSLEAQLTDVRSDMERQNYEYKMLMD
IKTRLEMEIATYRQLLEGQDSQLSGGSFKTGSK

>Caecilian_K15L
MSYSFVQNTSSRGGSGVSSRLSSGGPHVAHRAASVHGGYGGSGISASSGRMVSSGFSSGGGFASGGGFGGGYGGGFGGGYGGGFGGGFGSCSGG
ADMLLTGNEKQTMQNLNDRLANYLDKVRSLEEANTDLERKIREWYEKQVSKPGSTGGRDYSQYYKTIEDLRDKILASTIDNAKIVLEVDNARLA
ADDFRMKYENELALRQSVEADINGLRRVLDELTLNRADMEMQIESLKEELAYLKKNHEEEMGELGNQMTGQISVEMDAAPGVDLTKILNDMREQ
YERLTEKNRKEAETWFFSQSEQLQKEVSTNVEQVQTSKTEITDLRRTLQGLEIELQSQLSMKAGLEATLSDIECRYANQLVQMQSVISSVEAQL
ADLRSDMERQNHEYKMLMDIKTRLEMEIATYRQLLEGHDAQITVGGSGGIGGSGSTSTTTKITTTKEERRY

>Caecilian_K24L
MQNLNDRLAAYLDKVRSLEEANTELERKIKEWYDKHRPGSSTGGPGRDYSKYYQAIDDLNKKIIDATIDNGRIILQIDNARLAADDFKLKYENE
LVLRQSVEADINGLRRVLDELTLTKSHQEMQVESLTEELLFLKKNHEEEVKGLKGTPVGDIDVQMNAAAGIDLTKILNDMRAQYEGLAEKNRRE
AEEQFHKRSGDLKQQISSNLQQGQTSKTEMTDLRRSLQALEIELQSLLAHKKTLEETLAETEGRFCLQIAQMQATISSIEEQLAAIRADMEQQS
SDYEQLLDIKTRLEKEIETYRRLLDGQGGTKQGYKSENNY

>Caecilian_K24L2
MAFQAGSVSIRRAGGSISGQHGGDSLWGQGGSGGHVGSQSLRVGGSHGGWQAGSGGWQAGSGGWQAGSGVAHAGSVVGHAGYGVGQAGFGVGQA
GFGFGFDQAGFGGSGFGDGAFGSVQVIGGEGLFPGNEKETMQNLNDRLAAYLDKVRSLEEANTELERKIKEWYDKHRPGSSTGGPGRDYSKYYQ
AIDDLNKKIIDATIDNGRIILQIDNARLAADDFKLKYENELVLRQSVEADINGLRRVLDELTLTKSHQEMQVESLTEELLFLKKNHEEEVKGLK
GTPVGDIDVQMNAAAGIDLTKILNDMRAQYEGLAEKNRREAEEQFHKRSGDLKQQISSNLQQGQTSKTEMTDLRRSLQALEIELQSLLAHKKTL
EETLAETEGRFCLQIAQMQATISSIEEQLAAIRADMEQQSSDYEQLLDIKTRLEKEIETYRRLLDGQGGSGSSSSGSSSSWSSGQAAKVPNKVT
KVRTIIEEIHDGKVVSTRVKEES

>Caecilian_K24L3
MATFRTFQSFVNAPSLSDSCSRVSLQSSQGGGIGFGGFSGGSGASYGFNSSFGGGFGNSFSGGFGGGEGFFVASEKQTMQNLNDRLAAYLENVR
ALESANLELESKIKAFYEKQTGAGAITSRDYSKYYQIIEDLRSKIFDATVDNARILLQIDNARLAADDFRLKYENELTLRQSVEADINGLRRVL
DDLTLAKTDLEMQIETLTEELAFLKKNHEEELKTLQGTTSGQVNVEMDAAPGVDLTKILNDMRANYEALAEKNRRDALEWYNQKSGELKKEIST
GVQQVETSKTEITDLRRTLQGLEIELQSQLAMKKSLEDTLADTECRYGAQLVQIQGVITGVEDQLAQIRNDMERQNAEYRSLMDIKTRLEIEIE
TYRRLLDGELGGLSSFQSSSNTMTSQSGSGGSGSAASVDSKKDPTKTRKIKTIVEEVVDGKVISSQVKESEEMIRY

>Caecilian_K24L4
MATFRTYSSLAVPKTKNSVQNYGGSSTAGFSGASLGGISGGFGGGKGGSFGGSQSAGFISGSGTGFGGGYSVGFGGGKGGSFGGSQSAGFLSGS
GTGFGGGYSDGYGGGFGYAGAGFHGVSFGGDSLLSGNEKQTMQNLNDRLAAYLDKVRALEEANTELEQKIKEWYQKHGHGGITGGPARDYSKYY
QMIEDLKNQIIAANIDNARIILLIDNARMAADDFRLKYDNELALRQSVEADINGLRRVLDELTLSKSDLEMQLESQMEELTFLKKNHEEEMKSL
TGTTVGEVSVEMDAAPGIDLTKRLNDMRAQYETLAEQNRKEAEARFLEQSRELKKEISAGVEQVQSSKTEITDLRRTLQSLEIELQSQLALKNS
LESTLAETEGRFCVQLANLQSRISSVEEQLLQIRSDMEHQSSEYKMLLDIKTRLEKEIETYRHLLDGEGGSSSTTYRSSTYSMSS

>Caecilian_K20L1
MALSGRIPSLGSVTYSSQMSGSRHRPPSAYGGAGGFGTRISVPSYNTGLVFTGGADGSFQFSTSAKDLPFSGNEKSTMQNLNERLASYLSKVHS
LEKANSKLELQIKEWYEKRAPVAGGDYSQYYQVIEELRTKIHDIRLDNAKTLLQIDNAKLICDDFRIKYEKELAIHHGVECDIQGMHRVLDELT
MTKADLELQIEGLQEELMYLKKNHEEEVVELRKQLGGNVNVELDAAPGKDLGKLLADMRLQYEELTKKKRQEVKDWFDKQTEELNAQVMVSTAE
TKTSKNEITSARHTAQELEIQLQSDISKKAALENTLSETEARYGMQLAQIQTIITSLEAQLARLRTDAQSQSKEHMLLMDVKIRLEMEIATYRR
LLDGEDNRSEALELEDALKEQSKSRKIKTIIEEVVDGKIVSSQVREVEEKM

>Caecilian_K24L5
MAFSSNFLSRTSMSKAQSGPHGGSFGSNFSSRSFSGAGFGGGMSSGFGGGMSSGFSGGNSFGFTGGNVLLTGGEKQTMQNLNDRLAAYLEKVRA
LEEANTELELKIHQYYEKHLNSKNPSTRDYTTYYQIIEDLKNKIFTATMDNTRILLQVDNARLAADDFRLKFQNEQVLHQAVEADTNGLRKVLD
ELTLSRSDLEMQIENLTEELAFLKKDHEEEMSSIQVGTGQVNVEMDAVPGADLTKILNDMRDNYENLTERYRRDAEAWYTDKSNDLKKEISTGI
EQVQSSKTEISDLRRTFQTLEIELQSQLAMKKSLEDTLADTEGRYCTQIAGMQGTIGSVEEQLAEIRGDMSRQNAEYQQLLNVKTRLEMEIETY
RLLLDGEPSNAYVKSQAESNSTSQYGSNSQYASTAEELKKDQTRTRKIKTIVTDMVDGKVVSSQEEIIEEKIN

>Caecilian_K24L6
MAFQAGSVSIRRAGGSISGQHGGDSLWGQGGSGGHVGSQSLRVGGSHGGWQAGSGGWQAGSGGWQAGSGVAHAGSVVGHAGYGVGQAGFGVGQA
GFGFGFDQAGFGGSGFGDGAFGSVQVIGGEGLFPGNEKETMQNLNDRLAAYLDKVRSLEEANTELERKIKEWYDKHRPGSSTGGPGRDYSKYYQ
AIDDLNKKIIDATIDNGRIILQIDNARLAADDFKLKYENELVLRQSVEADINGLRRVLDELTLTKSHQEMQVESLTEELLFLKKNHEEEVKGLK
GTPVGDIDVQMNAAAGIDLTKILNDMRAQYEGLAEKNRREAEEQFHKRSGDLKQQISSNLQQGQTSKTEMTDLRRSLQALEIELQSLLAHKKTL
EETLAETEGRFCLQIAQMQATISSIEEQLAAIRADMEQQSSDYEQLLDIKTRLEKEIETYRRLLDGQGGSGSSSSGSSSSWSSGQAAKVPNKVT
KVRTIIEEIHDGKVVSTRVKEES

>Caecilian_K20L2
MAATRWLNLWNDVYKMPGQRLYRSRSFGGGAFPSGGYLDGGEKLTMQNLNERLAIYMDKVSSLEKANSTLEVQIKEWLEKQTPVEGGDYNKYYQ
VIKELQAKILKDQIESGKIVLRMDNIKLASEDFRLKYNNEQGMRMIMENDIQRMRQIRDELTVGNCNLELELEELQEQLIYLKKNHGEEVEVLR
KNMVGKVSVEVDAVPGGGIEKVMADMRLQYEELTKKKQQEAKELFDKQTKELNMQVTVSTEEMNKRRLRLPGSLRPFETWR

>Caecilian_K24L7
MAFKQSAGSTRVSMGGGSTRVSMGGGSTRVSVGGGKGNFGGAACDAAGVSGGGSFGFAGGQGGFGGGHGGAFGSVQGGSFGGGFGGGQGGSFGG
GFGGGQGGGFGGGFGGGQGGSFGGGFGGGQGGGFGGGFGGGQGGGFGGEFGHGGEGLLSGTEKETMQNLNDRLAAYLDKVRALETANSELEQKI
KEWYEKHRPGGSTGKGPRDYSKYYPIIEDLKNKIIAATIDNAKIILQIDNARLAADDFRLKYENELTLRHTVEADINGLRRVLDELTLSKADLE
SQLESLTEEYALLKKNHDEEIKGLQSSGVGQLNVEMNAAPGTDLTKLLNDMRGQYEDLAEKNRKEAENRFNEASKALKQEISAGAEQMQSSKTE
ISDLRRILQGLEIELHSALAMKKSLEDTLAETEGRYCMEIAKIQAMISSTEEQLSEIRADMENQSAEYEVLLDIKTRLEMEIETYRRLLDGEGM
GSGRGYQHDSSSSSRGSHGYQHDSSSSLSSRESPEPNKTRKIKTIVEDIVDGVVMKSTVQEREEAMK

>Caecilian_K24L8

MPRSPTRAEREQIPGDKGEIPTLQAARNHPYLRPTTNQIAYLNPDAEISLLPDRDVPESLDIRESCKSPRSAPYAHPPNLGWTTVGDACLKRTR
KSNVKGTCLHSREAIKDQNVSTFSVPLFSFLTLPERAAFLLATMAFSSKQSSGSVRISGGGAGAQFAGSQSGGYGAQSFGGGQFGGFGGGQGGG
FGGGQVGGFGGSQVGGFGGGQGGGFGGGQGGGFGGGFGGAQGGFVGGSFGGGESLLSGSEKETMQNLNDRLAAYLDKVRSLEEANTELERKIKE
WYDKHRPGGTAGGPARDYTKYYKMIEDLTNQIIAVTIDNAKVILQIDNARLAADDFKLKYENELALRHSVEADINGLRRVMDELTLSKADLESR
LESLNEEMMSLKKNHEEEIKGLQSSGAGQLNVEMNATPGNDLTKLLNDMRGQYEDLAEKNRREAENRFNEASKALKQEISAGAEQMQSSNTEIS
DLRRTLQGLEIELQSALAMKKSLEDTLAETEGRYCMEIARIQAMISSTEEQLSDIRADMENQSAEYEMLLDIKTRLEMEIETYRRLLDGEGIGQ
RSSYPPPTTKPATRGTQKTRKVKTITDEVVDGVVISSKVQEYEEKV

>Caecilian_K24L9
MAFQAGSVSIRRAGGSISGQHGGDSLWGQGGSGGHVGSQSLRVGGSHGGWQAGSGGWQAGSGGWQAGSGVAHAGSVVGHAGYGVGQAGFGVGQA
GFGFGFDQAGFAGSGFGDGAFGSVQVIGGEGLFPGNEKETMQNLNDRLAAYLDKVRSLEEANTELERKIKEWYDKHRPGSSTGGPGRDYSKYYQ
AIDDLNKKIIDATIDNGRIVLQIDNARLAADDFKLKYENELVLRHSVEADINGLRRVLEELTLTKSHQEMQVESLTEELLFLKKNHEEEVKGLK
GTPVGDIDVQMNAAAGIDLTKILNDMRAQYEGLAEKNRREAEEQFHKRSGDLKQQISSNLQQGQTSKTEMTDLRRSLQALEIELQSLLAHKKSL
EETLAETEGRFCLQIAQMQATISSIEEQLAAIRADMEQQSSDYEQLLDIKTRLEKEIETYRRLLDGQGGSGSSSSGSSSSWSSGQAAKVPNKVT
KVRTIIEEIHDGKVVSTRVKEES

>Caecilian_K24L10
MALRTQTTSHRVISKSGGGSLVGGMGSSGMKFSGGSSYGSFSGGSLGGGFGAGSMSAGGGYGSSQGFGVGGFQGAGFDVSGVGGGAAFGSGAGF
GGGAGFGGGAGFGGSAGFGGGAGFLSAGYGGGFGGGDSLLSGTEKQTMQNLNDRLAAYLDKVRALEEANTELEIKIREWYEKHRPTGPSGAGAR
DYSKYYPIIEDLKNKIVAATIDNARLILQIDNARLAADDFRLKYENELYFRQTVEADINGLRRVLDELTLSKADLESQLESLIEEKEFLKKNHE
QEMKSLSGTTVGEVNVEMNAAPGVDLTKLLNDMRTQYETLAEKNRRDAEEQFNKKSLELRKEISAGTEQMQSSKSEISDLRRTLQGLEIELQAQ
LALKKSLESTLAEIEGRYCLQLSQIQATISSVEDQLAELRSDMSRQTSEYEALLDIKTRLEMEIQTYRRLLDGEGMSSHSGGSSWSTGGDSDSG
KRDPTKSRMIKTYIEDIVDGKVISSRVNVTEEKI

>Caecilian_K24L11
MASQTYSSRQSSSVRIGGGSAGAQYAGSQSGGYGAQQSFGGGQIGGFGGSQVGGFGGGQGFGFGDGFGSGQGSGFGGGQGGGFGGGQGGGFGGG
QGGGFGGSFGGAQGGGFGGGESLLSGSEKETMQNLNDRLAAYLEKVHSLEQANTELEQRIKEWYDKHRPGGTTGGPGRDYSKYYQMIEDLRNQI
IAATIDNAKVILQIDNARLAADDFKLKYENELALRHSVEADINGLRRVMDELTLSKSDFESQLESLTEEYAMLKKNHDEEVKGVQSSGVGQLNV
EMNAAPGIDLTKLLNDMRGQYETLAEQNRKDAENRFQEASKSLKQEISAGAEQVHTSKSEITDLRRALQGLELELQGLLATKASLEQTLAEREG
HYCVEIAKIQATISAIEEQLSDMRADMENQSAEYEILLDIKTRLEMEIETYRRLLDGEGIGSHSGSQGSSGWQGSQGSHSSSSSATKEPNKTRK
IKTIVEDIVDGVVMKSTVQEREEAVK

>Caecilian_K24L12
MASQTYSSRQSSSVRIGGGAAGAQFAGSQSGGYGAQQSFGGGQIGGFGGSQVGGFGGGQVIGFGGGQGSGFGDGFGSGQGSGFGGGQGGGFGGS
QGGGFGGSFGGAQGGGFGGSFGGAQGGGLGGGESLLSGSEKETMQNLNDRLAAYLEKVHSLEQANTELEQRIKEWYDKHRPGGTTGGPGRDYSK
YYQMIEDLRNQIIAATIDNAKIILQIDNARLAADDFKLKYENELALRHSVEADINGLRRVMDELTLSKSDFESQLESLTEEYAMLKKNHDEEVK
GVQSSGVGQLNVEMNAAPGIDLTKLLNDMRGQYETLAEQNRKDAENRFQEASKSLKQEISAGAEQVHTSKSEITDLRRALQSLELELQGLLATK
ASLEQTLAEREGHYCVEIAKIQATISAIEEQLSDIRADMENQSAEYEILLDIKTRLEMEIETYRRLLDGEGIGSHSGSQGSSGSQGSSGWQGSS
GSQGSSGWQGSQGSHSSSSAAKKEVQKVRKVTTITDEMEDGKVISSKVNKYEEKM

>Caecilian_K18L2
MASLHVPRSLSSSHQTPHSTTRTSWLKFSSAPAMKIHGVSPDKGDFDFSVFTPGIFESNKKETMQNLNNRLASYLEKVHALEKANEKLESQIKE
KLTKVSPSRKDYGPLFAQIIALRNKIDNAVKDNFQLQLQIDNTQLAKDDFKQRWEFAAAECESYKRDLQILKKVKEEHKSRLGVLRVQLESWED
ELLSLKKSHKEELAALRASLVGPQVDVEVDVVRGPDLALILAGIRSQYEAVVQKNEEDAEAWYKTKVSTLPVQFWKENEALEDAKSELSEKQRN
LKSLQITLESLKKQVNALDGTLQETEFCYSTKLHEIQKMIASLEKEFVEVKCSMQSQKNEYEALLLIKETLEAEIEVYRRLLEGDIQKKLIAPQ
PVVPDVKTRKIVKIITQTLVNGELVDMTSDIKEIEESS

>Caecilian_K18L1
MAQPLSFSSASSHRSSVSGSGRPWPLSASSMSGVSASLGSRLKMALPALRSSMDSLLQQNNHQEMMQALNERLAGYLQRVRGLEATNHRLEDEI
CEITARRGPCERDWDSYERPLVELRKQVEDLNMDNAKLLLQIDNARLAADDFRVKLEAEQAICDGVQKDTMGLRKMIEDTSHLRMKLDSELEVM
KEEYAHLRKSHKEEVEALRDMIANNNVKVEVDAPKKRDLNDSVAEVRSQYEKMAEQNRADAENWYKDKFDNMTQEANVNTQALEQAKTEMTELR
RQLQNLEIERQTLQKTVDSLENTLRETEDHYGNNLANLNRILNKLQEELAACRADIERQVRDYEVLLNLKTKLEDEINHYHRLLEGASEGVAPK
PPAATGPGRQTVKKVTVTTQEIVNGQVVTSKSQAVIE

B

>Platypus_K80
MVMSSVRQVFVSHYPDQAQGRRGIIAVPGATSLRSSPSPPSPTPPLPPRHPRAMAGLPQSFSSLSGCGPSGLGWRMSGEAGFSSCSFTGGDAPH
SFCKVTVDPCLLLPLDIKVDPAIQQQKAREREEMKTLNDKFVSLIGKVQYLEQRNQLLETRWAFLKEQESGPINCSNIYEAYTTRLQTELADVS
QERGRLEAELQQVLGSVDEYRIRYEEEIAKRTDMEFTFVQLKKDLDAECLRRTELETKLQGLQSFVELMKNVYQQELQNLMAEVKDVSVTVGMD
SRCHVNLNGIVEEVKAQYDAIAAKSLEEAEAYSRNRLEERQARSAECGNSLQASRSEISDLNIQIQKLRSQKLSLKSHCLHLEESIKEAEEQGN
VAFQDAKAKLLQLEAALHKAKQDMALQLRDYQELMNVKLALDIEIATYRKLVEGEESRMDCPSSAVVSTVQSRNRPAKATPLGPSQSLSCKKKC
EKKGPVIKITELTEKLLSQESEATQ

>Platypus_K7
MAMHFSSRSAVPPGRVSFTRVSSVGSTRSTGGFGSSSLYNLGAGGGRKVSSAGRYYGVAGSAGFGPGAGIQEVSINPSLLAPLNLEIDPQHQKV
RKEEREQIKVLNNKFASFIDKVRFLEQQNKMLETKWSLLQEQKSGKSSNIPTLFESYINSLRHQLDGLLSDQGRLEGELKSMQDLVEDFKNKYE
DEINKRTSAENEFVVIKKDVDNAYMNKVELEAQVDSLTDEIKFLRSLYELELNELQAQISDTSVVLSMDNNRSLDLDGIIADVKAQYEEIAQRS
RAEAETMYQIKFETLQAKAGKHGEDLRNTKTEISEMTRNIQRLQSEIDNIKGQRAKLEAAIAEAEERGELALKDARAKQDELEAALQKAKQDMA
RQLREYQELLSIKLALDIEIATYRKLLEGEESRLAGDGIGSVNVAMVNNSRSYRSQGQNLGFGGTMGLNALNFGVRPSTSPGATGVNQTYSYQF
TTSSQKSDRN

>Platypus_K81L

MSCGSGFGGLRAFSCVSACGPRPGRCCISAAPYRGVSCYRGLTGFGSRSLGGFCGGYRAGCGRSFGYRSGGVCGPSPPCITSVSVNESLLAPLN
LEIDANAQGVKHEEKEQIKCLNSKFAAFIDKVRFLEQQNKLLETKWQFYQNRKCCESNLEPLFDGYIETLKRELECVEADRGRLESELYHVQEV
LEGFKKKYEEEVTLRATAENEFVALKKVSDVDCAYLRKSDLEANAESLTEEINFLRALYEEEIRVLQAHISDTSVIVKMDNSRELNMDCVIAEI
KAQYDDIATRSRAEAESWYRSKCEEMKATVIRHGESLRRTKEEINELNRMIQRLTAEVENAKCQNSKLEAAVTESEQQGEAALNDAKCKLAGLE
EALLKAKQDMACLVKEYQEVMNSKLGLDIEIATYRRLLEGEEFRLCEGVGAVNVWLSSSRGGVVCGDLCTTGARPVTGGVCSAPCSGNLVVSTG
GPCVPCGPCGPSVGSFSAGSCGLGSGGVGSCGISSYGVGSCVSTCRKC

>Platypus_K83L2
MSCGSYCAGSGLGLRAFSCVSACGPRPGRCCISAAPYRGVSCYRGLTGFGSRSLGGFCGGYRAGCGRSFGYRSGGVCGPSPPCITSVSVNESLL
TPLNLEIDPNAQGVKQEEKEQIKCLNSKFAAFIDKVRFLEQQNKLLETKWQFYQNRKCCESNLEPLFDGYIETLKRELECVEADRGRLESELYH
VQEVLEGFKKKYEEEVTLRATAENEFVALKKDVDCAYLRKSDLEANAESLTEEINFLRALYEEEIRVLQAHISDTSVIVKMDNSRELNMDCVIA
EIKAQYDDIASRSRAEAESWYRSKCEEMKATVIRHGESLRRTKEEINELNRMIQRLTAEVENAKCQNSKLEAAVTESEQQGEAAVNDAKCKLAG
LEEALLKAKQDMACLVKEYQEVMNSKLGLDIEIATYRRLLEGEECRLCEGVGAVNVCVSSSRGGVVCGDLCASGARAVTGGVCSAGCGGNVVVG
TGGGCVPCGGVSACSARSCTTSCNRC

>Platypus_K83L1
MACGSGFGFRNFGCRSALVPRFGDRCCISAAPYRGVSCYRGLAGFGSRSLGGFCGGFRAGCGRSFGYRSGGVCGPSPPCITGVSVNESLLTPLN
LEIDANAQGVKHEEKEQIKCLNSKFAAFIDKVRFLEQQNKLLETKWQFYQNRKCCESNLEPLFDGYIETLKRELECVEADRGRLESELYHVQEV
LEGFKKKYEEEVALRATAENEFVTLKKDVDCAYLRKSDLEANVEALIEEINFLRALYEEEIRVLQAHISDTSVVVKMDNSRELNMDCVIAEIKA
QYDDIASRSRAEAESWYRSKCEEMKATVIRHGESLRRTKEEINELNRMIQRLTAEVENAKCQNSKLEAAVTESEQQGEAALNDAKCKLAGLEEA
LLKAKQDMACLLKEYQEVMNSKLGLDIEIATYRRLLEGEECRLCEGVGAVNVCVSSSRGGVVCGDLCATGARPVTGGVCSAPCSGNLVVSTGGP
CAPCATPAPCGPGGSCSGSVGVGSYGLGAFGFGKC

>Platypus_K85L2
MSSRAPRLSAGCGAGALSSRSAVVPRAHSCISAVAYRSGGVPIPGRSLGGAVSSRIAGGRGRSLGAGPGFGYRSGGVCGPSPPCITAVTVNESL
LTPLNLEIDANAQCVKHEEKEQIKCLNDKFAAFIDKVRFLEQQNKLLETKWQFYQNRKCCESNLEPLFDGYIETLKRELECVEADRGRLESELS
HMQEVLEGFKQRFEEEVALRATAENEFVVLKKDVDCAYLRKADLEANVEVLMEEISFLQALFEEEIRLLQSQISDTSVIVKMDNSRDLNMDCVV
AEIKAQYDDIASRSRAEAESWYRSKCEEMKATVIKQGENLRRTKDEINELNRLIQRLTAEIENTKQQRSKLEAAVAEAEQQGEVAVSDAKAKLA
GLEAALQKAKQDMACQLREYQELLNIKLALDIEIATYRRLLEGEESRLCDGVNSVNICVSRSGGGVACGDLSSSVSRGTTISSGTSCPTSRGVS
CSSARSVRFA
>Platypus_K85L1
MSCRSYRISSGCGVSRTFSSSSAVAPRVGDRCCISAAPYRGVSCYRGLTGFGSRSLSAVGACGPRISMGGYRPGCGRSFGYRSGGVCGPSPPCI
TTVSVNESLLTPLNLEIDPNAQGVKHEEKEQIKCLNSKFAAFIDKVRFLEQQNKLLETKWQFYQNQRCCQSNMEPLFNGYVETLKRELECVEAD
SGRLVSELNHMQEVLEGYKKKYEEEVALRATAENEFVVLKKDVDSAYLRKSDLEANAETLTEEIDFLRSLYEEEIRLLHSQISDTSVIVKMDNS
RELNMDCVVAEIKAQYDDIASRSRAEAESWYRSKCEEMKATVVRHGESLRRTKEEINELNRMIQRLTAEVENAKCQNAKLEAAVAEAEQQGEAA
LNDAKCKLAGLEAALLKAKQDMACLLKEYQEVMNSKLGLDIEIATYRRLLEGEESRLCEGVGAVNVCNSSRGGVTCGGFSCSTTPGRQISTGSS
MSGGSYTVMAPDSCAPCLPRGPGFSCGSSRSVRFA

>Platypus_K84
MSCRSLISSRRCTSGFSSCSALAPQASSCQRISTVSCRSGVGSGRRAFAGFGSRSAVSFGSCSPRMAVASSFPGRCGFGFGGGYGGSYRLGRAG
GFGYRSGGVCGPSPPCITAVTVNESLLTPLNLEIDPNAQRVKHEEKEQIKGLNTKFATFIDKVRFLEQQNKLLETKWRFMQQQKSSRSNLEPLF
ETYIRTLQQRLDSAASDRARLESEYSQIQGVLEGFKNKYEEEVGFRAAAENEFVALKKEVDMAFLNKADLEANLNTLVQENHFLRTLYEEEIRL
LQSQISETSVIVKMDNSRDLDLDGIIAEVKAQYEEIAQRSRAEAESWYQSKYEEMRIAAGQHNDSLRNSRNNINELNRQIQRLRAEVEHAKAQR
CKLEAAVAEAEQQGEAALNDARAKLMDLEAALQQAKQDMARQVREYQELMNVKLALDIEIATYRRLLEGEESRLCEGVGPVNISVSSSRGGVVC
GTESPFTTTTSSYTRSGACFSSSSGSRVASARSSGSGCGLSLGRSRVSVGASRGACGVSVDTCPPCPVPSGGFSSGSGRSSSGRFVSVSCSSRR
AQC

>Platypus_K82
MSCRSFPSTPRCGSRSFSSCSAVLPRPGSRYVLSRGPCRAGGYRALGCFASRSLCNVGTGKPRVAAVSCLPSRGGFGYRAGGACGTGPCIAPVT
INESLLIPLELEIDPNVQRVKKDEKEQIKCLNNKFASFIDKVRFLEQQNKLLETKWSFLQEQKCCQNNLEPLFEAYISTLRRQLECVVGDHGRL
EAELSGMQEVLESYKRKYEEELSLRPCAENEFVALKKDVDCAFLVKADLETNVEALIQEIDFLRTLFEEEICLLQSQISETSVIVKMDNSRDLD
LDGVIAEVKAQYDDIASRSKAEAESWYQCRFEELRVSAGKHCDTLRSSKNEILEMNRLIQRLQQEIDSTKGQRCKLEAAVAQAEQQGEAALNDA
KCKLAGLEEALQKAKQDMACLLKEYQEVMNSKLGLDIEIATYRRLLEGEESR

>Platypus_K75
MSRQSTVTFQTGGRRKGFSAASAVTPSAGRTRISSVSVSRSGAGGGGGGLAWTGCGGGGFGSRSLYGLGGSKRVSISSGGFRGGYGGRAGGGGG
YGLGSGLGGGFGGGFGGGFPVCPPGGIQEVTINQSLLAPLNLQIDPTIQRVRKEEREQIKTLNNKFASFIDKVRFLEQQNKVLDTKWELLQQQG
TKTVRQNLEPLFDAYISDLRRQLDTVTAERGRLDSELRSMQDVVEDFKAKYEDEINKRTAAENEFVALKKDVDGAYMNKVDLEAKVNSLSDEIN
FLRVLYEAELSQMQTQISDTSVVLSMDNNRSLDLDSIISEVKAQYEDIAQRSRAEAESWYQTKYEELQVTAGRHGDDLRNTKHEISEMNRMIQR
LRSEIDSVKKQCASLQTAIADAEQRGEMALKDARAKLVDLEDALQKAKQDMARQLREYQELMNVKLALDIEIATYRKLLEGEECRLSGEGVSPV
NVSVVTSTMSSGYGSGSGYGLGSGSGHGLGGGGGSYSYTTSGGHSLGGGSGHGLGVAGFSAGSGRGFGSGGGSGSSVKFVSTTSSSRKSYKH

>Platypus_K6aL
MSCKSTVTMHSSGRRGFSAASALAPGACRTGFSSLSVSRSGAGGSGFGRVCGGGAGGAGFGSRSLYNLGGSKRISISAGGCYGGRGGVVGSGFG
SGFGGGFGGGFGSGMGGGFGGGFGGGFPVCPPGGIQEVTINQSLLAPLNLQIDPTIQRVRTEEREQIKTLNNKFASFIDKVRFLEQQNKVLDTK
WQLLQQQGTKTVNQNLEPLFESYISNLRRQLDSILSERGRLDSELRGMQDLVEDFKAKYEDEINKRTAAENEFVTLKKDVDAAYMNKVELTAKV
DSLMDEINFLRALYEMELSQMQTQISDTSVVLTMDNNRCLDLDSIIAEVKAQYEEIAQRSRAEAESWYQSKYEELQQTAGRHGDDLRNTKHEIS
EMNRMIQRLRSEIDHVKKQCANLQAAIADAEQRGELALKDARVKLEELEAALQKAKQDMARQLREYQELMNVKLALDVEIATYRKLLEGEECRL
SGEGVGPVNISVVSSTVSSGHGGGSGVCSGYGLGGGSGLSGGLSVGGSSFSSSSGRCLGGGFGSGSTGSSSVRFSTTTSSSRKSYKH

>Platypus_K5
MLRQSSVSFQSGGRKGFSAASAVTPSASRTSFSSVSVSRSGGGGGGAGFGRASGACGPGFGSRSLYNLGGSKRISISGGGYRSGFGGRAGGGGG
YGFGGGSGGGYGFGGGAGSGFALGGGAGGFGGGFGGGFPVCPPGGIQEVTVNQSLLAPLNLQIDPTIQRVRTEEREQIKTLNNKFASFIDKVRF
LEQQNKVLDTKWELLQQQGTKTVRQNLEPLFESYISNLRRQLDSILNERGRLDSELRGMQDLVEDFKAKYEDEINKRTTAENEFVMLKKDVDAA
YMNKVELEAKVDGLTDEINFLRALYEAELAQMQTSVSDTSVVLSMDNNRSLDLDSIIAEVKAQYEDIANRSRTEAESWYQTKYEELQLTAGRHG
DDLRNTKHEISEMNRMIQRLRSEIDNVKKQCANLQAAIADAEQRGELALKDARAKLEELEAALQKAKQDMARQLREYQELMNVKLALDIEIATY
RKLLEGEECRLSGEGVNPVNISVVTSSVSSGYGSGGGSGFGLGGGLGGGLGGGSGYSYSSGGGHSLGGGLSVSTGGGGFSAGSGRGLGGGFGGG
SGGSSGSSVKFVSTTTSSRKSYKS

>Platypus_K71
MSRQFNCKSSKGSFSGRSAILPGGRGASSGSYRAGSKATAAAGVVGGFGSRSLYSLGGTRSISLNVAGGGGGTRSSGYGFGAGAGGCGPGRVRA
SGFAGSMFGSVALGPACPSSCPPGGIHQVTVNQSLLQPLDVKVDPEIQKVRTQEREQIKVLNNKFASFIDKVRFLEQQNQVLETKWDLLQQLDL
SNCKNNLEPIFEGYISNLRRQVEGLNGDRVRLDSELRSVRDLVEDFKKRYEEEINRRTAAENEFVVLKKDVDAAYTNKVELQAKVDSLDGEIKF
LKCLHEAEIAQIQSHISDTSVILSMDNNRDLDLDSIIDEVRVQYEDIALKSKAEAEALYQTKFLELQLVAGRHGDDLKHTKNEISELSRLIQRI
RSEIENAKKQCANLETAIADAEQRGDCALKDARAKLDDLEGALHSAKEELARLLRDYQELMSVKLALDVEIATYRKLLEGEECRMSGDFPSSVS
ISVISSTSGPSGSTGYGYRPSAVSGGYAVSSGYAVSGGYAVSGGGSSSSGARAGEARRGSASDFKDALAKGSSLVSPTRKSPR

>Platypus_K72
MSRQLSCRPNAERGDLSSLSAVLARKGGSSSGSYRAGPKVAGAAGVVGGFGSRSLYSLGGTRSISLNVAGGCARSSGYGFGAGAGGYGPGRVRA
SGFAGSMFGSVALGPACPSSCPPGGIHQVTVNQSLLQPLDVKVDPEIQKVRTQEREQIKVLNNKFASFIDKVRFLEQQNQVLETKWELLQQLDL
NNCKKNLEPIFEGYIGNLRRQLEGLSGDRVRLDSELRSVRDLVEDFKKRYELEINRRTEAENQFVLLKKDADAAYTVKVELQAKVDSLDKEIKF
LKCLYDAEIAQIQTHVSETSVILSMDNNRDLDLDSIIEEVRSQYEDIALKSKSEAEALYQSKIQELQLAAGRHGDDLKHTKNEISELNRLIQRI
RCEVTNVKKQCANLETAIADAEQRGDCALKDARAKLDDLEGALHSAKEELARLLRDYQELMSVKLALDVEIATYRKLLEGEECRMSGENPSSVS
ISVISSGGGGYGYRPSAVSGGYSASLAGSPAGGARGGDAKVKVPNTSEPKDVLVKTASGGGSPAKKTSR

>Platypus_K73
MSRQLNCRPAGERLGFSGCSAVISSRVSSSTASFRAGVKGSCAGFGSRSLFSLGGSRRISISAAGGVSRSGSLALGAGAGTGLVGGRGAGFVGT
VFGSAGLGPLCPSVCPPGGIPQVTVNKSLLAPLHVQVDPEIQRVRTQEREQIKVLNNKFASFIDKVRFLEQQNQVLETKWDLLQQLDLSNCKNN
LEPIFEGYISNLRRQVECLNGDRVRLDSELRSMRDLVEDFKKRYENEINRRTAAENEFVVLKKDVDAAYTNKVELQAKVDSLDGEIKFLKCLYE
GEIAQIQSHISDTSVILSMDNNRDLDLDSIIDEVRLQYEDIAHKSKAEAEALYQNKIQELQVAAGRHGDDLKHTKGEILELNRLIQRIRSEIGN
VKKQCANLEMAIADAEQRGDCALKDARAKLDELEGALNSAKEELARLLRDYQELMSVKLALDIEIATYRKLLEGEECRMSGEYPNSVSISVISS
TSGGGGAGAFSVGFGSTSGYSYKAGATDVKTKAGCSSEAKESKAPGAVSLPKKNPR

>Platypus_K74
MSRQFTYKSGVPGKGGFSGCSAVLPGGRGASSGSYRAGPKAAGAAGVVGGFGSRSLYSLGGTRSISLNVAGGGGARNSGYGFGAGAGGYGPGRV
RASGFAGSMFGSVALGPACPSSCPPGGIHQVTVNQSLLQPLDVKVDPEIQKVRTQEREQIKVLNNKFASFIDKVRFLEQQNQVLETKWDLLQQL
DLSNCKNNLEPIFEGYISNLRRQVEGLNGDRVRLDSELRSMRDLVEDFKKRYEEEINKRTAAENEFVVLKKDVDAAYTNKVELQAKVDSLDGEI
KFLKCLYEGEIAQIQSHISDTSVILSMDNNRDLDLDSIIDEVRVQYEDIALKSKAEAEALYQTKFQELQLAAGRHGDDLKHTKNEISELSRLIQ
RIRLEIENAKKQCANLETAIADAEQRGDCALKDARAKMDELEGALHSAKEELARLLRDYQELMSVKLALDIEIATYRKLLEGEECRMSGEYSNS
VSISVISSSTATSAGGFGLSSASAYGYRPGSAGGGFGGCVTGGGSCTPRGDVRTSKGSAGEFKDTLTKAPALSSPTKKTTR

>Platypus_K2L2
MSRQLGWKSRSGGFTSGSAVLTGGGRRTSSSSFSVCRPGGSRRSAGSGTFTSKSLVSLGGTKSISTSVAGGLGALDVGFLGGGLGVSGLSAGGL
LGAGRGPGGFGPACPPGGIQAVTVNQSLLQPLDLKVDPEIGNVKTQEREQIKVLNNKFASFIDKVRFLEQQNQVLETKWELLQQVNTTTQTSDL
SALFEAYISSLKQQVDVLNAQRTQQDSELRTIQDRVEEYKKKFEDEINSRTNAENDFVLLKKDVDAAFMVKVDLNTKVEGLQREIEFLKVFYEA
ELSEVQRNVSDTSVVLSMDNNRSLDLDSILQEVKAQYEEIAQRSKAEAEALYQYKYQELQVSVGKHGDDLKVVKTEISELNRTIQRLRAEIEHV
KKQCASVQEAILEAEQRGERTVKDAQAKLVELEEALQMAREDLARMLRDYQELLSVKLSLDVEIATYRTLLEGEECRLSGDFSSNVSYSVVKSV
SSTQGLSQSSLGSGGRGGPYVPGGLLVSKSNSSTSRNTVSSSVSGSDSVSISGVQDSSSGRSLRRSSEVGTRDSPASLVK

>Platypus_K2L1
MSRQLSCSSRGGGGGRGFSSGSVLVSGGSRRVASTSCMMRRGGGGGGGGGFCSGGAGGGGGFGSRSLVSLGGSKSISMSVAGSGGGGFGRGGGG
GGFGGGGGGFGGGGGGFGGGGGGFGVGGGGFGGLGGGGFGGLGGFGRPGGPVCPPGGIQEVTINQSLLQPLDVKVDPEIGNVRTQEREQLKTLN
NQFASFIDKVQFLEQQNKVLETKWELLQQVNTTKRTNNLEPIFEAYISSLRQHKDSLSGEKTRLESELSSLQELVEDFKKKYEDEINKRANAEN
EFVTIKKDVDSSFMVKVDLQAKMDDLTREIEFLRVLYDMELSQMHQSVSDTNVVLSMDNNRSLDLESIISEVKAQYEEIAKRSKMEAETLYQSK
YQELQVTAGSHGDRLKQTKMEISELNRMIQRLQGEIAHVKKQCKSISDAVAEAEQHGERALQDAAQKMAGLEAALQKAKEDLARLMKDYQDLMN
VKLALDVEIATYRKLLEGEECRMSGDYPSQVTVSVVSSSVSMTSGVGSGSGGGGFGSGGGFSSGGGSGGGYGSGGGSGSGGGFGSGGGGRVGSS
GGVGGSSSGTKVSVSRPGH

>Platypus_K1
MIRQFSSKSSYQSGGGGGGFSSGSAGVGGSRRVTSSSSSVRRSTGGRYGGGGGFCRGGAGGGGGGFGSRSLVSLGGSKSISMSVAAGGGHFGGF
GGGGFGGGGFGGGGFGGGGFGGGGFGGGGFGGRGGFGGPGGGFGPVCPPGGIQEVQINQSLLQPLNVEIDPEIQKVKTQEREQIKTLNNKFATF
IDKVRFLEQQNKVLETKWELLQQVNTTTRTNNLDPIFEAYISSLRHQIDTIKSDRSRLDSELKNMQDLVEDYRAKYEDEINKRTAAENEFVTIK
KDVDAAYMTKVDLQAKVDSLVQEINFYKVLFDAELSQMQTHISDTNVILSMDNNRSLDLDSIISEVKAQYEEIAQRSKAEAEALYQHKYQELQV
TAGRHGDSLKNTKTEISELNRMIQRIRAEIENVKKQIASLQQAIADAEQRGEAALKDAQGKLQELEEVLQKTKEELARLLKDYQELMNVKLALD
VEIATYRKLLEGEECRMSGDFQDNVSISVVNSTVSMSGGGGGGFGRGGGGGGYGSGGGGGYGSGGGGYGSGGGGYGSSGGGYGSGGGGYGSGGG
GYGSGGGGSGGGLGGGRGSGSGGGGGFGGGSGGGSKTSGGGGGGSSSVKFSSSTSSTKRVIR

>Platypus_K77
MSRQYSSKSACGGGGRRVFSARSTMAFRTGGRTGVSAGRVGGRCGSGGGGFGGYGSRSLYNLGGTKSISVSVAGAGYGFRGGSCGGGGFGGRGF
GSGSLGGGGGFGGFGPGGFGPVCPPGGIQEVQINQSLLQPLNLEIDPEIQKVKTQEREQIKVLNNKFASFIDKVRFLEQQNKVLETKWELLQQV
NTTTRTNNLDSLFEAYIASLRRQLDCLNSERMRQDSELRSMQDVVEDFKSKYEEEINRRTNAENDFVILKKDVDAAYMSKVELQANVESLMREL
EFLKCLYESELSQMQTHISDTNVILSMDNNRSLDLDSIIAEVKAQYEEIAQRSKAEAEALYQHKYQELQVTAGRHGDDLKSSKMEISELHRVIQ
RLQAEITNVKKQIGHVQTSISDAEERGERALQDAQQKLEELETAQQKAKEELARLLRDYQELMSVKLSLDVEIATYRKLLEGEECRMSGELQSP
VSVSVQSSHTTISGGGGGSYGGGYGSSSVRGSGFGSGGGGSYRGGSSGGGGSSYGGGSSVRGGSGISRSSGGVKSGGNSRVQIIQTSTNTSSKR
IFE

>Platypus_K76
MSRQATLATASKSGSSGGASRTFSGRSAVVTTSSTRTSGSRAGGAAVGSGMSACAGYGGMGGGFGSRSLYSLGGNKKISISVAGGSVAGGGFGG
GRSGGRGMGGAAGGFGGGFGGGFGGGFGGGAGGFGGGAGGFGGGFGGPGGSGGLPASIQEVTVNQSLLQPMHVEVDPEFSVVRKQEKEQIKTLN
NKFATFIDKVRFLEQQNKVLETKWELLQQQGGQSKTGTNNLEPFFQTYISSLQSHLSGLLSEKGRLEGELTSMEELVEDFKKKYEDEINKRTAA
ENEFVVLKKDVDAAYMTKVELEAKVESLSDEVNFLKALYDAEMSQVHSETSDTSVVLSMDNNRFLDLDSIIAEVKAQYEEIAQRSKAEAEALYK
TKLGELESTAGRHGDDLKSTKSEISELNRMIQRLRAEIESVKKQNTNLQTAIADAEQRGELALKDANAKLVELKAALQQAKDDLARLLRDYQEL
MSVKLSLDIEIATYRTLLEGEEYRMSGECQNEVSIAMVSSHSSSSSAAAGGAGGAGGRIRGGIAGGVSSSVSGGGSRVSSSTVSSSVSGGSSRA
VGGGLSSGAVTSVGGSRAVGGTSSSSRVAPASGLTQRATTKF

>Platypus_K3L2

MAQQATKASGGASRTFSGRSAVVATSSTRTTAARSSSATRSSGSGISSGMSACAGYGGMGGGFGSRSLYSLGGNKKISISVAGGSMSGGGSRAG
GFGGGRGGGRGLGGGFGGGFGGGAGGFGGGAGGFGGGAGGFGGPGGFGGPGGFGGPGGFGGPGGPGGHPAGIQEVTVNQSLLQPLKVEIDPQFS
MVKTQEKEQIKTLNNKFVTFIDKVRFLEQQNKVLETKWELLQQQGGHSKTSTNNLEPFFQTYISSLQSHLSGLLGDKDRLNDELKSMQDMVEDF
KKKYEDEVNKRTTAENDFVVLKKDVDTAYMTKVELEAKTESLTDEISFLKALYDAEMSHTQQSVSDTSVVLSMDNNRSLNLDSIIAEVKAQYEE
IAQRSKAEAEALYQHKLGELESTAGRHGDDLKNTKSEISELNRMIQRLRAEIENVKKQNTNLQTAIADAEQRGELALKDANAKLVELKAALQQA
KEDLARLMKDYQELMNVKLALDVEIATYRKLLEGEECRMSGECQSTCSIAMVSSASSSSSASAGVRGGSSVVSGGRVAGSSTVVSGGRVGGSST
VVSGGRVGGGGSVGVSTGVSSSVSTGVSSGSRVGQSVVVGGVAPVGPARVPAVGVAPVASRVPAVPTAVPVVPSVPAPRIVQTTTTSQRSTTQF

>Platypus_K3L1
MNRQVCRTTVSSGGGKGFSGRSAVVSGSSRMSSCMARSGGGGGACGSRSGGAGGFGSRSLYSLGGSKSISISVGARGGGFGGGRGVCGGGFGGG
GFGGVGGGFGGGFGGAGGFGGAGGFGGAGGFGGAGGFGGGGFGGPGGFGGPGGFGGPGGFGPGGFPGGIQEVTVNQSLLQPLNVEIDPQIGAVK
SQEREQIKTLNNKFATFIDKVRFLEQQNKVLETKWELLQQHGTGSISGTNNLEPLFETYISSLHRHLDGLLSERSRLDGELRSMQELVEDFKRK
YEEEINKRTTAENEFVTLKKDVDAAYMNKVELQSRVTSLTDEINFLRALYEMELSQMQSHVSDTSVVLSMDNNRFLNLDSIIAEVKAQYEEIAQ
RSKAEAEALYQTKLGELQSTAGRHGDDLKSTKSEIMELNRMIQRLRAEIESVKKQNANLQTAIADAEQRGELALKDARVKLEELQAALQQAKED
LARLMKDYQELMNVKLALDVEIATYRKLLEGEECRMSGEHQNAVSISVVSSNTSTSSAVGGGFGRGGGFGSGGGFGSGGGFGSGGGFGSGGGFG
SGGGFGSSGGGGFSSGSRCGLGGGFSSGSSRGGVSGSSVRFSQSSSSQRCPR

>Platypus_K4
MTSRQQSVRSSSRGFSCGSAAVTCGRRSGFSAASSGAGCRGSGAGAFGSRSLFNLGGNKSISISVAGARQNAGGFGAGGFGAGGFGGGIGGFGV
GGFGSGAFGGQIGGKGSGGFPVCPAGGIQAVTINQSLLAPLNLEIDPEIQKVRTEEREQIKTLNNKFASFIDKVRFLEQQNKVLETKWNLLQQQ
GTTTTSKHLEPFFEAYISTLRKQLDNLLSDKGRLQSELKTMQDTVEDFKSKYEEEINKRTTAENDFVVLKKDVDAAYMIKVELEAKVDALNDEI
NFLRVLYEAELAQIQTTVADTSVILSMDNNRCLDLDSIIAEVKAQYEEIAQRSKAEAEALYQTKFQELQSSAGKHGDDLKSTKMEISELNRLIQ
RLRAEIENVKKQCETLQTSVADAEQRGEAALKDARTKLTELEVALQKAKEELARLLRDYQELMSVKLALDIEIATYRKLLEGEECRMSGEFQSA
VSISVVNGGSSSSGGHGGSLGSFGGLCSGGNFSGGNFSGGSGFGGVCSSKVVSSKNWRTSSR

>Platypus_K79
MKATFSRQVYSGHGGRRGGGGSQRVFSSSSASGGGGGGCRTTFSSVTVARGSGGGGFGSRSLYNLGGGKSISVSVAGGDRARGFGVGGGGLACG
MGGFGGTYGPPRGAFPACPPGGIQAVTINQSLLQPLNVEIDPEIQRVRTQEREQIKTLNNKFASFIDKVRFLEQQNKVLETKWELLQQQGQGTG
VTKSNLDPIFESYQSSLRRQLDCLQSERGRLDSELKSMQDLLDEYKNKYEDEINKRTAAENEFVVLKKDVDAAYMGRVDLQAKVGTLTEEINFL
RCLYEAELSQVQTHVSDTNVVLSMDNNRCLDLDGIIAEVKAQYEEIARRSRAEAESWYQSQYEELQVTAGKHGDHLRDTKNEISELTRTIQRLQ
SEIDTAKKQCQQLQTAIADAEQRGDMAIKDAQAKWADLEAALHNSKEELARLLKDYQELMNVKLALDVEIATYRKLLECEESRMSGECPSAISI
SVSGSSSTVSGGACRVGFGGSVSSGVKGGYGSGYSSGYGSVGGGGGGYGVAKGGSGPGSTSVIKKSMTTTVKSSSRRY

>Platypus_K78
MLPLYVCTPPPQHPQTYIYTNTQLQAGTQVRRRGGARDLLLAKPEFAACSFSRRSVLCTVPGTWYEAELLKRGTAENDFVALKKKADLAYVNKA
ELEAQATTLKDEINFVQELYKEELAQVQKRIGDTAVILSMDNSRCLDPDGIIADVKAQYEAIVRQSREEAESWYKMRYNYLKKDLTLQGDSIQE
TEAQISELTCVIQRLQQEIEVVKKLCTNLQVATAEVEQCGELALKDARAKLTDLEAALGAAKRDLAQLLRDYQEMMNVKMGLDIEVATYQTLLE
GEECRWVLGAEGGGPRNWRDGIS

>Platypus_K8
MSVKVTQKTTVQRTSSGPLNFSSRSYGGGAVYGGGQRYLGRPAGLGGYGAVGFEAVSINQSLLAPLNLQIDPTFQRVRKEEREQIKNLNDKFAS
FIDKVRFLEQKNKMLETKWSLLQGQKSSRSNMAAMFESYISSLRRQLESLDQDKLRLEGELENMQALVEDLRMKYEDEINKRTEKENEFVLLKK
DVDAAYMNKVELESRLEGLTDEINFLRQLYEEELRELQAQISETSVVLSMDNNRHLDLDGIIAEVKAQYEEIAQRSKAEAESMYQIKYEELQVR
AGRHGDDLNRTKSEISEIHRSISRLQGEIDAIKAQNQGLKAAIADAEQRGELALKDAQAKLEKLETALHEAKQERARQLREYQELLSIKLALDV
EIATYRKLLEGEENRLEAGMQKMNIHAKAVVGYRGGLNAAYKIMNPGVGFNGRDFLQPIIPPAPHNAKAVIMVKKIESRDGKIVSESSDVL

>Kiwi_K80
MTNPCKAFTSGSLSSWEVSGKMSRGKAAGTSPDRQGCSSPTGVASNSYDSLSLHRGGRYRRSSYPSFSIDGRLLIPLHLDIDPDFQVMRQQEKE
DIKLLNNQFVSLIGKVQCLEQQNKILTTRWNFLKDQDNSRSESDIKAIYDQYMTKMNQEMKALSYERDNLELELTKVLETMDEFRNKYEDEIRL
CSGMEYTFTELKKDLDVSSLHRTELEVKLHGLQDLMDLKKTIYEQELEELLTEIKDISVVLGIDNTCKLDLSKIVEEVRAQYEALALRSWEEAE
ALTRRKLNEGNTQSATYGGHLLDSRREIADMNIQIQKLRSCIVSQKSQCLYLEENIKEAGEHGDTALKDAKAKLVRLEEALQKSKENMAHLVKE
YQELMNIKLALDVEILTYRKLMEGEESSMESPIPTVISTIHSRPKHPFSSALRNSKVFARGTGSTNSETNPKGGSTKVLPSRSYSNSSTMPEAD
VYISGARPKCNSLSTKECS

>Kiwi_K7
MSRSVSFSSHSAAIPGISKVRVSTVSSTRGGGGAGFGRPGGFGSSSLYNLGSASKRTSFGRGGSSYSTVSGYGYGGGFGMGVSPAGIQEVTINQ
NLLTPLNLEIDPNIQRVRKEEKEQIKTLNNRFASFIDKVRSLEQHNKMLETKWSLLQDQKTARSNIAPMFEAYINNLRRQLDGLLNDKGRLEGE
LKNMQDLVEDFKNKYEDEINRRTAAENEFVVLKKDVDAAYMNKVELEAKVDALTDEINFLRSLYEAELHELQAQISDTSVVLSMDNNRNLDLDS
IIAEVKAQYEEIANRSRAEAESWYQSKYEALQVTAGKHGDDLRNTKNEILEINRMIQRLQGEIENAKAQRAKLEAAIAEAEERGELAIKDAKAK
LEKLEAALQKAKQDMAKQLREYQELMNVKLALDIEIATYRKLLEGEESRLAGDGIGNVNISVVSSSGGGGYSSSSGFLGGGIGGGIGGGLSLGG
VMGSGALGFSSGGGSQSYTVTSTSSTRRSIRK

>Kiwi_K84L
MSCRSYHVHSGAGGLRSFGSCSAVIPRNMKRCTISSVSCWGGSGAGYRGLGCFSSRSVGSNAACKPRMAISSCHPLQYGYGYGYGTSGIGLGYGGA
GFGYRVGGASGAGTPIAQVTINEHLLRPLKLEMDPNLQSVKYQEKEQIKTLNNKFASFIDKVRFLEQQNKVLETKWRFLQDQKHYRSNIEPMFE
TYISNLKKQLDVLGSDKAKLETELSNMQDILEDYKKKYEEETHQRMSAENEFVMLKKDVDCAYMNQAELEAKVESLIPEINFLRSLYETEMAQL
QASISDTSVIVQMDNSRGLNMDDIIADVKAQYEDIAKRSRADAESWYYSKYEELRETAGKHDDSLRNTKNEIMELNRMIQRLNGELENTKAQRC
KLEGATAEAEEHGQVAIKDAKCKLSELEAALQKAKQDMACQLREYQELMNVKMALDIEIATYRKLLEGEESRLYIDGGCPVNISVCRSQGGITC
DADPCVGGSYSSSSRTLFRSGGLATGGGVCSKSIVTGSGDACAPCTSAGGYSAASGNSSNVKFTSTTTSYRVKY

>Kiwi_K5L4
MGGPGFPALPAGGIHEVSVNQSLLKPLNLEIDPNIQSIRKDEKEQIQTLNNKFASFIDKSEKSTSPFQVRFLEQENKVLETKWALLQEQGNKTV
KNNIEPLFETYINNLRRQLNSLLADKENLAGELNKMQSLAEDFKNKYEDEINKRAAVENEFVILKKEVDAAYMNKTELQARLDSLMEEIHFLRA
LYEAELSQMQSQISDTSVILTMDNNRSLDMDSIIAEVKAQYEDIANRSRAEAESWYQSRYEELQATAGRHGDDLRNTKQEISELNRHVQRLRSE
IDSVKKQCASLQTAIADAEQRGEMALKDARAKLAELEAALQQAKADLARQLREYQELMNVKLALDIEIATYRKLLEGEECRLSGEGASAVNISV
TRTGYGSGNCLTLGGSSGFGVGSGVCAGGIGFSSGSGQGMAGSCVGGGSSSSVKYVSTTSSTKRCY

>Kiwi_K5L3

MHKRALLSPCRAQVHLFSLLLLAPGQHLCSFILAVASIPNQKPAMSRQSTVRIQRGRSGFSTASAVVPNTCRTSFSSRSVTRVGGCNAGSGFAR
VGGGFGSRSLYNVGGCKRISVAGRGGGFYGSAGFGGGAGSVYGCGGGFGMPVNTGYGYGAFGGGMGGPGFPAGGIHEVSVNQSLLKPLNLEIDP
SVQRIRKEEKEQIKTLNNKFASFIDKVRFLEQQNKVLETKWSLLQEQGMKTVRNNIEPLFETYINNLRMQLNSLLSDKGRLEGELVNTQYLVED
FKKKYEDEINRRTVAENEFVTLKKDVDATYMNKVELQAKADAMSEEINFLRALYEAELSQMQTQISDTSVILTMDNNRNLDLDSIISEVKAQYE
DIANRSRAEAESWYQTKYEELQATAGRHGDDLRNTKQEISELNRHVQRLRSEIDNVKKQCANLKAAIADAEERGELALKDARAKLAELEDALQQ
AKADLARQLREYQELMNVKLALDIEIATYRKLLEGEECRLAGDGVPVNISVTRTTIGTGYGGGSNLSMGGGVCSMGNSFSCGSGPSVSNTAFGA
GSSSSMNFVSSSSTRRSYRS

>Kiwi_K5L2
MSRISFRSSTGGGMRGFSSGSAIVGGSSGARSSFSSVSVSRVGGGRAGGGGGFGAGGGFGSRSLYNLGGSKRISYSSVGGGLRSGAGGGYSFGG
GAGYGVCYGGGAGAGFGLGGAGGGGGYGLGSGFGVGGPGFGGRGXXXXXXXXXXXXXGGPGFVCPPGGIHEVTVNQSLLAPLKLDIDPEIQKVR
THEREQIKTLNNKFASFIDKVRFLEQQNKVLETKWTLLQEQGHTVTRKSLEPLFEAYINNLRRQLDSLMGERGRLDSELRNMQDLVEDFKNKYE
DEINRRTGAENEFVVLKKDVDGAFMNKVELQAKADSLADEINFLRALYEAELSQMQQQVSDTSVVLSMDNNRNLDLNSIIAEVKAQYEDIANRS
RAEAEAWYQNKYEELQVSAGRHGDDLRNTKIEISEINRMIQRLRNEIDSVKKQCANLQAAIAEAEERGEMALKDAKAKLTELEDALQKAKADLA
RQLREYQELMNVKLALDIEIATYRKLLEGEESRLAGEGVGAVSVSVVSTSSGMGYGSSGMGGGLGMGGGLGMGGGGGGGSSGYSMSSSSGFGMG
GGSGALGGGXXXXXXXSFSSGSGRGISSSTGSSMRIVSKTTTSKKTIR

>Kiwi_K5L1
MARSGGGGGGGFGRLIGGGGGGGGSCAGFGSKSLYNLGGTKRISIGVGSSFRAAFGGGAGGSSGLGSGGAGGGLGLGLGSGGGGGGLGGFSGGLGG
GRSLGGFGVGPPGVGTIQEVTVNQSLLAPLNLEIDPNIQQVRKDEKEQIKTLNNKFASFIDKVRFLEQQNKVLETKWTLLQEQGQKNNTGKTNM
DPLFEAYINNLKRQLASLLNERGHMDGELKNMQDLVEDFKNKYEEEINQRTAAENEFVVLKKDVDAAYMIKVELEAKVDALTDELNFLRALYDA
ELAQLSAQVSDTAVILSMDNNRDLDLSSIIAEVKAQYEDIANRSRAEAEAWYQTKFEELQATAGKHGDDLRNTKGEISELSRLIQRIRSEIENV
RSQCATLQTAIGDSEERGELALKDARAKMIELEDALQKAKADMARQLHEYQELMNIKLALDIEIATYRKLLEGEESRLSGEGLNPIGYSVISSS
SGMAGGGGGGFGGLSLSGGALEVEDLVSEVVVVVSEVDMEEEVVLAWEEAAVLASQVVMATEEEEECATLQTAIGDSEERGELALKDARAKMIEL
EDALQKAKADMARQLHEYQELMNIKLALDIEIATYRKLLSGEGLNPIGYFSVLEEAVVVEATALEVEDLVSEVVVVVSEVDMEEEVVLAWEEAA
VLASQVVMATEEEEEVAAG

>Kiwi_HBS1
MSRQCYTSSSLLGRRGFSSASAVCGLGRGNSSSASVCQPVGRRCGIGGFSSRSVCDLGRGQRISFGGNCRSGVYGGAGVGRCGVAYGGGRFGVG
TVIGFGNCGSYGGLGNYRGLGDGVAIGGYGGGIGIGLAGGRSEGIRGVSIHPELLKPLCVGVDPEECQVRTHEKEQIKNLNNQFACFIDKVRLL
EQQNKVLTTKWELLQQYVLPASRRNLEPVFENFICNLRKQLECVLGERERLENEERCLRDLVQEYKCKYEDEINKRTAAENEFVVLKKDVDCLY
LTKEELEVRVGLLRQQLEFLKCIYAEERAQLDCQLCDTSVIVQMDNSRDLDMEGIIKSVECCYEEIAQKSKAEVEAFYQTRLEELHSSRGKFCD
DLRNNQSEIAELNRMIQKLQCESDNVKKQIAALQTAICDAEQRGDCALKDARQKLIDLQTALQQAKDKMACLLRDYQELLNVKLALDIEIATYR
TLLEGEESRICTGNPVSVAVVSGGGTVGECRTLSGIGGKCTVKTGGVSSGLGVVSSFGVGGTGFSARSVDCLPRVGGGFGARSAVSCVGREVIS
GADAVQCSPSVANLGSVVCAGVEQCNPGGVIIPGAGVCATGNRYNTAMRVVRTTR

>Kiwi_HBS2
MSRQAPTGRSVLGRRGFSSASEICGRSYAASACQPVRCGAGAYSSRSACNLGGNRRISYVNGVCGTGCYGEFGYGGVGYGSVGGRVGLCGPRGY
SIVRGYPDRKADGIQGICIDERLLKPLCVGVDPLEHEIRCQEKEQIKTLNTQFACFIDKVRFLEQQNKVLETKWGLLQQHVLPKKGKNLELYFE
NYICDLRKHLDCLLCEKQKLGSEECATSQLVEEFKCKYEEEINRRTTVENEFVALKKDADCIFLNKEELEVKVDLLRRQLELLKCVFEEERSQV
DRQLCDTSVIVKMDNNRDLDMESIIKNVECWYQEIAQKSKEEVDAFYQTRFQELQDKRGKYCDDLQSNKCEISELTRMIQKLQCELENVKKQVT
CLQTSICDAEQRGDCALKDAREKHIEMQNALQKAKDELACMLRDYQELLNVKLALDIEIATYKTLLEGEESRWVTESLFLFPWMSELQA

>Kiwi_HBS3
MVGLRGRSCKSEGLCRGHINKNLLQPLCAKIDPEIQHIRMQEREQMKSLNNQFACFIDKVQHLEQENRVLATKRDLLQKQVMPSQKNLKHVFDS
FICSLQRQLDLLLHERGQMEPERNDMEKLVEVFKCKYEQEVTRHTAAKNEFILLKKDVDCVYLTKAELEAKLETLRQETEFLKCMSAQEIAELE
KSLCDTSVIVKMDNSRGLAMEGILKSIECRYEDIAQKSKAELNVLYRTRYQELEEAKGRLYNELKSHQQEIEELSFVIQRWQRDLENMKKQVSS
LQTSVCDTEQRGDCALKDAREKHIEMQNALQKAKDELAYMLRDYQELLNVKLALDIEITTYKTLLEGEERRGYSIVRGYPDRKADGIQGICIDE
RLLKPLCVGVDPLEHEIRCQEKEQIKTLNTQFACFIDKKNFP

>Kiwi_K78LT
MNRQFSRFGDGDFSSFSAHCGTLGSGRSSSALSRCDNNRGEERYGGYGYGYGSRSLHNLGGFWNVSLCGGYRGEGRGYGRCLGFGGRRHLGTGF
GRRECPVGAFQGGQAGPGIKEVHVNTSLLRPVHVQIDPEFQRVRSDEKEQIKTLNNKFASFIDKVQCLEQQNQALMTKWELLQQQSAQPEESRS
IASFFKSYISSLQRQLETLQNQKEQLDPEAYNMLRFVEDYKKRFEDEINKRMCKEEEFVELKKELDSAYMGKMEFDVRVEILKQELEFLRCLYE
AELSQLQTVVGNTDVILSMDNHRELNMDGIIEEVRREYEGIAQKSKAEVDDMYQGRYQDLQDMWVNYREQLRNSYQEIQELTRQIQRLRPEIEI
IRKKNASLQEAIRDADQCGNSAIKDGHEKLQELEHALQQAKDELARLLHDYQELLNVKLALDVEIAMYRSLLEEEETRYVQTIHPG

>Kiwi_K78L5
MAVDMDVELVALVEAMEEELAALEEVSLIQQVKCQEKEEIKNLNNQFASFIDKKKQPCLGSLPVEALEAGVEGATALALPSVVALEEDVDCAYM
TKVELEAKVGALTDEINFLRAMYEEELAQMQTISRDLSVVVSMDNNRYLDLDSIIEEVRRQYEQIAQSSRAEAEAWYQSQYEELQSTAGRHGDN
LRNTKIEIQELTRNVQRLRTEIESVKKQNQQLQAAIAEAEERGEMALKDARKKLEELECALQKDKEELARLLKEYQELLNIKIALDVEIAMYRK
LLEGEESRLCGDGISNVNVSAVGRTTIAGGRAGGFGAGSGMGGGVCVVGGGSITGGSCGVGGGILGGGFSSGTGGFGAGSGMGGGVCVVGGGSI
TGGSCGVGGGILGGGFSSGSGRMCSSGGGSFGPGGGSSSVRRCVTTTTVKSSGVKY

>Kiwi_K78L4
MSRQSVCRSFGGGSRRGYSSCSAIGGGFGGGGSRSRTSYSSYSTSRGFGGGGRSGGFSSRSLHNIGSSGRIAMGGCYGGGGYGSRMSFGGGFGG
MGGGGGMGGYGGGMGGYGGGMGGFGGGGMGGGGGMGGFGGGGMGGFGGPGFGGPGFGGPGRGGPGIQPVQVXXXXXXXXXXPVHVEIDPQIQQV
KNQEKEQIKTLNNQFASFIDKVRFLEQQNKVLSTKWELLQQQGPSGPRKNLDVIFENYIQNLRRQLDSILAQRGQLESELQSMQQYVEDYKNKY
EEEINRRTSAENEFVVLKKDVDCAYMTKVELEAKVGALTDEINFLRAIYEEELSQMQTISRDLSVVVSMDNNRHLDLDSIIEEVRRQYEQIAQS
SRAEAEAWYQSQYEQLQSTAGRHGDNLRNTKIEIQELTRNVQRLRTEIENVKKQNQQLQAAIAEAEERGEMALKDARVKLEELECALQKDKEEL
ARLLKEYQELLNIKIALDVEIAMYRKLLEGEESRLYGEGMSNVNVSVVGRTTVSGGRGGMGGGFGGSSGMGGGFGGGSSMGGGFGGSSGMGGGF
GGGSSMGGGFGGGSSMGGGVCGIGGGYGGGSFGGSSGIGGGMHSGGFSSGSGRMCSSGGGGNFGSSGGSSSVRRCVTTTSVKSSGVRF

>Kiwi_K78L3
MSRQSACRSFGGGSRRGYSSCSAIGGGFGGGGSRSRTSYSSYSTSRGFGGGGRSGGFSSRSLHNTGSSGRMAMGGCYGGGGYGSRTGLGGGFGG
MGGMGGFGGGMGGFGGGMGGGGMGGFGGPGFPGGIHPVQVDSALLRPVHVEIDPQIQQVKNQEKEQIKTLNNQFASFIDKVRFLEQQNKVLSTK
WELLQQQGPSGPRKNLDVIFEDYIQNLRRKLESLVGQRGELESELQSMQQYVEDYKTKYEEEINRRTAAENEFVVLKKDVDCAYMTKVELEAKV
GALTEEINFLRAMYEEELSQMQTISRDLSVVVSMDNNRHLDLDSIIEEVRRQYEQIAQSSRAEAEAWYQSQYEQLQSTAGRHGDNLRNTKIEIQ

ELTRNVQRLRTEIENVKKQNQQLQAAIAEAEERGEMALKDARVKLEELECALQKDKEELARLLKEYQELLNIKIALDVEIAMYRKLLEGEESRL
YGEGMSNVNVSVVGRTTVSGGFGGGSSMGGGVCGIGGGYGGGSFGGSSGIGGGMHSGGFSSGSGRMCSSGGGGNFGSSGGSSSVRRCVTTTSVK
SSGVRF

>Kiwi_K78L2
MSRQSACRSFGGGSRRGYSSCSAIGGGFGGGGSRSRTSYSSYSTSRGFGGGGRSGGFSSRSLHNTGSSGRMAMGGCYGGGGYGSRTGLGGGFGG
MGGMGGFGGGMGGFGGGMGGGGMGGFGGPGFPGGIHPVQVDSALLRPVHVEIDPQIQQVKNQEKEQIKTLNNQFASFIDKVRFLEQQNKVLSTK
WELLQQQGPSGPRKNLDVIFEDYIQNLRRKLESLVGQRGELESELQSMQQYVEDYKTKYEEEINRRTAAENEFVVLKKDVDCAYMTKVELEAKV
GALTEEINFLRAMYEEELSQMQTISRDLSVVVSMDNNRHLDLDSIIEEVRRQYEQIAQSSRAEAEAWYQSQYEQLQSTAGRHGDNLRNTKIEIQ
ELTRNVQRLRTEIENVKKQNQQLQAAIAEAEERGEMALKDARVKLEELECALQKDKEELARLLKEYQELLNIKIALDVEIAMYRKLLEGEESRL
YGEGMSNVNVSVVGRTTVSGGFGGGSSMGGGVCGIGGGYGGGSFGGSSGIGGGMHSGGFSSGSGRMCSSGGGGNFGSSGGSSSVRRCVTTTSVK
SSGVRF

>Kiwi_K78L1
MKTLNNKFASFIDKVRLLEQQNKVLETKWKLLQEQGHGTGPNSKNLDQFFEAYVNGLRKQLDGLSNEKHQLEAELKNFQDLVEDFKSKYEEEIN
RRTAAENDFVLLKKDVDGVYMNKVELQANVDALADEINFLKCLYEAELSQMQQQVSDTSVVLSMDNNRKLDLNSIIAEVKAQYEEIAKRSQMEA
ECWYQCKYEELQATAGKHGDSLKDTKTEISELNRMIQRIRAEIENVKKQCETLQASIVDAEERGEVALKDAKAKLTELETALQKAKADLTRQLR
EYQELMTVKLALDIEIATYRKLLEGEECRMSGECQSAVSICK

>Kiwi_K8
MSVTVTRKTMRSTPIVSGRSFSSHSYTTNPSVRMSTASVFGAYGGSRYGAGLYRGPAAVPSMGGSITAVSVNQSLLTPLNLEIDPNIQRVRKEE
KEQIKTYEEEINHRTEKENEGWGVRRRCGAELGPHVRRAARAQPCPVPPQELRELQSQISDTSVVLSMDNNRSLDLDSIIAEVKAQYEEIASRS
RAEAESMYQIKYEELKTTAGKHGDDLRNTRTEINELNRMIQRIQAEIEALKNQRASLETAIAEAEERGEMALKDAKGKLAELEAALQKAKADMA
RQLREYQELMNVKLALDIEIATYRKLLEGEESRLESGMQNLSIQTKTTGYSGGYGGGFSSGFRSGFDSGYGGSFSGGFGSSFSSTSYSGPPSAL
ESSAATKSRAIVIKKIETRDGKLVSESSDVLPN

>Ostrich_K80
MTNPCKAFTSGSLCSWEVSGKMSGRKAASTSSDCQGRSSPTGVASNSCDSLSIHRDGRNHHFSYPSFSIDGRLLVPLHLDIDPDFQARRQQEKE
DIKLLNNQFVSLIGKVQCLEQQNKILTTRWNFLKDQDNSHSESDIKAIYDQYMSKMNQEMKALNYERENLELELTKVLDTMDNFRSKYEDEIRL
CSGMEYTFTELKKDLDASSLHRTELEVKLHGLQDLMDLKKTIYEQELEELLTEIKDISVVLGIDNTCKLDLSKIVEEVRAQYEALALRSWEEAE
SLTRRKLNEGNTQSATYGGHLLDSRREIADLNIQIQKLRSCIVSQKSQCLYLEENIKEAGEHGEMALKDAKAKLVKLEEALQKSKENMAHLVKE
YQALMSIKLALDVEILTYRKLMEGEESSMESPIPTVISTIHSNPKQPSSSALRNSKVFARGTGSTNSETNPKGGSTKMLPSRSHSNSSAAPEAD
AYISEAQPKYNSPSIKECS

>Ostrich_K7
MSRSVSFSSRSVAVPGVSQMRVSTVSSTRGGGGASFGRAGGFGRPGLYTRGSASNRISLGRGGSSYSVHSGYGYGGGLGMGFGPAGIQEVNINQ
SLLTPLNLEIDPNIQRVRKEEKEQIKTLNNRFASFIDKVRFLEQQNKMLETKWSLLQDQKTSRSNIAPMFEAYINNLRHQLDGLLNDKGRLEGE
LKNMQDLVEDFKNKYEDEINKRTAAENEFVVLKKDVDAAYMNKVELEAKVDALTDEINFLRSLYEAELRELQAQISDTSVVLSMDNSRNLDLDS
IIAEVKAQYEEIANRSRAEAESWYQSKYEALQVTAGKHGDDLRNTKNEISEINRMIQRLQSEIENAKAQRAKLEAAISEAEERGELAIKDAKAK
LEELEAALQKAKQDMARQLREYQELMNVKLALDIEIATYRKLLEGEESRLAGDGFGNVNISVVSSSGGGGYSSSSGFLGGGIGGGLSLGAGMGS
GALGFSSGGGSKSYTLTTTSSTRRSIRK

>Ostrich_K5L4
MSRQCAVRNQSKIGFSAASAYIPNTCSSTSFCSRSVSQGGSCGTTAGFGRYGGGFGSRSLYSLGGCKRISVAGRGGGFYGPAGFGAGSGIACGF
GGAAGGAFRFGGGMAGPGFPALPAGGIHEVSVNQNLLKPLNLEIDPNIQSIRKDEKEQIQTLNNKFASFIDKVRFLEQENKVLETKWALLQEQG
NKTVINNIEPLFETYINNLRRQLNNLLADKENLAGELNKVQSLAEDFKNKYEEEINKRAAVENEFVILKKEVDAAYMTKTELQARLDSLVEEID
FLRALYEAELSQMQTQISDTSVILTMDNNRSLDMDSIIAEVKAQYEDIANRSRAEAESWYQSRYEELQATAGRHGDDLRNTKQEISELNRHVQR
LRSEIDGVKKQCVSLQTAIADAEQRGELALKDARAKLAELEDALQKAKADLARQLREYQELMNVKLALDIEIATYRKLLEGEECRLSGEGASAV
NISVTRTGYGSGNCLTLGGSSGLGVGGGVCAGGTGFSSGSGQGMARSCVGGGSSSSVKYVSTTSSTKRCY

>Ostrich_K5L3
MSRQSTVRIQRGRSGFSTASAVVPNTCRTSFSSRSVSQVGGCNAGSGFARVGGGFGSRSLYSVGGCKRISVAGRGGGFYGPAGFGGGAGSVYGC
GGGFGMPVNTGYGYSAFGGGMGGPGFPAGGIHEVSVNQSLLKPLNLEIDPSIQRIRKEEKEQIKTLNNKFASFIDKVRFLEQQNKVLETKWSLL
QEQGMKTVRSNIEPLFETYINNLRMQLNSLLSDKGRLEGELVNTQYLVEDFKKKYEDEINRRTVAENEFVTLKKDVDAAYMNKVELQAKADALS
EEINFLRALYEAELSQMQTQISDTSVILTMDNNRNLDLDSIISEVKAQYEDIANRSRAEAESWYQTKYEELQATAGRHGDDLRNTKQEISELNR
HVQRLRSEIDGVKKQCANLKTAIADAEERGELALKDAKAKLAELEDALQKAKADLARQLREYQELMNVKLALDIEIATYRKLLEGEECRLAGDG
VPVNISVTRTTIGTGYGGGSNVGMGGGVCSMGNSFSCGNGPGVSSTTLGAGSSSSVKFVSTSSTRRSYRS

>Ostrich_K5L2
MSRISFRSSTGGGMRGFSSGSAIVGGSGGARSSFSSVSVSRVGGGRAGGGGGFGAGGGFGSRSLYNLGGSKRISYSSIGGGLRSGAGAGYGFGG
GAGFGVGYGGGAGAGFGLGGAGGGGGYGLGSGFGLGGPGFGGRGGPGFPVCPPGGIHEVTVNQSLLAPLKLDIDPEIQKVRTQEREQIKTLNNK
FASFIDKVRFLEQQNKVLETKWTLLQEQGQTVTRKSLEPLFEAYINNLRRQLDSLMGERGRLDSELRNMQDMVEDFKNKYEDEINRRTGAENEF
VVLKKDVDGAFMNKVELQAKADSLADEINFLRALYEAELSQMQQQVSDTSVVLSMDNNRNLDLNSIIAEVKAQYEDIANRSRAEAEAWYQNKYE
ELQVSAGRHGDDLRNTKIEISEINRMIQRLRNEIESVKKQCANLQAAIAEAEERGELALKDAKAKLAELEDALQKAKADLARQLREYQELMNVK
LALDIEIATYRKLLEGEESRLAGEGVGAVSVSVVSTSSGMAYGSGSSLGMGGGLCVGGGGGSGGGYSLSSGGGGSENGGLSYGGGSSFSSGSGR
GISSSTGGSVRIVSKTTTSKKTIR

>Ostrich_K5L1
MSMARSGGGGGAFGRLSGGGGGGGGGSAAFGSRSLYNLGGSKRISIGVGSSFRAAFGGGAGGSSGLGSGGGGSYGFGSGGAGGGLGLGLGLGGG
GGGGGGGGYGFGGSAGGLGFGGGHGGGGGFGGVGGLGGIQEVTINQSLLAPLNLEIDPNIQQVRKDEKEQIKTLNNKFASFIDKVRFLEQQNKV
LETKWTLLQEQGQKNNTGKNNLDPLFEAYINNLKRQLASLLNERGRMDGELKNMQDLVEDFKNKYEEEINRRTAAENEFVVLKKDVDAAYMTKV
ELEAKVDALTDELNFLRALYDAELAQLSAQVSDTAVILSMDNNRDLDLSSIIAEVKSQYEDIANRSRAEAEAWYQNKFEELQATAGKHGEDLRN
TKGEISELSRLIQRIRSEIENVRNQCATLQTAIGDSEERGELALKDAKAKMMELEDALQKAKADMARQLREYQELMNIKLALDIEIATYRKLLE
GEESRLSGEGLNPISYSVISSSSGMAGGGGGFGGLSLSGGGGGSGLGLGGGGGSGFGLGGGAGGGNYSFGSGGGLGLGGGSGLGGGYGGGSALG
LGGGSSLGIASGHGYGGGGGGSGLSTGGGNFSSGSAKGTNPGVKIVSKTSSSKKSIKSQSLKNAVQPE

>Ostrich_HBS1

GNSSSASVCQPVGRRCGIGGFSSRSVCDLGRGQRISFGGSCRSGVYGGAGVGRGVAYGGGRFGVGTVIGFGNCGSYGSLGSYRGLGDGVTIGGY
SSGVGLAGGRSEGIRGVSIHPELLKPLCVGVDPEECQVRTHEKEQIKNLNNQFACFIDKVRLLEQQNKVLTTKWELLQQYVLPASRRNLEPVFE
NFICNLRKQLECVLGERERLENEERCLRDLVQEYKCKYEDEINKRTAAENEFVVLKKDVDCLYLTKEELEVRVGLLRQQLEFLKCIYAEERAQL
DCQLCDTSVIVQMDNSRDLDMEGIIKSVECCYEEIAQKSKAEVEAFYQTRLEELHSSRGKFCDDLRNNQSEIAELNRMIQKLQCESDNVKKQIA
ALQTAICDAEQRGDCALKDARQKLIDLQTALQQAKDKMACLLRDYQELLNVKLALDIEIATYRTLLEGEESRICTGNPVSVAVVSGGGTVGECR
TLSGIGGKCAVKTGGASSGLGAVSSFGVGGTGFSARSVDCLPR

>Ostrich_HBS2
MVCLHGRSCKSEGLHRVHISENLLQPLYVKIDPEIQHIQMQEREQMNSLNNQFACFIDKVRFLEQQNKVLETKWGFLQQYVLPKKGKNLELYFE
NYICDLRKRLDCLLCEKQKLGSEECATSQLVEEFKCKYEEEINRRTTAENEFVALKKDADCIFLNKGELEVKVDLLRRQLELLKCVFEEERAQV
DRQLCDTSVIVKMDNNRDLDMESIIKNVECWYQEIAQKSKEEVDAFYQTRFQELQDKRGKYCDDLQSNKCEISELTRMIQKLQCELETVKKQVT
SLQTSICDVEQCGDCALKDAREKHIELQNALQKAKDELACMLRDYQELLNVKLALDIEIATYKTLLEGEESR

>Ostrich_K78LT
MQEVTTNQNFLAPLSLDIKPEIWKVQKLKKEQIKTLNNKCTSFVDSRYEEEISSCLAAEDGSVVLKDILPWIPSQVHVNTSLLRPVHVQIDPEF
QRARSDEKEQIKTLNNKFASFIDKVQCLERQNQALMTKWELLQQQCTQPEESRSIASLFRSYISNLQRQLETLQNQKEQLDPEAYNMLRLVEDY
KKRFEDEINKHMCKEEEFVELKKELDSTYMGKTEFDVRVEILKQELEFLRCLHEAELSQLQTVVGNIDVILSTDNHRELNMDGIIEEIRREYDG
IARRSKAEVDAMYQGRYQDLQNMWVNHREQLRNTYQEIQELTRHIQRLRPEIEIVRKKNTSLQEAIKDADQRGNSAIKDGQEKLQDLEHALQQA
KDELARLLHDYQELLNVKLALDVEIAMCRSLLEEEETRIQQGSPTRISVAAALEGGGHPLEVEEEAQVVVVLALEEEEATGLRVEAMGLLEEEA
LVVGAPALAVEDPPLEGEAMGLLEEEALEEEEGDMGLEVAGAVLTVGV

>Ostrich_K78L
MSRQSICRSFGGGSKRGYSSCSAIGGGFGGSGGRSRISYSSFSTSRGTGGGGHCGGFSSRSLHNMGGSGRISLGGSYGGGYGCRIGGFGGSYGG
GICSFGGGVIGGGIGSFGGSVRGGPGFPGGIHPVQVDPSLLRPVHVEIDPQIQQVKCQEKEQIKTLNNQFASFIDKVRFLEQQNKVLSTKWELL
QQQGPTGPRKNLDVLFENYIQNLRRKLESLVGQRGQLESELQSMRQYVEDYKTKYEEEINRRTAAENEFVVLKKDVDCAYMTKVELEAKVGALT
DEINFLRCIYEEELSQMQTISRDLSVVVSMDNSRHLDLDSIIEEVRRQYEQIAQNSRAEAEAWYQSRYEELQSTAGRHGDSLRNTKIEIQELTR
NVQRLRTEIENVKKQNQQLQAAIAEAEERGEMALKDARRKLEELESALQKDKEELARLLKEYQELLNIKIALDVEIAMYRKLLEGEENRLCGDN
LSNVNVSVVGRTTIAGGRAGGFGAGSGMGGGVCAVGGGSCGVGGGILSGGFSSGGGGFASSSGSSSVRRCVTTTTVKSSGVKY

>Zebra_finch_K80
MTNPSSAFSSGSLSSGEAAARMSPDGGASDGHGSVGSDGHGSVGSDGHGSVGSDGHGSPGSHGHGSPGSHGHGSPGSHGHGSPGSHGHGSPGSH
GHGSPGSHGHGSPGILQSSPGSDGHGSPGSHGHGSPGILRGSPGILRGSPRSPLPGQGKLPPAVRLDIDTEFQAVRQQEKEDIKVLNNQFVTLI
EKVQSLEQQNKILTTRWNFLKDQDNSHSDSDIKAIYEQYVSKMNQEMKALNYEQENLESELTKVLSTMDTFRSKYEDEIRLCSGMEFTFMELKK
DLDVSTLHRTELEVKLKGLQELLELKKTIYEQELEELLTEIKDISVVLGIDSCSRPDLGRIVEDVRAQYEALALRSWEEAEALTRRKLTDSSSQ
SGSFGGHLLDSRREIADLNIRIQKLRSCIVSQKSQCLYLEEHIREAGEQGEGTLRDAKAKVAGLEEALQRSREHMAQLVREYQELMNIKLALDV
EILTYRKLVEGEENSMEQPIPTVISAVHSRPKPLSASTLRNSRLFAQGSGHVEPSRGGPGRAQDVPGTAATRTQPKFSSGAAGECS

>Zebra_finch_K7
MSRSVTFSSRSAAGPALSQLRVSSVSSGRSSALGRGFGSASLYSLGSAGRRASPATGSSFGARSGYGFGGSLSPGGIQEVTVNQSLLTPLNLEI
DPSIQRVRKEEKEQIKTLNNKFASFIDKVRFLEQQNKMLETKWSLLQEQKTTRSNIAPMFETYISNLRRQLDGLLADKGRLEGELKNMQDLVED
FKTKYEDEINKRTAAENEFVVLKKDVDAAYMNKVELEAKVDALTDEINFLRAFHEAELNELQAQISDTSVVLSMDNSRNLDLDSIIAEVKAQYE
EIANRSRAEAESWYQSKYEALQVTAGKHGDDLRNTKNEITEINRVIQRIQGEIENAKAQRAKLEAAVAEAEERGEMALKDARAKLEELEAALQK
AKADMARQLREYQELMNVKLALDIEIATYRKLLEGEESRLAGDGVGNVNISVVSSSGGGGYSSSSGFLGAGIGGGLSLGSGMGSGALGFSSGGS
TKSYTITTTSSSRRSVRK

>Zebra_finch_K5L4
MSRQCTVRSLGRTSFSAASACIPNASSSSLCLRPVPQAGSCGATTGYGRFIGGFGSRSLHSLGECQRISVAGRGGVSYGPAGFGTGTGISYGFG
GAVAGPFGFGGGPGFPAVPVGGIHEVSVNQSLLKPLNLEFHPKIKSIRKDEKDQIQTLNNKFASFIDKVRFLEQQNKVLETKWALLQEQGNKTV
RKNIEPLFDTYLNNLRRHLSSLMTDKENLAGELSKMHSIAEDFKNKYEEEVNKRADVENEFVTLKKEVDTAYLDKTKLQARLDSLTEEIDFLRA
LYEAELSQIQSQISDTSVILTMDNNRSLNMEGIIAEVKAQYEEIANRSRAEAESWYQSRYEELQATAGRHGDDLRNTKQEISELNRHIQRLRSE
IDSVKKQVTALQTAITDAEHRGELALKDARTKLEDLETALQKAKADLARQLREYQELMNVKLALDIEIVTYRKLLEGEECRLSGEGAGSVNISV
TRTTSGMGYGSRSCLSPSSGAGGEVCAGMGMGYISGSGYGTGGSCVLGGSSSSFKSISTSSSMRGCY

>Zebra_finch_K5L3
MSRQSTVRIQRGRSGFSAASAMVPNTCRTSFSSCSVTRLGSCNAGSGFARVGGGFGSKSLYNVGGCKKISVAGRGGSFYGSAGFGGGTGSVYGG
GFGAPANLGYGYGAFGGGPGFPAGGIHEVSINQSLLKPLNLEIDPNIQRIRKEEKEQIKTLNNKFASFIDKVRFLEQQNKVLETKWSLLQEQGM
KTVRNNLEPLFETYINNLRVQLNSLLSDKGRLEGELVNTQYLVEDFKKKYEDEINRRTIAENEFVTLKKDVDASYMNKVELQARADALSEEINF
LRALYEAELSQMQTQISDTSVVLTMDNNRNLDLDSIISEVKAQYEDIANRSRAEAESWYQTKYEELQATAGRHGDDLRNTKQEISELNRHVQRL
RSEIDSVKKQCANLKAAIADAEERGELTLKDAKAKLAELEDALQQAKADLARQLREYQELMNVKLALDIEIATYRKLLEGEECRLAGDGVPVNI
SVTRTTVGTGYGGGSNLSMGGGICNLGNSFNCGSVPGVGSTTLGAGSSSSMKFVSSSSTRRSYRS

>Zebra_finch_K5L2
MSRISFRSSTGGGMRGFSSGSAIVGGGGGGTRSSFSSVSVSRVGGGRAGGGGGFGAGGGFGSRSLYNLGGSKRISYSSVSGGLRSGAGGGYSFG
LGYGGGAGAGFGLGGAGGGGYGVGSGFGLGGPGFGGRGGPGFPVCPPGGIHEVTVNQSLLAPLKLDIDPEIQKVRTQEREQIKTLNNKFASFID
KVRFLEQQNKVLETKWTLLQEQGHTVTRKSLEPIFEAYINNLRRQLDSLMGERGRLDSELRSMQDMVEDFKNKYEDEINRRTGAENEFVVLKKD
VDGAYMNKVELQGKADALADEINFLRALYEAELSQMQQQVSDTSVVLSMDNNRNLDLNSIIAEVKAQYEDIANRSRAEAEAWYQNKYEELQVSA
GRHGDDLRNTKIEISEINRMVQRLRNEIDSVKKQCANLQAAIAEAEERGEMALKDAKAKLTELEDALQKAKADLARQLREYQELMNVKLALDIE
IATYRKLLEGEESRLAGEGVGAVSVSVVSSSSGMGYGGGSCLGLGGGLGMGGGGGSSSYTMSSSGFGGGSGGFSGGLNYGGGSSFSSGSSRGIS
SSTGGSVRIVSKTTTSKKTIR

>Zebra_finch_K5L1
MNRTLSMRAGGGGRSYSAASAIIPSSNRAGFSSVSVARSGGGGGGFGRLIGGGGGSSSGGGGGFGSRSLYNLGGSKRISIGVGSSFRAAFGSGA
AGGSGLGGGGGVGGWGLGSGGAGGGLGLGLGAGGGGFGFGGGAGGLGYGGGYGGSGGFGPGGLGGLGGFPVGLGGGRNPVGFSGGPSGVGTIQE
VTVNQSLLAPLNLEIDPNIQQVRKDEKEQIKTLNNKFASFIDKVRFLEQQNKVLETKWTLLQEQGQKNNSGKSNLDPLFEAYINNLRRQLANLL
NERGRMDGELKNMQDLVEDFKNKYEEEINRRTAAENEFVVLKKDVDAAYMNKVELEAKVDALSDELNFLRALYEAELAQLSAQVSDTAVILSMD
NNRDLDLSSIIAEVKAQYEDIANRSRAEAEAWYQTKFEELQATAGKHGDDLRNTKGEISELNRLIQRIRSEIENTRNQCATLQTAIGDSEERGE
LALKDAKAKMIDLEDALQKAKADMARQLREYQELMNVKLALDIEIATYRKLLEGEESRLSGEGLNPISYSVISSGSSIAGGAGLGGGFGGLSLS

GGGGGSGFGLGGGIGNGFGLGGGGGSSFGLGAGGGGGGNYSFGSTGGLGLGAGGGLGGGFGGGSGLGTAGSYSHAGGGSSTLSTSGGNFSSGSA
KGTNPGVKIVSKTSSSKKSIKSQSLKSATEPE

>Zebra_finch_HBS1
MSRQCYTSGSILGRRGFSSASAVCGLGRPNSSSASVCQPVGRRCGIGGFSSRSVCDLGRGQRISFGGSCRSGVYGGAGVGRCGVAYGGGRFGTV
VGFGNCASFGGLGSYRGLGDGVAMGGYGGGIGIGLSGGRSEGIRGVSIHPELLKPLCVGVDPEECQVRTHEKEQIKNLNNQFACFIDKVRLLEQ
QNKVLTTKWELLQQYVLPASRRNLEPVFENFICNLRKQLECVMGERERLENEERCLRDLVQEFKCKYEDEINKRTAAENEFVVLKKDVDCLYLT
KEELEVRVGLLRQQLEFLKCIYAEERAQLDCQLCDTSVIVQMDNSRDLDMEGIIKSVECCYEEIAQKSKAEVEAFYQTRLEELHSSRGKFCDDL
RNNQSEIAELNRMIQKLQCESDNVKKQISALQTAICDAEQRGDCALKDARQKLVDLQTALQQAKDKMACLLRDYQELLNVKLALDIEIATYRTL
LEGEESRICTGNPVSVAVVSGGGTVGECRSLSGIGGKCTVKSGGGGAGLGMVSSFGNAGFSTRSVDCVPKVGAGFGARSAVSCVGREVLTGVDG
VQCAAGMGNLGNLGNLGNLGNLGNLGNLGSVVCAGVEQCGPGPVLIPGAPGVCGNRFSTAVRVVRTSR

>Zebra_finch_HBS2
MSRQAPTVRSVLGRRGFSSASEICGRSYAASASQPVRCGAYSSRSVCNLGGSRRISYVNGGCSTGCFGEMGFGGMGNGNAGRGRIGLCGLRGYS
IVRGYPERKADGIQGICIDERLLKPLCVGVDPLEHEIRCQEKEQIKTLNTQFACFIDKVRFLEQQNKVLETKWGLLQQYVLPKKGKNLELYFEN
YICDLRKRLDCLLCEKQKLGSEECATSQLVEEFKCKYEEEINRRTTVENEFVALKKDADCIFLNKEELEVKVDLLRRQLELLKCVFEEERAQVD
RQLCDTSVIVKMDNNRDLDMESIIKNVECWYQEIAQKSKEEVDAFYQTRFQELQDKRGKFCHDLQSNKCEISELTRMIQKLQCELESVKKQVSC
LQTSICDVEQRGDCALKDAREKHVELQNALQKAKDELACMLRDYQELLNVKLALDIEIATYKTLLEGEESRICVGNPVSVSVVSSGYNIPDDCG
MMANGAVCGYGSLGRRAGRHSSQTGGFSSRSAGIHPKRVLSSVAKQCIPEVFCQAGGVSCKAGGFSSRSGGYPARTVVSAGSGGLNARMGPCQA
GQVLSFGNQGCVIRQLAGPPVVVSSSPEVVGCNNGVVGNFGVVRDPCVVP

>Zebra_finch_K78LT
MRRYFSGYGDGSFTSSSAHCGTLGGGDGRFRGFGYGYGSRSLHNLGGFRNVYTGGGYRGEGVRYGSCLGFGGWRQPERGFGGRGYFVGGFQSGT
TGLGGTYRESSGREVLRGGLGIGEQGAGQGLGQAGVLRGIEEVHVNTNLLRPIQLQVDPEFQRARSDEREQIKALNNKFASFIEKVQCLERQNQ
ALLAKWELLQQQSSGPKESRNINSFFQSYITNLQRQLETLQSQKEQLDPEAYNMLQLVEDYKNRFEDEINKRTSKEEEFVELKKELDSAYMGKM
EFEVRVDILRQELEFLRCLYEAELSQLQTVVGNVDVILSMDNQRDLNMDGIIEEVRREYEGMAHRSTAEVDAMYRGRYQDLQNMWVSQREQLRN
SHQEIQELARQIQRLQPEIEITRKRNSSLQDAVKDAEHRGSSAVRDGQEKLEELENALQQAKDDLSQLVHDYQELLNVKLGLDIEIAMYRSLLE
EEENRIQEGSPATICVIDYNARASGVFGGIQSVQGRMSSGGTSGSGMKGPVSGGGKGGSSGGGGSCSGGGGSISGGGKGSRYGGGGSSGSGGSI
CGGGGSSGSGGSMSKGGSSSGGWGSSSGGGGSMSGGGKGSGYGGGGSSGSGGSICGGGGSSGSGGSMSKGGSSSGGWGSSSGGGGSMSGGGKGS
GYGGGGSSGSGGSICGGGGSSGSGGSMSKGGSSSGGWGSSSGGGGSMSGGGKGSGYGGGGSSGSGGSICGGGGSSGSGGSMSKGGSSSGGWGSS
SGGGGSMSGGGKGSGYGGGGSSGSGGSICGGGGSSGSGGSMSKGGSSSGGWGSSSGGGGSMSGGGKGSGYGGGGSSGSGGSICGGGGSSGSGGS
MSKGGSSSGGWGSSSGGGGSMSGGGKGSGYGGGGSSGSGGSICGGGGSSGSGGSMSKGGSSSGGWGSSSGGGGSMSGGGKGSGYGGGGSSGSGG
SMSKGGSSSGGWGSSSGGGGSMSGGGKGSGSGGGVSSSGSGGSISKGGSSSGGGGSSCGGGKGSGSGGQGSSGSGGSMSGGGQSSGGYGSGSGR
CSPQSGGMSSGGGSSCGRRGSMSGGGGQGSSGGGGSMSGGGQSSGGHGSGSGSCSSHSGGMSSGGGSSCGRRGSMSGGGGQGSSGSGGSMSGGG
QSSGGYGSGSGSCSSQSGGMSSGGGSSCGRRGSMSGSGGQGSSGGGGSMSGGGQSSGGHGSGSGSCSSHGGGMSSGGGSSCGRRGSMSGCGGQG
SSGGGGSMSGGSQSSGGHGSGSGSCSSHSGGMSSGGGSSCGRRGSMSGSGGQGSSGGGGSMSGGSQSSGGHGSGSGSCSSHSGGMSSGGGSSCG
RRGSMSGSGGQGSSGGGGSMSGGGQSSGGHGSGSGSCSSHGGGMSSGGGSSCGRRGSMSGGGGQGSSGGGGSMSGGSQSSGGHGSGSGSCSSHS
GGMSSGGGSSCGRRGSMSGSGGQGSSGGGGSMSGGSQSSGGHGSGSGSCSSHSGGMSSGGGSSCGRRGSMSGSGGQGSSGGGGSMSGGGQSSGG
HGSGSGSCSSQSGGMSSGGGSSCGRRGSMSGGGQGSSGSGGSMSGGGQSSGGHGSGSGSCSSHSGGMSSGGGSSCGRRGSMSGGGGQGSSGSGG
SMSGGGQSSGGHGSGSGSCSSHSGGMSSGGGSSCGRRGSMSGGGGQGSSGSGGSMSGGSQSSGGHGSGSGSCSSHSGGMSSGGGSSCGRRGSMS
GGGQGSSGSGGSMSGGGQSSGGYGSGSGRCSSHSGGMSSGGGSSCGRRGSMSGGGGQGSSGSGGSMSGGGQSSGGHGSGSGRCSSHSGGMSSGG
GSSCGRRGSMSGGGGQGSSGSGGSMSGGGQSSGGHGSGSGSCSSHSGGMSSGGGSSCGRRGSMSGSGGQGSSGGGGSMSGGGQSSGGHGSGSGS
CSSHSGGMSSGGGSSCGRRGSMSGGGGQGSSGSGGSMSGGGQTAGGGGSSSGSCSPHSGGMSSGGWSSSGRRGSMSGGGGSSGYGGSSYGSGGS
SYGSGGCIGGGEGGSRYGGSSSRRGSMSGGGSSSGGYGSGSGSCSPHSGGMSSGGWSSSGRRGSMSGGGHSSGGYGSGSGSGSCSPHSGGMSSG
SWSSSGRRGSMSGEGGSSGHGGSSYGSSGCTGGREGGSGYGGSSGYGGGSCTRGSMSGGGSSSGGLGSGSGSGGSSSGGGISSGYGGSSGSGGS
SSGCIGGGDGGSGYGGSSSRRGSMSGGGSSSGGYGSGSGSCSPHSGGMSSGGWSSSGRRGSMSGGGGSSGYGASSYGSGRSIGGGEGGSAGYGG
GSSGRRGSISGGGEGGGSYGSGGSSYGSGGSSYGSGGCTGGGEGGSGYGGTGSSVRRGSMSGGGHSSGGLGSGSGSGGSSSGGGISSGYEGSSG
SGGSSSGCIGGGDGGSGYGGSSSRRGSISGGGHSSGGYGSGSGSCSPHSGGMSSGGWSSSGRRGSMSGGGGSSGYGGSSYGSGGSSYGSGGCIG
GGEGGSGYGGTGSSGRRGSMSGGGHSSGGYGSGSGSGSGSSHSGHMSSGSWSGSGRRGSMSGEGGSSGHGGSSYGSSGCTGGGEGGSGYGGSSG
YGGGSCTSGSMSGGGSSSGGLGSGSGSGGSSSGGGISSGYGGSSGSGGSSSGCIGGGDGGSGYGGSSSRRGSMSGGGSSSGGYGSGSGTGSCSP
HSGGMSSGSWSGSGRRGSMSGGGSSSGGYGSGSGSCSPHSGGMSSGGWSSSGRRGSMSGGGASSGGYGSGSGSCSPHSGGMSSGSWSSSGRRGS
MSGEGGSSGHGGSSYGSSGCIGGGEGGSGYGGSSSYGGGSGRRGSISGGGEGGGSYGSGGSSYGSGGCTGGGEGGSGYGGGSGYGGFSGYGGGS
AYGGGSSGRRGSISGGRRSSISGVWGSSSAGTCRPGSGGEGGWDCSGGDSCYGGGTCVDASCRDATEECRSGEAWSSPSPGSWSAYGSGSVSPG
DAAHK

>Zebra_finch_K78L4
MSRQSVCRSFGVGSKRGFSSCSAVGGGFGGGSRSRISYSSFSTSRGIGGGGGHCGGFSSRSLHNLGGSTRISMGSSYGSGYGCRIGGFGGGFPG
GFGGIGGGVIGGGMGCFGGPIRGGPGFPGGIQPVQVDPTLLRPVHVDIDPQIQQVKCQEKEQIKTLNNQFASFIDKVRFLEQQNKVLSTKWELL
QQQGPTGPRKNLDVIFENYIQNLRRRLESLLGQRGQLESELQNMRQYVEEFKTKYEEEINRRTAAENEFVVLKKDVDCAYMTKVELEAKVGALT
DEINFLRCIYEEELSQMQTISRDLSVVVSMDNNRHLDLDSIIEEVRRQYEQIAQNSRAEAEAWYQSRYEELQSTAGRHGDSLRNTKIEIQELSR
NVQRLRAEIENVKKQNQQLQAAIAEAEERGEMAVKDARRKLEELECALSKDKEELARLLKEYQELLNIKIALDVEIAMYRKLLEGEENRLCLEN
PSNVNVSVVGRTTVCGGRSGASFGASNGLGGGVCTVGGGNIISSSCGMGGGILSGGFSSGSGRMCSTAGGNFMAGGGSSSVRRCVTTTTVKSSG
VKY

>Zebra_finch_K78L3
MSRQSVCRSFGVGGRKGFSSCSAVGGGFGGGGGRSKISYSSYSSSRGGGGGGHCGGFSSRSLHSMGGSRRIAMGGCYGGGGYGGRMGGFGGGMS
CGGMGGGGMGVGGMGMGGFGGGMGGGAGMGGFGGGMSSGGMGGMGGFGGPGFPGGIQPVQVDPSLLRPVHVEIDPQIQQVKNQEKEQIKTLNNQ
FASFIDKVRFLEQQNKVLSTKWELLQQQGPTGPRKNLDVIFENYIQNLRRRLESLLGQRGQLESELQNMRQYVEEFKTKYEEEINRRTAAENEF
VVLKKDVDCAYMTKVELEAKVGALTDEINFLRCIYEEELSQMQTISRDLSVVVSMDNNRHLDLDSIIEEVRRQYEQIAQSSRAEAEAWYQSRYE
ELQNTAGKHGDSLRNTKIEIQELSRNVQRLRAEIENVKKQNQQLQAAIAEAEERGEMAVKDARRKLEELECALQKDKEELARLLKEYQELLNTK
IALDVEIAMYRKLLEGEENRLCNDSMSNVNVSVVGRTTVSGGRGGMGGGFGGSGMGGGFGGSSCGMGGGFGGGSGMGGGMGGGGMCGGGSSFGG
GSMGGSCGMGGGMYAGGFSSGSGRMCGSGGGSSSVRRCVTTTSVKSSGVRF

>Zebra_finch_K78L2
MSRQSVCRSFGGGSRRGFSSCSAIGGGFGGGSRSRSSYSSYSVSRGFGGGGRCGGFGSRSLHNMGGSARMSMGGCYGGGYGGRMGGFGGSYGGL
GSFGGGMGGGGGGMCGFGGMGGGGMGGGGGMGGFGGGMGGFGSGMGGGGMGGFGGPGFGMGGPGRGGPGIQPVQIDTTLLRPVHVEIDPQIQQV
KNQEKEQIKTLNNQFASFIDKVRFLEQQNKVLSTKWELLQQQGPTGPRKNLDVIFENYIQNLRRQLDSILAQRGQLESELQNMQQYVEDYKTKY
EEEINRRTTAENEFVVLKKDVDSAYMTKVELEARVGALTDEINFLRCIYEEELSQMQTISRDLSVVVSMDNNRHLDLDSIIEEVRRQYEQIAQS

SRAEAEAWYQSQYEQLQSTAGRHGDNLRNTKIEIQELTRNIQRLRAEIENVKKQNQQLQAAIAEAEERGEMALKDARLKLEELESALQKDKEEL
ARLLKEYQELLNVKIALDVEIAMYRKLLEGEENRLCNDTMSNVNVSVVGRTTVSGGRGGMGGGFGGSGMGGGFGGSGMGGGFGGGSGMGGGMGG
GGICGGGSSFGGGSMGGSCGMGGGMYAGGFSSGSGRMCGSGGGSSSVRRCVTTTSVKSSGVRF

>Zebra_finch_K78L1
MTSPLPPGTDQLPVPIKRGTQQLLPQDLALLASQAPASAFCSEFWGLFTMSRQSCALGRGFSSSSVSFGSKNKVMFGSVPRGGCRGSGAAGGFS
SRSLYSLGGTKSTSLGGFGGAGGACRGFGFGAGAGFGGGYGAGCFGGGPGSGFGGGFDGGMGGQGFPPCPPGGIREVTINQSLLAPLNLEIDPE
IQKVRTQEREEIKKLNDKFASFIDKVRFLEQQNRVLETKWKLLQEQGGTGTGSRNLDPFFETYISGLRKQLDCLSSEKLQLETELKSFQDMVED
FKTKYEEEINKRTAAENEFVLLKKDVDGAYMTKVELQGKLDSLSDEINFLKCLYEAELSQMQQQVSDTSVVLSMDNNRSLDLDSIIAEVKAQYE
EIANRSRLEAESWYRCKYEELQATAGKHGDSLKDTKIEISELNRVIQRIRAEIESVKKQCETIQTSIADVEQRGEVALKDARDKLAELETALQK
AKTDMARQLREYQDLMNVKLALDVEIATYRKLLEGEECRMSGECQSSVSISVVGGSSSVGSGYGSGMCLGGGGIGFGSGKGSCSMGGAGLCFSL
GGSGFGAGGGFGSLSGGSNSVVVGGSGTVLKNSGPTSRRP

>Zebra_finch_K8
MSVSIPRQPLRSSSAVPGRSFSSRSFTAGPVLGPYGGLRGGSGGLGGPSWVPGAGGGITAVTINRSLLAPLDVAVDPELQELRREEKEQIKTLN
DKFAAFIDKVRFLEQQNKLLETKWGLLRAQPPPRSALGGLLEGYAGTLRRQLEGLGQERQRLRAELSHVRGLVEEFKAKYEEEINHRTEKENEF
VLLKKDVDEAYMSKVELESRLESLTDEINFLRQLYEEELQELRAQVSDTAVVVSMDNSRQLDMAGVLAEVRAQYEDIAGRSRAEAEGLYQVKYE
ELKTAAGKQGDDLRQTRSQIQELNRRIQRIQAEIEALKNQRSGLEAAVAEAEERGELALRDARAKLAGLEGALAQAKQDLARQLREYQELMNVK
LALDIEIATYRKLLEGEESRLEAAVQNLSIHTKTSGYLGGFGGGFGGGFGGPFGSTVVSSGYSVPPPPPESTAPLKSRAIVIKKIETRDGKLVS
ESSDVLES

>Nanorana_K80L
MASFSNAFSYDVPPWQSLNGQNHPSPSPALSETYATESCTTTTTNGSHASQSFEEVFKSKLMVSTDTQTTVSQQDDAEAKLSSALMSFRDKILY
LEKQRELLQERWSIVQVEDTSEIDLEPIYLSYISRLLAQVNSVNKQNNQTQSSVLDMMDSINAIKDKYEDEFNTRTDMEYAFVDLKKDVDACSM
DKTELEVKQRHLHDFIELLTTIYEHELKEVMEESGDISVLLNMDSGCPLNFQNIVKEIKERYESIAAKSREEAQALSKKKLQQGVLQAGRYETE
LENSRSQISQLNSQIPRLRSEVLSVQTQCMNLEQKVSVAKMKSSAALKDANAKLAEVQEALQKAKQDVARLIREYQDLMNVKLALDIEIVTYKK
LLEGEEIRLNSPTVVNVHCETEVRRPSQSRNSSSSWSVHSSKSDY

>Nanorana2_02
MSYQKGFSTRSAIPQGGVSSVRISQTRTSSGAGLGRPANWSSSSLSNVGSVRKSVSYGVSSGRSGVGGGSLYGIGGGSGFGGGFGGGYGGGQGT
GPGIHEVTINQSLLAPLNLEIDPNIQVVRKEEREQIKTLNNKFASFIDKVRFLEQQNKVLETKWDLLQNQKSSKASNIAPMFEAYIANLRRQLD
TLINDKGRLDGEFRNMQDLVEDFKNKYEDEINRRTAAENEFVVLKKDVDGAYMNKVELEAKVDALTDEINFLRALYEAELRELQAQISDTSVVL
SMDNNRALDLNSIIAEVKAQYEDIAAKSRAEAESTYQYKYQELQATAGRHGDDLRNTKTEISELNRAIQRLQAEIENVKAQRAKLEAQIAEAEG
RGELALKDASGKLEELQAALQKAKQDMARQLREYQELMNVKLALDIEIATYRKLLEGEESRLAGEGVGAVSMSVVNSSSSSGGGYGGSSFGGGS
GYGGSSLKFGSGGGSSSGISYAMTTSSSSKSSMRY

>Nanorana2_03
MTHKQYRKSGVRNFSSSSLCAPGNASGFSVSSYRRGPSFGSRSLYNAGSLGGRMSVASYHPVRGSYGYGGLGSGLRVGGAGSAFSCRVGGGYSG
GGGVGGGFGAGGGYGVGGGFGVGGGIGGYGAGFSPVGITPVTVNQNLLTPLNLEIDPNLHSLRKEEKEQIKTLNNRFASFIDKVRFLEQQNKML
ETKWTLLQEQKTVKSHIEPYFEAYLNNLRRQSEGLGGEKARLEAELKNMQDTVEDYKNKYEDEINKRTAAENEFVVLKKDVDGAFMNKADLEAK
VGMLRDEINFLQQIYQQEIDQLHAQISDTSVVVSMDNSRDLDMESIIAEVKAQYEDMAKKSRTEAESWYQTRYEQLRISVGKHGDDLRNTKNEI
AEHNRMISRIKGEIENVKSQRAKLEAAIAEAEERGEAAIRDAKNKLVELEDALQKAKKEMARLLRDYQELMNVKLALDIEIATYRKLLEGEECR
MAGDGGSVSISVVSSTTGGVIGGGYDGGSGFYHGGSGFSSGSGRGLAGSRFSSGSTMSSSGGYITTGGIHSSSTGGTVHPGGYSSGGSGSSSVN
VTKTTSSTRKI

>Nanorana2_04
MKQKDPEDLKVGLGKLSKEGETVGSRKSGGEGKRRKLPTGFRDTKEAGPGAVGGAKEPMKHGGVVRRGALLRDREAIWRQAIPITFNMTGGIKA
TWYEQFWWFKTAGPELSRSERASLGLLGARKRETAEIATKCPALTCLSLVTISASVRAFRISRLTLIDVCEAAGRNGDIATGRPPVSLRETYHV
TAALSKYFDLMSSRWPNLMNEEERSSAARPRTGGCNSSGSPCSSKMSVGSFHLGKAGHGHGHGFGHDFGGSYGSGGITPVTVNQSLLAPLNLEI
DSNIQRVRTEEKNQIKGLNNKFASFIDKVRFLEQQNKMLETKWDLLQEQKTARCQIEPLFEAFIGNLRRHLDSLECESARLEAERSSMEDMMEG
FKRKYEEEMNRRTAAENEFVLLKRDVDAAFMNKAELQAKADALTDEISFLRTLYDAEISQLQAQISDTSVIVSMDNSRDLDLDSIVAEVRAQYE
EIANRSRAEAESMYQSRFDDLRMASGRNGNDLESSRNEIAELNRAIQALKGEIVCAKSQRAALEAAINEAEERGEAAIRDAKHKLAELEAALQK
AKQDMARQLREYQELMNVKLALDIEIATYKKMLEGEEHRLTSDGNVNICK

>Nanorana2_05
MSVGSYCSGKSGHGSGFGIVGLGRGFGGQCGGITPVTVNQGLLAPLNLEIDPSIQRVRTEEKDQIKGLNNKFASFIDKVRFLEQQNKMLETKWG
LLQEQRTARCQIDPLFEAFISNLRRQLESLECEGARLAAERNSMEGTVEELKRRYEDEVNRRTAAENEFVSLKRDVDAAFMNKAELQAKADSLT
DEINFLRTLYDAEISQLQAQISDTSVVVSMDNSRDLDMGSIIAEVRAQYEEIANRSRAEAEAMYQSRFEDMRMAAGRNGNDLQNSRNEIAELNR
TIQRLRGEIECAKSQRAALESAISEAEERGEAAVRDAKNKLAELEAALQKAKQDMARQMREYQELMNVKLALDIEIATYKKMLEGEEYRLASDG
HVNLFYTPALEENTIRGDTMEDTMEDNNITIIKEDSPRSAMSPK

>Nanorana2_06
MSVGSYCSGKSGHGSGFGIVGLGRGVGGQCGGITPVTVNQGLLAPLNLEIDPSIQRVRTEEKDQIKGLNNKFASFIDKVRFLEQQNKMLETKWG
LLQEQRTARCQIEPLFEAFINNLRRQLESLECEGARLEAERNSMEGNVEELKRRYEDEVNRRTAAENEFVSLKRDVDAAFMNKAELQAKADSLT
DEINFLRTLYDAEISQLQAQISDTSVVVSMDNSRDLDMDSMIAEVRAQYEEIANRSRAEAEAMYQSRVSDLRMAAGRNGNDLQNSRNEIAELNR
TIQRLRGEIECAKSQRAALESAISEAEERGEAAVRDAKNKLAELEAALQKAKQDMARQMREYQELMNVKLALDIEIATYKKMLEGEEYRLASDG
HVNLFYTPALEENTNRGDTMEDTMEDTNITIIREDSPRPAMSPK

>Nanorana2_07
MSVGSYCSGKSGHGHGSGFGIVGLGRGVGGQCGGITPVTVNQGLLAPLNLEIDPSIQRVRTEEKDQIKGLNNKFASFIDKVRFLEQQNKMLETK
WGLLQEQRTARCQIEPLFEAFISNLRRQLESLECEGTRLAEERNSMEGTVEELKRRYEDEVNRRTAAENEFVSLKRDVDAAFMNKAELQAKANS
LTDEINFLRTLYDAEISQLQAQISDTSVVVSMDNSRDLDMGSIIAEVRAQYEEIANRSRAEAEAMYQSRFEDLRMAAGRNGNDLQNSRNEIAEL
NRTIQRLRGEIEFAKSQRAALESAISEAEERGEAAVRDAKNKLAELEAALQKAKQDMARQMREYQELMNVKLALDIEIATYKKMLEGEEYRLAS
DGHVNLFYTPALEENTIRGDNMEDTMEETMEDTNITIIREDSPRSAMSPK

>Nanorana2_08
MLGMATRYNIPSPVQVPIVLDQKDIRCQSEVIVQEKVDMDLDLVLLGLVVELEDNVEESPLLLFVQTVGNIVGLGRGVGGQCGGITPVTVNQGL
LAPLNLEIDPSIQRVRTEEKDQIKGLNNKFASFIDKVRFLEQQNKMLETKWGLLQEQRTARCQIEPLFEAFISNLRRQLESHNVWVSICPVNTF

LVHTLRFIYEDEVNRRTAAENEFVSLKRDVDAAFMNKAELQAKADSLTDEINFLRTLYDAEISQLQAQISDTSVVVSMDNSRDLDMDSMIAEVR
AQYEEIANRSRAEAEAMYQSRFEDVRMAAGRNNNDLQNSKNEIAELNRTIQRLKGEIECAKSQRAALESAISEAEERGEAAVRDAKNKLAELEA
ALQKAKQDMARQMREYQELMNVKLALDIEITTYKKMLEGEEYRLASDGHVNLFYTPGLEENTIRGDTMEDTSITIKREDSPRTLAKRSAVPTLA
IYQDLLNCCRDLNSILDLAYLRFRDSARRTFYEFGNKSGRLLPRSVRARRAAPTVSLNILSTIEEKLFNHFMSSILHPVHAGNHAQA

>Nanorana2_09
MSVGSYCSGKSGHGHGSGFGIVGLGHGFGGQCGGISPVTVDQGLLAPLNLEIDPSIQRVRTEEKDQIKGLNNKFASFIDKVRFLEQQNKMLETK
WGLLQEQRTARCQIEPLFEAFISNLRRQLESLECEGARLAAERNSMEGTVEELKRRYEDEVNRRTAAENEFVSLKRDVDAAFMNKAELQAKADS
LTDEINFLRTLYDAEISQLQAQISDTSVVVSMDNSRDLDMDSMIAEVRAQYEEIANRSRAEAEAMYQSRFEDVRMAAGRNGNDLQNSRNEIAEL
NRTIQRLRGEIECAKSQRAALESAISEAEERGEAAVRDAKNKLAELEAALQKARQDMARQMREYQELMNVKLALDIEIATYKKMLEGEEYR

>Nanorana2_10
MCGSGGITPVTINQGLLAPLSLEIDPNIQRVRTDEKNQIKGLNNKFASFIDKVRFLEQQNKMLETKWGFLQGQKTARCQIEPLFEAFIGNLRRQ
LENLECERARLDAERNSMEGTVEELRRRYEDEVNRRTAAENEFVSLKRDVDAAFMNKAELQAKADSLSDEINFLRALYDAEISQLQAQISDTSV
LVSMDNSRDLDINGILAEVKAQYEEIANRSRAEAEAMYQSRFEDLRNAAGRNGNDLQNSKNEIADLNRTIQQLKGEIECAKSQRSALEVAINEA
EERGEAAVRDAKHKLAELEAALQKAKQDMARQMREYQELMNVKLALDIEIATYKKMLEGEEHR

>Nanorana2_11
MSHHSGHGSHGHRNFTSSSACLPAVSKHSFGSIATKKVSVGHKMVPSYSSKSVYSVVSGGHKLSSGLKAGHGYSFAGIGHGLSSASYGYGGSFC
SSGITPVTVNQSLLAPLNLEIDPNIQRVRIDEKDQIKGLNNKFATFIDKVRFLEQQNKMLETKWALLQEKKPAKSHIEPLFEAYISNLRKQLEH
LGSERAHLEAERRNMEDAVEEFKKKYEEEINRRTAAENEFVGLKRDVDAAFINKAELQSKVDSLTDEINFLRALFDAEIAQLQAQISDISVIVS
MDNSRDLDMDSIIAEVKAQYEEIANRSRSEAEAMYQSRYEELRMTAGKHGDDLRNTKHEITELTRLIQRLKGEIESVKSQRAKLESAITEAEER
GEAAVKDAKHKLAELEAALQKAKQDMARQLREYQELMNVKLALDIEIATYRKLLEGEEGRMVGEGHGSVSISVVNNTTGHSSGSQHHHIGGGFS
SSSLSKGKHGHHL

>Nanorana2_12
MSFQTNHSGASYRNFSTSSLPRNVSRQSVCSSRLVSGGSRIQPHYSRSAYNVGSGRAQKISVATHNTGMSGYGGAGFGAGFGSGMGSGGRFGGS
SFGQGGFGGAGFGSVGFGGAGFCGAHGGITNVTVNESLLAPLNLEIDPNIQRMRKEEKEQIKTLNNKFASFIDKVRFLEQQNKMLETKWAFLQN
QKTVKSNIEPLFESYINNFRRQLDCLDNEKSRLEGEKKNMEDLVEEFKRKYEDELRKRTAAENEFVVLKKDVDAAFMNKTELQAKADSLNDELD
FLRTVYDMEICQLQSQISDTSVIVSMDNSRDLDMDGIISEIKAQYQEIANKSRVEAESWYQSKYEELKTTAGKHGDNLRSTKNEISDLNRGVQR
LKTEIENAKAQRAKLESAIAQAEERGEMALKDGKNKLADLEEALQKVKQDMARQLKEYQELMNTKLALDLEIAAYKKLLEGEECRLSGECAGSV
KISVVSSSMGGSISNAGGFYGDSGMSSGAGFGGASVIGRGVGGSRFISSGGVSCSSGTANRGFSSGTGYTGGTSSSQGTITRSTSSSCSRRM

>Nanorana2_13
MSASRQMTYSVGGAKKNVSSASVCPAGANRNSFSSVSVSRSSSGGVGAGHAGSGGFGSRSLYCLGGSRKISIPTGLQSQSGSGSGYGAGVGAGF
GVGSGFGIGQGFGGNSGFPVCPPGGIQQVTVNQSLLAPLNLEIDPQIQKVRTEEREQIKTLNNKFASFIDKVRFLEQQNKVLETKWKLLQEQGV
KVVKNNIEPLFEAYINSLRRQLDSLVNDKARLDGELKQMQDAVEEFKHKYEDEINKRTAAENEFVVLKKDVDIAYTNKVELESKVAALTDEINF
LRALYEAELAQMQGQISDTSVILSMDNNRNLDLNSIISEVKAQYEEIANRSRQEAESWYQNKYEELQVSAGRHGDDLKSTKAEIAELNRYINKL
RSEIDSVKKQCAKLQTAIADAEQRGELAVKDAKQKLSGLEDALQKAKQDMARQLKEYQELMNVKLALDIEIATYRKLLEGEESRLAGEGVGPVN
ISVVSSAYGSSSGVGHSYGVGGGFGFSSGSGAGMGCGYGNGVSGSGAFVSGGQGMSSGGYSFNNLKPGGFMSGGGSNSGVIVSKTVTASRKN

>Nanorana2_14
MSLAHQTMKKTFSTASAVPAGANRNSFSSYSTVRSSSGGLGGGRASTGGFGSRSLQNLGYGKRMSQSSQSRYGYGSGFGAGSGSGFAIGGGAGY
GVGSGFGAGHGFGGGQGYGGGQGFGGGQGFGGGAGFPVCPPGGIQEVTINQSLLAPLNLEIDPNIQRVRTEEREQIKTLNNKFASFIDKVRFLE
QQNKVLETKWSLLQEQGVKTVRSNIEPMFESYINTLRRQLDSLVNDKSRLDSELRQMQDCVEDFKNKYEDEINKRTAAENEFVVLKKDVDAAYM
NKVELECKVDALTDEINFLRALYEAELLQMQGQISDTQVVLSMDNNRVLDLDCIIAEVKAQYEDIANRSRKEAESWYQTKYEELQVSAGRHGDD
LKSTKTEISELNRMITRLRSEIENVKKQCAKLQASIAEAEERGELAIKDAKHKLIELEEALQKAKQDMARQLKDYQELMNVKLALDIEIATYRK
LLEGEESRLAGDGVGAVNISVVSSSYGSGYGMGGGSGYGMGGGSGYGMGGGSGIGFGGGSGYGVGGGSSGYGAGGSYGGGSFSAGSGKLAGFGS
GSGSNSSVKIISKTSTSSRRH

>Nanorana2_15
MSGQVVSNVSVSVVGGGSSAGGSGFGSGYGVGGGSGYGVGGGSGYGVGGGVGGGSGYGVGGGSGYGGGGSGCGGGAGGAGGAGGGGFGSRSLFN
VGGRKTISISTAGGQGGFGGGGIGFGVGAGGGGGFGGGSGGFAYGGQGFGSGGGQAGITEVTINQSLLAPLNLEIDPNIQKVRVQEREQIKTLN
NKFASFIDKVRFLEQQNKVLETKWSLLQEQGGQVKGGSRGKSNIEAMFEAYMNSLKRQLDALQNDKYRLDGELRNMQDLVDDFKNRYEDEINKR
TSAENDFVILKKDVDAAYMNKVELEAKVDALTDEINFLRSLYEQEMAQLQSQISDTSVILSMDNNRALDLDSIIAEVKAQYEDIAKKSRAEAES
TYQSKVKELQASAGAQGDVLKTTKNEISELNRKLQRLRAEIENVKKQNAKLQTAIAEAEDRGELVLKDAHAKLAELEAALQNAKQDMARQLREY
QELMNVKLALDVEIATYRKLLEGEEIRLTTDTNVSISVVSGKTSLGSGGGGGGAGGSYGGSGGSYGGSGGSYGGAGGSYGGGFGGSGGSAGGYG
FSSGSSAGYGFSAGGGGGSSVRFASSQSSYRS

>Nanorana2_16
MSYNTRFSSSNAASSRGFSSSSMGGGGGRTISSSSQFGGGGLRSAPSAGFGSRSLLNVGGGRKVSSISSSRDSGFGGGYGGGSGFGGGSGFGGG
YGGGSGPAPPSIVNVTVNRQLLTPLSFEIDPNVSVVKKEEREQIKTLNNKFASFIDKVRFLEQQNKVLETKWSLLQQQGGGQSGQRSNIEPLFQ
AYINNLKKQLDALGSDKGRLESELRNEQDRVEEFKRRYEEEINLRTSAENDFVVLKKDVDAAYLNKVELDAKVNALNDEIAFLRTLYDQELNEL
RQQVSDTSVILSMDNNRSLNLDDIIAEVKGQYEELANKSRAEAEDTYKNKFQQLQSVAGQQGDELKNQKNEISQLNRAIQKLKAEIENVKKQIA
ALQIAIAEAEQRGENALNDARGKLAELEAALQRLKQEMNSQLREYQELMNVKLALDVEIATYRKLLEGEESRISGEVSNTVNEEGDSLKRVEET
CKADHSDQVTSS

>Nanorana2_17
MTSRSLYHSLGGSRKIAVIPGNRTSVGGYGFGGGGGGGGFGGGAGSGYGGGMGAGGAFGGGGGYGGPGGYGGGGFPDGGPDFPVCPPGGIQQVT
INQSLLSPLNLEIDPSIQKVKTEEREQIKTLNNKFASFIDKVRFLEQQNQVLKTKWSFLQEHGQKGGTKRSTLEPLFETYINNLRRQLDSINND
RGRLESELRNMQDMVEDFKNKYEDEINKRTSAENEFVVLKKDVDAAYMNKVELECKADQLTDEINFLRTLYEAEMNEVQGQIADTSVILSMDNN
RSLDLQGIINEVKSQYDEIAQRSKYEAEAAYAMKFEQLKQTAGQHGSNLKSSKTEIAELNRAIQRLKAEIESVKKQCASLQTAISEAEERGELA
LKDAQKKLNDLQAALKKSKEDLAKQMRDYQELMNVKLSLDIEIATYRTLLEGEECRMSGEIVNPVNISVVSGSTSYNIGGGGGGGAGGSMAMGG
GGGGGYGASGGGGYGAGGGGGYGAGGGGGYGGGGGGGFGTGGGFGAGGVGYGAGGGSYGAGGGGYGGSGGYGGGSSMGAGGGGGDYSGGGSFSS
GSRQGMTFVSKTSTASSRTSY

>Nanorana2_18

MNAVTSGRTTMSHIQRSGATKPGKASAGDFCGAKGFSSCSTGGGYGKANYSSSSLIRSGGPGSYSVRLGAGRSNYGTSSLYCSGGSKRISIATG
TNGGISTVGKGLSFSGERFPLCPRGGIQNVTVNQILLQPVKADIDPNFQKVRIQEREQIKTLNNKFASFIDKVRFLEQQNKILETKWKFMQEQS
KKLANMKDNIKPLFEVYISCLQRQLDTTKNEKCRLEGDLKNMQDVVEDFKAKYEEEINKRANAENEFVSLKKDVDSLYMQKTDLDGKQDALVDE
LNFLKTLFDAEVAEMQARISNTNVILTMDNNRDLDLDGVVAEAKAQYEEIAARSRAEAESNYARQFQQLKDTLGQHGDNLHDHRNEIQELNGMI
RRLHGEIDCVKKQICALETAISDAEGRGEVALKDAKAKLSELEAALQKAKEDLAVQLRNYQELLNVKMALDLEIATYRKLLEGEECRIHGEVDN
NVTMSVYSSAGKVAIPSLSCVAGSCSVPRSSAAGAGYSSILKSSNTGASGCGTGTCHINSKVANATGGITALLSTTHTKRAY

>Nanorana2_19
MLYEEEINKRANAENEFVSLKKDVDCLYMQKTDLDGKQDALVDELSFLKTLFDAEVAEMQAQISNTNVILTMDNNRDLDLDGVVAEAKAQYEEI
AARSRAEAESNYARQFQQLKDTLGQHGDNLRDHRNEIQELNGMIKRLHGEIECVKKQICALETAISDAEGRGEVALKDAKAKLSELEAALQKAK
EDLAVQLRNYQELLNVKMALDLEIATYRKLLEGEECRIHGEVDNNVTMSVYSSAGKVAIPSLSCVAGSCSVPRSSAAGAGYSSILTSSNSGASS
CGTGTCNINSKVANATGGITSLMSSTRSKRAY

>Nanorana2_20
MSNLKQMQSGGGGSRGFSVCSLGGGRSGGSRSSSFASQSLHNAQCLGGRKRTSVASSVRVGSSYGGGAFGGSGGFGGGAGGYGGGSGGYGGGAG
GHGGGAGGHGGGAGGHGGGAGGYGGGGSGFGGGVGFGGGAGGYGGGQGGDPGFPVCPPGGIQQVSINQSLLKPLNVEIDPNIQKVKTEEREQIK
TLNNKFASFIDKVRFLEQQNKVLETKWHLLQEQGSKGGSKKNNLEPLFENFINILNRQLDSLLSEKGRLDNDLKGMQEMVEDYKKKYEDEINKR
TAAENEFVVLKKDVDAAFMVKTDLEIKVDLYTDELNFLRTLYDAVRMPSNVFKHTMSGQVVSNVSVSVVGGGSSAGGSGFGSGYGVGGGSGYGV
GGGSGFGVGFGVGGGVGGGSGYGVGGGSGYGGGGSGYGVGGGNCYVGGGQGSGNYNQSSTKSSVAVSKTVTSSVKQSY

>Nanorana2_K8
MSIKMSKVTYRTSSSAVPRSSGFSSFSYSGGPVGARASTGSFSLGSSYGGGARFGSGYRTAYGGQGAGAGSAGITHVSVNQSLLAPLNLEIDPN
IHIVRTQEKEQIMTLNNKFASFIDKVRFLEQQNKMLETKWGLLQNQKITRSNIDGMFEAYINNLRRQLDSLGQEKQRLESELGNMQGLVEDFKN
KYEDEINKRTEMENDFVLLKKDVDEAYMNKVQLEARLESLTDEINFLRHLYEEELRELQSQISDTQVVLSMDNNRNLDLDSIIAEVKTQYEEIA
NKSRAEVEGMYQLKYQELQASAGRHGDDLKNTKTEISELNRYITRLQSEIDSLKAQRANLEAQIAEAEERGELAMKDARSKLNELEAALQKAKQ
DMARQLREYQELMNVKLALDIEIATYRKLLEGEESRLESGIQNLSIQTKTTVSSGYSGGLQSSYTGGLQSSYTGGLQSGYGGGLGGSYMYASSA
NSSPLETSRTKRSIVVKTVETKDGRVLSESSDVFSK

>Axolotl_K8
TAVSVNQSLLAPLNLEIDPSIQEMRTQEKEQIKTLNNKFASFIDKVRFLEQQNKMLETKWGLLQNQKTTRSNMDSMFEAYINNLRRQLDGLGQE
KMKLENELGNMQGMVEDFKNKYEDEINKRTECENEFVLIKKDVDEAYMNKVQLETRLEGLTDEINFLRELYAAELNELQSQISDTSVVLSMDNN
RSLDLDSIIADVKAQYEEIANKSRADAENMYQIKYQELQTSAGRYGDDLRSTKNEIAEMNRGINRLQAEIDALKNQRANLDAAIVEAEERGKLA
VADARSKLAELEAALQKAKQDMARQLCEYQELMNVKLALDIEIATYRKLLEGEENRLESGMQNVNIQTKTSAYQVNSAYGGSGYGGSGYGGSGY
GTSGFGSGFSNAFSGGSYAVNASPLESSRTKRTVLVKTVETKDGKVTSESSDYFNKP

>Axolotl_K7L
VTFNQSLLTPLNLEIDPTLSDVRKEEREQIKTLNNKFASFIDKVRFLEQQNKVLETKWELLQNQKSSKTSNIPAMYEQYINNLRRQMDGLCNDK
GRLDGELRNMQDMVEDFKNKYEDEINKRTAAENEFVVLKKDVDAAFMNKVQLETSVDALSDEINFLRGIYEMELHEMQAKVTDTSVVLSMDNNR
ALDLDSIIAEVKAQYEDIANRSRTEAESHYQSKYQELQENAGKHGDDLRTTKNEISELNRMIQRLQAEIENVKNQRGKMEGSIAECEERGELAL
KDAREKLAELEAALNKAKQDLAHQARDYQELLSCKLALDIEIATYRK

>Axolotl_K5L1
VTINQSLLSPLNLEIDPTIQRVRTEEREQIKTLNNKFASFIDKVRFLEQQNKVLDTKWQLLQEQGHKTTKSNVEPMFETYINNLRRQIDALGNE
RHRLDADLRNMQDMVEDFKNKYEDEINKRTAAENDFVMLKKDVDAAFMNKEELDSKVVSLNEELNFLRAVYEEELRQMQTQISDTSVILSMDNN
RHLNLDSIIAEVKAQYEHIANTSRIEAETWYQTKYEELQSSAGRHGDDLRSTKAEISELNRMIQRLRSEIDNVKKQCEKLQHAISEAEERGELA
LKDARKKLADLEAALQKAKEDMAHQLRDYQELMNVKLALDLEIATYRK

>Axolotl_K5L4
VTINQSLLSPLNLEIDPEIQKVRTEEREQIKTLNNKFASFIDKVRFLEQQNQMLQTKWDFLQEHGQKGGSKRDNLEPMFEAYCNNMRRQVERLT
SDRSRLDNEHRNMQDLVEDYKNKYEDEINKRTAAENEFVVLKKDVDSAFMTKVELNGKVDTLTDELQFYKSLYDAELTAMQGQMSDTSVILSMD
NNRSLDLNSLIAEARAHYEDIANKSRAEAESTYASKFQQLQAAAGQHGDHLKSSKTEISELNRTIQRFKAEIESVKKQCANLQTAIAEAEERGE
LALKDARKKLAELEAALQKCKEEMARQLKEYQDLMNVKMALDLEIATYR

>Axolotl_K80L
EVRDLERQNKILEAQLSLLKDRNSYKSNANQIMQVSSNNLKQQIDNLLREKEKLETELANMEALLKDLKNRYEDEINRRNQLENDFVLTKKDLD
DTYLQKVDTESKLDSTSDEINYLRQLFDEEIRELESQVHNAEITVELDNTRELEMKQVMEEVKQQYQAMAEKGQQEAEHWYKSKLDDIANEAQR
HNEDLKNVKNEISELTRQIQRGNGEIDALKNQRTNLENAIGMAEDKGEQAMRNTKDNIQELEEALKRAKQDMAGKVREYQDLMNTKLALDIEIA
TYRKLLEGEETRLIGPAVQTQMYSAVDRLAGSLMQDLPISSMASTNVSSGFSAPANTNVSFNYSA
PVSANPAPTKKPVMIKTIETVNGQRISESS

>Axolotl_K5L2
TQVTVNPQLLVPYTPDFDHTVSDVKKEEREQIKTLNNKFAAFIDKVRFLEQQNKVLETKWDLLQQQGGKTGSTSKSNIDPLFEAYISSMRRQLD
GLNNDHGRLDGDLRKTKDQVEDFKNKYEDEINRRTAAENDFVVLKKDVDAAYMNKVELEARMDALTDEINFLRALYETELAEVQGNATDTAVIL
SMDNNRNLDLKSLIADVKSQYEAIALKSQQEAEAGYAQKYQQLQSAALQYGDGAKTTKSEISELNRQIQRLRAEIENVKKQCANLQTSAEEVED
RGEHALQDARSKLNELEAALQKAKQDLALQLRDYQELMGIKLALDMEIATYR

>Axolotl_K5L3
VTVDPSLQTPFNVEIDPEIQKVRTEEREQIKDLNNKFAAFIDKVRFLEQQNKVLETKWSLLQEQGGITTGSQRDNIEPIFEEYISNLRQQLEGL
NGDRGRLDGELRTMQDMVEEFKQKYEEEINRRTVAENEFVMLKKDVDAAYMAKVELESKVDTISDETSFSRTLFDAELSQLQAQISDTSVILSM
DNNRVLDLNSIIAEVKSQYEEIANRSRNDAELTYRFKYEELQSSADQHVDVLRNTKNEISELNRMTQKLKVEIENVKKQISKLQTAITEAEERG
ELALKDAKEKLSDLEAALQKAKEDMALQLREYQELLNVKLSLDIEIATYRK

>Caecilian_K8
MSLVTKKVQYRMSSSGPRPAGGFSSFSSSPAIGSRVSTAPFALSTAGSRYGSGYRLGYSGPAAGAGGITAVTVNPSLLAPLNLEIDPTIQHVRT
QEKDQIKTLNNKFASFIDKVRFLEQQNKMLETKWNLLQSQKVTKSNTDGMFDAYICNLRRQLDALGQEKFKLEAELSNMQGLVEDFKNKYEDEI
NKRTEMENDFVLLKKDVDEAYMNKVQLESRLEGLTDEINFLRQLYEEELRELQSQISETAVVLSMDNNRNLDLDSIIADVKAQYEDIANRSRAE
AEKMYQIKYEELQVSAGRHGDDLRNTKSQIAEMTRMITRLQSEIDALKGQRTNLEEAIKEAEEQGELALKDARGKLAELETALQRAKQDMARQL

REYQDLMNVKLALDIEIATYKKLLEGEENRLESGMQTMNVQTKTYSGGLSSAFGSGGYVSGGLSSAFGSGGYFPTNNYIVNSSPLESTKTTKRT
VVVKTVETKDGRVLSESSDVFSKP

>Caecilian_K80
MASFSESEAFSERRNLSDFTTSTPEMCNGYTAGASVNGEADSRSSTYSHSSAITHVTEHKTQLKSSLKKRSSTKRVTIQEKGTEETESLNDKLN
SFTEKLQILEQQKQMLEDRWTHLQEQEISQSNQIDMEPLYQTYISKLLKDVTKINKENNDIQSKLTGMMDSVDDFKDKYEDEFNTRIDLEYSFV
QLKKDLDSISLDNTQLETQVNEVQGTIQLMKSIYEEEMKDLKNNIKDISVVLEMDNRCTVDLEGIVKDVRERYEAIAAKSREDAEALCKAKLNE
KAAKAGKYGNDLIKSRSDITDLNMKIQKMRSEMLAMQDQSIHLENAITESKENGESATKDAEAKLAELESALQKAKQDIARQVREHQELMNIKL
SLDMEIVTYKKLLEGEESRMQVPTAAIISVQTGPSRSLQKGRASEWEGRPNPEPRRKNHSKNLLIKAIEHHTQVHSK

>Caecilian_K7L
MSFSQRSVTYSSGGVRGGGFTAGSAIPGIRQVRVSQAYSSSGGGSGFARPGSFTSSSLQNFGSTSKRISYGVGSRPGLGFGGGAAGFGGGFGVG
TGLVPGQGIQQVTINQNLLTPMNLEIDPSIQIVRKEEKDQIKTLNNKFASFIDKVRFLEQQNKVLETKWELLQNQKTSKTSNVCPYFETYILNL
RRQLETLANEKGRLGGELKNMQDMVEDYKNKYEDEINKRTTVENDFVVLKKDVDAAYMNKVELEAKADSLNDEINFMRALCEAELRELQTQISD
TSVVLSMDNNRNLDLDSIIAEVKAQYEDMVNKSRAEAETLYQHKYEELQMTAGRHGDDLRNTKTEISEMNRMIQRLQAEIENMKNQRAKLEASI
AEAEERGELALNDARSKLAELEAALKKAKEDMARQLREYQELMNVKLALDIEIATYKKLLEGEESRLTGDGVGSVSVSVVNSSMGSSGLGGGSG
YGVSFGSGMGSSGLNFGGGIGGGSGAMKSYSVSTSSSRSSYKR

>Caecilian_K5L1
MSFYHQSTSGSGSTRKGFSASSAIIPGTRTSYSSVSMSRSGGGGGGSFGRVGGGAQSFGSRSLYNVGGSNRISIGGGIGSRYGSAGAASGLGWG
GAGGSFVLGNAPFGGSPGFPVCPPGGIKEVTINQSLLAPLNVEIDPAIQRIRTEEREQIKTLNNKFASFIDKVRFLEQQNKVLETKWNLLQQQG
HKTTKANTDPLFEVYITNLRRQLESLGNDGIRLQSDLRSVQDGVEDWKNKYEDEINKRTAAENEFVVLKKDVDAAYMNKVELESKADALTDELN
FLRNLHEAELQQMQSQISDTSVILSMDNNRALDLDSIIDEVKAQYEDIANRSRAEAESWYQTKYEELQVNAGRHGDDLRNTKTEIAELNRMQQR
LRSEIDNVKKQIANLQAAIADAEQRGELAIKDAKNKLAELEDALMKAKQEMANQLREYQNLMNVKLALDVEIATYKKLLEGEESRLSGDGVGAV
SISVVNSSSGSGYGIGGGSGLGFGGGMGLGGSSISTGGGRIGGYGTSSSFGSGGGGGGSSVKIVTKSSSHSRRF

>Caecilian_K5L2
MNRSKVFSSRSSVAVGGRSGFGSGASISQGGPRFSQGNVSSRSLHELGGTKKISCSVAGNAPSVFASRGSSYGVASGFGAGGFGGAGGFGGAGG
FGFGGGLGFGGGPGFPVCPPGGIQEVTINQSLLAPLNLEIDPTIQRVRTEEREQIKTLNNKFASFIDKVRFLEQQNKVLETKWNLLQEQGGQTT
TTRRNRLEPYFESYIANLRKQLDGLQNDKNRLDGELRNMQDSVDDFKNRYEDEINKRTAAENDFVVLKKDVDAAYMNKVELEGKLDALTDEINF
LRSLYELEIGNMQTQIADTSVVLSMDNNRALDLNSIIDEVRVQYEDIANRSRAEAEALYKNKFQELQVQVGHHGDSLKNTKSEISELNRMIQRL
RAEIENVKKHCANLQNSIAEAEERGERALKDARDKLAELEAALKKAKEDMARQLREYQELMNVKLALDVEIATYRKLLEGEECRMSGEIANPVS
VSVVSSNTTTGGGAISGGGYGGGFGVGGMGSGGGIGFGFGGGSGSSSIGFGSRGGSGNSGVKIISTTSSSSYSQRLH

>Caecilian_K5L3
MRRSQQSYSTSGGVSMGGKGFSSVSVGGMGSAGGMRSGGGGGGRVSFSSASVSHVGRSGGGLGRVGSAMGCRSLGGTKSTCITTGSVRGGGGGF
GGGHASGIIFGGGAAGSGFGGGAGGAGFGGGAGGVGFGGGVGGGGFGGVGGGGFGGAGFPGGAGGMGMAGGPGFPVCPPGGIQPVSINQSLLTP
LHLEIDPQLQKVRKEEREQIKTLNNKFASFIDKVRFLEQQNQVLETKWSFLREHTTKTGTKRDNIEPMFEAYISNLRRQLEALKNDRGRLEGEL
KNMQDLVEDFKRKYEDEINKRTAAENEFVVLKKDVDKAYMDKVDLEVKVDTLTDHINFLRALYDAELQEVHGQLSDTSVILSMDNNRDLDLSGL
IAEVKAQYEDIAARSKAEAEGVFETKFQQLQQAAGQHGDSLRNSKTEISDLNRQMQRMKAEIESVKKQIAKLQAAIAEAEERGERALKDARAKL
AELEAALQKAKEEMARQLKEYQDLMNVKLSLDIEIATYRTLLEGEESRMAGEITNEVSISVVSSQTSVSSGGAGGFGAGGAGGFGAGGGGGFSV
GGGAGGFMAGGGGGLGGGVGGGYGAGAHLSGGGGGGFGAGSGGGFVSGGSSFSSSSRGGGGGRSSGAHMVSSSSTSSKKMY

>Caecilian_K5L4
MRRSQQSYSTSGGVSMGGKGFSSVSVGGMGSAGGMRSGGGGGGRVSFSSASVSHVGRSGGGLGRSLGCRSLGGTKSISISTGYARGGGGGGGFG
GGHASGIIFGAGAGGAGFGGGAGGAGFGGGAGGVGFGGGVGGGGFGGAGGGGFGGAGFPGGAGGMGMAGGPGFPVCPPGGIQPVSINQSLLTPL
HLEIDPELQKVRKEEREQIKTLNNKFASFIDKVRFLEQQNQVLETKWSFLREHTTKTGTKRNNIDPMFEAFISTLRRRLETLKNERGRLEGDLK
NMQDLLEDFKHKYEDEINKRTTAENDFVVLKKDVDNAYMGKVELEVRVDTLTDEINFLRALYDAELQEVQGQLSDTSVILSMDNNRDLDLSGLI
AEVKAQYEDIAARSKAEAEGVFETKFQQLQQAAGQHGDTLRNSKTEISDLNRQMQRMKAEIESAKKQIAKLQGAIAEAEERGERALNDARQKLA
ELEAALQKAKEEMARQLKEYQDLMNVKLSLDIEIATYRTLLEGEESRMAGEITNEVSISVVSSQTSVSSGGAGGFGAGGAGGFGAGGAGGFGAG
GGGGFGVGGGAGGYMAGGGGGLGGGVGGGYGTGGGGGFGAGSGGGFVSGGSSFSSSSRGGGGGRSSGVAIVSTTSSSSSSKKYIKPNV

>Caecilian_K5L5
MSLRQQSQASMHRKGFSASSVGGVSGGGRRGGFSSSSASQVGGGHRSASITNRSLYNLGGNKRTSIYPSSASSIQGGGSAFGGGSMGFGVGGAG
GTGFGGFGSAAGGGGFGGGAGAGFPGGPGFPVCPPGGIQQVTINQSLLQPISVEIDPSLQKVRLEEREQIRSLNNKFASFIDKVRFLEQQNQVL
ETKWSLLQDQSHKTSIKRDNMTPLFEAYISNLKRKLDLLMNDKGRLVGELKNMQDLAEEFKRKYEEEMNKRTAAENEFVALKKDVDVTYMTKVD
LEAKVHSLTDKINFLRTLYDAELTEVQGQLSDTSVILSMDNNRDLDLSGIIAEVRAQYEDIAAISKAEAEAAFDNKFQQLQNTAGQHSDNLRQS
KNDISDMNSRIKRLQSEIETVKKQIAKLQAEIAEVEQRGELALKDARTKLAELEAALQKAKEDMAHRVKEYQALMNVKLALDIEIATYRTLLEG
EECRMAGEIINTVSISVVSSNSAISGEQFGSGGGGGHGSGFGTGQGGSSRIGVGGGMQSGSVSVSSSSKKGHQML

>Caecilian_K5L6
MMRQSQSHSQVQKGQSFGSRSVAGGSMRVQNVGGGARGFSACSAGGGGGRSKISMGSGNRAGGMSSASLCFLGGTKRTSVCSSNSVRGGGAVAG
GGFGGGVGGGVGGGVGVGCGIGVGGGAGFGGGAGFGGGAGAGGFVGGAGPGFGGPVPGGPGFPVCPPGGIKQVTINQSLLTPLNVEIDPNIQKV
RTEEREQIKTLNNKFASFIDKVRFLEQQNQVLETKWAFLQENTQKTGTKRDNIEPLFQTYISNMKRQLDKIIGERSRLDSDLKSMQVLVEDYRK
KYEDEINKRTAAENEFVVLKKDVDTAYMKKVELEAKVYGLTDEINFLRQLHDTELQEVSGGLSDTSVILSMDNNRDLDLSGIIVEVKAQYEEIA
AISKAEAEAVCDNKFRQLQATAGQAGNTIKNSKNEISDLNRQMQRMKSEIESVKKQIASLQTAIAEAEKRGEAALNDAKKKLADLEAALQKAKE
DMAHRLKEYQNLMNSKLSLDVEIATYRTLLEGEESRMSGEISSTVSISVVSGSSSVSGGGSGGYGGASGGMGGASGGYGGASGGMGGASGGYGG
ASGGYGGASGGFGGQGSSSKGYSQGGSGGSSYSSSMAVSSSSSSKKQSY

>Caecilian_K5L7
MMRQSQSHSQVQKGQSFGSRSVAGGSMRVQNVGGGARGFSACSAGGGGGRSKISMGSGNRAGGMSSASLCFLGGTKRTSVCSSNSVRGGGAVAG
GGFGGGFGGGVGGGVGGGVGVGGGIGVGGGAGFGGGAGFGGGAGFGGGAGAGGFVGGAGPGFVGPIPGGPGFPVCPPGGIKQVTINQSLLTPLN
VEIDPNIQKVRTEEREQIKTLNNKFASFIDKVRFLEQQNQVLETKWAFLQENTQKTGTKRDNIEPLFQAYISNMKRQLDKIIGERSRLDSELKS
MQVLVEDFKKKYEDEINKRTAAENEFVVLKKDVDAAYMNKVQLEARVDGLTDEINFLRHLYESELQEVSGGLSDTSVILSMDNNRDLDLSGIIA
EVKAQYEEIAAISKAEAEAVCDNKFRQLQATAGQAGNTIKNSKNEISDLNRQMQRMKSEIESVKKQIASLQTAIAEAEKRGEAALNDAKKKLAD
LEAALQKAKEDMAHRLKEYQNLMNSKLSLDVEIATYRTLLEGEESRMSGEISSTVSISVVSGSSSVSGGGSGGYGGASGGMGGASGGYGGASGG
MGGASGGYGGASGGMGGASGGYGGASGGYGGASGGFGGQGSSSKGYSQGGSGGSSYSSSMAVSSSSSSKKQSY

```
>Caecilian_K5L8
MMRQSQSHSQVQKGQSFGSRSVAGGSMRVQNVGGGARGFSACSAGGGGGRSKISMGSGNRAGGMSSASLCFLGGTKRTSVCSSNSVRGGGVVAG
GGFGGGFGGGVGGGVGGGVGVGGGAGFGGGAGFGGGAGAGGFVGGAGPGFGGPVPGGPGFPVCPPGGIKQVTINQSLLTPLNVEIDPNIQKVRT
EEREQIKTLNNKFASFIDKVRFLEQQNQVLETKWAFLQENTQKTGTKRDNIEPLFQAYISNQKRQLDKIIGERSRLDSELKSMQVLVEDFKKKY
EDEINKRTAAENEFVVLKKDVDAAYMNKVQLEARVDGLTDEINFLRHLYESELQEVSGGLSDTSVILSMDNNRDLDLSGIIAEVKAQYEEIAAI
SKAEAEAVCDNKFRQLQATAGQAGNTIKNSKNEISDLNRQMQRMKSEIESVKKQIASLQTAIAEAEKRGEAALNDAKKKLADLEAALQKAKEDM
AHRLKEYQNLMNSKLSLDVEIATYRTLLEGEESRMSGEISSTVSISVVSGSSSVSGGGSGGYGGASGGMGGASGGYGGASGGMGGASGGYGGAS
GGYGGASGGYGGASGGFGGQGSSSKGYSQGGSGGSSYSSSMAVSSSSSSKKQSY

>Caecilian_K5L9
MSFQIGKSKCGNANRVSFSSSSVSRASYGGSGRGSSGYSSRSLINLGRSKQISLGGGGGSSRFSARSGYGFGGGAGGVFAGGISGGLGGGIGGG
YGGSPGFPVCPPGGIQEVTINQSLLAPLNLEIDPAIQRIRQEEREQIKTLNNKFASFIDKVRFLEQQNKVLETKWALLQEQSPRGGTNRNNLDP
IFVAYINKLKRQLDNITNDRSRLNSELKNMQDVVEEFKKKYEDEINKRTATENEFVVLKKDVDAAYMKKVDLQSKVDSLVDEINFLRALYEAEL
SQMQTQVSDTSVILSMDNNRDLNLDSIIAEVKAQYEEIANRSRAEAESWYQSKYEELQVTAGRHGDDLRSTKTQISELTRMMQRLKSEIEILKK
QIASLQAAVSEAEERGELALKDARKKLSDLETALQKAKEDMARQLREYQELLNVKLALDVEIATYKKLLEGEECRMSGEHGSAVSISVVSSSSG
SRGGIGGSIGSGIGSGIGGSISGGIGGGSYGSYGGGFSSGSGRGGTSIVKKTTYSSQSSSKRY
```

Suppl. Fig. S2. Amino acid sequences of type I and II keratins of platypus, birds, and amphibians. The amino acid sequences of type I (**A**) and type II (**B**) keratins used for comparative analysis of cysteine contents are shown in FASTA format. The sequences are sorted according to species and, within each species, according to the order of the corresponding genes in the type I or type II keratin gene clusters, unless this order is currently unknown (axolotl and caecilian). Accession numbers and data about the genes are provided in Supplementary Tables S7-S13. Preliminary names and accession numbers of zebrafish (*Danio rerio*) keratins are provided in Supplementary Table S14. Keratin amino acid sequences of species shown in Figures 1 and 2 are not included here but in Suppl. Fig. S1. Species: platypus (*Ornithorhynchus anatinus*), kiwi (*Apterix australis*), ostrich (*Struthio camelus australis*), zebra finch (*Taeniopygia guttata*), Nanorana/Tibetan frog (*Nanorana parkeri*), axolotl (*Ambystoma mexicanum*), caecilian (*Microcaecilia unicolor*).

A

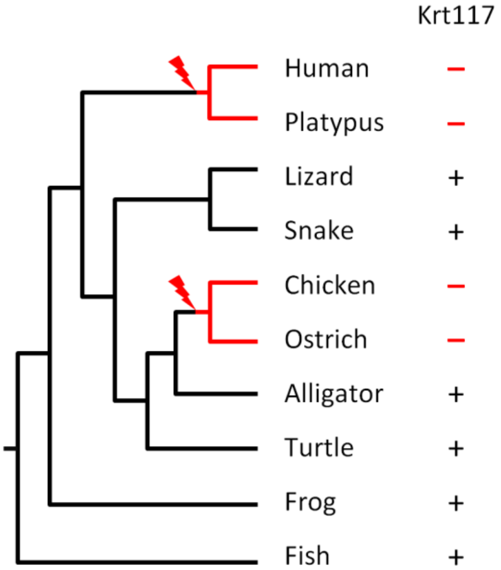

B

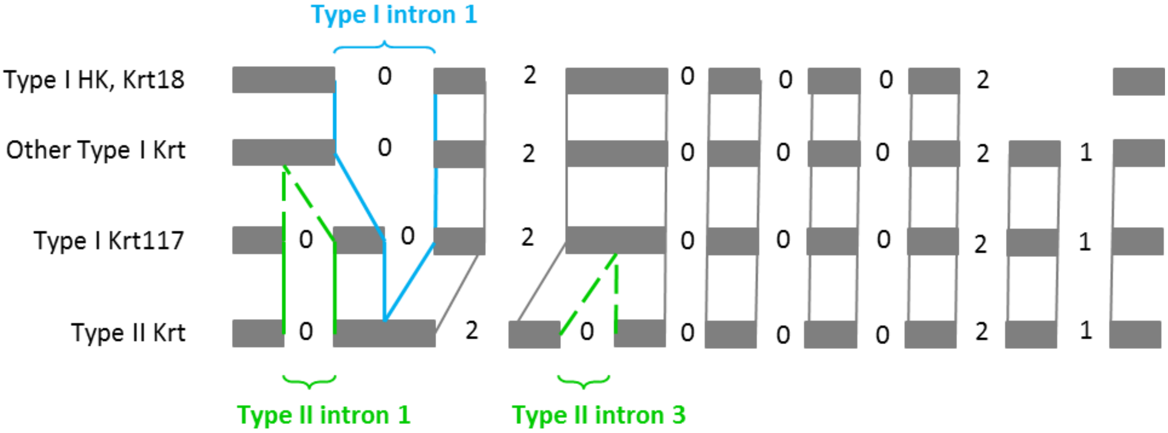

C

```
                1                                                                                80
   Fish_K117   M--SVPFTQQGFSSSSLGGWMLDSPCIVPQRYSHSMYGGAGCFRTRISQGLYFNS----------------SCSAGGDWP
   Frog_K117   MSFSTRSISQSARFGVFASPGVNRARSVA-----GGASTVRMSSANMTSSAFA------------------ASPAFEVGV
  Allig_K117   MA-SSLS-RNLFCFGVPQGPRLNWISAAGLQ---PGLLSIHGGAGSIGSYLSSASDAFVKCVPGGSYEPGIGSGASGIGL
 Turtle_K117   MA-S-FS-RS----GVTLGPRLSWVRVVGLQGGVPGGLGVCGGAGSTSSRLFSGSDGIVG--------PGLGGGAREPGL
 Lizard_K117   MS-TTFSSRSLSFSGSVRGPRSGSTLSIARPQTASNIYGVMNSLPPSAKDTMA------------------RRYGPETDI
  Snake_K117   MP-TTFSSHSMS-SGSIQGPRGRRLLALGRPQAVPSIHRVIRDKTRLSYGD--------------------GQGFMDTNI
               <----------------------------- encoded by exon 1 --------------------------------

               81                                                                              160
   Fish_K117   ASNGGKFTMQNLNDRLACYLEKVRSLENKNRELQLNIDEFRVKTTYI-TKDYANYFSIISDLKKKITRSCSENQNLLLQI
   Frog_K117   TSNNGKETMQNLNDRLSNYLERVRSLELANHELERKIREYLDKKAAVGSMDVSGYYKTINLLRSQINDATIDNTRLVLSI
  Allig_K117   LGMNEKDTMQNLNDRLAAYLERVCSLEEANGQLEQRIQELQVKKTLARHYDPSSCFSTIAELRSQIQDESVRNSQLTLQL
 Turtle_K117   LSMNEKDTMQNLNDRLAAYLETVRSLEEANGQLERQIREVYAKRAQAGCQDLSGYFSTIAELRSQIQAASTSNAQLILQI
 Lizard_K117   LDINQKGTMQNLNDRLAAYLGRVHSLEESNAQLEQNIREYYAKRASAGGPDLSGYFNTLSELKAKIEQETLNNAGLLLRI
  Snake_K117   LDMNEKGTMKNLNDRLAAYLDKVLSLEKSNMQLEQNIQEFYTKQTSTGTHDMSAYFNAMIQMKSEIQKETLKNAGLLLQI
                encoded by exon 1 --><---------- encoded by exon 2 ------------><- enc. exon 3

               161                                                                             240
   Fish_K117   ESSRLAAEDFKMKSEMEINMRTTVEADLLDVRRFRDGLTLTMSSLELTLEGLNEERIPMDREHDEEMEERRRRGAGEVKV
   Frog_K117   DNAKLAADDFKIKYESELAIRTGAESDIAGLRRVLDELTLNKTDLELDIESLKEELIYLKKSHEEELAVVRSSAKGNVDV
  Allig_K117   DNAKLAANDFRMKYEAELATRLGVESDIKGLRKVLDGLTEARASLQVQIESLKEELTYLQQNHGEEVAALQGHLGGSVSV
 Turtle_K117   DNATLAADDFRVKFESELAIRRGGESDVAGLRKVLDEVTLSRASLQEELGSLQEELAQLQKNHGEEAAALQGRLGGSVSV
 Lizard_K117   DNAKLAADDFKVKLESEVAIRLSLEGDVNGLRKVLDDVNASRASLQVQVDNLQEELVYLKRNHEEEVASLQGRLGGTVNV
  Snake_K117   DNARLAADDFRVKLESEVAMRLSLEGDLGGLRKALEELNASRASLQVQADNLQEELACLKRNHKEEVASLQGHLGGTVNV
               enc. exon 3 ><-------------- encoded by exon 4 -----------------><- enc. exon 5
```

```
             241                                                                            320
  Fish_K117  EVDDSGSCDLEKVLAEMREQYEIMIKNNKKEIEKWYQKNIKALEEKIRIFTTEVETHRSEISVLKRSYQSLEITRQSLCT
  Frog_K117  QVDSAPPVDLAQIMTDVRAQYESMMEKNRQEVEAWYRGQSENLNKEVAINTTALQTSKSEITDLKRTIQSLEIELQSLLS
 Allig_K117  EVDASPGINLVKTLAEIRDQYEDIIEKNQREAEAWHKQQCETFTQEVATSSEALQAARTKATELKRIVQSLEIEFQMLQS
Turtle_K117  EVDSAPGVDLVKTLAEIRDQYEDVIERNHREAAAWHKEQCETVAQEVATSAEAVQGARTKLTELRRVVQGLEVELQSLYS
Lizard_K117  EVDSMPGVDLQKVLAEIRDQYEDVMEKNRQEAEALHKAQCDAINQQVADSSEALQAAQLKITELRRLAQALEIELQSLRS
 Snake_K117  EVDSMPGTDLQKVLGEVRDQYEGVMEKNRQEAEALHQSQCDALNQEVSVSTEALQSAQMKMVELKRLAQALEIELQSLWS
             ------ encoded by exon 5 ------------->&lt;--------- encoded by exon 6 ------------

             321                                                                            400
  Fish_K117  EIECWQQNICAVNKQTSAQLSCHQSVITALETKLQQMKACITEHRIKHDTVLELKTRLEGEIAEYRRLLEGEGCEHKAL-
  Frog_K117  MKNALEGTLAETEARYGSELNHLQAMITQVELEMQNLRSDAERQSFEYKLLLDIKTRLEMEIATYRRLLEGE--DNRFSQ
 Allig_K117  TKEALEGTLAETESHYGVEMAQLQNLVAGKEAELAQLRTDTQRQANEYQHLLDLKTRLEVEIATYQHLLEGQ--EVRSEA
Turtle_K117  MKEALEGTLAETRAGSGAELEQLRECVARKEAELAQLRTDTQRQAEEHQQLLDLKTRLEMEIATYRRLLEGD--DVRSDA
Lizard_K117  TKIALEGTLVETESRYGMELSRLRELISARESELFQLRSEAQRQAEDYKRLMDIKNKLEQEIATYRRLLEG------LDT
 Snake_K117  MKEALEGTLAEVESHYGLELSQLQNLVAAREAELLQLKSDAQNQAEDYKRLMDIKNRLEQEIATYRCLLEG------SES
             >&lt;----------------------- encoded by exon 7 -------------------------------->&lt;--

             401                                                 452
  Fish_K117  VIKQVTEEVAEQKPHIE-------KRVKIIVEELVDGQVVSSSVDIQVHDVE
  Frog_K117  TETQK-----DVTIISKEQTKSSIKKVKTVIEEVVDGKVVSSRVEEMTEKL-
 Allig_K117  KSP-KQEILPEA--VSKAPTT---RRIKTLIEELVDGEVVSSCIEEVEHPL-
Turtle_K117  KSPVRQ-TPPEA--VNSSPTS---RRVKTVIEELLDGRVVSSRTEEVEHPL-
Lizard_K117  EPP----SPPEHT------TS---RRVKTLIEELVDGQVVSSRVEEVEQPL-
 Snake_K117  EPL----TTPEPS------VS---RRVKTVIEELVDGKVVSSRMEEVEHQL-
             exon 8 -->&lt;--------- encoded by exon 9 ------------>
```

Suppl. Fig. S3. *Krt117*, a keratin gene with a unique exon-intron structure, has been lost in mammals and birds. (**A**) The presence (+) or absence (-) of *Krt117* was mapped onto a simplified phylogenetic tree of vertebrates. Gene loss events (flash symbols) were inferred from the distribution of *Krt117* in extant species. (**B**) Exon-intron structure of *Krt117* and other keratin genes. Exons are depicted as grey boxes. The numbers between the exons indicate the phase of splicing of the intron. Homologous splice sites are connected by vertical lines. Intron 1 of type I keratin genes is not conserved in type II keratin genes, whereas introns 1 and 3 of type II keratin genes are not conserved in type I keratin genes. *Krt117* has the same introns as other type I keratins but, in addition, it has an intron at a position homologous to intron 1 of type II keratins. Note that the lengths of exon symbols are not proportional to the lengths of the exons. (**C**) Amino acid sequence alignment of K117 proteins. The segments encoded by the different exons of the *Krt117* gene are indicated below the sequences. Residues identical in all species are shown with red fonts, and weakly conserved residues are shown with blue fonts. The alignment was made with the Multalin program. Species: alligator (*Alligator sinensis*, abbreviation: Allig), fish (guppy, *Poecilia reticulata*), frog (*Xenopus tropicalis*), lizard (*Anolis carolinensis*), snake (*Protobothrops mucrosquamatus*), turtle (*Chrysemys picta bellii*).

>Ami_K78LT
MSLLSPSCKSGDGGKDFSSCSAHCGSVGVGSSSSSICQLGESRGGKGYFGYGYGSKSLHNLGGTRRISVCNGYQG
GGRGYGVCWERYGGWGYGRGGFGGGNFGGGGARFGGICRGGLIGGGLGGGDIGRSWGIQGVHVNGNLLRPVHVEI
DPDFQRVRAHEREQIKSLNDKFACFIDKVRCLEQENQVLLTKWELLQQQGGQTGVHRDIEPFFQSYICHLRQQRD
TLQSQKEELNLEDCKMLQIVNEYKASYEKEIDRRLCTEEEFVNLKKNLDCIYMGKMEVGVKVNMLREEIEFLKCL
YATERSDVQRIAQDTNVVVSMDNCRDLDVEGIIAEIRREYEGLAQKSQAEVNAVYQSKYQDLHNTWGRHCDRLRT
RQHEIQELTRLIQKLRLDIGEVKKKNACLQEEIANAERHGCCAVSDAQEKLGELRCSLLKAKDNLAHLLRDYQEL
LNVKMALDIEIATYRALLEGEECRITEGLPGPIPVINHGTWVSGSAGGRYGGFPWCWGGYGGFRGGHCGWGGGIN
WGIEGGHCSIRRGFSSRSGGHYSRNIRCSPPNYGGSKGGGFSSRSEGGNVKVCISRGGEPSFGGSCSGGVGGIFG
MGGSCESGGGGHGSVGGSQGGGGGFISGGECSFGDGRYGYGKSDCCVVGGYGSGVGGHSGVAVSSEWGGGSSGSE
GYSSGGTRFGSRRGGRSSGRNCCVFGGYGSRRTGTSGIQISSGWTGGSAKGGECTARDGVSGSGGSGWSRGRDCC
VIGGDGPGAGVSDGYSSGAGGYSSGVGYGCGGREYGSGGAIISGEGSSSGASASCSTGPYGPAGWDHAGSGGCVS
VGGGYDSGVTAIPIPVGGSSSGGVGSCGSGAGGNLSGERYSFGGRGYGCGVSSISGGEGSCGGDASIGVGHSESV
GYISGARGHSSVNIGSDECSTGCGSGGGAYSAGAGASSGMERCSSGVGGSSGGVVDICGSGVHSSRVVGSSSGAG
GCSSGGGCSGGSGGHTSGSTNTPGAVGHCSSGSGGGSCFGSGGYGSGGGGGSGERGYSSSRIGGCASSTYSPRAG
GSFGGGGLRSGGIGRTGGSSGGIGPGGYGFGAGGSSSGGGYSCGSGGYGSIRGSYSGSGGYGSGTGGYGSGAGGS
PGGRGYSCGSGGYSSGAGGSTSGGVCSSGGRGYGSGSGEYDSGAGGLSSGDVCSTSGGYGSGGGGSGGYGSGSGG
YDSGAGRLSSGDVCRISGGGYDSGVGGSGGYGSGSGAYDSGAGRLSSGDVCRISGGGYGSGGGDSGEYGSGSGAD
DSGAGRLISGDVCRISGRGYNSGAERSEEYSSGNEAYDSEAGRLSCGDVCSVSGRSPGADGFCSGDACSISGGGY
GSGGGGSGGYGSGSGAHDSETGRLSSGDVCSIGAGGYGSGGGGSGGYGSGSGAHDSETGRLSSGDVCSIGAGGYG
SGVGGSGGYGSRSDGYSSGAGGSSSVRVCSISGGGYSSGAGGSSSRGERSISGGGYDSGICGGGGYSGGGGYGSG
VGGYSSGEVGGSTTGGISGGGEYGSGTGGSCEVGSGSVGGGSLGGTGYSSGVGESSSGGIGPCGSGGHGSVGRGY
SGSIGAGDYGSGHGDSCEVAGSGSGLGGSSGAGASGGGGYSPGGTGSSGDVGGSSGEGVGCGSGGFGSIGRNYSG
SGGYSSGPGGYGSGAGGSTCEGEDYGFGGRGSPGGGGGSFGEAGSCSSGRYGSVGGGRSGSGGYCSGEGGSSGGR
YGSGVGGSSRIGGSSGGGGRYSSGGEGYTSRGWSISGGRASCGSGRYGPGAWGYGSGAGGSSSRVGYSCGGGGYS
SGGRWGSSGWAGSTGGEGYSSGAGRHGSSSGRYSSFSGHAGSCLGDGYGSRSWGHYEGRGSGSGGSSGWGGGCGP
GGTDSSGVGGHSLGETGSPGGIGSCSGGGGSSGGGGLGPGSGGFDSGAGGSSSGGVCSIGGGGYGSGGVGTGSEG
VCSIDGGGYGSGSGGHGSGIGGSSSGGVCSIGGGGYGSGSGGYGSGEGGSGFGSGGYGSGVGGSSSGGVCTIDGG
GYGSGSGGHGSGIGGSSSGGVCTIGGGEYGSGSGGYSSGGGGSGSGAGGYGSGAGGSSSGGVCTIGGGEYGSGSG
GYSSGGGGSGSGAGGYSCGAGGGSGSGTGGGEGSGSGRYSCGGISGGTGSGRYGYGGGSYSGGRLSSRGSSTR

>Asi_K78LT
MSQLSPSCKSGDGGKDFSSCSAHCGSVGVGSSSSSICQSGESRGGRGYFGYGYGSKSLHNLGGTRRISVCDGYQG
GGRGYGVCWERYGGWGYGRGGFGGGNFGGGGARFGGICRGGLVGGGLGGGDIGRSWGIQGVHVNGNLLRPVHMEI
DPDFQRVRAHEKEQIKSLNDKFACFIDKVRCLEQENQVLLTKWELLQQQGGQTGVHRDIEPFFQSYICHLRRQRD
TLQSQKEELNLEDCKMLQIVNEYKASYEKEIDRRLCTEEEFVNLKKNLDCIYMGKMEVGVKVNILREEIEFLKCL
YAMELSDVQRIAQDTNVVVSMDNCRDLDVEGIIAEIRREYEGLAQKSQAEVNAVYQSKYQDLHNTWGRHCDRLRT
RQHEIQELTRLIQKLRLDIGDVKKKNACLQEEIADVERRGCCAVSDAQEKLRELRCSLLKAKDNLAHLLRDYQEL
LNVKMALDIEIATYRALLEGEECRITEGLPGPIPVINHGTWVSGSAGGRYGGFPWCWGGYGGFRGGHCGWGGGRN
WGIVGGHCSIRRGFSSRSGGHYSRNIRCSPPNYGGSKGGGFSSRSEGGNVKVCISRGGEPSFGGSCSGGVGGIFG
MGGSCESGGGGHGSVGGSQAGGGGFISGGECSFGDGRYGYGKSDCCVVGGYGSGVGGHSGVAVSSKWDGGSSGCE
GYSSGGTRFGSRRGGRSSGRNCCVFGGYGSRRTGTSGIQISSGWTGGRAEGGECTARDGVSGSGGSGWSRGRDCC
VIGGDGPGAGVSDGYSSGAGGYSSGVGYGCGGREYGSGGAIISGEGSSSGASVSCSTGPYGPAGWDHAGGGGCVS
GGGGYDSGVTVIPIPVGGSSSGGVGSCGCGGYGSGAGGSLSGERYSFGGRGYGCGVSSISGGEGSCGGDASIGGG
HSGSVGYISGARGHSSVNIGSDECSTGCGSGGGAYSAGAGASSGMERCSSGVGGSSGGVVDICGSGVHSSRVVGS
SSGAGGSSSGGGCSGGSGGHTSGSTNTPGAIGHCSSGSSGSGGGSRFGSGGYGSGGGGGSGERGYSSSRIGGCAS
STYSPGAGGSSGGGGLRSGGIGRTGGSSGGIGPGGYGSGAGGSFSGGGYSCGSGGYGSIRGSYSGSGGYGSGTGG
YGSGAGGSPGGRGYSCGSGGYSSGAGGSTSGGVCSSGGGGYVSGSGEYDSGAGGLSSGDVCSTSGGGYGSGGGGS
GGYDSGAGRLSCGDVVDTSGGYGSGGGGSGGYDSGAGRLSSGDVCSISVGGYDSGVGGSGGYGSGSGAYDSGAGR
LSSGDVCRISGGGYGSGGGDSGEYGSGSGADDSGAGRLISGDVCRISGRGYNSGAERSEEYSSGNGAYDSEAGRL
SCGDVCSISGRSPGADGFCSGDACSISGGGYGSGGGGSGGYGSGSGEHDSETGRLSSGDVCSIGAGGYGSGGGGS
GGYGSRSGGYSSGAVGSSSVRVCSISGGGYSSGAGGSSSRGERSISGGGDDSGICGGGGYSGGAQGWGXGYSSGE
VGGSTTGGIPGGRGYGSGTGGSCEVGSGSVGGGSLGGTGYSSGVGESSSGGIGPCGSSGHGSVGRGYSGSIGAGD
YGSGHGGSCEVAGSGSGLGGNSGAGASGGGGYSTGGTGSSGDVGGSSGEGVGCGSGGFGSIGRDYSGSGGYSSGP
GGYGSGAGVSTCEGEDYGFGVRGSPGGGGGSCGEAGSCSSGRYGSVGGGRSGSGGYCSGEGGSSGGRYGSGVGGS
SRIGGSSGGGGRYSSGGEGYTSRGWSILGGRASCGSGRYGPGAWGYGSGAGGSSSQVGYSCGGGGYSSGGRSSSG
EMGTCSSGRYDSGAGGYSPGGRGGSSGWAGSTGGEGYSSGAEGSSGAGRHGSNSGRYSSFSGHAGSCLGDGCGSW
SWGHYEGRGSGSGGSSGWGGGCGPGGADSSGVGGHSLGETGSPGDMGSCSGGGGSSGGGGLGPGSGGFDSGAGGS
SSGGVCSIGGGGYGSGGVGTGSEGVCSIDGGGYGSGSGGHGSGIGGSSSGGVCSIGGGEYGSGSGRYGSGAGGSS
SGGVCSIGGGEYGSGSGGYSSGGRGSGSGAGGYGSGVGGSSSGGVCTIGGGGYSSGSGGFGSGRGGSGSGAGGYS
CGAGGGSGSGIGGGEGSGSGRYSCGGVSGGTGSGRYGYGGGSYSGGRLSSRGSSTR

```
>Gga_K78LT
MSHHFFRFGDGNFSSSSAHCGTLGSGRGSSAMSHWEDDGGQRFGGYGYGYGHGYGSRSLHNLSGFRNFSTGGIYG
GDGGWYGRHGGYYDGRYLDRDLGRRVYFVGDFQGGAGRMYGGGSSRGMLSGGLREQGAGQGSAHPAAGGGIEEVH
VNTNLLRPIQVQVDPEFQRMRSDEKEQIKTLNXQFASFIDKVQCLERQNQALMTKWELLQQQSSRPEESRSITSF
FQSYINNLQRQLDMLQSQKEQLDPEAYEMLQLVEDYKKRFEDEINKRASKEEEYVELKKELDSTYMRKMEFEVRV
EILKQELEFLRCLYEAELSQLQTVVDNTNVILSMDNGREVNMDGIIEEVRQEYEKIAQKGKDEVNAMYQGRYQDL
QNMRVNQREQLRYTYQEIQELTRQIQRLQPEIEIVRRENDSRQEAIRDAEQRGSSAVKDGQKKLQELENALQQAR
EDLARILHDYQELLNAKLSLDVEIAMYRSLLEEEETRIQEGSPATVRVVTRSGSSAGGFGREYGDLNREKEEMST
DDSNRMTWSEITPGGGREGERRDSGSTYGSREHRGGEAGRYHTGRSSGSSSGYGYETGGRISRAGSTSGGGHSSG
GFGGSSGSGRCSPHGGGLSSGGSSGSGRRGSISGEGGSSGHGGSSYGSGSGGSICGGGGSGSSSGGSFGSGGSSS
GGGHSSGGFGSSSGSGSGRRGSISGGGGSSGHGGSSYGSGSGGSICRGGSSGAGSGSGGSFGSGGSSSGGGHSSG
GFGGSSGSGRCSPHGGGLSSGGSSGSGRRGSVSGEGGSSGHGGSSYGSGSGGSICGGGGSGSSSGGSFGSGGSSS
GGGHSSGGFGSSSGGFGSSSGSGSGRRGSISGGGGSSGHGGSSYGSGSGGSICRGGSSGAGSGSGGSFGSGGSSS
GGGHSSGGFGGSSGSGRCSPHGGGLSSGGSSGSGRRGSISGEGGSSGHGGSSYGSGSGGSICGGGGSGSSSGGSF
GSGGSSSGGGHSSGGFGSSSGSGSGRRGSISGGGGSSGHGGSSYGSGSGGSICRGGSSGAGSGSGGSFGSGGSSS
GGGHSSGGFGGSSGSGRCSPHGGGLSSGGSSGSGRRGSISGEGGSSGHGGSSYGSGSGGSICGGGGSGSSSGGSF
GSGGSSSGGGHSSGGFGSSSGSGSGRRGSISGGGGSSGHGGSSYGSGSGGSICRGGSSGAGSGSGGSFGSGGSSS
GGGHSSGGFGGSSGSGRCSPHGGGLSSGGSSGSGRRGSISGEGGSSGHGGSSYGSGSGGSICGGGGSGSSSGGSF
GSGGSSSGGGHSSGGFGSSSGSGSGRRGSISGGGGSSGHGGSSYGSGSGGSICRGGSSGAGSGSGGSFGSGGSSS
GGGHSSGGFGGSSGSGRCSPHGGGLSSGGSSGSGRRGSFQGXGGSSGHGGSSYGSGSGGSICGGGGSGSSSGGSF
GSGGSSSGGGHSSGGFGSSSGSGSGRRGSISGGGGSSGHGGSSYGSGSGGSICRGGSSGAGSGSGGSFGSGGSSS
GGGHSSGGFGGSSGSGRCSPHGGGLSSGGSSGSGRRGSVSGEGGSSGHGGSSYGSGSGGSICGGGGSGSSSGGSF
GSGGSSSGGGHSSGGFGSSSGSGSGRRGSISGGGGSSGXGGSSYGSGSGGSICRGGSSGAGSGSGGSFGSGGSSS
GGGHSSGGFGGSSGSGRCSPHGGGLSSGGSSGSGRRGSISGEGGSSGHGGSSYGSGSGGSICGGGGSGSSSGGSF
GSGGSSSGGGHSSGGFGSSSGSGSGRRGSISGGGGSSGHGGSSYGSGSGGSICRGGSSGAGSGSGGSFGSGGSSS
GGGHSSGGFGGSSGSGRCSPHGGGLSSGGSSGSGRRGSISGEGGSSGHGGSSYGSGSGGSICGGGGSGSSSGGSF
GSGGSSSGGGHSSGGFGSSSGSGSGRRGSISGGGGSSGHGGSSYGSGSGGSICRGGSSGAGSGSGGSFGSGGSSS
GGGHSSGGFGGSSGSGRCSPHGGGLSSGGSSGSGRRGSISGEGGSSGHGGSSYGSGSGGSICGGGGSGSSSGGSF
GSGGSSSGGGHSSGGFGSSSGSGSGRRGSISGGGGSSGHGGSSYGSGSGGSICRGGSSGAGSGSGGSFGSGGSSS
GGGHSSGGFGGSSGSGRCSPHGGGLSSGGSSGSGRRGSISGEGGSSGHGGSSYGSGSGGSICGGGGSGSSSGGSF
GSGGSSSGGGHSSGGFGSSSGSGSGRRGSISGGGGSSGHGGSSYGSGSGGSICRGGSSGAGSGSGGSFGSGGSSS
GGGHSSGGFGGSSGSGRCSPHGGGLSSGGSSGSAXSISGEGGSSGHGGXGISYGSGSGGSICGGGGSGSSSGGSF
GSGGSSSGGGHSSGGFGSSSGSGSGRRGSISGGGGSSGHGGSSYGSGSGGSICRGGSSGAGSGSGGSFGSGGSSS
GGGHSSGGFGGSSGSGRCSPHGGGLSSGGSSGSGRRGSISGEGGSSGHGGSSYGSGSGGSICGGGGSGSSSGGSF
GSGGSSSGGGHSSGGFGSSSGSGSGRRGSISGGGGSSGHGGSSYGSGSGGSICRGGSSGAGSGSGGSFGSGGSSS
GGGHSSGGFGGSSGSGRCSPHGGGLSSGGSSGSAXGSISGEGGSSGHGGXISYGSGSGGSICGGGGSGSSSGGSF
GSGGSSSGGGHSSGGFGSSSGSGSGRRGSISGGGGSSGHGGSSYGSGSGGSICRGGSSGAGSGSGGSFGSGGSSS
GGGHSSGGFGGSSGSGRCSPHGGGLSSGGSSGSGRRGSISGEGGSSGHGGSSYGSGSGGSICGGGGSGSSSGGSF
GSGGSSSGGGHSSGGFGSSSGSGSGRRGSISGGGGSSGHGGSSYGSGSGGSICRGGSSGAGSGSGGSFGSGGSSS
GGGHSSGGFGGSSGSGRCSPHGGGLSSGGSSGSGRRGSISGEGGSSGHGGSSYGSGSGGSICGGGGSGSSXGGSF
GSGGSSSGGSFGSGGSSSGGGHSSGGFGSSSGSGSGRRGSISGGGGSSGHGGSSYGSGSGGSICGGGGSGSGSGF
GGSFGSGGSVSHDAGTFSGGSCISPGSHSCGVSGSSSCGEGYGYVGSCSGDISSSGREGYGFESGHFSGGDGCYE
GGGECGGYEYYGPRAETGECSSETGPSCPRYGYLSGSGIGYGTSDFSGDYYHK
```

Suppl. Fig. S4. Predictions of amino acid sequences of K78LT keratins of archosaurs. The intermediate filament domains are underlined. Cysteine residues are highlighted by red shading. Glycine and serine residues, which are highly abundant in the head and tail domains, are marked by light and dark grey shading. “X”s with blue shading indicate uncertain positions corresponding to frame-shifts in the genomic DNA sequence available in GenBank. Note that the sequences of K78LT proteins of the Chinese alligator (Asi, *Alligator sinensis*), translation of GenBank gene LOC102373665 + two 3’-terminal exons) and chicken (Gga, *Gallus gallus*) (Suppl. Table S1) after these frameshift sites are hypothetical. The sequence of the American alligator (Ami, *Alligator mississippiensis*) K78LT (Suppl. Table S2) is the translation of a gene without frame-shifts.

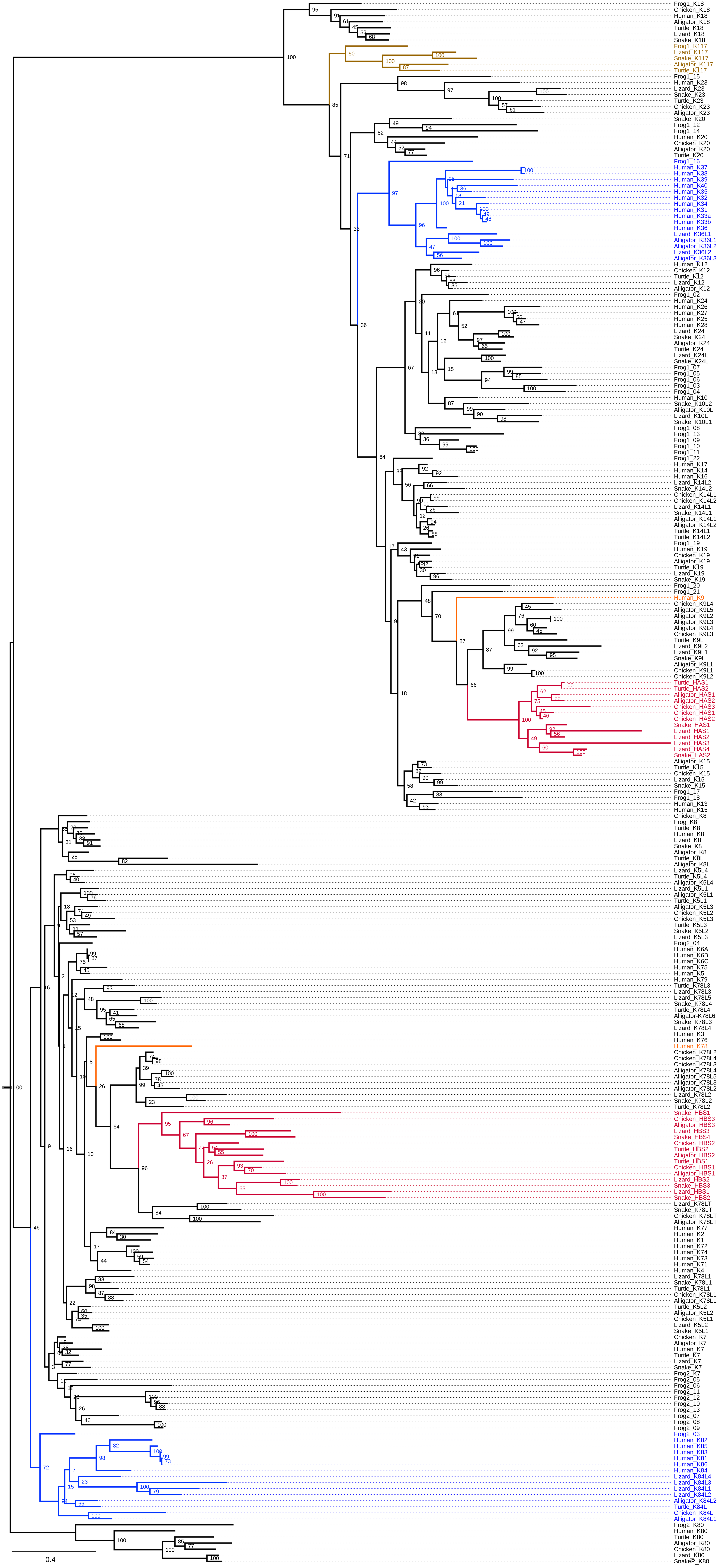

Suppl. Fig. S5. Maximum likelihood phylogenetic analysis of type I and type II keratins of tetrapods. The phylogenetic tree was generated with PhyML. Hair keratins and orthologs are indicated with blue fonts and lines. Sauropsid-specific cysteine-rich keratins (HAS, HBS) are shown with red fonts and lines. Bootstrap values are indicated. Bayesian phylogenetitic inference yielded similar results. Amino acid sequences: Suppl. Fig. S1. Accession numbers: Suppl. Tables S1-S6.

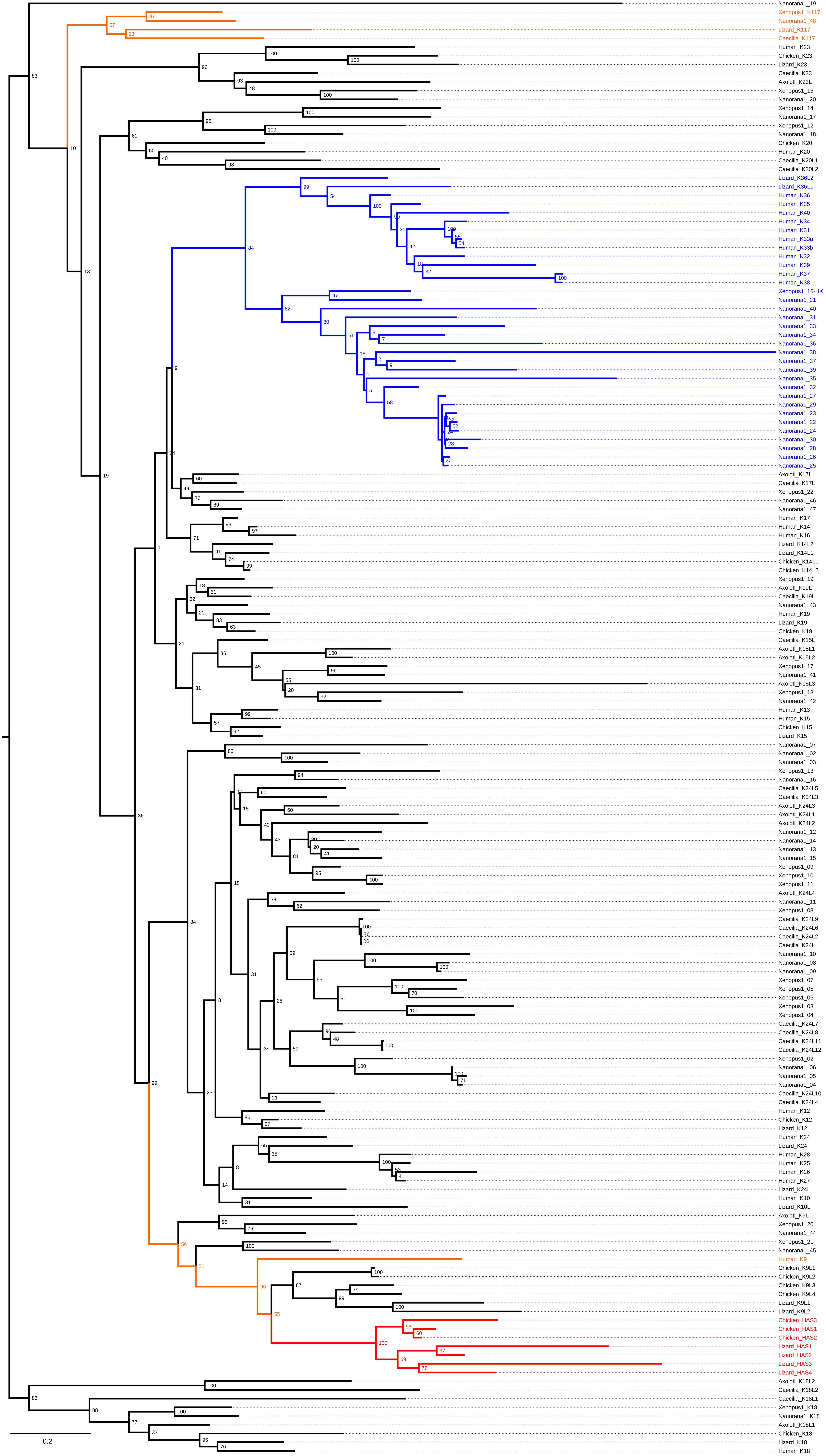

Suppl. Fig. S6. Maximum likelihood phylogenetic analysis of type I keratins of amphibians (*X. tropicalis*, Tibetan frog, axolotl, caecilian), chicken, lizard, and human. The phylogenetic tree was generated with PhyML. Sauropsid-specific cysteine-rich keratins (HAS) are shown with red fonts and lines. Hair keratins and orthologs are indicated with blue fonts and lines. Note that hair keratin orthologs of Xenopus and Nanorana have a low cysteine content. Bootstrap values are indicated. The scale bar represents substitutions/site. Bayesian phylogenetitic inference yielded similar results. Amino acid sequences are provided in Suppl. Fig. S1 and S2. Accession numbers and information about the genes is shown in the Suppl. Tables S1-S13.

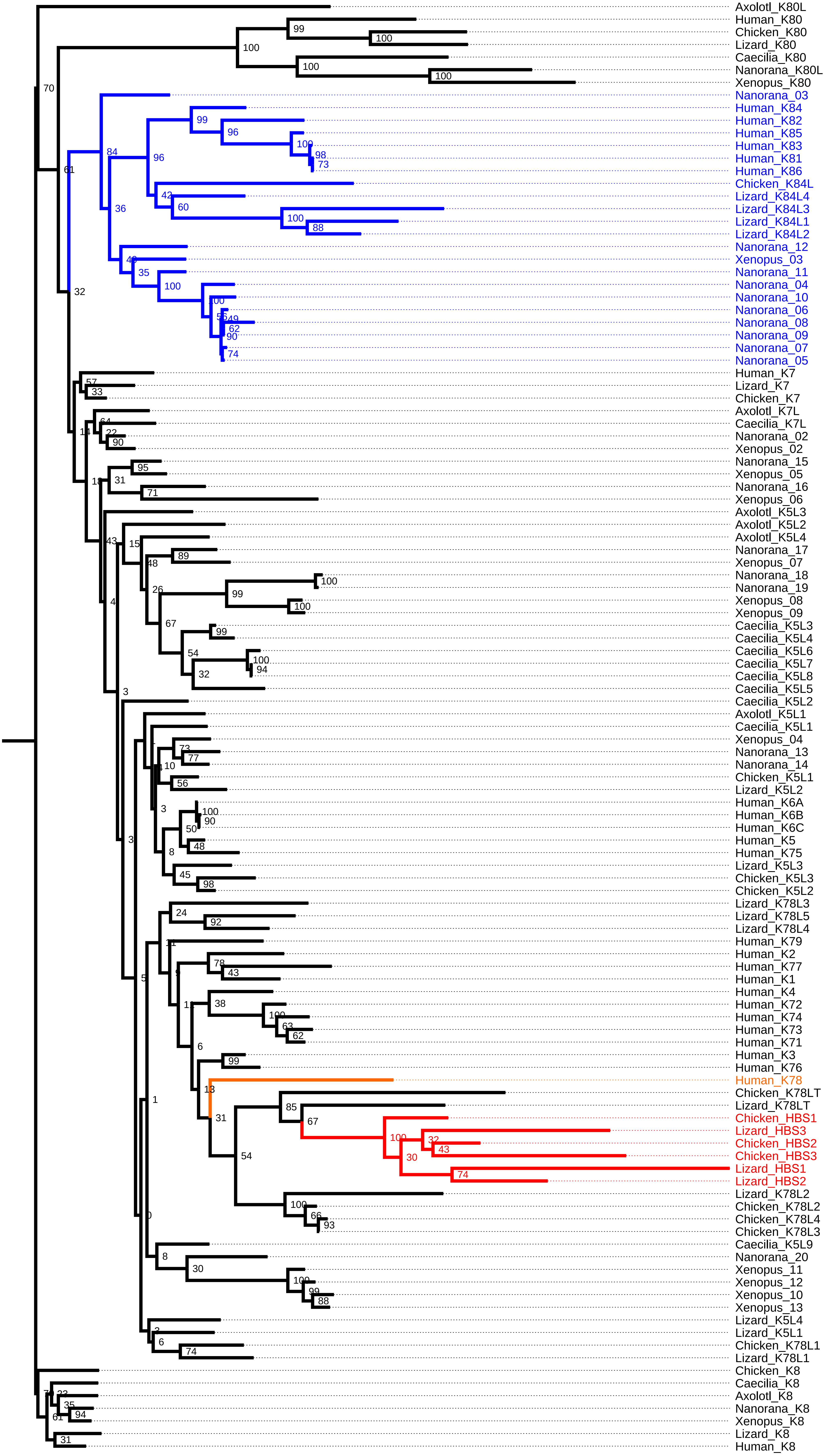

Suppl. Fig. S7. Maximum likelihood phylogenetic analysis of type II keratins of amphibians (*X. tropicalis*, Tibetan frog, axolotl, caecilian), chicken, lizard, and human. The phylogenetic tree was generated with PhyML. Sauropsid-specific cysteine-rich keratins (HBS) are shown with red fonts and lines. Hair keratins and orthologs are indicated with blue fonts and lines. Note that hair keratin orthologs of Xenopus and Nanorana have a low cysteine content. Bootstrap values are indicated. Bayesian phylogenetitic inference yielded similar results. Amino acid sequences: Suppl. Fig. S1 and S2. Accession numbers and information about the genes: Suppl. Tables S1-S13.

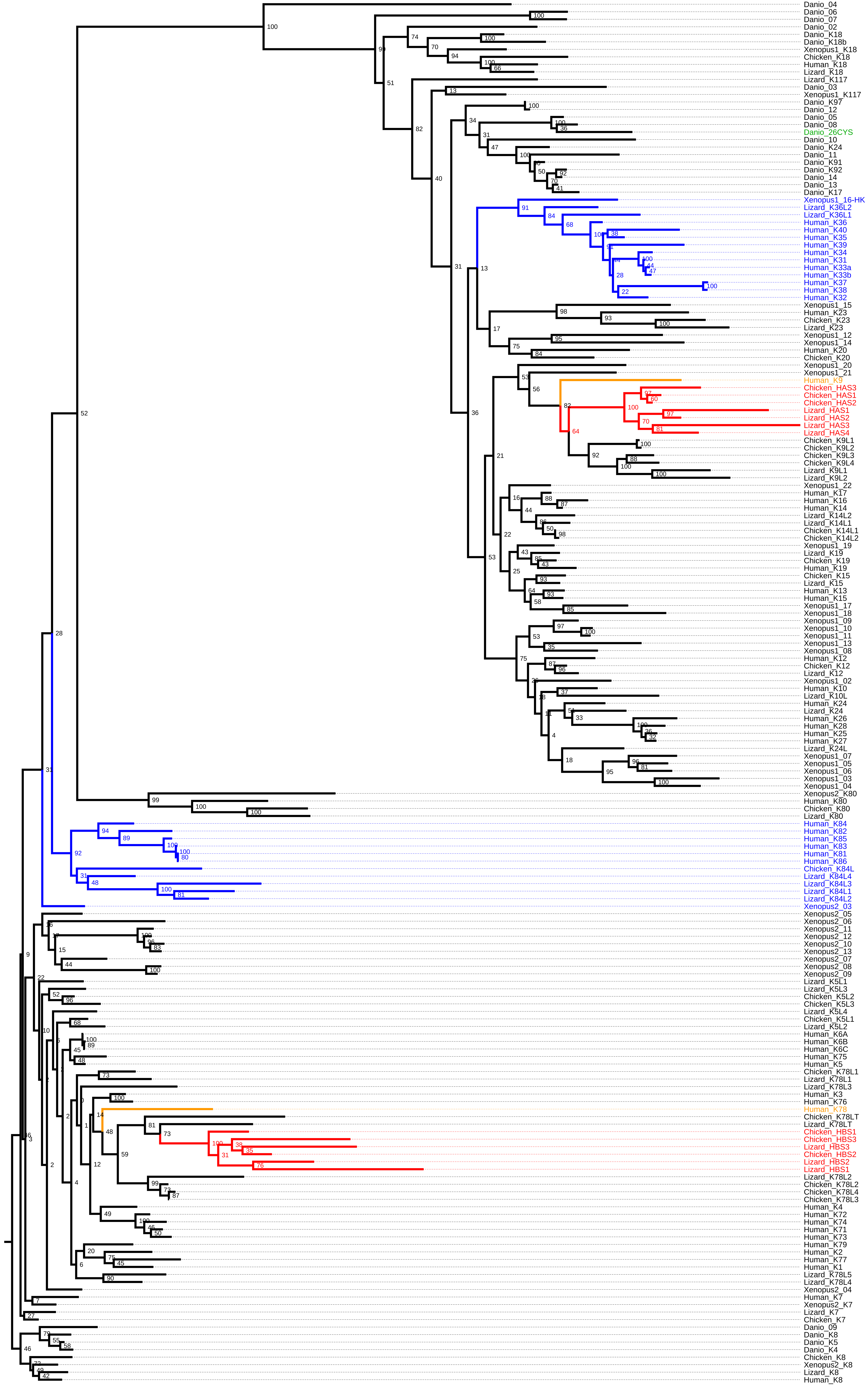

Suppl. Fig. S8. Maximum likelihood phylogenetic analysis of type I and type II keratins of fish (*Danio rerio*) and tetrapods (*Xenopus tropicalis*, lizard, chicken, human). The phylogenetic tree was generated with PhyML. Hair keratins and orthologs are indicated with blue fonts and lines. Sauropsid-specific cysteine-rich keratins (HAS and HBS) are shown with red fonts and lines. A fish keratin with 26 cysteine residues is highlighted by green fonts. Bootstrap values are indicated. Bayesian phylogenetitic inference yielded similar results. Accession numbers of *Danio* keratins are shown in the Suppl. Table S14.

A

```
        1                                                                                                  100
E1BZZ9  ----------------------------------------------------------------------------------------------------
  HAS1  MSCNIKETITVSSKGRSSGGSCIIGGGGGARISSYAIGSGRGFSGRSYCGGVNYGGGLSVGSLAGGSYGGGNCYGNGLGFGLGGGVVVGGLGSDCLLSSC

        101                                                                                                200
E1BZZ9  ----------------------------------------------------------------------------------------------------
  HAS1  DEKVTMQNLNDRLASYLDKVKCLEKENAELECRIREWYATQGLSCEPRDYSCYYKEIEDLQNQIVCATIDNNKIILDIDNSRMAADDFRVKYETELALRQ

        201                                                                                                300
E1BZZ9  --------------------------------------------MNCLRKQSTGDVSVEVNACPGPDLRQILEDLRCQYETLIARNRKEVEDWYECKIEE
  HAS1  SVEADINGLRQVLDQLTLCRSDLEAQLESLREELCCLKKNHEEEMNCLRKQSTGDVSVEVNACPGPDLRQILEDLRCQYETLIARNRKEVEDWYECKIEE

        301                                                                                                400
E1BZZ9  VNREVITSGQEVETCNNQVTELRRQLQALEIDLQAQLSQRNNLESSLAETECQYNTLLGELQNQITCVEQQLAEIRAEIECQNQEYKTLLDVKCRLEQEI
  HAS1  VNREVITSGQEVETCNNQVTELRRQLQALEIDLQAQLSQRNNLESSLAETECQYNTLLGELQNQITCVEQQLAEIRAEIECQNQEYKTLLDVKCRLEQEI

        401                                                              466
E1BZZ9  QTYRCLLEGGQQDLIHGGGIGVGSGVGGGVIRTSHTYTTTTASHCQPQVPPCKTGDIQVTCRRICD
  HAS1  QTYRCLLEGGQQDLIHGGGIGVGSGVGGGVIRTSHTYTTTTASHCQPQVPPCKTGDIQVTCRRICD
```

B

```
        1                                                                                                  100
E1C8E5  ----------------------------------------------------------------------------------------------------
  HAS2  MSSVSSGRYTTCGIGGSRGFSGRSYCGGVNYGGGLSSGSLVGGSYGGGLGAAVLGGCSGIGFSGGSARFGGGIGGGLGISLGGGVVGGGFSGDGILLSGD

        101                                                                                                200
E1C8E5  ----------------------------------------------------------------------------------------------------
  HAS2  EKVTMQNLNDRLASYLDKVRCLEQENADLECRIREWYAKQGPFCEPRDYSCYYKEIEDLQNQIVCATIDNNKIILNIDNSRMTADDFRVKYETELALRQS

        201                                                                                                300
E1C8E5  -------------------------------------------MNCLRKQSTGDVSVEVNACPGPDLRKILEEMRCQYETLIERNRKEVEDWYECKIEEV
  HAS2  VEADINGLRQVLDQLTLCRSDLEAQLESLREELCCLKKNHEEEMNCLRKQSTGDVSVEVNACPGPDLRKILEEMRCQYETLIERNRKEVEDWYECKIEEV

        301                                                                                                400
E1C8E5  NREVITSGQEVETCNNQVTELRRQLQALEIDLQAQLSQRDNLESSLAETECRYNNHLGELQTQITCVEQQLADLRAEMECQNQEYKILLDVK--------
  HAS2  NREVITSGQEVETCNNQVTELRRQLQALEIDLQAQLSQRDNLESSLAETECRYNNHLGELQTQITCVEQQLADLRAEMECQNQEYKILLDVKCRLEQEIH

        401                                                                  470
E1C8E5  ----------------------------------------------------------------------
  HAS2  TYRCLLEGGQQDLIQQGGIGQLSGLGGGVARTGGIGGGGIIRTSHTYTSSAQMPSCAAAEIQVPCRRICD
```

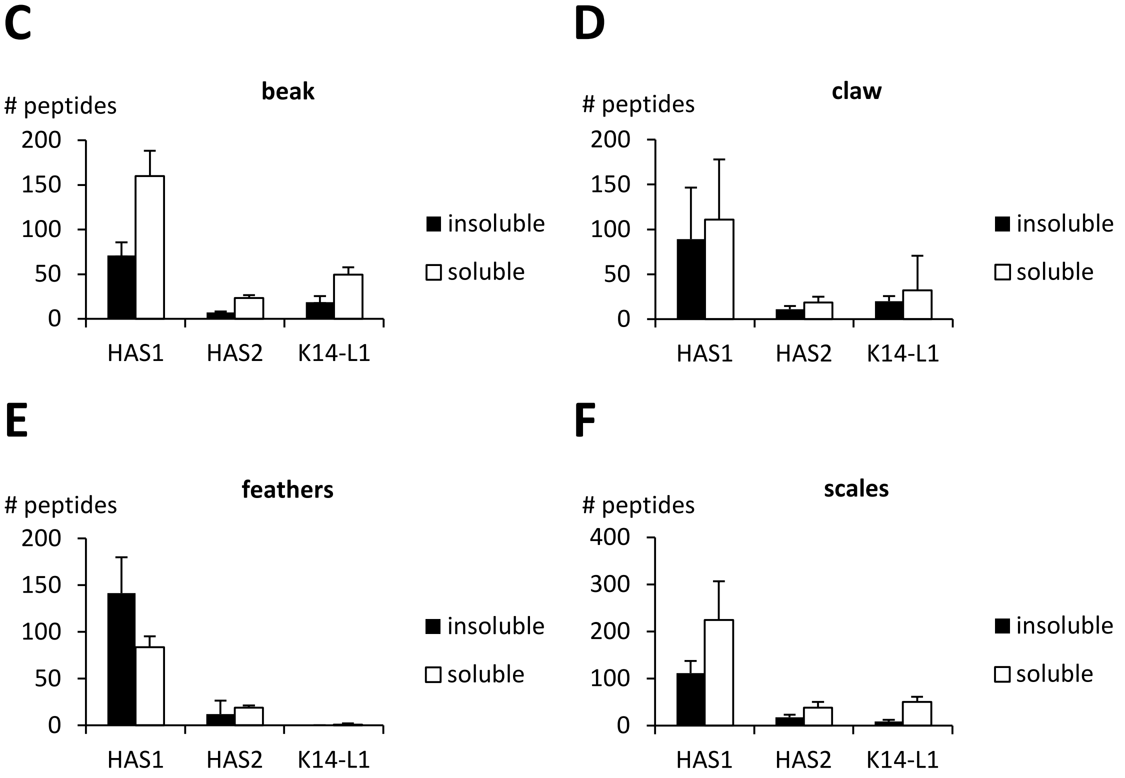

Suppl. Fig. S9. Detection of HAS1 and HAS2 keratin fragments in the proteomes of hard skin appendages of the chicken. Results of proteomic analysis of cornified chicken skin appendages (Rice et al., 2013) were re-analyzed using protein predictions as defined in the present study. Amino acid sequence alignments showed that E1BZZ9 (Uniprot accession number) and E1C8E5, that were detected by proteomics, corresponded to fragments of keratins HAS1 (**A**) and HAS2 (**B**), respectively. Red fonts mark identical residues. (**C-F**) Number of peptides detected by proteomics in cornified chicken skin appendages (soluble and insoluble fractions). Bars indicate means of n=4 samples. Error bars indicate standard deviations. Peptide counts of K14L1 (keratin 14-like 1) are shown for comparison.

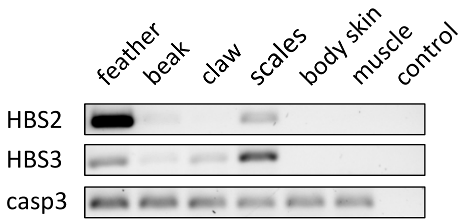

Suppl. Fig. S10. Keratins HBS2 and HBS3 are differentially expressed in hard skin appendages of the chicken. Gene expression was investigated by RT-PCR analysis on RNA extracted from chicken tissues. Caspase-3 (casp3) is ubiquitously expressed and was investigated for comparison. In a control reaction, no cDNA was added to the PCR. PCR products were run on an agarose gel and DNA was visualized by GelRed fluorescent dye.

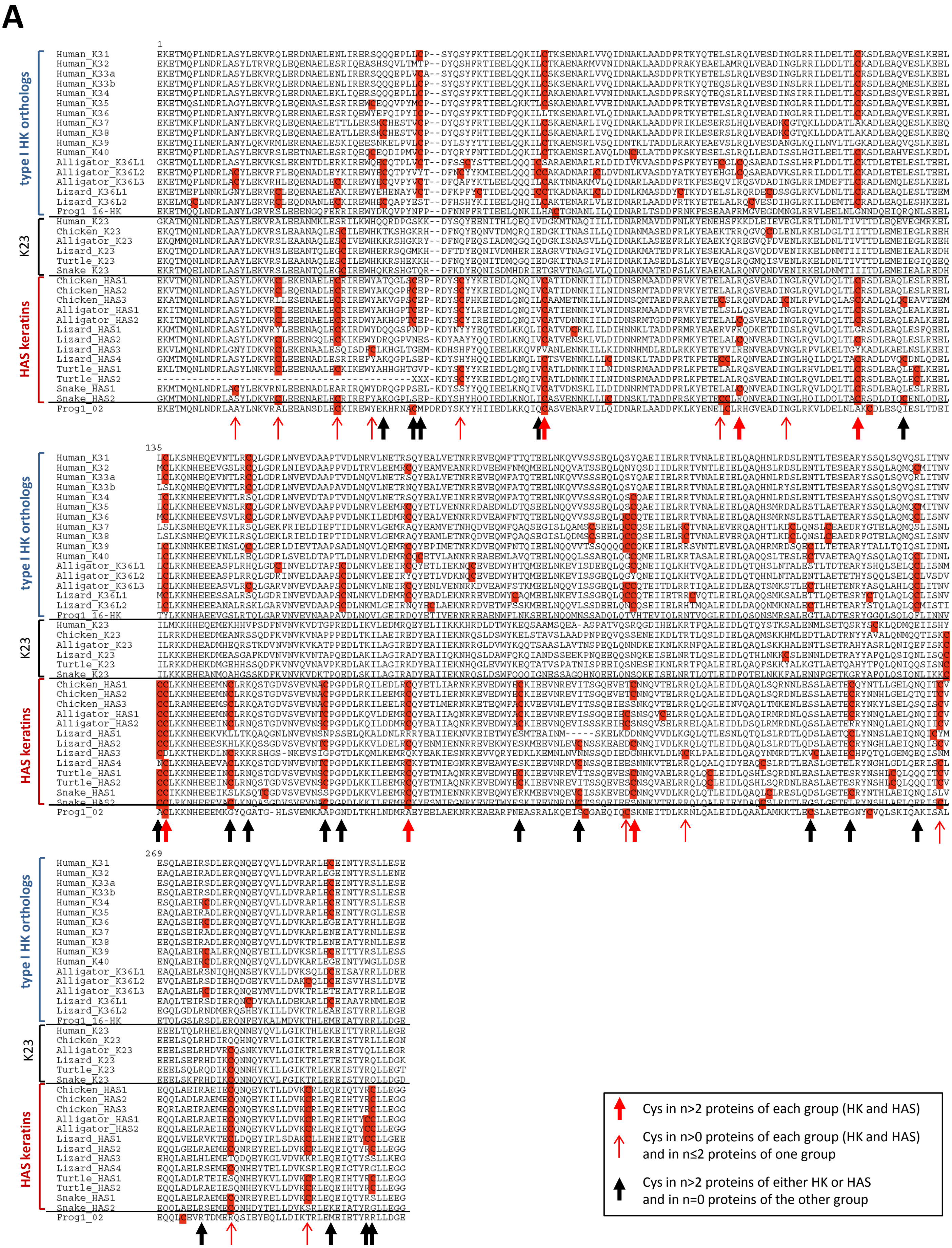

Suppl. Figure S11. Continued on the next page.

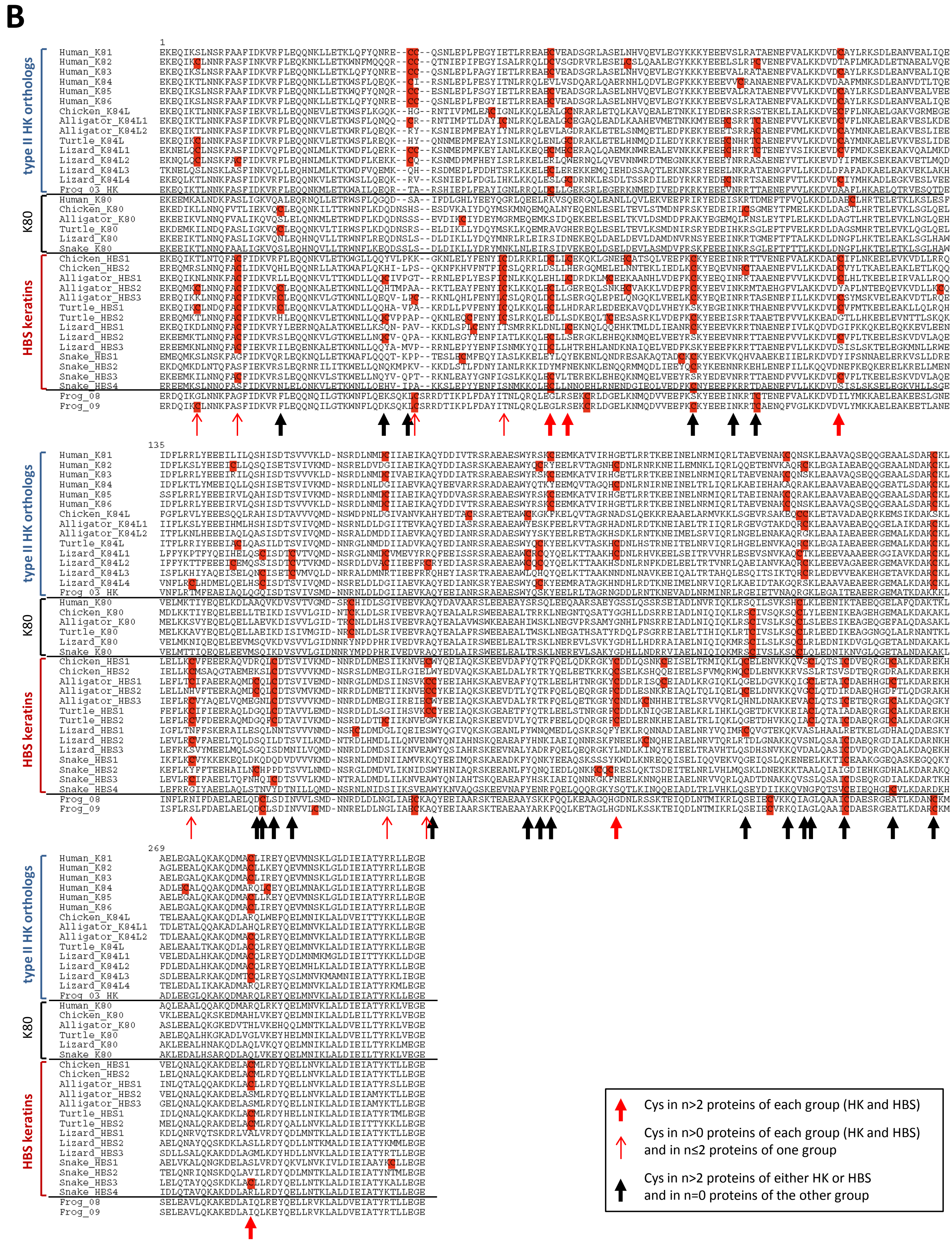

Supplementary Figure S11. Comparison of positions of cysteine residues in the intermediate filament (IF) domains of hair keratin (HK) orthologs, HAS and HBS keratins. The amino acid sequences of the IF domains of type I keratins (**A**) and type II keratins (**B**) were aligned with the Seaview program. Cysteine residues (C) are highlighted by red shading. The sequences of evolutionary ancient keratins with low to medium cysteine contents (K23 and K80) are included for comparison. Type I and type II frog (*X. tropicalis*) keratins with the highest cysteine contents are also included in the alignment. Vertical arrows indicate positions with cysteine in multiple proteins. Red arrows (definition in the boxed legends on the right) indicate likely convergent evolution towards cysteine at particular positions whereas black arrows indicate cysteine residues that are specific for either HAS or type I HK or HBS or type II HK (without consideration of cysteine in K23, K80 or frog keratins). Due to a sequence gap in the genome sequence, the sequence of turtle HAS2 is incomplete at the amino-terminus.

```
>Chicken_K12
KKETMQNLNDRLAAYLDKVRSLEDANTELERKIREWYEKNGPGAAVPGSGNDYSK--YYP
IIEDLRNKIINATIDNARIILQVDNARLAADDFRLKYENEVALRQSVEADINGLRRVLDE
LTLTRADLEMQIESLNEELAYLKKNHEEELQGLQSSAV-GQVSVEMDAAPGIDLTKLLND
MRGQYEVIAEQNRKEAEAWFNEKSGELKREISTNTEQLQSGKSEITDLKRTLQNLEIELQ
SQLAMKKSLEDTLAETEGGYCAQLSQMQLQIGNLESQLLQVRADMERQSAEYQQLLDIKS
RLEMEIETYRRLLDGE
>Chicken_K20
EKSTMQNLNDRLAAYLERVRSLEKANSLLEKQIKEWHEKNTV-----GVRSDYSS--YFQ
TIEDLQSKIGAAQLENAKLVLQIDNAKLAADDFRLKYENELLLRQSVESDINGLLRVLDD
LTLTKSDLESQIENVNEEIVFLKKNHEEEVDRLRKQVG-GSVNVEVDAAPGTDLASIMEN
MRRQYEEMAEKNRQEAKERFEKQTEALNQEVAINVEQLQEQRREVTDRRQICQSLELELQ
THLNMKKPLEDTLAETEARYSFQLAQIQETIAKLEAQLRQLRADMEAQNTEYSVLLDIKT
RLELEIATYRRLLEGE
>Chicken_K23
EKQTMQNLNDRLAAYLDKVRSLEAANAQLESCILEWHKTKSH-----GKRHDFNQ--YEQ
NVTDMQRQIEDGKITNASILLQIDNANMASEDFRLKYEAEKTRRQGVQCDLENLRKELDG
LTIITTDLEMEIEGLREEHILRRKDHEEDMEANRSSQD-FKVNVKVNAPPPEDLTKILAE
IREDYEAIIEKNRQSLDSWYKELSTAVSLAADPNPEQVQSSENEIKDLTRTLQSLDIELQ
AQMSKKHMLEDTLADTRNYYAVALQNMQQTISKCEEELSQLRHDIRQQHNQYRVLLGIKT
RLEKEISTYRLLLEGN
>Chicken_K15
EKITMQNLNDRLATYLQKVRALEDANAELEVKIRDWYQKEAPS----SPARDYSN--YYK
IIEDLQDKILAATIDNSRVVLEIDNARLAADDFRLKHENELFLRQSVESDINGLRRVLDE
LTLSRADLEMQIESLKEELAYLKKNHEEEMKEYSNQLS-GKVSVEMDAAPGIDLTRILSE
MREQYEALAEKYRKDAEAWFLTQTTELNHEVATHTEQIQSSKSEITELRRTMQSLEIELQ
SQISMKAGLEANLRDTEGRYCAQLAQIQNLITSVEEQLSEIRCDMERQNQEYRMLMDIKS
RLEQEIATYRQLLETQ
>Chicken_K19
EKTTMQNLNDRLASYLDKVRALEEANSDLETKIREWYLKQGP-----GPARDYSP--YYK
AIEDLRDQILAATIDNSKVVLQIDNARLAADDFKTKFETEQALRMSVEADINGLRRVLDE
LTLARTDLELQIENLKEELAYLKKNHEEEMSALGGQVA-GQVSVEVDSAPGIDLSKILAD
MRDQYEHMAEKNRKDAEAWFHSKTEELNREVAVNTEQLQSSKSEVTDLRRTLQGLEIELQ
SQLSMKGALESTLADTEGRYGAQLAQIQDMIGSIEAQLAELRADMERQNSEYKMLMDIKT
RLEQEIATYRQLLEGQ
>Chicken_K9L1
EKITMQNLNDRLASYLDKVRRLEEDNAQLELHIREWYRKQAP-----SVSKDYSS--YYQ
TIEQLQNQIISATVDNNRMLLDIDNSKMTADDFRMKYENELVIRQSVESDVNGLRNLLDD
LTHTRSSLESELESLKEELIALKRNHEEELRQLQSQTG-GDVSVEVNAAPGEDLTKILND
LRNEYEQIIEKNRREVEQWYEVKIEEVNRQVTSSSQDIQTSSHQLTELKREMQNLEIELQ
AQLSTKNSLENSLAETESRYGCLLQQIQGQINSVEEELANIRCEMEGQNQEYKMLLGIKT
RLEQEIAQYRALLNEG
>Chicken_K9L2
EKITMQNLNDRLASYLDKVRRLEEDNAQLELHIREWYRKQAP-----SVSKDYSS--YYQ
TIEQLQNQIISATVDNNRMLLDIDNSKMTADDFRMKYENELVIRQTVESDINGLRNMLDD
LTLVRSSLESELESLKDELIALKRNHEEELRQLQSQTG-GDVSVEVNAAPGEDLTKILND
LRNEYEQIIEKNRREVEQWYEVKIEEVNRQVTSSSQDIQTSSHQLTELKREMQNLEIELQ
AQLSTKDSLENSLAETESRYGCLLQQIQGQINSVEEELANIRCEMEGQSQEYKMLLGIKT
RLEQEIAQYRALLHEG
>Chicken_K9L3
EKLTMQNLNDRLASYMDKVRGLEEENAHLERLIREWYQKQGP-----TGSKDYSQ--YYR
TIEELQNQIVGANVDLNRMLLDIDNTRMTVDDFRLKYETEYTLHQSVASDINGLRPLLDQ
LTLSRSDLETQFESLKEELIYLKKNHEEEMRGLQTQSS-GDVNVEVNATPGTNLMEKLNE
MRCEYERLIENNRREVESWYETKMEEVNQQVHSSGQEIQSSNQQISELRREYQSLEIELQ
SQLSMVDSLQSNLEDTERRYNMQLQQIQGMIGPLEEELASIRCEMESQNEEYKMLLGIKT
RLEQEIAQYRALLEEG
>Chicken_K9L4
EKLTMQNLNDRLASYLDKVRSLERENARLEQLIREWYQKQGP-----TGRKDYSH--YYT
TIEDLQNQILTAAVETNKILLDLDNTRMTAEDFRTKYETEYGLRQNVEADINTLRPLLDS
LTLSRTDLEMQFESLKEEMINLKKDHEEDMKSLQTQSS-GDVNVEVNAAPGEDLLKKLND
MRQEYEQIIQKNREEVESWYESKMEEVSQQVHSSGQELESSNQQISVLRRDYQSLEIELQ
SELSRVGALQSNLEDTERRYNMQLQQIQGMIGPLEEELASIRCEMESQNEEYKMLLGIKT
RLEQEIAQYRALLNEG
>Chicken_HAS1
EKVTMQNLNDRLASYLDKVKCLEKENAELECRIREWYATQGLS----CEPRDYSC--YYK
EIEDLQNQIVCATIDNNKIILDIDNSRMAADDFRVKYETELALRQSVEADINGLRQVLDQ
LTLCRSDLEAQLESLREELCCLKKNHEEEMNCLRKQST-GDVSVEVNACPGPDLRQILED
LRCQYETLIARNRKEVEDWYECKIEEVNREVITSGQEVETCNNQVTELRRQLQALEIDLQ
AQLSQRNNLESSLAETECQYNTLLGELQNQITCVEQQLAEIRAEIECQNQEYKTLLDVKC
RLEQEIQTYRCLLEGG
>Chicken_HAS2
EKVTMQNLNDRLASYLDKVRCLEQENADLECRIREWYAKQGPF----CEPRDYSC--YYK
EIEDLQNQIVCATIDNNKIILNIDNSRMTADDFRVKYETELALRQSVEADINGLRQVLDQ
LTLCRSDLEAQLESLREELCCLKKNHEEEMNCLRKQST-GDVSVEVNACPGPDLRKILEE
MRCQYETLIERNRKEVEDWYECKIEEVNREVITSGQEVETCNNQVTELRRQLQALEIDLQ
AQLSQRDNLESSLAETECRYNNHLGELQTQITCVEQQLADLRAEMECQNQEYKILLDVKC
RLEQEIHTYRCLLEGG
>Chicken_HAS3
EKATMQNLNDRLASYLDKVRLLESENAELECRIREWYAKVGPS----CEPRDYSC--FHK
EIEDLQNQILCAAMETNKILLNIDNSQMTAEDFRAKYETECSLRQNVDADICNLRPVLDQ
LASCKADLQLQCEAVTEEMCCLRANHEEEMSCLRKQAT-GDVSVEVNACPGPDLRKILEE
LRCQYETLMERNRKETEQWFACKVEEVNLEVITSSQEIESSNKQVTELRRQLQGLEINVQ
AQLTMKENLESSLAETECRYNKYLSELQSQISCVEQRLAEIRAEMECQNQEYKSLLDVKC
RLEQEIHTYRCLLEGG
>Chicken_K14L1
EKETMQNLNDRLAAYLDKVRALEEANTDLEVKIREWYKKQGP-----GPERDYSP--YYR
TIEELRSKILAATVENANIVLQIDNARLAADDFRTKFETEQALRLSVEADINGLRRVLDE
LTLSRADLEMQIENLKEELAYLKKNHEEEMNALRGQVG-GEISVEMDAAPGIDLTKILAE
MREQYESLAEKNRRDAEQWFFSKTEELNREVAVNTEQLQSGKTEITELRRTIQSLEIDLQ
SQLSTKAALEGTLADTEARYGTQLAQLQALITSVEEQLAELRCDMERQNHEYRVLLDVKC
RLEQEIATYRRLLEGE
>Chicken_K14L2
EKETMQNLNDRLAAYLDKVRALEEANTDLEVKIREWYKKQGP-----GPERDYSP--YYR
TIEELRSKVLGATVDNANLLLQIDNARLTADDFRTKFETEQALRLSVEADINGLRRVLDE
LTLSRADLEMQIENLKEELAYLKKNHEEEMNALRGQVG-GEISVEMDAAPGIDLTKILAE
MREQYESLAEKNRRDAEQWFFSKTEELNREVAVNTEQLQSGKTEITELRRTIQSLEIDLQ
SQLSTKAALEGTLADTEARYGTQLAQLQALITSVEEQLAELRCDMERQNHEYRVLLDVKC
RLEQEIATYRRLLEGE
>Chicken_K18
EKETMQDLNERLATYLEKVRSLENNNRRLEAQIRESLAKRGP------STRDWGN--YWD
TIQQLRDKIYDSAVENARTVLQIDNARLAADDFRVKYEAELAIRTSVESDISGLRKVLDD
TNMARLQLEGEIEALREELIFMKKNHEEEVNALQGQAASAGLTVEVDAPQAQDLGQALAE
LRAQYDALAKKNVEELEKQWGQQISETTLEVTQSTKEVDTARSRLLELRRSVQSLEIDLD
ALRSQKRGLEGSLAETEARYGAQLEQLQGLILRGEEELAQARAELQRQNREHCALQDAKS
```

```
KLEAEIATYRALLEGG
>Alligator_K24
EKETMQNLNDRLANYLDKVHALEEANADLEYKIHQWYEKHGPAAA--ELTRDYSK--YYQ
IIEDLRNQIISATIGNASIVLQIDNSQLAAEDFRLKYENELFLHRSVEADSNGLRRVLDD
LTIAKSNLEAQLESLTEELVYLKKSHEEEMKSLKSTST-GDVNVEINAAPGIDLTKLLND
MRAQYESLAEQNRREAEEQFNKMSQPLQKQIYDDAGAVSSATNELTEMKRTIQGLEIELQ
TVMAQKCSLEGTLARTEENYSAQLSQRQLHISNLEEQLQQIRSEMECQNAEYEQLLGIKI
RLEMEIETYKRLLDGE
>Alligator_K10L
EKETMQNLNDRLASYLGKVQALEEANAELEHKIKDWYETLGSYTE--GGSRDYSK--YYT
TIEDLRNQIIAATVENASIILQTDNARLAVDDFKVKYENELYLRQNVEADIRGLRKVLDE
QTLAKSNLEIQIESLADEVAYLKKNHEEEMNDLRSSAS-PDVNVEINTAPGIDLTTRLNK
MRTEYEALAEQNRKDVETWFNEKSKELNVQINFSAEETNTNKSQITELKRTFQGLEIEMK
SQLALKQSLEATLAETEARYCAQLSQIQVVISSVETQIQQIRDDMEWQNAEYEQLLDIKI
HLENEIETYRHLIDRE
>Alligator_K12
KKETMQNLNDRLAAYLDKVRSLEEANTELEHKIREWYEKNGPGAGSLGAGNDYSK--YYP
IIEDLRNKIINATIDNARIVLQIDNARLAADDFRLKYENEVALRQSVEADINGLRRVLDE
LTLTRADLEMQIESLNEELAYLKKNHEEELQGFQGNAT-GQVSVEMDAAPGIDLTKLLND
MRGQYEAIAEQNRKDAEAWFNEKSGELKKEISTNTEQLQSGRSEITDLKRTLQSLEIELQ
SQLAMKKSLEDTLAETEAGYCTQLSQMQLQIGNLENQLFQVRADMERQNAEYQQLLDIKT
RLEMEIETYRRLLDGE
>Alligator_K20
EKLTMQNLNDRLASYLEKVHSLEKTNSQLEKQIREWYANNSS-----STRQDYSP--YYN
TIEELRNKIGAAQLENARLILQIDNAKLAADDFRMKYESELAIRQGAENDTAGLLKVIDD
LTLTKADLESQVEGMTEELAFLKKNHEEEVDRLRKQLG-GTVNVEVDAAPSIDLAKIMEN
MRQQYESMAQKNRQEAKDRFDRQTEELNQVVAVNTEQLNSKKTEITDLKRTFQSLEIELQ
SQLSMKKALEDTLAETEARYGSLLAQIQAAIGNVEGQLVQVRADMERQNNEYNILLDVKT
RLEMEIATYRRLLEGE
>Alligator_K23
EKQMMQNLNDRLAAYLDKVRSLEAANAQLESCIVEWHKKRSH-----GKRYDFNQ--FEQ
SIADMQGQIQDGKISNASVLLQIDNAKLASEEFRLKYEAEKYQREGVQFDVENLRKELDN
LTIVTTDLEMEIEGLREEHILRKKDHEADMHEQRSTKD-VNVNVKVKATPPEDLTKILAG
IREDYEAIIEKNRRGLDVWYKQQTSAASLAVTNSPEQLQNNDKEIKNLKRNFQALEIELQ
AQLSKKHALENTLSETRAHYASRLQNIQEFISKCEEELSELRHDVRCQSNKYKVLLGIKT
RLEREISTYQLLLEGR
>Alligator_K36L1
GKETMQLLNDRLASYLEKVKSLEKENTDLERKIREWQECQTP-----LVCTDFSS--CYS
TTEELQQQICSARAENARLRLDIDIVKVASDDFSFKYEYECGLCQSAEADISSLHRFLDD
LTLCKTDLETELESLTEELLCLRKNHEEEASPLRHQLG-DCINVELDTAPSCDLNKVLEE
IRCQYETLIEKNQCEVEDWYHTQMEELNKEVISDEEQLQGCQNEIIQLKHTVQALEIDLQ
TQHSLNTALESTLTDTEARYSHQLSELQCLISNMEAQLAELRSNIQHQNSEYKVLLDVKS
QLDCEISAYRRLLDEE
>Alligator_K36L2
EKETMQFLNDRLACYLEKVRFLEQENADLEYKIREWYECQTP-----YVYTDFNC--YYK
MIEELQQQICCAKADNARLCLDVDNLKVASDDYATKYEYEHGLCQSAEADVKSLRRILDD
LTLCKADLETELESLTEELLCLRKNHEEEASPLRRQLG-DRINVELDAAPSCDLNKVLEE
IRYQYETLVDKNQCEVEDWYHTQMEELNKEVISSGEQLQGYQNEIIQLKHTVQALEIDLQ
TQHNLNTALENTLAETETHYSQQLSELQCLVSDVEVQLAELRSDIEHQDGEYKVLLDAKC
QLDCEISVYHSLLDVE
>Alligator_K36L3
EKETMQFLNDRLACYLEKVRHLEQENADLECKIREWYECQVP-----YVCTDFQA--FYK
TLEELQQQILCAKTNNAKMVLQIDNARLAADDFRTKFESEQVIRQSVDADINGLRRIMDE
FTLCRADLEMQLESLKEELLCLKKNHEEEASVLRCQLG-ARVNVEVDAAPSCDLNKVLNE
IRCQYENLVENNRKDVEAWFSTKMEELNQQVISSGEQLQCCQTEITDLRRTIQALEIDLE
AQTSMKSTLECTLHETENRYASQLAHLQCLITNVEAQLAELRCDIERQNQEYMVLLDVKT
RLETEIATYRRLLEGE
>Alligator_K15
EKITMQNLNDRLASYLDKVRALEEANADLEVKIRDWYQKQGPT----SPARDYSP--YYK
TIEDLRDKILAATIDNSRIILEIDNARLAADDFRMKYENELYLRQSVEADINGLRRVLDE
LTLARADLEMQIENLKEELAYLKKNHEEEMREYSNQMT-GKVSVEMDAAPGVDLTKVLAE
MREQYEALADKNRREAEAWFFTKTEELNREVATSTEQIQTSKSEMTELRRTLQGLEIELQ
SQLSMKAGLEANLADTESRYCAQLAQLQAMITSIEEQLAELRCDMERQNQEYKMLLDIKT
RLEQEIATYRRLLDGQ
>Alligator_K19
EKITMQNLNDRLASYLDKVRALEEANSELEIKIRDWYQKQGP-----GPSRDYSP--YYK
TIEDLRDKILAATIDNSKIVLQIDNARLAADDFKTKFETEQGLRLSVEADINGLRRLLDE
LTLARTDLEMQIENLKEELAYLKKNHEEEMNAHAGQAG-GQVSVEVDSAPGIDLTKILAE
MRDQYEFMAEKNRKDAEGWFNSKTEELNQEVAVNTEQLQTSRTEITDLRRTLQGLEIELQ
SQLSMKAALEGTLADTENRYGAQLSQIQCLISNIEAQLGDLRAEMERQNSEYKMLMDIKT
RLEQEIATYHQLLEGQ
>Alligator_K9L1
EKTTMQNLNDRLATYLDKVRRLEEENADLEERIREWYRKQGP-----SISKDYSP--YYQ
TIEQLQNQIISATVDNNKLILDIDNSKMIADDFRMKYENELAIRQGVEADINGLRNFLDE
LVRVRSTLELEVETLKDELLTLKKNHEEEMRLLQSQTG-GDVNVEVNAAPGQDLTKILND
MRQEYEEIIAKNRREVEQWYEVKMEEVNQQVTSSGQEVQTSSHQLTELRRVFQSLEIELQ
TQLSMKASLENNLAETEARYGAQLQQIQAMINCVEEELAQLRCEMESQNQEYKMLLGIKT
RLEQEIAQYHGLLQEG
>Alligator_K9L2
EKMTMQNLNERLASYLDKVRHLEEENANLERLIREWYQNHGP-----QCGRDYSQ--YYG
PIEDLQNQIISATVDLNKVLLDIDNTRMTADDFRIKYETEYSLRQNVEGDINGLRPILDQ
LTLARSDLEAQYESLKEELISLKKNHEEEMRGLQSQSG-GDVNVEVNTTPDSNLMQKLNE
MRCEYEQIIENNRKEVERWYETKMEEVNQEVHTSGQEIQSSNHQVSELRRDYQTLEIELQ
SQLSMVQSLQSNLEDTERRYNIQLQQIQNTITTVEEELANIRCEIESQNYEYKMLLDIKC
HLEQEIAQYRALLEEG
>Alligator_K9L3
EKMTMQNLNERLASYLDKVRHLEEENANLERLIREWYQNHGP-----QCGRDYSQ--YYG
PIEDLQNQIISATVDLNKVLLDIDNTRMTADDFRIKYETEYSLRQNVEGDINGLRPILDQ
LTLARSDLEAQYESLKEELISLKKNHEEEMRGLQSQSG-GDVNVEVNTTPDSNLMQKLNE
MRCEYEQIIENNRKEVERWYETKMEEVNQEVHTSGQEIQSSNHQVSELRRDYQTLEIELQ
SQLSMVQSLQSNLEDTERRYNIQLQQIQNTITTVEEELANIRCEIESQNYEYKMLLDIKC
HLEQEIAQYRALLEEG
>Alligator_K9L4
EKMTMQNLNERLASYLDKVRRLEEENANLEQLIREWYQKQGP-----QGGRDYSQ--YYK
TIEDLQNQIISATVDLNKVLLDLDNTRMTVDDFRLKYETEYGLHQNVESDINGLRPMLDQ
LTLARSDLEAQYESLKEELISLKKNHEEEMKGLQSQSS-GDVSVEVNAAPGTNLTEKLNE
MRGEYERLIENNRREVESWFEAKMEEVNQQVHSSSQEMESSNQQVSELRREYQTLEIELQ
SLLSMLQSLQSNLEDTERRYNMQLQQIQAMIIPVEEELANIRCEMESQNEEYKMLLGIKT
RLEQEIAQYRALLEEG
>Alligator_K9L5
EKMTMQNLNERLASYLDKVRCLEEDNLQLERLIREWYQTHGP-----QGGKDYSH--YYG
PIEDLLNQIVLATVANNKIILDLDNARMTAEDFRMKYETEYSLRQSVEGDINGLRPVLDN
LTLAKSDLEMQFESLREELIQLKKNHQEEMSSLQSESG-GDVSVEVNAAPGEDLMGKLNE
MRKEYEYIIQRNREEVERWYESKMEEVRHEVHTSGQEIESGNQQVSELRREYQRLEIELQ
```

```
SQLSTLQSLQSNLEDTDYRYNMQLQQIQNMIIPVEEELASIRCEMESQNEEYKMLLGIKT
RLEQEICQYRELLDEG
>Alligator_HAS1
EKITMQNLNDRLASYLDKVRCLEEENAELECRIREWYAKHGPT----CEPKDYSC--YLR
EIEDLQNQIVCATIENNKIVLNIDNSRMAADDFRVKYETELSLLQSVEADINGLRQVLDQ
LTLCRSDLEAQLEALKEELCCLKKNHEEEMNCLRKQST-GDVSVEVNTCPGPDLKQVLDE
MRCQYEAIIAQNRKEVEDWYACKIEEVNREVISSGQEIECSNSQVCELRRQLQALEIDLQ
AQIRMRDNLQSSLAETECRYNNQLAELQNQIICVEQQLAELRAEIECQNQEYKVLLDVKC
RLEQEIHTYCCLLEGG
>Alligator_HAS2
EKITMQNLNDRLASYLDKVRCLEEENAELECRIRDWYAKHGPT----CEPKDYSC--YIR
EIEDLQNQIVCATVDNNKIILDIDNSRMTGDDFRMKYETELALCQSVEADINGLRQVLDQ
LTLCRSDLEAQLEALKEELCCLKKNHEEEINCLRNQST-GDVNVEVNSCPGPDLKQILDD
MRCQYEAIIAQNRKEVEDWYACKIEEVNQEVISSSKEIECSNSQVSELRRELQALEIDLD
SQLRMRDNLQSSLAETERRYNNQLAELQNQIICVEQQLAELRAEIECQNQEYKVLLDVKC
RLEQEIQTYCCLLEGG
>Alligator_K14L1
EKETMQNLNDRLAAYLDKVRALEEANSELEIKIRDWYKKQGP-----GPDRDYSP--YYR
TIEDLRNKILAATVDNANLILQIDNARLAADDFRTKFETEQALRMSVEADINGLRRVLDE
LTLARADLEMQIENLKEELAYLKKNHEEEMNALRGQVG-GEITVEMDAAPGVDLTKILTE
MREQYEHLADKNRREAEQWFFKQTEELNQEVAINTEQLQSSKSEITELRRTIQGLEIELQ
SQLSTKAALEGTLADTEARYGSQLAQIQGLITSVEEQLAELRCDMERQNHEYKILLDVKC
RLEQEIATYRRLLEGE
>Alligator_K14L2
EKETMQNLNDRLAAYLDKVRALEEANSELEIKIRDWYKKQGP-----GPDRDYSP--YYR
TIEDLRNKILSATVDNANLLLQIDNARLTADDFRTKFETEQALRMSVEGDINGLRRVLDE
LTLAKADLEMQLENLSEELAYLKKNHEEEMNALRSQVG-GEITVEMDAAPGVDLTKVLAE
MREQYEHLADKNRREAEQWFFKQTEELNKEVAINTEQLQSGKSEIMELRRTIQGLEIDLQ
SQLSMKAALEGTLADTEARYGSQLAQIQGLITSVEEQLAELRCDMERQNHEYKILLDVKC
RLEQEIATYRRLLEGE
>Alligator_K117
EKDTMQNLNDRLAAYLERVCSLEEANGQLEQRIQELQVKK-------TLARHYDPSSCFS
TIAELRSQIQDESVRNSQLTLQLDNAKLAANDFRMKYEAELATRLGVESDIKGLRKVLDG
LTEARASLQVQIESLKEELTYLQQNHGEEVAALQGHLG-GSVSVEVDASPGINLVKTLAE
IRDQYEDIIEKNQREAEAWHKQQCETFTQEVATSSEALQAARTKATELKRIVQSLEIEFQ
MLQSTKEALEGTLAETESHYGVEMAQLQNLVAGKEAELAQLRTDTQRQANEYQHLLDLKT
RLEVEIATYQHLLEGQ
>Alligator_K18
EKETMQDLNDRLASYLEKVRSLETANHNLEVQIREFLDKKGP------STQDWTH--YWD
IIEDLRNKIYEESVDNARIVLQIDNARLAADDFRVKYEGELAIRQSVEADIAGLRKVIDD
TNMSRLQLESEIENLKEELIYMKKNHEEEAKSLQAQVSGSGLTVEVDSPKTHDLGKIMTD
IRAQYEALAQKNREDLDKYWNQQITESTIEITQSTKDMDTAHGTLTELRRTYQTLEIELE
SLRNLKGSLEANLMEVENRYGMQLEHLNGLLLRTEAELMQVRTEVQRQAEEYQALLNIKV
KLEAEIATYRRLLEGG
>Turtle_K24
EKGTMQNLNDRLAKYLDKVRALEEANADLEHKIRQWYEKHDPAAA--GLRYDYSK--YYQ
IIEDLKNQIISATIDNAKLVLQIDNAKLAVDDFRLKYENELFLHQSIEADINGLRRVLDD
LTMTRSNLEAQLESLTEELVYLKKNHEGEMKSFQHGST-GEVNVEINATPGVDLTKLLNE
MRAQYEYLAEQNRREAEEQFNKMSQQLQQQISDDAGAADSARHEITEIKRTLQTLDIELQ
SLLAKKCSLEGTLAEIEGNYGCQLSQIQLQISNLEEQLKQIRSEMECQNAEYEQLLGIKT
RLEMEIETYRRLLNGE
>Turtle_K12
EKETMQNLNDRLAAYLNKVHSLEEGNTDLEHKIREWYEKNGPGAGGPGAGHDYSK--YYP
IIEDLRNKIINASIDNARIVLQIDNARLAADDFRLKYENEAALRQSVEADINGLRRVLDE
LTLTRADLEMQIESLNEELAYLKKNHEEELQGFQGNAT-GQVSVEMDAAPGIDLTKLLND
MRGQYEVIAEQNRKEAEAWFNEKSMELKREISTNTEQLQSGKSEITDLRRILQGLEIELQ
SQLAMKKSLEDTLAETEASYCAQLSQIQLQIGNLESQLFQVRADMERQNAEYQQLLDIKT
RLEMEIETYRRLLDGE
>Turtle_K20
EKLTMQNLNDRLASYLEKVHFLEKANSQLEKQIREWYANSSS-----TTRHDHSP--YFK
TIEDLQNKIGAAHLENARLLLQIDNAKLASDDFRMKYENELAIKQNVENDLAGLLRVLDD
LTLTRSDLALQIEGLNEELTFLKKNHEEEVGELRKQLG-GTVNVEVDAAPSIDLAKIMEN
MRQQYDSMAEKNRQEAKDRFDKQTEEVNQEVAINIEQLQTKKTEITDLRRTFQGLEIELQ
SQLSTKKALEDTLAEMEARYGSQLAQIQAAIGNVEAQLMQLRADMQSQSTEYSTLLDIKT
RLEMEIATYRRLLEGE
>Turtle_K23
EKQTMQNLNDRLAAYLEKVRSLEAANAQLESCILEWHKKRSH-----EKKHDFNQ--YEQ
NITDMQGQIEDGKITNASIFLHIDNAKIASEDLSLKYEVEQSLRQGMQISVENLRKELDN
LTIVTTDLEMEIEGIQEEQILRKKDHEKDMGEHHSSQD-FKVNVQVNAVPKEDLAKILAG
IRKDYEAIIEKNHQGLEVWYKEQTATVSPATNISPEEIQSNESEIKNLRRTFQALEIELQ
AQFSKKYALKGTLAETQAHYTFQLQNIQQSISNCEEELSQLRQDIKCQNNHYKILLGIKT
RLEKEISTYRQLLEGT
>Turtle_K15
EKVTMQNLNDRLASYLDKVRALEQANTDLEIKIRDWYQKQGPT----SPDRDYSP--YYK
IIDELRDKILAATIDNSRVILEIDNARLAADDFRLKYENELFLRQSVEADINGLRRVLDE
LTLSRADLEMQIENLKEELAFLKKNHEEEMKEYSNQMG-GKVSVEMDAAPGVDLTSILSE
MREQYEILADKNRRDAEAWFYKQTEELNREVATSTEQIHTSKSEITELRRTLQGLEIELQ
SQLSMKAGLEANLAETEGRYCAQLAQLQAMITSIEEQLAELRCDMERQNQEYRMLLDIKT
RLEQEIATYRRLLEGQ
>Turtle_K19
EKSTMQNLNDRLASYLDKVRALEEANSELEVKIRDWYQKQGP-----GPAHDYSL--YYK
TIEDLRDKILAATIDNSKIVLQIDNARLAADDFKTKYETEQALRMSVEADISGLRRVMDE
LTLARTDLEMQIESLKEELAYLKKNHEEEMKALGGQTG-GQVSVEVDSAPGIDLTKILAD
MRDQYEVMAEKNRKDAETWFNSKTEELNQEVAINTEQLQTSKTEITDLRRTLQGLEIELQ
SQLSMKAALEGTLADTENRYGAQLAQIQHLIGNVEAQLTDLRADMERQNSDYKQLMDIKT
RLEQEIATYRQLLEGQ
>Turtle_K9L
EKITMQNLNDRLASYLNKVRALEEENANLENLIKEWYLRKGPI----ATLKDYSR--YYE
EIEQLNNQIVNSSVEVNKVILNIDNTRMTIEDFKLKYETELGLRQNVEGDINGLRPLLDQ
LTLTRSDLELEFESLKEELISLKKNHEEELKGLQPQTS-GDVNVEVNATPGYDLTEILND
LRREYEHIIENNRKEVEKWYETKISEVNHEVHSSGRDIESSTKQVTELRREYQNVEIELQ
SLLSTIQSLQSSLADTEGRYNIQLNQIQTIIVSVEEELASIRCEIDNQNQEYRLLLGIKN
QLEQEIAQYRHLLDEG
>Turtle_HAS1
EKLTMQNLNDRLASYLNKVRCLEEENAALECKIKEWYAHHGHT----GVPKDYSC--YYK
EIEDLQNQIVCATLDNNKIILNIDNSRMAADDFRLKFETELALRQSVEADINGLRHVLDE
LTLCRSDLEAQLECLKEELCCLKKNHEEEINCLRNQST-GDVSVEVNSCPGPDLKKILEE
MRCQYETMIAQNRKEVEDWYECKIEEVNREVITSSQEVESCNNQVAELRRQLQCLEIDLQ
SHLSQRDNLEASLAETESRYNSHLCQLQQQITCVEQQLADLRTEIESQNHEYKVLLDVKC
RLEQEIHTYRCLLEGG
>Turtle_HAS2
-----------------------------------------------XXXKDYSC--YYK
EIEDLQNQIVCATLDNNKIILNIDNSRMAADDFRLKFETELALRQSVEADINGLRQVLDE
LTLCRSDLEAQLECLKEELCCLKKNHEEEINCLRNQST-GDVSVEVNSCPGPDLKKILEE
```

MRCQYETMIAQNRKEVEDWYECKIEEVNREVITSSQEVESCNSQVAELRRQLQCLEIDLQ
SHLSQRDNLEASLAETESRYNSHLCQLQQQITCVEQQLADLRAEIESQNHEYKVLLDVKC
RLEQEIHTYRCLLEGG
>Turtle_K14L1
EKETMQNLNDRLATYLDKVRALEEANSDLEIKIKEWYKKQGP-----SPDRDYSP--YYR
TIEDLRSKILAATIDNANIVLQIDNARLAADDFRTKFETELALRMSVEADINGLRRVLDE
LTLARADLEMQIENLKEELAYLKKNHEEEMNILRSQLG-GEITVEMDAAPGVDLTKILAD
MREQYESLAEKNRKEAEQWFFTKTEELNREVAMNTEQLQSGKSEITELRRTVQGLEIELQ
SQLSMKAALEGTLAETESRYGAQLAQLQGLITSVEEQLAELRCDMERQNHEYKILVDVKT
RLEQEIATYRRLLEGE
>Turtle_K14L2
EKETMQNLNDRLANYLDKVRALEEANSDLEIKIKEWYKKQGP-----GPDRDYSP--YYR
TIEDLRSKILAATVDNASLLLQIDNARLTADDFRTKFETEQALRMSVEADINGLRRVLDE
LTLARADLEMQIENLKEELAYLKKNHEEEMNILRSQLG-GDITVEMDAAPGVDLTKILAD
MREQYESLAEKNRKEAEQWFFTQTEQLNREVAMNTEQLQSGKSEITELRRTVQGLEIELQ
SQLSMKAALEGSLAETEARYGSQLAQLQGLITSVEEQLAELRCDMERQNHEYKILVDVKT
RLEQEIATYRRLLEGE
>Turtle_K117
EKDTMQNLNDRLAAYLETVRSLEEANGQLERQIREVYAKRAQ-----AGCQDLSG--YFS
TIAELRSQIQAASTSNAQLILQIDNATLAADDFRVKFESELAIRRGGESDVAGLRKVLDE
VTLSRASLQEELGSLQEELAQLQKNHGEEAAALQGRLG-GSVSVEVDSAPGVDLVKTLAE
IRDQYEDVIERNHREAAAWHKEQCETVAQEVATSAEAVQGARTKLTELRRVVQGLEVELQ
SLYSMKEALEGTLAETRAGSGAELEQLRECVARKEAELAQLRTDTQRQAEEHQQLLDLKT
RLEMEIATYRRLLEGD
>Turtle_K18
EKETMQDLNERLASYLDKVRRLETDNQKLETQIREFMDKKGP------SSRDWSH--YFD
IIEDLRKQILDSAVDNARIVLQIDNARLAADDFRVKFESELAIRQSVESDINGLRKVLDD
TNMSRLQLEGEIESLKEELIFMKKNHQDEVDSLQAQITNSGLTVEVDAAQPQDLGKVMAE
IRAQYDNLAQKNMADLDQQWNQMITQSTVEITQNTKEIEVARSSVSELRRTYQTLEIELE
SLRNLKASLEANLQDVEQRYAMQMEHLNGQLLRAEAELAQIRNDIQRQSEDYQALLNIKD
KLEAEIATYRRLLEGG
>Lizard_K24
EKETMQNLNNRLANYLEKVRSLEEANAELEVKIRHWYEKNGPGAP--GVVRDYTK--YHQ
IIEDLRNKIVNTTIDNANVVLQIDNARLAADDFRLKFENELFLRQSVESDINGLRRVLDE
LTMNRSDLEAQLENLTEELAYLKKNHEEEINMLKSNST-GDVTVEMNAAPGIDLTKLLND
MRAQYEDLAEQNRREAEEQFHKMSQPLQQQIYDDAGAVSSARNELIELKRSFQSLEIELQ
SLLAKKASLEGTLTETEANYSSQLHQLQLQVSNLEEQLQQIRSETECQNSEYQQLLGIKT
RLEMEIETYRRLLDGE
>Lizard_K24L
EKHTMQNLNDRLANYLGKVHALEEANAELELKIKDWYAKCSIP----DTSRDYTK--YFN
IIEDLRGKILRQTIENAGIVLQIDNARLAADDFKLKYENELFLHQNVENDINGLRRVLDD
LTMHRSDLELQIEGLNEELAYLKKNHEEELKSFRGASS-GDVSVEMDAAPGVDLTKLLNS
MRSQYEELAEKNRQEAEDQFNKQVGALRQEISANVDQLSTGKTEITDLRRTLQGLELELQ
AQLAMKQSLEGTLAETERNYCDQLAQIQSQIAGLEEQLLQLRADMENQNSDYQHLLGIKT
RLEQEIETYRRLLDGE
>Lizard_K10L
EKETMQNLNDRLASYLAKVHALEKANSELELKIKNWYEKNAFQAD--GGFRDYSK--YYA
SIDELRNKIVSASVNNSNIILQIDNARLAADDFRMKYENEVFFRQSIEADINGLHKVFDE
VILSKSNLELQIESLIEELAFLKKNHDEEIRGLRGPTS-GNVSVEMNAVPGPDLTILLNK
MREEYEALAEQNRQDAETWFVKKSNELNTTISMSAKETSTNKKEIAELRRTLQGLEIDLK
TQLALRQSLETTLAETEDGYCSQLLQMQGLINNTEAQVKQVRDDMESQNADYRQLLDIKI
HLENEIETYRHLIDSE
>Lizard_K12
EKETMQNLNDRLAAYLDRVHSLEEANTELERKIREWYEKNGPGTGALGAGRDYSK--YYP
VIEDLRNKIINATIENARIVLQIDNARLAADDFRLKFENEVALRQSVEADINGLRRVLDE
LTLTRADLEMQIETLNEELAYLKKNHEEELIGFQSNAT-GQVSVEMDAAPGVDLTKLLNE
MRAQYESIAEQHRKEAEAWFNEKSGELKREISTHSEQLQYGKSELTDLKRTLQSLEIELQ
SQLALKKSLEDTLAETEAGYCTQLAQMQLQIGNVESQLFQVRADMERQNAEYQNLMDIKT
RLEMEIETYRRLLDGE
>Lizard_K23
EKQTMQDLNDRLAAYLDKVHSLEEANTQLEGCIREWHERRSQ-----GMKHDFTD--YEQ
NIVDMHERIEAGRVTNAGIVLQIDNAKMATEDFRLKYESEKALRQSVQDDVENIRKELDN
MTIIITDLEMEIEALREDHILKKKEHEKDMEASGAFKD-VNVNVKVNTAPQEDLAKILAG
IRADYEAIIQKNHQSLDAWFQKQIANDSSEEKPNQEEQQSNQKEISELNRTLQSLEIDLQ
THLNKKCSLENNLDETRAYYAFQLQSIQNLISQCEEELSEFRHDIKCQNNQYKVLLGIKT
RLETEISTYRQLLDGK
>Lizard_K36L1
EKETMEFLNNRLGNYLERVRCLEQENAELEAKIREWYEHENA-----YVCTDFKP--FYC
TIDELQQQICCTKADNAKLCLDIDNLKMASDDYCTKYDYELSLRQRDECDSSGLRKVLDN
LTLCRADLEAQLESLTEEMMCLKKNHEEESSALRSQLG-DRINVEVDAAPSCNLNKVLDE
MRCQYEADLEKNRREVEDWYCAQMEELNKEVISSGEQLQCCQTEIIETRRCVQTLEIELQ
AQQSMKSALETTLQETESRYCTQLAQLQCLISNVEAQLTEIRSDIERQNCDYKALLDEKA
RLDCEIAAYRNMLEGE
>Lizard_K36L2
EKELMQCLNDRLANYLERVRCLEQDNANLECKIREWHECQAP-----YESTDFHS--HYK
IIEELQHQILCAKTENARLVLEIDNARLAADDFRTKYETELALRQCVESDIHGLRKILDE
MTLCRADLEAQLESHKEELLCLKKNHEEEANALRSKLG-ARVNVEVDAAPSCDLNKMLGE
IRNQYECLAEKNRRDVETWFSSKMEELNQQVLSSDEELQNCQSEIIELRHTMQALEIDLD
AQQNMKNALECTLHETEARYSTQLAQLQCMISTIEGQLGDLRNDMERQSHEYKILLDVKT
RLEAEISTYRRLLEGE
>Lizard_K15
EKVTMQNLNDRLATYLDKVRALEEANTELEVKIRDWHQKQAPK----SPSRDYSQ--YYK
IIEDLRDKILGTTIDNSRVILEIDNARLAADDFRLKYENELHLRQSVEADINGLRRVLDE
LTMSKSDLEMQIEGLKEDLAYLNKNHEELMKEFSTQLT-GKVSVEMDAAPGIDLTKILAD
MREQYEHLAEQNRKDAETWFFSKTEELNREVATNTEQIQTSKSEISELRRTIQGLEIELQ
SQLSMKAGLESNLAETEGRYCAQLAQIQNLITGVEEQLAELRNDMERQNQEYKMLMDIKT
RLEQEIATYRNLLEGQ
>Lizard_K19
EKITMQNLNDRLASYLDKVHALEESNADLEVKIREWYQKQGPT----SPARDYSH--YYK
TIEDLRDKILDATIDNNKIVLQIDNARLAADDFKTKFESEQALRMSVEGDINGLRRVLDE
LTLARSDLELQIETLKEELAYLKKNHEEELGALSGHLG-GQVSVEVDSAPGIDLTKILAE
MRDQYELLADKNRRDAEAWFTTKTEELSREVAVNTEQLQSSKTEITDLRRTLQGLEIELQ
SQLSMKAALEGTLADTENRYSAQLCQIQNLISNIEAQLVEVRSDMERQNSDYKMLMDIKS
RLEQEIQTYRQLLDGH
>Lizard_K9L1
EKSTMQNLNDRLAAYMNKVKYLEDENAQLEVLIREWYQKHGDV----GTVRDYTH--HYQ
EIDQLFNELVNETDDYNKILLNIDNTRMTAEDFQIKYQTEAGLRQNVEADINGLRPMLDQ
LTLARSDLEAQFESAKEELITLKKNHQEALRELQSQRGHGDINVEVNAAPGPDVKQRLDQ
LRAEYEAIIDNNRREVEQWFESKMEEVRQQVSSSGLEISSGNQQVTDLRRQYQTLEIELQ
SALSMIQSLQSNLEDTERRYNLQLQQIQSMIQPVEGELASLRGEIESQSQEYQSLLGIKT
RLEQEIAQYRQLLDEG
>Lizard_K9L2
EKSTMQNLNDRFASYLNKVKSLEAENNQLETLIREWYQKHGQT----SEPKDYSS--YYT
EIDQLVNELISASLESNKILLDVDNARMAAEDFKLKYETEYGLRQNVDADLQGLRPLLDK

```
LTMDKSDLEMQYESLHEELVHLKKNHDEVMKSQHHQSG-GDVNVEVNSSPGQDLTKTLND
LRQEYEEIIMKNRKEVQQWFEAKMEEARQQQSPESQEGGSGRHQITELTREYQTLEIELQ
TQLSTIQALQNNLNNTEGGYNMQLQQLASQIEPVEAELAGIKGEIQNQTQEYQTLLGIKT
YLEQEINQYRQLLEEG
>Lizard_HAS1
KKMTMQNLNDRLASYLDNVRYLEEENAQLECKIRDWYDQQGSP----NDPKDYNY--YYE
QTEDLKKQLICATVDCRKLILDIHNNKLTADDFRMRYEAERVFRQDKETDIDSLRQVLDQ
LTLGRSDLEAQLESFQEELCCLKKNHEEKVKLLTKQAQ-GNLNVEVNSNPSSELQKALDN
LRRRYEAIIEKNVKEIETWYESMTEAINM-----SKELKDDNNQVVDLKGQLQTLESNLQ
TQLSLRDTLQDSLAETQCLYNNSLAEIQNQICYMEQQLVELRVKTECLDQEYIRLSDAKC
LLEHEIETYCCLLGEE
>Lizard_HAS2
EKMTMQNLNDRLASYLDKVRCLEEENGQLECKIKEWYDRQGPV----NESKDYAA--YYQ
QIEDLKNQIVCATVENSKLVLDIDNNRMTADDFRMKYETELALCQNVEADINGLRQVLDQ
LTLCRSDLEAQLESLREELCCLKKNHEEESKHLKKQSS-GDVSVEVNTCPGPDLKKVLDE
LRCQYENMIENNRREVEKWYESKMEEVNLEVCNSSKEAEDCNNQIVDLKRQLQTLEIDLQ
AQLSLRDTLQDSLAETECRYNGHLAEIQNQISCVEQQLGELRAEMECQNREYRELLDVKC
RLEQEIQTYRCLLEGG
>Lizard_HAS3
EKVLMQNLNDRLGNYIDKVRCLEKENAALESQISDFCLKHGLT----GEMKDHSH--FYQ
QIEELKKQVFVANLENNKILLKIDNNHMDLEDLRKKYETEYVIRENVEADVNGLRPVLKE
LTGYKADLKAELNSLKEEICDLKKTHEKDLNCRKKRSH-GS-NKEVSIGPGTDLKQMLKE
MRQKYEAKIESNRKEVVQRYEDKLKEMNLNVNNSSQDIEDGNHKVLDLKCKLPALEIDLQ
AQYNMRDTLKVCLAEIECHFQTQLGEMQEQISNMEHQLAELHLEMETQDQEYKGLVDVKK
RLEQEIQTYSSLLKEG
>Lizard_HAS4
GKMTMQNLNDRLASYLDKVRCLEEENADLESRIREFYAKQGPL----SEPKDYSH--YHQ
QIEDLKNQLICTSVENNKLLLCIDNSKLTADDFRTKFETECCLRQNVEADINGLHQILDQ
LTACRADLDVQCDNLQDELNCLKKNHAEEVACLKKQST-GDVNVEVNTCPGPDLKKILEE
MRCKYETMIEGNRKEVEAWYESKIEEVNRDVCNSSQEIEESNNKVTELRRQLQALQIDYE
AQCSLRDTLEASLGETELRYNGHLGDLQERISCLEQQLAELRSEMECQNHEYTELLDVKS
RLEQEIGTYRGLLEGG
>Lizard_K14L1
EKETMQNLNDRLASYLDKVRALEEANTDLEVKIKEWYKKQGP-----SPERDYSP--YFR
TIEELRSKILAATVDNASIVLQIDNARLAADDFRNKFETEHALRVNVEADINGLRRVLDE
LTLARADLEMQIENFKEELVYLRKNHEEEMSIFRSQSG-GEISVEMDAAPGVDLTKILSE
MREQYESLAEKNRKDAEQWFFSKTEELTREVAINTEQLQSGKTEITELRRTYQNLEIELN
AQLSMKAALESSLADTEARYGAQLSQIQVLITNVEEQLADLRCEMERQNHEYKMLLDVKT
RLEQEIATYRRLLEGE
>Lizard_K14L2
EKETMQNLNDRLATYLDKVRALEHANTELEVKIREWYQKRGP-----GPQRDYSS--YFR
TIEDLRSKILAASVQNATILLQLDNAKLTADDFRTKYETEHALRTSVEADIHGLRKVMDE
LTLTRSDLEMQLENLKEELAYLHKNHEEELKVLRSQMG-GEITVEMDAAPGVDLTKILAE
MREQYEHLAEKNRKDAEQWFFSKTEELNQEVAINTEQLQSGKSEVIELRRTLQGLEIELQ
TQLSMKSSLEGTLAETESRYGLQLNQLQALITSVEEQLAELRCDMERQNSEYKILLDVKT
RLEQEISTYRRLLEGE
>Lizard_K117
QKGTMQNLNDRLAAYLGRVHSLEESNAQLEQNIREYYAKRAS-----AGGPDLSG--YFN
TLSELKAKIEQETLNNAGLLLRIDNAKLAADDFKVKLESEVAIRLSLEGDVNGLRKVLDD
VNASRASLQVQVDNLQEELVYLKRNHEEEVASLQGRLG-GTVNVEVDSMPGVDLQKVLAE
IRDQYEDVMEKNRQEAEALHKAQCDAINQQVADSSEALQAAQLKITELRRLAQALEIELQ
SLRSTKIALEGTLVETESRYGMELSRLRELISARESELFQLRSEAQRQAEDYKRLMDIKN
KLEQEIATYRRLLEGL
>Lizard_K18
EKETMQDLNDRLASYLEKVRSLEADNRKLEIQIREHLDKKGP------SARDWSH--YFE
LIEDLRNQIFDQTIDNSRIVLQIDNARLAADDFRVKFEAELAIRQSVENDINGLRRVIDD
TNMMRLQLESEIEALKEELVFMKKNHQDEVNNLQAQIANSGLTVEVDAPKTQDLGKIMAE
IRAQYDVLAQKNLEDLDKYWSQQITESTVVITEHSKDIEKARANITDLRRSVQTHEIELE
SARNLRASLEANLHEVEMRYGLQMEHLNGIILRTEADLAQLRNDVQRQAEEYQILLNIKD
KLEAEITTYRHLLEGG
>Snake_K24
EKETMQNLNSRLANYLEKVRSLEEANAELERKIRHWYEKNAPGAP--GVTRDYSK--YHR
IIDELRNQILSMTVENANVVLQIDNARLAADDFRMKFENELFLRQSVEGDINGLRRVLDD
LTMNRSDLEAQLESFTEELAYLKKNHEEEMTLLRSSST-GDVTVEMNAAPGIDLTKLLND
MRAQYEDLAEQNRREAEEQFIRMSQPLQQQIHDDAGAASSARNELIELKRSLQSLEIELQ
SLLAKKASLEGTLSETEASYSTQLSQLQMQVSSLEEQLQQIRAETECQNSEYQQLLGIKT
RLEMEIETYRRLLDGE
>Snake_K24L
EKQTMQNLNDRLANYLGKVHALEEANSELELRIKEWYGKFSAT----DTDRDYSK--YFK
IIEDLRNQICRYTIENAGIVLQVDNARLAADDFKLKYENELFLHQSVENDINGLRRVLDD
LALNKADLELQVESLTEELVYLKKNHEEELMSFRGVAS-GDVTVEMDAAPGVDLTKLLND
MRSQYEELADKNRREAEEQFNKQVGALRQQISANVDQLSSGKSEITELRRTMQGLELELQ
AQMAMKQSLEGTLAETERNYCDQLGQIQVQISSFEEQLLQIRSDMENQNAEYQQLLGIKT
RLEKEIETYHRLLDGE
>Snake_K10L1
EKETMQNLNDRLASYLAKVHALEEANGDLEQKIRNWYQKSATH----INEGLYNDSKYNS
SIEDLRNKIITASIRNAGTILQIDNARLAADDFRMKYENELFLHQNVEADINGLKKLLDE
LSMSKSDLEIQIKSLTEELTFLKKNHEEEMKNWQGTVA-GDINVEINAAPGVDLTAVLNK
MRTEYEALAEQNRKDAETWFIEKSKELNTTINVSAKETTTNKKQIADLRHILQSLEIDMK
TQLSLRQSLERTLAETEGQYCAQLLKIQGKISSVENQVQQIREDMECQNADYRHLLDIKI
RLENEIETYRRLIDGE
>Snake_K10L2
-XXXMQNLNDRLATYLGNVQALEEANSELEGKIAEWYEMFGAGNQA-GQQQDYSE--YYA
IIDDLRNQIIATSIDNGTLILQIDNARLAGADFKLKYDNEVAIHQSVEMDANGLRKALEE
IEITSSDLKVQINSFTEELEQMKKNHEEDMVSMNDSTAAGDINVEMNVTPGVDLTTLLNQ
MRGEYEALAEQNREDMEAWFHEQSQELNRQINTSVEETSTNKSEITEMKRALQGLEIELQ
SQLSLKQSLEATLAETEGQYCAQLSQIQEVIGSVEGQVQQIRDDMECQNREYEQLLDVKI
RLENEIDTYRSLLDGS
>Snake_K20
-XXXMQNLNDRLAAYLEKVRSLETSNSVFEKQIREWYEKSSP-----ITKQDYSA--YFK
VIRELQDKITEAREDNTRIILQIDNSKLAADDFRVKYETELGLRLSVEYDITGLQGLIDD
LTLVRTDLEQQIESLKEELAYLRKQHDEETARLRGQLS-GNVSVEVDAAPGIDLAQMMEN
MRQQYEILAEKNRQEAKDRFDKLIEQLNEEVTTTTYQLETHRSEITDRRNTLQSLEIELR
SQLSWKTERENALAEIEAHYRTILTQIQMAIGNIEAQLMQLRSDMEQQSMEYSILLDIKV
KLEAEISTYRRLLEGE
>Snake_K23
EKRTMQELNDRLASYLEKVRSLEEANTQLEGCIREWHQKRSH-----GTQRDFKD--YEQ
NISDMHDRIETGRVTNAGLVLQIDNAKMATEDFSLKYEAEKALRQNVQNDVENIRKELDN
MTIIITDLEMEIEALREDHILKKKEHEANMQAHGSSKD-FKVNVKVDKTPQEDLSKILAG
IRADYEAIIEKNRQSLDSWFQQQIGNESSAGQHNQEELQNSQKEISELNRTLQTLEIDFQ
TELNKKFALENNFGETKGRYAFQLQSIQNLIKKCEEELSKFRHDIKCQNNQYKVLFGIKT
RLEREISTYRQLLDGD
>Snake_K15
EKITMQNLNDRLANYLDKVRALEEANTELEIKIRDWHQKQAPT----SQAKDYSN--YYK
```

```
IISDLRNKIGDCTCDNSKVVLEIDNARLAADDFRLKYENELHLRQGVESDINGLRRVMDE
LTLFKSDMEMQIEEKKEDLAYLKKNHEELMKEYSTQLT-GQVNVEMDAAPGVDLTKILAD
MREQYEQMAERNRRDAEAWFFTKTEELNREVATHTEQLQTSKTEISELRRTIQSLEIELQ
SHLSMKAGLESNLADTEGRYCAQLAQIQNLITGVEEQLAELRNDMERQNQEYRMLMDIKT
RLEQEIATYRNLLEGQ
>Snake_K19
EKVTMQNLNDRLANYLDKVRALEEANTELEVKIREWYQKQGP-----TASRDFSL--YFK
TIEDLREKIKDATIGNNSIILQIDNARLAADDFRTKFETEQALRISVETDINGLRRVLDE
LTLSRADLEMQIENLKEELAYLKKNHEEEMSTLSGQLG-GQVSVEVDSAPGIDLTKILAD
MRDQYELLAEKNRRDAEAWFTSKTEELNKEVAVNTEQLQTSKTEITDLRRTLQSLEIELQ
SQLSMKSALEGTLADTEARYGNRLCQIQGLISNIEAQLVEVRSDMERQNNDYKMLMDIKT
RLEQEIETYRQLLDGQ
>Snake_K9L
EKATMQNLNDRLANYLDQVKRLEDENCQLEIWIKEWYQKHGDI----GPDKDYSC--YYQ
EIDQLYNELIAETDDYNKIILNIDNTRMTAEDFKLKYNTEAGLCKNVEADINGLRPLLDQ
LTLAKSDLEMQFESLREELICLKKNHEETLRGLRGQRGHGDVSVEVNAAPGQDLKQRLDQ
LRCEYENIIEQNRKEVEVWYETKMEEVRQQVSTSDQEITSSNHQLSELRREYQTLEIELQ
SQISMIQSLQTNLEDTERRYNMQLQQIQAMIEPVEGELASIRCEIESQNQEYQLLLGIKT
RLEQEICQYRRLLEEG
>Snake_HAS1
EKMTMQNLNDRLACYLEKVRNLEEENADLEARIRQWYDRQGPP----SELKDYSS--YYQ
QIEELKNQIVCATVDNSKLILDIDNNRMTADDFRLKYETELALCQNVEADINGLRQVLDQ
LTLCRSDLEAQLESLREELCCIKKNHEEEIKSLKSQTC-GDVSVEVNSSPGPDLKKVLEE
MRCQYETMIENNRKEVEQWYERKMEEVNQEVCISSKEVEDCNNQVVDLKRQLQTLEIDLQ
AQLCLRESLQDSLGETECRYNTHLAEIQNQISLVEQQLAELRAEMECQNREYRELLDVKC
RLEQEIQTYRSLLEEG
>Snake_HAS2
GKMTMQNLNDRLASYLDKVRCLEEENAELECRIREFYAKQGPL----SEPKDYSH--YHQ
QIEDLKNQLICASVENNKLLLCIDNSKLTADDFRSKYETECCLRQNVEADINGLHQILDQ
LTACRSDLDIQCENLQDELCCLRKNHEEEVACLKNQAS-GDVSVEVNACPGPDLKKILEE
MRCKYESMIEGNRKEVETWYESKIEEVNRDVCTSSQEIEESNNKVTELRRQLQALEIEYD
AQCSLRDTLEGSLGETELRYNSHLAELQERISCLEQQLAELRSEMECQNHDYTELLDVKS
RLEKEIATYRGLLEGG
>Snake_K14L1
EKQTMQNLNDRLANYLDKVRALEEANTSLEIKINEWYQKQGP-----APDRDYSS--YFR
KIEDLKNQILAASIDNASVLLQIDNARLAADDFRTKFETEQALRLNVESDINGLRRVLDE
LTLSRSDLEMQIENLKEELAYLKKNHEEEMNAFRKQVS-GEITVEMDATPGVDLTKILTD
MREQYETLAEKNRKDAEQWFFTKTEDLNREVATNTVQLQTSKTEIVELRRTIQSLEIDLQ
AQLSMKSALEGTLVETETRYGTQLGQIQGMITNVEEELAALRCDMERQNHEYKILLDVKT
RLEQEIATYRRLLEGE
>Snake_K14L2
EKETMQNLNDRLAAYLDKVRALEESNASLEVRIKEWYQKQAP-----GPDRDYSY--YFR
IIEDLRSKIVSATVENADVLLQIDNAKLTADDFKNKFEIEQALRMSIESDINGLRNVLDD
LTSTRIHLEKNLELLTEELLHLKKNHKEEMGLLQSQMG-GEITVEMDAAPGVDLTKILAD
MREQYENLAEKNRRDAEQWFFSKTEELNREVAINTEQLQSGQSEVIELRRTLQGLEIDLQ
AQLSTKAALEGTLADIESRYSLQLAHIQGLIINVEEELATLRCDMERQNHEYKILLDVKT
RLEQEIAMYRHLLEGE
>Snake_K117
EKGTMKNLNDRLAAYLDKVLSLEKSNMQLEQNIQEFYTKQTS-----TGTHDMSA--YFN
AMIQMKSEIQKETLKNAGLLLQIDNARLAADDFRVKLESEVAMRLSLEGDLGGLRKALEE
LNASRASLQVQADNLQEELACLKRNHKEEVASLQGHLG-GTVNVEVDSMPGTDLQKVLGE
VRDQYEGVMEKNRQEAEALHQSQCDALNQEVSVSTEALQSAQMKMVELKRLAQALEIELQ
SLWSMKEALEGTLAEVESHYGLELSQLQNLVAAREAELLQLKSDAQNQAEDYKRLMDIKN
RLEQEIATYRCLLEGS
>Snake_K18
EKETMQDLNDRLANYLDKVRSLEADNRKLEIQIREYMEKKGP------SARDWGR--HFQ
IIDGLKNQIYDQSVDNARVVLQIDNARLAADDFRVKYEAELAIRQSVESDIFGLRKVIDD
TNMSRLQLESEIEALKEELIFMKKNHQDEVTTLQAQIANSGLTVEVDAPKSQDLAKIMAE
IRAQYDALAQKNLEDLDKYWSQQISESTTVINQNSKEIETARVTMTDLRRTVQTLEIELE
SMRNVRASLEGNLLEVESRYAMQIEHLNGILLRTEAELSQLRNDVQRQADDYQALLNIKD
KLEAEIATYRHLLEGG
>Human_K9
EKSTMQELNSRLASYLDKVQALEEANNDLENKIQDWYDKKGPA----AIQKNYSP--YYN
TIDDLKDQIVDLTVGNNKTLLDIDNTRMTLDDFRIKFEMEQNLRQGVDADINGLRQVLDN
LTMEKSDLEMQYETLQEELMALKKNHKEEMSQLTGQNS-GDVNVEINVAPGKDLTKTLND
MRQEYEQLIAKNRKDIENQYETQITQIEHEVSSSGQEVQSSAKEVTQLRHGVQELEIELQ
SQLSKKAALEKSLEDTKNRYCGQLQMIQEQISNLEAQITDVRQEIECQNQEYSLLLSIKM
RLEKEIETYHNLLEGG
>Human_K10
EKVTMQNLNDRLASYLDKVRALEESNYELEGKIKEWYEKHGNSHQ--GEPRDYSK--YYK
TIDDLKNQILNLTTDNANILLQIDNARLAADDFRLKYENEVALRQSVEADINGLRRVLDE
LTLTKADLEMQIESLTEELAYLKKNHEEEMKDLRNVST-GDVNVEMNAAPGVDLTQLLNN
MRSQYEQLAEQNRKDAEAWFNEKSKELTTEIDNNIEQISSYKSEITELRRNVQALEIELQ
SQLALKQSLEASLAETEGRYCVQLSQIQAQISALEEQLQQIRAETECQNTEYQQLLDIKI
RLENEIQTYRSLLEGE
>Human_K12
EKETMQNLNDRLASYLDKVRALEEANTELENKIREWYETRGTGTAD-ASQSDYSK--YYP
LIEDLRNKIISASIGNAQLLLQIDNARLAAEDFRMKYENELALRQGVEADINGLRRVLDE
LTLTRTDLEMQIESLNEELAYMKKNHEDELQSFRVGGP-GEVSVEMDAAPGVDLTRLLND
MRAQYETIAEQNRKDAEAWFIEKSGELRKEISTNTEQLQSSKSEVTDLRRAFQNLEIELQ
SQLAMKKSLEDSLAEAEGDYCAQLSQVQQLISNLEAQLLQVRADAERQNVDHQRLLNVKA
RLELEIETYRRLLDGE
>Human_K13
EKITMQNLNDRLASYLEKVRALEEANADLEVKIRDWHLKQSPA----SPERDYSP--YYK
TIEELRDKILTATIENNRVILEIDNARLAADDFRLKYENELALRQSVEADINGLRRVLDE
LTLSKTDLEMQIESLNEELAYMKKNHEEEMKEFSNQVV-GQVNVEMDATPGIDLTRVLAE
MREQYEAMAERNRRDAEEWFHAKSAELNKEVSTNTAMIQTSKTEITELRRTLQGLEIELQ
SQLSMKAGLENTVAETECRYALQLQQIQGLISSIEAQLSELRSEMECQNQEYKMLLDIKT
RLEQEIATYRSLLEGQ
>Human_K14
EKVTMQNLNDRLASYLDKVRALEEANADLEVKIRDWYQRQRP-----AEIKDYSP--YFK
TIEDLRNKILTATVDNANVLLQIDNARLAADDFRTKYETELNLRMSVEADINGLRRVLDE
LTLARADLEMQIESLKEELAYLKKNHEEEMNALRGQVG-GDVNVEMDAAPGVDLSRILNE
MRDQYEKMAEKNRKDAEEWFFTKTEELNREVATNSELVQSGKSEISELRRTMQNLEIELQ
SQLSMKASLENSLEETKGRYCMQLAQIQEMIGSVEEQLAQLRCEMEQQNQEYKILLDVKT
RLEQEIATYRRLLEGE
>Human_K15
EKITMQNLNDRLASYLDKVRALEEANADLEVKIHDWYQKQTPT----SPECDYSQ--YFK
TIEELRDKIMATTIDNSRVILEIDNARLAADDFRLKYENELALRQGVEADINGLRRVLDE
LTLARTDLEMQIEGLNEELAYLKKNHEEEMKEFSSQLA-GQVNVEMDAAPGVDLTRVLAE
MREQYEAMAEKNRRDVEAWFFSKTEELNKEVASNTEMIQTSKTEITDLRRTMQELEIELQ
SQLSMKAGLENSLAETECRYATQLQQIQGLIGGLEAQLSELRCEMEAQNQEYKMLLDIKT
RLEQEIATYRSLLEGQ
>Human_K16
```

```
EKVTMQNLNDRLASYLDKVRALEEANADLEVKIRDWYQRQRP-----SEIKDYSP--YFK
TIEDLRNKIIAATIENAQPILQIDNARLAADDFRTKYEHELALRQTVEADVNGLRRVLDE
LTLARTDLEMQIEGLKEELAYLRKNHEEEMLALRGQTG-GDVNVEMDAAPGVDLSRILNE
MRDQYEQMAEKNRRDAETWFLSKTEELNKEVASNSELVQSSRSEVTELRRVLQGLEIELQ
SQLSMKASLENSLEETKGRYCMQLSQIQGLIGSVEEQLAQLRCEMEQQSQEYQILLDVKT
RLEQEIATYRRLLEGE
>Human_K17
EKATMQNLNDRLASYLDKVRALEEANTELEVKIRDWYQRQAP-----GPARDYSQ--YYR
TIEELQNKILTATVDNANILLQIDNARLAADDFRTKFETEQALRLSVEADINGLRRVLDE
LTLARADLEMQIENLKEELAYLKKNHEEEMNALRGQVG-GEINVEMDAAPGVDLSRILNE
MRDQYEKMAEKNRKDAEDWFFSKTEELNREVATNSELVQSGKSEISELRRTMQALEIELQ
SQLSMKASLEGNLAETENRYCVQLSQIQGLIGSVEEQLAQLRCEMEQQNQEYKILLDVKT
RLEQEIATYRRLLEGE
>Human_K18
EKETMQSLNDRLASYLDRVRSLETENRRLESKIREHLEKKGP------QVRDWSH--YFK
IIEDLRAQIFANTVDNARIVLQIDNARLAADDFRVKYETELAMRQSVENDIHGLRKVIDD
TNITRLQLETEIEALKEELLFMKKNHEEEVKGLQAQIASSGLTVEVDAPKSQDLAKIMAD
IRAQYDELARKNREELDKYWSQQIEESTTVVTTQSAEVGAAETTLTELRRTVQSLEIDLD
SMRNLKASLENSLREVEARYALQMEQLNGILLHLESELAQTRAEGQRQAQEYEALLNIKV
KLEAEIATYRRLLEDG
>Human_K19
EKLTMQNLNDRLASYLDKVRALEAANGELEVKIRDWYQKQGP-----GPSRDYSH--YYT
TIQDLRDKILGATIENSRIVLQIDNARLAADDFRTKFETEQALRMSVEADINGLRRVLDE
LTLARTDLEMQIEGLKEELAYLKKNHEEEISTLRGQVG-GQVSVEVDSAPGTDLAKILSD
MRSQYEVMAEQNRKDAEAWFTSRTEELNREVAGHTEQLQMSRSEVTDLRRTLQGLEIELQ
SQLSMKAALEDTLAETEARFGAQLAHIQALISGIEAQLGDVRADSERQNQEYQRLMDIKS
RLEQEIATYRSLLEGQ
>Human_K20
EKMAMQNLNDRLASYLEKVRTLEQSNSKLEVQIKQWYETNAP-----RAGRDYSA--YYR
QIEELRSQIKDAQLQNARCVLQIDNAKLAAEDFRLKYETERGIRLTVEADLQGLNKVFDD
LTLHKTDLEIQIEELNKDLALLKKEHQEEVDGLHKHLG-NTVNVEVDAAPGLNLGVIMNE
MRQKYEVMAQKNLQEAKEQFERQTAVLQQQVTVNTEELKGTEVQLTELRRTSQSLEIELQ
SHLSMKESLEHTLEETKARYSSQLANLQSLLSSLEAQLMQIRSNMERQNNEYHILLDIKT
RLEQEIATYRRLLEGE
>Human_K23
GKATMQNLNDRLASYLEKVRALEEANMKLESRILKWHQQRDP-----GSKKDYSQ--YEE
NITHLQEQIVDGKMTNAQIILLIDNARMAVDDFNLKYENEHSFKKDLEIEVEGLRRTLDN
LTIVTTDLEQEVEGMRKELILMKKHHEQEMEKHHVPSD-FNVNVKVDTGPREDLIKVLED
MRQEYELIIKKKHRDLDTWYKEQSAAMSQE-AASPATVQSRQGDIHELKRTFQALEIDLQ
TQYSTKSALENMLSETQSRYSCKLQDMQEIISHYEEELTQLRHELERQNNEYQVLLGIKT
HLEKEITTYRRLLEGE
>Human_K24
EKQTMQNLNDRLANYLDKVRALEEANTDLENKIKEWYDKYGPGSGDGGSGRDYSK--YYS
IIEDLRNQIIAATVENAGIILHIDNARLAADDFRLKYENELCLRQSVEADINGLRKVLDD
LTMTRSDLEMQIESFTEELAYLRKNHEEEMKNMQGSSG-GEVTVEMNAAPGTDLTKLLND
MRAQYEELAEQNRREAEERFNKQSASLQAQISTDAGAATSAKNEITELKRTLQALEIELQ
SQLAMKSSLEGTLADTEAGYVAQLSEIQTQISALEEEICQIWGETKCQNAEYKQLLDIKT
RLEVEIETYRRLLDGE
>Human_K25
EKVTMQNLNDRLASYLDSVHALEEANADLEQKIKGWYEKFGPGSCR-GLDHDYSR--YFP
IIDDLKNQIIASTTSNANAVLQIDNARLTADDFRLKYENELALHQSVEADVNGLRRVLDE
ITLCRTDLEIQYETLSEEMTYLKKNHKEEMQVLQCAAG-GNVNVEMNAAPGVDLTVLLNN
MRAEYEALAEQNRRDAEAWFNEKSASLQQQISEDVGATTSARNELTEMKRTLQTLEIELQ
SLLATKHSLECSLTETESNYCAQLAQIQAQIGALEEQLHQVRTETEGQKLEYEQLLDIKL
HLEKEIETYCLLIGGD
>Human_K26
EKVTMQNLNDRLASYLDHVHALEEANADLEQKIKGWYEKCEPGSSR-EHDHDYSR--YFS
VIEDLKRQIISATICNASIVLQNDNARLTADDFRLKYENELALHHSVEADTSGLRRVLDE
LTLCTTDLEIQCETLSEELTYLKKSHEEEMEVLQYTAG-GNVNVEMNATPGVDLTVLLNN
MRAEYEDLAEQNRKDAEAWFNERSATLQQQISDHEGAATAARNELTELKRNLQTLEIELQ
SLMAVKHSYECSLAETEGNYCNQLQQIQDQIGVMEEQLQQIRTETEGQKLEYEQLLDVKI
FLEKEIDIYCNLLDGE
>Human_K27
EKVTMQNLNDRLASYLENVRALEEANADLEQKIKGWYEKFGPGSCR-GLDHDYSR--YFP
IIDELKNQIISATTSNAHVVLQNDNARLTADDFRLKFENELALHQSVEADINGLRRVLDE
LTLCRTDLEIQLETLSEELAYLKKNHEEEMKALQCAAG-GNVNVEMNAAPGVDLTVLLNN
MRAEYEALAEQNRRDAEAWFNEKSASLQQQISDDAGATTSARNELIEMKRTLQTLEIELQ
SLLATKHSLECSLTETESNYCAQLAQIQAQIGALEEQLHQVRTETEGQKLEYEQLLDIKV
HLEKEIETYCLLIDGE
>Human_K28
EKVTMQNLNDRLASYLDNVRALEEANAELERKIKGWYEKYGPGSCR-GLDHDYSR--YHL
TIEDLKNKIISSTTTNANVILQIDNARLAADDFRLKYENELTLHQNVEADINGLRRVLDE
LTLCRTDQELQYESLSEEMTYLKKNHEEEMKALQCAAG-GNVNVEMNAAPGVDLAVLLNN
MRAEYEALAEQNRKDAEAWFNEKSASLQQQISHDSGAATFARSQLTEMRRTLQTLEIQLQ
SLMATKHSLECSLTETESNYCTQLAQIQAQIGALEEQLHQVRTETEGQKLEYEHLLDVKV
HLEKEIETYCRLIDGD
>Human_K31
EKETMQFLNDRLASYLEKVRQLERDNAELENLIRERSQQQEP-----LLCPSYQS--YFK
TIEELQQKILCTKSENARLVVQIDNAKLAADDFRTKYQTELSLRQLVESDINGLRRILDE
LTLCKSDLEAQVESLKEELLCLKSNHEQEVNTLRCQLG-DRLNVEVDAAPTVDLNRVLNE
TRSQYEALVETNRREVEQWFTTQTEELNKQVVSSSEQLQSYQAEIIELRRTVNALEIELQ
AQHNLRDSLENTLTESEARYSSQLSQVQSLITNVESQLAEIRSDLERQNQEYQVLLDVRA
RLECEINTYRSLLESE
>Human_K32
EKETMQFLNDRLASYLTRVRQLEQENAELESRIQEASHSQVL-----TMTPDYQS--HFR
TIEELQQKILCTKAENARMVVNIDNAKLAADDFRAKYEAELAMRQLVEADINGLRRILDD
LTLCKADLEAQVESLKEELMCLKKNHEEEVGSLRCQLG-DRLNIEVDAAPPVDLTRVLEE
MRCQYEAMVEANRRDVEEWFNMQMEELNQQVATSSEQLQNYQSDIIDLRRTVNTLEIELQ
AQHSLRDSLENTLTESEARYSSQLAQMQCMITNVEAQLAEIRADLERQNQEYQVLLDVRA
RLEGEINTYRSLLENE
>Human_K33a
EKETMQFLNDRLASYLEKVRQLERDNAELENLIRERSQQQEP-----LVCASYQS--YFK
TIEELQQKILCSKSENARLVVQIDNAKLASDDFRTKYETELSLRQLVESDINGLRRILDE
LTLCRSDLEAQVESLKEELLCLKQNHEQEVNTLRCQLG-DRLNVEVDAAPTVDLNQVLNE
TRSQYEALVETNRREVEQWFATQTEELNKQVVSSSEQLQSYQAEIIELRRTVNALEIELQ
AQHNLRDSLENTLTESEARYSSQLSQVQRLITNVESQLAEIRSDLERQNQEYQVLLDVRA
RLECEINTYRSLLESE
>Human_K33b
EKETMQFLNDRLASYLEKVRQLERDNAELENLIRERSQQQEP-----LLCPSYQS--YFK
TIEELQQKILCSKSENARLVVQIDNAKLAADDFRTKYQTEQSLRQLVESDINSLRRILDE
LTLCRSDLEAQMESLKEELLSLKQNHEQEVNTLRCQLG-DRLNVEVDAAPAVDLNQVLNE
TRNQYEALVETNRREVEQWFATQTEELNKQVVSSSEQLQSYQAEIIELRRTVNALEIELQ
AQHNLRYSLENTLTESEARYSSQLSQVQSLITNVESQLAEIRSDLERQNQEYQVLLDVRA
RLECEINTYRSLLESE
```

```
>Human_K34
EKETMQFLNDRLASYLEKVRQLERDNAELEKLIQERSQQQEP-----LLCPSYQS--YFK
TIEELQQKILCAKAENARLVVNIDNAKLASDDFRSKYQTEQSLRLLVESDINSIRRILDE
LTLCKSDLESQVESLREELICLKKNHEEEVNTLRSQLG-DRLNVEVDTAPTVDLNQVLNE
TRSQYEALVEINRREVEQWFATQTEELNKQVVSSSEQLQSCQAEIIELRRTVNALEIELQ
AQHNLRDSLENTLTESEAHYSSQLSQVQSLITNVESQLAEIRCDLERQNQEYQVLLDVRA
RLECEINTYRSLLESE
>Human_K35
EKETMQSLNDRLAGYLEKVRQLEQENASLESRIREWCEQQVP-----YMCPDYQS--YFR
TIEELQKKTLCSKAENARLVVEIDNAKLAADDFRTKYETEVSLRQLVESDINGLRRILDD
LTLCKSDLEAQVESLKEELLCLKKNHEEEVNSLRCQLG-DRLNVEVDAAPPVDLNRVLEE
MRCQYETLVENNRRDAEDWLDTQSEELNQQVVSSSEQLQSCQAEIIELRRTVNALEIELQ
AQHSMRDALESTLAETEARYSSQLAQMQCMITNVEAQLAEIRADLERQNQEYQVLLDVRA
RLECEINTYRGLLESE
>Human_K36
EKETMQFLNDRLANYLEKVRQLERENAELESRIQEWYEFQIP-----YICPDYQS--YFK
TIEDFQQKILLTKSENARLVLQIDNAKLAADDFRTKYETELSLRQLVEADINGLRRILDE
LTLCKADLEAQVESLKEELMCLKKNHEEEVSVLRCQLG-DRLNVEVDAAPPVDLNKILED
MRCQYEALVENNRRDVEAWFNTQTEELNQQVVSSSEQLQCCQTEIIELRRTVNALEIELQ
AQHSMRNSLESTLAETEARYSSQLAQMQCLISNVEAQLSEIRCDLERQNQEYQVLLDVKA
RLEGEIATYRHLLEGE
>Human_K37
EKETMKFLNDRLANYLEKVRQLEQENAELETTLLERSKCHES-----TVCPDYQS--YFR
TIEELQQKILCSKAENARLIVQIDNAKLAADDFRIKLESERSLHQLVEADKCGTQKLLDD
ATLAKADLEAQQESLKEEQLSLKSNHEQEVKILRSQLG-EKFRIELDIEPTIDLNRVLGE
MRAQYEAMVETNHQDVEQWFQAQSEGISLQAMSCSEELQCCQSEILELRCTVNALEVERQ
AQHTLKDCLQNSLCEAEDRYGTELAQMQSLISNLEEQLSEIRADLERQNQEYQVLLDVKA
RLENEIATYRNLLESE
>Human_K38
EKETMQFLNDRLANYLEKVRQLEQENAELEATLLERSKCHES-----TVCPDYQS--YFH
TIEELQQKILCSKAENARLIVQIDNAKLAADDFRIKLESERSLRQLVEADKCGTQKLLDD
ATLAKADLEAQQESLKEEQLSLKSNHEQEVKILRSQLG-EKLRIELDIEPTIDLNRVLGE
MRAQYEAMLETNRQDVEQWFQAQSEGISLQDMSCSEELQCCQSEILELRCTVNALEVERQ
AQHTLKDCLQNSLCEAEDRFGTELAQMQSLISNVEEQLSEIRADLERQNQEYQVLLDVKT
RLENEIATYRNLLESE
>Human_K39
EKETMQILNERLANYLQKVRMLERENAELESKIQEESNKELP-----VLCPDYLS--YYT
TIEELQQKILCTKAENSRLVSQIDNTKLTADDLRAKYEAEVSLRQLVESDANGLKQILNV
LTLGKADLEAQVQSLKEELLCLKNNHKEEINSLQCQLG-ERLDIEVTAAPSADLNQVLQE
MRCQYEPIMETNRKDVEQWFNTQIEELNQQVVTSSQQQQCCQKEIIELRRSVNTLEVELQ
AQHRMRDSQECILTETEARYTALLTQIQSLIDNLEAQLAEIRCALERQNQEYEILLDVKS
RLECEITTYRSLLESS
>Human_K40
EKETMQFLNDRLASYLEKVRSLEETNAELESRIQEQCEQDIP-----MVCPDYQR--YFN
TIEDLQQKILCTKAENSRLAVQLDNCKLATDDFKSKYESELSLRQLLEADISSLHGILEE
LTLCKSDLEAHVESLKEDLLCLKKNHEEEVNLLREQLG-DRLSVELDTAPTLDLNRVLDE
MRCQCETVLANNRREAEEWLAVQTEELNQQQLSSAEQLQGCQMEILELKRTASALEIELQ
AQQSLTESLECTVAETEAQYSSQLAQIQCLIDNLENQLAEIRCDLERQNQEYQVLLDVKA
RLEGEINTYWGLLDSE
```

Suppl. Fig. S12. Amino acid sequence alignment of the intermediate filament domains of type I keratins used for the phylogenetic analysis shown in Figure 3. The multiple sequence alignment is shown in fasta_aln format.

```
>Chicken_K80
EKEDIKLLNNQFVTLIEKVQCLEQQNKILTTRWNFLKDQDNSH--SESDVKAIYDQYMSK
MNQEMQALNYEQENLESELTEVLSTMDNFRSKYEDEIRLCSGMEYTFMELKKDLDASTLH
RTELEVKLSGLQELMDLKKTIYEQELEELLTEI--KDISVVLGIDNTCKL-DLSRIVEEV
RAQYEALALRSWEEAEALTRRKLNEGNTQSATYGGHLLDSRREIADLNIQIQKLRSCIVS
QKSQCLYLEENIKEAGEHGEMALKDAKAKLVKLEEALQKSKEDMAHLVKEYQELMNIKLA
LDLEILTYRKLVEGE
>Chicken_K7
EKEQIKTLNNRFASFIDKVRFLEQQNKMLETKWSLLQDQKT----TRSNIVPMFEAYITN
LRRQLDGLLNDKGRLEGELRNMQDLVEDFKAKYEDEINKRTTAENEFVVLKKDVDAAYMN
KVELEAKVDALTDEINFLRSLYEAELRELQAQI--SDTSVVLSMDNSRNL-DLDSIIAEV
KAQYEDIANRSRAEAESWYQSKFEALQVTAGKHGDDLRNTKNEITEINRVIQRLQGEIEN
AKAQRAKMEAAIAEAEERGELAVKDARAKLEELEAALQKAKQDMARQLREYQELMNVKLA
LDIEIATYRKLLEGE
>Chicken_K84L
EKEQIKTLNNKFASFIEKVRLLEQQNEVLETKWSFLKGQKH----GRNTIVPMLEACIGN
LKKQLEALGCNRARLETQLKAVQEALETNKKIYEDERSRRSSTEKELIALKKDVECFFLN
KAELGAKVGRMEGEFGFLRVLYEEESQQLRAHI--SDTAVVVQMDNSWDP-NLDGIVANV
KAHYEDTACRSRAETEAWCKGKFKELQVTAGRNADSLQEKKREAAELARMVKKLSGEVRS
AKEQCCKLETAVADAEQRKEMSIKDAKSKLTELEAALQKAKQDLARQLWEFQELMNIKLA
LDVEITTYKKLLEGE
>Chicken_K5L3
EKEQIQTLNNKFASFIDKVRFLEQENKVLETKWALLQEQGHKT--VRNNIEPLFEAYINN
LRRQLSSLLADKENIGGELSKVQSLAEDFKSKYEEEINKRTAAENEFVILKKEVDSTYMT
KTELQARLDALLEEIDFLRALYEAELSQLQSQI--ADTSVILTMDNNRSL-DMDSIIAEV
KAQYEDIAKRSREEAESWYQSRYEELQATAGRHGDDLRSTKQEISELNRHVQRLRAEIDS
VKKQCASLQTAIADAEQRGELALKDARDKLAELETALQQAKADLARQLREYQELMNVKLA
LDIEIATYRKLLEGE
>Chicken_K5L2
EKEQIKTLNNKFASFIDKVRFLEQQNKVLETKWSLLQEQGMKT--VRNNLEPLFETYINN
LRMQLNSLLSDKGRLEGELVNTQYLVEDFKKKYEDEINRRTIAENEFVTLKKDVDASYMN
KVELQAKADALTEEINFLRALYEAELSQMQTQI--SDTSVVLTMDNNRNL-DLDSIIAEV
KAQYEDIANRSRAEAESWYQTKYEELQATAGRHGDDLRSTKQEISELNRHVQRLRAEIDS
VKKQCANLKAAIADAEERGEIALKDAKAKLAELEDALQQAKADLARQLREYQELMNVKLA
LDIEIATYRKLLEGE
>Chicken_K5L1
EREQIKTLNNKFASFIDKVRFLEQQNKVLETKWSLLQEQGHTV--TRKSLEPLFETYINN
LRRQLDSLMGERGRLDSELRSMQDMVEDFKNKYEDEINRRTGAENEFVVLKKDVDGAYMN
KVELQAKADGLADEINFLRALYEAELSQMQQQV--SDTSVVLSMDNNRSL-DLNSIIAEV
KAQYEDIANRSRAEAEAWYQNKYEELQVSAGRHGDDLRNTKMEISEINRMVQRLRNEIES
VKKQCANLQAAIAEAEERGEMALKDAKSKLAELEDALQKAKADLARQLREYQELMNVKLA
LDIEIATYRKLLEGE
>Chicken_HBS1
EKEQIKNLNNQFACFIDKVRLLEQQNKVLTTKWELLQQYVLPA--SRRNLEPVFENFICN
LRKQLECVLGERERLENEERCLRDLVQEYKCKYEDEINKRTAAENEFVVLKKDVDCLYLT
KEELEVRVGLLRQQLEFLKCIYAEERAQMDCQL--CDTSVIVQMDNSRDL-DMEGIIKSV
ECCYEEIAQKSKAEVEAFYQTRLEELHSSRGKFCDDLRNNQSEIAELNRMIQKLQCESDN
VKKQIAALQTAICDAEQRGDCALKDARQKLVDLQTALQQAKDKMACLLRDYQELLNVKLA
LDIEIATYRTLLEGE
>Chicken_HBS2
EKEQIKTLNTQFACFIDKVRFLEQQNKVLETKWGLLQQYVLPK--KGKNLELYFENYICD
LRKRLDCLLCEKQKLGNEECATSQLVEEFKCKYEEEINRRTTVENEFVALKKDADCIFLN
KEELEVKVDLLRRQLELLKCVFEEERAQVDRQL--CDTSVIVKMDNNRDL-DMESIIKNV
ECWYQEIAQKSKEEVDAFYQTRFQELQDKRGKYCDDLQSNKCEISELTRMIQKLQCELEN
VKKQVSCLQTSICDVEQRGDCALKDAREKHVELQNALQKAKDELACMLRDYQELLNVKLA
LDIEIATYKTLLEGE
>Chicken_HBS3
EREQMRSLNNQFACLIDKVQHLEQQNRLLATKWAFLQKHILPS---QKNFKHVFNTFICS
LQRRLDSLLHERGQMELELNNTEKLIEDLKCKYEQEVNRCTAAENEFVLLKKDADCVYLT
KAELEAKLETLKQQIELLKCMSAQGTAEMEKSL--CDTSVVVKMDNSRSL-DMEGILRGI
ELWYEDVAQKSKAELDALYRTRYQELEETKRKQCSELKSHQQEIEELSFVMQRWQCDLEN
VKKRVSSLRTSVSDTEQRGDRALKDAREKHLELQNALQKAKDELACMLRDYQELLNVKLA
LDIEIATYKTLLEGE
>Chicken_K78LT
EKEQIKTLNSNFASFIDKVQCLERQNQALMTKWELLQQQSSRPE-ESRSITSFFQSYINN
LQRQLDMLQSQKEQLDPEAYEMLQLVEDYKKRFEDEINKRASKEEEYVELKKELDSTYMR
KMEFEVRVEILKQELEFLRCLYEAELSQLQTVV--DNTNVILSMDNGREV-NMDGIIEEV
RQEYEKIAQKGKDEVNAMYQGRYQDLQNMRVNQREQLRYTYQEIQELTRQIQRLQPEIEI
VRRENDSRQEAIRDAEQRGSSAVKDGQKKLQELENALQQAREDLARILHDYQELLNAKLS
LDVEIAMYRSLLEEE
>Chicken_K78L4
EKEQIKTLNNQFASFIDKVRFLEQQNKVLSTKWELLQQQGPSG--PRKNLDVIFENYIQG
LRRRLESLLGQRGELESELQNMRQYVEEYKTKYEEEINRRTAAENEFVVLKKDVDCAYMT
KVELEAKVGALTDEISFLRCIYEEELAQMQTIS--RDLSVVVSMDNNRHL-DLDSIIEEV
RRQYEQIAQNSRAEAEAWYQSRYEELQTTAGRHGDSLRNTKIEIQELTRNVQRLRAEIEN
VKKQNQQLQAAIAEAEERGEMALKDARRKLEELECALSKDKEELARLLKEYQELLNIKIA
LDVEIAMYRKLLEGE
>Chicken_K78L3
EKEQIKTLNNQFASFIDKVRFLEQQNKVLSTKWELLQQQGPSG--PRKNLDAIFENYIQG
LRRRLESLLGQRGELESELQNMRQYVEEYKTKYEEEINRRTAAENEFVVLKKDVDCAYMT
KVELEAKVGALTDEINFLRCIYEEELSQMQTIS--RDLSVVVSMDNNRHL-DMDSIIEEV
RRQYEQIAQNSRAEAEAWYQSRYEELQTTAGRHGDSLRNTKIEIQELTRNIQRLRAEIEN
VKKQNQQLQAAIAEAEERGEMALKDARVKLEELEAALSKDKEELARLLKEYQELLNVKIA
LDVEIAMYRKLLEGE
>Chicken_K78L2
EKEQIKTLNNQFASFIDKVRFLEQQNKVLSTKWELLQQQGPSG--PRKNLDVIFENYIQN
LRRQLDSILAQRGQLESELQNMQQYVEDYKNKYEEEINRRTTAENEFVVLKKDVDTAYMT
KVELEAKVGALTDQINFLRAIYEEELSQMQTIS--RDLSVVVSMDNNRHL-DMDSIIEEV
RRQYEQIAQSSRAEAEAWYQSQYEQLQSTAGMHGDSLRNTKIEIQELTRNIQRLRAEIEN
VKKQNQQLQAAIAEAEERGEMALKDARVKLEELEAALSKDKEELARLLKEYQELLNIKIA
LDVEIAMYRKLLEGE
>Chicken_K78L1
EREELKTLNNKFASFIDKVRFLEQQNKVLETKWKLLQEQSPTT--STRSLDPYFEAYISR
LRKQLDGLSSEKIQLESELKSFQDMVEDFKTKYEEEINKRTAAENDFVLLKKDVDGVYMN
KVELQAKLDSLADELNFLRNLYETELCQMQKTV--SDTSVVLSMDNNRCL-DLDSIIAEV
KAQYEEIANRSRLEAEAWYQSKYKELQATAGKHGDSLRDTKAEISELNRVIQRLRAEIEN
VKKQCESVQSSIADAEQRGELALKDARDKLTELETALKKAKADLAQQLRDYQELMNVKLA
LDIEIATYRKLLEGE
>Chicken_K8
EKEQIKTLNNRFASFIDKVRFLEQQNKVLETKWSLLQSQQP----QRSDLRGLFEGYVGT
LRRQLEGLGQERLRLEAELGSMQGLVEDFKNKYEEEINHRTEKENEFVLLKKDVDEAYMS
KVELESRLESLTDEINFLRQLYDEELSQMQTQI--SDTSVVLTMDNNRSL-DLDSIIAEV
KAQYEDIANRSGLEAESWYQTKYEELKTTAGKHGDDLRTTRSEISELNRMIQRIQAEIEV
LKNQRATLETAIAEAEERGEMALKDARAKLAELEAALQKAKQDLARQLREYQELMNVKLA
```

```
LDIEIATYRKLLEGE
>Alligator_K80
EKEEIKVLNNQFVALIKQVQSLELQNKMLETRWDFLKDQDNSS--SEVDIKCIYDEYMGR
MEQEMKSIDQEKEELESELTKVLDSMDDFRAKYEGEMQLRNSLEYTFLELKKDLDAGTLH
RTELEVKLNGLQEHMELKKSVYEQELQELLAEV--KDISVVLGIDTRCNL-DLHSIVEEV
RAQYEALAVWSWKEAEAHIWSKLNEGVPRSAMYGNHLFNSRREIADLNIQIQKLRSCIVS
LKSQCLSLEESIKEAGEQGEFALQDASAKLASLEEALQKGKEDVTHLVKEHQQLMNTKLA
LDVEILTYRKLVEGE
>Alligator_K7
EKEQIKTLNNKFASFIDKVRFLEQQNKMLETKWSLLQDQKT----ARSNIAPLFEAYISN
LRRQLDGLMNDKGRLEGELKNMQDLVEDFKNKYEDEINRRTTAENEFVVLKKDVDGAYMN
KVELEARVDALTDEINFLRSLYEAELQELQTQI--SDTSVVLSMDNSRNL-DLDSIIAEV
KAQYEDIANRSRAEAESWYQSKFEVLQATAGKHGDDLRSTKNEIAETNRMIMRLQSEIDN
VKNQRAKLEAAIAEAEERGEMAIKDARAKQDELEAALQKAKQDMARQLREYQDLMNVKLA
LDIEIATYRKLLEGE
>Alligator_K84L1
EKEQIKTLNNKFASFIDKVRFLEQQNKVLETKWSFLQNQQC----RRNTIMPTLDAYICN
LRKQLEALGCEGAQLEADLKAAHEVMETNKKMYEEECSRRTCAENEFVVLKKEVDCVFLN
KAELEARVESLKDEIIFLKSLYEEEIHMLHSHI--SDTSVIVQMDNSRNL-DLDGIITEV
KAQYEDIASRSRAEAEAWYESKFEELRVTAGRHADNLRDTKNEIAELTRIIQRLRGEVGT
AKDQRCKLEAAVAEAEQQGELAIKDARCKLTDLETALQQAKADLAHQLREYQELMNVKLA
LDIEIATYRKLLEGE
>Alligator_K84L2
EKEQIKTLNNKFASFIDKVRFLEQQNKVLETKWRFLQEQKR----YKSNIEPMFEAYIYN
LRRQLEVLAGDRAKLETELSNMQETLEDFKEKYEEETSRRACAENEFVMLKKDVDCAYMN
KAELEAKVESLIEEITFLKNLHEEEIAQLQASI--SDTSVIVQMDNSRAL-DMDDIIAEV
KAQYEDIANRSRADAESWYYSKYEELRETAGKHSDKLRNTKNEIMELSRVIQRLNGELEN
AKAQRGKLEAAIAEAEERGEMALKDAKCKLTDLEAALQKAKQDMACQLREYQELMNVKLA
LDIEIATYRKLLEGE
>Alligator_K5L4
EKEQIKTLNNKFASFIDKVRFLEQQNKVLETKWSLLQEHKT----ARSNIEPMFEAYISN
LRRQVEVLGGERIRLDSELKGMQDVVEDFRIRYEDEIHKRTAAENEFVVLKKDVDAAYMN
KVELGAKVDGLTDEINFLRALYEAELAQMQSQI--SETSVVLSMDNNRNL-DLDSIIEEV
KAQYEDIANRSRAEAESWYQSKYEELQLTAGRHGDDLRNTKVEITEMSRMIHRLHSEIDS
VKKQCASLQTAIADAEQRGELALKDARVKLAELEDALQKAKQDMARQLREYQELMNVKLA
LDIEIATYRKLLEGE
>Alligator_K5L3
EKEQIQTLNNKFASFIDKVRFLEQQNKVLETKWMLLQEQGQKT--VRNNIEPLFETYINN
LRIQLNGLVGDRGRLEGELDNTQNLLEDFKNRYEEETNRRHDAENDFVTLKKDVDGAFMN
KVELQAKVDSLIDEINFLRAIYEAELSQMQTQI--TDTSVVLSMDNNRSL-DMDSIIAEV
KAQYEDIANRSRVEAESWYQVKYEELQATAGRHGDDLRNTKHEISEINRHIQRLRADIES
VKRQCASLQAAIAEAEERGEMALKDARDKLAQLEDALQQAKADLARQLREYQELMNVKLA
LDIEIATYRKLLEGE
>Alligator_K5L2
EREQIKTLNNKFASFIDKVRFLEQQNKVLETKWTLLQEQGQTV--TRKTLEPLFEAYINN
LRRQLDSLLGDGPRLDSELRNMQDLVEDFKNKYEDEINRRTAAENEFVMLKKDVDNAYMN
KVELQAKADSLADEINFLRALYDAELSQVHQQV--SDTSVILSMDNNRSL-NLDSIISDV
KAQYEDIANRSRAEAESWYQSKYEELQVSAGRHGEDLRSTKMEITEINRMIQRLRNEIDN
VKKQCANLQAAIAEAEERGEMALKDARAKLADLEEALQKAKADLARQLREYQELMNVKLA
LDIEIATYRKLLEGE
>Alligator_K5L1
EKEQIKTLNNKFASFIDKVRFLEQQNKVLETKWALLNDQGQKSSLSKNNMDPLFEAYISN
LKKQLNSLLNERGRLDGELKNMQDLVEDFKNKYEDEINRRTTAENEFVVLKKDVDAAYMN
KVELEAKVDALMDELNFLRALYDAELAQLSAQM--SDTSVILSMDNNRDL-DLSSIITEV
KAQYEDIANRSRAEAEAWYQTKFEELQATAGKHGDDLRNTKGEISELTRLIQRIRAEIEN
ARNQCANLQTAIADSEERGELALKDAKLKLAELEDALQKAKADMTRQLREYQELMNVKLA
LDIEIATYRKLLEGE
>Alligator_HBS1
EKEQIKNLNNKFACFIDKVRLLEQQNKVLTTKWDLLQQCIVPG--TRRNLEPLYESFICN
LKKQLEHLLCDRDKLMCEEKAANHLVDEFKCKYEEQINRRTAAENEFVVLKKDVDCLHLT
KEELEIRVALLRQQLEFLTCIFAEERAQMDCQL--CDTSVIVEMDNNRGL-DMDSIINSV
KCCYEEIAHKSKAEVEAFYQTRLEELHTNRGKYCDDLRISQCEIADLKRGIQKLQGELDG
VKKQIGCLETAICDAEHHGDCTLKDAREKHINLQTALQQAKDKLACLLRDYQELLNVKLA
LDIEIATYRSLLEGE
>Alligator_HBS2
EKEQMKCLNNQFACFIDKVQCLEQQNKALETKWNLLQQHTMPA--ARKTLEAYFENYICK
LKKQLECLLGEREQLSNKECVAKKLVDEFRCKYEEVINKRMTAEHGFVLAKKDVDYAFLN
TEEQEVKVDLLKCQLELLNHVFTEERAQMDCQL--CDTSVMVKMDNRRDL-DMETIIKNV
ECCYKEIAQKSKEEVDTLYQTRFQELQEQRGRFCDDLESNKREIAQLTQLIQELQCELDN
VKKQVGCLQTDIRDAEQHGDFTLQDGRAKHVELQNALQKAKDELASMLRDYQELLNVKLA
LDIEIAMYKTLLEGE
>Alligator_HBS3
EREQIKKLNNQFACFIDKVRCLEQQNKVLGTKWNLLQEQVLPC---RKNLQHLFENYLCS
LQRQLDCLLSERGQLEPELQNGQKLVEELKCKYEQEINRRTASENEFILLKKDVDCSYMS
KVELEAKVDTLRQEIEFLRCVYAQELVQMEGNL--CDTSVVVKMDNNRDL-DMEGIIREI
ECWYEEIAQKSKAEVDALYRTRFQELQETKGRYCNDLKCNHHEITELSRVVQRLQHNLDN
AKKEVACLQTSICETEQRGDCALKDAQGKYGELQNALQKAKDELASMLRDYQELLNIKLA
LDIEIAMYKTLLEGE
>Alligator_K78LT
EKEQIKSLNDKFACFIDKVRCLEQENQVLLTKWELLQQQGGQTG-VHRDIEPFFQSYICH
LRRQRDTLQSQKEELNLEDCKMLQIVNEYKASYEKEIDRRLCTEEEFVNLKKNLDCIYMG
KMEVGVKVNILREEIEFLKCLYAMELSDVQRIA--QDTNVVVSMDNCRDL-DVEGIIAEI
RREYEGLAQKSQAEVNAVYQSKYQDLHNTWGRHCDRLRTRQHEIQELTRLIQKLRLDIGD
VKKKNACLQEEIADVERRGCCAVSDAQEKLRELRCSLLKAKDNLAHLLRDYQELLNVKMA
LDIEIATYRALLEGE
>Alligator-K78L6
EREQIKTLNNKFASFIDKVRFLEQQNKVLETKWDLLQGTTT----TRVSLEPMFEAYINN
LQCQLENLLGNRGQLEGELRNLQDLVEDFKKKYEEEINRRTAIENEFVMSKKDADHSFMN
KVELQVKVDSLIDEISFLRAFFKAELDQMQGQI--TDTNVILQMDNNRDL-DLNSIISEV
KAQYEDIANRSRAEAEAWYQSKFQELQTTAMTHGDDLKNTRNEISELTRIIQRLKAEIDN
VKKQIAGLQTAIADAEQRGEMALKDARAKLSDLQNALQTAKDELAHLLRDYQELMNVKLA
LDIEIATYRTLLEGE
>Alligator_K78L5
EKEQIKTLNNQFATFIDKVRFLEQQNKVLSTKWELLQQQGHSG--PRKDLSSLFESYIQN
LKRQLDSILHQREQLESDLQNMQPHVEEYKKKYEEEINRRATAENEFVVLKKDVDNAYMN
KVQLEAKVQALMDEIDFLRHVFEEELSQMHTIS--RDLSVVVSMDNTRHL-DMDSIIDEV
RRQYEDIARSSRAEAEEWYQSRYEELQNTAGRHGDNLRNTKQEIQELTRCIQRLRSEIEN
VKKQVRALQAAIAEAEDRGEMALKDARSKLQELEAALQKDKEELARLLKEYQELLNIKIA
LDIEIAMYRKLLEGE
>Alligator_K78L4
EKEQIKTLNNQFATFIDKVRFLEQQNKVLSTKWELLQQQGHSG--PSKDLSSLFEIYIQN
LKRQLDSILDERGQLVSELQKMQGHLEDYKTKYEEEINRRTTAENEFVVLKKDVDNAYMS
KVELEAKVQALTDEINFLRYVYEEEMSQMHTIS--RDLSVVVSMDNTRHL-DMDSIIDEV
RRQYEDIARSSRAEAEEWYQSRYEELQNTAGRHGDNLRNTKQEIQELTRCIQRLRSEIEN
```

VKKQHQQLQAAIAEAENRGEMALKDARSKLQELEAALQKDKEELARLLKEYQELLNIKIA
LDIEIAMYRKLLEGE
>Alligator_K78L3
EKEQIKTLNNKFATFIDKVRFLEQQNKVLSTKWELLQQQGHSG--PRKDLSSLFENYIQN
LRRQLDSIHGQRGQLESELQNMQNYVEDYKNKYEDEINRRTAAENEFVVLKKDVDCAYMT
KVDLEAKVQALIDEINFLRYVYEEELSQMQTIS--RDLSVVVSMDNSRHL-DMDSMIDEV
RRQYEDIARSSRAEAEAWYQSRYEELQTTAGRHGDNLRNTKQEIQELTRCIQRLRSEIEN
VKKQXXX---

>Alligator_K78L2
EKEQIKTLNNKFATFIDKVRFLEQQNKVLSTKWELLQQQGPSG--PRRDLTALYENYIEN
LRRQLDNVLSQRGPLESQLHDMQGYVEDYKNKYEEEINRRTSAENEFVVLKKDVDCAYMT
KVDLEAKVQALIDEINFLRYVFEEELSQLQTIS--RDLSVVVSMDNSRHL-NLDSIIDEI
RAQYEQIAQSSRAEAEAWYQSRYEELQSTAGQHGESLRNTRQEIQELTRCIQKLRSEIEN
VRKQCEQLQAAIAEAEERGEMALKDARCKLEELECALHKDKEELARLLKEYQELLNVKIA
LDIEIAMYRKLLEGE
>Alligator_K78L1
EREQMKTLNDKFASFIDKVRFLEQQNKVLETKWKLLQEQGQGTGPHSRSLDQLYEVYINS
LRKQLDGLTSEKSQLQLELKNCEDLVEDFKTKYDEEINKRTAAENDFVLLKKDVDAAYMN
KVELQAKLDSLADELNFLRCVYDAELSQMQQTV--SDTSVVLSMDNNRNL-DLDSIIAEV
KAQYEEIAQKSRAEAEAWYQCKYQELQVTAGKHGDSLKDTKAEISEMNRMIQRLRAEIEN
VKKQCENLQTSVADAEQRGELALKDARVKLSELEAALQKAKTELTRQLRDYQELMNVKLA
LDIEIATYRKLLEGE
>Alligator_K8L2
EKEELKTLNNQFVHLIERVCSLEQHKKVLETKCSILKSQKA----TPARMENLLKTYISQ
LKKHLADQGQMKGKLHLELHEIQDIMDENKQKYEEEFNLRNEKENDFLLMKKEMDEAYIS
QNELEGKLDRLRDELEFLKNFYDQEMHELQTEVHMYKESVVISMPSQPSL-EVDSIVSEA
QSQYEKMAERSCKEAEKAYRDRHNKLKDSMGRHGEELQTMKTEINDLTRKSQHIQLEIQN
LKEQCNKLKEDIANADDRYKFPIEEAKNKLHMLENAIQKARQDMAHHLREYQELLNTKLG
LDIEIATYRKLLEGE
>Alligator_K8L1
EKEQIKSLNNKFASFIDKVRFLEQQNQVLETKWSLLQGQRT----TRSNMTGMFEAYITN
LRRQLEGLDQDKLRLESELGNVQGLVEEFRSKYEDEINHRTEKENEFVLLKKDVDEAYMN
KVELESRLEGLTDEISFLRQLYEEELHQLQSQV--SNTSVVLSMDNNRSL-DMDSIIADV
RNQYEDIANQSRAEAEGMYKIKYEELRNTASKHGDDLRNTKAEISELTRTIQRMQAEIDA
VKGQRANLEAAIAEAEERGEMAVRDARGKLAELEAALQKAKQDMARQLREYQELMNVKLA
LDIEIATYRKLLEGE
>Turtle_K80
EKDEMKILNDQFASLIGKVQCLEQQNKVLTIRWSFLKDQDNSR--SELDIKLLYDQYMSK
LKQEMRAVGHEREQLESELTEVLKSMDNIRSRYEDEIHKRSGLEFTFVELKRDLDAGSMH
RTELEVKLQGLQELMELKKAVYEQELQELLAEI--KDISIVMGIDNRCNL-DLSRIVEEV
RAQYEALAFRSWEEAEALTWSKLNEGATHSATYRDQLFSGRREIAELTIHIQKLRTCIVS
LKSQCLRLEEDIKEAGGNGQLALRNANTKLAELEQALHKGKADLVGLVKEYHELMNIKLA
LDVEILTYRKLVEGE
>Turtle_K7
EKEQIKTLNNKFASFIDKVRFLEQQNKVLETKWSLLQDQKT----TRSNIAPMFEAYINN
LRKQLDGWLNDKGRLEGELKNMQDLVEDFRNKYEDEINKRTAAENEFVILKKDVDASYMN
KVELEAKVDALTDEINFLRSLYEAELRELQAQV--SDTSVVLTMDNNRNL-DMDSIIAEV
KAQYEDMAKKSRMEAESWYQTKFETLQATAGKHGDELRNTKTEITEITRMIHRLQAEIDN
VKSQRAKLEAAIAEAEERGERAIKDAMAKQEELEAALNKAKQDMARQLREYQELMNVKLA
LDIEIATYRKLLEGE
>Turtle_K84L
EKEQIKCLNNKFASFIDKVRFLEQQNKVLETKWSFLREQKH----YQNNMEPMFEAYISN
LKRQLENLGCDRAKLETELHNMQDILEDYKRKYEEECNHRTCAENEFVVLKKDVDCAYMN
KAELEVKVESLIDEITFLRRIYEEEIACLQASI--LDTSVIVQMDNNRGL-NMDDIIADV
KAQYEDIANRSRAEAESWYQCKYEELKVTASKHCDNLHSTRNEITELNRVIQRLHGELEN
VKAQRCKLEGAIAEAEERGEMALKDAKCKLAELEAALTKAKQDLACQLREYQELMNVKLA
LDIEIATYRKLLEGE
>Turtle_K5L4
EKEQIKTLNNKFASFIDKVRFLEQQNKVLETKWCLLQEQKT----AKSNIEPMFEAYINN
LRRQLEALGGERLRLDGELKNMQDTVEDFRIRYEDEINKRTAAENEFVVLKKDVDIAYMN
KVELEAKVDALTDEVNFLRALYEAELAHLQSQI--SDTSVVLSMDNSRNL-DLDGIIAEV
KAQYEDIANRSRAEAESWYQTKYEELQLTAGRHGDDLRNTKIEISEMNRMIQRLRSEIDN
VKKQCASLQTAIADAEQRGELALKDAQAKLAELEEALQKAKADMARQLREYQELMNVKLA
LDIEIATYRKLLEGE
>Turtle_K5L3
EKEQIKTLNNQFASFIDKVRFLEQQNRVLETKWSLLQEQGQKT--VKNNIEPFFENYIGN
LRRQLNNLMNERGRLEGEVNNMQNVVEDFKNRYEDEINKRTTAENDFVTLKKDVDSAYMN
KVELVAKVDSLTDEINFLRALYEEELAQMQTQV--SNTSVVLSMDNNRDL-DLDGIIAEV
KAQYEEIANRSRAEAESWYQTKYEELQVTAGRHGDDLRNTKHEISELTRIVQRLRNEIDN
VKRQCANLQAAIAEAEERGELALKDAQGKLAELEDALQKAKGDLARQLREYQELMNVKLA
LDIEIATYRKLLEGE
>Turtle_K5L2
EREQIKTLNNKFASFIDKVRFLEQQNKVLETKWTLLQEQGQTT--VKRSLEPLFDAYINN
LRRQLDNLLGERGRLDSELRNMQDMVEDFKNKYEDEINRRTAAENDFVVLKKDVDGSYMN
KVELQAKADALADEINFLRALYEAELSQMQQQV--SDTSVVLSMDNNRSL-DLDSIIREV
KAQYEEIANRSRAEAESWYQTKFEELQISVGRHGDDLRNTKVEISEINRMIHRLRNEIDN
VKKQCASLQAAIAEAEERGEMALKDARAKLAELEDALQKAKADLARQLREYQELMNVKLA
LDIEIATYRKLLEGE
>Turtle_K5L1
EKEQIKSLNNKFASFIDK-RFLEQQNKVLETKWTLLQDQGQKSNSNKNNLEPLFESYINN
LRKQLNHLLNERGRMEGELKNTQDLVEDFKNKYEDEINRRTAAENEFVVLKKDVDAAYMN
KIDLEAKVDALMDEINFLRTLYDAELAQLNVQV--SDTSVILSMDNNRDL-DLSSIVNEV
KAQYEDIANRSRAEAESWYQNKFEELRATAGKHGDDLRNTKGEISELNRLTQRLRAEIEN
ARNQCASLQTAIADSEDRGELALKDARMKVADLEDALQKAKQDMTRQLREYQELMNVKLA
LDIEIATYRKLLEGE
>Turtle_HBS1
EKEQIKCLNDQFACFIDKVRCLEQQNKVLATKWDLLQQHAVPA---KRDLLPVFENYICQ
LKKQLECLLHDRARLEDEEKAVQDLVHEYKSKYEGEINKRTHAENEFVVLKKDVDCVFMT
KEELEAKLLQLRQHFEFLKCIFIEERAQLDGQL--CDTAVVLEMDNSRDL-DLSGIIHSV
KCCYEEIAQKSKGEAETFYQTRLEELSTNRGRFCDDLKNIQGEIAELKRLIHKLQGETDK
VKKEIACLQTAIADAEQRGDCALKDAREKHIDLQNALQKAKDKLACMLRDYHELLNIKLA
LDIEIATYRTMLEGE
>Turtle_HBS2
EREQMKTLNNQFACFIDKVRHLEQQNKVLETKWNLLQQCVPPA--PQKNLEQCFENYICS
LRKQLEDLLSDKEQLTCEESASRKLVDEFKCKYEEEISRRTAAETEFVVLKKEADGTLLH
KEELEVNTTLSKQKLEFLRCVFDEERAQMDGQF--CDTAVIVKMDNSRDL-DTDCIIKNV
EGWYKEIAQRSKEEVDVLYQTRFEELQDQRGRFCEDLERNKHEIAELTRLIQKLQGETDH
VKKQIACLQTAICDAEQRGDCALKDARGKHMELQNALQRAKDELACMLRDYQALLNVKLA
LDIEITTYKKLLEGE
>Turtle_K78L4
EREQIKTLNNKFASFIDKVRFLEQQNKVLETKWDLLQQQGTTI--TKIPIDPLFEAYINN
LRRRLDSLVTDRGRLDGELRNMQDLVEDFRKKFEEEIHRRTSAENEFVVLKKEVDAAYMK
KMELQAKVDSLIDEINLLRAVYEAELAQMQGQI--KDTNVILQMDNNRDL-DLNSIIAEV

```
KAQYEDIANRSRAEAEAWYQSRYQELQVTATTHGEDLKVTRNEISEVSRVIQRLKAEIDS
VKKQIAGLQTAIADAEQRGELALKDARGKLADLQNALQTTKDELARLLRDYQELMNVKLS
LDIEIATYRKLLEGE
>Turtle_K78L3
EREQIKTLNNKFASFIDKVRFLEQQNKVLETKWNLLQQQPTSN--AGNNLEPAFEAYVGS
LRKHLDSLVGERGRQDSELKNMQDLVEDLKHKYEEEINRRMAAENEFVLLKKDLDAAYMK
KLEMEAKVAAMTDEINFLRALYETELAQMQRQI--SDTSVILSMDNNRAL-DLDGIIAEV
KAHYEDMANRSRAEANSMYQGKFQELQVTAGKHDNDLKNSKLEISELTRLIHRLQAELEN
VKKQCAKLQSAVAETEGRGELTLKDAREKLVELEMALQKAKEETARLLRDYQELLNIKLA
LDIEIATYKTLLEGE
>Turtle_K78L2
EKEQIKVLNNQFAVFIDKVRFLEQQNKVLVTKWELLQQQGGTI--IKKDLAPLFENFIQS
LRRRLDGLQNQKGQLQSELENMQQYVEEYKHKYEDEINKRTAAENEFVVLKKDVDNAYMV
KVELETRVQGLTDEINFLRFIFEEELSQLQTIS--RDLSVVVSMDNNRHL-DLDGIIQEV
RNQYEIIAQRSRAEAEAWYQSRYEELQSTAGRHGDNLRNTKTEIQELTRSIQRMRAEIES
VKKQIANLQSAIAEAEEKGELALKDARAKLDDLEHALQKDKADLANLLKEYQDLLNVKLA
LDIEIAMYRKLLEGE
>Turtle_K78L1
EREQMKTLNNKFVSFIDKVRFLEQQNKVLETKWKLLQEQGQGP--IKKSLDQLFEAYSNG
LRKQLDGLLNERRQLDTERKNFQDLVEDFKHKYEEEINKRTVVENDFVVLKRDLDVAYMS
KVELQVKLDSLTDEINFLRCLYEAELSQMQQTA--SDTSVVLSMDNNRNL-DLDSIIREV
KAQYEEIAQRSRAEAESCYQCKFEELQVTAGKHGDSLRDSKSEISELNRMIQRLQAEIEN
VKKQCERLQTSIADAEQQGELVLKDAKGKLAELEGALQKAKADLARQLREYQELMNVKLA
LDIEIATYRKLLEGE
>Turtle_K8L
EKEQIKTLNNKFAHFINKVRTLEQENQVLKTKWNILQGQKT----GPSNMEKMLKAYINS
LQKQLEDLGQQRVKLESDQGNMQDLVEEFKRKYEDEINNRSERENQFVLTKKDVDEAYIK
KQELEVKLQSLTDEINFLMELYAKELRELQADVEMSNNPVVISVDSSPKL-DVDSIIAEA
RVQYEKMAENSREEAENKYRITYEQIQEKAGQHGEELRAAKAEINELNWMIQRIRAEIAA
LKEQNAKLEANIVQAEEQGKLAVADATGKLQKMEHALQKAKEDMVVQLREYQALMNLKLA
LDIEIATYRKLLEGE
>Turtle_K8
EKEQIKTLNNKFASFIDKVRFLEQQNKMLETKWDLLQGQKT----TRSNMNNLFEAYINN
LRRQLDGLGQEKLKLEAELGNMQGLVEDFKNKYEDEINHRTEKENEFVLLKKDVDEAYMN
KVELESRLESLTDEINFLRQLYDEELRELQSQV--SDTSVVLTMDNNRKL-DLDGIIAEV
RAQYEEMANRSRAEAENMYKTKYEEIQAVAGKHGDDLRSTKTEINELNRMIHRLQSEIDA
LKGQRASLEAAITEAEERGEMAIKDASAKLAELEGALQRAKQDMARQLREYQELMNVKLA
LDIEIATYRKLLEGE
>Lizard_K80
EKEEIKTLNNQFASLIGKVQNLEQHNQVLLTRWNFLREQDNSL--SDLDIKLLYDQYMNR
LRLEIRSIDNEKEQLDAELDEVLDAMDNVRNSYEEEINKRTGMEFTFTALKKDLDNGFLH
KTELEAKLSGLHAWVELMKNIQEQELEEVMSQV--KDVSVVLGIDNNRYNPDPHRIVEDV
RAQYEALAIRSWEELEALTRSKLNEREVLSVKYGDHLLHDRRAIAELNIQIQKMRSCILS
LKSQCLRLEDNIKDVGLQGETALNDAKAKLAKLEEALHNAKQDLAQLVKQYQELMNIKLA
LDIEILTYRKLMEGE
>Lizard_K7
EKEQIKTLNNKFASFIDKVRFLEQQNKMLETKYSLLQEQKT----TKSNLAPIFETYISN
LRRQLDSLMNDKGRLEGELRNMQDLVEDFKSKYEDEINKRTTAENDFVVLKKDVDASYMN
KVELESKVDGLTDEVNFLREFYEAELRELHNQI--SDTSVVLQMDNNRNL-DLDSIIAEV
KAQYEEIANKSRAEAEAFYQTKYEALQVTAGKHGDDLRNTKTEISDLNRTIQRMQAEIEN
IKNQRGKLEAAITEAEERGELAVKDARAKLQELENALNKAKQEMARQLREYQELMNVKMS
LDIEIATYRKLLEGE
>Lizard_K84L1
EKNELQCLNSKFASFIDKVQFLEQQNLMLKTKWDFLQERKC----CKSNMEPMFKEYIAN
LKKEQECMECERAQLQAEMKNWREALEVNKKKFEEECHRRTCTENEYVSIKKEVDCVFMD
KSEKEAKVQALMKDLFFYKPTFYQEIHELQSCI--SDTCVTVQMDNSRGL-NMDCVMEVY
RRQFEEISSRSRAEAEAWCRCQYQELKTTAAKHCDNLRHVKEELSEITRVVHRLESEVSN
VKAQCTKLEEEVAAAEERGGIAVKDARCKLVELEDALHKAKQDMACQLREYQDLMNMKMG
LDIEITTYKKLLDGE
>Lizard_K84L2
EKNQLQCLNSKFACFIDKVRFLEQHNLMLKTKWDFLKEKKC----QKSNMDPMFHEYISR
LRKELERLQWERNQLQVEVNNWRDTMEGNKKKYEEEYNRRASAENEYVTLKKEVDYIFME
KSEKEAKVETLMQDIFFYKTTFEEEICEMQSSI--SDTCVTVKMDNSRDL-DVACIIEEF
RCRYEDIASRSRAEAEAWCQCQYQELKTTAAKHSDNLRNFKDELEELTRTVNRLQAEIAN
VKAQRCKLEEEVAGAEERGEMAVKDAKCKLFDLEDALHKAKQDMACQLREYQELMHLKLA
LDIEIATYRKLLEGE
>Lizard_K84L3
TKNELQSLNSKLASFINKVQLLEQHNLMLKTKWDFVQEMKQ----HRSDMEPLFDDHTSR
LKKELECLEREKKEMQIEHDSSAQTLEKNKSRYEEELNKRATAENGFVLLKKDLDFAFAD
KAELEAKVEKLAKHISFLKHIYAQEISELQNCI--SETCVMVQLDNRRAL-DMNRTIEEF
RRQHEYIASRTRAEAEAWLQHQYQELKTTAAKNNDNLNAVKEEIQALTRTAHQLESQITS
IKTQRCRLEDEVVEAKEHGETAVKDARCKLSDLEEALRKAKQDMTCQLREYQSLMNVKMA
MNIEIATYRKLIEGE
>Lizard_K84L4
EKEQLKTLNNKFASFIDKVRFLEQQNKVLETKWSLLQEQKR----VKSNIEPLFDGLIHK
LKKQLESLGCDRNKLESDLTSSRDILEDYRKRYEDECNRRTSAENEFVTLKKDVDCIYMH
KADLEGKVESLVEEVNFLRCLHDMELQELHSCI--SDTSVIVQMDNSREL-DLDGIIAEV
KAQYEDIANKSRYEAESWYQCKYEEMRATAGKHNDHLRDTKNEIMELNRVIQRLKAEIDT
AKGQRSKLEAAVAEAEERGEMALKDAKCKLTELEDALIKAKADMARQLREYQELMNVKLA
LDIEIATYRKMLEGE
>Lizard_K5L4
EKEQIKTLNNKFASFIDKVRFLEQQNKVLETKWSLLQDQKI----TRHNFDAMFEAYITN
LRRQLEGILGERPRLESELKNMQDTVEDFRIRYEDEINKRTAAENEFVVLKKDVDAAFMN
KVELEARVDSINDEATFLKSFYDAELAQMHAQV--SDTSVILQMDNSRNL-DLDSIIAEV
KAQYEDIANRSRAEAESWYQSKYEELQLSAGRHGDDLHNTKIEISELNRMIQRLHSEIDN
VKKQCAGLQASIADAEQRGELALKDARNKLAGLEEALTKAKADMARQLREYQELMNVKLA
LDIEIATYRKLLEGE
>Lizard_K5L3
EREQIKTLNNKFASFIDKVRFLEQQNKVLETKLTLLQEQGQKT--CKSNIEPLFEMYIND
LRKKLNNLENDRGRLLGELNNIQNLVEDCKNKYEDEINKRATAENDFVTLKKDVDAAYLN
KVELESKADSLNCEVNFLRALYEEELAQMHTQI--SDTSVVLSMDNNRNL-DMDSIIAEV
KAQYEDIANKSRAESETFYQCKYEELQTAAGRHGDDLRNTKQEISEINRHIHRLRAEIDN
VKKQCANLQAAIAEAEERGELALKDARQKLAELEDALQKAKQDMARQLKEYQDLMNVKLA
LDIEIATYRKLLEGE
>Lizard_K5L2
EREQIKTLNNKFASFIDKVRFLEQQNKVLETKWDLLQQQGTTC--QQSTMEPLFDVYITN
LRRQLEALTNERGRLDSELRNMQDMVEDYKNKYEDEINKRTGSENEFVVLKKDVDGAYMN
KIELAGKFESLNDEIEFLKAMYDAELSQMQQHV--SDTSVVLSMDNNRCL-DLNSIIAEV
KAQYEDIAQRSRTEAESWYQTKYEELQVSAGRHGDDLRNTKTEISELNRMIQRLRSEIDS
FKKQCANLQAAIAEAEDRGEMALKDAKHKLSELEEALQRTKQDLARQLKEYQELMNVKLA
LDIEIATYKKLLEGE
>Lizard_K5L1
EKEQIKTLNNKFASFIDKVRFLEQQNKVLETKWTLLQDQGFKTGANKGNLDPLFEAYINN
LRKQLNGLINEKGRLDGELRNMQDIVEDFKTKYEEEINRRTSAENEFVMLKKDVDAAYMN
```

```
KVELEAKVDSLTDELNFLRALYEAELAQLQAQM--SDTAVILSMDNNRDL-DLNGIVSEV
KAQYEDIANRSRAEAEAWYQTKFEELQATAGRHGDDLRNTKGEISELNRLIQRIRAEIEN
ARNQCANMQTAIADAEDRGELALKDARAKLIELEEALQKAKEEMTRLLREYQELMNVKLA
LDIEIATYRKLLEGE
>Lizard_HBS1
EKEQIKDLNNQFACFIDKVRYLEERNQALATKWELLKSQNPA---VKKDLSPLCENYITS
MRRKLDNLLCEKNQLQQEHKTMLDLIEANRCKYEEEVKRRTNAENEFIVLKQDVDGIFKK
QKELEQKKEVLEENIGFLTNFFSKERAILESQL--NNTSVVVNMDNSRCL-DMDGLIQEI
ESWYQGIVQKSKGEANLFYWNQMEDLQSKRSQFYEKLRQNNADIAELNRYVQIMRCQVGT
EKQKVASLHAALRETKELGDHALQDAESKYKDLQNRVQTSKDRLVALVRDYQDLMNTKLA
LDIEIATYKTLLEGE
>Lizard_HBS2
EKEEMKTLNNQFACFIDKVRSLEQQNKVLGTKWELLNQCVQPA---KKNLEGYYENFIAT
LKKQLECLLSERGKLEHEQKNMQELVEEYRSKYEEEVNRRTTAENEFVVLKKDVDCVFLT
KEELESKVDVLAQELEVLRCVFAEELTQLDSQI--LDTSVILKMDNTRDL-HMDLILQNV
ENWYQNIAHNSKQEAEAFYHNKIAEIQNNRSKFNEELKCNQHEIAELNRVTQRLRTDLGN
AKKQVSNLQSAICDAEQRGDIALKDARNKHAELQNAYQQSKDKLASLLRDYQDLLNTKMA
LDIEIATYKMMLEGE
>Lizard_HBS3
EREEMKNLNNQFACFIEKVRILEQENKALETKWNLLQQYAMV---PRRNLEPYYENFISN
MKYQIDCLLHTREHLANDKNAIQELVEDFKSKYEEEFKRRTTAENEFVLLKKDVDSISLS
KTELEGKMNLLRWELEFRKSVYMEELMQLSGQI--SDMNILVQMDNNRDL-DMDSIIKNV
EAWYQSIAHRSKEEVNALYADRFQELQEQRGKYLNDLKINQYEIEELTRAVHTLQSDHSN
VKKQVDALQASICDVDQRGDQALKDAQEKHSDLLSALQKAKDDLAGMLKDYHDLLNVKLA
LDIEIATYRTLLEGE
>Lizard_K78LT
EREQIKTLNDQFACFIDKVRHLEQQNKLLETKWSCLQQQEPM---EKQNTEHLYQNYIVT
LKKQLDLLLDEREQLKLEQVKFQDVVEEYKSRYEGVINRRLGAENEFVVLKKDVDCAYTS
KVELEVKVDSLRQEQDFHRCVYDTELESL-ATA--SDTNVVVSMDNSRDL-DMEGIIESV
RCQYEEIAQRSKDEVNALYETKYKELQTTWGHHCNSLSSSRHEIQELTRLIQRYKADVEN
GKKQVEALQVAVADHEQQGDCTLKDAKEKLAEVEKALQSTKDELARLLRDYQELLNVKVA
LDIEIAMYKSLLEGE
>Lizard_K78L5
EREQIKTLNNKFATFIDKVRFLEQQNKVLETKWDLLYQQGTPA--LRDNLEPLYEAYIDT
LRRQFESLTNDKNHLDIELKNTQELVEDFKSKYEDEINKRTACENDFVVLKKDVDGSYMH
KIDLGSKADSLTQHIQFLRALFETELSQVQSQV--RDTNVVLQMDNNRDL-DLNGIIAEV
RAQYEDIANRSRAEAETWYQSKYQELQTTAQKHCDELKTTKDDIAEMSRVVHKLRAEIEI
VKKAVNGLQSSIAESEQRGEIALKDAQKKLLDLQSALTSSKDELARLLRDYQELMNVKLA
LDVEIATYRTLLEGE
>Lizard_K78L4
EREQIKTLNNKFASFIDKVRFLEQQNKVLETKLNLLQQQGSTA--TTIHIDPMFEAYINN
LRNQLDGLVGDRGRLDGELRNTQDLVEDYRKRYEDEINKRTTAENEFVDIKKNVDGGFMH
KIELGAKADGLTDEINFLRALHEAELSQIQGRI--SDTNIVLQMDNNRDI-DLNSIISEV
KAQYEDIANRSRAEAEALYQSKFQELQSTAVNHGDDLKNTKNEIAELSRIIQRMKAEIDN
VKKQIAGLQTAIADAEQRGELALKDARVKLTDLQHALQSAKDELARLLRDYQELMNVKLA
LDVEIATYRTLLEGE
>Lizard_K78L3
EREQIKCLNDKFASFIDKVRFLEQQNKILETKLNLLQQQPMPH--LKRKADHTFEAYIHG
LRKQLESLSTERGRLGSEWKNMQDLVEDFKRRYEEEINKRTTAENEFVLLKKDVDLAYMN
KMELKTRTASLDKEVDFLRSLYEEEQAQMQSQV--TDTSIILSMDNSRDL-DMDVIIAEV
KAQYEDMANRSRAEVDAMYQSKFQELQLTAGKHDDALKNSKTEILELNRLIHRLRAETGT
VKKQCAFLQSSIAEAEERGETALKDAREKLTKLENATQKSNEELVRLLRDYQELLNVKIS
LDIEIATYKTLLEGE
>Lizard_K78L2
EKEQIKLLNNQFANFIDKVRFLEQQNKVLQTKWQILQQQTQGRG-PMQNLDGEYQLFLNG
LRNQIDQIRSQKQQLMSELQNMQQVVEDSKNKYEQEINQRTTSENEFVVLKKDVDSAYMT
KVDLDAKVGGIVEQINFFRRIFDIELSQIPIGG--KDTSVVVNMDDSRDL-NLEGMIDEV
RAQYEEVARSSRAEAETWYRSQYEALQNAAGRHGENLQNTRQEIQELTRNIQRLRTEIEH
AKKQAAQMQSAIAEAEERGEMALRDAKQKLDELEGSLTKDKEVLARLLKEYQDLLNVKLA
LDMEIAMYRKLLEGE
>Lizard_K78L1
EKEQMKTLNNKFASFIDKVRFLEQQNKVLETKYKLLQEQGPGA--IQKHLDPMYEAYINN
LRRQLECLQNEKRGLDSELKNFQDMVEDYKTKYEEEINKRTQAENDFVILKKDVDAAYMN
KVELQAKCDALIDEINFMKCLYEAELSQMQQTV--SDTSVILQMDNNRCL-DMDSIIAEV
KARYEEIAQMGRIDAESFYQSKYEELQASVGKHGDNLQKTKAEISEHNRMIQRIRAEIDN
VKKQCEKLKSSIAEAEERGELALKDARNKLTELEEALQKAKEEMTRLLKEYQELMSCKLA
LDVEIATYKKLLEGE
>Lizard_K8
EKEEIKSLNNKFAGFIDKVRFLEQQNKMLDTKWSLLQNQKI----TRSNMDSMFEAYINN
LRRQLDGLGQERVRLDSELGNMQGLVEEFKSKYEDEINHRTEKENDFVLLKKDVDEAYMN
KIELESRLESLTDELNFLRQLHEEELRVLQSQI--SDTSVVLSMDNNRSL-DLDGIIAEV
KAQYEEVAARSRAEAESTYKSKYEELQSVAGKHGDDLRITKNEISELNRLITRIQSEIDG
LKGQRANLESAIAEAEERGELAVKDATAKLSELEAALQRAKQDMARQLREYQELMNVKLA
LDIEIATYRKLLEGE
>SnakeP_K80
EKEEIKTLNNQFAALIGKVQSLEQHNQVLLTRWNFLKEQDSSL--SDLDIKLLYDRYMNK
LNLEIRSIDVEKEQLDSELDEVLASMDVFRSKFEEEINKRSGLEFTFTTLKKDLDNGFLH
KTELETKLSGLHAWVELMTTIQEQELEEVMSQI--KDVSVVLGIDNNRYMPDPHRIVEDV
RAQYEDLAIRSWEELEALTRSKLNEREVLSAKYGDHLLNDRRVIAELNIQIQKMRSCIVS
LKSQCLQLEDNIKNVGLQGETALNDAKAKLAKLEDALHSARQDLAQLVKEYQELMNIKLA
LDIEILTYRKLVEGE
>Snake_K7
EKEQIMVLNNKFASFIDKVRFLEQQNKMLETKWSILQQQKTST---RNNTAPVFDAYINN
LRRQLDSLQNDKGRLEGELKNMQDLVEDFKNKYEDEINKRTTAENDFVLIKKDVDASYMN
KVELEARVDGLTDEINFLRALYDAELAELQNQV--SDTSVILQMDNNRNL-DLDSIIAEV
KAQYEEIANKSRIEAESWYQSKFEVLQATAGKHGDDLRSTRNEIAEINRLILRLQADIDN
VKNQRAKLESAIAEAEERGTLAVKDAKGKLEELEVAMNKAKQEMAHQLREYHELMNVKLG
LDIEIATYRKLLEGE
>Snake_K5L2
EGEQIKILNNKFASFIDKVRFLEQQNKVLETKWTLLQKHDQKT--VMSNVEPLFEAYINN
LRTQVNDLEKEKVKLDGELHTTQNLVEDYKSKYEDEINKRTDAENDFVILKKEVDAAYMN
KADLETRLQSLVDETDFLRLLHETELADLQRQI--SDTSVVLSMDNNRYL-DLDSIIAEV
KAQYEDIAKKSRAEAEAWYQSKFEELQGTAGRHSDDVRNAKHEISELNRHVQRIKAEIES
VKKQCANLQAKIAEAEDRGEQALKDARQKLAELEDGLQKAKQNMAKLLKDYQELLNVKMA
LDVEIATYRKLLEGE
>Snake_K5L1
EREQIKTLNNKFASFIDKVRFLEQQNKVLETKWELLQRQGTQV--QQRNLEPLFENYIAN
LRKQFDYLVNERGRLDMELRNMQDMVEDFKNKYEDEINKRTGAENEFVVLKKDVDGAYMN
KIELASKSDSLNDEIEFLKALFEAELSQMQQQV--SDTSVVLSMDNNRSL-DLNSIIAEV
KAQYEDIANRSRTEAESWYQSKYEELQVSAGRHGDDLRNTKTEISELNRVIQRLRSEIDA
YKKQCANIQAAIAEAEDRGELALKDAKQKLNELEECLQKTKQELARQLKEYQDLMNIKLA
LDIEIATYRKMLEGE
>Snake_HBS1
EMEQMKSLNSKFAGFIDKVQRLEQKNQLLETKWAFLQQQTKP---PTESLECMFEQYIAS
```

```
LKKELEYLQYEKENLQNDRESAKAQTADCKCKYEEKVKQHVAAEKEIIELRKDVDYIFSN
NAELERKVSLLDREIKFLKCVYKKEKEQLDKQD--QDVDVVVKMDNSRNL-DMDNIIAMV
RKQYEEIMQRSKAEADKFYQNKYEEAQSKSSSYKWDLKNREQQISELIQQVEKVQGEIQS
LQKENEELKKTICEAAKGGEQASKEGQQKYAELVKALQNGKDELASLVRDYQKVLNVKIV
LDIEIAAYKCLLEGE
>Snake_HBS2
EKDQMKDLNTQFASFIEKVRSLEQRNRILITKWELLQNQKMPK--VKKDLSTLFDNYIAN
LRKKIDYMFNEKNKLENQQRMMQDLIEEYQCRYKEENNRKEHLENEFILLKQQVDNEFKE
QKERELKRELLMENKEFLKYFFTEEHAILNCHP--PDTSVVLNMDNSRGL-DMDVLIKNI
DSWYHNIAQRSKEEANLFYQNQIEDLQNKKCQCRESLQKTSDEITELNRLVHLMQSKLDN
EKKKTAALQIAIGDIEHKGDHALKDARAKQTELQNRIQNSKDQLAVILRDYQDLMNTKLA
LDIEIATYNTMLEGE
>Snake_HBS3
EKEEMKILNNQFACFIDKVRSLEQQNKVLSTKWELLNQYVQPT---RKNLEAYYGNFIGS
LKKQLECVLTEREKLEHEQKNMQELVEEYRSRYEDEVNRRTTAENDFVVLKKDVDCVFLT
KEELESKVDVLAQELEVLRCIFAEELTQFNHQI--CDTSVVLKMDNTRAL-DMDLILKNV
EAWYQNIAHTSKQEAEAFYHSKIAEIQNNRGKFNEELKNNQHEIAELNRVVQRLQADTDN
AKKQVSSLQSAICDSEQRGDIALKDARTKHLELQTAYQQSKDKLACLLRDYQELLNTKLA
LDIEIATYKALLEGE
>Snake_HBS4
EREEMKSLNNQFASFIDKVRNLELQNKVLETKWNLLQEHVIP---AKKSLEPYYENFISN
MKKQLECLLNNQEHLRNENDGIEQLVEDFKCNYEEEFKRRTDAENEFVLLKKDVDSISLS
KSELEGKVHLLSGELEFRRGIYAEELAQLSTNV--YDTNILLQMDNSRNL-NIDSIIKSV
EAWYKNVAQGSKEEVNAFYENRFQELQQQRGKYSQTLKINQQEIADLTRLVHKLQSEYDI
IKKQVNGFQTSVCEIEQHGDCVLKDARDKHIDLQTAVQKAKDDLARLLRDYQELLNTKLA
LDIEIATYKTLLEGE
>Snake_K78LT
EREQIKTLNDKFVSFIDKVKHLEQQNKLLETKWNCLQQQEPV---EKANIQPLFENYITS
LKQQLQRLLNEREQLQLEQSKFQDMVEEFKSRYEEEINHRMSAENNFVLLKKDVDIAYSN
KVDLEVKVESLRQELNFIRCVFEAEIESLQAAN--AETNVIVSMDNSRVL-DMEGIIHSV
RSQYEEIAQKSKDEVNALYENKYKELQNTWGNYCNNLSSGRHEIQELTRMVQRCRSEVES
TKKQIDALQTSIADNEQRGESALKDAKLKLTEVENNLQSSKDELARLLRDYQNVLNVKIA
LDIEIAMYKSLLEGE
>Snake_K78L4
EREQIKTLNNKFASFIDKVRFLEQQNKVLETKWDLLCQQGSPA--LRDNLEPIYEAFIEA
LRRQFDSLCNDKNHLDIELKNTQELVEDFKNKYEDEINKRTACENDFVVLKKDVDGSYMH
KIDLGSKADSLTQYIQFLRALFDAELTQLHSQV--KETNVILQMDNNRDL-DLDSIIAEV
KAQYEDIANRSRAEAEAWYQTKFQELQSTAFKHGDDLKTTKDEIADLSRIVQRIRSEIEI
VKKAISSLQSSLGEAEQRGELALKDARTKLLDLQTALTSAKDELARLLRDYQELMNVKLA
LDVEIATYRTLLEGE
>Snake_K78L3
EREQIKTLNNKFASFIDKVRFLEQQNKVLETKWNLLQQHGSSV--PKLSLEPLFENYILN
LRRQLDSLIGNRAKLDSDLRNMQDLVEDYRRKYEEEINKRTTAENDFVGIKKDVDVSFMQ
KVDLQAKVDRLIDEINFLKALHEAELIQIQGQI--SDTNVVLQMDNNRDL-DLNSIIAEV
KAQYEDIANRSRAETEAWYQNKFQELQIIAVGHGDDLKTIKNEIRELNRLINRLKAETEN
VIKTISRIQKAIADAEERGQLALKDAQVKLNDLQQAIQNSKDELARLLRDYQELMNVKLA
LDIEIATYRTLLEGE
>Snake_K78L2
EKEQIKVLNNQFASFIDKVRFLEQQNKVLQTKWQILQRQSQGIG-PTMNLDADFQQFLNS
LRSQIENIRSQKGQLTTELQSMQHYVEEYKNKYEEEINRRTTAENDFVVLKKDVDCAYLS
KSNLDSRVGALADQINFLRRIFEVEISQYQTGG--QDTSVVVSMDNNTHL-NLEGIIDEV
RSQYEEIARSSRAEAESWYHSQYEALQNTAGRHGDNLRNTRQEIQELTRNIQRLRSEIEH
VKKQXXX-----------------------------------------------------
---------------
>Snake_K78L1
EREQIKTLNNKFASFIDKVRFLEQQNKVLETKWKLLQEQGQGTG-GQKNLDHFFEAYISN
LRRQLDGLLNERRGFESELKNFQDLVEDFKNKYEEEINKRTAAENDFVLLKKDVDAAYMN
KVELQAKMDALTDEINFLRCMFEAELNQMQQSV--SDTSVVLQMDNNRSL-DLNSIIAEV
KAQYEDIAQKSRAEAESWYQCKYEELQVSAGRHGDNLRDTKTEIAELNRTIQRMRAEIEN
VKKQCEKLQASIAEAEERGELALKDAREKLSELEEALQKSKEEMARLLKEYQELMNVKLA
LDIEIATYRKLLEGE
>Snake_K8
EKEEIKTLNNKFANLIDKVRFLEQQNKMLETKWSLLQNQKT----TRSNMDSMFEAYINN
LRRQLENLGQERARLDAELGNMQGLVEEFKNKYEDEINHRTEKENEFVLLKKDVDEAYMN
KVELESRMECLTDEINFLRQFYDEELRVLQSQI--SDTSVILSMDNNRSL-DLDGIITDV
KAQYEEIANRSRAEAERMYQIKYEELQTVAGKHGDDLRSTKNEINELSRMINRIQSEIDG
LKGQRASLEAAITEAEERGELAVKDANNKLAELESAMQRAKQDMARQLREYQELLNTKLA
LDIEIATYRKLLEGE
>Human_K1
EREQIKSLNNQFASFIDKVRFLEQQNQVLQTKWELLQQVDTST--RTHNLEPYFESFINN
LRRRVDQLKSDQSRLDSELKNMQDMVEDYRNKYEDEINKRTNAENEFVTIKKDVDGAYMT
KVDLQAKLDNLQQEIDFLTALYQAELSQMQTQI--SETNVILSMDNNRSL-DLDSIIAEV
KAQYEDIAQKSKAEAESLYQSKYEELQITAGRHGDSVRNSKIEISELNRVIQRLRSEIDN
VKKQISNLQQSISDAEQRGENALKDAKNKLNDLEDALQQAKEDLARLLRDYQELMNTKLA
LDLEIATYRTLLEGE
>Human_K2
EREQIKTLNNKFASFIDKVRFLEQQNQVLQTKWELLQQMNVGT--RPINLEPIFQGYIDS
LKRYLDGLTAERTSQNSELNNMQDLVEDYKKKYEDEINKRTAAENDFVTLKKDVDNAYMI
KVELQSKVDLLNQEIEFLKVLYDAEISQIHQSV--TDTNVILSMDNSRNL-DLDSIIAEV
KAQYEEIAQRSKEEAEALYHSKYEELQVTVGRHGDSLKEIKIEISELNRVIQRLQGEIAH
VKKQCKNVQDAIADAEQRGEHALKDARNKLNDLEEALQQAKEDLARLLRDYQELMNVKLA
LDVEIATYRKLLEGE
>Human_K3
EREQIKTLNNKFASFIDKVRFLEQQNKVLETKWNLLQQQGTSSISGTNNLEPLFENHINY
LRSYLDNILGERGRLDSELKNMEDLVEDFKKKYEDEINKRTAAENEFVTLKKDVDSAYMN
KVELQAKVDALIDEIDFLRTLYDAELSQMQSHI--SDTSVVLSMDNNRSL-DLDSIIAEV
RAQYEDIAQRSKAEAEALYQTKLGELQTTAGRHGDDLRNTKSEIIELNRMIQRLRAEIEG
VKKQNANLQTAIAEAEQHGEMALKDANAKLQELQAALQQAKDDLARLLRDYQELMNVKLA
LDVEIATYRKLLEGE
>Human_K4
EREQIKLLNNKFASFIDKVQFLEQQNKVLETKWNLLQQQTTTT--SSKNLEPLFETYLSV
LRKQLDTLGNDKGRLQSELKTMQDSVEDFKTKYEEEINKRTAAENDFVVLKKDVDAAYLN
KVELEAKVDSLNDEINFLKVLYDAELSQMQTHV--SDTSVVLSMDNNRNL-DLDSIIAEV
RAQYEEIAQRSKAEAEALYQTKVQQLQISVDQHGDNLKNTKSEIAELNRMIQRLRAEIEN
IKKQCQTLQVSVADAEQRGENALKDAHSKRVELEAALQQAKEELARMLREYQELMSVKLA
LDIEIATYRKLLEGE
>Human_K5
EREQIKTLNNKFASFIDKVRFLEQQNKVLDTKWTLLQEQGTKT--VRQNLEPLFEQYINN
LRRQLDSIVGERGRLDSELRNMQDLVEDFKNKYEDEINKRTTAENEFVMLKKDVDAAYMN
KVELEAKVDALMDEINFMKMFFDAELSQMQTHV--SDTSVVLSMDNNRNL-DLDSIIAEV
KAQYEEIANRSRTEAESWYQTKYEELQQTAGRHGDDLRNTKHEISEMNRMIQRLRAEIDN
VKKQCANLQNAIADAEQRGELALKDARNKLAELEEALQKAKQDMARLLREYQELMNTKLA
LDVEIATYRKLLEGE
>Human_K6B
```

```
EREQIKTLNNKFASFIDKVRFLEQQNKVLDTKWTLLQEQGTKT--VRQNLEPLFEQYINN
LRRQLDNIVGERGRLDSELRNMQDLVEDLKNKYEDEINKRTAAENEFVTLKKDVDAAYMN
KVELQAKADTLTDEINFLRALYDAELSQMQTHI--SDTSVVLSMDNNRNL-DLDSIIAEV
KAQYEEIAQRSRAEAESWYQTKYEELQITAGRHGDDLRNTKQEIAEINRMIQRLRSEIDH
VKKQCANLQAAIADAEQRGEMALKDAKNKLEGLEDALQKAKQDLARLLKEYQELMNVKLA
LDVEIATYRKLLEGE
>Human_K6C
EREQIKTLNNKFASFIDKVRFLEQQNKVLDTKWTLLQEQGTKT--VRQNLEPLFEQYINN
LRRQLDSIVGERGRLDSELRNMQDLVEDLKNKYEDEINKRTAAENEFVTLKKDVDAAYMN
KVELQAKADTLTDEINFLRALYDAELSQMQTHI--SDTSVVLSMDNNRNL-DLDSIIAEV
KAQYEEIAQRSRAEAESWYQTKYEELQVTAGRHGDDLRNTKQEIAEINRMIQRLRSEIDH
VKKQCASLQAAIADAEQRGEMALKDAKNKLEGLEDALQKAKQDLARLLKEYQELMNVKLA
LDVEIATYRKLLEGE
>Human_K6A
EREQIKTLNNKFASFIDKVRFLEQQNKVLETKWTLLQEQGTKT--VRQNLEPLFEQYINN
LRRQLDSIVGERGRLDSELRGMQDLVEDFKNKYEDEINKRTAAENEFVTLKKDVDAAYMN
KVELQAKADTLTDEINFLRALYDAELSQMQTHI--SDTSVVLSMDNNRNL-DLDSIIAEV
KAQYEEIAQRSRAEAESWYQTKYEELQVTAGRHGDDLRNTKQEIAEINRMIQRLRSEIDH
VKKQCANLQAAIADAEQRGEMALKDAKNKLEGLEDALQKAKQDLARLLKEYQELMNVKLA
LDVEIATYRKLLEGE
>Human_K7
ESEQIKTLNNKFASFIDKVRFLEQQNKLLETKWTLLQEQKSAK---SSRLPDIFEAQIAG
LRGQLEALQVDGGRLEAELRSMQDVVEDFKNKYEDEINHRTAAENEFVVLKKDVDAAYMS
KVELEAKVDALNDEINFLRTLNETELTELQSQI--SDTSVVLSMDNSRSL-DLDGIIAEV
KAQYEEMAKCSRAEAEAWYQTKFETLQAQAGKHGDDLRNTRNEISEMNRAIQRLQAEIDN
IKNQRAKLEAAIAEAEERGELALKDARAKQEELEAALQRGKQDMARQLREYQELMSVKLA
LDIEIATYRKLLEGE
>Human_K8
EKEQIKTLNNKFASFIDKVRFLEQQNKMLETKWSLLQQQKT----ARSNMDNMFESYINN
LRRQLETLGQEKLKLEAELGNMQGLVEDFKNKYEDEINKRTEMENEFVLIKKDVDEAYMN
KVELESRLEGLTDEINFLRQLYEEEIRELQSQI--SDTSVVLSMDNSRSL-DMDSIIAEV
KAQYEDIANRSRAEAESMYQIKYEELQSLAGKHGDDLRRTKTEISEMNRNISRLQAEIEG
LKGQRASLEAAIADAEQRGELAIKDANAKLSELEAALQRAKQDMARQLREYQELMNVKLA
LDIEIATYRKLLEGE
>Human_K71
EREQIKALNNKFASFIDKVRFLEQQNQVLETKWELLQQLDLNN--CKNNLEPILEGYISN
LRKQLETLSGDRVRLDSELRNVRDVVEDYKKRYEEEINKRTAAENEFVLLKKDVDAAYAN
KVELQAKVESMDQEIKFFRCLFEAEITQIQSHI--SDMSVILSMDNNRNL-DLDSIIDEV
RTQYEEIALKSKAEAEALYQTKFQELQLAAGRHGDDLKNTKNEISELTRLIQRIRSEIEN
VKKQASNLETAIADAEQRGDNALKDARAKLDELEGALHQAKEELARMLREYQELMSLKLA
LDMEIATYRKLLESE
>Human_K72
EREQIKALNNKFASFIDKVRFLEQQNQVLETKWNLLQQLDLNN--CRKNLEPIYEGYISN
LQKQLEMLSGDGVRLDSELRNMQDLVEDYKKRYEVEINRRTAAENEFVVLKKDVDAAYMN
KVELQAKVDSLTDEIKFFKCLYEGEITQIQSHI--SDTSIVLSMDNNRDL-DLDSIIAEV
RAQYEEIALKSKAEAETLYQTKIQELQVTAGQHGDDLKLTKAEISELNRLIQRIRSEIGN
VKKQCADLETAIADAEQRGDCALKDARAKLDELEGALHQAKEELARMLREYQELVSLKLA
LDMEIATYRKLLESE
>Human_K73
EREQIKVLNNKFASFIDKVRFLEQQNQVLETKWELLQQLDLNN--CKNNLEPILEGYISN
LRKQLETLSGDRVRLDSELRSVREVVEDYKKRYEEEINKRTTAENEFVVLKKDVDAAYTS
KVELQAKVDALDGEIKFFKCLYEGETAQIQSHI--SDTSIILSMDNNRNL-DLDSIIAEV
RAQYEEIARKSKAEAEALYQTKFQELQLAAGRHGDDLKHTKNEISELTRLIQRLRSEIES
VKKQCANLETAIADAEQRGDCALKDARAKLDELEGALQQAKEELARMLREYQELLSVKLS
LDIEIATYRKLLEGE
>Human_K74
EREQIKVLNDKFASFIDKVRFLEQQNQVLETKWELLQQLDLNN--CKKNLEPILEGYISN
LRKQLETLSGDRVRLDSELRSMRDLVEDYKKRYEVEINRRTTAENEFVVLKKDADAAYAV
KVELQAKVDSLDKEIKFLKCLYDAEIAQIQTHA--SETSVILSMDNNRDL-DLDSIIAEV
RMHYEEIALKSKAEAEALYQTKIQELQLAASRHGDDLKHTRSEMVELNRLIQRIRCEIGN
VKKQRASLETAIADAEQRGDNALKDAQAKLDELEGALHQAKEELARMLREYQELMSLKLA
LDMEIATYRKLLEGE
>Human_K75
EREQIKTLNNKFASFIDKVRFLEQQNKVLETKWALLQEQGSRT--VRQNLEPLFDSYTSE
LRRQLESITTERGRLEAELRNMQDVVEDFKVRYEDEINKRTAAENEFVALKKDVDAAYMN
KVELEAKVKSLPEEINFIHSVFDAELSQLQTQV--GDTSVVLSMDNNRNL-DLDSIIAEV
KAQYEDIANRSRAEAESWYQTKYEELQVTAGRHGDDLRNTKQEISEMNRMIQRLRAEIDS
VKKQCSSLQTAIADAEQRGELALKDARAKLVDLEEALQKAKQDMARLLREYQELMNIKLA
LDVEIATYRKLLEGE
>Human_K76
EREQIKTLNNKFASFIDKVRFLEQQNKVLETKWELLQQQTTGS--GPSSLEPCFESYISF
LCKQLDSLLGERGNLEGELKSMQDLVEDFKKKYEDEINKRTAAENEFVGLKKDVDAAFMN
KVELQAKVDSLTDEVSFLRTLYEMELSQMQSHA--SDTSVVLSMDNNRCL-DLGSIIAEV
RAQYEEIAQRSKSEAEALYQTKLGELQTTAGRHGDDLRNTKSEIMELNRMIQRLRAEIEN
VKKQNANLQTAIAEAEQRGEMALKDANAKLQDLQTALQKAKDDLARLLRDYQELMNVKLA
LDVEIATYRKLLEGE
>Human_K77
EREQIMVLNNKFASFIDKVRFLEQQNQVLQTKWELLQQVNTST--GTNNLEPLLENYIGD
LRRQVDLLSAEQMRQNAEVRSMQDVVEDYKSKYEDEINKRTGSENDFVVLKKDVDAAYVS
KVDLESRVDTLTGEVNFLKYLFLTELSQVQTHI--SDTNVILSMDNNRSL-DLDSIIDAV
RTQYELIAQRSKDEAEALYQTKYQELQITAGRHGDDLKNSKMEIAELNRTVQRLQAEISN
VKKQIEQMQSLISDAEERGEQALQDAWQKLQDLEEALQQSKEELARLLRDYQAMLGVKLS
LDVEIATYRQLLEGE
>Human_K78
ETQEIRTLNNQFASFIDKVRFLEQQNKVLETKWHLLQQQGLSG--SQQGLEPVFEACLDQ
LRKQLEQLQGERGALDAELKACRDQEEEYKSKYEEEAHRRATLENDFVVLKKDVDGVFLS
KMELEGKLEALREYLYFLKHLNEEELGQLQTQA--SDTSVVLSMDNNRYL-DFSSIITEV
RARYEEIARSSKAEAEALYQTKYQELQVSAQLHGDRMQETKVQISQLHQEIQRLQSQTEN
LKKQNASLQAAITDAEQRGELALKDAQAKVDELEAALRMAKQNLARLLCEYQELTSTKLS
LDVEIATYRRLLEGE
>Human_K79
EREQIKTLNNKFASFIDKVRFLEQQNKVLETKWALLQEQGQNLGVTRNNLEPLFEAYLGS
MRSTLDRLQSERGRLDSELRNVQDLVEDFKNKYEDEINKHTAAENEFVVLKKDVDAAYMG
RMDLHGKVGTLTQEIDFLQQLYEMELSQVQTHV--SNTNVVLSMDNNRNL-DLDSIIAEV
KAQYELIAQRSRAEAEAWYQTKYEELQVTAGKHGDNLRDTKNEIAELTRTIQRLQGEADA
AKKQCQQLQTAIAEAEQRGELALKDAQKKLGDLDVALHQAKEDLTRLLRDYQELMNVKLA
LDVEIATYRKLLESE
>Human_K80
EKEEMKALNDKFASLIGKVQALEQRNQLLETRWSFLQGQDSAI----FDLGHLYEEYQGR
LQEELRKVSQERGQLEANLLQVLEKVEEFRIRYEDEISKRTDMEFTFVQLKKDLDAECLH
RTELETKLKSLESFVELMKTIYEQELKDLAAQV--KDVSVTVGMDSRCHI-DLSGIVEEV
KAQYDAVAARSLEEAEAYSRSQLEEQAARSAEYGSSLQSSRSEIADLNVRIQKLRSQILS
VKSHCLKLEENIKTAEEQGELAFQDAKTKLAQLEAALQQAKQDMARQLRKYQELMNVKLA
LDIEIATYRKLVEGE
```

```
>Human_K81
EKEQIKSLNSRFAAFIDKVRFLEQQNKLLETKLQFYQNREC----CQSNLEPLFEGYIET
LRREAECVEADSGRLASELNHVQEVLEGYKKKYEEEVSLRATAENEFVALKKDVDCAYLR
KSDLEANVEALIQEIDFLRRLYEEEILILQSHI--SDTSVVVKLDNSRDL-NMDCIIAEI
KAQYDDIVTRSRAEAESWYRSKCEEMKATVIRHGETLRRTKEEINELNRMIQRLTAEVEN
AKCQNSKLEAAVAQSEQQGEAALSDARCKLAELEGALQKAKQDMACLIREYQEVMNSKLG
LDIEIATYRRLLEGE
>Human_K82
EKEQIKCLNNRFASFINKVRFLEQKNKLLETKWNFMQQQRC----CQTNIEPIFEGYISA
LRRQLDCVSGDRVRLESELCSLQAALEGYKKKYEEELSLRPCVENEFVALKKDVDTAFLM
KADLETNAEALVQEIDFLKSLYEEEICLLQSQI--SETSVIVKMDNSREL-DVDGIIAEI
KAQYDDIASRSKAEAEAWYQCRYEELRVTAGNHCDNLRNRKNEILEMNKLIQRLQQETEN
VKAQRCKLEGAIAEAEQQGEAALNDAKCKLAGLEEALQKAKQDMACLLKEYQEVMNSKLG
LDIEIATYRRLLEGE
>Human_K83
EKEQIKSLNSRFAAFIDKVRFLEQQNKLLETKLQFYQNREC----CQSNLEPLFAGYIET
LRREAECVEADSGRLASELNHVQEVLEGYKKKYEEEVALRATAENEFVALKKDVDCAYLR
KSDLEANVEALIQEIDFLRRLYEEEIRILQSHI--SDTSVVVKLDNSRDL-NMDCIVAEI
KAQYDDIATRSRAEAESWYRSKCEEMKATVIRHGETLRRTKEEINELNRMIQRLTAEVEN
AKCQNSKLEAAVAQSEQQGEAALSDARCKLAELEGALQKAKQDMACLIREYQEVMNSKLG
LDIEIATYRRLLEGE
>Human_K84
EKEQIKTLNNKFASFIDKVRFLEQQNKLLETKWSFLQEQKC----IRSNLEPLFESYITN
LRRQLEVLVSDQARLQAERNHLQDVLEGFKKKYEEEVVCRANAENEFVALKKDVDAAFMN
KSDLEANVDTLTQEIDFLKTLYMEEIQLLQSHI--SETSVIVKMDNSRDL-NLDGIIAEV
KAQYEEVARRSRADAEAWYQTKYEEMQVTAGQHCDNLRNIRNEINELTRLIQRLKAEIEH
AKAQRAKLEAAVAEAEQQGEATLSDAKCKLADLECALQQAKQDMARQLCEYQELMNAKLG
LDIEIATYRRLLEGE
>Human_K85
EKEQIKSLNSRFAAFIDKVRFLEQQNKLLETKWQFYQNQRC----CESNLEPLFSGYIET
LRREAECVEADSGRLASELNHVQEVLEGYKKKYEEEVALRATAENEFVVLKKDVDCAYLR
KSDLEANVEALVEESSFLRRLYEEEIRVLQAHI--SDTSVIVKMDNSRDL-NMDCIIAEI
KAQYDDVASRSRAEAESWYRSKCEEMKATVIRHGETLRRTKEEINELNRMIQRLTAEIEN
AKCQRAKLEAAVAEAEQQGEAALSDARCKLAELEGALQKAKQDMACLLKEYQEVMNSKLG
LDIEIATYRRLLEGE
>Human_K86
EKEQIKSLNSRFAAFIDKVRFLEQQNKLLETKLQFYQNREC----CQSNLEPLFEGYIET
LRREAECVEADSGRLASELNHVQEVLEGYKKKYEEEVSLRATAENEFVALKKDVDCAYLR
KSDLEANVEALIQEIDFLRRLYEEEIRVLQSHI--SDTSVVVKLDNSRDL-NMDCIIAEI
KAQYDDIVTRSRAEAESWYRSKCEEMKATVIRHGETLRRTKEEINELNRMIQRLTAEVEN
AKCQNSKLEAAVAQSEQQGEAALSDARCKLAELEGALQKAKQDMACLIREYQEVMNSKLG
LDIEIATYRRLLEGE
```

Suppl. Fig. S13. Amino acid sequence alignment of the intermediate filament domains of type II keratins used for the phylogenetic analysis shown in Figure 4. The multiple sequence alignment is shown in fasta_aln format.

```
>Frog2_K80
EEAETK-LRAALATFNDKIRYLEKQREILESRW-SMIQTEE------TTQ-TDLEPQ--Y
LSYISRLLGEVKNVTQNNHQTQRQLLDMMDSVNDMKDKFEDELFRRTDVEYSFVELKKDA
DGRSLEQTELETRKQDIKEMIDLMKTVYKQELKELM-EDS--GDISVLVNME-QSCPLNL
DKVVEEVKAQYERIASWSRDEAQALSKNKLAEGVQRVGRYELELKSSRSEITHLNSKIQR
LRSEIQSIQKQCSKLEQDVSLAQTNSNMAIVDANTKVTEIQDVLLKAKQDMAKQLRDYQE
LLHVKMALDVEIAAYNLLL-EGE
>Frog2_K7
EREQIKTLNNKFASFIDKVRFLEQQNKVLETKW-ELLQNQKS-----AKA-SNVGPL--F
DAYIANLRRQLDGLTGDKGKLEGELRNMQDLVEDFKNKYEDEINKRTSAENEFVVLKKDV
DAAYMNKVELEAKMEALTDEINFLRALYEAELRELQ-EQI--SDTSVVLSMD-NNRALDM
DSIIAEVKAQYEDIANKSRANAEAVYQNKFQELQATAGRHGDDLRTTKTEISELNRAMQR
LQAEIESVKAQRAKLEAQIAEAEERGELALKDARTKLAELEAALQKAKQDMARQLREYQE
LMNVKLALDIEIATYRKLL-EGE
>Frog2_03
EKEQIKTLNNKFASFIDKVRFLEQQNKMLETKW-AILQEQR------TAR-SHIEPL--F
EAYIGNLRRQLDCLLGEKSRLEGERKNMEDIVEDFKRKYEEEVNRRTTAENEFVVLKKDV
DAAFLHKAELQTRVESQTDEVNFLRTMFEAEIAQLQ-GQI--SDTSVIVSMD-NNRNLDL
DGIIAEVKAQYEEIANRSRAEAESWYQSKYEELRLTAGRNGDDLRNTKNEVADLNRMINR
LRGEIETVNAQRGKLEGAITEAEERGEMATKDAKKKLADLEEGLQKAKQDMARQLREYQE
LMNVKLALDIEIATYRKLL-EGE
>Frog2_04
EREQIKTLNNKFASFIDKVRFLEQQNKVLETKW-NLLQEQGVK----SVK-CNIDPL--F
EAYISALRRQLDSLLSEKVRLDSDLRQMQDAVEDYKKKYEDEINKRTSVENEFVVLKKDV
DAAYMNKVELEAKLNALTDEINFLRAVYEAEIAQMQ-TQI--SDTSVVLSMD-NNRTLDL
NGIIAEVKAQYEDIANRSRAEAESWYQTKYEELQQSAGRHGDDLRSTKTEISELNRMINR
LRSEIDSVKKQCAKLQAAITEAEERGELALKDAKKKLADLEEALQKCKQEMARQLREYQE
LMNVKLSLDVEIATYRKLL-EGE
>Frog2_05
EREQIKTLNNKFASFIDKVRFLEQQNKVLETKW-SLLQEQGGQVKG-SRK-NNIDPI--F
DAYINSLKRQLDALQNDKLRLDGELRNMQDLVDDFKNKYEDEINKRTSAENEFVVIKKDV
DAAYMGKVELEAKVDALTDELNFLRTFFEAELGELQ-AQI--SDTSVVLSMD-NNRALDL
ESIIAEVKSQYEDIAKKSRAEAESVYQGKVQELQASAGEQGDVLRNTKSEISELNRKSQR
LKAEIENVKKQIAKLQASITEAEERGDLVLKDAQSKLAELEAALQKVKQDMARQLREYQE
LMNVKLALDVEIATYRKLL-EGE
>Frog2_06
EREKIKLLNNKFASFIDKVRFLEQQNKVLETKW-SLLQKQQVS----KSRSSEIESI--F
NTLITSLRSQLDSLVKDKGLLGGELQVMKDHAENFKNKFELEFKKRTADENDFVSLKKDV
DATYLIQVQLETKQKALDDELAFLRNLYKEELDGIK-QQT--AGTSVVLSMD-NSRMLDL
GDIIADVKAQYEDTTRRSKEEAEAAYQSKYQQLQLAAGQQGEDLKSSKKEISEVNRSVQK
LRNEIESVKKQIASLQVSIQDSERRGDIALRDAQEKLVNLERALQKAKQEMAQQLREYQT
LLNVKLALDVEIATYRKLL-EGE
>Frog2_07
EREQIKTLNNKFASFIDKTRFLEQQNQVLKTKW-ELLQEHGQKGV--TKR-SNIVPL--F
EAYISNLRRSLDSLLNDKHRLESDLRSTQDLVEDYKNKYEDEINKRTTAENEFVVLKKDV
DGAYMNKVELDCKVDALTDEINFLRTLYEAEVQDLQ-GQL--SDISVVLSMD-NNRALDL
DGIIAEVKCQYEEIANKSKAEAEAACAMKFQQLQATAGQHGDSLKSSKTEIADLNRMIQR
LKAEIESVKKQCANLQTSITEAEERGELAVKDAQKKLDELQEALRKAKEEMAKQLHDYQR
LMSIKLALDIEIATYKKLL-EGE
>Frog2_08
ERDQIKGLNNKFAGFIDKVRFLEQQNQILGTKW-NFLQEKSQKLC--SRR-DTIKPL--F
DAYITNLQRQLEGLRSEKCRLDGELKNMQDVVEEFKSKYEEEINKRTCTENEFVALKKDV
DILYMKKAELEAKEESLGNEINFLRNIFDAELAELQ-DCL--SDINVVLSMD-NNRDLDL
NGLIAECKAQYEEIAARSKTEAEAAYSKKFQQLKEAAGQHGDNLRSSKTEIQDLNTMIKR
LQSEIECVKKQIAGLQAAICDAESRGEATLKDANCKMSELEAVLQKAKEDLAIQLREYQE
LLRVKLALDVEIATYRTLL-EGE
>Frog2_09
ERDQIKCLNNKFASFIDKVRFLEQQNQILGTKW-NFLQDKSQKLC--SRR-DTIKPL--F
DAYITNLQRQLEGLRSEKCRLDGELKNMQDVVEEFKCKYEEEINKRTCAENQFVGLKKDV
DVLYMKKAELEAKEETLANEISFLRSLFDAELAELQ-DCL--SDINVVLCMD-NNRELDL
NGLIAECKAQYEEIAARSKTEAEAAYARKFQQLKETAGLHGDNLKSSKTEIQDLNTMIKR
LQSEIECVRKQIAGLQAAICDAESRGEATLKDARCKMSELEAVLQKAKEDLAIQLKEYQE
LLRVKLALDVEIATYRTLL-EGE
>Frog2_10
EREQIKTLNNKFAAYIDKVRFLEQQNKVLETKW-KLLQEQGTKGS--TKR-ANLEPL--F
EKYIGDLNKYLSTLINEKGSLEHELKNLQGFVEDYKKKYEEEINKRTKAENDFVVVKKGV
DTAYMANRELEAKADGVTSEINFLKTLYAAELSQVH-DSV--TNTSVVLTMD-NNRDLNL
EGIIQEVKAQLELSAQRSKMEAEALYDNKYKELQRTAEGHGDSIKNSKSEIAELNRRIQR
LKAEIESTKKQIAGLNQSIAGAEEKGNLALKDAEKKIQDLEAAEKKLKEDMARQLKEYQE
LLAAKISLDVEISTYGLML-GGE
>Frog2_11
EREQIKTLNNKFAAYIDKVRFLEQQNKVLETKW-KLLQEQGTKHS--TKR-ANLEPL--F
EKYIGDLRRYLDTLINERGRLEHELKNQQDLVEEYKKKYEDEINKRTKAENDFVLLKKDV
DAAFMAKTDLEARVDGLTSEINFLRTLYAAELSQVH-EQV--TDTSVILTMD-NNRDLNL
SGIIQEVKCQYEQIAQRSKMEAEALYDNKYKELQKTVEGHGHSIKNSKAEIAELNRNIQR
LRAEIENLKKQIAGLNQSIAGAEEKGNLALKDAEKKLHDLEAAEKKLKEDMARQLKEYQE
LLAAKISLDVEISTYGLML-GGE
>Frog2_12
EREQIKTLNNKFAAYIDKVRFLEQQNKVLETKW-KLLQEQGTKGS--TKR-ASLEPL--F
EKYIGDLRRYLDTLTNEKARLLQELKNLQDLVEEYKKKYEDEINKRTKAENDFVVLKKDV
DAAYMIKTELEAKVDAVTSEINFLRTLFAAELSQVH-DSV--TDTSVVLTMD-NNRDFNL
EGIIQDVKAQLELAAQRSKMEAEALYDNKYKELQRTAEGHGDSIKNSKTEIAELNRRIQR
LRAEIENIKKQIAGLNQSIAGAEEKGNLALKDAEKKLQDLEAAEKKLKEDMARQLKEYQE
LLAAKISLDVEISTYGLML-GGE
>Frog2_13
EREQIKTLNNKFAAYIDKVRFLEQQNKVLETKW-KLLQEQGTKGS--TKR-ANLEPL--F
EKYIGDLNKYLSTLINEKDRLTQELKNLQVLVEDYKKKYEDEITKRTKAENDFVLLKKDV
DTVYMAKTELEAKVDAVTSEINFLRTLYAAELSQVH-DTV--TDTSVVLTMD-NNRDLNL
EGIIKDVKAQLELCAQRSKMEAEALFDNKYKELQKTAEGHGDSIKNSKSEIAELNRRIQR
LKAEIENIKKQIAGLNQSIAGAEEKGNLALKDAEKKLQDLEAAEKKLKEDMARQLKEYQE
LLAAKIALDVEIGTYGLML-GGE
>Frog_K8
EKEQIKTLNNKFASFIDKVRFLEQQNKMLETKW-SLLQNQK------ATR-SNMDAM--F
EAYIGNLRRQLDGLGQDKMRLESELGNMQGLVEDFKNKYEDEINKRTELENEFVLLKKDV
DEAYMNKVQLEARLEALTDEINFLRQLYEEELRELQ-SQI--SDTSVVLSMD-NNRSLDL
DGIIAEVRAQYEDIANKSRLEVESMYQVKYQELQASAGRHGDDLKNTKAEISELTRYTTR
LQSEIDALKAQRSNLEAQIAEAEERGELALKDARTKLAELEAALKNAKQDMARQLREYQE
LMNVKLALDIEIATYRKLL-EGE
>Chicken_K80
EKEDIKLLNNQFVTLIEKVQCLEQQNKILTTRW-NFLKDQDNS----HSE-SDVKAI--Y
DQYMSKMNQEMQALNYEQENLESELTEVLSTMDNFRSKYEDEIRLCSGMEYTFMELKKDL
DASTLHRTELEVKLSGLQELMDLKKTIYEQELEELL-TEI--KDISVVLGID-NTCKLDL
SRIVEEVRAQYEALALRSWEEAEALTRRKLNEGNTQSATYGGHLLDSRREIADLNIQIQK
LRSCIVSQKSQCLYLEENIKEAGEHGEMALKDAKAKLVKLEEALQKSKEDMAHLVKEYQE
```

```
LMNIKLALDLEILTYRKLV-EGE
>Chicken_K7
EKEQIKTLNNRFASFIDKVRFLEQQNKMLETKW-SLLQDQK------TTR-SNIVPM--F
EAYITNLRRQLDGLLNDKGRLEGELRNMQDLVEDFKAKYEDEINKRTTAENEFVVLKKDV
DAAYMNKVELEAKVDALTDEINFLRSLYEAELRELQ-AQI--SDTSVVLSMD-NSRNLDL
DSIIAEVKAQYEDIANRSRAEAESWYQSKFEALQVTAGKHGDDLRNTKNEITEINRVIQR
LQGEIENAKAQRAKMEAAIAEAEERGELAVKDARAKLEELEAALQKAKQDMARQLREYQE
LMNVKLALDIEIATYRKLL-EGE
>Chicken_K84L
EKEQIKTLNNKFASFIEKVRLLEQQNEVLETKW-SFLKGQK------HGR-NTIVPM--L
EACIGNLKKQLEALGCNRARLETQLKAVQEALETNKKIYEDERSRRSSTEKELIALKKDV
ECFFLNKAELGAKVGRMEGEFGFLRVLYEEESQQLR-AHI--SDTAVVVQMD-NSWDPNL
DGIVANVKAHYEDTACRSRAETEAWCKGKFKELQVTAGRNADSLQEKKREAAELARMVKK
LSGEVRSAKEQCCKLETAVADAEQRKEMSIKDAKSKLTELEAALQKAKQDLARQLWEFQE
LMNIKLALDVEITTYKKLL-EGE
>Chicken_K5L3
EKEQIQTLNNKFASFIDKVRFLEQENKVLETKW-ALLQEQGHK----TVR-NNIEPL--F
EAYINNLRRQLSSLLADKENIGGELSKVQSLAEDFKSKYEEEINKRTAAENEFVILKKEV
DSTYMTKTELQARLDALLEEIDFLRALYEAELSQLQ-SQI--ADTSVILTMD-NNRSLDM
DSIIAEVKAQYEDIAKRSREEAESWYQSRYEELQATAGRHGDDLRSTKQEISELNRHVQR
LRAEIDSVKKQCASLQTAIADAEQRGELALKDARDKLAELETALQQAKADLARQLREYQE
LMNVKLALDIEIATYRKLL-EGE
>Chicken_K5L2
EKEQIKTLNNKFASFIDKVRFLEQQNKVLETKW-SLLQEQGMK----TVR-NNLEPL--F
ETYINNLRMQLNSLLSDKGRLEGELVNTQYLVEDFKKKYEDEINRRTIAENEFVTLKKDV
DASYMNKVELQAKADALTEEINFLRALYEAELSQMQ-TQI--SDTSVVLTMD-NNRNLDL
DSIIAEVKAQYEDIANRSRAEAESWYQTKYEELQATAGRHGDDLRSTKQEISELNRHVQR
LRAEIDSVKKQCANLKAAIADAEERGEIALKDAKAKLAELEDALQQAKADLARQLREYQE
LMNVKLALDIEIATYRKLL-EGE
>Chicken_K5L1
EREQIKTLNNKFASFIDKVRFLEQQNKVLETKW-SLLQEQGHT----VTR-KSLEPL--F
ETYINNLRRQLDSLMGERGRLDSELRSMQDMVEDFKNKYEDEINRRTGAENEFVVLKKDV
DGAYMNKVELQAKADGLADEINFLRALYEAELSQMQ-QQV--SDTSVVLSMD-NNRSLDL
NSIIAEVKAQYEDIANRSRAEAEAWYQNKYEELQVSAGRHGDDLRNTKMEISEINRMVQR
LRNEIESVKKQCANLQAAIAEAEERGEMALKDAKSKLAELEDALQKAKADLARQLREYQE
LMNVKLALDIEIATYRKLL-EGE
>Chicken_HBS1
EKEQIKNLNNQFACFIDKVRLLEQQNKVLTTKW-ELLQQYVLP----ASR-RNLEPV--F
ENFICNLRKQLECVLGERERLENEERCLRDLVQEYKCKYEDEINKRTAAENEFVVLKKDV
DCLYLTKEELEVRVGLLRQQLEFLKCIYAEERAQMD-CQL--CDTSVIVQMD-NSRDLDM
EGIIKSVECCYEEIAQKSKAEVEAFYQTRLEELHSSRGKFCDDLRNNQSEIAELNRMIQK
LQCESDNVKKQIAALQTAICDAEQRGDCALKDARQKLVDLQTALQQAKDKMACLLRDYQE
LLNVKLALDIEIATYRTLL-EGE
>Chicken_HBS2
EKEQIKTLNTQFACFIDKVRFLEQQNKVLETKW-GLLQQYVLP----KKG-KNLELY--F
ENYICDLRKRLDCLLCEKQKLGNEECATSQLVEEFKCKYEEEINRRTTVENEFVALKKDA
DCIFLNKEELEVKVDLLRRQLELLKCVFEEERAQVD-RQL--CDTSVIVKMD-NNRDLDM
ESIIKNVECWYQEIAQKSKEEVDAFYQTRFQELQDKRGKYCDDLQSNKCEISELTRMIQK
LQCELENVKKQVSCLQTSICDVEQRGDCALKDAREKHVELQNALQKAKDELACMLRDYQE
LLNVKLALDIEIATYKTLL-EGE
>Chicken_HBS3
EREQMRSLNNQFACLIDKVQHLEQQNRLLATKW-AFLQKHIL-----PSQ-KNFKHV--F
NTFICSLQRRLDSLLHERGQMELELNNTEKLIEDLKCKYEQEVNRCTAAENEFVLLKKDA
DCVYLTKAELEAKLETLKQQIELLKCMSAQGTAEME-KSL--CDTSVVVKMD-NSRSLDM
EGILRGIELWYEDVAQKSKAELDALYRTRYQELEETKRKQCSELKSHQQEIEELSFVMQR
WQCDLENVKKRVSSLRTSVSDTEQRGDRALKDAREKHLELQNALQKAKDELACMLRDYQE
LLNVKLALDIEIATYKTLL-EGE
>Chicken_K78LT
EKEQIKTLNSNFASFIDKVQCLERQNQALMTKW-ELLQQQSSRP---EES-RSITSF--F
QSYINNLQRQLDMLQSQKEQLDPEAYEMLQLVEDYKKRFEDEINKRASKEEEYVELKKEL
DSTYMRKMEFEVRVEILKQELEFLRCLYEAELSQLQ-TVV--DNTNVILSMD-NGREVNM
DGIIEEVRQEYEKIAQKGKDEVNAMYQGRYQDLQNMRVNQREQLRYTYQEIQELTRQIQR
LQPEIEIVRRENDSRQEAIRDAEQRGSSAVKDGQKKLQELENALQQAREDLARILHDYQE
LLNAKLSLDVEIAMYRSLL-EEE
>Chicken_K78L4
EKEQIKTLNNQFASFIDKVRFLEQQNKVLSTKW-ELLQQQGPS----GPR-KNLDVI--F
ENYIQGLRRRLESLLGQRGELESELQNMRQYVEEYKTKYEEEINRRTAAENEFVVLKKDV
DCAYMTKVELEAKVGALTDEISFLRCIYEEELAQMQ-TIS--RDLSVVVSMD-NNRHLDL
DSIIEEVRRQYEQIAQNSRAEAEAWYQSRYEELQTTAGRHGDSLRNTKIEIQELTRNVQR
LRAEIENVKKQNQQLQAAIAEAEERGEMALKDARRKLEELECALSKDKEELARLLKEYQE
LLNIKIALDVEIAMYRKLL-EGE
>Chicken_K78L3
EKEQIKTLNNQFASFIDKVRFLEQQNKVLSTKW-ELLQQQGPS----GPR-KNLDAI--F
ENYIQGLRRRLESLLGQRGELESELQNMRQYVEEYKTKYEEEINRRTAAENEFVVLKKDV
DCAYMTKVELEAKVGALTDEINFLRCIYEEELSQMQ-TIS--RDLSVVVSMD-NNRHLDM
DSIIEEVRRQYEQIAQNSRAEAEAWYQSRYEELQTTAGRHGDSLRNTKIEIQELTRNIQR
LRAEIENVKKQNQQLQAAIAEAEERGEMALKDARVKLEELEAALSKDKEELARLLKEYQE
LLNVKIALDVEIAMYRKLL-EGE
>Chicken_K78L2
EKEQIKTLNNQFASFIDKVRFLEQQNKVLSTKW-ELLQQQGPS----GPR-KNLDVI--F
ENYIQNLRRQLDSILAQRGQLESELQNMQQYVEDYKNKYEEEINRRTTAENEFVVLKKDV
DTAYMTKVELEAKVGALTDQINFLRAIYEEELSQMQ-TIS--RDLSVVVSMD-NNRHLDM
DSIIEEVRRQYEQIAQSSRAEAEAWYQSQYEQLQSTAGMHGDSLRNTKIEIQELTRNIQR
LRAEIENVKKQNQQLQAAIAEAEERGEMALKDARVKLEELEAALSKDKEELARLLKEYQE
LLNIKIALDVEIAMYRKLL-EGE
>Chicken_K78L1
EREELKTLNNKFASFIDKVRFLEQQNKVLETKW-KLLQEQSPT----TST-RSLDPY--F
EAYISRLRKQLDGLSSEKIQLESELKSFQDMVEDFKTKYEEEINKRTAAENDFVLLKKDV
DGVYMNKVELQAKLDSLADELNFLRNLYETELCQMQ-KTV--SDTSVVLSMD-NNRCLDL
DSIIAEVKAQYEEIANRSRLEAEAWYQSKYKELQATAGKHGDSLRDTKAEISELNRVIQR
LRAEIENVKKQCESVQSSIADAEQRGELALKDARDKLTELETALKKAKADLAQQLRDYQE
LMNVKLALDIEIATYRKLL-EGE
>Chicken_K8
EKEQIKTLNNRFASFIDKVRFLEQQNKVLETKW-SLLQSQQ------PQR-SDLRGL--F
EGYVGTLRRQLEGLGQERLRLEAELGSMQGLVEDFKNKYEEEINHRTEKENEFVLLKKDV
DEAYMSKVELESRLESLTDEINFLRQLYDEELSQMQ-TQI--SDTSVVLTMD-NNRSLDL
DSIIAEVKAQYEDIANRSGLEAESWYQTKYEELKTTAGKHGDDLRTTRSEISELNRMIQR
IQAEIEVLKNQRATLETAIAEAEERGEMALKDARAKLAELEAALQKAKQDLARQLREYQE
LMNVKLALDIEIATYRKLL-EGE
>Alligator_K80
EKEEIKVLNNQFVALIKQVQSLELQNKMLETRW-DFLKDQDNS----SSE-VDIKCI--Y
DEYMGRMEQEMKSIDQEKEELESELTKVLDSMDDFRAKYEGEMQLRNSLEYTFLELKKDL
DAGTLHRTELEVKLNGLQEHMELKKSVYEQELQELL-AEV--KDISVVLGID-TRCNLDL
HSIVEEVRAQYEALAVWSWKEAEAHIWSKLNEGVPRSAMYGNHLFNSRREIADLNIQIQK
```

```
LRSCIVSLKSQCLSLEESIKEAGEQGEFALQDASAKLASLEEALQKGKEDVTHLVKEHQQ
LMNTKLALDVEILTYRKLV-EGE
>Alligator_K7
EKEQIKTLNNKFASFIDKVRFLEQQNKMLETKW-SLLQDQK------TAR-SNIAPL--F
EAYISNLRRQLDGLMNDKGRLEGELKNMQDLVEDFKNKYEDEINRRTTAENEFVVLKKDV
DGAYMNKVELEARVDALTDEINFLRSLYEAELQELQ-TQI--SDTSVVLSMD-NSRNLDL
DSIIAEVKAQYEDIANRSRAEAESWYQSKFEVLQATAGKHGDDLRSTKNEIAETNRMIMR
LQSEIDNVKNQRAKLEAAIAEAEERGEMAIKDARAKQDELEAALQKAKQDMARQLREYQD
LMNVKLALDIEIATYRKLL-EGE
>Alligator_K84L1
EKEQIKTLNNKFASFIDKVRFLEQQNKVLETKW-SFLQNQQ------CRR-NTIMPT--L
DAYICNLRKQLEALGCEGAQLEADLKAAHEVMETNKKMYEEECSRRTCAENEFVVLKKEV
DCVFLNKAELEARVESLKDEIIFLKSLYEEEIHMLH-SHI--SDTSVIVQMD-NSRNLDL
DGIITEVKAQYEDIASRSRAEAEAWYESKFEELRVTAGRHADNLRDTKNEIAELTRIIQR
LRGEVGTAKDQRCKLEAAVAEAEQQGELAIKDARCKLTDLETALQQAKADLAHQLREYQE
LMNVKLALDIEIATYRKLL-EGE
>Alligator_K84L2
EKEQIKTLNNKFASFIDKVRFLEQQNKVLETKW-RFLQEQK------RYK-SNIEPM--F
EAYIYNLRRQLEVLAGDRAKLETELSNMQETLEDFKEKYEEETSRRACAENEFVMLKKDV
DCAYMNKAELEAKVESLIEEITFLKNLHEEEIAQLQ-ASI--SDTSVIVQMD-NSRALDM
DDIIAEVKAQYEDIANRSRADAESWYYSKYEELRETAGKHSDKLRNTKNEIMELSRVIQR
LNGELENAKAQRGKLEAAIAEAEERGEMALKDAKCKLTDLEAALQKAKQDMACQLREYQE
LMNVKLALDIEIATYRKLL-EGE
>Alligator_K5L4
EKEQIKTLNNKFASFIDKVRFLEQQNKVLETKW-SLLQEHK------TAR-SNIEPM--F
EAYISNLRRQVEVLGGERIRLDSELKGMQDVVEDFRIRYEDEIHKRTAAENEFVVLKKDV
DAAYMNKVELGAKVDGLTDEINFLRALYEAELAQMQ-SQI--SETSVVLSMD-NNRNLDL
DSIIEEVKAQYEDIANRSRAEAESWYQSKYEELQLTAGRHGDDLRNTKVEITEMSRMIHR
LHSEIDSVKKQCASLQTAIADAEQRGELALKDARVKLAELEDALQKAKQDMARQLREYQE
LMNVKLALDIEIATYRKLL-EGE
>Alligator_K5L3
EKEQIQTLNNKFASFIDKVRFLEQQNKVLETKW-MLLQEQGQK----TVR-NNIEPL--F
ETYINNLRIQLNGLVGDRGRLEGELDNTQNLLEDFKNRYEEETNRRHDAENDFVTLKKDV
DGAFMNKVELQAKVDSLIDEINFLRAIYEAELSQMQ-TQI--TDTSVVLSMD-NNRSLDM
DSIIAEVKAQYEDIANRSRVEAESWYQVKYEELQATAGRHGDDLRNTKHEISEINRHIQR
LRADIESVKRQCASLQAAIAEAEERGEMALKDARDKLAQLEDALQQAKADLARQLREYQE
LMNVKLALDIEIATYRKLL-EGE
>Alligator_K5L2
EREQIKTLNNKFASFIDKVRFLEQQNKVLETKW-TLLQEQGQT----VTR-KTLEPL--F
EAYINNLRRQLDSLLGDGPRLDSELRNMQDLVEDFKNKYEDEINRRTAAENEFVMLKKDV
DNAYMNKVELQAKADSLADEINFLRALYDAELSQVH-QQV--SDTSVILSMD-NNRSLNL
DSIISDVKAQYEDIANRSRAEAESWYQSKYEELQVSAGRHGEDLRSTKMEITEINRMIQR
LRNEIDNVKKQCANLQAAIAEAEERGEMALKDARAKLADLEEALQKAKADLARQLREYQE
LMNVKLALDIEIATYRKLL-EGE
>Alligator_K5L1
EKEQIKTLNNKFASFIDKVRFLEQQNKVLETKW-ALLNDQGQKSS--LSK-NNMDPL--F
EAYISNLKKQLNSLLNERGRLDGELKNMQDLVEDFKNKYEDEINRRTTAENEFVVLKKDV
DAAYMNKVELEAKVDALMDELNFLRALYDAELAQLS-AQM--SDTSVILSMD-NNRDLDL
SSIITEVKAQYEDIANRSRAEAEAWYQTKFEELQATAGKHGDDLRNTKGEISELTRLIQR
IRAEIENARNQCANLQTAIADSEERGELALKDAKLKLAELEDALQKAKADMTRQLREYQE
LMNVKLALDIEIATYRKLL-EGE
>Alligator_HBS1
EKEQIKNLNNKFACFIDKVRLLEQQNKVLTTKW-DLLQQCIVP----GTR-RNLEPL--Y
ESFICNLKKQLEHLLCDRDKLMCEEKAANHLVDEFKCKYEEQINRRTAAENEFVVLKKDV
DCLHLTKEELEIRVALLRQQLEFLTCIFAEERAQMD-CQL--CDTSVIVEMD-NNRGLDM
DSIINSVKCCYEEIAHKSKAEVEAFYQTRLEELHTNRGKYCDDLRISQCEIADLKRGIQK
LQGELDGVKKQIGCLETAICDAEHHGDCTLKDAREKHINLQTALQQAKDKLACLLRDYQE
LLNVKLALDIEIATYRSLL-EGE
>Alligator_HBS2
EKEQMKCLNNQFACFIDKVQCLEQQNKALETKW-NLLQQHTMP----AAR-KTLEAY--F
ENYICKLKKQLECLLGEREQLSNKECVAKKLVDEFRCKYEEVINKRMTAEHGFVLAKKDV
DYAFLNTEEQEVKVDLLKCQLELLNHVFTEERAQMD-CQL--CDTSVMVKMD-NRRDLDM
ETIIKNVECCYKEIAQKSKEEVDTLYQTRFQELQEQRGRFCDDLESNKREIAQLTQLIQE
LQCELDNVKKQVGCLQTDIRDAEQHGDFTLQDGRAKHVELQNALQKAKDELASMLRDYQE
LLNVKLALDIEIAMYKTLL-EGE
>Alligator_HBS3
EREQIKKLNNQFACFIDKVRCLEQQNKVLGTKW-NLLQEQVL-----PCR-KNLQHL--F
ENYLCSLQRQLDCLLSERGQLEPELQNGQKLVEELKCKYEQEINRRTASENEFILLKKDV
DCSYMSKVELEAKVDTLRQEIEFLRCVYAQELVQME-GNL--CDTSVVVKMD-NNRDLDM
EGIIREIECWYEEIAQKSKAEVDALYRTRFQELQETKGRYCNDLKCNHHEITELSRVVQR
LQHNLDNAKKEVACLQTSICETEQRGDCALKDAQGKYGELQNALQKAKDELASMLRDYQE
LLNIKLALDIEIAMYKTLL-EGE
>Alligator_K78LT
EKEQIKSLNDKFACFIDKVRCLEQENQVLLTKW-ELLQQQGGQT---GVH-RDIEPF--F
QSYICHLRRQRDTLQSQKEELNLEDCKMLQIVNEYKASYEKEIDRRLCTEEEFVNLKKNL
DCIYMGKMEVGVKVNILREEIEFLKCLYAMELSDVQ-RIA--QDTNVVVSMD-NCRDLDV
EGIIAEIRREYEGLAQKSQAEVNAVYQSKYQDLHNTWGRHCDRLRTRQHEIQELTRLIQK
LRLDIGDVKKKNACLQEEIADVERRGCCAVSDAQEKLRELRCSLLKAKDNLAHLLRDYQE
LLNVKMALDIEIATYRALL-EGE
>Alligator-K78L6
EREQIKTLNNKFASFIDKVRFLEQQNKVLETKW-DLLQGTT------TTR-VSLEPM--F
EAYINNLQCQLENLLGNRGQLEGELRNLQDLVEDFKKKYEEEINRRTAIENEFVMSKKDA
DHSFMNKVELQVKVDSLIDEISFLRAFFKAELDQMQ-GQI--TDTNVILQMD-NNRDLDL
NSIISEVKAQYEDIANRSRAEAEAWYQSKFQELQTTAMTHGDDLKNTRNEISELTRIIQR
LKAEIDNVKKQIAGLQTAIADAEQRGEMALKDARAKLSDLQNALQTAKDELAHLLRDYQE
LMNVKLALDIEIATYRTLL-EGE
>Alligator_K78L5
EKEQIKTLNNQFATFIDKVRFLEQQNKVLSTKW-ELLQQQGHS----GPR-KDLSSL--F
ESYIQNLKRQLDSILHQREQLESDLQNMQPHVEEYKKKYEEEINRRATAENEFVVLKKDV
DNAYMNKVQLEAKVQALMDEIDFLRHVFEEELSQMH-TIS--RDLSVVVSMD-NTRHLDM
DSIIDEVRRQYEDIARSSRAEAEEWYQSRYEELQNTAGRHGDNLRNTKQEIQELTRCIQR
LRSEIENVKKQVRALQAAIAEAEDRGEMALKDARSKLQELEAALQKDKEELARLLKEYQE
LLNIKIALDIEIAMYRKLL-EGE
>Alligator_K78L4
EKEQIKTLNNQFATFIDKVRFLEQQNKVLSTKW-ELLQQQGHS----GPS-KDLSSL--F
EIYIQNLKRQLDSILDERGQLVSELQKMQGHLEDYKTKYEEEINRRTTAENEFVVLKKDV
DNAYMSKVELEAKVQALTDEINFLRYVYEEEMSQMH-TIS--RDLSVVVSMD-NTRHLDM
DSIIDEVRRQYEDIARSSRAEAEEWYQSRYEELQNTAGRHGDNLRNTKQEIQELTRCIQR
LRSEIENVKKQHQQLQAAIAEAENRGEMALKDARSKLQELEAALQKDKEELARLLKEYQE
LLNIKIALDIEIAMYRKLL-EGE
>Alligator_K78L3
EKEQIKTLNNKFATFIDKVRFLEQQNKVLSTKW-ELLQQQGHS----GPR-KDLSSL--F
ENYIQNLRRQLDSIHGQRGQLESELQNMQNYVEDYKNKYEDEINRRTAAENEFVVLKKDV
DCAYMTKVDLEAKVQALIDEINFLRYVYEEELSQMQ-TIS--RDLSVVVSMD-NSRHLDM
```

```
DSMIDEVRRQYEDIARSSRAEAEAWYQSRYEELQTTAGRHGDNLRNTKQEIQELTRCIQR
LRSEIENVKKQXXX----------------------------------------------
-----------------------
>Alligator_K78L2
EKEQIKTLNNKFATFIDKVRFLEQQNKVLSTKW-ELLQQQGPS----GPR-RDLTAL--Y
ENYIENLRRQLDNVLSQRGPLESQLHDMQGYVEDYKNKYEEEINRRTSAENEFVVLKKDV
DCAYMTKVDLEAKVQALIDEINFLRYVFEEELSQLQ-TIS--RDLSVVVSMD-NSRHLNL
DSIIDEIRAQYEQIAQSSRAEAEAWYQSRYEELQSTAGQHGESLRNTRQEIQELTRCIQK
LRSEIENVRKQCEQLQAAIAEAEERGEMALKDARCKLEELECALHKDKEELARLLKEYQE
LLNVKIALDIEIAMYRKLL-EGE
>Alligator_K78L1
EREQMKTLNDKFASFIDKVRFLEQQNKVLETKW-KLLQEQGQGT---GPHSRSLDQL--Y
EVYINSLRKQLDGLTSEKSQLQLELKNCEDLVEDFKTKYDEEINKRTAAENDFVLLKKDV
DAAYMNKVELQAKLDSLADELNFLRCVYDAELSQMQ-QTV--SDTSVVLSMD-NNRNLDL
DSIIAEVKAQYEEIAQKSRAEAEAWYQCKYQELQVTAGKHGDSLKDTKAEISEMNRMIQR
LRAEIENVKKQCENLQTSVADAEQRGELALKDARVKLSELEAALQKAKTELTRQLRDYQE
LMNVKLALDIEIATYRKLL-EGE
>Alligator_K8L
EKEELKTLNNQFVHLIERVCSLEQHKKVLETKC-SILKSQK------ATP-ARMENL--L
KTYISQLKKHLADQGQMKGKLHLELHEIQDIMDENKQKYEEEFNLRNEKENDFLLMKKEM
DEAYISQNELEGKLDRLRDELEFLKNFYDQEMHELQ-TEVHMYKESVVISMP-SQPSLEV
DSIVSEAQSQYEKMAERSCKEAEKAYRDRHNKLKDSMGRHGEELQTMKTEINDLTRKSQH
IQLEIQNLKEQCNKLKEDIANADDRYKFPIEEAKNKLHMLENAIQKARQDMAHHLREYQE
LLNTKLGLDIEIATYRKLL-EGE
>Alligator_K8
EKEQIKSLNNKFASFIDKVRFLEQQNQVLETKW-SLLQGQR------TTR-SNMTGM--F
EAYITNLRRQLEGLDQDKLRLESELGNVQGLVEEFRSKYEDEINHRTEKENEFVLLKKDV
DEAYMNKVELESRLEGLTDEISFLRQLYEEELHQLQ-SQV--SNTSVVLSMD-NNRSLDM
DSIIADVRNQYEDIANQSRAEAEGMYKIKYEELRNTASKHGDDLRNTKAEISELTRTIQR
MQAEIDAVKGQRANLEAAIAEAEERGEMAVRDARGKLAELEAALQKAKQDMARQLREYQE
LMNVKLALDIEIATYRKLL-EGE
>Turtle_K80
EKDEMKILNDQFASLIGKVQCLEQQNKVLTIRW-SFLKDQDNS----RSE-LDIKLL--Y
DQYMSKLKQEMRAVGHEREQLESELTEVLKSMDNIRSRYEDEIHKRSGLEFTFVELKRDL
DAGSMHRTELEVKLQGLQELMELKKAVYEQELQELL-AEI--KDISIVMGID-NRCNLDL
SRIVEEVRAQYEALAFRSWEEAEALTWSKLNEGATHSATYRDQLFSGRREIAELTIHIQK
LRTCIVSLKSQCLRLEEDIKEAGGNGQLALRNANTKLAELEQALHKGKADLVGLVKEYHE
LMNIKLALDVEILTYRKLV-EGE
>Turtle_K7
EKEQIKTLNNKFASFIDKVRFLEQQNKVLETKW-SLLQDQK------TTR-SNIAPM--F
EAYINNLRKQLDGWLNDKGRLEGELKNMQDLVEDFRNKYEDEINKRTAAENEFVILKKDV
DASYMNKVELEAKVDALTDEINFLRSLYEAELRELQ-AQV--SDTSVVLTMD-NNRNLDM
DSIIAEVKAQYEDMAKKSRMEAESWYQTKFETLQATAGKHGDELRNTKTEITEITRMIHR
LQAEIDNVKSQRAKLEAAIAEAEERGERAIKDAMAKQEELEAALNKAKQDMARQLREYQE
LMNVKLALDIEIATYRKLL-EGE
>Turtle_K84L
EKEQIKCLNNKFASFIDKVRFLEQQNKVLETKW-SFLREQK------HYQ-NNMEPM--F
EAYISNLKRQLENLGCDRAKLETELHNMQDILEDYKRKYEEECNHRTCAENEFVVLKKDV
DCAYMNKAELEVKVESLIDEITFLRRIYEEEIACLQ-ASI--LDTSVIVQMD-NNRGLNM
DDIIADVKAQYEDIANRSRAEAESWYQCKYEELKVTASKHCDNLHSTRNEITELNRVIQR
LHGELENVKAQRCKLEGAIAEAEERGEMALKDAKCKLAELEAALTKAKQDLACQLREYQE
LMNVKLALDIEIATYRKLL-EGE
>Turtle_K5L4
EKEQIKTLNNKFASFIDKVRFLEQQNKVLETKW-CLLQEQK------TAK-SNIEPM--F
EAYINNLRRQLEALGGERLRLDGELKNMQDTVEDFRIRYEDEINKRTAAENEFVVLKKDV
DIAYMNKVELEAKVDALTDEVNFLRALYEAELAHLQ-SQI--SDTSVVLSMD-NSRNLDL
DGIIAEVKAQYEDIANRSRAEAESWYQTKYEELQLTAGRHGDDLRNTKIEISEMNRMIQR
LRSEIDNVKKQCASLQTAIADAEQRGELALKDAQAKLAELEEALQKAKADMARQLREYQE
LMNVKLALDIEIATYRKLL-EGE
>Turtle_K5L3
EKEQIKTLNNQFASFIDKVRFLEQQNRVLETKW-SLLQEQGQK----TVK-NNIEPF--F
ENYIGNLRRQLNNLMNERGRLEGEVNNMQNVVEDFKNRYEDEINKRTTAENDFVTLKKDV
DSAYMNKVELVAKVDSLTDEINFLRALYEEELAQMQ-TQV--SNTSVVLSMD-NNRDLDL
DGIIAEVKAQYEEIANRSRAEAESWYQTKYEELQVTAGRHGDDLRNTKHEISELTRIVQR
LRNEIDNVKRQCANLQAAIAEAEERGELALKDAQGKLAELEDALQKAKGDLARQLREYQE
LMNVKLALDIEIATYRKLL-EGE
>Turtle_K5L2
EREQIKTLNNKFASFIDKVRFLEQQNKVLETKW-TLLQEQGQT----TVK-RSLEPL--F
DAYINNLRRQLDNLLGERGRLDSELRNMQDMVEDFKNKYEDEINRRTAAENDFVVLKKDV
DGSYMNKVELQAKADALADEINFLRALYEAELSQMQ-QQV--SDTSVVLSMD-NNRSLDL
DSIIREVKAQYEEIANRSRAEAESWYQTKFEELQISVGRHGDDLRNTKVEISEINRMIHR
LRNEIDNVKKQCASLQAAIAEAEERGEMALKDARAKLAELEDALQKAKADLARQLREYQE
LMNVKLALDIEIATYRKLL-EGE
>Turtle_K5L1
EKEQIKSLNNKFASFIDK-RFLEQQNKVLETKW-TLLQDQGQKSN--SNK-NNLEPL--F
ESYINNLRKQLNHLLNERGRMEGELKNTQDLVEDFKNKYEDEINRRTAAENEFVVLKKDV
DAAYMNKIDLEAKVDALMDEINFLRTLYDAELAQLN-VQV--SDTSVILSMD-NNRDLDL
SSIVNEVKAQYEDIANRSRAEAESWYQNKFEELRATAGKHGDDLRNTKGEISELNRLTQR
LRAEIENARNQCASLQTAIADSEDRGELALKDARMKVADLEDALQKAKQDMTRQLREYQE
LMNVKLALDIEIATYRKLL-EGE
>Turtle_HBS1
EKEQIKCLNDQFACFIDKVRCLEQQNKVLATKW-DLLQQHAV-----PAK-RDLLPV--F
ENYICQLKKQLECLLHDRARLEDEEKAVQDLVHEYKSKYEGEINKRTHAENEFVVLKKDV
DCVFMTKEELEAKLLQLRQHFEFLKCIFIEERAQLD-GQL--CDTAVVLEMD-NSRDLDL
SGIIHSVKCCYEEIAQKSKGEAETFYQTRLEELSTNRGRFCDDLKNIQGEIAELKRLIHK
LQGETDKVKKEIACLQTAIADAEQRGDCALKDAREKHIDLQNALQKAKDKLACMLRDYHE
LLNIKLALDIEIATYRTML-EGE
>Turtle_HBS2
EREQMKTLNNQFACFIDKVRHLEQQNKVLETKW-NLLQQCVPP----APQ-KNLEQC--F
ENYICSLRKQLEDLLSDKEQLTCEESASRKLVDEFKCKYEEEISRRTAAETEFVVLKKEA
DGTLLHKEELEVNTTLSKQKLEFLRCVFDEERAQMD-GQF--CDTAVIVKMD-NSRDLDT
DCIIKNVEGWYKEIAQRSKEEVDVLYQTRFEELQDQRGRFCEDLERNKHEIAELTRLIQK
LQGETDHVKKQIACLQTAICDAEQRGDCALKDARGKHMELQNALQRAKDELACMLRDYQA
LLNVKLALDIEITTYKKLL-EGE
>Turtle_K78L4
EREQIKTLNNKFASFIDKVRFLEQQNKVLETKW-DLLQQQGTT----ITK-IPIDPL--F
EAYINNLRRRLDSLVTDRGRLDGELRNMQDLVEDFRKKFEEEIHRRTSAENEFVVLKKEV
DAAYMKKMELQAKVDSLIDEINLLRAVYEAELAQMQ-GQI--KDTNVILQMD-NNRDLDL
NSIIAEVKAQYEDIANRSRAEAEAWYQSRYQELQVTATTHGEDLKVTRNEISEVSRVIQR
LKAEIDSVKKQIAGLQTAIADAEQRGELALKDARGKLADLQNALQTTKDELARLLRDYQE
LMNVKLSLDIEIATYRKLL-EGE
>Turtle_K78L3
EREQIKTLNNKFASFIDKVRFLEQQNKVLETKW-NLLQQQPTS----NAG-NNLEPA--F
EAYVGSLRKHLDSLVGERGRQDSELKNMQDLVEDLKHKYEEEINRRMAAENEFVLLKKDL
```

```
DAAYMKKLEMEAKVAAMTDEINFLRALYETELAQMQ-RQI--SDTSVILSMD-NNRALDL
DGIIAEVKAHYEDMANRSRAEANSMYQGKFQELQVTAGKHDNDLKNSKLEISELTRLIHR
LQAELENVKKQCAKLQSAVAETEGRGELTLKDAREKLVELEMALQKAKEETARLLRDYQE
LLNIKLALDIEIATYKTLL-EGE
>Turtle_K78L2
EKEQIKVLNNQFAVFIDKVRFLEQQNKVLVTKW-ELLQQQGGT----IIK-KDLAPL--F
ENFIQSLRRRLDGLQNQKGQLQSELENMQQYVEEYKHKYEDEINKRTAAENEFVVLKKDV
DNAYMVKVELETRVQGLTDEINFLRFIFEEELSQLQ-TIS--RDLSVVVSMD-NNRHLDL
DGIIQEVRNQYEIIAQRSRAEAEAWYQSRYEELQSTAGRHGDNLRNTKTEIQELTRSIQR
MRAEIESVKKQIANLQSAIAEAEEKGELALKDARAKLDDLEHALQKDKADLANLLKEYQD
LLNVKLALDIEIAMYRKLL-EGE
>Turtle_K78L1
EREQMKTLNNKFVSFIDKVRFLEQQNKVLETKW-KLLQEQGQG----PIK-KSLDQL--F
EAYSNGLRKQLDGLLNERRQLDTERKNFQDLVEDFKHKYEEEINKRTVVENDFVVLKRDL
DVAYMSKVELQVKLDSLTDEINFLRCLYEAELSQMQ-QTA--SDTSVVLSMD-NNRNLDL
DSIIREVKAQYEEIAQRSRAEAESCYQCKFEELQVTAGKHGDSLRDSKSEISELNRMIQR
LQAEIENVKKQCERLQTSIADAEQQGELVLKDAKGKLAELEGALQKAKADLARQLREYQE
LMNVKLALDIEIATYRKLL-EGE
>Turtle_K8L
EKEQIKTLNNKFAHFINKVRTLEQENQVLKTKW-NILQGQK------TGP-SNMEKM--L
KAYINSLQKQLEDLGQQRVKLESDQGNMQDLVEEFKRKYEDEINNRSERENQFVLTKKDV
DEAYIKKQELEVKLQSLTDEINFLMELYAKELRELQ-ADVEMSNNPVVISVD-SSPKLDV
DSIIAEARVQYEKMAENSREEAENKYRITYEQIQEKAGQHGEELRAAKAEINELNWMIQR
IRAEIAALKEQNAKLEANIVQAEEQGKLAVADATGKLQKMEHALQKAKEDMVVQLREYQA
LMNLKLALDIEIATYRKLL-EGE
>Turtle_K8
EKEQIKTLNNKFASFIDKVRFLEQQNKMLETKW-DLLQGQK------TTR-SNMNNL--F
EAYINNLRRQLDGLGQEKLKLEAELGNMQGLVEDFKNKYEDEINHRTEKENEFVLLKKDV
DEAYMNKVELESRLESLTDEINFLRQLYDEELRELQ-SQV--SDTSVVLTMD-NNRKLDL
DGIIAEVRAQYEEMANRSRAEAENMYKTKYEEIQAVAGKHGDDLRSTKTEINELNRMIHR
LQSEIDALKGQRASLEAAITEAEERGEMAIKDASAKLAELEGALQRAKQDMARQLREYQE
LMNVKLALDIEIATYRKLL-EGE
>Lizard_K80
EKEEIKTLNNQFASLIGKVQNLEQHNQVLLTRW-NFLREQDNS----LSD-LDIKLL--Y
DQYMNRLRLEIRSIDNEKEQLDAELDEVLDAMDNVRNSYEEEINKRTGMEFTFTALKKDL
DNGFLHKTELEAKLSGLHAWVELMKNIQEQELEEVM-SQV--KDVSVVLGIDNNRYNPDP
HRIVEDVRAQYEALAIRSWEELEALTRSKLNEREVLSVKYGDHLLHDRRAIAELNIQIQK
MRSCILSLKSQCLRLEDNIKDVGLQGETALNDAKAKLAKLEEALHNAKQDLAQLVKQYQE
LMNIKLALDIEILTYRKLM-EGE
>Lizard_K7
EKEQIKTLNNKFASFIDKVRFLEQQNKMLETKY-SLLQEQK------TTK-SNLAPI--F
ETYISNLRRQLDSLMNDKGRLEGELRNMQDLVEDFKSKYEDEINKRTTAENDFVVLKKDV
DASYMNKVELESKVDGLTDEVNFLREFYEAELRELH-NQI--SDTSVVLQMD-NNRNLDL
DSIIAEVKAQYEEIANKSRAEAEAFYQTKYEALQVTAGKHGDDLRNTKTEISDLNRTIQR
MQAEIENIKNQRGKLEAAITEAEERGELAVKDARAKLQELENALNKAKQEMARQLREYQE
LMNVKMSLDIEIATYRKLL-EGE
>Lizard_K84L1
EKNELQCLNSKFASFIDKVQFLEQQNLMLKTKW-DFLQERK------CCK-SNMEPM--F
KEYIANLKKEQECMECERAQLQAEMKNWREALEVNKKKFEEECHRRTCTENEYVSIKKEV
DCVFMDKSEKEAKVQALMKDLFFYKPTFYQEIHELQ-SCI--SDTCVTVQMD-NSRGLNM
DCVMEVYRRQFEEISSRSRAEAEAWCRCQYQELKTTAAKHCDNLRHVKEELSEITRVVHR
LESEVSNVKAQCTKLEEEVAAAEERGGIAVKDARCKLVELEDALHKAKQDMACQLREYQD
LMNMKMGLDIEITTYKKLL-DGE
>Lizard_K84L2
EKNQLQCLNSKFACFIDKVRFLEQHNLMLKTKW-DFLKEKK------CQK-SNMDPM--F
HEYISRLRKELERLQWERNQLQVEVNNWRDTMEGNKKKYEEEYNRRASAENEYVTLKKEV
DYIFMEKSEKEAKVETLMQDIFFYKTTFEEEICEMQ-SSI--SDTCVTVKMD-NSRDLDV
ACIIEEFRCRYEDIASRSRAEAEAWCQCQYQELKTTAAKHSDNLRNFKDELEELTRTVNR
LQAEIANVKAQRCKLEEEVAGAEERGEMAVKDAKCKLFDLEDALHKAKQDMACQLREYQE
LMHLKLALDIEIATYRKLL-EGE
>Lizard_K84L3
TKNELQSLNSKLASFINKVQLLEQHNLMLKTKW-DFVQEMK------QHR-SDMEPL--F
DDHTSRLKKELECLEREKKEMQIEHDSSAQTLEKNKSRYEEELNKRATAENGFVLLKKDL
DFAFADKAELEAKVEKLAKHISFLKHIYAQEISELQ-NCI--SETCVMVQLD-NRRALDM
NRTIEEFRRQHEYIASRTRAEAEAWLQHQYQELKTTAAKNNDNLNAVKEEIQALTRTAHQ
LESQITSIKTQRCRLEDEVVEAKEHGETAVKDARCKLSDLEEALRKAKQDMTCQLREYQS
LMNVKMAMNIEIATYRKLI-EGE
>Lizard_K84L4
EKEQLKTLNNKFASFIDKVRFLEQQNKVLETKW-SLLQEQK------RVK-SNIEPL--F
DGLIHKLKKQLESLGCDRNKLESDLTSSRDILEDYRKRYEDECNRRTSAENEFVTLKKDV
DCIYMHKADLEGKVESLVEEVNFLRCLHDMELQELH-SCI--SDTSVIVQMD-NSRELDL
DGIIAEVKAQYEDIANKSRYEAESWYQCKYEEMRATAGKHNDHLRDTKNEIMELNRVIQR
LKAEIDTAKGQRSKLEAAVAEAEERGEMALKDAKCKLTELEDALIKAKADMARQLREYQE
LMNVKLALDIEIATYRKML-EGE
>Lizard_K5L4
EKEQIKTLNNKFASFIDKVRFLEQQNKVLETKW-SLLQDQK------ITR-HNFDAM--F
EAYITNLRRQLEGILGERPRLESELKNMQDTVEDFRIRYEDEINKRTAAENEFVVLKKDV
DAAFMNKVELEARVDSINDEATFLKSFYDAELAQMH-AQV--SDTSVILQMD-NSRNLDL
DSIIAEVKAQYEDIANRSRAEAESWYQSKYEELQLSAGRHGDDLHNTKIEISELNRMIQR
LHSEIDNVKKQCAGLQASIADAEQRGELALKDARNKLAGLEEALTKAKADMARQLREYQE
LMNVKLALDIEIATYRKLL-EGE
>Lizard_K5L3
EREQIKTLNNKFASFIDKVRFLEQQNKVLETKL-TLLQEQGQK----TCK-SNIEPL--F
EMYINDLRKKLNNLENDRGRLLGELNNIQNLVEDCKNKYEDEINKRATAENDFVTLKKDV
DAAYLNKVELESKADSLNCEVNFLRALYEEELAQMH-TQI--SDTSVVLSMD-NNRNLDM
DSIIAEVKAQYEDIANKSRAESETFYQCKYEELQTAAGRHGDDLRNTKQEISEINRHIHR
LRAEIDNVKKQCANLQAAIAEAEERGELALKDARQKLAELEDALQKAKQDMARQLKEYQD
LMNVKLALDIEIATYRKLL-EGE
>Lizard_K5L2
EREQIKTLNNKFASFIDKVRFLEQQNKVLETKW-DLLQQQGTT----CQQ-STMEPL--F
DVYITNLRRQLEALTNERGRLDSELRNMQDMVEDYKNKYEDEINKRTGSENEFVVLKKDV
DGAYMNKIELAGKFESLNDEIEFLKAMYDAELSQMQ-QHV--SDTSVVLSMD-NNRCLDL
NSIIAEVKAQYEDIAQRSRTEAESWYQTKYEELQVSAGRHGDDLRNTKTEISELNRMIQR
LRSEIDSFKKQCANLQAAIAEAEDRGEMALKDAKHKLSELEEALQRTKQDLARQLKEYQE
LMNVKLALDIEIATYKKLL-EGE
>Lizard_K5L1
EKEQIKTLNNKFASFIDKVRFLEQQNKVLETKW-TLLQDQGFKTG--ANK-GNLDPL--F
EAYINNLRKQLNGLINEKGRLDGELRNMQDIVEDFKTKYEEEINRRTSAENEFVMLKKDV
DAAYMNKVELEAKVDSLTDELNFLRALYEAELAQLQ-AQM--SDTAVILSMD-NNRDLDL
NGIVSEVKAQYEDIANRSRAEAEAWYQTKFEELQATAGRHGDDLRNTKGEISELNRLIQR
IRAEIENARNQCANMQTAIADAEDRGELALKDARAKLIELEEALQKAKEEMTRLLREYQE
LMNVKLALDIEIATYRKLL-EGE
>Lizard_HBS1
EKEQIKDLNNQFACFIDKVRYLEERNQALATKW-ELLKSQNP-----AVK-KDLSPL--C
```

```
ENYITSMRRKLDNLLCEKNQLQQEHKTMLDLIEANRCKYEEEVKRRTNAENEFIVLKQDV
DGIFKKQKELEQKKEVLEENIGFLTNFFSKERAILE-SQL--NNTSVVVNMD-NSRCLDM
DGLIQEIESWYQGIVQKSKGEANLFYWNQMEDLQSKRSQFYEKLRQNNADIAELNRYVQI
MRCQVGTEKQKVASLHAALRETKELGDHALQDAESKYKDLQNRVQTSKDRLVALVRDYQD
LMNTKLALDIEIATYKTLL-EGE
>Lizard_HBS2
EKEEMKTLNNQFACFIDKVRSLEQQNKVLGTKW-ELLNQCVQ-----PAK-KNLEGY--Y
ENFIATLKKQLECLLSERGKLEHEQKNMQELVEEYRSKYEEEVNRRTTAENEFVVLKKDV
DCVFLTKEELESKVDVLAQELEVLRCVFAEELTQLD-SQI--LDTSVILKMD-NTRDLHM
DLILQNVENWYQNIAHNSKQEAEAFYHNKIAEIQNNRSKFNEELKCNQHEIAELNRVTQR
LRTDLGNAKKQVSNLQSAICDAEQRGDIALKDARNKHAELQNAYQQSKDKLASLLRDYQD
LLNTKMALDIEIATYKMML-EGE
>Lizard_HBS3
EREEMKNLNNQFACFIEKVRILEQENKALETKW-NLLQQYAM-----VPR-RNLEPY--Y
ENFISNMKYQIDCLLHTREHLANDKNAIQELVEDFKSKYEEEFKRRTTAENEFVLLKKDV
DSISLSKTELEGKMNLLRWELEFRKSVYMEELMQLS-GQI--SDMNILVQMD-NNRDLDM
DSIIKNVEAWYQSIAHRSKEEVNALYADRFQELQEQRGKYLNDLKINQYEIEELTRAVHT
LQSDHSNVKKQVDALQASICDVDQRGDQALKDAQEKHSDLLSALQKAKDDLAGMLKDYHD
LLNVKLALDIEIATYRTLL-EGE
>Lizard_K78LT
EREQIKTLNDQFACFIDKVRHLEQQNKLLETKW-SCLQQQEP-----MEK-QNTEHL--Y
QNYIVTLKKQLDLLLDEREQLKLEQVKFQDVVEEYKSRYEGVINRRLGAENEFVVLKKDV
DCAYTSKVELEVKVDSLRQEQDFHRCVYDTELESLA-TAS---DTNVVVSMD-NSRDLDM
EGIIESVRCQYEEIAQRSKDEVNALYETKYKELQTTWGHHCNSLSSSRHEIQELTRLIQR
YKADVENGKKQVEALQVAVADHEQQGDCTLKDAKEKLAEVEKALQSTKDELARLLRDYQE
LLNVKVALDIEIAMYKSLL-EGE
>Lizard_K78L5
EREQIKTLNNKFATFIDKVRFLEQQNKVLETKW-DLLYQQGTP----ALR-DNLEPL--Y
EAYIDTLRRQFESLTNDKNHLDIELKNTQELVEDFKSKYEDEINKRTACENDFVVLKKDV
DGSYMHKIDLGSKADSLTQHIQFLRALFETELSQVQ-SQV--RDTNVVLQMD-NNRDLDL
NGIIAEVRAQYEDIANRSRAEAETWYQSKYQELQTTAQKHCDELKTTKDDIAEMSRVVHK
LRAEIEIVKKAVNGLQSSIAESEQRGEIALKDAQKKLLDLQSALTSSKDELARLLRDYQE
LMNVKLALDVEIATYRTLL-EGE
>Lizard_K78L4
EREQIKTLNNKFASFIDKVRFLEQQNKVLETKL-NLLQQQGST----ATT-IHIDPM--F
EAYINNLRNQLDGLVGDRGRLDGELRNTQDLVEDYRKRYEDEINKRTTAENEFVDIKKNV
DGGFMHKIELGAKADGLTDEINFLRALHEAELSQIQ-GRI--SDTNIVLQMD-NNRDIDL
NSIISEVKAQYEDIANRSRAEAEALYQSKFQELQSTAVNHGDDLKNTKNEIAELSRIIQR
MKAEIDNVKKQIAGLQTAIADAEQRGELALKDARVKLTDLQHALQSAKDELARLLRDYQE
LMNVKLALDVEIATYRTLL-EGE
>Lizard_K78L3
EREQIKCLNDKFASFIDKVRFLEQQNKILETKL-NLLQQQPMP----HLK-RKADHT--F
EAYIHGLRKQLESLSTERGRLGSEWKNMQDLVEDFKRRYEEEINKRTTAENEFVLLKKDV
DLAYMNKMELKTRTASLDKEVDFLRSLYEEEQAQMQ-SQV--TDTSIILSMD-NSRDLDM
DVIIAEVKAQYEDMANRSRAEVDAMYQSKFQELQLTAGKHDDALKNSKTEILELNRLIHR
LRAETGTVKKQCAFLQSSIAEAEERGETALKDAREKLTKLENATQKSNEELVRLLRDYQE
LLNVKISLDIEIATYKTLL-EGE
>Lizard_K78L2
EKEQIKLLNNQFANFIDKVRFLEQQNKVLQTKW-QILQQQTQGR---GPM-QNLDGE--Y
QLFLNGLRNQIDQIRSQKQQLMSELQNMQQVVEDSKNKYEQEINQRTTSENEFVVLKKDV
DSAYMTKVDLDAKVGGIVEQINFFRRIFDIELSQIP-IGG--KDTSVVVNMD-DSRDLNL
EGMIDEVRAQYEEVARSSRAEAETWYRSQYEALQNAAGRHGENLQNTRQEIQELTRNIQR
LRTEIEHAKKQAAQMQSAIAEAEERGEMALRDAKQKLDELEGSLTKDKEVLARLLKEYQD
LLNVKLALDMEIAMYRKLL-EGE
>Lizard_K78L1
EKEQMKTLNNKFASFIDKVRFLEQQNKVLETKY-KLLQEQGPG----AIQ-KHLDPM--Y
EAYINNLRRQLECLQNEKRGLDSELKNFQDMVEDYKTKYEEEINKRTQAENDFVILKKDV
DAAYMNKVELQAKCDALIDEINFMKCLYEAELSQMQ-QTV--SDTSVILQMD-NNRCLDM
DSIIAEVKARYEEIAQMGRIDAESFYQSKYEELQASVGKHGDNLQKTKAEISEHNRMIQR
IRAEIDNVKKQCEKLKSSIAEAEERGELALKDARNKLTELEEALQKAKEEMTRLLKEYQE
LMSCKLALDVEIATYKKLL-EGE
>Lizard_K8
EKEEIKSLNNKFAGFIDKVRFLEQQNKMLDTKW-SLLQNQK------ITR-SNMDSM--F
EAYINNLRRQLDGLGQERVRLDSELGNMQGLVEEFKSKYEDEINHRTEKENDFVLLKKDV
DEAYMNKIELESRLESLTDELNFLRQLHEEELRVLQ-SQI--SDTSVVLSMD-NNRSLDL
DGIIAEVKAQYEEVAARSRAEAESTYKSKYEELQSVAGKHGDDLRITKNEISELNRLITR
IQSEIDGLKGQRANLESAIAEAEERGELAVKDATAKLSELEAALQRAKQDMARQLREYQE
LMNVKLALDIEIATYRKLL-EGE
>SnakeP_K80
EKEEIKTLNNQFAALIGKVQSLEQHNQVLLTRW-NFLKEQDSS----LSD-LDIKLL--Y
DRYMNKLNLEIRSIDVEKEQLDSELDEVLASMDVFRSKFEEEINKRSGLEFTFTTLKKDL
DNGFLHKTELETKLSGLHAWVELMTTIQEQELEEVM-SQI--KDVSVVLGIDNNRYMPDP
HRIVEDVRAQYEDLAIRSWEELEALTRSKLNEREVLSAKYGDHLLNDRRVIAELNIQIQK
MRSCIVSLKSQCLQLEDNIKNVGLQGETALNDAKAKLAKLEDALHSARQDLAQLVKEYQE
LMNIKLALDIEILTYRKLV-EGE
>Snake_K7
EKEQIMVLNNKFASFIDKVRFLEQQNKMLETKW-SILQQQKT-----STR-NNTAPV--F
DAYINNLRRQLDSLQNDKGRLEGELKNMQDLVEDFKNKYEDEINKRTTAENDFVLIKKDV
DASYMNKVELEARVDGLTDEINFLRALYDAELAELQ-NQV--SDTSVILQMD-NNRNLDL
DSIIAEVKAQYEEIANKSRIEAESWYQSKFEVLQATAGKHGDDLRSTRNEIAEINRLILR
LQADIDNVKNQRAKLESAIAEAEERGTLAVKDAKGKLEELEVAMNKAKQEMAHQLREYHE
LMNVKLGLDIEIATYRKLL-EGE
>Snake_K5L2
EGEQIKILNNKFASFIDKVRFLEQQNKVLETKW-TLLQKHDQK----TVM-SNVEPL--F
EAYINNLRTQVNDLEKEKVKLDGELHTTQNLVEDYKSKYEDEINKRTDAENDFVILKKEV
DAAYMNKADLETRLQSLVDETDFLRLLHETELADLQ-RQI--SDTSVVLSMD-NNRYLDL
DSIIAEVKAQYEDIAKKSRAEAEAWYQSKFEELQGTAGRHSDDVRNAKHEISELNRHVQR
IKAEIESVKKQCANLQAKIAEAEDRGEQALKDARQKLAELEDGLQKAKQNMAKLLKDYQE
LLNVKMALDVEIATYRKLL-EGE
>Snake_K5L1
EREQIKTLNNKFASFIDKVRFLEQQNKVLETKW-ELLQRQGTQ----VQQ-RNLEPL--F
ENYIANLRKQFDYLVNERGRLDMELRNMQDMVEDFKNKYEDEINKRTGAENEFVVLKKDV
DGAYMNKIELASKSDSLNDEIEFLKALFEAELSQMQ-QQV--SDTSVVLSMD-NNRSLDL
NSIIAEVKAQYEDIANRSRTEAESWYQSKYEELQVSAGRHGDDLRNTKTEISELNRVIQR
LRSEIDAYKKQCANIQAAIAEAEDRGELALKDAKQKLNELEECLQKTKQELARQLKEYQD
LMNIKLALDIEIATYRKML-EGE
>Snake_HBS1
EMEQMKSLNSKFAGFIDKVQRLEQKNQLLETKW-AFLQQQTK-----PPT-ESLECM--F
EQYIASLKKELEYLQYEKENLQNDRESAKAQTADCKCKYEEKVKQHVAAEKEIIELRKDV
DYIFSNNAELERKVSLLDREIKFLKCVYKKEKEQLD-KQD--QDVDVVVKMD-NSRNLDM
DNIIAMVRKQYEEIMQRSKAEADKFYQNKYEEAQSKSSSYKWDLKNREQQISELIQQVEK
VQGEIQSLQKENEELKKTICEAAKGGEQASKEGQQKYAELVKALQNGKDELASLVRDYQK
VLNVKIVLDIEIAAYKCLL-EGE
>Snake_HBS2
```

```
EKDQMKDLNTQFASFIEKVRSLEQRNRILITKW-ELLQNQKMP----KVK-KDLSTL--F
DNYIANLRKKIDYMFNEKNKLENQQRMMQDLIEEYQCRYKEENNRKEHLENEFILLKQQV
DNEFKEQKERELKRELLMENKEFLKYFFTEEHAILN-CHP--PDTSVVLNMD-NSRGLDM
DVLIKNIDSWYHNIAQRSKEEANLFYQNQIEDLQNKKCQCRESLQKTSDEITELNRLVHL
MQSKLDNEKKKTAALQIAIGDIEHKGDHALKDARAKQTELQNRIQNSKDQLAVILRDYQD
LMNTKLALDIEIATYNTML-EGE
>Snake_HBS3
EKEEMKILNNQFACFIDKVRSLEQQNKVLSTKW-ELLNQYVQ-----PTR-KNLEAY--Y
GNFIGSLKKQLECVLTEREKLEHEQKNMQELVEEYRSRYEDEVNRRTTAENDFVVLKKDV
DCVFLTKEELESKVDVLAQELEVLRCIFAEELTQFN-HQI--CDTSVVLKMD-NTRALDM
DLILKNVEAWYQNIAHTSKQEAEAFYHSKIAEIQNNRGKFNEELKNNQHEIAELNRVVQR
LQADTDNAKKQVSSLQSAICDSEQRGDIALKDARTKHLELQTAYQQSKDKLACLLRDYQE
LLNTKLALDIEIATYKALL-EGE
>Snake_HBS4
EREEMKSLNNQFASFIDKVRNLELQNKVLETKW-NLLQEHVI-----PAK-KSLEPY--Y
ENFISNMKKQLECLLNNQEHLRNENDGIEQLVEDFKCNYEEEFKRRTDAENEFVLLKKDV
DSISLSKSELEGKVHLLSGELEFRRGIYAEELAQLS-TNV--YDTNILLQMD-NSRNLNI
DSIIKSVEAWYKNVAQGSKEEVNAFYENRFQELQQQRGKYSQTLKINQQEIADLTRLVHK
LQSEYDIIKKQVNGFQTSVCEIEQHGDCVLKDARDKHIDLQTAVQKAKDDLARLLRDYQE
LLNTKLALDIEIATYKTLL-EGE
>Snake_K78LT
EREQIKTLNDKFVSFIDKVKHLEQQNKLLETKW-NCLQQQEP-----VEK-ANIQPL--F
ENYITSLKQQLQRLLNEREQLQLEQSKFQDMVEEFKSRYEEEINHRMSAENNFVLLKKDV
DIAYSNKVDLEVKVESLRQELNFIRCVFEAEIESLQ-AAN--AETNVIVSMD-NSRVLDM
EGIIHSVRSQYEEIAQKSKDEVNALYENKYKELQNTWGNYCNNLSSGRHEIQELTRMVQR
CRSEVESTKKQIDALQTSIADNEQRGESALKDAKLKLTEVENNLQSSKDELARLLRDYQN
VLNVKIALDIEIAMYKSLL-EGE
>Snake_K78L4
EREQIKTLNNKFASFIDKVRFLEQQNKVLETKW-DLLCQQGSP----ALR-DNLEPI--Y
EAFIEALRRQFDSLCNDKNHLDIELKNTQELVEDFKNKYEDEINKRTACENDFVVLKKDV
DGSYMHKIDLGSKADSLTQYIQFLRALFDAELTQLH-SQV--KETNVILQMD-NNRDLDL
DSIIAEVKAQYEDIANRSRAEAEAWYQTKFQELQSTAFKHGDDLKTTKDEIADLSRIVQR
IRSEIEIVKKAISSLQSSLGEAEQRGELALKDARTKLLDLQTALTSAKDELARLLRDYQE
LMNVKLALDVEIATYRTLL-EGE
>Snake_K78L3
EREQIKTLNNKFASFIDKVRFLEQQNKVLETKW-NLLQQHGSS----VPK-LSLEPL--F
ENYILNLRRQLDSLIGNRAKLDSDLRNMQDLVEDYRRKYEEEINKRTTAENDFVGIKKDV
DVSFMQKVDLQAKVDRLIDEINFLKALHEAELIQIQ-GQI--SDTNVVLQMD-NNRDLDL
NSIIAEVKAQYEDIANRSRAETEAWYQNKFQELQIIAVGHGDDLKTIKNEIRELNRLINR
LKAETENVIKTISRIQKAIADAEERGQLALKDAQVKLNDLQQAIQNSKDELARLLRDYQE
LMNVKLALDIEIATYRTLL-EGE
>Snake_K78L2
EKEQIKVLNNQFASFIDKVRFLEQQNKVLQTKW-QILQRQSQGI---GPT-MNLDAD--F
QQFLNSLRSQIENIRSQKGQLTTELQSMQHYVEEYKNKYEEEINRRTTAENDFVVLKKDV
DCAYLSKSNLDSRVGALADQINFLRRIFEVEISQYQ-TGG--QDTSVVVSMD-NNTHLNL
EGIIDEVRSQYEEIARSSRAEAESWYHSQYEALQNTAGRHGDNLRNTRQEIQELTRNIQR
LRSEIEHVKKQXXX----------------------------------------------
-----------------------
>Snake_K78L1
EREQIKTLNNKFASFIDKVRFLEQQNKVLETKW-KLLQEQGQGT---GGQ-KNLDHF--F
EAYISNLRRQLDGLLNERRGFESELKNFQDLVEDFKNKYEEEINKRTAAENDFVLLKKDV
DAAYMNKVELQAKMDALTDEINFLRCMFEAELNQMQ-QSV--SDTSVVLQMD-NNRSLDL
NSIIAEVKAQYEDIAQKSRAEAESWYQCKYEELQVSAGRHGDNLRDTKTEIAELNRTIQR
MRAEIENVKKQCEKLQASIAEAEERGELALKDAREKLSELEEALQKSKEEMARLLKEYQE
LMNVKLALDIEIATYRKLL-EGE
>Snake_K8
EKEEIKTLNNKFANLIDKVRFLEQQNKMLETKW-SLLQNQK------TTR-SNMDSM--F
EAYINNLRRQLENLGQERARLDAELGNMQGLVEEFKNKYEDEINHRTEKENEFVLLKKDV
DEAYMNKVELESRMECLTDEINFLRQFYDEELRVLQ-SQI--SDTSVILSMD-NNRSLDL
DGIITDVKAQYEEIANRSRAEAERMYQIKYEELQTVAGKHGDDLRSTKNEINELSRMINR
IQSEIDGLKGQRASLEAAITEAEERGELAVKDANNKLAELESAMQRAKQDMARQLREYQE
LLNTKLALDIEIATYRKLL-EGE
>Human_K1
EREQIKSLNNQFASFIDKVRFLEQQNQVLQTKW-ELLQQVDTS----TRT-HNLEPY--F
ESFINNLRRRVDQLKSDQSRLDSELKNMQDMVEDYRNKYEDEINKRTNAENEFVTIKKDV
DGAYMTKVDLQAKLDNLQQEIDFLTALYQAELSQMQ-TQI--SETNVILSMD-NNRSLDL
DSIIAEVKAQYEDIAQKSKAEAESLYQSKYEELQITAGRHGDSVRNSKIEISELNRVIQR
LRSEIDNVKKQISNLQQSISDAEQRGENALKDAKNKLNDLEDALQQAKEDLARLLRDYQE
LMNTKLALDLEIATYRTLL-EGE
>Human_K2
EREQIKTLNNKFASFIDKVRFLEQQNQVLQTKW-ELLQQMNVG----TRP-INLEPI--F
QGYIDSLKRYLDGLTAERTSQNSELNNMQDLVEDYKKKYEDEINKRTAAENDFVTLKKDV
DNAYMIKVELQSKVDLLNQEIEFLKVLYDAEISQIH-QSV--TDTNVILSMD-NSRNLDL
DSIIAEVKAQYEEIAQRSKEEAEALYHSKYEELQVTVGRHGDSLKEIKIEISELNRVIQR
LQGEIAHVKKQCKNVQDAIADAEQRGEHALKDARNKLNDLEEALQQAKEDLARLLRDYQE
LMNVKLALDVEIATYRKLL-EGE
>Human_K3
EREQIKTLNNKFASFIDKVRFLEQQNKVLETKW-NLLQQQGTSSI--SGT-NNLEPL--F
ENHINYLRSYLDNILGERGRLDSELKNMEDLVEDFKKKYEDEINKRTAAENEFVTLKKDV
DSAYMNKVELQAKVDALIDEIDFLRTLYDAELSQMQ-SHI--SDTSVVLSMD-NNRSLDL
DSIIAEVRAQYEDIAQRSKAEAEALYQTKLGELQTTAGRHGDDLRNTKSEIIELNRMIQR
LRAEIEGVKKQNANLQTAIAEAEQHGEMALKDANAKLQELQAALQQAKDDLARLLRDYQE
LMNVKLALDVEIATYRKLL-EGE
>Human_K4
EREQIKLLNNKFASFIDKVQFLEQQNKVLETKW-NLLQQQTTT----TSS-KNLEPL--F
ETYLSVLRKQLDTLGNDKGRLQSELKTMQDSVEDFKTKYEEEINKRTAAENDFVVLKKDV
DAAYLNKVELEAKVDSLNDEINFLKVLYDAELSQMQ-THV--SDTSVVLSMD-NNRNLDL
DSIIAEVRAQYEEIAQRSKAEAEALYQTKVQQLQISVDQHGDNLKNTKSEIAELNRMIQR
LRAEIENIKKQCQTLQVSVADAEQRGENALKDAHSKRVELEAALQQAKEELARMLREYQE
LMSVKLALDIEIATYRKLL-EGE
>Human_K5
EREQIKTLNNKFASFIDKVRFLEQQNKVLDTKW-TLLQEQGTK----TVR-QNLEPL--F
EQYINNLRRQLDSIVGERGRLDSELRNMQDLVEDFKNKYEDEINKRTTAENEFVMLKKDV
DAAYMNKVELEAKVDALMDEINFMKMFFDAELSQMQ-THV--SDTSVVLSMD-NNRNLDL
DSIIAEVKAQYEEIANRSRTEAESWYQTKYEELQQTAGRHGDDLRNTKHEISEMNRMIQR
LRAEIDNVKKQCANLQNAIADAEQRGELALKDARNKLAELEEALQKAKQDMARLLREYQE
LMNTKLALDVEIATYRKLL-EGE
>Human_K6B
EREQIKTLNNKFASFIDKVRFLEQQNKVLDTKW-TLLQEQGTK----TVR-QNLEPL--F
EQYINNLRRQLDNIVGERGRLDSELRNMQDLVEDLKNKYEDEINKRTAAENEFVTLKKDV
DAAYMNKVELQAKADTLTDEINFLRALYDAELSQMQ-THI--SDTSVVLSMD-NNRNLDL
DSIIAEVKAQYEEIAQRSRAEAESWYQTKYEELQITAGRHGDDLRNTKQEIAEINRMIQR
LRSEIDHVKKQCANLQAAIADAEQRGEMALKDAKNKLEGLEDALQKAKQDLARLLKEYQE
LMNVKLALDVEIATYRKLL-EGE
```

```
>Human_K6C
EREQIKTLNNKFASFIDKVRFLEQQNKVLDTKW-TLLQEQGTK----TVR-QNLEPL--F
EQYINNLRRQLDSIVGERGRLDSELRNMQDLVEDLKNKYEDEINKRTAAENEFVTLKKDV
DAAYMNKVELQAKADTLTDEINFLRALYDAELSQMQ-THI--SDTSVVLSMD-NNRNLDL
DSIIAEVKAQYEEIAQRSRAEAESWYQTKYEELQVTAGRHGDDLRNTKQEIAEINRMIQR
LRSEIDHVKKQCASLQAAIADAEQRGEMALKDAKNKLEGLEDALQKAKQDLARLLKEYQE
LMNVKLALDVEIATYRKLL-EGE
>Human_K6A
EREQIKTLNNKFASFIDKVRFLEQQNKVLETKW-TLLQEQGTK----TVR-QNLEPL--F
EQYINNLRRQLDSIVGERGRLDSELRGMQDLVEDFKNKYEDEINKRTAAENEFVTLKKDV
DAAYMNKVELQAKADTLTDEINFLRALYDAELSQMQ-THI--SDTSVVLSMD-NNRNLDL
DSIIAEVKAQYEEIAQRSRAEAESWYQTKYEELQVTAGRHGDDLRNTKQEIAEINRMIQR
LRSEIDHVKKQCANLQAAIADAEQRGEMALKDAKNKLEGLEDALQKAKQDLARLLKEYQE
LMNVKLALDVEIATYRKLL-EGE
>Human_K7
ESEQIKTLNNKFASFIDKVRFLEQQNKLLETKW-TLLQEQKS-----AKS-SRLPDI--F
EAQIAGLRGQLEALQVDGGRLEAELRSMQDVVEDFKNKYEDEINHRTAAENEFVVLKKDV
DAAYMSKVELEAKVDALNDEINFLRTLNETELTELQ-SQI--SDTSVVLSMD-NSRSLDL
DGIIAEVKAQYEEMAKCSRAEAEAWYQTKFETLQAQAGKHGDDLRNTRNEISEMNRAIQR
LQAEIDNIKNQRAKLEAAIAEAEERGELALKDARAKQEELEAALQRGKQDMARQLREYQE
LMSVKLALDIEIATYRKLL-EGE
>Human_K8
EKEQIKTLNNKFASFIDKVRFLEQQNKMLETKW-SLLQQQK------TAR-SNMDNM--F
ESYINNLRRQLETLGQEKLKLEAELGNMQGLVEDFKNKYEDEINKRTEMENEFVLIKKDV
DEAYMNKVELESRLEGLTDEINFLRQLYEEEIRELQ-SQI--SDTSVVLSMD-NSRSLDM
DSIIAEVKAQYEDIANRSRAEAESMYQIKYEELQSLAGKHGDDLRRTKTEISEMNRNISR
LQAEIEGLKGQRASLEAAIADAEQRGELAIKDANAKLSELEAALQRAKQDMARQLREYQE
LMNVKLALDIEIATYRKLL-EGE
>Human_K71
EREQIKALNNKFASFIDKVRFLEQQNQVLETKW-ELLQQLDLN----NCK-NNLEPI--L
EGYISNLRKQLETLSGDRVRLDSELRNVRDVVEDYKKRYEEEINKRTAAENEFVLLKKDV
DAAYANKVELQAKVESMDQEIKFFRCLFEAEITQIQ-SHI--SDMSVILSMD-NNRNLDL
DSIIDEVRTQYEEIALKSKAEAEALYQTKFQELQLAAGRHGDDLKNTKNEISELTRLIQR
IRSEIENVKKQASNLETAIADAEQRGDNALKDARAKLDELEGALHQAKEELARMLREYQE
LMSLKLALDMEIATYRKLL-ESE
>Human_K72
EREQIKALNNKFASFIDKVRFLEQQNQVLETKW-NLLQQLDLN----NCR-KNLEPI--Y
EGYISNLQKQLEMLSGDGVRLDSELRNMQDLVEDYKKRYEVEINRRTAAENEFVVLKKDV
DAAYMNKVELQAKVDSLTDEIKFFKCLYEGEITQIQ-SHI--SDTSIVLSMD-NNRDLDL
DSIIAEVRAQYEEIALKSKAEAETLYQTKIQELQVTAGQHGDDLKLTKAEISELNRLIQR
IRSEIGNVKKQCADLETAIADAEQRGDCALKDARAKLDELEGALHQAKEELARMLREYQE
LVSLKLALDMEIATYRKLL-ESE
>Human_K73
EREQIKVLNNKFASFIDKVRFLEQQNQVLETKW-ELLQQLDLN----NCK-NNLEPI--L
EGYISNLRKQLETLSGDRVRLDSELRSVREVVEDYKKRYEEEINKRTTAENEFVVLKKDV
DAAYTSKVELQAKVDALDGEIKFFKCLYEGETAQIQ-SHI--SDTSIILSMD-NNRNLDL
DSIIAEVRAQYEEIARKSKAEAEALYQTKFQELQLAAGRHGDDLKHTKNEISELTRLIQR
LRSEIESVKKQCANLETAIADAEQRGDCALKDARAKLDELEGALQQAKEELARMLREYQE
LLSVKLSLDIEIATYRKLL-EGE
>Human_K74
EREQIKVLNDKFASFIDKVRFLEQQNQVLETKW-ELLQQLDLN----NCK-KNLEPI--L
EGYISNLRKQLETLSGDRVRLDSELRSMRDLVEDYKKRYEVEINRRTTAENEFVVLKKDA
DAAYAVKVELQAKVDSLDKEIKFLKCLYDAEIAQIQ-THA--SETSVILSMD-NNRDLDL
DSIIAEVRMHYEEIALKSKAEAEALYQTKIQELQLAASRHGDDLKHTRSEMVELNRLIQR
IRCEIGNVKKQRASLETAIADAEQRGDNALKDAQAKLDELEGALHQAKEELARMLREYQE
LMSLKLALDMEIATYRKLL-EGE
>Human_K75
EREQIKTLNNKFASFIDKVRFLEQQNKVLETKW-ALLQEQGSR----TVR-QNLEPL--F
DSYTSELRRQLESITTERGRLEAELRNMQDVVEDFKVRYEDEINKRTAAENEFVALKKDV
DAAYMNKVELEAKVKSLPEEINFIHSVFDAELSQLQ-TQV--GDTSVVLSMD-NNRNLDL
DSIIAEVKAQYEDIANRSRAEAESWYQTKYEELQVTAGRHGDDLRNTKQEISEMNRMIQR
LRAEIDSVKKQCSSLQTAIADAEQRGELALKDARAKLVDLEEALQKAKQDMARLLREYQE
LMNIKLALDVEIATYRKLL-EGE
>Human_K76
EREQIKTLNNKFASFIDKVRFLEQQNKVLETKW-ELLQQQTTG----SGP-SSLEPC--F
ESYISFLCKQLDSLLGERGNLEGELKSMQDLVEDFKKKYEDEINKRTAAENEFVGLKKDV
DAAFMNKVELQAKVDSLTDEVSFLRTLYEMELSQMQ-SHA--SDTSVVLSMD-NNRCLDL
GSIIAEVRAQYEEIAQRSKSEAEALYQTKLGELQTTAGRHGDDLRNTKSEIMELNRMIQR
LRAEIENVKKQNANLQTAIAEAEQRGEMALKDANAKLQDLQTALQKAKDDLARLLRDYQE
LMNVKLALDVEIATYRKLL-EGE
>Human_K77
EREQIMVLNNKFASFIDKVRFLEQQNQVLQTKW-ELLQQVNTS----TGT-NNLEPL--L
ENYIGDLRRQVDLLSAEQMRQNAEVRSMQDVVEDYKSKYEDEINKRTGSENDFVVLKKDV
DAAYVSKVDLESRVDTLTGEVNFLKYLFLTELSQVQ-THI--SDTNVILSMD-NNRSLDL
DSIIDAVRTQYELIAQRSKDEAEALYQTKYQELQITAGRHGDDLKNSKMEIAELNRTVQR
LQAEISNVKKQIEQMQSLISDAEERGEQALQDAWQKLQDLEEALQQSKEELARLLRDYQA
MLGVKLSLDVEIATYRQLL-EGE
>Human_K78
ETQEIRTLNNQFASFIDKVRFLEQQNKVLETKW-HLLQQQGLS----GSQ-QGLEPV--F
EACLDQLRKQLEQLQGERGALDAELKACRDQEEEYKSKYEEEAHRRATLENDFVVLKKDV
DGVFLSKMELEGKLEALREYLYFLKHLNEEELGQLQ-TQA--SDTSVVLSMD-NNRYLDF
SSIITEVRARYEEIARSSKAEAEALYQTKYQELQVSAQLHGDRMQETKVQISQLHQEIQR
LQSQTENLKKQNASLQAAITDAEQRGELALKDAQAKVDELEAALRMAKQNLARLLCEYQE
LTSTKLSLDVEIATYRRLL-EGE
>Human_K79
EREQIKTLNNKFASFIDKVRFLEQQNKVLETKW-ALLQEQGQNLG--VTR-NNLEPL--F
EAYLGSMRSTLDRLQSERGRLDSELRNVQDLVEDFKNKYEDEINKHTAAENEFVVLKKDV
DAAYMGRMDLHGKVGTLTQEIDFLQQLYEMELSQVQ-THV--SNTNVVLSMD-NNRNLDL
DSIIAEVKAQYELIAQRSRAEAEAWYQTKYEELQVTAGKHGDNLRDTKNEIAELTRTIQR
LQGEADAAKKQCQQLQTAIAEAEQRGELALKDAQKKLGDLDVALHQAKEDLTRLLRDYQE
LMNVKLALDVEIATYRKLL-ESE
>Human_K80
EKEEMKALNDKFASLIGKVQALEQRNQLLETRW-SFLQGQD------SAI-FDLGHL--Y
EEYQGRLQEELRKVSQERGQLEANLLQVLEKVEEFRIRYEDEISKRTDMEFTFVQLKKDL
DAECLHRTELETKLKSLESFVELMKTIYEQELKDLA-AQV--KDVSVTVGMD-SRCHIDL
SGIVEEVKAQYDAVAARSLEEAEAYSRSQLEEQAARSAEYGSSLQSSRSEIADLNVRIQK
LRSQILSVKSHCLKLEENIKTAEEQGELAFQDAKTKLAQLEAALQQAKQDMARQLRKYQE
LMNVKLALDIEIATYRKLV-EGE
>Human_K81
EKEQIKSLNSRFAAFIDKVRFLEQQNKLLETKL-QFYQNRE------CCQ-SNLEPL--F
EGYIETLRREAECVEADSGRLASELNHVQEVLEGYKKKYEEEVSLRATAENEFVALKKDV
DCAYLRKSDLEANVEALIQEIDFLRRLYEEEILILQ-SHI--SDTSVVVKLD-NSRDLNM
DCIIAEIKAQYDDIVTRSRAEAESWYRSKCEEMKATVIRHGETLRRTKEEINELNRMIQR
LTAEVENAKCQNSKLEAAVAQSEQQGEAALSDARCKLAELEGALQKAKQDMACLIREYQE
```

```
VMNSKLGLDIEIATYRRLL-EGE
>Human_K82
EKEQIKCLNNRFASFINKVRFLEQKNKLLETKW-NFMQQQR------CCQ-TNIEPI--F
EGYISALRRQLDCVSGDRVRLESELCSLQAALEGYKKKYEEELSLRPCVENEFVALKKDV
DTAFLMKADLETNAEALVQEIDFLKSLYEEEICLLQ-SQI--SETSVIVKMD-NSRELDV
DGIIAEIKAQYDDIASRSKAEAEAWYQCRYEELRVTAGNHCDNLRNRKNEILEMNKLIQR
LQQETENVKAQRCKLEGAIAEAEQQGEAALNDAKCKLAGLEEALQKAKQDMACLLKEYQE
VMNSKLGLDIEIATYRRLL-EGE
>Human_K83
EKEQIKSLNSRFAAFIDKVRFLEQQNKLLETKL-QFYQNRE------CCQ-SNLEPL--F
AGYIETLRREAECVEADSGRLASELNHVQEVLEGYKKKYEEEVALRATAENEFVALKKDV
DCAYLRKSDLEANVEALIQEIDFLRRLYEEEIRILQ-SHI--SDTSVVVKLD-NSRDLNM
DCIVAEIKAQYDDIATRSRAEAESWYRSKCEEMKATVIRHGETLRRTKEEINELNRMIQR
LTAEVENAKCQNSKLEAAVAQSEQQGEAALSDARCKLAELEGALQKAKQDMACLIREYQE
VMNSKLGLDIEIATYRRLL-EGE
>Human_K84
EKEQIKTLNNKFASFIDKVRFLEQQNKLLETKW-SFLQEQK------CIR-SNLEPL--F
ESYITNLRRQLEVLVSDQARLQAERNHLQDVLEGFKKKYEEEVVCRANAENEFVALKKDV
DAAFMNKSDLEANVDTLTQEIDFLKTLYMEEIQLLQ-SHI--SETSVIVKMD-NSRDLNL
DGIIAEVKAQYEEVARRSRADAEAWYQTKYEEMQVTAGQHCDNLRNIRNEINELTRLIQR
LKAEIEHAKAQRAKLEAAVAEAEQQGEATLSDAKCKLADLECALQQAKQDMARQLCEYQE
LMNAKLGLDIEIATYRRLL-EGE
>Human_K85
EKEQIKSLNSRFAAFIDKVRFLEQQNKLLETKW-QFYQNQR------CCE-SNLEPL--F
SGYIETLRREAECVEADSGRLASELNHVQEVLEGYKKKYEEEVALRATAENEFVVLKKDV
DCAYLRKSDLEANVEALVEESSFLRRLYEEEIRVLQ-AHI--SDTSVIVKMD-NSRDLNM
DCIIAEIKAQYDDVASRSRAEAESWYRSKCEEMKATVIRHGETLRRTKEEINELNRMIQR
LTAEIENAKCQRAKLEAAVAEAEQQGEAALSDARCKLAELEGALQKAKQDMACLLKEYQE
VMNSKLGLDIEIATYRRLL-EGE
>Human_K86
EKEQIKSLNSRFAAFIDKVRFLEQQNKLLETKL-QFYQNRE------CCQ-SNLEPL--F
EGYIETLRREAECVEADSGRLASELNHVQEVLEGYKKKYEEEVSLRATAENEFVALKKDV
DCAYLRKSDLEANVEALIQEIDFLRRLYEEEIRVLQ-SHI--SDTSVVVKLD-NSRDLNM
DCIIAEIKAQYDDIVTRSRAEAESWYRSKCEEMKATVIRHGETLRRTKEEINELNRMIQR
LTAEVENAKCQNSKLEAAVAQSEQQGEAALSDARCKLAELEGALQKAKQDMACLIREYQE
VMNSKLGLDIEIATYRRLL-EGE
>Frog1_K18
EKETMQDLNDRLASYLERVRSLEAANKKLEVQIRQHTEKKGPS--------KDWSPY--Y
KT-IEDLRKQVFDSTLENSQLVLQIDNARLAADDFRVKYEAEMAIRMSVEGDITGLRKLI
DDTNVSRMNLENEIESLKEELIFLKKNHQDDVTELQ-AQV--ARSAVTVEVD-APKSQDL
GKIMTELRAQYDGLAQKNRDDVEKWYQSKVDEHTVQVNLDTEALHSAKSSVTDLRRTVQS
LEIELESLRNQKASLEGTLHDTEARYAMELEMLGGTAMAMEAELVQVRSDCQRQQQEYQA
LLNTKMKLEAEIHTYRRLL-EGD
>Frog1_02 13C
----MQNLNDRLAAYLNKVRALEEANSDLECKIREWYEKHRNAC---MPD-RDYSKY--Y
HI-IEDLKKQIQCASVENARVILQIDNARLAADDFKLKYENELCLRHGVEADINGLRKVL
DELNLAKCDLESQIESLTDEIACLKKNHEEEMKGYQ-GAT----GHLSVEMK-AAPGNDL
TKHLNDMRAEYEELAEKNRKEAEARFNEASRALKQEISCGAEQIQCSKNEITDLKRNLQA
LEIDLQAALAMKKTLECSLAETEGNYCVQLSKIQAKISALEQQLCEVRTDMERQSIEYEQ
LLDIKTRLEMEIETYRRLL-DGE
>Frog1_03
EKQTMQNLNDRLASYLDKVHELEGKNAELERKIKDWYEKHGPAPP--EAP-RDYSKY--F
KE-IEALKAQIIAASKQNAVILLQCDNARLAADDFKQKFENEQAIRYSVEADINGLRRVM
DDLTLSRAELEGQLESLTEELAYLKKNHDDEVRSIKVTEA----GKVSVEMN-AAPSNDL
TKRLNDMRAQYEDLARNNRQEAENNFNRISADLKNQMHHVQITMDESRTEVTNLKKTLQS
LQIELQSQLAQKNSLEILLAETEARFCMKLSHLQETIACVEQRLEQLRAESECQRTEYQQ
LLEMKTKLEGEIATYRSLL-D--
>Frog1_04
EKETMQNLNDRLASYLDKVNALEAANAELERKIKEWYDKQKPSTTTGEGA-KDYSKY--Y
KD-IEDLKAKIIAASKENAALLLQCDNARLAADDFKQKYESELHLRQTVEADINGLRRVM
DELNLAKTDLDSQFQGLTEELASLKKNHEDEVKGVQVTEA----GKVKVEMN-AAPSNDL
TKRLNDMRAQYEDLAKKNRQELENKFNAATGPVKEKIKEDVKEDTTVKTQISDLKRSLQA
LEIELQSLLAMKKSLELQLAETEGRFCMKLSVLQENIASIEQHLEQLRAESECQKDEYDQ
LLEMKTKLENEIKTYQSLL-D--
>Frog1_05
-XXXMQNLNDRLANYLDKVNALEAANAELERKIKEWYDKQPTTS---DGG-KDYSKY--Y
SL-IDELNLKITNATKDNAGITLQADNARLAADDFNNKYENERALYQMMDNDINGLRRVV
DDFTLSKFDLESQFEGLFEEMVFIKKKHDDEIKGTQNTKL----SQVSVEMK-AQPGTDL
TKILNDMRAQHEALAEKNRREAEDRYNTMTAELRQQISAGAGQMQASKTEISERKRTLQA
LETDLHSKLSMKQSLELMLAETEGIYCMKLSRIQVTISSIEEHLAQLKADSESQHSQYQQ
LLGIKTRLEQEIETYRRLL-DGE
>Frog1_06
EKETMQNLNDRLANYLDKVNALEAANAELERKIKEWYDKQPTT----SDGGKDYNKY--Y
SL-INELNQKILNAALDNAELALHCENAKLAAEDFRLKYESEWTLSQAVVSDINGLRKLM
DDLTLSKSSLESQIESLSEELAYLNKTHDEEIKGTQ--NT--KLSQVSVEMK-AQPGTDL
TKILNDMRAQHEALAQKNRREAEDRYNKMSAELRQQISAGVGQVQTSKTETSELKKTLQA
LEIELQAQIAKKQSLELMLAETEGSYCMKLSRIQVTISSIEEHLAQLKGDSECQRSKYEQ
LLGIKIRLEQEIEMYRKLL-EGG
>Frog1_07
KKETMQNLNDRLANYVDKVRVLEAANAELEWKINEWYDKQRSTS---DGG-KDYSKY--A
TL-IDDLKLKILSTTKDNRGIPLQSDNARLAIDNFRLKYENEQILHHSMEAAINTLHRVI
DDLTLSKSKLQSQLESLTEELAYLKKNHDEETKGAP-KAT---RGQVSVELK-ATPGTDL
TKILNDMRAQHEALAQKNRREAEDRYEKLSADLKQRISAGAGRVQTGKSEISELQRTLRA
LEIELQSQLAMKQSLEVTVGKTEGSYCRKLSRIQVTISSIEEHLAQLKADSESQHSQYQQ
LLGIKTRLEQEIETYRRLL-DGE
>Frog1_08
EKHTMQNLNDRLANYLDKVKALETDNNDLEKKIREWYEKLRPESGG-VGT-VDYSKY--L
PI-IEDLRKKIMESTLENAKILLQTDNARLAADDFRLKYENELALRQSVEADINGLRRVL
DELTLCKADLELQIESLTEELAYLKKNHKEELDALR-GGP---AGQLTVEMN-AAPAVDL
TKLLNDMREQYETLAEKNRRETEARFNEQSKDIKKEILAGVQQVQSNTTEISDLKRSLQG
LEIELQSQLAMKQSLEKTLAETEGRFCSQLGQLQNLITSVEEQLFQLRNDMELQSNEYKQ
LLDIKTRLEQEIETYRKLL-EGE
>Frog1_09
DKQTMQNLNDRLAAYLEKVRALEAANADLELKIREWYEKQRTSGIG-AGA-KDFSKY--F
DI-ITDLRSKILFATIDNSRVVLQIDNAKLAADDFRLKFENELALRQSVEADINGLRRVL
DELTLARGDLEMQIESLTEELAYLKKNHEEEMSIAK-SSA---AGQVTVEMD-AAPGTDL
TNILNNMRADYETLAEKNRRDAELWFNQKSGELKKEILTGVELVQSSKSEISDLRRSLQS
LEIELQSQLAMKKSLEDSLAETDGRYGAQLQNIQLSIRSLEEQLLQIRSDMERQNMEYKQ
LLDIKTRLEMEIETYRRLL-EGE
>Frog1_10
EKHAMQNLNDRLASYLDKVKALEASNVTLEGKIHDWYDSKVDAGI--GAGSKDYSKY--F
EI-INELRSKIGAATVGNATVTLQIDNARLAADDFRLKFENELALRQSVEADIAGLRRVL
DELTLARGDLEMQIESLTEELAYLKKNHEEEMSHAK-SQS---AGKVSVEMD-AAPGVDL
TNILNNMRADYEILAEKNRRDAELWFNQKSGELKKEISVGVEQVQASKSEITELKRSLQS
```

LEIELQSQLAMKQSVEGNLSEVQGFYSAQLLQIQNTISSLEEQLLQIRSDMEHQNMEYKQ
LLDIKTRLEMEIETYRRLL-EGE
>Frog1_11
EKHAMQNLNDRLASYLEKVRALEASNADLEGKIRTWHEKQTGTGLGAGS--KDYSKY--F
EI-ISELRGKIHGATVDNATVTLQIDNARLAADDFRLKFENELALRQSVEADIAGLRRVL
DELTLARGDLEMQIESLTEELAYLKKNHEEEMMHAK-SQS---AGKVSVEMD-AAPGVDL
TNILNNMRADYEILAEKNRRDAELWFTQKSGELKKEISVGVEQVQASKSEITELKRSLQS
LEIELQSQLAMKQSVEGNLNELQGFYSAQLLQIQNTISSLEEQLLQIRSDMEHQNMEYKQ
LLDIKTRLEMEIETYRRLL-DGE
>Frog1_12
EKQTMQNLNDRLATYLEKVRSLEKSNSKIELQIKEWHSKNAG-----VGE-KDYQGY--Y
KT-IEALQNEILNSTMVNAEILLKIDNAKLAAEDFRLKFESERSLKIGVEKDIIGLRKLI
DDLNLQRIDLEHQYESLQENLAYLRKNHEEEASVLR-NQI---TGHINVEVD-AAPSVDL
AKVLADMRAQCEAVVSKIKQDAKQQFETQIEEVNIQIGGNVTELEKHRSSVKEVKRHVQS
LEIELQSEISKKDALNATLNNINAQYAAQLLHMQTAIKCIEEQLIQIRSDMSRQSQEYEI
LLNIKLRLELEIETYRRLL-EGE
>Frog1_13
EKHTMQNLNDRLAAYLDKVRALETANHHLEVQIKEWYEANMHCY---KER-NDYSKY--M
VV-IEELRRQITNGTPANARILLQIDNAKLAADDFRMKYENELALRQSVESDVNGLRRVL
DELTLARSDLELQLEGLNEELAFLKKNHQEEISSIK---V--GSGQVTVEMD-AAPAVDL
TLLLNNMRADYEVVAEKNRKEVEEWFLKKSAALKQDILIEAQEVQSSHSEISELKRTQQT
LEIELQALLSMKSSLEGTLSETEGRYCLSLSQIQMRISSIEEQLHQIRMDMSRQNEDYLR
LLDVKTRLEREIETYRILL-EGE
>Frog1_14
EKETMRNLNNRLATYLEKVRSLEKANAELEGQIKEWYQQNSG-----FGK-RDDAHY--F
QV-IEELTKQIVNAKMENGKLLLQMDNATLALDDFKMKLEYELNLGAAVEKDLSELRKVV
DDLTMTRTDLESQIESLKEEMVYLKKNHTQDMVGLH-KQE----GTIDVEVD-AAPSVDL
GKTMEDMRSQYEQLVEKQRLEAKYGFDRKVEEWNEEVQTNTAELEKCRKDLSELKHKVQD
LEIESQAELSKRKAAGNALDNVNSRYTMELADKQEKIARFEEHLQQTRRDVGFQIQEFTI
LFDLKNRLEAEISTYRRLL-DAE
>Frog1_15
GKETMQNLNDRLASYLEKVRSLEEANTGLESKIQLWHRNRAQ-----NHK-RDYSVY--E
KT-IGELQSQLVDGHMNGAKIKLQMENAKLAFDAFKRKYETEKTIRTALEGDLESLRRAM
DNLTIVRTDLEMEVEGMRKELIYMRMGHEEDIKLAQ-FQK--KGASVDVKID-APPSVDL
AKTIAEIRKEYETLIEKNRREASEWYS---------------------------------
---------HQKHGLEGTLAETEANAAGQLQKIQATICRMEAELSNVRGELERQNIEYRA
LLDVKTRLENEIATYRELL-EGD
>Frog1_16
EKETMQQLNDRLANYLGRVRSLEEENGQFERRIREWYDKQVP-----YNF-PDFNNF--F
RT-IEELQNKILHACTGNANLILQIDNARLTSDDFRNKFESEAAFRMGVEGDMNGLRRVL
EELNLGNNDQEIQRQNLSEELTYLKKNHAEEVGSLR-TQL---GARVNVEVN-AAPAVDL
NQVLGEIRDQYETIVERNRREAENWFLNKSEELNQQMNSSADQLQTVHTEVIQLRNTVQG
LEIDLQSQNSMRSALEGTLADTESRYGGQLSQLQFLINNVETQLGSLRSDLERQNFEYKA
LMDVKTHLEMEIATYRRLL-DGE
>Frog1_17
EKYTMQNLNDRLASYLAKVKTLEVANTDLEAKIRDWYSKQGSV----AVSEQSFGTL--Y
TT-IGELRQKILAATINNSKLVLEIDNARLAADDFKLKYENEFSLRQTVENDISGLRHIL
DDLTMTRSDLELQIESLKEELIYLKANHEEEVSEKK-QHA---AGTVSVELD-AVPGVGL
LNTLNDLREQYESIADRNRREAEAWFLSQVEGLQKEVVSSTQQIQSTKSESTELRRGLQS
LEIELQSQLSAKAGLEASLAETEGRYAAQLFQIQNIISSMEAQLSDLRSDLERQNQEYRT
LLDIKSRLEQEIATYHQLL-EGQ
>Frog1_18
EKSTMQNLNDRLSNYLEKVRCLEETNADLEKKIHGWHENQGSVT---VHE-QNYLPY--F
AT-IDELRQKIHAAFIDKNRVLLGLDNARLAAEDFKFKYENEHSMRLNVETDINGLRKLL
DEMTLSRADLELQIENLKEELTYMKKNHQEEMGAKS-QQV---IGNVNVEMD-APPMNDL
TKILSEMRENYEYLMSKNRRDLETWYLSQSESLQNKVEYITE--QTSKSEITDVRRNVQG
LEAELQSQYSKKAAIEDALADTEERYAMQLSHIQSIIGGIEAQLYDLRYDLEHQNQEHKS
LLAIKSHLEAEIATYHKLL-EGQ
>Frog1_19
EKETMQNLNDRLAMYLDKVRALEEANSGLELKIREWYEKQGP-----SPS-RDYSHY--F
AI-IEDLRGKILDATVDNSKIVLQIDNARLAADDFRTKYENELALRQSVECDINGLRKVL
DELTLCRTDLEHQIESLKEELAYLKKNHEEEMNAMR-GQV---GGQVSVEVD-AAPTVDM
NKKLAEMREQYELLAEKNRRDAEAWFFSQSEELNKVVATHTEQIQTSKSEITDLRRTIQG
LEIELQSQLSMKAALENTLSETEARYGMQLAQIQNVIGSIEAQLADLRSDMERQNHEYKL
LMDIKTRLEKEISTYRQLL-EGH
>Frog1_20
--MTMQNLNDRLANYLDKVHALEEANAELEKKIKDWYNNHRPAS---SKA-PDYGEY--F
KT-IDDLQNKIIAAYANNHKAILAVDNTRLTIDDYRLKYENERALRQSAEADISGLRRVM
DDLTLCKSDLEAQVESLTEELAYLKKNHDEEVKAMQ-GQA---TGAVNVEMN-VAPSVDL
SKSMADMRAEYEKLAERYRREVEEQFQAMTKDLQKQVVASVREVQTSKSEITDLRNVLQS
LEIELQSQLSMKAALEASLAETEGQYCVKLSHIQELILRVQNNISDIWTQMETQSSEYKL
LLDIKSQLEQEIATYRELL-DAQ
>Frog1_21
EKITMQNLNDRLATYLNKVKSLEEANTDLEKKIRNWYDTHGPSP---VQE-KDYGPF--Y
RT-IEDLQKKILGATIDNGRIVLQIDNAKLAADDFRMKYENELILRQTVESDINGLKRLL
DDLTLNRSDLESQLENLKEELAGLKKNHEEEMKALK-VQL---VGDVTVDMK-AAPGIDL
QKVLGDLRHDYEQIMQKNQKDIEFWYQEKTAELRKNVSGSTQEIQTTNTQVTELRRTLQN
LEIDLQAQLSTKAALEASLAETEGRYCLQLSQLQALIQNVEAELANIRCEMENQSHEYKI
LLDIKTRLEQEINTYRNLL-DGQ
>Frog1_22
EKYTMQNLNDRLAAYLEKVRSLEKANGELEIKIRDWYQKQAP-----GPA-ADYSHY--Y
KT-IEDLRNKILSATMDNAGLLLQIDNAKLAADDFRTKYETELALRMNVEADINGLRRVL
DDLTINRSDLEIQIESLKEELAYLKKNHEEEMIALR-GQA---TGQVNVEMD-AAPSSDL
TKTLAEMRIQYENLAEKNRRDVEEWFFKKTEELNKEVANSSVMIQSSKTEITDLKRTLQS
LEIELQTQLSMKAALEGSLAETEGRYCVQLSQIQGLISSVEAQLGELRSDMERQSHEYKI
LMDVKTRLEQEIATYRRLL-EGE
>Frog1_K117
GKETMQNLNDRLASYLERVRSLEQANHELELKIREYCEKKAA-----IGS-PDVSGY--Y
NI-INLLRSQINDASIDNARLVLQIDNAKLAADDFKIKYESELAIRTGVESDIIGLRRVL
DELTLNKTDLELEIESLKEELIYLKKNHEEELAVAR-ASA---RGQVDVQVD-SAPPVDL
SQIMSDVRAQYEGMMEKNRQEVEAWYRGQSENLNKEVATNTAALQTSKSQITDLKRTIQS
LEIELQSLLSMKGALEGTLAETEARYGAELNHLQAMITQIELDLQNLRSDAERQSLEYKL
LLDAKTRLEMEISTYRRLL-EGE
>Chicken_K12
KKETMQNLNDRLAAYLDKVRSLEDANTELERKIREWYEKNGPGAAVPGSG-NDYSKY--Y
PI-IEDLRNKIINATIDNARIILQVDNARLAADDFRLKYENEVALRQSVEADINGLRRVL
DELTLTRADLEMQIESLNEELAYLKKNHEEELQGLQ-SSA---VGQVSVEMD-AAPGIDL
TKLLNDMRGQYEVIAEQNRKEAEAWFNEKSGELKREISTNTEQLQSGKSEITDLKRTLQN
LEIELQSQLAMKKSLEDTLAETEGGYCAQLSQMQLQIGNLESQLLQVRADMERQSAEYQQ
LLDIKSRLEMEIETYRRLL-DGE
>Chicken_K20
EKSTMQNLNDRLAAYLERVRSLEKANSLLEKQIKEWHEKNTV-----GVR-SDYSSY--F
QT-IEDLQSKIGAAQLENAKLVLQIDNAKLAADDFRLKYENELLLRQSVESDINGLLRVL
DDLTLTKSDLESQIENVNEEIVFLKKNHEEEVDRLR-KQV---GGSVNVEVD-AAPGTDL

ASIMENMRRQYEEMAEKNRQEAKERFEKQTEALNQEVAINVEQLQEQRREVTDRRQICQS
LELELQTHLNMKKPLEDTLAETEARYSFQLAQIQETIAKLEAQLRQLRADMEAQNTEYSV
LLDIKTRLELEIATYRRLL-EGE
>Chicken_K23
EKQTMQNLNDRLAAYLDKVRSLEAANAQLESCILEWHKTKSH-----GKR-HDFNQY--E
QN-VTDMQRQIEDGKITNASILLQIDNANMASEDFRLKYEAEKTRRQGVQCDLENLRKEL
DGLTIITTDLEMEIEGLREEHILRRKDHEEDMEANR-SSQ---DFKVNVKVN-APPPEDL
TKILAEIREDYEAIIEKNRQSLDSWYKELSTAVSLAADPNPEQVQSSENEIKDLTRTLQS
LDIELQAQMSKKHMLEDTLADTRNYYAVALQNMQQTISKCEEELSQLRHDIRQQHNQYRV
LLGIKTRLEKEISTYRLLL-EGN
>Chicken_K15
EKITMQNLNDRLATYLQKVRALEDANAELEVKIRDWYQKEAPS----SPA-RDYSNY--Y
KI-IEDLQDKILAATIDNSRVVLEIDNARLAADDFRLKHENELFLRQSVESDINGLRRVL
DELTLSRADLEMQIESLKEELAYLKKNHEEEMKEYS-NQL---SGKVSVEMD-AAPGIDL
TRILSEMREQYEALAEKYRKDAEAWFLTQTTELNHEVATHTEQIQSSKSEITELRRTMQS
LEIELQSQISMKAGLEANLRDTEGRYCAQLAQIQNLITSVEEQLSEIRCDMERQNQEYRM
LMDIKSRLEQEIATYRQLL-ETQ
>Chicken_K19
EKTTMQNLNDRLASYLDKVRALEEANSDLETKIREWYLKQGP-----GPA-RDYSPY--Y
KA-IEDLRDQILAATIDNSKVVLQIDNARLAADDFKTKFETEQALRMSVEADINGLRRVL
DELTLARTDLELQIENLKEELAYLKKNHEEEMSALG-GQV---AGQVSVEVD-SAPGIDL
SKILADMRDQYEHMAEKNRKDAEAWFHSKTEELNREVAVNTEQLQSSKSEVTDLRRTLQG
LEIELQSQLSMKGALESTLADTEGRYGAQLAQIQDMIGSIEAQLAELRADMERQNSEYKM
LMDIKTRLEQEIATYRQLL-EGQ
>Chicken_K9L1
EKITMQNLNDRLASYLDKVRRLEEDNAQLELHIREWYRKQAP-----SVS-KDYSSY--Y
QT-IEQLQNQIISATVDNNRMLLDIDNSKMTADDFRMKYENELVIRQSVESDVNGLRNLL
DDLTHTRSSLESELESLKEELIALKRNHEEELRQLQ-SQT---GGDVSVEVN-AAPGEDL
TKILNDLRNEYEQIIEKNRREVEQWYEVKIEEVNRQVTSSSQDIQTSSHQLTELKREMQN
LEIELQAQLSTKNSLENSLAETESRYGCLLQQIQGQINSVEEELANIRCEMEGQNQEYKM
LLGIKTRLEQEIAQYRALLNEGQ
>Chicken_K9L2
EKITMQNLNDRLASYLDKVRRLEEDNAQLELHIREWYRKQAP-----SVS-KDYSSY--Y
QT-IEQLQNQIISATVDNNRMLLDIDNSKMTADDFRMKYENELVIRQTVESDINGLRNML
DDLTLVRSSLESELESLKDELIALKRNHEEELRQLQ-SQT---GGDVSVEVN-AAPGEDL
TKILNDLRNEYEQIIEKNRREVEQWYEVKIEEVNRQVTSSSQDIQTSSHQLTELKREMQN
LEIELQAQLSTKDSLENSLAETESRYGCLLQQIQGQINSVEEELANIRCEMEGQSQEYKM
LLGIKTRLEQEIAQYRALLHEGQ
>Chicken_K9L3
EKLTMQNLNDRLASYMDKVRGLEEENAHLERLIREWYQKQGP-----TGS-KDYSQY--Y
RT-IEELQNQIVGANVDLNRMLLDIDNTRMTVDDFRLKYETEYTLHQSVASDINGLRPLL
DQLTLSRSDLETQFESLKEELIYLKKNHEEEMRGLQ-TQS---SGDVNVEVN-ATPGTNL
MEKLNEMRCEYERLIENNRREVESWYETKMEEVNQQVHSSGQEIQSSNQQISELRREYQS
LEIELQSQLSMVDSLQSNLEDTERRYNMQLQQIQGMIGPLEEELASIRCEMESQNEEYKM
LLGIKTRLEQEIAQYRALL-EEG
>Chicken_K9L4
EKLTMQNLNDRLASYLDKVRSLERENARLEQLIREWYQKQGP-----TGR-KDYSHY--Y
TT-IEDLQNQILTAAVETNKILLDLDNTRMTAEDFRTKYETEYGLRQNVEADINTLRPLL
DSLTLSRTDLEMQFESLKEEMINLKKDHEEDMKSLQ-TQS---SGDVNVEVN-AAPGEDL
LKKLNDMRQEYEQIIQKNREEVESWYESKMEEVSQQVHSSGQELESSNQQISVLRRDYQS
LEIELQSELSRVGALQSNLEDTERRYNMQLQQIQGMIGPLEEELASIRCEMESQNEEYKM
LLGIKTRLEQEIAQYRALLNEGQ
>Chicken_HAS1
EKVTMQNLNDRLASYLDKVKCLEKENAELECRIREWYATQGLS----CEP-RDYSCY--Y
KE-IEDLQNQIVCATIDNNKIILDIDNSRMAADDFRVKYETELALRQSVEADINGLRQVL
DQLTLCRSDLEAQLESLREELCCLKKNHEEEMNCLR-KQS---TGDVSVEVN-ACPGPDL
RQILEDLRCQYETLIARNRKEVEDWYECKIEEVNREVITSGQEVETCNNQVTELRRQLQA
LEIDLQAQLSQRNNLESSLAETECQYNTLLGELQNQITCVEQQLAEIRAEIECQNQEYKT
LLDVKCRLEQEIQTYRCLL-EGG
>Chicken_HAS2
EKVTMQNLNDRLASYLDKVRCLEQENADLECRIREWYAKQGPF----CEP-RDYSCY--Y
KE-IEDLQNQIVCATIDNNKIILNIDNSRMTADDFRVKYETELALRQSVEADINGLRQVL
DQLTLCRSDLEAQLESLREELCCLKKNHEEEMNCLR-KQS---TGDVSVEVN-ACPGPDL
RKILEEMRCQYETLIERNRKEVEDWYECKIEEVNREVITSGQEVETCNNQVTELRRQLQA
LEIDLQAQLSQRDNLESSLAETECRYNNHLGELQTQITCVEQQLADLRAEMECQNQEYKI
LLDVKCRLEQEIHTYRCLL-EGG
>Chicken_HAS3
EKATMQNLNDRLASYLDKVRLLESENAELECRIREWYAKVGPS----CEP-RDYSCF--H
KE-IEDLQNQILCAAMETNKILLNIDNSQMTAEDFRAKYETECSLRQNVDADICNLRPVL
DQLASCKADLQLQCEAVTEEMCCLRANHEEEMSCLR-KQA---TGDVSVEVN-ACPGPDL
RKILEELRCQYETLMERNRKETEQWFACKVEEVNLEVITSSQEIESSNKQVTELRRQLQG
LEINVQAQLTMKENLESSLAETECRYNKYLSELQSQISCVEQRLAEIRAEMECQNQEYKS
LLDVKCRLEQEIHTYRCLL-EGG
>Chicken_K14L1
EKETMQNLNDRLAAYLDKVRALEEANTDLEVKIREWYKKQGP-----GPE-RDYSPY--Y
RT-IEELRSKILAATVENANIVLQIDNARLAADDFRTKFETEQALRLSVEADINGLRRVL
DELTLSRADLEMQIENLKEELAYLKKNHEEEMNALR-GQV---GGEISVEMD-AAPGIDL
TKILAEMREQYESLAEKNRRDAEQWFFSKTEELNREVAVNTEQLQSGKTEITELRRTIQS
LEIDLQSQLSTKAALEGTLADTEARYGTQLAQLQALITSVEEQLAELRCDMERQNHEYRV
LLDVKCRLEQEIATYRRLL-EGE
>Chicken_K14L2
EKETMQNLNDRLAAYLDKVRALEEANTDLEVKIREWYKKQGP-----GPE-RDYSPY--Y
RT-IEELRSKVLGATVDNANLLLQIDNARLTADDFRTKFETEQALRLSVEADINGLRRVL
DELTLSRADLEMQIENLKEELAYLKKNHEEEMNALR-GQV---GGEISVEMD-AAPGIDL
TKILAEMREQYESLAEKNRRDAEQWFFSKTEELNREVAVNTEQLQSGKTEITELRRTIQS
LEIDLQSQLSTKAALEGTLADTEARYGTQLAQLQALITSVEEQLAELRCDMERQNHEYRV
LLDVKCRLEQEIATYRRLL-EGE
>Chicken_K18
EKETMQDLNERLATYLEKVRSLENNNRRLEAQIRESLAKRG------PST-RDWGNY--W
DT-IQQLRDKIYDSAVENARTVLQIDNARLAADDFRVKYEAELAIRTSVESDISGLRKVL
DDTNMARLQLEGEIEALREELIFMKKNHEEEVNALQ-GQA--ASAGLTVEVD-APQAQDL
GQALAELRAQYDALAKKNVEELEKQWGQQISETTLEVTQSTKEVDTARSRLLELRRSVQS
LEIDLDALRSQKRGLEGSLAETEARYGAQLEQLQGLILRGEEELAQARAELQRQNREHCA
LQDAKSKLEAEIATYRALL-EGG
>Alligator_K24
EKETMQNLNDRLANYLDKVHALEEANADLEYKIHQWYEKHGPAAA--ELT-RDYSKY--Y
QI-IEDLRNQIISATIGNASIVLQIDNSQLAAEDFRLKYENELFLHRSVEADSNGLRRVL
DDLTIAKSNLEAQLESLTEELVYLKKSHEEEMKSLK-STS---TGDVNVEIN-AAPGIDL
TKLLNDMRAQYESLAEQNRREAEEQFNKMSQPLQKQIYDDAGAVSSATNELTEMKRTIQG
LEIELQTVMAQKCSLEGTLARTEENYSAQLSQRQLHISNLEEQLQQIRSEMECQNAEYEQ
LLGIKIRLEMEIETYKRLL-DGE
>Alligator_K10L
EKETMQNLNDRLASYLGKVQALEEANAELEHKIKDWYETLGSYTE--GGS-RDYSKY--Y
TT-IEDLRNQIIAATVENASIILQTDNARLAVDDFKVKYENELYLRQNVEADIRGLRKVL

```
DEQTLAKSNLEIQIESLADEVAYLKKNHEEEMNDLR-SSA---SPDVNVEIN-TAPGIDL
TTRLNKMRTEYEALAEQNRKDVETWFNEKSKELNVQINFSAEETNTNKSQITELKRTFQG
LEIEMKSQLALKQSLEATLAETEARYCAQLSQIQVVISSVETQIQQIRDDMEWQNAEYEQ
LLDIKIHLENEIETYRHLI-DRE
>Alligator_K12
KKETMQNLNDRLAAYLDKVRSLEEANTELEHKIREWYEKNGPGAGSLGAG-NDYSKY--Y
PI-IEDLRNKIINATIDNARIVLQIDNARLAADDFRLKYENEVALRQSVEADINGLRRVL
DELTLTRADLEMQIESLNEELAYLKKNHEEELQGFQ-GNA---TGQVSVEMD-AAPGIDL
TKLLNDMRGQYEAIAEQNRKDAEAWFNEKSGELKKEISTNTEQLQSGRSEITDLKRTLQS
LEIELQSQLAMKKSLEDTLAETEAGYCTQLSQMQLQIGNLENQLFQVRADMERQNAEYQQ
LLDIKTRLEMEIETYRRLL-DGE
>Alligator_K20
EKLTMQNLNDRLASYLEKVHSLEKTNSQLEKQIREWYANNSS-----STR-QDYSPY--Y
NT-IEELRNKIGAAQLENARLILQIDNAKLAADDFRMKYESELAIRQGAENDTAGLLKVI
DDLTLTKADLESQVEGMTEELAFLKKNHEEEVDRLR-KQL---GGTVNVEVD-AAPSIDL
AKIMENMRQQYESMAQKNRQEAKDRFDRQTEELNQVVAVNTEQLNSKKTEITDLKRTFQS
LEIELQSQLSMKKALEDTLAETEARYGSLLAQIQAAIGNVEGQLVQVRADMERQNNEYNI
LLDVKTRLEMEIATYRRLL-EGE
>Alligator_K23
EKQMMQNLNDRLAAYLDKVRSLEAANAQLESCIVEWHKKRSH-----GKR-YDFNQF--E
QS-IADMQGQIQDGKISNASVLLQIDNAKLASEEFRLKYEAEKYQREGVQFDVENLRKEL
DNLTIVTTDLEMEIEGLREEHILRKKDHEADMHEQR-STK---DVNVNVKVK-ATPPEDL
TKILAGIREDYEAIIEKNRRGLDVWYKQQTSAASLAVTNSPEQLQNNDKEIKNLKRNFQA
LEIELQAQLSKKHALENTLSETRAHYASRLQNIQEFISKCEEELSELRHDVRCQSNKYKV
LLGIKTRLEREISTYQLLL-EGR
>Alligator_K36L1
GKETMQLLNDRLASYLEKVKSLEKENTDLERKIREWQECQTP-----LVC-TDFSSC--Y
ST-TEELQQQICSARAENARLRLDIDIVKVASDDFSFKYEYECGLCQSAEADISSLHRFL
DDLTLCKTDLETELESLTEELLCLRKNHEEEASPLR-HQL---GDCINVELD-TAPSCDL
NKVLEEIRCQYETLIEKNQCEVEDWYHTQMEELNKEVISDEEQLQGCQNEIIQLKHTVQA
LEIDLQTQHSLNTALESTLTDTEARYSHQLSELQCLISNMEAQLAELRSNIQHQNSEYKV
LLDVKSQLDCEISAYRRLL-DEE
>Alligator_K36L2
EKETMQFLNDRLACYLEKVRFLEQENADLEYKIREWYECQTP-----YVY-TDFNCY--Y
KM-IEELQQQICCAKADNARLCLDVDNLKVASDDYATKYEYEHGLCQSAEADVKSLRRIL
DDLTLCKADLETELESLTEELLCLRKNHEEEASPLR-RQL---GDRINVELD-AAPSCDL
NKVLEEIRYQYETLVDKNQCEVEDWYHTQMEELNKEVISSGEQLQGYQNEIIQLKHTVQA
LEIDLQTQHNLNTALENTLAETETHYSQQLSELQCLVSDVEVQLAELRSDIEHQDGEYKV
LLDAKCQLDCEISVYHSLL-DVE
>Alligator_K36L3
EKETMQFLNDRLACYLEKVRHLEQENADLECKIREWYECQVP-----YVC-TDFQAF--Y
KT-LEELQQQILCAKTNNAKMVLQIDNARLAADDFRTKFESEQVIRQSVDADINGLRRIM
DEFTLCRADLEMQLESLKEELLCLKKNHEEEASVLR-CQL---GARVNVEVD-AAPSCDL
NKVLNEIRCQYENLVENNRKDVEAWFSTKMEELNQQVISSGEQLQCCQTEITDLRRTIQA
LEIDLEAQTSMKSTLECTLHETENRYASQLAHLQCLITNVEAQLAELRCDIERQNQEYMV
LLDVKTRLETEIATYRRLL-EGE
>Alligator_K15
EKITMQNLNDRLASYLDKVRALEEANADLEVKIRDWYQKQGPT----SPA-RDYSPY--Y
KT-IEDLRDKILAATIDNSRIILEIDNARLAADDFRMKYENELYLRQSVEADINGLRRVL
DELTLARADLEMQIENLKEELAYLKKNHEEEMREYS-NQM---TGKVSVEMD-AAPGVDL
TKVLAEMREQYEALADKNRREAEAWFFTKTEELNREVATSTEQIQTSKSEMTELRRTLQG
LEIELQSQLSMKAGLEANLADTESRYCAQLAQLQAMITSIEEQLAELRCDMERQNQEYKM
LLDIKTRLEQEIATYRRLL-DGQ
>Alligator_K19
EKITMQNLNDRLASYLDKVRALEEANSELEIKIRDWYQKQGP-----GPS-RDYSPY--Y
KT-IEDLRDKILAATIDNSKIVLQIDNARLAADDFKTKFETEQGLRLSVEADINGLRRLL
DELTLARTDLEMQIENLKEELAYLKKNHEEEMNAHA-GQA---GGQVSVEVD-SAPGIDL
TKILAEMRDQYEFMAEKNRKDAEGWFNSKTEELNQEVAVNTEQLQTSRTEITDLRRTLQG
LEIELQSQLSMKAALEGTLADTENRYGAQLSQIQCLISNIEAQLGDLRAEMERQNSEYKM
LMDIKTRLEQEIATYHQLL-EGQ
>Alligator_K9L1
EKTTMQNLNDRLATYLDKVRRLEEENADLEERIREWYRKQGP-----SIS-KDYSPY--Y
QT-IEQLQNQIISATVDNNKLILDIDNSKMIADDFRMKYENELAIRQGVEADINGLRNFL
DELVRVRSTLELEVETLKDELLTLKKNHEEEMRLLQ-SQT---GGDVNVEVN-AAPGQDL
TKILNDMRQEYEEIIAKNRREVEQWYEVKMEEVNQQVTSSGQEVQTSSHQLTELRRVFQS
LEIELQTQLSMKASLENNLAETEARYGAQLQQIQAMINCVEEELAQLRCEMESQNQEYKM
LLGIKTRLEQEIAQYHGLLQEGQ
>Alligator_K9L2
EKMTMQNLNERLASYLDKVRHLEEENANLERLIREWYQNHGP-----QCG-RDYSQY--Y
GP-IEDLQNQIISATVDLNKVLLDIDNTRMTADDFRIKYETEYSLRQNVEGDINGLRPIL
DQLTLARSDLEAQYESLKEELISLKKNHEEEMRGLQ-SQS---GGDVNVEVN-TTPDSNL
MQKLNEMRCEYEQIIENNRKEVERWYETKMEEVNQEVHTSGQEIQSSNHQVSELRRDYQT
LEIELQSQLSMVQSLQSNLEDTERRYNIQLQQIQNTITTVEEELANIRCEIESQNYEYKM
LLDIKCHLEQEIAQYRALLEEGQ
>Alligator_K9L3
EKMTMQNLNERLASYLDKVRHLEEENANLERLIREWYQNHGP-----QCG-RDYSQY--Y
GP-IEDLQNQIISATVDLNKVLLDIDNTRMTADDFRIKYETEYSLRQNVEGDINGLRPIL
DQLTLARSDLEAQYESLKEELISLKKNHEEEMRGLQ-SQS---GGDVNVEVN-TTPDSNL
MQKLNEMRCEYEQIIENNRKEVERWYETKMEEVNQEVHTSGQEIQSSNHQVSELRRDYQT
LEIELQSQLSMVQSLQSNLEDTERRYNIQLQQIQNTITTVEEELANIRCEIESQNYEYKM
LLDIKCHLEQEIAQYRALLEEGQ
>Alligator_K9L4
EKMTMQNLNERLASYLDKVRRLEEENANLEQLIREWYQKQGP-----QGG-RDYSQY--Y
KT-IEDLQNQIISATVDLNKVLLDLDNTRMTVDDFRLKYETEYGLHQNVESDINGLRPML
DQLTLARSDLEAQYESLKEELISLKKNHEEEMKGLQ-SQS---SGDVSVEVN-AAPGTNL
TEKLNEMRGEYERLIENNRREVESWFEAKMEEVNQQVHSSSQEMESSNQQVSELRREYQT
LEIELQSLLSMLQSLQSNLEDTERRYNMQLQQIQAMIIPVEEELANIRCEMESQNEEYKM
LLGIKTRLEQEIAQYRALLEEGQ
>Alligator_K9L5
EKMTMQNLNERLASYLDKVRCLEEDNLQLERLIREWYQTHGP-----QGG-KDYSHY--Y
GP-IEDLLNQIVLATVANNKIILDLDNARMTAEDFRMKYETEYSLRQSVEGDINGLRPVL
DNLTLAKSDLEMQFESLREELIQLKKNHQEEMSSLQ-SES---GGDVSVEVN-AAPGEDL
MGKLNEMRKEYEYIIQRNREEVERWYESKMEEVRHEVHTSGQEIESGNQQVSELRREYQR
LEIELQSQLSTLQSLQSNLEDTDYRYNMQLQQIQNMIIPVEEELASIRCEMESQNEEYKM
LLGIKTRLEQEICQYRELL-DEG
>Alligator_HAS1
EKITMQNLNDRLASYLDKVRCLEEENAELECRIREWYAKHGPT----CEP-KDYSCY--L
RE-IEDLQNQIVCATIENNKIVLNIDNSRMAADDFRVKYETELSLLQSVEADINGLRQVL
DQLTLCRSDLEAQLEALKEELCCLKKNHEEEMNCLR-KQS---TGDVSVEVN-TCPGPDL
KQVLDEMRCQYEAIIAQNRKEVEDWYACKIEEVNREVISSGQEIECSNSQVCELRRQLQA
LEIDLQAQIRMRDNLQSSLAETECRYNNQLAELQNQIICVEQQLAELRAEIECQNQEYKV
LLDVKCRLEQEIHTYCCLL-EGG
>Alligator_HAS2
EKITMQNLNDRLASYLDKVRCLEEENAELECRIRDWYAKHGPT----CEP-KDYSCY--I
```

```
RE-IEDLQNQIVCATVDNNKIILDIDNSRMTGDDFRMKYETELALCQSVEADINGLRQVL
DQLTLCRSDLEAQLEALKEELCCLKKNHEEEINCLR-NQS---TGDVNVEVN-SCPGPDL
KQILDDMRCQYEAIIAQNRKEVEDWYACKIEEVNQEVISSSKEIECSNSQVSELRRELQA
LEIDLDSQLRMRDNLQSSLAETERRYNNQLAELQNQIICVEQQLAELRAEIECQNQEYKV
LLDVKCRLEQEIQTYCCLL-EGG
>Alligator_K14L1
EKETMQNLNDRLAAYLDKVRALEEANSELEIKIRDWYKKQGP-----GPD-RDYSPY--Y
RT-IEDLRNKILAATVDNANLILQIDNARLAADDFRTKFETEQALRMSVEADINGLRRVL
DELTLARADLEMQIENLKEELAYLKKNHEEEMNALR-GQV---GGEITVEMD-AAPGVDL
TKILTEMREQYEHLADKNRREAEQWFFKQTEELNQEVAINTEQLQSSKSEITELRRTIQG
LEIELQSQLSTKAALEGTLADTEARYGSQLAQIQGLITSVEEQLAELRCDMERQNHEYKI
LLDVKCRLEQEIATYRRLL-EGE
>Alligator_K14L2
EKETMQNLNDRLAAYLDKVRALEEANSELEIKIRDWYKKQGP-----GPD-RDYSPY--Y
RT-IEDLRNKILSATVDNANLLLQIDNARLTADDFRTKFETEQALRMSVEGDINGLRRVL
DELTLAKADLEMQLENLSEELAYLKKNHEEEMNALR-SQV---GGEITVEMD-AAPGVDL
TKVLAEMREQYEHLADKNRREAEQWFFKQTEELNKEVAINTEQLQSGKSEIMELRRTIQG
LEIDLQSQLSMKAALEGTLADTEARYGSQLAQIQGLITSVEEQLAELRCDMERQNHEYKI
LLDVKCRLEQEIATYRRLL-EGE
>Alligator_K117
EKDTMQNLNDRLAAYLERVCSLEEANGQLEQRIQELQVKK-------TLA-RHYDPSSCF
ST-IAELRSQIQDESVRNSQLTLQLDNAKLAANDFRMKYEAELATRLGVESDIKGLRKVL
DGLTEARASLQVQIESLKEELTYLQQNHGEEVAALQ-GHL---GGSVSVEVD-ASPGINL
VKTLAEIRDQYEDIIEKNQREAEAWHKQQCETFTQEVATSSEALQAARTKATELKRIVQS
LEIEFQMLQSTKEALEGTLAETESHYGVEMAQLQNLVAGKEAELAQLRTDTQRQANEYQH
LLDLKTRLEVEIATYQHLL-EGQ
>Alligator_K18
EKETMQDLNDRLASYLEKVRSLETANHNLEVQIREFLDKKGPST-------QDWTHY--W
DI-IEDLRNKIYEESVDNARIVLQIDNARLAADDFRVKYEGELAIRQSVEADIAGLRKVI
DDTNMSRLQLESEIENLKEELIYMKKNHEEEAKSLQ-AQV--SGSGLTVEVD-SPKTHDL
GKIMTDIRAQYEALAQKNREDLDKYWNQQITESTIEITQSTKDMDTAHGTLTELRRTYQT
LEIELESLRNLKGSLEANLMEVENRYGMQLEHLNGLLLRTEAELMQVRTEVQRQAEEYQA
LLNIKVKLEAEIATYRRLLEGGE
>Turtle_K24
EKGTMQNLNDRLAKYLDKVRALEEANADLEHKIRQWYEKHDPAAA--GLR-YDYSKY--Y
QI-IEDLKNQIISATIDNAKLVLQIDNAKLAVDDFRLKYENELFLHQSIEADINGLRRVL
DDLTMTRSNLEAQLESLTEELVYLKKNHEGEMKSFQHGST----GEVNVEIN-ATPGVDL
TKLLNEMRAQYEYLAEQNRREAEEQFNKMSQQLQQQISDDAGAADSARHEITEIKRTLQT
LDIELQSLLAKKCSLEGTLAEIEGNYGCQLSQIQLQISNLEEQLKQIRSEMECQNAEYEQ
LLGIKTRLEMEIETYRRLL-NGE
>Turtle_K12
EKETMQNLNDRLAAYLNKVHSLEEGNTDLEHKIREWYEKNGPGAGGPGAG-HDYSKY--Y
PI-IEDLRNKIINASIDNARIVLQIDNARLAADDFRLKYENEAALRQSVEADINGLRRVL
DELTLTRADLEMQIESLNEELAYLKKNHEEELQGFQ-GNA---TGQVSVEMD-AAPGIDL
TKLLNDMRGQYEVIAEQNRKEAEAWFNEKSMELKREISTNTEQLQSGKSEITDLRRILQG
LEIELQSQLAMKKSLEDTLAETEASYCAQLSQIQLQIGNLESQLFQVRADMERQNAEYQQ
LLDIKTRLEMEIETYRRLL-DGE
>Turtle_K20
EKLTMQNLNDRLASYLEKVHFLEKANSQLEKQIREWYANSSS-----TTR-HDHSPY--F
KT-IEDLQNKIGAAHLENARLLLQIDNAKLASDDFRMKYENELAIKQNVENDLAGLLRVL
DDLTLTRSDLALQIEGLNEELTFLKKNHEEEVGELR-KQL---GGTVNVEVD-AAPSIDL
AKIMENMRQQYDSMAEKNRQEAKDRFDKQTEEVNQEVAINIEQLQTKKTEITDLRRTFQG
LEIELQSQLSTKKALEDTLAEMEARYGSQLAQIQAAIGNVEAQLMQLRADMQSQSTEYST
LLDIKTRLEMEIATYRRLL-EGE
>Turtle_K23
EKQTMQNLNDRLAAYLEKVRSLEAANAQLESCILEWHKKRSH-----EKK-HDFNQY--E
QN-ITDMQGQIEDGKITNASIFLHIDNAKIASEDLSLKYEVEQSLRQGMQISVENLRKEL
DNLTIVTTDLEMEIEGIQEEQILRKKDHEKDMGEHH-SSQ---DFKVNVQVN-AVPKEDL
AKILAGIRKDYEAIIEKNHQGLEVWYKEQTATVSPATNISPEEIQSNESEIKNLRRTFQA
LEIELQAQFSKKYALKGTLAETQAHYTFQLQNIQQSISNCEEELSQLRQDIKCQNNHYKI
LLGIKTRLEKEISTYRQLL-EGT
>Turtle_K15
EKVTMQNLNDRLASYLDKVRALEQANTDLEIKIRDWYQKQGPT----SPD-RDYSPY--Y
KI-IDELRDKILAATIDNSRVILEIDNARLAADDFRLKYENELFLRQSVEADINGLRRVL
DELTLSRADLEMQIENLKEELAFLKKNHEEEMKEYS-NQM---GGKVSVEMD-AAPGVDL
TSILSEMREQYEILADKNRRDAEAWFYKQTEELNREVATSTEQIHTSKSEITELRRTLQG
LEIELQSQLSMKAGLEANLAETEGRYCAQLAQLQAMITSIEEQLAELRCDMERQNQEYRM
LLDIKTRLEQEIATYRRLL-EGQ
>Turtle_K19
EKSTMQNLNDRLASYLDKVRALEEANSELEVKIRDWYQKQGP-----GPA-HDYSLY--Y
KT-IEDLRDKILAATIDNSKIVLQIDNARLAADDFKTKYETEQALRMSVEADISGLRRVM
DELTLARTDLEMQIESLKEELAYLKKNHEEEMKALG-GQT---GGQVSVEVD-SAPGIDL
TKILADMRDQYEVMAEKNRKDAETWFNSKTEELNQEVAINTEQLQTSKTEITDLRRTLQG
LEIELQSQLSMKAALEGTLADTENRYGAQLAQIQHLIGNVEAQLTDLRADMERQNSDYKQ
LMDIKTRLEQEIATYRQLL-EGQ
>Turtle_K9L
EKITMQNLNDRLASYLNKVRALEEENANLENLIKEWYLRKGPI----ATL-KDYSRY--Y
EE-IEQLNNQIVNSSVEVNKVILNIDNTRMTIEDFKLKYETELGLRQNVEGDINGLRPLL
DQLTLTRSDLELEFESLKEELISLKKNHEEELKGLQ-PQT---SGDVNVEVN-ATPGYDL
TEILNDLRREYEHIIENNRKEVEKWYETKISEVNHEVHSSGRDIESSTKQVTELRREYQN
VEIELQSLLSTIQSLQSSLADTEGRYNIQLNQIQTIIVSVEEELASIRCEIDNQNQEYRL
LLGIKNQLEQEIAQYRHLLDEGN
>Turtle_HAS1
EKLTMQNLNDRLASYLNKVRCLEEENAALECKIKEWYAHHGHT----GVP-KDYSCY--Y
KE-IEDLQNQIVCATLDNNKIILNIDNSRMAADDFRLKFETELALRQSVEADINGLRHVL
DELTLCRSDLEAQLECLKEELCCLKKNHEEEINCLR-NQS---TGDVSVEVN-SCPGPDL
KKILEEMRCQYETMIAQNRKEVEDWYECKIEEVNREVITSSQEVESCNNQVAELRRQLQC
LEIDLQSHLSQRDNLEASLAETESRYNSHLCQLQQQITCVEQQLADLRTEIESQNHEYKV
LLDVKCRLEQEIHTYRCLL-EGG
>Turtle_HAS2
-----------------------------------------------XXX-KDYSCY--Y
KE-IEDLQNQIVCATLDNNKIILNIDNSRMAADDFRLKFETELALRQSVEADINGLRQVL
DELTLCRSDLEAQLECLKEELCCLKKNHEEEINCLR-NQS---TGDVSVEVN-SCPGPDL
KKILEEMRCQYETMIAQNRKEVEDWYECKIEEVNREVITSSQEVESCNSQVAELRRQLQC
LEIDLQSHLSQRDNLEASLAETESRYNSHLCQLQQQITCVEQQLADLRAEIESQNHEYKV
LLDVKCRLEQEIHTYRCLL-EGG
>Turtle_K14L1
EKETMQNLNDRLATYLDKVRALEEANSDLEIKIKEWYKKQGP-----SPD-RDYSPY--Y
RT-IEDLRSKILAATIDNANIVLQIDNARLAADDFRTKFETELALRMSVEADINGLRRVL
DELTLARADLEMQIENLKEELAYLKKNHEEEMNILR-SQL---GGEITVEMD-AAPGVDL
TKILADMREQYESLAEKNRKEAEQWFFTKTEELNREVAMNTEQLQSGKSEITELRRTVQG
LEIELQSQLSMKAALEGTLAETESRYGAQLAQLQGLITSVEEQLAELRCDMERQNHEYKI
LVDVKTRLEQEIATYRRLL-EGE
>Turtle_K14L2
```

```
EKETMQNLNDRLANYLDKVRALEEANSDLEIKIKEWYKKQGP-----GPD-RDYSPY--Y
RT-IEDLRSKILAATVDNASLLLQIDNARLTADDFRTKFETEQALRMSVEADINGLRRVL
DELTLARADLEMQIENLKEELAYLKKNHEEEMNILR-SQL---GGDITVEMD-AAPGVDL
TKILADMREQYESLAEKNRKEAEQWFFTQTEQLNREVAMNTEQLQSGKSEITELRRTVQG
LEIELQSQLSMKAALEGSLAETEARYGSQLAQLQGLITSVEEQLAELRCDMERQNHEYKI
LVDVKTRLEQEIATYRRLL-EGE
>Turtle_K117
EKDTMQNLNDRLAAYLETVRSLEEANGQLERQIREVYAKRAQ-----AGC-QDLSGY--F
ST-IAELRSQIQAASTSNAQLILQIDNATLAADDFRVKFESELAIRRGGESDVAGLRKVL
DEVTLSRASLQEELGSLQEELAQLQKNHGEEAAALQ-GRL---GGSVSVEVD-SAPGVDL
VKTLAEIRDQYEDVIERNHREAAAWHKEQCETVAQEVATSAEAVQGARTKLTELRRVVQG
LEVELQSLYSMKEALEGTLAETRAGSGAELEQLRECVARKEAELAQLRTDTQRQAEEHQQ
LLDLKTRLEMEIATYRRLL-EGD
>Turtle_K18
EKETMQDLNERLASYLDKVRRLETDNQKLETQIREFMDKKG------PSS-RDWSHY--F
DI-IEDLRKQILDSAVDNARIVLQIDNARLAADDFRVKFESELAIRQSVESDINGLRKVL
DDTNMSRLQLEGEIESLKEELIFMKKNHQDEVDSLQ-AQI--TNSGLTVEVD-AAQPQDL
GKVMAEIRAQYDNLAQKNMADLDQQWNQMITQSTVEITQNTKEIEVARSSVSELRRTYQT
LEIELESLRNLKASLEANLQDVEQRYAMQMEHLNGQLLRAEAELAQIRNDIQRQSEDYQA
LLNIKDKLEAEIATYRRLL-EGG
>Lizard_K24
EKETMQNLNNRLANYLEKVRSLEEANAELEVKIRHWYEKNGPGAP--GVV-RDYTKY--H
QI-IEDLRNKIVNTTIDNANVVLQIDNARLAADDFRLKFENELFLRQSVESDINGLRRVL
DELTMNRSDLEAQLENLTEELAYLKKNHEEEINMLK-SNS---TGDVTVEMN-AAPGIDL
TKLLNDMRAQYEDLAEQNRREAEEQFHKMSQPLQQQIYDDAGAVSSARNELIELKRSFQS
LEIELQSLLAKKASLEGTLTETEANYSSQLHQLQLQVSNLEEQLQQIRSETECQNSEYQQ
LLGIKTRLEMEIETYRRLL-DGE
>Lizard_K24L
EKHTMQNLNDRLANYLGKVHALEEANAELELKIKDWYAKCSIP----DTS-RDYTKY--F
NI-IEDLRGKILRQTIENAGIVLQIDNARLAADDFKLKYENELFLHQNVENDINGLRRVL
DDLTMHRSDLELQIEGLNEELAYLKKNHEEELKSFR-GAS---SGDVSVEMD-AAPGVDL
TKLLNSMRSQYEELAEKNRQEAEDQFNKQVGALRQEISANVDQLSTGKTEITDLRRTLQG
LELELQAQLAMKQSLEGTLAETERNYCDQLAQIQSQIAGLEEQLLQLRADMENQNSDYQH
LLGIKTRLEQEIETYRRLL-DGE
>Lizard_K10L
EKETMQNLNDRLASYLAKVHALEKANSELELKIKNWYEKNAFQA---DGGFRDYSKY--Y
AS-IDELRNKIVSASVNNSNIILQIDNARLAADDFRMKYENEVFFRQSIEADINGLHKVF
DEVILSKSNLELQIESLIEELAFLKKNHDEEIRGLR-GPT---SGNVSVEMN-AVPGPDL
TILLNKMREEYEALAEQNRQDAETWFVKKSNELNTTISMSAKETSTNKKEIAELRRTLQG
LEIDLKTQLALRQSLETTLAETEDGYCSQLLQMQGLINNTEAQVKQVRDDMESQNADYRQ
LLDIKIHLENEIETYRHLI-DSE
>Lizard_K12
EKETMQNLNDRLAAYLDRVHSLEEANTELERKIREWYEKNGPGTGALGAG-RDYSKY--Y
PV-IEDLRNKIINATIENARIVLQIDNARLAADDFRLKFENEVALRQSVEADINGLRRVL
DELTLTRADLEMQIETLNEELAYLKKNHEEELIGFQ-SNA---TGQVSVEMD-AAPGVDL
TKLLNEMRAQYESIAEQHRKEAEAWFNEKSGELKREISTHSEQLQYGKSELTDLKRTLQS
LEIELQSQLALKKSLEDTLAETEAGYCTQLAQMQLQIGNVESQLFQVRADMERQNAEYQN
LMDIKTRLEMEIETYRRLL-DGE
>Lizard_K23
EKQTMQDLNDRLAAYLDKVHSLEEANTQLEGCIREWHERRSQ-----GMK-HDFTDY--E
QN-IVDMHERIEAGRVTNAGIVLQIDNAKMATEDFRLKYESEKALRQSVQDDVENIRKEL
DNMTIIITDLEMEIEALREDHILKKKEHEKDMEAS--GAF--KDVNVNVKVN-TAPQEDL
AKILAGIRADYEAIIQKNHQSLDAWFQKQIANDSSEEKPNQEEQQSNQKEISELNRTLQS
LEIDLQTHLNKKCSLENNLDETRAYYAFQLQSIQNLISQCEEELSEFRHDIKCQNNQYKV
LLGIKTRLETEISTYRQLL-DGK
>Lizard_K36L1
EKETMEFLNNRLGNYLERVRCLEQENAELEAKIREWYEHENA-----YVC-TDFKPF--Y
CT-IDELQQQICCTKADNAKLCLDIDNLKMASDDYCTKYDYELSLRQRDECDSSGLRKVL
DNLTLCRADLEAQLESLTEEMMCLKKNHEEESSALR-SQL---GDRINVEVD-AAPSCNL
NKVLDEMRCQYEADLEKNRREVEDWYCAQMEELNKEVISSGEQLQCCQTEIIETRRCVQT
LEIELQAQQSMKSALETTLQETESRYCTQLAQLQCLISNVEAQLTEIRSDIERQNCDYKA
LLDEKARLDCEIAAYRNML-EGE
>Lizard_K36L2
EKELMQCLNDRLANYLERVRCLEQDNANLECKIREWHECQAP-----YES-TDFHSH--Y
KI-IEELQHQILCAKTENARLVLEIDNARLAADDFRTKYETELALRQCVESDIHGLRKIL
DEMTLCRADLEAQLESHKEELLCLKKNHEEEANALR-SKL---GARVNVEVD-AAPSCDL
NKMLGEIRNQYECLAEKNRRDVETWFSSKMEELNQQVLSSDEELQNCQSEIIELRHTMQA
LEIDLDAQQNMKNALECTLHETEARYSTQLAQLQCMISTIEGQLGDLRNDMERQSHEYKI
LLDVKTRLEAEISTYRRLL-EGE
>Lizard_K15
EKVTMQNLNDRLATYLDKVRALEEANTELEVKIRDWHQKQAPK----SPS-RDYSQY--Y
KI-IEDLRDKILGTTIDNSRVILEIDNARLAADDFRLKYENELHLRQSVEADINGLRRVL
DELTMSKSDLEMQIEGLKEDLAYLNKNHEELMKEFS-TQL---TGKVSVEMD-AAPGIDL
TKILADMREQYEHLAEQNRKDAETWFFSKTEELNREVATNTEQIQTSKSEISELRRTIQG
LEIELQSQLSMKAGLESNLAETEGRYCAQLAQIQNLITGVEEQLAELRNDMERQNQEYKM
LMDIKTRLEQEIATYRNLL-EGQ
>Lizard_K19
EKITMQNLNDRLASYLDKVHALEESNADLEVKIREWYQKQGPT----SPA-RDYSHY--Y
KT-IEDLRDKILDATIDNNKIVLQIDNARLAADDFKTKFESEQALRMSVEGDINGLRRVL
DELTLARSDLELQIETLKEELAYLKKNHEEELGALS-GHL---GGQVSVEVD-SAPGIDL
TKILAEMRDQYELLADKNRRDAEAWFTTKTEELSREVAVNTEQLQSSKTEITDLRRTLQG
LEIELQSQLSMKAALEGTLADTENRYSAQLCQIQNLISNIEAQLVEVRSDMERQNSDYKM
LMDIKSRLEQEIQTYRQLL-DGH
>Lizard_K9L1
EKSTMQNLNDRLAAYMNKVKYLEDENAQLEVLIREWYQKHGDV----GTV-RDYTHH--Y
QE-IDQLFNELVNETDDYNKILLNIDNTRMTAEDFQIKYQTEAGLRQNVEADINGLRPML
DQLTLARSDLEAQFESAKEELITLKKNHQEALRELQ-SQR--GHGDINVEVN-AAPGPDV
KQRLDQLRAEYEAIIDNNRREVEQWFESKMEEVRQQVSSSGLEISSGNQQVTDLRRQYQT
LEIELQSALSMIQSLQSNLEDTERRYNLQLQQIQSMIQPVEGELASLRGEIESQSQEYQS
LLGIKTRLEQEIAQYRQLLDEGQ
>Lizard_K9L2
EKSTMQNLNDRFASYLNKVKSLEAENNQLETLIREWYQKHGQT----SEP-KDYSSY--Y
TE-IDQLVNELISASLESNKILLDVDNARMAAEDFKLKYETEYGLRQNVDADLQGLRPLL
DKLTMDKSDLEMQYESLHEELVHLKKNHDEVMKSQH-HQS---GGDVNVEVN-SSPGQDL
TKTLNDLRQEYEEIIMKNRKEVQQWFEAKMEEARQQQSPESQEGGSGRHQITELTREYQT
LEIELQTQLSTIQALQNNLNNTEGGYNMQLQQLASQIEPVEAELAGIKGEIQNQTQEYQT
LLGIKTYLEQEINQYRQLLEEGK
>Lizard_HAS1
KKMTMQNLNDRLASYLDNVRYLEEENAQLECKIRDWYDQQGSP----NDP-KDYNYY--Y
EQ-TEDLKKQLICATVDCRKLILDIHNNKLTADDFRMRYEAERVFRQDKETDIDSLRQVL
DQLTLGRSDLEAQLESFQEELCCLKKNHEEKVKLLT-KQA---QGNLNVEVN-SNPSSEL
QKALDNLRRRYEAIIEKNVKEIETWYESMTEAINMS-----KELKDDNNQVVDLKGQLQT
LESNLQTQLSLRDTLQDSLAETQCLYNNSLAEIQNQICYMEQQLVELRVKTECLDQEYIR
LSDAKCLLEHEIETYCCLL--GE
```

```
>Lizard_HAS2
EKMTMQNLNDRLASYLDKVRCLEEENGQLECKIKEWYDRQGPV----NES-KDYAAY--Y
QQ-IEDLKNQIVCATVENSKLVLDIDNNRMTADDFRMKYETELALCQNVEADINGLRQVL
DQLTLCRSDLEAQLESLREELCCLKKNHEEESKHLK-KQS---SGDVSVEVN-TCPGPDL
KKVLDELRCQYENMIENNRREVEKWYESKMEEVNLEVCNSSKEAEDCNNQIVDLKRQLQT
LEIDLQAQLSLRDTLQDSLAETECRYNGHLAEIQNQISCVEQQLGELRAEMECQNREYRE
LLDVKCRLEQEIQTYRCLL-EGG
>Lizard_HAS3
EKVLMQNLNDRLGNYIDKVRCLEKENAALESQISDFCLKHGLT----GEM-KDHSHF--Y
QQ-IEELKKQVFVANLENNKILLKIDNNHMDLEDLRKKYETEYVIRENVEADVNGLRPVL
KELTGYKADLKAELNSLKEEICDLKKTHEKDLNCRK-KRS--HGSNKEVSIG---PGTDL
KQMLKEMRQKYEAKIESNRKEVVQRYEDKLKEMNLNVNNSSQDIEDGNHKVLDLKCKLPA
LEIDLQAQYNMRDTLKVCLAEIECHFQTQLGEMQEQISNMEHQLAELHLEMETQDQEYKG
LVDVKKRLEQEIQTYSSLLKEGQ
>Lizard_HAS4
GKMTMQNLNDRLASYLDKVRCLEEENADLESRIREFYAKQGPL----SEP-KDYSHY--H
QQ-IEDLKNQLICTSVENNKLLLCIDNSKLTADDFRTKFETECCLRQNVEADINGLHQIL
DQLTACRADLDVQCDNLQDELNCLKKNHAEEVACLK-KQS---TGDVNVEVN-TCPGPDL
KKILEEMRCKYETMIEGNRKEVEAWYESKIEEVNRDVCNSSQEIEESNNKVTELRRQLQA
LQIDYEAQCSLRDTLEASLGETELRYNGHLGDLQERISCLEQQLAELRSEMECQNHEYTE
LLDVKSRLEQEIGTYRGLL-EGG
>Lizard_K14L1
EKETMQNLNDRLASYLDKVRALEEANTDLEVKIKEWYKKQGP-----SPE-RDYSPY--F
RT-IEELRSKILAATVDNASIVLQIDNARLAADDFRNKFETEHALRVNVEADINGLRRVL
DELTLARADLEMQIENFKEELVYLRKNHEEEMSIFR-SQS---GGEISVEMD-AAPGVDL
TKILSEMREQYESLAEKNRKDAEQWFFSKTEELTREVAINTEQLQSGKTEITELRRTYQN
LEIELNAQLSMKAALESSLADTEARYGAQLSQIQVLITNVEEQLADLRCEMERQNHEYKM
LLDVKTRLEQEIATYRRLL-EGE
>Lizard_K14L2
EKETMQNLNDRLATYLDKVRALEHANTELEVKIREWYQKRGP-----GPQ-RDYSSY--F
RT-IEDLRSKILAASVQNATILLQLDNAKLTADDFRTKYETEHALRTSVEADIHGLRKVM
DELTLTRSDLEMQLENLKEELAYLHKNHEEELKVLR-SQM---GGEITVEMD-AAPGVDL
TKILAEMREQYEHLAEKNRKDAEQWFFSKTEELNQEVAINTEQLQSGKSEVIELRRTLQG
LEIELQTQLSMKSSLEGTLAETESRYGLQLNQLQALITSVEEQLAELRCDMERQNSEYKI
LLDVKTRLEQEISTYRRLL-EGE
>Lizard_K117
QKGTMQNLNDRLAAYLGRVHSLEESNAQLEQNIREYYAKRAS-----AGG-PDLSGY--F
NT-LSELKAKIEQETLNNAGLLLRIDNAKLAADDFKVKLESEVAIRLSLEGDVNGLRKVL
DDVNASRASLQVQVDNLQEELVYLKRNHEEEVASLQ-GRL---GGTVNVEVD-SMPGVDL
QKVLAEIRDQYEDVMEKNRQEAEALHKAQCDAINQQVADSSEALQAAQLKITELRRLAQA
LEIELQSLRSTKIALEGTLVETESRYGMELSRLRELISARESELFQLRSEAQRQAEDYKR
LMDIKNKLEQEIATYRRLL-EGL
>Lizard_K18
EKETMQDLNDRLASYLEKVRSLEADNRKLEIQIREHLDKKGPSA-------RDWSHY--F
EL-IEDLRNQIFDQTIDNSRIVLQIDNARLAADDFRVKFEAELAIRQSVENDINGLRRVI
DDTNMMRLQLESEIEALKEELVFMKKNHQDEVNNLQ-AQI--ANSGLTVEVD-APKTQDL
GKIMAEIRAQYDVLAQKNLEDLDKYWSQQITESTVVITEHSKDIEKARANITDLRRSVQT
HEIELESARNLRASLEANLHEVEMRYGLQMEHLNGIILRTEADLAQLRNDVQRQAEEYQI
LLNIKDKLEAEITTYRHLLEGGE
>Snake_K24
EKETMQNLNSRLANYLEKVRSLEEANAELERKIRHWYEKNAPGAP--GVT-RDYSKY--H
RI-IDELRNQILSMTVENANVVLQIDNARLAADDFRMKFENELFLRQSVEGDINGLRRVL
DDLTMNRSDLEAQLESFTEELAYLKKNHEEEMTLLR-SSS---TGDVTVEMN-AAPGIDL
TKLLNDMRAQYEDLAEQNRREAEEQFIRMSQPLQQQIHDDAGAASSARNELIELKRSLQS
LEIELQSLLAKKASLEGTLSETEASYSTQLSQLQMQVSSLEEQLQQIRAETECQNSEYQQ
LLGIKTRLEMEIETYRRLL-DGE
>Snake_K24L
EKQTMQNLNDRLANYLGKVHALEEANSELELRIKEWYGKFSAT----DTD-RDYSKY--F
KI-IEDLRNQICRYTIENAGIVLQVDNARLAADDFKLKYENELFLHQSVENDINGLRRVL
DDLALNKADLELQVESLTEELVYLKKNHEEELMSFR-GVA---SGDVTVEMD-AAPGVDL
TKLLNDMRSQYEELADKNRREAEEQFNKQVGALRQQISANVDQLSSGKSEITELRRTMQG
LELELQAQMAMKQSLEGTLAETERNYCDQLGQIQVQISSFEEQLLQIRSDMENQNAEYQQ
LLGIKTRLEKEIETYHRLL-DGE
>Snake_K10L1
EKETMQNLNDRLASYLAKVHALEEANGDLEQKIRNWYQKSATHIN--EGL-YNDSKY--N
SS-IEDLRNKIITASIRNAGTILQIDNARLAADDFRMKYENELFLHQNVEADINGLKKLL
DELSMSKSDLEIQIKSLTEELTFLKKNHEEEMKNWQ-GTV---AGDINVEIN-AAPGVDL
TAVLNKMRTEYEALAEQNRKDAETWFIEKSKELNTTINVSAKETTTNKKQIADLRHILQS
LEIDMKTQLSLRQSLERTLAETEGQYCAQLLKIQGKISSVENQVQQIREDMECQNADYRH
LLDIKIRLENEIETYRRLI-DGE
>Snake_K10L2
-XXXMQNLNDRLATYLGNVQALEEANSELEGKIAEWYEMFGAGNQA-GQQ-QDYSEY--Y
AI-IDDLRNQIIATSIDNGTLILQIDNARLAGADFKLKYDNEVAIHQSVEMDANGLRKAL
EEIEITSSDLKVQINSFTEELEQMKKNHEEDMVSMN-DST--AAGDINVEMN-VTPGVDL
TTLLNQMRGEYEALAEQNREDMEAWFHEQSQELNRQINTSVEETSTNKSEITEMKRALQG
LEIELQSQLSLKQSLEATLAETEGQYCAQLSQIQEVIGSVEGQVQQIRDDMECQNREYEQ
LLDVKIRLENEIDTYRSLL-DGS
>Snake_K20
-XXXMQNLNDRLAAYLEKVRSLETSNSVFEKQIREWYEKSSP-----ITK-QDYSAY--F
KV-IRELQDKITEAREDNTRIILQIDNSKLAADDFRVKYETELGLRLSVEYDITGLQGLI
DDLTLVRTDLEQQIESLKEELAYLRKQHDEETARLR-GQL---SGNVSVEVD-AAPGIDL
AQMMENMRQQYEILAEKNRQEAKDRFDKLIEQLNEEVTTTTYQLETHRSEITDRRNTLQS
LEIELRSQLSWKTERENALAEIEAHYRTILTQIQMAIGNIEAQLMQLRSDMEQQSMEYSI
LLDIKVKLEAEISTYRRLL-EGE
>Snake_K23
EKRTMQELNDRLASYLEKVRSLEEANTQLEGCIREWHQKRSH-----GTQ-RDFKDY--E
QN-ISDMHDRIETGRVTNAGLVLQIDNAKMATEDFSLKYEAEKALRQNVQNDVENIRKEL
DNMTIIITDLEMEIEALREDHILKKKEHEANMQAHG-SSK---DFKVNVKVD-KTPQEDL
SKILAGIRADYEAIIEKNRQSLDSWFQQQIGNESSAGQHNQEELQNSQKEISELNRTLQT
LEIDFQTELNKKFALENNFGETKGRYAFQLQSIQNLIKKCEEELSKFRHDIKCQNNQYKV
LFGIKTRLEREISTYRQLL-DGD
>Snake_K15
EKITMQNLNDRLANYLDKVRALEEANTELEIKIRDWHQKQAPT----SQA-KDYSNY--Y
KI-ISDLRNKIGDCTCDNSKVVLEIDNARLAADDFRLKYENELHLRQGVESDINGLRRVM
DELTLFKSDMEMQIEEKKEDLAYLKKNHEELMKEYS-TQL---TGQVNVEMD-AAPGVDL
TKILADMREQYEQMAERNRRDAEAWFFTKTEELNREVATHTEQLQTSKTEISELRRTIQS
LEIELQSHLSMKAGLESNLADTEGRYCAQLAQIQNLITGVEEQLAELRNDMERQNQEYRM
LMDIKTRLEQEIATYRNLL-EGQ
>Snake_K19
EKVTMQNLNDRLANYLDKVRALEEANTELEVKIREWYQKQGP-----TAS-RDFSLY--F
KT-IEDLREKIKDATIGNNSIILQIDNARLAADDFRTKFETEQALRISVETDINGLRRVL
DELTLSRADLEMQIENLKEELAYLKKNHEEEMSTLS-GQL---GGQVSVEVD-SAPGIDL
TKILADMRDQYELLAEKNRRDAEAWFTSKTEELNKEVAVNTEQLQTSKTEITDLRRTLQS
LEIELQSQLSMKSALEGTLADTEARYGNRLCQIQGLISNIEAQLVEVRSDMERQNNDYKM
```

```
LMDIKTRLEQEIETYRQLL-DGQ
>Snake_K9L
EKATMQNLNDRLANYLDQVKRLEDENCQLEIWIKEWYQKHGDI----GPD-KDYSCY--Y
QE-IDQLYNELIAETDDYNKIILNIDNTRMTAEDFKLKYNTEAGLCKNVEADINGLRPLL
DQLTLAKSDLEMQFESLREELICLKKNHEETLRGLR-GQR--GHGDVSVEVN-AAPGQDL
KQRLDQLRCEYENIIEQNRKEVEVWYETKMEEVRQQVSTSDQEITSSNHQLSELRREYQT
LEIELQSQISMIQSLQTNLEDTERRYNMQLQQIQAMIEPVEGELASIRCEIESQNQEYQL
LLGIKTRLEQEICQYRRLL-EEG
>Snake_HAS1
EKMTMQNLNDRLACYLEKVRNLEEENADLEARIRQWYDRQGPP----SEL-KDYSSY--Y
QQ-IEELKNQIVCATVDNSKLILDIDNNRMTADDFRLKYETELALCQNVEADINGLRQVL
DQLTLCRSDLEAQLESLREELCCIKKNHEEEIKSLK-SQT---CGDVSVEVN-SSPGPDL
KKVLEEMRCQYETMIENNRKEVEQWYERKMEEVNQEVCISSKEVEDCNNQVVDLKRQLQT
LEIDLQAQLCLRESLQDSLGETECRYNTHLAEIQNQISLVEQQLAELRAEMECQNREYRE
LLDVKCRLEQEIQTYRSLLEEGQ
>Snake_HAS2
GKMTMQNLNDRLASYLDKVRCLEEENAELECRIREFYAKQGPL----SEP-KDYSHY--H
QQ-IEDLKNQLICASVENNKLLLCIDNSKLTADDFRSKYETECCLRQNVEADINGLHQIL
DQLTACRSDLDIQCENLQDELCCLRKNHEEEVACLK-NQA---SGDVSVEVN-ACPGPDL
KKILEEMRCKYESMIEGNRKEVETWYESKIEEVNRDVCTSSQEIEESNNKVTELRRQLQA
LEIEYDAQCSLRDTLEGSLGETELRYNSHLAELQERISCLEQQLAELRSEMECQNHDYTE
LLDVKSRLEKEIATYRGLL-EGG
>Snake_K14L1
EKQTMQNLNDRLANYLDKVRALEEANTSLEIKINEWYQKQGP-----APD-RDYSSY--F
RK-IEDLKNQILAASIDNASVLLQIDNARLAADDFRTKFETEQALRLNVESDINGLRRVL
DELTLSRSDLEMQIENLKEELAYLKKNHEEEMNAFR-KQV---SGEITVEMD-ATPGVDL
TKILTDMREQYETLAEKNRKDAEQWFFTKTEDLNREVATNTVQLQTSKTEIVELRRTIQS
LEIDLQAQLSMKSALEGTLVETETRYGTQLGQIQGMITNVEEELAALRCDMERQNHEYKI
LLDVKTRLEQEIATYRRLL-EGE
>Snake_K14L2
EKETMQNLNDRLAAYLDKVRALEESNASLEVRIKEWYQKQAP-----GPD-RDYSYY--F
RI-IEDLRSKIVSATVENADVLLQIDNAKLTADDFKNKFEIEQALRMSIESDINGLRNVL
DDLTSTRIHLEKNLELLTEELLHLKKNHKEEMGLLQ-SQM---GGEITVEMD-AAPGVDL
TKILADMREQYENLAEKNRRDAEQWFFSKTEELNREVAINTEQLQSGQSEVIELRRTLQG
LEIDLQAQLSTKAALEGTLADIESRYSLQLAHIQGLIINVEEELATLRCDMERQNHEYKI
LLDVKTRLEQEIAMYRHLL-EGE
>Snake_K117
EKGTMKNLNDRLAAYLDKVLSLEKSNMQLEQNIQEFYTKQTS-----TGT-HDMSAY--F
NA-MIQMKSEIQKETLKNAGLLLQIDNARLAADDFRVKLESEVAMRLSLEGDLGGLRKAL
EELNASRASLQVQADNLQEELACLKRNHKEEVASLQ-GHL---GGTVNVEVD-SMPGTDL
QKVLGEVRDQYEGVMEKNRQEAEALHQSQCDALNQEVSVSTEALQSAQMKMVELKRLAQA
LEIELQSLWSMKEALEGTLAEVESHYGLELSQLQNLVAAREAELLQLKSDAQNQAEDYKR
LMDIKNRLEQEIATYRCLL-EGS
>Snake_K18
EKETMQDLNDRLANYLDKVRSLEADNRKLEIQIREYMEKKG------PSA-RDWGRH--F
QI-IDGLKNQIYDQSVDNARVVLQIDNARLAADDFRVKYEAELAIRQSVESDIFGLRKVI
DDTNMSRLQLESEIEALKEELIFMKKNHQDEVTTLQ-AQI--ANSGLTVEVD-APKSQDL
AKIMAEIRAQYDALAQKNLEDLDKYWSQQISESTTVINQNSKEIETARVTMTDLRRTVQT
LEIELESMRNVRASLEGNLLEVESRYAMQIEHLNGILLRTEAELSQLRNDVQRQADDYQA
LLNIKDKLEAEIATYRHLL-EGG
>Human_K9
EKSTMQELNSRLASYLDKVQALEEANNDLENKIQDWYDKKGPA----AIQ-KNYSPY--Y
NT-IDDLKDQIVDLTVGNNKTLLDIDNTRMTLDDFRIKFEMEQNLRQGVDADINGLRQVL
DNLTMEKSDLEMQYETLQEELMALKKNHKEEMSQLT-GQN---SGDVNVEIN-VAPGKDL
TKTLNDMRQEYEQLIAKNRKDIENQYETQITQIEHEVSSSGQEVQSSAKEVTQLRHGVQE
LEIELQSQLSKKAALEKSLEDTKNRYCGQLQMIQEQISNLEAQITDVRQEIECQNQEYSL
LLSIKMRLEKEIETYHNLL-EGG
>Human_K10
EKVTMQNLNDRLASYLDKVRALEESNYELEGKIKEWYEKHGNSHQ--GEP-RDYSKY--Y
KT-IDDLKNQILNLTTDNANILLQIDNARLAADDFRLKYENEVALRQSVEADINGLRRVL
DELTLTKADLEMQIESLTEELAYLKKNHEEEMKDLR--NV--STGDVNVEMN-AAPGVDL
TQLLNNMRSQYEQLAEQNRKDAEAWFNEKSKELTTEIDNNIEQISSYKSEITELRRNVQA
LEIELQSQLALKQSLEASLAETEGRYCVQLSQIQAQISALEEQLQQIRAETECQNTEYQQ
LLDIKIRLENEIQTYRSLL-EGE
>Human_K12
EKETMQNLNDRLASYLDKVRALEEANTELENKIREWYETRGTGTAD-ASQ-SDYSKY--Y
PL-IEDLRNKIISASIGNAQLLLQIDNARLAAEDFRMKYENELALRQGVEADINGLRRVL
DELTLTRTDLEMQIESLNEELAYMKKNHEDELQSFR-VGG---PGEVSVEMD-AAPGVDL
TRLLNDMRAQYETIAEQNRKDAEAWFIEKSGELRKEISTNTEQLQSSKSEVTDLRRAFQN
LEIELQSQLAMKKSLEDSLAEAEGDYCAQLSQVQQLISNLEAQLLQVRADAERQNVDHQR
LLNVKARLELEIETYRRLL-DGE
>Human_K13
EKITMQNLNDRLASYLEKVRALEEANADLEVKIRDWHLKQSPA----SPE-RDYSPY--Y
KT-IEELRDKILTATIENNRVILEIDNARLAADDFRLKYENELALRQSVEADINGLRRVL
DELTLSKTDLEMQIESLNEELAYMKKNHEEEMKEFS-NQV---VGQVNVEMD-ATPGIDL
TRVLAEMREQYEAMAERNRRDAEEWFHAKSAELNKEVSTNTAMIQTSKTEITELRRTLQG
LEIELQSQLSMKAGLENTVAETECRYALQLQQIQGLISSIEAQLSELRSEMECQNQEYKM
LLDIKTRLEQEIATYRSLL-EGQ
>Human_K14
EKVTMQNLNDRLASYLDKVRALEEANADLEVKIRDWYQRQRP-----AEI-KDYSPY--F
KT-IEDLRNKILTATVDNANVLLQIDNARLAADDFRTKYETELNLRMSVEADINGLRRVL
DELTLARADLEMQIESLKEELAYLKKNHEEEMNALR-GQV---GGDVNVEMD-AAPGVDL
SRILNEMRDQYEKMAEKNRKDAEEWFFTKTEELNREVATNSELVQSGKSEISELRRTMQN
LEIELQSQLSMKASLENSLEETKGRYCMQLAQIQEMIGSVEEQLAQLRCEMEQQNQEYKI
LLDVKTRLEQEIATYRRLL-EGE
>Human_K15
EKITMQNLNDRLASYLDKVRALEEANADLEVKIHDWYQKQTPT----SPE-CDYSQY--F
KT-IEELRDKIMATTIDNSRVILEIDNARLAADDFRLKYENELALRQGVEADINGLRRVL
DELTLARTDLEMQIEGLNEELAYLKKNHEEEMKEFS-SQL---AGQVNVEMD-AAPGVDL
TRVLAEMREQYEAMAEKNRRDVEAWFFSKTEELNKEVASNTEMIQTSKTEITDLRRTMQE
LEIELQSQLSMKAGLENSLAETECRYATQLQQIQGLIGGLEAQLSELRCEMEAQNQEYKM
LLDIKTRLEQEIATYRSLL-EGQ
>Human_K16
EKVTMQNLNDRLASYLDKVRALEEANADLEVKIRDWYQRQRP-----SEI-KDYSPY--F
KT-IEDLRNKIIAATIENAQPILQIDNARLAADDFRTKYEHELALRQTVEADVNGLRRVL
DELTLARTDLEMQIEGLKEELAYLRKNHEEEMLALR-GQT---GGDVNVEMD-AAPGVDL
SRILNEMRDQYEQMAEKNRRDAETWFLSKTEELNKEVASNSELVQSSRSEVTELRRVLQG
LEIELQSQLSMKASLENSLEETKGRYCMQLSQIQGLIGSVEEQLAQLRCEMEQQSQEYQI
LLDVKTRLEQEIATYRRLL-EGE
>Human_K17
EKATMQNLNDRLASYLDKVRALEEANTELEVKIRDWYQRQAP-----GPA-RDYSQY--Y
RT-IEELQNKILTATVDNANILLQIDNARLAADDFRTKFETEQALRLSVEADINGLRRVL
DELTLARADLEMQIENLKEELAYLKKNHEEEMNALR-GQV---GGEINVEMD-AAPGVDL
SRILNEMRDQYEKMAEKNRKDAEDWFFSKTEELNREVATNSELVQSGKSEISELRRTMQA
```

```
LEIELQSQLSMKASLEGNLAETENRYCVQLSQIQGLIGSVEEQLAQLRCEMEQQNQEYKI
LLDVKTRLEQEIATYRRLL-EGE
>Human_K18
EKETMQSLNDRLASYLDRVRSLETENRRLESKIREHLEKKG------PQV-RDWSHY--F
KI-IEDLRAQIFANTVDNARIVLQIDNARLAADDFRVKYETELAMRQSVENDIHGLRKVI
DDTNITRLQLETEIEALKEELLFMKKNHEEEVKGLQ-AQI--ASSGLTVEVD-APKSQDL
AKIMADIRAQYDELARKNREELDKYWSQQIEESTTVVTTQSAEVGAAETTLTELRRTVQS
LEIDLDSMRNLKASLENSLREVEARYALQMEQLNGILLHLESELAQTRAEGQRQAQEYEA
LLNIKVKLEAEIATYRRLLEDGE
>Human_K19
EKLTMQNLNDRLASYLDKVRALEAANGELEVKIRDWYQKQGP-----GPS-RDYSHY--Y
TT-IQDLRDKILGATIENSRIVLQIDNARLAADDFRTKFETEQALRMSVEADINGLRRVL
DELTLARTDLEMQIEGLKEELAYLKKNHEEEISTLR-GQV---GGQVSVEVD-SAPGTDL
AKILSDMRSQYEVMAEQNRKDAEAWFTSRTEELNREVAGHTEQLQMSRSEVTDLRRTLQG
LEIELQSQLSMKAALEDTLAETEARFGAQLAHIQALISGIEAQLGDVRADSERQNQEYQR
LMDIKSRLEQEIATYRSLL-EGQ
>Human_K20
EKMAMQNLNDRLASYLEKVRTLEQSNSKLEVQIKQWYETNAP-----RAG-RDYSAY--Y
RQ-IEELRSQIKDAQLQNARCVLQIDNAKLAAEDFRLKYETERGIRLTVEADLQGLNKVF
DDLTLHKTDLEIQIEELNKDLALLKKEHQEEVDGLH-KHL---GNTVNVEVD-AAPGLNL
GVIMNEMRQKYEVMAQKNLQEAKEQFERQTAVLQQQVTVNTEELKGTEVQLTELRRTSQS
LEIELQSHLSMKESLEHTLEETKARYSSQLANLQSLLSSLEAQLMQIRSNMERQNNEYHI
LLDIKTRLEQEIATYRRLL-EGE
>Human_K23
GKATMQNLNDRLASYLEKVRALEEANMKLESRILKWHQQRDP-----GSK-KDYSQY--E
EN-ITHLQEQIVDGKMTNAQIILLIDNARMAVDDFNLKYENEHSFKKDLEIEVEGLRRTL
DNLTIVTTDLEQEVEGMRKELILMKKHHEQEMEKHH-VPS---DFNVNVKVD-TGPREDL
IKVLEDMRQEYELIIKKKHRDLDTWYKEQSAAMSQEAASPA-TVQSRQGDIHELKRTFQA
LEIDLQTQYSTKSALENMLSETQSRYSCKLQDMQEIISHYEEELTQLRHELERQNNEYQV
LLGIKTHLEKEITTYRRLL-EGE
>Human_K24
EKQTMQNLNDRLANYLDKVRALEEANTDLENKIKEWYDKYGPGSGDGGSG-RDYSKY--Y
SI-IEDLRNQIIAATVENAGIILHIDNARLAADDFRLKYENELCLRQSVEADINGLRKVL
DDLTMTRSDLEMQIESFTEELAYLRKNHEEEMKNMQ-GSS---GGEVTVEMN-AAPGTDL
TKLLNDMRAQYEELAEQNRREAEERFNKQSASLQAQISTDAGAATSAKNEITELKRTLQA
LEIELQSQLAMKSSLEGTLADTEAGYVAQLSEIQTQISALEEEICQIWGETKCQNAEYKQ
LLDIKTRLEVEIETYRRLL-DGE
>Human_K25
EKVTMQNLNDRLASYLDSVHALEEANADLEQKIKGWYEKFGPGSCR-GLD-HDYSRY--F
PI-IDDLKNQIIASTTSNANAVLQIDNARLTADDFRLKYENELALHQSVEADVNGLRRVL
DEITLCRTDLEIQYETLSEEMTYLKKNHKEEMQVLQ-CAA---GGNVNVEMN-AAPGVDL
TVLLNNMRAEYEALAEQNRRDAEAWFNEKSASLQQQISEDVGATTSARNELTEMKRTLQT
LEIELQSLLATKHSLECSLTETESNYCAQLAQIQAQIGALEEQLHQVRTETEGQKLEYEQ
LLDIKLHLEKEIETYCLLI-GGD
>Human_K26
EKVTMQNLNDRLASYLDHVHALEEANADLEQKIKGWYEKCEPGSSR-EHD-HDYSRY--F
SV-IEDLKRQIISATICNASIVLQNDNARLTADDFRLKYENELALHHSVEADTSGLRRVL
DELTLCTTDLEIQCETLSEELTYLKKSHEEEMEVLQ-YTA---GGNVNVEMN-ATPGVDL
TVLLNNMRAEYEDLAEQNRKDAEAWFNERSATLQQQISDHEGAATAARNELTELKRNLQT
LEIELQSLMAVKHSYECSLAETEGNYCNQLQQIQDQIGVMEEQLQQIRTETEGQKLEYEQ
LLDVKIFLEKEIDIYCNLL-DGE
>Human_K27
EKVTMQNLNDRLASYLENVRALEEANADLEQKIKGWYEKFGPGSCR-GLD-HDYSRY--F
PI-IDELKNQIISATTSNAHVVLQNDNARLTADDFRLKFENELALHQSVEADINGLRRVL
DELTLCRTDLEIQLETLSEELAYLKKNHEEEMKALQ-CAA---GGNVNVEMN-AAPGVDL
TVLLNNMRAEYEALAEQNRRDAEAWFNEKSASLQQQISDDAGATTSARNELIEMKRTLQT
LEIELQSLLATKHSLECSLTETESNYCAQLAQIQAQIGALEEQLHQVRTETEGQKLEYEQ
LLDIKVHLEKEIETYCLLI-DGE
>Human_K28
EKVTMQNLNDRLASYLDNVRALEEANAELERKIKGWYEKYGPGSCR-GLD-HDYSRY--H
LT-IEDLKNKIISSTTTNANVILQIDNARLAADDFRLKYENELTLHQNVEADINGLRRVL
DELTLCRTDQELQYESLSEEMTYLKKNHEEEMKALQ-CAA---GGNVNVEMN-AAPGVDL
AVLLNNMRAEYEALAEQNRKDAEAWFNEKSASLQQQISHDSGAATFARSQLTEMRRTLQT
LEIQLQSLMATKHSLECSLTETESNYCTQLAQIQAQIGALEEQLHQVRTETEGQKLEYEH
LLDVKVHLEKEIETYCRLI-DGD
>Human_K31
EKETMQFLNDRLASYLEKVRQLERDNAELENLIRERSQQQEP-----LLC-PSYQSY--F
KT-IEELQQKILCTKSENARLVVQIDNAKLAADDFRTKYQTELSLRQLVESDINGLRRIL
DELTLCKSDLEAQVESLKEELLCLKSNHEQEVNTLR-CQL---GDRLNVEVD-AAPTVDL
NRVLNETRSQYEALVETNRREVEQWFTTQTEELNKQVVSSSEQLQSYQAEIIELRRTVNA
LEIELQAQHNLRDSLENTLTESEARYSSQLSQVQSLITNVESQLAEIRSDLERQNQEYQV
LLDVRARLECEINTYRSLL-ESE
>Human_K32
EKETMQFLNDRLASYLTRVRQLEQENAELESRIQEASHSQVL-----TMT-PDYQSH--F
RT-IEELQQKILCTKAENARMVVNIDNAKLAADDFRAKYEAELAMRQLVEADINGLRRIL
DDLTLCKADLEAQVESLKEELMCLKKNHEEEVGSLR-CQL---GDRLNIEVD-AAPPVDL
TRVLEEMRCQYEAMVEANRRDVEEWFNMQMEELNQQVATSSEQLQNYQSDIIDLRRTVNT
LEIELQAQHSLRDSLENTLTESEARYSSQLAQMQCMITNVEAQLAEIRADLERQNQEYQV
LLDVRARLEGEINTYRSLL-ENE
>Human_K33a
EKETMQFLNDRLASYLEKVRQLERDNAELENLIRERSQQQEP-----LVC-ASYQSY--F
KT-IEELQQKILCSKSENARLVVQIDNAKLASDDFRTKYETELSLRQLVESDINGLRRIL
DELTLCRSDLEAQVESLKEELLCLKQNHEQEVNTLR-CQL---GDRLNVEVD-AAPTVDL
NQVLNETRSQYEALVETNRREVEQWFATQTEELNKQVVSSSEQLQSYQAEIIELRRTVNA
LEIELQAQHNLRDSLENTLTESEARYSSQLSQVQRLITNVESQLAEIRSDLERQNQEYQV
LLDVRARLECEINTYRSLL-ESE
>Human_K33b
EKETMQFLNDRLASYLEKVRQLERDNAELENLIRERSQQQEP-----LLC-PSYQSY--F
KT-IEELQQKILCSKSENARLVVQIDNAKLAADDFRTKYQTEQSLRQLVESDINSLRRIL
DELTLCRSDLEAQMESLKEELLSLKQNHEQEVNTLR-CQL---GDRLNVEVD-AAPAVDL
NQVLNETRNQYEALVETNRREVEQWFATQTEELNKQVVSSSEQLQSYQAEIIELRRTVNA
LEIELQAQHNLRYSLENTLTESEARYSSQLSQVQSLITNVESQLAEIRSDLERQNQEYQV
LLDVRARLECEINTYRSLL-ESE
>Human_K34
EKETMQFLNDRLASYLEKVRQLERDNAELEKLIQERSQQQEP-----LLC-PSYQSY--F
KT-IEELQQKILCAKAENARLVVNIDNAKLASDDFRSKYQTEQSLRLLVESDINSIRRIL
DELTLCKSDLESQVESLREELICLKKNHEEEVNTLR-SQL---GDRLNVEVD-TAPTVDL
NQVLNETRSQYEALVEINRREVEQWFATQTEELNKQVVSSSEQLQSCQAEIIELRRTVNA
LEIELQAQHNLRDSLENTLTESEAHYSSQLSQVQSLITNVESQLAEIRCDLERQNQEYQV
LLDVRARLECEINTYRSLL-ESE
>Human_K35
EKETMQSLNDRLAGYLEKVRQLEQENASLESRIREWCEQQVP-----YMC-PDYQSY--F
RT-IEELQKKTLCSKAENARLVVEIDNAKLAADDFRTKYETEVSLRQLVESDINGLRRIL
DDLTLCKSDLEAQVESLKEELLCLKKNHEEEVNSLR-CQL---GDRLNVEVD-AAPPVDL
```

```
NRVLEEMRCQYETLVENNRRDAEDWLDTQSEELNQQVVSSSEQLQSCQAEIIELRRTVNA
LEIELQAQHSMRDALESTLAETEARYSSQLAQMQCMITNVEAQLAEIRADLERQNQEYQV
LLDVRARLECEINTYRGLL-ESE
>Human_K36
EKETMQFLNDRLANYLEKVRQLERENAELESRIQEWYEFQIP-----YIC-PDYQSY--F
KT-IEDFQQKILLTKSENARLVLQIDNAKLAADDFRTKYETELSLRQLVEADINGLRRIL
DELTLCKADLEAQVESLKEELMCLKKNHEEEVSVLR-CQL---GDRLNVEVD-AAPPVDL
NKILEDMRCQYEALVENNRRDVEAWFNTQTEELNQQVVSSSEQLQCCQTEIIELRRTVNA
LEIELQAQHSMRNSLESTLAETEARYSSQLAQMQCLISNVEAQLSEIRCDLERQNQEYQV
LLDVKARLEGEIATYRHLL-EGE
>Human_K37
EKETMKFLNDRLANYLEKVRQLEQENAELETTLLERSKCHES-----TVC-PDYQSY--F
RT-IEELQQKILCSKAENARLIVQIDNAKLAADDFRIKLESERSLHQLVEADKCGTQKLL
DDATLAKADLEAQQESLKEEQLSLKSNHEQEVKILR-SQL---GEKFRIELD-IEPTIDL
NRVLGEMRAQYEAMVETNHQDVEQWFQAQSEGISLQAMSCSEELQCCQSEILELRCTVNA
LEVERQAQHTLKDCLQNSLCEAEDRYGTELAQMQSLISNLEEQLSEIRADLERQNQEYQV
LLDVKARLENEIATYRNLL-ESE
>Human_K38
EKETMQFLNDRLANYLEKVRQLEQENAELEATLLERSKCHES-----TVC-PDYQSY--F
HT-IEELQQKILCSKAENARLIVQIDNAKLAADDFRIKLESERSLRQLVEADKCGTQKLL
DDATLAKADLEAQQESLKEEQLSLKSNHEQEVKILR-SQL---GEKLRIELD-IEPTIDL
NRVLGEMRAQYEAMLETNRQDVEQWFQAQSEGISLQDMSCSEELQCCQSEILELRCTVNA
LEVERQAQHTLKDCLQNSLCEAEDRFGTELAQMQSLISNVEEQLSEIRADLERQNQEYQV
LLDVKTRLENEIATYRNLL-ESE
>Human_K39
EKETMQILNERLANYLQKVRMLERENAELESKIQEESNKELP-----VLC-PDYLSY--Y
TT-IEELQQKILCTKAENSRLVSQIDNTKLTADDLRAKYEAEVSLRQLVESDANGLKQIL
NVLTLGKADLEAQVQSLKEELLCLKNNHKEEINSLQ-CQL---GERLDIEVT-AAPSADL
NQVLQEMRCQYEPIMETNRKDVEQWFNTQIEELNQQVVTSSQQQQCCQKEIIELRRSVNT
LEVELQAQHRMRDSQECILTETEARYTALLTQIQSLIDNLEAQLAEIRCALERQNQEYEI
LLDVKSRLECEITTYRSLL-ESS
>Human_K40
EKETMQFLNDRLASYLEKVRSLEETNAELESRIQEQCEQDIP-----MVC-PDYQRY--F
NT-IEDLQQKILCTKAENSRLAVQLDNCKLATDDFKSKYESELSLRQLLEADISSLHGIL
EELTLCKSDLEAHVESLKEDLLCLKKNHEEEVNLLR-EQL---GDRLSVELD-TAPTLDL
NRVLDEMRCQCETVLANNRREAEEWLAVQTEELNQQQLSSAEQLQGCQMEILELKRTASA
LEIELQAQQSLTESLECTVAETEAQYSSQLAQIQCLIDNLENQLAEIRCDLERQNQEYQV
LLDVKARLEGEINTYWGLL-DSE
```

Suppl. Fig. S14. Amino acid sequence alignment of the intermediate filament domains of keratins (type I and type II combined) used for the phylogenetic analysis shown in Supplementary Figure S5. The multiple sequence alignment is shown in fasta_aln format.
